# Phylogeny of teleost connexins reveals highly inconsistent intra- and interspecies use of nomenclature and misassemblies in recent teleost chromosome assemblies

**DOI:** 10.1186/s12864-020-6620-2

**Published:** 2020-03-11

**Authors:** Svein-Ole Mikalsen, Marni Tausen, Sunnvør í Kongsstovu

**Affiliations:** 1 Faculty of Science and Technology, University of the Faroe Islands, Vestara Bryggja 15, FO-100 Tórshavn, Faroe Islands; 2 Present affiliation: Bioinformatics Research Centre, Aarhus University, C. F. Møllers Allé 8, 8000 Aarhus C, Denmark; 3 Amplexa Genetics A/S, Hoyvíksvegur 51, FO-100 Tórshavn, Faroe Islands

**Keywords:** Connexins, Genome duplication, Mammals, Nomenclature, Ohnologs, Orthologs, Paralogs, Phylogenetic trees, Teleosts

## Abstract

**Background:**

Based on an initial collecting of database sequences from the gap junction protein gene family (also called connexin genes) in a few teleosts, the naming of these sequences appeared variable. The reasons could be (i) that the structure in this family is variable across teleosts, or (ii) unfortunate naming. Rather clear rules for the naming of genes in fish and mammals have been outlined by nomenclature committees, including the naming of orthologous and ohnologous genes. We therefore analyzed the connexin gene family in teleosts in more detail. We covered the range of divergence times in teleosts (eel, Atlantic herring, zebrafish, Atlantic cod, three-spined stickleback, Japanese pufferfish and spotted pufferfish; listed from early divergence to late divergence).

**Results:**

The gene family pattern of connexin genes is similar across the analyzed teleosts. However, (i) several nomenclature systems are used, (ii) specific orthologous groups contain genes that are named differently in different species, (iii) several distinct genes have the same name in a species, and (iv) some genes have incorrect names. The latter includes a human connexin pseudogene, claimed as *GJA4P*, but which in reality is *Cx39.2P* (a delta subfamily gene often called *GJD2like*). We point out the ohnologous pairs of genes in teleosts, and we suggest a more consistent nomenclature following the outlined rules from the nomenclature committees. We further show that connexin sequences can indicate some errors in two high-quality chromosome assemblies that became available very recently.

**Conclusions:**

Minimal consistency exists in the present practice of naming teleost connexin genes. A consistent and unified nomenclature would be an advantage for future automatic annotations and would make various types of subsequent genetic analyses easier. Additionally, roughly 5% of the connexin sequences point out misassemblies in the new high-quality chromosome assemblies from herring and cod.

## Background

Large-scale sequencing techniques developed since the turn of the century have caused a virtual explosion of species with sequenced genomes. A critical part of making all these genomes useful is the process of annotation, of which gene identification and gene naming are indispensable parts [1–3]. Computerized annotation by algorithms and the use of previously identified sequences available in databanks are needed to keep up with the flow of new genomes. However, computerized annotations are only as good as the assumptions behind the algorithms and the available data, including identifications, allow.

The Human Gene Nomenclature Committee states as the first point in its summary guidelines that “each approved gene symbol must be unique” [4]. Some general principles of naming genes in zebrafish (and by extension in other teleosts) are outlined by the Zebrafish Information Network [5]. The Zebrafish Nomenclature Conventions states that “genes should be named after the mammalian ortholog whenever possible” [5]. We here understand orthologs in the same meaning as originally defined by Fitch [6, 7], who divided homologs into two main classes: orthologs and paralogs. In simple terms, orthologs are the same genes in different species. All the other genes in a gene family are paralogs, whether intraspecies or interspecies. Note that in this context, the functional relationship or expression pattern is irrelevant (in contrast to some deviant definitions of orthologs, for example on p. 726 in ref. [8]). Thus, a pseudogene in one species can be an ortholog of a functional gene in another species, even if the pseudogene has no known function or is not expressed.

Giving unique names to unique genes [4] and naming teleost genes according to the mammalian ortholog [5] appear as sound principles. The Zebrafish Nomenclature Conventions details that in the case of duplicated genes resulting from genome duplication, “symbols for the two zebrafish genes should be the same as the approved symbol of the human or mouse ortholog followed by “a” or “b” to indicate that they are duplicated copies” [5]. In the case of tandem gene duplication, the duplicates “with a single mammalian ortholog should have gene symbols appended with a .1, .2, using the same symbol as the mammalian ortholog” [5]. This may not always be easy to establish unequivocally, as it requires much work and there may be a long time between the initial genome assembly and the complete genome being assembled into chromosomes. A good indication of orthology may come from phylogenetic analyses.

Of course, reality is often not simple, as both genome duplications, tandem gene duplications, gene losses, the formation of pseudogenes, retrotranscription and reinsertion, and other genetic events may have occurred since the evolutionary separation of the different species in question. Two genome duplications occurred during the early evolution of vertebrates after the divergence of the urochordates [9–11]. These genome duplications are common to both teleosts and tetrapods. Additionally, another genome duplication occurred in the early evolution of teleosts [12–14].

The pairs of genes created by genome duplication are called ohnologs [15, 16]. As such, ohnologs are a specific subgroup of paralogs [6, 7]. Being on different chromosomes, different genetic events may happen for each member of an ohnologous pair, such as mutations of various kinds, gene losses, tandem gene duplication at one of the sites, etc. It is therefore not necessarily a 1:1 relationship between ohnologs in teleosts (e.g., one of the ohnologs could be lost in one or several species), or between mammalian and teleost orthologs [6, 7]. Furthermore, the synteny (the linear order of genetic elements in DNA) can be muddled. Adding to this evolutionary genetic complexity, there are also technical and bioinformatic caveats, making complete and perfect genome assemblies unlikely. Presently, the published genome assemblies are often estimated to be around 90% complete [17, 18], being in thousands of scaffolds instead of a few tens of chromosomes. Moreover, numerous kinds of assembly errors [19, 20] can further complicate the annotation process.

It was early observed that certain gene families had unusually large number of members in fish model species [21]. One of these gene families is the gap junction protein gene family, encoding the proteins called connexins (for simplicity, we will generally refer to the genes as connexin genes). This family has approximately twice as many members in teleost species as in other vertebrates [22–24], and as such has retained more than its fair share of genes generated by genome duplication compared with many other gene families, which generally retain 1 to 20% of the duplicated genes (see review by Glasauer and Neuhauss [25]).

Both a size-based (in kiloDalton) nomenclature and a Greek nomenclature have been used in naming the genes in this family (e.g., *connexin43*, abbreviated *cx43*, in the size nomenclature is the same as *gap junction protein alpha 1 gene*, abbreviated *gja1*, in the Greek nomenclature). A disadvantage with a size-based nomenclature is that the protein size may vary in different species, and thus the relationship with the corresponding genes/proteins in other species may not be immediately clear. The Greek nomenclature divides the group into the subfamilies alpha (gja), beta (gjb), gamma (gjc), delta (gjd) and epsilon (gje) and with a number that initially stated the chronology of gene detection. The Human and Mouse Gene Nomenclature Committees have decided to use the Greek gene nomenclature for the connexin genes. A novel connexin nomenclature was very recently suggested by Premzl [26], but only mammals were taken into consideration.

The connexin genes are chordate-specific genes, urochordates being the most primitive organisms having these genes [24, 27], which in the vertebrates have evolved into the distinct subfamilies [22, 24, 28, 29]. The connexin proteins are transmembrane molecules that aggregate into hexamers forming a pore through the membrane, often called a hemichannel. Traditionally, it was supposed that hemichannels would not act alone, but rather line up with a corresponding hemichannel from the neighboring cell to form a channel directly from the cytosol in one cell to the cytosol in the other cell, through which small water-soluble molecules and ions can diffuse [30]. In some tissues, such as the heart and uterus, these channels are of utmost importance for passing the electrical impulse from cell to cell, making these organs contract in a synchronized manner [31, 32]. The channels are probably also involved in cellular homeostasis and growth control [33], possibly through interactions with numerous proteins involved in signaling and regulation [34–36]. Additionally, there are now strong indications that hemichannels are functional in their own right [37–39].

The teleosts are the most species-rich group among vertebrates. In connection with the sequencing and assembly of the Atlantic herring genome [40], we collected some teleost connexin sequences, and soon noticed that the naming appeared variable. The two most obvious explanations for the variability were (i) that the structure in this family is variable across the teleosts, or (ii) unfortunate naming. We therefore examined the connexins in teleost species more closely, and selected species spanning the range of divergence times in this vertebrate group [41, 42]. A genome duplication occurred at the basis of the teleosts ~ 350 million years ago, and the Elopomorpha (to which eels belong) was the first group to diverge ~ 300 million years ago, and hence we selected Japanese eel (*Anguilla japonica*), partly supported by European and American eel (*Anguilla anguilla* and *Anguilla rostrata*) [43–46]. The Clupeiformes, to which Atlantic herring (*Clupea harengus*) [17, 40] belongs, and Cypriniformes, to which zebrafish (*Danio rerio*) [47] belongs, had a common divergence ~ 250 million years ago, and soon after (~ 240 million years ago) split into separate groups. The Acantomorphata diverged ~ 150 million years ago, and later split into several subgroups, of which the Gadiformes, to which Atlantic cod (*Gadus morhua*) [48] belongs, is one. The Perciformes, to which three-spined stickleback (*Gasterosteus aculeatus*) [49] belongs, diverged ~ 100 million years ago. The Tetraodontiformes (pufferfishes) are among the most recently diverged groups, ~ 70 million years ago, and both Japanese pufferfish (*Takifugu rubripes,* often called *Fugu rubripes*, and here called Fugu) and green spotted pufferfish (*Tetraodon nigroviridis,* here called Tetraodon) are members of this group. The two pufferfishes have very condensed genomes compared with most other teleosts [50, 51].

As the genes should be named after the mammalian ortholog whenever possible [52], the connexin sequences from several mammals were included. The sequences were analyzed phylogenetically, using the names indicated in the databases whenever possible. Our results show that a considerable degree of inconsistency exists in the naming of the connexin genes in fish species. There is even a case of inconsistent naming among the human sequences. In our opinion, making the naming in this gene family more congruent and consistent is indeed possible, which will improve the quality and usefulness of future genome annotations.

## Results

### The structure of the teleost gap junction protein gene family

**Fig. 1:**
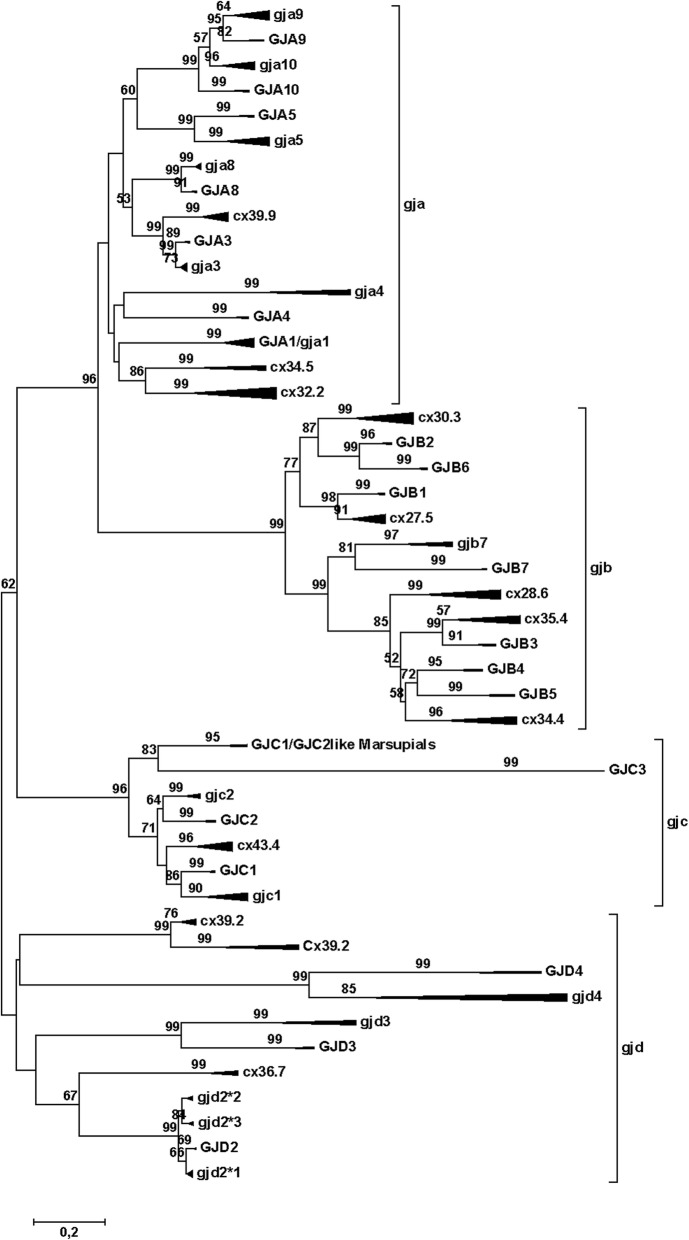
Phylogenetic tree for the gap junction protein (connexin) gene family. The mammalian branches are indicated by upper case letters; teleost branches are indicated by lower case letters. The width of the triangles indicates the number of taxa included in the branch, and the length of the triangles indicates the sequence variation within the branch. The tree was made by the Minimum Evolution method, using amino acids (354 amino acid sequences with 201 positions in the final dataset) and the Dayhoff substitution matrix. The bootstrap values (500 replicates) > 50% are shown next to the branches. To avoid disruptive long-branch attraction, some sequences were excluded (see text). This model gives results that are quite close to the majority of results as summed up in Suppl. Table 1, and thus is close to an average tree from all the phylogenetic analyses. The major difference is that the mammalian *GJA10* and teleost *gja10* have switched places. In the original three, the root of the *GJD* family splits up in three very close branches, but using the rooting function in the Mega Tree Explorer, they were collected them into one common basal branch. Note the commonly occurring dichotomy with the mammalian sequences in one of the sub-branches and the teleost sequences in the other sub-branch, although some of the teleost groups do not have a mammalian counterpart (and vice versa). The scale bar (lower left) indicates the number of amino acid substitutions per site

**Fig. 2:**
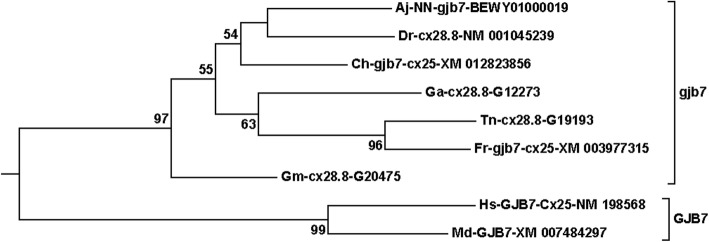
The *GJB7*/*gjb7* branch from the compressed tree shown in Fig. 1. This is an example of a group where all teleost species have only one member, and therefore probably have lost the expected ohnolog partner at a very early stage before the divergence of the different teleosts, similar to most of the other connexins located on the same chromosome (see Table 2). The naming of the sequences is as follows. The two-letter abbreviation indicates the species (Aj, *Anguilla japonica* = Japanese eel; Dr., *Danio rerio* = zebrafish; Ch, *Clupea harengus* = Atlantic herring; Ga, *Gasterosteus aculeatus =* three-spined stickleback; Tn, *Tetraodon nigroviridis* = green spotted pufferfish; Fr, *Takifugu rubripes* = Fugu (Japanese pufferfish); Gm, *Gadus morhua* = Atlantic cod; Hs, *Homo sapiens* = human; Md, *Monodelphis domestica* = opossum) followed by an abbreviation of the name of the sequence in the database (using upper and lower case letters as indicated in the database), and finally, the accession number in the database. NN indicates that there was No Name for the sequence in the database. Further details about the naming are found in the Methods section

**Fig. 3:**
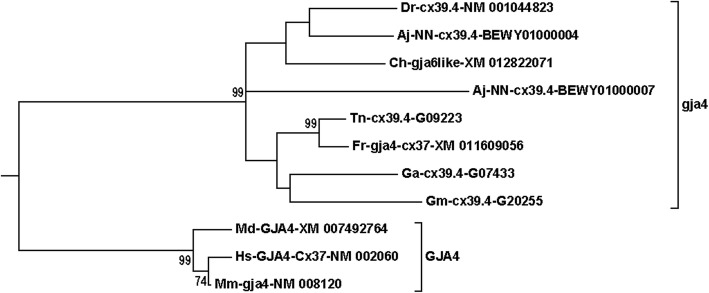
The *GJA4*/*gja4* branch from the compressed tree shown in Fig. 1. This is an example of a group where eel (Aj) has two members, whereas all the other teleosts have one member. The eel pair is found on two different chromosomes (Table 2), suggesting that one member was lost somewhere in-between the divergence of eels and the other teleosts. Moreover, note that the herring (Ch) member is wrongly named *gja6like* in GenBank; the correct name would be *gja4*. See legend of Fig. 2 and Methods section for details about naming of the sequences

**Fig. 4:**
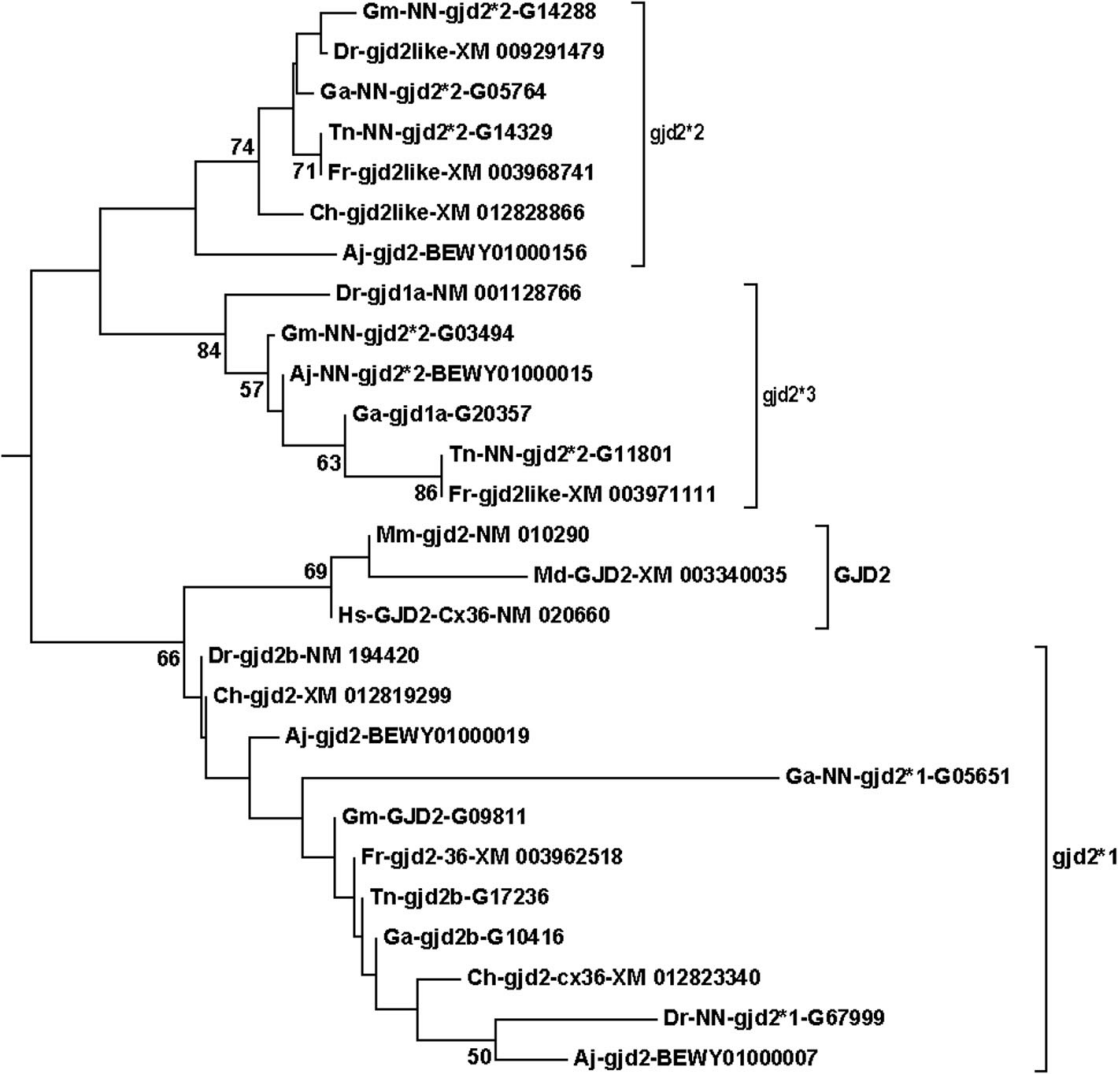
The *gjd2* branch from the compressed tree shown in Fig. 1. This is an example of a group where the structure is considerably more complex in teleosts than in mammals. First, there is one teleost group, here called gjd2*1, that in the majority of statistical models locates closest to mammalian *GJD2*. Gjd2*1 contains two sequences from most fishes, and each members of the pairs are on different chromosomes in all species (Table 2). Secondly, there are two subgroups (here called gjd2*2 and gjd2*3) that are, according to this statistical model, slightly more distantly connected to mammalian *GJD2*. In this statistical model, the gjd2*2 and gjd2*3 subgroups have a phylogenetic distribution that is “ohnologically perfect” in that it divides into two subgroups containing one sequence from each species. In all species, the pairs of sequences are found on two different chromosomes (Suppl. Table 7). See legend of Fig. 2 and Methods section for details about naming of the sequences

**Fig. 5:**
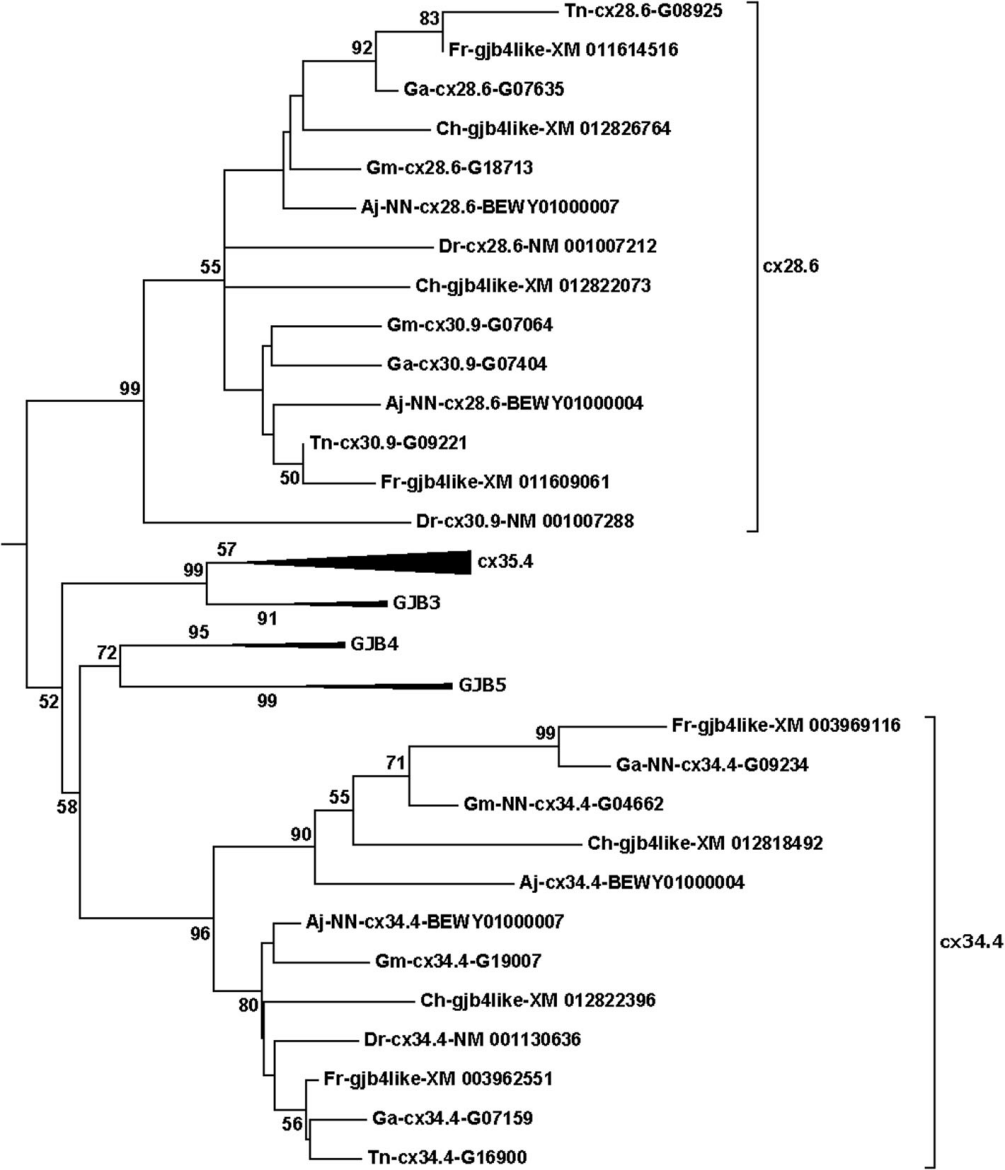
*GJB3*/*GJB4*/*GJB5* related sequences from the compressed tree shown in Fig. 1. This is an example where teleost sequences with the same names are found in clearly distinct branches of the tree. In this case, four Fugu (Fr) and four herring (Ch) sequences are called *gjb4like*. Two sequences from each species located into each of the two groups here called *cx28.6* and *cx34.4*. Note also that mammalian *GJB4* and *GJB5* were always found as a dichotomous pair, and that *cx34.4* never mixed into the dichotomous *GJB4*/*GJB5* pair (Suppl. Table 1). Similarly, *cx28.6* generally split off at the foot of the collected *GJB3*/*GJB4*/*GJB5*/*cx35.5*/*cx34.4* clade, but in a few cases (with poorer statistics) was positioned closer to *GJB3*/*cx35.4* (Suppl. Table 1). Thus, there is no evidence to support *cx28.6* or *cx34.4* being more closely related to *GJB4* than to *GJB5* as the naming (*gjb4like*) could suggest. See legend of Fig. 2 and Methods section for details about naming of the sequences

**Fig. 6:**
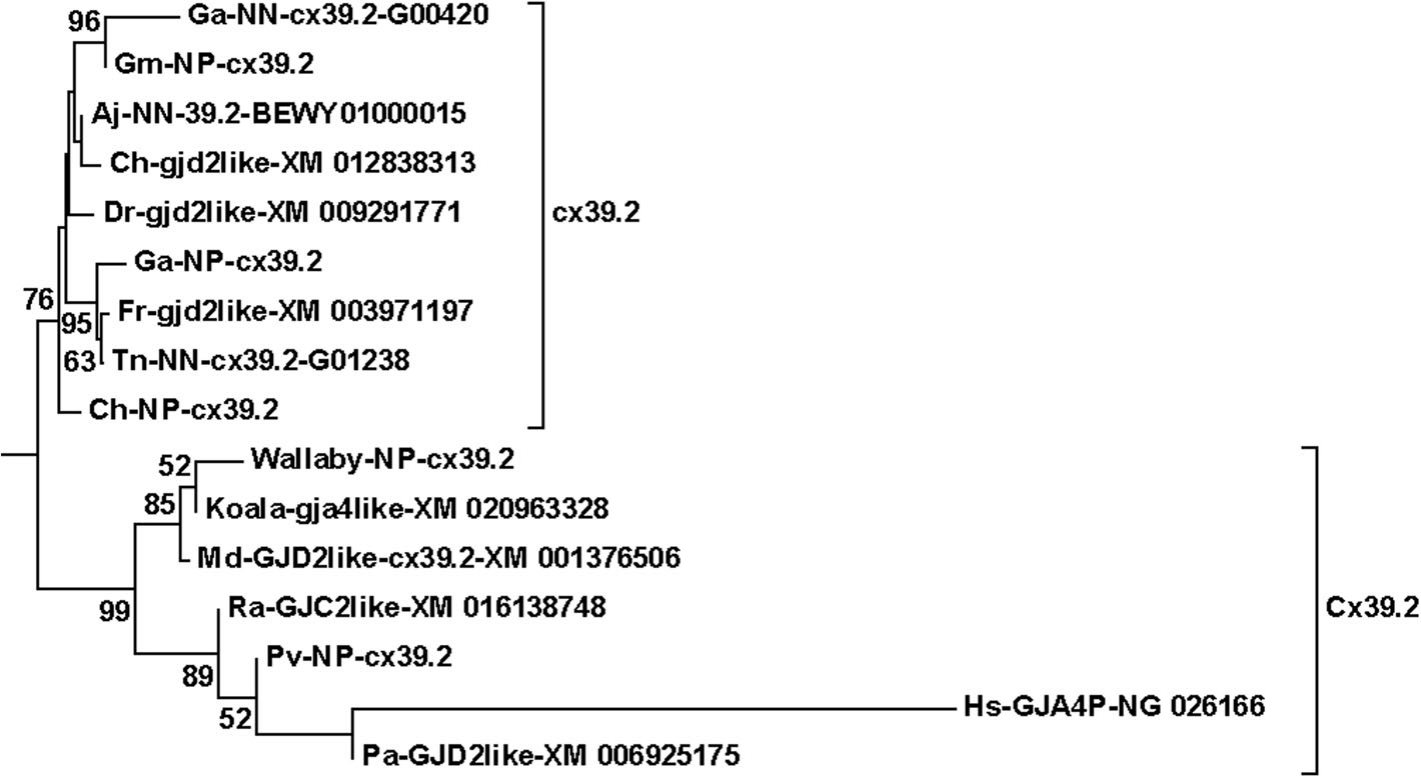
The human pseudogene “*GJA4P*” (NG_02166) always located together with *cx39.2*/*gjd2like* sequences. Note that these “*gjd2like”* sequences must not be confused with paralogous sequences that have the same name in other groups (*cx36.7* and gjd2*2). See legend of Fig. 2 and Methods section for details about naming of the sequences

The compressed tree with the connexin subfamilies for teleosts and mammals is shown in Fig. 1. All sequences involved are shown in Suppl. Fig. 1–12. A few of the expanded branches are shown in Figs. 2-6 (Fig. 2, *gjb7*; Fig. 3, *gja4*; Fig. 4, *gjd2*; Fig. 5, the “*gjb4like*” complex; Fig. 6, *cx39.2*), and the remaining branches are shown in Suppl. Fig. 14.^1^ In this tree, and in all trees made for the major statistical analyses (Suppl. Table 1), the *GJE1*/*gje1*/*cx23* group was omitted, because the inclusion of the *GJE1* orthologous group caused long-branch attraction [53, 54]. In fact, the long-branch attraction was so intense that it ripped apart both the delta and gamma subfamilies, and caused the highly variable groups of *GJC3* and *GJD4* to locate in the vicinity of the *GJE1* group (compare Fig. 1 and Suppl. Fig. 15). However, we did include a human pseudogene in the *Cx39.2* group (Fig. 6), but not the corresponding pseudogenes from some other mammals (Suppl. Fig. 12). This orthologous group is further discussed below. We also excluded rodent *gja6* (which is the ortholog of the human pseudogene sometimes called *Cx43pX* [29]) and a cod *gjd2* sequence (Gm-NN-gjd2*1-G01582). This sequence often split out from its expected *gjd2* group, and we excluded it to make clearer distinctions within the different *gjd2* groups.

Overall, it was evident that the structure of the connexin gene family was similar across all the teleosts. There were examples of species-specific gene duplications or lack of genes, but at the present time we cannot with certainty ascribe all such “anomalies” to biological and genetic reality or to partial genome sequencing and/or erroneous genome assembly. The overall similarity should make it rather simple to extend the gene identifications to other teleost species when their genomes are sequenced, thereby easing their annotation. However, this is dependent on consistency in naming of the genes in the family, which is presently at lack as shown below.

### The mixture of nomenclatures

As can be seen in Figs. 2 to 6 (and also in Suppl. Fig. 14 and Suppl. Tables 3–6), there was often little consistency in naming within many of the gene clades, as some of the genes were named by the size nomenclature and others are named by the Greek nomenclature. We will here sum up the nomenclature for some of the teleost species. More details are found in the Supplementary Tables and Supplementary Figures.

Zebrafish is undoubtedly the most highly investigated teleost [47], with its genome sequencing starting in 2001, the first genome assemblies available in Ensembl around 2005, with the latest assemblies and annotations from 2017/2018 (Ensembl release 91, CRCz11). Thus, we would expect the gene nomenclature to be of good standard and being consistent with the intentions expressed in the Zebrafish Nomenclature Conventions [52]. In zebrafish, among the 38 unique and predicted genes present in GenBank (Suppl. Table 3 and Suppl. Fig. 5), 25 genes followed the size nomenclature and 13 genes followed the Greek nomenclature. The naming of 37 predicted genes in Ensembl was rather similar to GenBank, with 31 sequences having the same name as in Ensembl (Suppl. Table 3).

Fugu was the first teleost with its genome published [50], with the last genome assembly from 2011 (in Ensembl) and annotations from 2018 [55]. In July 2019, the Fugu annotation was updated in GenBank. Many of the previous GenBank predictions changed names from the combined Greek and size nomenclature (*gja1-cx43*) to Greek nomenclature only (*gja1*). In many cases the accession numbers also changed (Suppl. Table 4). After the GenBank update, three genes followed the size nomenclature and 38 genes followed Greek nomenclature. One previously predicted gene (Fr-*gja3like*-XM_003970457) was lost in the update. Fourteen genes can now be said to have the same naming in GenBank and Ensembl (disregarding upper/lower case letters, and considering *gja5a* = *gja5*), all in the Greek nomenclature (Suppl. Table 4). In Ensembl, 16 Fugu genes followed the Greek nomenclature, 21 genes followed size nomenclature, one gene had no name, and four genes were not predicted (Suppl. Table 4).

For cod sequences in Ensembl (Suppl. Fig. 10, Suppl. Table 5), eight followed Greek nomenclature (six in upper case and two in lower case), 18 followed size nomenclature, 17 were predicted but not named, and one was not predicted (but found by us). The recently available cod chromosome level genome assembly in GenBank [56] and the corresponding gene predictions provided us with the possibility to compare the naming of the new GenBank predictions with the Ensembl cod gene predictions (Suppl. Table 5). Only four sequences had been given the same name in Ensembl and GenBank (considering lower/upper case letters as identical; Suppl. Tables 4 and 6).

For the GenBank predictions in herring, 32 genes followed the Greek nomenclature, four followed the size nomenclature, and eight followed a mixed nomenclature, in addition to two non-predicted genes (one of them, *gja8*, considered as a part of an unrelated gene; Suppl. Fig. 9 and Suppl. Table 6). In the recent annotation from the novel chromosomal level assembly of herring added to the Ensembl database (Sept. 2019) [57], the predictions contained nine genes in Greek nomenclature and 20 genes in size nomenclature, in addition to 14 predicted genes with no name, and three genes that were lost, probably due to erroneous genome assembly (see below) (Suppl. Table 6). Only two genes had completely identical names in Ensembl and GenBank; four genes if upper/lower case letters and combination Greek-size nomenclatures were considered identical to the lower case Greek nomenclature.

Only a few of the eel connexins in the GenBank transcriptome shotgun assemblies had been named, with several having a hybrid nomenclature not commonly used (such as *CXA5*, *cxb1*, *CXG1*, etc.).

### Multiple names for a distinct ortholog within teleosts

There were three common inconsistencies within an orthologous group, two of which are considered in this section, and the third in the next section. The first was that some genes within the group are named according to the Greek nomenclature, and other genes according to the size nomenclature. For example, within the *GJB7* group (also called *connexin25* in mammals), some teleost sequences were named *gjb7* and other sequences were named *cx28.8,* and some combined the Greek and size nomenclature such as *gjb7-cx25* (Fig. 2).

The second inconsistency was that evident orthologs had been given different numbers in the Greek nomenclature. One example was the teleost orthologs for mammalian *GJA4*, also called *connexin37* (Fig. 3). They were called *gja4* in Fugu, *cx39.4* in Tetraodon, stickleback and zebrafish, and *gja6like* in Atlantic herring. It should be noted that *GJA6* is a different gene group that was generated by a mammalian-specific gene duplication of *GJA1* (*connexin43*), maybe by retrotransposition. *GJA6* is a pseudogene in humans and some other species (called connexin43-related pseudogene on the X chromosome, *Cx43pX*, in ref. [23, 29]). In other species, including rodents, dog and elephant, *GJA6* appears to be a functional gene [23, 29]. Another example is found within the major *GJD2* group (Fig. 2c). Zebrafish NM_001128766 and stickleback ENSGACG00000020357 (no GenBank entry) were both called *gjd1a*, whereas the orthologs in Fugu were both called *gjd2like* (Fig. 4).

### Distinct genes having identical names

The third common inconsistency was that clearly different sequences had the same name. In Fugu (using the GenBank sequences predicted before July 2019), there were two of each for *Cx32.2like*, *gjb1like*, *gjb2like*, and *gjb3like* genes; three *gja3like* and *gjc1like* genes; and four *gjb4like* and *gjd2like* genes (Fig. 4; Suppl. Table 4 and 7).

Atlantic herring (*Clupea harengus*) had its genome sequenced, assembled and annotated in GenBank in 2015 [17, 58], and a recently a chromosomal level assembly [59, 60] was annotated in Ensembl in the fall of 2019 [57]. Thus, the prediction and naming of the genes should describe much of the current status for automatic annotation. In the GenBank 2015 annotation, there were two of each for *gja5like*, *gjd2*, and *gjd3like*; three of *C*x*32.2*, *gjc1like* and *gjd2like* genes; and four genes called *gja3like* and *gjb4like* (Fig. 4, Suppl. Table 6 and 7). In the Ensembl annotation, each of the names occurred only once, but on the other hand, 14 of the gene predictions were un-named (Suppl. Table 6).

We will use teleost *gjd2like* and *gjd2* as examples. *Gjd2like* was used in several more or less closely related genes in the delta subfamily. More specifically, sequences with this name were found among the *cx36.7*, *cx39.2,* and the central *gjd2* groups. These groups are shortly discussed below.

The central *gjd2* group (Fig. 4) is a complex of sequences that are all closely related to the mammalian *GJD2*. Previously, these genes were named *connexin36* in mammals and *connexin35* or *connexin35.1* [61] in fish. While mammals have one *GJD2* gene, teleosts have up to four (as in zebrafish, Fugu, and stickleback) in this central *gjd2* group. For convenience, we named groups of the teleost genes in the central *gjd2* group as gjd2*1, gjd2*2 and gjd2*3, because they sometimes split into three groups, depending on the statistical analysis. Occasionally, one or two sequences split out of the gjd2*1 group, and ended in-between the other *gjd2*/*GJD2* groups. This happened particularly often with Gm-NN-gjd2*1-G01582 (sequence found in Suppl. Fig. 10), which is why we excluded this sequence during the statistical analyses. Generally, the sequences within gjd2*2 and gjd2*3 stayed as unified groups, usually as a dichotomous clade (for discussion of ohnologies within these groups, see below).

The mammalian *GJD2* is somewhat promiscuous in terms of which teleost sequence group it most closely adhered to, but most often it was gjd2*1 or gjd2*2. In zebrafish, these genes are among the few places where “a” and “b” have been added to some of the gene names in the databases. In the gjd2*2/*3 group, one of the zebrafish (and stickleback) genes is called *gjd1a* (but there is no *gjd1b*) and the other *gjd2like*. In the gjd2*1 group, one of ohnologs in zebrafish, Tetraodon, stickleback and cod is called *gjd2b* (but there is no *gjd2a*).

Another group named *gjd2like* (in Fugu and Atlantic herring) was the *cx36.7* group, called *Dr17927* in a previous paper [24]. This group often branched off from the foot of the central *gjd2* complex itself (Fig. 1), but in a few statistical analyses it located closer to *gjd3* or *gjd4* (Suppl. Table 1). As yet, there are no mammalian members in this group, and our previous work [24] suggested that this group was specific to fish.

Another orthologous group often named *gjd2like* has previously, and more uniquely, been called *cx39.2* [29]. This orthologous group divided its location between the delta (most commonly) and gamma subfamilies depending on the model run, but it never located within or at the foot of the central *gjd2* group (in contrast to *cx36.7*). The first mammalian member in the *cx39.2* group was found in opossum [29], but we here show that this ortholog is also present in several other mammals, like several Afrotheria (Suppl. Fig. 12) and bats (Fig. 6), sometimes as obvious pseudogenes. A human pseudogene (NG_026166), named “*Homo sapiens* gap junction alpha 4 pseudogene on chromosome 17” (*GJA4P*) is not a pseudogene related to *GJA4* but rather to the *cx39.2* (*GJD2-like*) group according to the phylogenetic analyses (Fig. 6). Alignments of NG_026166 against *GJA4* and representatives from the *cx39.2*-like group clearly indicated a closer relationship with the latter (Table 1; Suppl. Figs. 13A and 13B; Suppl. Tables 8 and 9). In a comparison at amino acid level between the conserved domains of human GJA4P and GJA4 vs. eel cx39.2 and cx39.4 (Table 1), the identity levels between the GJA4/cx39.4 (human/eel) orthologs were ~ 55%, the same as for GJA4P/cx39.2 (human/eel), which is clearly higher than GJA4P/GJA4 (human/human; ~ 38%) and GJA4P/cx39.4 (human/eel; ~ 34%). Also at nucleotide level, the human *GJA4P* showed higher identities to *cx39.2* than to *GJA4* from opossum and bat (Suppl. Table 9), e.g., conserved domains of Hs-*GJA4P* was 53.9% identical to opossum *GJA4*-XM_007492764 and 65.3% identical to opossum *cx39.2* (= Md-*GJD2like*-XM_001376506) (Suppl. Table 9). Thus, the alignments were consistent with the phylogenetic results (Figs. 1 and 6), and thereby settled this pseudogene (NG_026166) to be incorrectly named in humans. It is not *GJA4P*, but rather *Cx39.2P*.

**Table 1:** Conserved domains of human “GJA4P” are more similar to cx39.2 than to GJA4 at amino acid level

	Hs-GJA4P	Aj-39.2	Hs-GJA4	Aj-39.4–1	Aj-39.4–2
Hs-GJA4P–NG_026166	100.00	54.92	37.82	34.72	33.16
Aj-NN-cx39.2	54.92	100.00	48.70	40.41	44.56
Hs-GJA4-Cx37	37.82	48.70	100.00	53.89	54.40
Aj-NN-gja4-cx39.4–1	34.72	40.41	53.89	100.00	68.21
Aj-NN-gja4-cx39.4–2	33.16	44.56	54.40	68.21	100.00

Human (Hs) *GJA4P* was aligned as well as possible to the other connexin sequences at nucleotide level before being translated. The alignment is shown in Suppl. Fig. 13B. Note that the identity between eel (Aj) cx39.2 and human GJA4P is around 55%, which is at the same level as the identity between eel cx39.4 (gja4) and human GJA4. Further note that the identities between eel cx39.2 and eel gja4 are around 40%, which is the same level as the identity between human GJA4P and human GJA4. This is consistent with the results shown in the phylogenetic analyses elsewhere in this paper. Thus, human GJA4P (NG_026166) is incorrectly named, and is in reality a Cx39.2P sequence

### On teleost connexin ohnologies

The phylogenetic analyses provided a strong indication of the presence of several ohnologous pairs in teleosts. However, distinguishing between paralogous pairs that have been created by tandem gene duplication and ohnologous pairs created by genome duplication might be difficult, especially if the assembly only exists as contigs or relatively short scaffolds. If a novel teleost genome assembly is being made, it would be valuable to have the answer to this question established in other species, simply because the naming should be different in the two cases. Thus, it is of importance to show whether the ohnologous relationship can be traced across teleosts in a reasonably systematic way. In other words, is the genomic location of a gene and its potential ohnolog in one or two species sufficient to give indications for other species?

As of today, most eukaryotic draft genome assemblies consist of thousands of scaffolds, and even if these scaffolds can be Mb long, they are just a fraction of the size of most eukaryotic chromosomes. For such scaffolds, only connexin genes positioned rather closely are informative. When this analysis started, chromosomal assemblies were not available for herring and cod, but both became available during the summer of 2019 [56, 59].

For looking more closely into ohnologous pairs, the Japanese eel genome assembly was used as a starting point, because eel is a member of the early diverging fishes. Table 2 summarizes the situation for the chromosomes (or linkage groups) containing the highest number of connexin genes, and Suppl. Table 10 gives the full overview. Eel linkage group (chromosome) 19 contained eight connexin genes (*gja1*, *cx34.5*, *cx28.9*, *cx32.2*, *gja10*, *gjb7*, gjd2*1, *gje1*). The same eight genes were found on zebrafish chromosome 20 and stickleback chromosome 18, and at least seven of them are collected at cod chromosome 21. Thus, there is a strong tendency that linked genes in eel also are linked in the other species. For some unknown evolutionary reason, this chromosome had relatively few examples of ohnologs. The ohnologous chromosome may have gone through some kind of genetic catastrophe. In fact, for the two connexins with the highest number of species showing ohnology, *gja1* and gjd2*1, the ohnologs were found on an “unexpected” chromosome (7 in eel, 14 in herring and 17 in zebrafish). We use the term “unexpected” because these ohnologs deviated more from the location patterns we found for the other connexins on eel chromosome 7 (see below).

**Table 2:** Ohnologies of teleost connexins harbored at the most connexin-rich chromosomes

Ohnolog A							Connexin	Ohnolog B						
Tn	Fr	Ga	Gm	Dr	Ch	Aj		Aj	Ch	Dr	Gm	Ga	Fr	Tn
–	18	18	21	20	15	19	*gje1*	–	14	–	–	–	–	–
14	1917	18	21	20	15	19	*cx34.5*	–	–	–	–	–	–	–
14	1917	18	21	20	15/15	19	*cx32.2*	–	–	–	–	–	–	–
14	1917	18	21	20/20	15	19	*cx28.9*	–	–	–	–	–	–	–
?	1725	18	21	20	15	19	*gja1*	7	14	17	7	–	–	–
?	16	18	?	20	14	19	*gja10*	–	19	–	5	128	1843	?
14	1688	18	21	20	?	19	*gjb7*	–	–	–	–	–	–	–
10	2	18	21	20	15	19	gjd2*1	7	14	17	5	15	–	–
21	7	10	22	17	19	7	*gja9*	sc68	?	16	6	20	12	?
–	2	18	21	17	15	7	gjd2*1	19	14	20	5	15	–	10
–	–	18	21	17	19	7	*gja1*	19	14	20	7	–	1725	14
–	2	10	22	17	19	7	*cx35.4*	4	14	–	5	15	12	10
–	2	10	22	17	19	7	*cx34.4*	4	14	–	5	15	12	10
21	10	10	22	19	19	7	*gja4*	4	–	–	–	–	–	–
21	10	10	22	17	19	7	*cx28.6*	4	14	19	5	15	15	10
7	14	4	10	5	8	8	*cx39.9*	15	20	–	7/7	7	15	1
7	14	4	10	5	?	8	*gjb1*	15	20	14	7	7	15	1
2	16	–	–	9	2	8	*gja8*	14	21	1	20	1	–	–
7	16	6	?	9	2	8	*gja5*	14	21	1	–	16	16	17
2/2/2	1/1	1	4	9	2	8	*cx30.3*	14	8/21	–	20	115	8	3
2	1	1	4	9	2	8	*gja3*	14	21	–	20	115	8	3

The following species abbreviations are used (second row): Aj, eel; Ch, Atlantic herring; Dr., zebrafish; Gm, Atlantic cod; Ga, stickleback; Fr, Fugu; Tn, Tetraodon. Using eel as a starting point, the chromosomes/linkage groups/scaffolds with the highest number of connexin genes were identified. The chromosomal location of the corresponding orthologs was identified in the other species (left part of the table). Subsequently, the chromosomal location of the ohnologous genes was identified in the same species (right part of the table). The order of the genes is given by their location on the eel chromosomes. Among the sequences mentioned in this table, there are five obvious examples of tandem gene duplications, indicated by several identical numbers with slashes in-between (e.g., 2/2/2 for *cx30.3* ohnolog A in Tn) and one example of a presumed gene duplication located to different chromosomes (*cx30.3* ohnolog B in Ch).?, the sequence is unplaced; sc68 and three or four digit numbers indicate scaffold number

Eel chromosome 7 contained five connexin genes, in addition to the ohnologs of *gja1* and gjd2*1, namely *gja4*, *gja9*, *cx28.6*, *cx35.4* and *cx34.4*, and four of their ohnologs were placed at chromosome 4 (and chromosome 19 for *gja1* and gjd2*1). In stickleback, all five genes were found on chromosome 10 (but chromosome 18 for *gja1* and gjd2*1), and three of the ohnologs were found on chromosome 15, the fourth ohnolog (*gja9*) on chromosome 20, and the fifth was missing. In Tetraodon, three of the five genes (*gja4*, *gja9*, *cx28.6*) were found on chromosome 21, and two of these had ohnologs, *gja9* on an unplaced scaffold, and *cx28.6* on chromosome 10. Tetraodon chromosome 10 also contained the single copies of *cx35.4* and *cx34.4*. In zebrafish, *gja9*, *cx28.6*, *cx35.4*, and *cx34.4* were found on chromosome 17. *Gja4* was present as a single paralog on chromosome 19, which also contained the ohnolog of *cx28.6*. Thus, we see for *gja4*, *gja9*, *cx28.6*, *cx35.4* and *cx34.4* on eel chromosome 7 that there was a strong tendency towards a pattern of consistency in distribution of ohnologous pairs to distinct chromosomes in all the investigated species, while *gja1* and gjd2*1 tended to deviate.

In general, teleosts had four genes that were very closely related to mammalian *GJD2*. Although one or two of the sequences in the gjd2*1 group occasionally split out from the remaining genes, the two ohnologs (Table 2) generally stayed together, and there should be no doubt about the proper ohnology. In 14 of 21 statistical analyses gjd2*1 grouped together with mammalian *GJD2*, and these were considered as the appropriate orthologs. Gjd2*2 and gjd2*3 often dichotomously grouped together (in 11 of 21 statistical analyses), but other times split up. We believe that gjd2*2 and gjd2*3 most likely are ohnologs, although it could not totally excluded that they are non-ohnologous paralogs located on different chromosomes.

If the genes that were linked in eel had broken linkages in other species, in many cases two or three of the most closely linked genes have moved to another chromosome than the rest of the group. A more complete overview containing all connexin genes and associated chromosomes is provided in Suppl. Table 10.

Of course, this analysis also showed closely related genes that were not ohnologs. E.g., the genes within the *cx34.5* and *cx32.2* groups (also known as *cx32.7*, *cx32.3* and *cx28.9*) are not ohnologs, because they all are located on the same chromosome (19 in eel, 15 in herring, 20 in zebrafish, 21 in cod, 18 in stickleback, 14 in spotted pufferfish, and scaffold1917 in Fugu).

In summary, over the range of divergence time, large stretches of the chromosomes have been maintained reasonably intact subsequent to the teleost genome duplication. Thus, the corresponding ohnologs are found on other non-random chromosomes. However, both gene losses and tandem duplications might have occurred over the considered evolutionary period, which could complicate the interpretations. Of course, this is even further complicated by the facts that the sequencing itself is probably not able to reach a complete coverage of the genome causing the partial or full absence of a gene, and that the assembly process is not straight-forward.

### Lack of expected connexin genes may point to chromosomal misassemblies

As an example of practical use of this kind of information, we here briefly apply the knowledge of the outlined patterns of the connexin genes on (i) the first published herring genome assembly [17, 62], which was used as basis for GenBank gene predictions from 2015 (XM accession numbers in GenBank); (ii) the new herring chromosome level assembly [59, 60]; and (iii) a herring genome assembly made by the present authors [40, 58]. Although the GenBank herring gene predictions were superior when compared with most other fishes (in the sense that the predictions tended to follow the expected gene patterns), there were still some features worth noting.

**Fig. 7:**
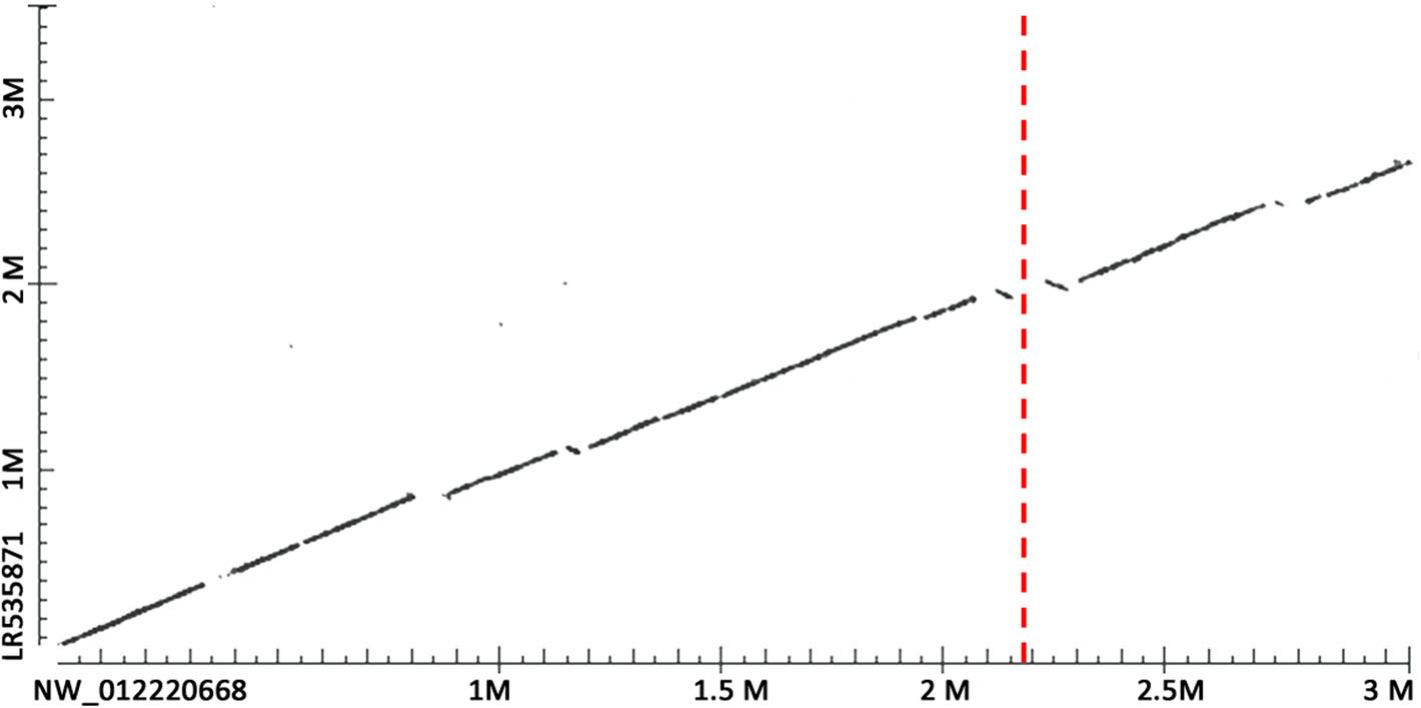
Problem in herring assembly of chromosome 15 at assumed position of *gjb7*. Scaffold NW_012220668 from the draft herring genome assembly contains *gjb7* in position 2,189,757–2,188,978 (i.e., on the reverse strand). This scaffold was aligned with herring chromosome 15 assembly LR535871 position 0 to 3,500,000 using the alignment option in Blast. The position of *gjb7* on NW_012220668 is indicated by the red dotted line. There are apparent inversions and breaks in the area where *gjb7* was expected in chromosome 15. The word size in the alignment was 256

- First, there was one easily found connexin (*cx39.2*) that was not predicted in the annotation from the 2015 herring genome assembly [17].
- Second, several connexin genes showed identical or near identical duplicates in the first herring genome assembly. The *gjb3like*-XM_ XM012822385 (one of the ohnologs in the *cx35.4* group) was identical to XM_012822374 and XM_012822365, found at three locations on scaffold NW_012217989. The *gjb3like*-XM_012818491 (the second ohnolog in the *cx35.4* group) was identical to XM_012818489; found at two locations on scaffold NM_012210726. The *gjb4like*-XM_012818492 (one of the ohnologs in the *cx34.4* group) was nearly identical to XM_012819490, and both were found on scaffold NW_012219726. The *gjd3like*-XM_012837668 was nearly identical to XM_012837669, and both were found on scaffold NW_012223269. Although such copies are not entirely biologically implausible, they are not probable, and are more likely caused by assembly errors. Indeed, in the initial states of our own assembly most of them were not present in duplicate sequences, only becoming so in the last step where our assembly was fused with the published herring genome [40]. In the recently released (summer 2019) herring chromosome level assembly [59, 60] most of these duplicates have collapsed into a single copy of the sequence.
- Third, three connexin genes have “disappeared” from the new herring chromosome level assembly. These are *gja9like*-XM_012824682, *gjb1like*-XM_012819602 and *gjb7like*-XM_012823856. The corresponding orthologs are found in the other teleost species, and - even more importantly - hits were found in the two other herring genome assemblies. We have verified the presence of these genes in our early assemblies [40]. This strongly indicates misassemblies in the new chromosome level assembly. More specifically, the lack of *gjb7* indicates a misassembly on chromosome 14 or 15. Indeed, an alignment of the relevant scaffold and chromosome showed breaks and inversion around the expected position of *gjb7* at chromosome 15 (Fig. 7). The apparent lack of the *gjb1* ohnolog indicates a misassembly on chromosome 8, where we indeed found breaks and inversions (not shown). We expected that the lack of the *gja9* ohnolog to indicate a misassembly on chromosome 14, but we found the relevant scaffold to align with chromosome 11, where again breaks and inversions were found (not shown).

Regarding the third point above, also the recent chromosome level assembly in cod [56] showed a “no hit” for the Gm-*gja10* ohnolog Gm-*cx52.6*-G05425 and for Gm-*gja5-*G04028 (Suppl. Table 5 and 10). The lack of *gja5* suggested a problem in the assembly of cod chromosome 20 around position 1,000,000 (Suppl. Fig. 16A). Gm-*cx52.6* is located on a small and unplaced contig (not even containing the full-length sequence of the gene), which was unusable for dot plot alignment at a chromosomal scale. By using suitable scaffolds containing the *cx52.6* ortholog from herring and stickleback, we believe there is a problem in assembly of cod chromosome 21 around position 2,700,000 (Suppl. Fig. 16B and C). Other alignments using the corresponding zebrafish sequence also pointed to the same location.

### A more consistent nomenclature suggestion

We believe that improved gene predictions and annotations are possible through the proper incorporation of knowledge into the algorithms. Furthermore, it would certainly help if the genes were labeled with unique names, as is one of the underlying logics in the instructions from the Human Gene Nomenclature Committee and the Zebrafish Gene Nomenclature Conventions. For most of the genes in the teleost connexin family, it is easy to suggest names that follow the nomenclature guidelines. Suppl. Fig. 17 presents a suggestion that follows the recommendations from the Human and Mouse Gene Nomenclature Committees and the Zebrafish Nomenclature Conventions, and Table 3 shows a translation between the nomenclature systems, including the recently suggested “alphabetic” system in mammals [26]. In our suggestion, we maintain the Greek nomenclature naming and numbering of those genes that are well established names in human and mouse, and transfer the naming conventions to the corresponding orthologs in teleosts. We fully avoided the “-like” names, as they often are used for several distinct genes and thus do not indicate a concrete orthologous group, and in this way can be misleading.

**Table 3:** Translation between different connexin nomenclature systems in mammals and teleosts. Some pseudogenes are not included in the overview

Mammals			Teleosts		
Greek	Size	Alphabet	**Amended Greek**	Commonly used Greek	Commonly used size
*GJA1*	*Cx43*	*CXNK1*^*2*^	***gja1a***	*gja1, gja1like*	*cx43, cx40.8*
			***gja1b***		
*–*	*–*	*–*	***gja2a***	*gja3like*	*cx39.9*
			***gja2b***		
*GJA3*	*Cx46*	*CXNJ1*	***gja3a***	*gja3, gja3like, gja3a, gja3b*	*cx48.5*
			***gja3b***		
*GJA4*	*Cx37*	*CXNH1*	***gja4a***	*gja4*	*cx39.4*
			***gja4b***		
*GJA5*	*Cx40*	*CXNI*	***gja5a***	*gja5, gja5like, gja5a, gja5b*	*cx41.8*
			***gja5b***		
*GJA6, GJA6P*	*Cx33, Cx43pX*	*Cxnk2*^*2*^	**–**	*–*	*–*
*GJA8*	*Cx50*	*CXNL*	***gja8a***	*gja8, gja8a, gja8b*	*cx44.1, cx44.2*
			***gja8b***		
*GJA9*	*Cx58*	*CXNM*	***gja9a***	*gja9, gja9like*	*cx52.9, cx55.5*
			***gja9b***		
*GJA10*	*Cx62, Cx59*	*CXNN*	***gja10a***	*gja10, gja10like*	*cx52.6, cx62, cx52.7*
			***gja10b***		
*–*	*–*	*–*	***gja11***		*cx32.7, cx34.5*
*–*	*–*	*–*	***gja12***		*cx28.1, cx28.9, cx32.2like*
*–*	*–*	*–*	***gja13***		*cx32.2, cx32.3, cx32.2like*
*GJB1*	*Cx32*	*CXNG*	***gjb1a***	*gjb1, gjb1like*	*cx27.5, cx31.7*
			***gjb1b***		
*GJB2*	*Cx26*	*CXNE*	***–***	*–*	*–*
*GJB3*	*Cx31*	*CXNC*	***gjb3a***	*gjb3like*	*cx35.4*
			***gjb3b***		
*GJB4*	*Cx30.3*	*CXNB*	***–***	*–*	*–*
*GJB5*	*Cx31.1*	*CXNA*	***–***	*–*	*–*
*GJB6*	*Cx30*	*CXNF*	***–***	*–*	*–*
*GJB7*	*Cx25*	*CXND*	***gjb7***	*gjb7*	*cx25, cx28.8*
*–*	*–*	*–*	***gjb8a***	*gjb2like, gjb6like*	*cx30.3, cx33.8*
			***gjb8b***		
*–*	*–*	*–*	***gjb9a***	*gjb4like*	*cx28.6, cx30.9*
			***gjb9b***		
*–*	*–*	*–*	***gjb10a***	*gjb4like*	*cx34.4*
			***gjb10b***		
*GJC1*	*Cx45*	*CXNQ*	***gjc1a***	*gjc1, gjc1like, gjc1a, gjc1b*	*cx45*
			***gjc1b***		
*GJC2*	*Cx47*	*CXNO*	***gjc2***	*gjc2*	*cx47.1*
*GJC3*	*Cx31.3*	*CXNP1*	***–***	*–*	*–*
*–*	*–*	*–*	***gjc4a***	*gjc1like*	*cx43.4, cx44.2*
			***gjc4b***		
*–*	*–*	*–*	***gjd1a***	*gjd1a, gjd2like*	
			***gjd1b***		
*GJD2*	*Cx36*	*CXNS*	***gjd2a***	*gjd2, gjd2b, gjd2like*	*cx35, cx35.1*
			***gjd2b***		
*GJD3*	*Cx31.9*	*CXNR*	***gjd3***	*gjd3, gjd3like*	
*GJD4*	*Cx40.1, Cx39*	*CXNU*	***gjd4a***	*gjd4, gjd4like*	*cx40.1*
			***gjd4b***		
^*1*^	*Cx39.2*	*–*	***gjd5a***	*gjd2like*	
			***gjd5b***		
*–*	*–*	*–*	***gjd6***	*gjd2like*	*cx36.7*
*GJE1*	*Cx23*	*CXNT*	***gje1***	*gje1, gje1like*	*cx23*

^1^There is no official Greek designation for this group, and the predicted genes in mammals have received different names, such as *GJA4like, GJD2like* or *GJC2like* (Suppl. Fig. 12)*.* Following our suggestion for the teleosts, the group should be called *GJD5* in mammals

^2^The human and mouse genes in the *CXNK* group have not been named consistently in the alphabetic nomenclature. According to our view, the naming in ref. [26] reflects the following orthologous relationships: *GJA1* = *Cx43* proper = human *CXNK2* = mouse *cxnk1*; human *GJA1P* = human *Cx43P1* = human *CXNK1* = no mouse ortholog; human *Cx43pX* = human *GJA6P* = no alphabet name in humans = mouse *GJA6* = mouse *cxnk2*

The mammalian Greek nomenclature (first column) is decided by the Human and Mouse Nomenclature Committees. The size nomenclature system in mammals (second column) mentions sizes used in humans and mouse, with exception of the third last row, which shows the size in opossum. The alphabet nomenclature system (third column) was recently suggested by Premzl for eutherian connexins (ref. [26]). Our suggested amended Greek nomenclature for teleosts is shown in bold in the fourth column. The groups with teleost ohnologs are indicated with a/b. The phylogenetic tree with the nomenclatures is shown in Suppl. Fig. 17

The subfamily number (*gjd1*/*2*/*3*/*4*, etc.) for the groups where new names are suggested does not consider the chronological order of detection, but rather the numbers that are available. For example, *cx39.9* is closely related to *gja3*, and is in fact often called *gja3like*. As *gja1*, *gja3*, *gja4, gja5,* and *gja6* already are occupied, while *gja2* is not, we suggest calling *cx39.9*/*gja3like* for *gja2*. The genes in the *cx34.5*, *cx28.9* and *cx32.2* groups are called *gja11*, *gja12* and *gja13*, respectively. We skip *gja7*, as this name has historically been used for *Cx45* (= *GJC1*).

Statistically, a strong link exists between *cx35.4*/*gjb3like* and *GJB3*. We therefore suggest that *cx35.4* should be called *gjb3*, despite the lack of the hallmark of the mammalian GJB3 protein, namely the amino acid sequence CX_5_CX_5_C in the second extracellular loop, where all other connexins (except the GJE1 proteins) have the sequence CX_4_CX_5_C.

There is a particular problem in the beta subfamily in that the mammalian *GJB2* and *GJB6* are always located in a dichotomous manner in the phylogenetic analyses, and similarly for *GJB4* and *GJB5*. There were no indications that *cx30.3* located closer to either of *GJB2* or *GJB6*, and similarly, *cx34.4* did not locate closer to either of *GJB4* or *GJB5*. It might be that *cx30.3* is a precursor gene for both *GJB2* and *GJB6*, and *cx34.4* is a precursor gene for *GJB4* and *GJB5*, as we have suggested earlier [23, 24]. Thus, several possibilities exist for naming these genes. *Cx30.3* could be called *gjb8* (following the present pattern in the Greek nomenclature), *pre-gjb2/6* (indicating the potential of being a precursor for the two mammalian genes), or *gjb26* (a variant of the previous, but with the potential danger that this could be mistaken for *cx26*). We suggest *cx30.3* is called *gjb8* and *cx34.4* is called *gjb10*. We further suggest that *cx28.6*, which generally located at the root of ((*GJB4*-*GJB5*)-*gjb10*)-(*GJB3*-*gjb3*) (parentheses indicate branching structure), is called *gjb9*.

In the gamma subfamily, there are two groups concerned with renaming. The first one is in marsupials, where the majority of statistical analyses (Suppl. Table 1) support *GJC1like*/*GJC2like* genes probably being the orthologs of eutherian *GJC3*, as originally suggested [29]. The second group is *cx43.4*/*44.2*/*gjc1like*, which we suggest is renamed *gjc4*.

In the delta subfamily, the major problems concern the *gjd2* complex. As briefly discussed above, we consider gjd2*2 and gjd2*3 probable ohnologs, and suggest that they are named *gjd1*, fitting with a zebrafish and a stickleback sequence within this group already named *gjd1*. The ohnolog pairs within gjd2*1 are probably orthologs with mammalian GJD2, and consequently we suggest they are named *gjd2*. The teleost *cx36.7*/*gjd2lik*e group never dichotomized with any of the mammalian genes and most often branched off from the root of the *gjd2* complex. We suggest this group should be called *gjd6*. The last group is the little-studied *cx39.2* group, which in mammals has a variety of names in database gene predictions, such as *GJC2like*, *GJD2like* and *GJA4like*. The mammalian genes robustly dichotomize with the corresponding teleost genes, which in the databases usually are called *gjd2like*. We suggest that this clade is called *GJD5* in mammals (thus, the human pseudogene NG_026166 should be called *GJD5P*) and *gjd5* in teleosts.

## Discussion

The first phylogenetic analyses of the (nearly) complete connexin gene family across the vertebrates, from mammals to teleosts, were performed in the early genomic era [22–24, 29]. The analyses indicated that there was a considerable degree of conservation in this gene family, and that the subfamily structure could be recognized across the different vertebrate groups. Using a wide basis of teleosts, from the early diverging eels to the late diverging pufferfishes, we here confirm that the substructure of the gene family in teleosts is stable and easily recognizable. On the other hand, there is a high degree of inconsistency in the naming of the teleost genes in the connexin family in two major genetic databases, GenBank and Ensembl, both internally within each database and between the databases. The naming does not generally follow the recommended guidelines from the Human Gene Nomenclature Committee [4] and the Zebrafish Nomenclature Conventions [5]. Even the annotations of the novel chromosome level genome assemblies in herring [59, 60] and cod [56], made available during 2019, possess such inconsistencies. A part of the differences probably reflects different annotation pipelines and the use of different databases as knowledge basis in Ensembl and GenBank.

We are confident that our overall results are robust, due to a high degree of consistency across several applied methods and parameters. We therefore suggest an amended and extended Greek nomenclature that follows the guidelines of the Human Gene Nomenclature Committee [4] and the Zebrafish Nomenclature Conventions [5]. The suggestion also includes the naming for ohnologs, i.e., duplicated genes generated by genome duplication.

We noted previously that connexin genes could indicate errors in a genome assembly [40], like duplicated areas or areas not sequenced. Having confirmed the stability of the substructure in the gene family in teleosts, we can be even more certain that different anomalies that may come up when analyzing the complete gene family in a teleost species are pointing out potential assembly errors. These types of analyses share some common grounds with Core Eukaryotic Gene Mapping Approach (CEGMA) [63] and Benchmarking Universal Single Copy Orthologs (BUSCO) [64, 65], in that selected genes are investigated for their presence in a genome to verify the completeness of a newly assembled genome. They differ in that the connexins are a family of genes, consisting of several subfamilies, as well as that these genes have two conserved domains that have some reciprocal similarities.

In the context of genome assembly and gene annotation, the connexins are a randomly selected gene family. It is curious how, even in very recent high-quality genome assemblies, such as those of Atlantic herring and cod, these genes can indicate certain potential misassemblies. This situation can possibly be extended to other gene families and single genes, as the number of missing BUSCOs in the herring genome in the herring genome increased from 2.9% (131/4584) in the draft herring genome assembly [17] to 8.1% (374/4584) in the chromosome level assembly [60] according to our analysis [40].

It is possible to analyze an un-annotated genome for a specific genes or gene families when it is known exactly what to look for, but the value of a genome assembly increases considerably if it is well annotated. Poor annotations may mislead, and certainly may make different kinds of comparisons more difficult. For example, a synteny analysis could potentially give wrong conclusions if the gene names do not indicate the proper genetic relationships. Our results suggest that it should be fully possible to improve the annotation of the connexin gene family. We would be surprised if the inconsistencies pointed out here only concern the connexin gene family.

## Conclusions

The practice of naming connexin genes in teleosts exhibits many inconsistencies. Commonly, distinct genes are assigned the same name, and there are examples of clearly incorrect names, even in mammals, including that of a human pseudogene (NG_026166). By using many different phylogenetic models, we could classify the teleost sequences that had a dichotomous relationship with the corresponding mammalian sequences, and thereby point out the sequences that should have the same name as their mammalian orthologous counterpart. Conversely, if there was no mammalian counterpart they should have a unique name. It was further settled which of the teleost sequences that existed in ohnologous pairs, and thereby should have their names followed by “a” or “b”. To quite some extent, it is possible to predict at which chromosome a teleost connexin should be located. We investigated two very recent high-quality chromosome assemblies (herring and cod), finding that roughly 5% of the expected connexin sequences were absent (two in cod and three in herring). We found likely misassemblies or gaps at the expected positions for the missing connexins in the chromosome assemblies.

## Methods

### Collection of sequences

The major procedures are schematically outlined in Suppl. Fig. 18. The collecting of sequences was done by several approaches. First, previously collected sequences [23, 24, 29] were checked against the present and updated versions of the genomes to include potential revisions of the gene sequences. This was done by searching GenBank (nucleotide collection, whole genome contigs, transcriptome shotgun assemblies, and specific genome assemblies), using nucleotide BLAST [66, 67], and Ensembl, using the corresponding BLAST/BLAT option built into Ensembl, in both cases obtaining pairwise alignments between our old sequences and the present sequences in the databases. Second, annotated and named sequences were found by using “connexin”, “gap junction protein”, “gja”, “gjb”, “gjc”, “gjd”, “gje” and the relevant species names as search terms in GenBank and Ensembl. If there was a lack of certain expected sequences in a certain species, the genome assemblies for the species in question were searched using the corresponding (assumed) orthologs from other species. When needed, multiple alignments by MUSCLE [68] were done, e.g., to settle the probable borders between introns and exons and to determine the percentages of identities between different sequences (e.g., in Suppl. Figs. 13A and 13B). By the combination of approaches described above, we found several connexin sequences presently not predicted in the databases, and they were included in our analyses (marked by NP as described below under *Naming terminology*).

If the experimentally confirmed or predicted sequences were available in GenBank, their accession numbers were also collected (to ensure the unique naming of the sequences). Depending on species and gene in question, we have used the NCBI Reference Sequences whenever possible. Otherwise, gene/RNA names or numbers were collected from Ensembl. All sequences, with GenBank accession numbers or Ensembl gene numbers if relevant, are provided in Supplement Figs. 1–12.

Among teleosts, we have collected sequences from zebrafish (*Danio rerio*, abbreviated Dr), stickleback (*Gasterosteus aculeatus,* Ga) [69], Japanese pufferfish (*Takifugu rubripes,* often called *Fugu rubripes*, called Fugu in the text, and abbreviated Fr) [50, 70], green spotted pufferfish (*Tetraodon nigroviridis,* Tn) [71], Atlantic herring (*Clupea harengus,* Ch) [17, 62], Atlantic cod (*Gadus morhua*, Gm) [48, 72] and European, American or Japanese eel (*Anguilla anguilla*, Aa; *Anguilla rostrata*, Ar; or *Anguilla japonica*, Aj). For eel, we have chosen to refer to an improved *Anguilla japonica* assembly [73, 74] because it has by far the longest scaffolds, aided by other genome shotgun assemblies of *A. japonica* [75], *A. anguilla* [44] and *A. rostrata* [46], as well as transcriptome shotgun assemblies from *A. anguilla* [76–78] and *A. japonica* [79].

As a comparison for the fish sequences, and to follow the Zebrafish Nomenclature Conventions [52], we collected sequences from humans (*Homo sapiens,* Hs; Suppl. Fig. 1), mouse (*Mus musculus,* Mm; Suppl. Fig. 2), and opossum (*Monodelphis domestica,* Md; Suppl. Fig. 3), and supplemented them with certain single sequences from platypus (*Ornithorhynchus anatinus*, Oa), koala (*Phascolarctos cinereus*), Tasmanian devil (*Sarcophilus harrisii*, Sh), wallaby (*Notamacropus eugenii*), large flying fox (*Pteropus vampyrus,* Pv), black flying fox (*Pteropus alecto*, Pa), Egyptian rousette (*Rousettus aegyptiacus*, Ra), aardvark (*Orycteropus afer afer,* Afer), manatee (*Trichechus manatus*, Tm), African elephant (*Loxodonta africana*, La) and armadillo (*Dasypus novemcinctus*, Dn) (Suppl. Figs. 4 and 12). All sequences are given in the Supplemental Information, where also the relevant database can be inferred according to the name/identity we have given the sequence.

Suggested deviations from the predicted sequences are indicated in the Supplemental Information. If the predicted sequences did not contain potential start and stop codons, we analyzed the genomes to extend the sequences to those codons, following the pattern established by connexins orthologs in other species. If the predicted sequences contained introns, we investigated whether moving the exon-intron borders improved the similarity between sequences and the established sequence patterns, even by including the whole intron as a part of the exon. In a few cases, we also suggested other types of modifications, following the patterns established for these sequences in other species. Furthermore, any unpredicted sequences (i.e., those not predicted in Ensembl or GenBank) we found during the present searches, were included.

Several pseudogenes exist in the gap junction gene family, also in humans [29]. With a single exception, obvious pseudogenes are not included in the analyses shown. The one exception is a novel human pseudogene (GenBank NG_026166; claimed as *GJA4* pseudogene) that we did not detect in our previous analyses [23, 24, 29]. Additionally, orthologs to NG_026166 were extracted from the genomes of several mammalian species (Suppl. Fig. 12).

### Naming terminology

To distinguish between human genes and other species, it is generally recommended that abbreviations for human gene names are spelled in upper case letters, while using lower case letters for other species. For the purpose of the present paper, this would be inconvenient as we often are referring to the gene groups, and we are here using upper case when referring to human genes and mammalian orthologous gene groups, while teleost genes in general are indicated by lower case letters. We also use upper case letters when we are referring to a whole orthologous group (i.e., mammalian plus teleost genes). There are some exceptions to the upper/lower case spelling, because when we refer to specific single genes, we use (as far as possible) the gene names given in GenBank or Ensembl.

To ensure uniqueness of every name used in the present work, we added the GenBank accession number or an abbreviated form of the Ensembl gene number to the names for which predictions were available in the present databases. Specific gene names were generally abbreviated as indicated by the database, or the abbreviations can be inferred from the database name. E.g., for XM_003965660, the full name (“definition”) is “*Takifugu rubripes* gap junction protein, alpha 9, 59 kDa (gja9), mRNA”. In this case, the name is given with both the Greek and size nomenclature, and the name is abbreviated in lower case in parentheses. Thus, we have here used the gene name Fr-*gja9-cx59*-XM_003965660*.* For XM_021466745, the full name is “*Danio rerio* connexin 55.5 (cx55.5), transcript variant X1, mRNA”. We here abbreviated the name to Dr-*cx55.5*-XM_021466745. For XM_011619942, the full name is “*Takifugu rubripes* gap junction alpha-10 protein-like (LOC1010664818), mRNA”, and it was abbreviated Fr-*gja10like*-XM_011619942. Where several transcript variants are experimentally shown or predicted, we only used transcript variant X1.

If the gene was predicted in the Ensembl database, but no name was available, we used a relevant gene name to indicate the correct group of sequences. For example, the Tetraodon *gjb2/6*-like sequence ENSTNIG00000010340 (with the corresponding transcript prediction ENSTNIT00000013438) had no name or description. We abbreviated the gene Tn-NN*-cx30.3-*G10340 (where NN = No Name). This is an example of a gene for which our transcript prediction differed from the database, as indicated in the Supplemental Information.

If the gene was not predicted in a species, but found in our Blast searches, it was suitably named but with the prefix NP (Not Predicted). One example is Tn-NP*-cx30.3*. Thus, Tetraodon has a total of four genes in the *Cx30.3* group, two that have been predicted and are named in Ensembl, one that has been predicted but not named, and one that has not been predicted by the database (but by us).

To be able to follow certain very closely related groups of sequences in an easy manner, previously un-named (or unpredicted) sequences in the *cx30.3* and *gjd2* groups were named with the postfixes *1/*2/*3 for the purposes of the present manuscript.

### Phylogenetic analyses

The phylogenetic analyses were performed in MEGA7 [80] or MEGA-X [81] using the conserved domains essentially as described in Cruciani and Mikalsen [24] because of the distant evolutionary relationship between mammals and fish. Here, we extended the previously defined conserved domains by 15 nucleotides in 3′-direction for the first conserved domain (i.e., into the sequence corresponding the intracellular loop), and by 15 nucleotides in both 5′- and 3′-direction for the second conserved domain. All sequences and the limits of the sequences used in the phylogenetic analyses are presented in the Suppl. Fig. 1–12, where previously defined conserved sequences [24] are marked in yellow, and the 15 nucleotide extensions are marked in grey.

The main questions for the phylogenetic analyses were related and also partly overlapping, and were as follows: (i) The connection between the naming of the teleost sequences (naming taken from the main databases GenBank and Ensembl) and their position in a specific orthologous group, i.e., do teleost orthologs have the same name? (ii) The (orthologous) relationships between the teleost sequences and the corresponding mammalian sequences. Is there a (reasonably) stable structure in the connexin gene family across the teleosts, i.e., do teleost connexins distribute into orthologous groups in a manner more or less similar to the mammalian sequences? (iii) The ohnologies among the teleost sequences. Note that our present questions do not concern the relatedness within the whole tree (i.e., the complete evolutionary history of the connexin gene family). The present knowledge of evolutionary history of the connexin gene family is graphically summarized in Fig. 4 in ref. [24]. The needed translation between the nomenclature systems is found in Table 3. This translation also includes the recently suggested “alphabetical” nomenclature in mammals [26].

Model selection was run in MEGA X using amino acid models. Settings were automatic tree building using Neighbor-Joining model and partial deletion using a site coverage cutoff of 95%. Minimal differences were found between the models estimated with similar Bayesian Information Criterion, but in general simpler models were preferred (Jones-Taylor-Thornton, Le-Gascuel and Dayhoff substitution matrices). We therefore ran the phylogenetic analyses with different construction methods (Maximum Likelihood, Maximum Parsimony and the two distance methods Neighbor-Joining and Minimum Evolution) using different substitution models as indicated in Suppl. Table 2. Several construction methods were used as they have different strengths and weaknesses with regard to the degree of relatedness of the sequences, the differences in evolutionary rates in different branches, how highly divergent sequences are behaving, etc. Settings for each particular analysis are available in Suppl. Table 2. Each method was used at both amino acid and nucleotide levels (the latter using only positions 1 and 2 in the codon), and in many cases with both bootstrap and interior branch statistics. In total, 21 statistical analyses were performed, and they are summarized in Suppl. Table 1, with the corresponding parameter settings in Suppl. Table 2. All these methods are included in the MEGA phylogenetic software. If all, or most, of the statistical comparisons supported a specific dichotomous relationship, we deemed the results more robust.

## Supplementary information

**Additional file 1. Suppl. Figure 1.** Human (*Homo sapiens*) connexins. **Suppl. Figure 2.** Mouse (*Mus musculus*) connexins. **Suppl. Figure 3.** Opossum (*Monodelphis domestica*) connexins. **Suppl. Figure 4.** *GJC1like* and *GJA9* connexin sequences from other marsupials and platypus. **Suppl. Figure 5.** Zebrafish (*Danio rerio*) connexins. **Suppl. Figure 6.** Japanese pufferfish (Fugu; *Takifugu rubripes*) connexins. **Suppl. Figure 7.** Green spotted pufferfish (*Tetraodon nigroviridis*) connexins. **Suppl. Figure 8.** Three-spined stickleback (*Gasterosteus aculeatus*) connexins. **Suppl. Figure 9.** Atlantic herring (*Clupea harengus*) connexins. **Suppl. Figure 10.** Atlantic cod (*Gadus morhua*) connexins. **Suppl. Figure 11.** Japanese eel (*Anguilla japonica*) connexins. **Suppl. Figure 12.** *Connexin39.2* (“*gjd2like*”) from mammals. **Suppl. Figure 13.** Comparisons of human “*GJA4P*” against *connexin39.2* and *GJA4*. **A.** Alignment of conserved domains in human “*GJA4P*” (NG_026166) against *connexin39.2* (“*gjd2like*”) in various species at protein level. **B.** Alignment of conserved domains in human “GJA4P” (NG_026166) against GJA4 (connexin37) from human and eel at protein level. **Suppl. Figure 14.** Expanded branches from the phylogenetic tree shown in Fig. 1. **A.** Expanded view of the mammalian and teleost *GJA1* branch. **B.** Expanded view of mammalian and teleost *GJA3* branch, and the associated teleost *cx39.9*. **C.** Expanded view of the mammalian and teleost *GJA4* branch. **D.** Expanded view of the mammalian and teleost *GJA5* branch. **E.** Expanded view of the mammalian and teleost *GJA9* and *GJA10* branches. **F.** Expanded view of the teleost *cx34.5* and *cx32.2* branches. **G.** Expanded view of the mammalian and teleost *GJB1* branch. **H.** Expanded view of mammalian and teleost *GJB2* and *GJB6* branch, and teleost *cx30.3* branches. **I.** Expanded view of the mammalian *GJB3* and teleost *cx35.4* branches. **J.** Expanded view of mammalian *GJB4* and *GJB5*, and teleost *cx34.4*. **K.** Expanded view of the mammalian and teleost *GJB7* branch. **L.** Expanded view of the teleost *cx28.6* group, and its relationship with *GJB3*/*GJB4*/*GJB5*. **M.** Expanded view of eutherian *GJC3* and marsupial *GJC1like* and *GJC2like* branches. **N.** Expanded view of mammalian and teleost *GJC1* and teleost *cx43.4* branches. **O.** Expanded view of mammalian and teleost *GJC2*, and its relationship with *GJC1* and *cx43.4*. **P.** Expanded view of mammalian and teleost *Cx39.2* branch. **Q.** Expanded view over the central *GJD2* complex. **R.** Expanded view of mammalian and teleost *GJD3* branch. **S.** Expanded view of mammalian and teleost *GJD4* branch. **T.** Expanded view of teleost *cx36.7* branch. **Suppl. Figure 15.** Compressed phylogenetic tree illustrating long-branch attraction between *gjc3*, *gjd4* and *gje1* groups. **Suppl. Figure 16.** Searching for positions of connexins lacking in chromosome assemblies. **A.** Problem in cod assembly of chromosome 20 at assumed position of *gja5*. **B.** Alignments with sequences from herring and stickleback point to the same area on cod chromosome 21, indicated expected position of *gja10-cx52.6*. **C.** Alignments of herring and stickleback scaffolds containing *cx52.6*. **Suppl. Figure 17.** A homogeneous and consistent nomenclature for gap junction protein genes. **Suppl. Figure 18.** Schematic outline of the major procedures.

**Additional file 2. Suppl. Table 1.** Statistical support for clade grouping. **Suppl. Table 2.** Parameter overview for statistical analyses of phylogenetic trees. **Suppl. Table 3.** Comparison between zebrafish connexin sequences from Ensembl and GenBank. **Suppl. Table 4.** Comparison between Fugu connexin sequences from Ensembl and GenBank. **Suppl. Table 5.** Comparison between cod connexin sequences from the Ensembl and GenBank assemblies. **Suppl. Table 6.** Comparison between herring connexin sequences from the GenBank genome assembly predictions and the Ensembl chromosomal level assembly predictions. **Suppl. Table 7.** Naming of connexin genes in Ensembl and GenBank. **Suppl. Table 8.** Percentages of amino acid identities between conserved domains in mammalian Cx39.2, including human “GJA4P”-NG_026166, and eel cx39.2 (one of the “gjd2like” sequences). **Suppl. Table 9.** Human *GJA4P* is more similar to *GJD2like* (*connexin39.2*) than GJA4 at nucleotide level. **Suppl. Table 10.** Ohnology among teleost connexins.

## Data Availability

The datasets supporting the conclusions of this article are included within the article and its additional files. All sequences, with all required information, are in the Additional File 1. Please note that the data have been handled manually, and human error and inconsistencies may have occurred. If significant errors are detected, we would be grateful to receive a notification.

## Abbreviations

BUSCO: Benchmarking Universal Single Copy Orthologs
CEGMA: Core Eukaryotic Gene Mapping Approach
Cx: Connexin
gja: Gap junction protein alpha gene
gjb: Gap junction protein beta gene
gjc: Gap junction protein gamma gene
gjd: Gap junction protein delta gene
gje: Gap junction protein epsilon gene
NN: No Name
NP: Not Predicted

## Footnotes

1 Please note that the naming of each single sequence in most of the figures and tables follows as closely as possible the naming and orthography (including lower and upper case letters) in the respective databases from where the sequences were collected (see also detailed explanation in the Methods section and in legend to Fig. 2).
