# Supplementary material for: Phylogeny of teleost connexins reveals highly inconsistent intra- and interspecies use of nomenclature and misassemblies in recent teleost chromosome assemblies
Source: BMC Genomics. 2020 Mar 11;21:223. doi: 10.1186/s12864-020-6620-2 (PMC7066803; doi:10.1186/s12864-020-6620-2)

Mikalsen SO, Tausen M, í Kongsstovu S. Phylogeny of teleost connexins reveals highly inconsistent intra- and interspecies use of nomenclature and misassemblies in recent teleost chromosome assemblies.

Contents

Suppl. Fig. 1. Human (*Homo sapiens*) connexins. 3

Suppl. Fig. 2. Mouse (*Mus musculus*) connexins.......... 10

Suppl. Fig. 3. Opossum (*Monodelphis domestica*) connexins. 16

Suppl. Fig. 4. *GJC1like* and *GJA9* connexin sequences from other marsupials and platypus 22

Suppl. Fig. 5. Zebrafish (*Danio rerio*) connexins.......... 24

Suppl. Fig. 6. Japanese pufferfish (Fugu; *Takifugu rubripes*) connexins. 36

Suppl. Fig. 7. Green spotted pufferfish (*Tetraodon nigroviridis*) connexins. 49

Suppl. Fig. 8. Three-spined stickleback (*Gasterosteus aculeatus*) connexins. 62

Suppl. Fig. 9. Atlantic herring (*Clupea harengus*) connexins. 76

Suppl. Fig. 10. Atlantic cod (*Gadus morhua*) connexins. 90

Suppl. Fig. 11. Japanese eel (*Anguilla japonica*) connexins. 103

Suppl. Fig. 12. *Connexin39.2* ("*gjd2like*") from mammals. 116

Suppl. Fig. 13. Comparisons of human "*GJA4P*" against *connexin39.2* and *GJA4*. 119

Suppl. Fig. 13A. Alignment of conserved domains in human "*GJA4P*" (NG_026166) against *connexin39.2* ("*gjd2like*") in various species at protein level. 119

Suppl. Fig. 13B. Alignment of conserved domains in human "GJA4P" (NG_026166) against GJA4 (connexin37) from human and eel at protein level. 120

Suppl. Fig. 14. Expanded branches from the phylogenetic tree shown in Fig. 1. 121

Suppl. Fig. 14A. Expanded view of mammalian and teleost *GJA1* branch. 121

Suppl. Fig. 14B. Expanded view of the mammalian and teleost *GJA3* branch, and the associated teleost *cx39.9*. 121

Suppl. Fig. 14C. Expanded view of the mammalian and teleost *GJA4* branch. 122

Suppl. Fig. 14D. Expanded view of the mammalian and teleost *GJA5* branch. 122

Suppl. Fig. 14E. Expanded view of the mammalian and teleost *GJA9* and *GJA10* branches. 123

Suppl. Fig. 14F. Expanded view of teleost *cx34.5* and *cx32.2* branches. 124

Suppl. Fig. 14G. Expanded view of mammalian and teleost *GJB1*. 124

Suppl. Fig. 14H. Expanded view of mammalian *GJB2* and *GJB6*, and teleost *cx30.3*. 125

Suppl. Fig. 14I. Expanded view of mammalian *GJB3* and teleost *cx35.4*. 125

Suppl. Fig. 14J. Expanded view of mammalian *GJB4* and *GJB5*, and teleost *cx34.4*. 126

Suppl. Fig. 14K. Expanded view of mammalian and teleost *GJB7*. 126

Suppl. Fig. 14L. Expanded view of the teleost *cx28.6* group, and its relationship with *GJB3*/*GJB4*/*GJB5*. 127

Suppl. Fig. 14N. Expanded view of mammalian and teleost *GJC1* and teleost *cx43.4*. 128

Suppl. Fig. 14O. Expanded view of mammalian and teleost *GJC2*, and its relationship with *GJC1* and *cx43.4*. 129

Suppl. Fig. 14P. Expanded view of mammalian and teleost *Cx39.2*. 129

Suppl. Fig. 14Q. Expanded view over the central *GJD2* complex. 130

Suppl. Fig. 14R. Expanded view of mammalian and teleost *GJD3*. 130

Suppl. Fig. 14S. Expanded view of mammalian and teleost *GJD4*. 131

Suppl. Fig. 14T. Expanded view of teleost *cx36.7*. 131

Suppl. Fig. 15. Compressed phylogenetic tree illustrating long-branch attraction between *gjc3*, *gjd4* and *gje1* groups. 132

Suppl. Fig. 16. Searching for positions of connexins lacking in chromosomal assemblies. 134

Suppl. Fig. 16A. Problem in cod assembly of chromosome 20 at assumed position of *gja5*. 134

Suppl. Fig. 16B. Alignments with sequences from herring and stickleback point to the same area on cod chromosome 21, indicated expected position of *gja10-cx52.6*. 135

Suppl. Fig. 16C. Alignments of herring and stickleback scaffolds containing *cx52.6*. 136

Suppl. Fig. 17. A homogeneous and consistent nomenclature for gap junction protein genes 137

Suppl. Fig. 18. Schematic outline of the major procedures. 139

All data in this Supplemental Information have been collected and curated manually. Human errors and inconsistencies cannot be excluded. We would be grateful if detected errors and major inconsistencies are reported to us (sveinom@setur.fo).

Suppl. Fig. 1. Human (*Homo sapiens*) connexins.

The chronology of the sequences are according to the Greek nomenclature. Both the Greek nomenclature and the size nomenclature are indicated, and the GenBank accession number is given for each entry.

Yellow: Conserved domains as defined by Cruciani and Mikalsen (2007)
Green: Conserved cysteine codons (cysteine signature)
Grey: 15 nt added at the ends of the conserved domains
Turquoise: Splice site.
Other colors are explained where necessary.

```
>Hs-GJA1-Cx43-NM_000165
ATGGGTGACTGGAGCGCCTTAGGCAAACTCCTTGACAAGGTTCAAGCCTACTCAACTGCT
GGAGGGAAGGTGTGGCTGTCAGTACTTTTCATTTTCCGAATCCTGCTGCTGGGGACAGCG
GTTGAGTCAGCCTGGGGAGATGAGCAGTCTGCCTTTCGTTGTAACACTCAGCAACCTGGT
TGTGAAAATGTCTGCTATGACAAGTCTTTCCCAATCTCTCATGTGCGCTTCTGGGTCCTG
CAGATCATATTTGTGTCTGTACCCACACTCTTGTACCTGGCTCATGTGTTCTATGTGATG
CGAAAGGAAGAGAAACTGAACAAGAAAGAGGAAGAACTCAAGGTTGCCCAAACTGATGGT
GTCAATGTGGACATGCACTTGAAGCAGATTGAGATAAAGAAGTTCAAGTACGGTATTGAA
GAGCATGGTAAGGTGAAAATGCGAGGGGGTTGCTGCGAACCTACATCATCAGTATCCTC
TTCAAGTCTATCTTTGAGGTGGCCTTCTTGCTGATCCAGTGGTACATCTATGGATTCAGC
TTGAGTGCTGTTTACACTTGCAAAAGAGATCCCTGCCCACATCAGGTGGACTGTTTCCTC
TCTCGCCCCACGGAGAAAACCATCTTCATCATCTTCATGCTGGTGGTGTCCTTGGTGTCC
CTGGCCTTGAATATCATTGAACTCTTCTATGTTTTCTTCAAGGGCGTTAAGGATCGGGTT
AAGGGAAAGAGCGACCCTTACCATGCGACCAGTGGTGCGCTGAGCCCTGCCAAAGACTGT
GGGTCTCAAAAATATGCTTATTTCAATGGCTGCTCCTCACCAACCGCTCCCCTCTCGCCT
ATGTCTCCTCCTGGGTACAAGCTGGTTACTGGCGACAGAAACAATTCTTCTTGCCGCAAT
TACAACAAGCAAGCAAGTGAGCAAAACTGGGCTAATTACAGTGCAGAACAAAATCGAATG
GGGCAGGCGGGAAGCACCATCTCTAACTCCCATGCACAGCCTTTTGATTTCCCCGATGAT
AACCAGAATTCTAAAAAACTAGCTGCTGGACATGAATTACAGCCACTAGCCATTGTGGAC
CAGCGACCTTCAAGCAGAGCCAGCAGTCGTGCCAGCAGCAGACCTCGGCCTGATGACCTG
GAGATCTAG

>Hs-GJA3-Cx46-NM_021954
ATGGGCGACTGGAGCTTTCTGGGAAGACTCTTAGAAAATGCACAGGAGCACTCCACGGTC
ATCGGCAAGGTTTGGCTGACCGTGCTGTTCATCTTCCGCATCTTGGTGCTGGGGGCCGCG
GCGGAGGACGTGTGGGGCGATGAGCAGTCAGACTTCACCTGCAACACCCAGCAGCCGGGC
TGCGAGAACGTCTGCTACGACAGGGCCTTCCCCATCTCCCACATCCGCTTCTGGGCGCTG
CAGATCATCTTCGTGTCCACGCCCACCCTCATCTACCTGGGCCACGTGCTGCACATCGTG
CGCATGGAAGAGAAGAAGAAAGAGAGGGAGGAGGAGGAGCAGCTGAAGAGAGAGAGCCCC
AGCCCCAAGGAGCCACCGCAGGACAATCCCTCGTCGCGGGACGACCGCGGCAGGGTGCGC
ATGGCCGGGGCGCTGCTGCGGACCTACGTCTTCAACATCATCTTCAAGACGCTGTTCGAG
GTGGGCTTCATCGCCGGCCAGTACTTTCTGTACGGCTTCGAGCTGAAGCCGCTCTACCGC
TGCGACCGCTGGCCCTGCCCCAACACGGTGGACTGCTTCATCTCCAGGCCCACGGAGAAG
ACCATCTTCATCATCTTCATGCTGGCGGTGGCCTGCGCGTCCCTGCTGCTCAACATGCTG
GAGATCTACCACCTGGGCTGGAAGAAGCTCAAGCAGGGCGTGACCAGCCGCCTCGGCCCG
GACGCCTCCGAGGCCCCGCTGGGGACAGCCGATCCCCCGCCCCTGCCCCCCAGCTCCCGG
CCGCCCGCCGTTGCCATCGGGTTCCCACCCTACTATGCGCACACCGCTGCGCCCCTGGGA
CAGGCCCGCGCCGTGGGCTACCCCGGGGCCCCGCCACCAGCCGCGGACTTCAAACTGCTA
GCCCTGACCGAGGCGCGCGGAAAGGGCCAGTCCGCCAAGCTCTACAACGGCCACCACCAC
CTGCTGATGACTGAGCAGAACTGGGCCAACCAGGCGGCCGAGCGGCAGCCCCCGGCGCTC
AAGGCTTACCCGGCAGCGTCCACGCCTGCAGCCCCCAGCCCCGTCGGCAGCAGCTCCCCG
CCACTCGCGCACGAGGCTGAGGCGGGCGCGGCGCCCCTGCTGCTGGATGGGAGCGGCAGC
AGTCTGGAGGGGAGCGCCCTGGCAGGGACCCCCGAGGAGGAGGAGCAGGCCGTGACCACC
GCGGCCCAGATGCACCAGCCGCCCTTGCCCCTCGGAGACCCAGGTCGGGCCAGCAAGGCC
AGCAGGGCCAGCAGCGGGCGGGCCAGACCGGAGGACTTGGCCATCTAG

>Hs-GJA4-Cx37-NM_002060
ATGGGTGACTGGGGCTTCCTGGAGAAGTTGCTGGACCAGGTCCAGGAGCACTCGACCGTG
GTGGGTAAGATCTGGCTGACGGTGCTCTTCATCTTCCGCATCCTCATCCTGGGCCTGGCC
GGCGAGTCAGTGTGGGGTGACGAGCAATCAGATTTCGAGTGTAACACGGCCCAGCCAGGC
TGCACCAACGTCTGCTATGACCAGGCCTTCCCCATCTCCCACATCCGCTACTGGGTGCTG
CAGTTCCTCTTCGTCAGCACACCCACCCTGGTCTACCTGGGCCATGTCATTTACCTGTCT
CGGCGAGAAGAGCGGCTGCGGCAGAAGGAGGGGGAGCTGCGGGCACTGCCGGCCAAGGAC
CCACAGGTGGAGCGGGCGCTGGCGGCCGTAGAGCGTCAGATGGCCAAGATCTCGGTGGCA
```

GAAGATGGTCGCCTGCGCATCCGCGGAGCACTGATGGGCACCTATGTCGCCAGTGTGCTC
TGCAAGAGTGTGCTAGAGGCAGGCTTCCTCTATGGCCAGTGGCGCCTGTACGGCTGGACC
ATGGAGCCCGTGTTTGTGTGCCAGCGAGCACCCTGCCCCTACCTCGTGGACTGCTTTGTC
TCTCGCCCCACGGAGAAGACCATCTTCATCATCTTCATGTTGGTGGTTGGACTCATCTCC
CTGGTGCTTAACCTGCTGGAGTTGGTGCACCTGCTGTGTCGCTGCCTCAGCCGGGGGATG
AGGGCACGGCAAGGCCAAGACGCACCCCCGACCCAGGGCACCTCCTCAGACCCTTACACG
GACCAGGTCTTCTTCTACCTCCCCGTGGGCCAGGGGCCCTCATCCCCACCATGCCCCACC
TACAATGGGCTCTCATCCAGTGAGCAGAACTGGGCCAACCTGACCACAGAGGAGAGGCTG
GCGTCTTCCAGGCCCCCTCTCTTCCTGGACCCACCCCCTCAGAATGGCCAAAAACCCCCA
AGTCGTCCCAGCAGCTCTGCTTCTAAGAAGCAGTATGTATAG

>Hs-GJA5-Cx40-NM_005266
ATGGGCGATTGGAGCTTCCTGGGAAATTTCCTGGAGGAAGTACACAAGCACTCGACCGTG
GTAGGCAAGGTCTGGCTCACTGTCCTCTTCATATTCCGTATGCTCGTGCTGGGCACAGCT
GCTGAGTCTTCCTGGGGGGATGAGCAGGCTGATTTCCGGTGTGATACGATTCAGCCTGGC
TGCCAGAATGTCTGCTACGACCAGGCTTTCCCCATCTCCCACATTCGCTACTGGGTGCTG
CAGATCATCTTCGTCTCCACGCCCTCTCTGGTGTACATGGGCCACGCCATGCACACTGTG
CGCATGCAGGAGAAGCGCAAGCTACGGGAGGCCGAGAGGGCCAAAGAGGTCCGGGGCTCT
GGCTCTTACGAGTACCCGGTGGCAGAGAAGGCAGAACTGTCCTGCTGGGAGGAAGGGAAT
GGAAGGATTGCCCTCCAGGGCACTCTGCTCAACACCTATGTGTGCAGCATCCTGATCCGC
ACCACCATGGAGGTGGGCTTCATTGTGGGCCAGTACTTCATCTACGGAATCTTCCTGACC
ACCCTGCATGTCTGCCGCAGGAGTCCCTGTCCCCACCCGGTCAACTGTTACGTATCCCGG
CCCACAGAGAAGAATGTCTTCATTGTCTTTATGCTGGCTGTGGCTGCACTGTCCCTCCTC
CTTAGCCTGGCTGAACTCTACCACCTGGGCTGGAAGAAGATCAGACAGCGATTTGTCAAA
CCGCGGCAGCACATGGCTAAGTGCCAGCTTTCTGGCCCCTCTGTGGGCATAGTCCAGAGC
TGCACACCACCCCCCGACTTTAATCAGTGCCTGGAGAATGGCCCTGGGGGAAAATTCTTC
AATCCCTTCAGCAATAATATGGCCTCCCAACAAAACACAGACAACCTGGTCACCGAGCAA
GTACGAGGTCAGGAGCAGACTCCTGGGGAAGGTTTCATCCAGGTTCGTTATGGCCAGAAG
CCTGAGGTGCCCAATGGAGTCTCACCAGGTCACCGCCTTCCCCATGGCTATCATAGTGAC
AAGCGACGTCTTAGTAAGGCCAGCAGCAAGGCAAGGTCAGATGACCTATCAGTGTGA

>Hs-GJA6P-43pX-NG_007152 (Underlined: sequence somewhat extended relative to entry)
GTTGGTGACTGGAGTGCCTCAGGCACCTCCTAGAGAAGGTTCAAGCTTAGTCCCCAGCTG
GAGGTAAGGTGTGGTTCTCAGTCCTTTTCATTTCCCCAATCCTGCTCCTGAGTACTGCAG
TTGAGTCAGCCTGGGATAATGAGCAGTTTGCCTTTCATTGAACACTCAACAGCCTGGTTG
TGAAAATGTCTGCTATGACCAGTCTTTGCCAATCTCCCATGTATGCTTCTGGGTTCTGCA
GATAATACTTGTGTCTCTTCCCCCACTCTTGTACCTGACACACATGTTCTACGTGATGTG
AAAAGAAGAGAAGTTGAACAGGCAATTGGGAGAACTCGAAGTTGCCCAAATTGATGATGT
CAGTGTGGAGATGCACTTGCAGGGAATTGAAATAAAGAAGTTCAAGTGTGGCCTTGAGAA
ACATGGAAGGGTGAGAATCCCAGGGAGCTTGCTGCGAACGTGTACCATTGGTGTCATCTT
CAGGCCTCTGTTTGAGGTGGCCTTCCTGATGATCCCGTGGTCTGTCTATGGATTCAGCCT
AAGTGTGGTTTACACTTGCAAACGAGATCTTTCCCCACATTAAGTGGACTGCTTACTCTC
TCGCCCCCGGAGAAAAGCACCTTCATCATCTTCATGCTGGTGGTGTCCTTGGTGTCTCTT
GCCTTGAACATCATTGCGTTATTCAGTGTCTCATTTAAGAGCATTTAAGGATCATGTGAA
GGATCAGGAGAGTGATACACCTGGCCTGCAGAGTCCCTCCAATGGCTCCCATATCAACTCT
TCCTCTCTCCACCATGTCCCCTCCTGGGGACAAGCTAGTTCCTGGAGAAAGAAACAATTC
CTCTTGCTGTAGGCACAACAAGGAAGTGAGCAAAAACCCCTTCTAGTTACAGTGCATAGCA
AGATTGAATGGAGCAGGCAGGAAGTACCCCCTCTGACTCCCAGTCTTTTGATTTCCCCTGC
TGATAAACAGAGTTCTTAAACAAAAGCAAAAACCTAGCTACTGGGCACTAGCCATGGTAG
GCCAGCAGCCATCCTGCAGAGCCAGTAGTCATGCCAGCAGCAGCCTGATGACCTAGAGAT
CAAG

>Hs-GJA8-Cx50-NM_005267
ATGGGCGACTGGAGTTTCCTGGGGAACATCTTGGAGGAGGTGAATGAGCACTCCACCGTC
ATCGGCAGAGTCTGGCTCACCGTGCTTTTCATCTTCCGGATCCTCATCCTTGGCACGGCC
GCAGAGTTCGTGTGGGGGGATGAGCAATCCGACTTCGTGTGCAACACCCAGCAGCCTGGC
TGCGAGAACGTCTGCTACGACGAGGCCTTTCCCATCTCCCACATTCGCCTCTGGGTGCTG
CAGATCATCTTCGTCTCCACCCCGTCCCTGATGTACGTGGGGCACGCGGTGCACTACGTC
CGCATGGAGGAGAAGCGCAAAAGCCGCGAGGCGGAGGAGCTGGGCCAGCAGGCGGGGACT
AACGGCGGCCCGGACCAGGGCAGCGTCAAGAAGAGCAGCGGCAGCAAAGGCACTAAGAAG
TTCCGGCTGGAGGGGACCCTGCTGAGGACCTACATCTGCCACATCATCTTCAAGACCCTC
TTTGAAGTGGGCTTCATCGTGGGCCACTACTTCCTGTACGGGTTCCGGATCCTGCCTCTG
TACCGCTGCAGCCGGTGGCCCTGCCCCAATGTGGTGGACTGCTTCGTGTCCCGGCCCACG
GAGAAAACCATCTTCATCCTGTTCATGTTGTCTGTGGCCTCTGTGTCCCTATTCCTCAAC
GTGATGGAGTTGGGCCACCTGGGCCTGAAGGGGATCCGGTCTGCCTTGAAGAGGCCTGTA
GAGCAGCCCCTGGGGGAGATTCCTGAGAAATCCCTCCACTCCATTGCTGTCTCCTCCATC
CAGAAAGCCAAGGGCTATCAGCTCCTAGAAGAAGAGAAAATCGTTTCCCACTATTTCCCC
TTGACCGAGGTTGGGATGGTGGAGACCAGCCCACTGCCTGCCAAGCCTTTCAATCAGTTC

GAGGAGAAGATCAGCACAGGACCCCTGGGGGACTTGTCCCGGGGCTACCAAGAGACACTG
CCTTCCTACGCTCAGGTGGGGGCACAAGAAGTGGAGGGCGAGGGGCCGCCTGCAGAGGAG
GGAGCCGAACCCGAGGTGGGAGAGAAGAAGGAGGAAGCAGAGAGGCTGACCACGGAGGAG
CAGGAGAAGGTGGCCGTGCCAGAGGGGGAGAAAGTAGAGACCCCCGGAGTGGATAAGGAG
GGTGAAAAAGAAGAGCCGCAGTCGGAGAAGGTGTCAAAGCAAGGGCTGCCAGCTGAGAAG
ACACCTTCACTCTGTCCAGAGCTGACAACAGATGATGCCAGACCCCTGAGCAGGCTAAGC
AAAGCCAGCAGCCGAGCCAGGTCAGACGATCTAACCGTATGA

>Hs-GJA9-Cx58-NM_030772
ATGGGGGACTGGAATCTCCTTGGAGATACTCTGGAGGAAGTTCACATCCACTCCACCATG
ATTGGAAAGATCTGGCTCACCATCCTGTTCATATTTCGAATGCTTGTTCTGGGTGTAGCA
GCTGAAGATGTCTGGAATGATGAGCAGTCTGGCTTCATCTGCAATACAGAACAACCAGGC
TGCAGAAATGTATGCTACGACCAGGCCTTTCCTATCTCCCTCATTAGATACTGGGTTCTG
CAGGTGATATTTGTGTCTTCACCATCCCTGGTCTACATGGGCCATGCATTGTACCGACTG
AGAGTTCTTGAGGAAGAGAGGCAAAGGATGAAAGCTCAGTTAAGAGTAGAACTGGAGGAG
GTAGAGTTTGAAATGCCTAGGGATCGGAGGAGATTGGAGCAAGAGCTTTGTCAGCTGGAG
AAAAGGAAACTAAATAAAGCTCCACTCAGAGGAACCTTGCTTTGCACTTATGTGATACAC
ATTTTCACTCGCTCTGTGGTTGAAGTTGGATTCATGATTGGACAGTACCTTTTATATGGA
TTTCACTTAGAGCCGCTATTTAAGTGCCATGGCCACCCGTGTCCAAATATAATCGACTGT
TTTGTCTCAAGACCAACAGAAAAGACAATATTCCTATTATTTATGCAATCTATAGCCACT
ATTTCACTTTTCTTAAACATTCTTGAAATTTTCCACCTAGGTTTTAAAAAGATTAAAAGA
GGGCTTTGGGGAAAATACAAGTTGAAGAAGGAACATAATGAATTCCATGCAAACAAGGCA
AAACAAAATGTAGCCAAATACCAGAGCACATCTGCAAATTCACTGAAGCGACTCCCTTCT
GCCCCTGATTATAATCTGTTAGTGGAAAAGCAAACACACACTGCAGTGTACCCTAGTTTA
AATTCATCTTCTGTATTCCAGCCAAATCCTGACAATCATAGTGTAAATGATGAGAAATGC
ATTTTGGATGAACAGGAAACTGTACTTTCTAATGAGATTTCCACACTTAGTACTAGTTGT
AGTCATTTTCAACACATCAGTTCAAACAATAACAAAGACACTCATAAAATATTTGGAAAA
GAACTTAATGGTAACCAGTTAATGGAAAAAAGAGAAACTGAAGGCAAAGACAGCAAAAGG
AACTACTACTCTAGAGGTCACCGTTCTATTCCAGGTGTTGCTATAGATGGAGAGAACAAC
ATGAGGCAGTCACCCCAAACAGTTTTCTCCTTGCCAGCTAACTGCGATTGGAAACCGCGG
TGGCTTAGAGCTACATGGGGTTCCTCTACAGAACATGAAAACCGGGGGTCACCTCCTAAA
GGTAACCTCAAGGGCCAGTTCAGAAAGGGCACAGTCAGAACCCTTCCTCCTTCACAAGGA
GATTCTCAATCACTTGACATTCCAAACACTGCTGATTCTTTGGGAGGGCTGTCCTTTGAG
CCAGGGTTGGTCAGAACCTGTAATAATCCTGTTTGTCCTCCAAATCACGTAGTGTCCCTA
ACGAACAATCTCATTGGTAGGCGGGTTCCCACAGATCTTCAGATCTAA

>Hs-GJA10-Cx62-NM_032602
ATGGGGGACTGGAACTTATTGGGTGGCATCCTAGAGGAAGTTCACTCCCACTCAACCATA
GTGGGGAAAATCTGGCTGACCATCCTCTTCATCTTCCGAATGCTGGTACTTCGTGTGGCT
GCTGAGGATGTCTGGGATGATGAACAGTCAGCATTTGCCTGCAACACCCGGCAGCCAGGT
TGCAACAATATCTGTTATGATGATGCATTCCCTATCTCTTTGATCAGGTTCTGGGTTTTA
CAGATCATCTTTGTGTCTTCTCCTTCTTTGGTCTATATGGGCCATGCACTTTATAGGCTC
AGGGCCTTTGAGAAAGACAGGCAGAGGAAAAAGTCACACCTTAGAGCCCAGATGGAGAAT
CCAGATCTTGACTTGGAGGAGCAGCAAAGAATAGATAGGGAACTGAGGAGGTTAGAGGAG
CAGAAGAGGATCCATAAAGTCCCTCTGAAAGGATGTCTGCTGCGTACTTATGTCTTACAC
ATCTTGACCAGATCTGTGCTGGAAGTAGGATTCATGATAGGCCAATATATTCTCTATGGG
TTTCAAATGCACCCCCTTTACAAATGCACTCAACCTCCTTGCCCCAATGCGGTGGATTGC
TTTGTATCCAGGCCCACTGAGAAGACAATTTTCATGCTTTTTATGCACAGCATTGCAGCC
ATTTCCTTGTTACTCAATATACTGGAAATATTTCATCTAGGCATCAGAAAAATTATGAGG
ACACTTTATAAGAAATCCAGCAGTGAGGGCATTGAGGATGAAACAGGCCCTCCATTCCAT
TTGAAGAAATATTCTGTGGCCCAGCAGTGTATGATTTGCTCTTCATTGCCTGAAAGAATC
TCTCCACTTCAAGCTAACAATCAACAGCAAGTCATTCGAGTTAATGTGCCAAAGTCTAAA
ACCATGTGGCAAATCCCACAGCCAAGGCAACTTGAAGTAGACCCTTCCAATGGGAAAAAG
GACTGGTCTGAGAAGGATCAGCATAGCGGACAGCTCCATGTTCACAGCCCGTGTCCCTGG
GCTGGCAGTGCTGGAAATCAGCACCTGGGACAGCAATCAGACCATTCCTCATTTGGCCTG
CAGAATACAATGTCTCAGTCCTGGCTAGGTACAACTACGGCTCCTAGAAACTGTCCATCC
TTTGCAGTAGGAACCTGGGAGCAGTCCCAGGACCCAGAACCCTCAGGTGAGCCTCTCACA
GATCTTCATAGTCACTGCAGAGACAGTGAAGGCAGCATGAGAGAGAGTGGGGTCTGGATA
GACAGATCTCGCCCAGGCAGTCGCAAGGCCAGCTTTCTGTCCAGATTGTTGTCTGAAAAG
CGACATCTGCACAGTGACTCAGGAAGCTCTGGTTCTCGGAATAGCTCCTGCTTGGATTTT
CCTCACTGGGAAAACAGCCCCTCACCTCTGCCTTCAGTCACTGGGCACAGAACATCAATG
GTAAGACAGGCAGCCCTACCGATCATGGAACTATCACAAGAGCTGTTCCATTCTGGATGC
TTTCTTTTTCCTTTCTTTCTTCCTGGGGTGTGTATGTATGTTTGTGTTGACAGAGAGGCA
GATGGAGGGGGAGATTATTTATGGAGAGATAAAATTATTCATTCGATACATTCAGTTAAA
TTCAATTCATAA

>Hs-GJB1-Cx32-NM_001097642
ATGAACTGGACAGGTTTGTACACCTTGCTCAGTGGCGTGAACCGGCATTCTACTGCCATT

GGCCGAGTATGGCTCTCGGTCATCTTCATCTTCAGAATCATGGTGCTGGTGGTGGCTGCA
GAGAGTGTGTGGGGTGATGAGAAATCTTCCTTCATCTGCAACACACTCCAGCCTGGCTGC
AACAGCGTTTGCTATGACCAATTCTTCCCCATCTCCCATGTGCGGCTGTGGTCCCTGCAG
CTCATCCTAGTTTCCACCCCAGCTCTCCTCGTGGCCATGCACGTGGCTCACCAGCAACAC
ATAGAGAAGAAAATGCTACGGCTTGAGGGCCATGGGGACCCCCTACACCTGGAGGAGGTG
AAGAGGCACAAGGTCCACATCTCAGGGACACTGTGGTGGACCTATGTCATCAGCGTGGTG
TTCCGGCTGTTGTTTGAGGCCGTCTTCATGTATGTCTTTTATCTGCTCTACCCTGGCTAT
GCCATGGTGCGGCTGGTCAAGTGCGACGTCTACCCCTGCCCCAACACAGTGGACTGCTTC
GTGTCCCGCCCCACCGAGAAAACCGTCTTCACCGTCTTCATGCTAGCTGCCTCTGGCATC
TGCATCATCCTCAATGTGGCCGAGGTGGTGTACCTCATCATCCGGGCCTGTGCCCGCCGA
GCCCAGCGCCGCTCCAATCCACCTTCCCGCAAGGGCTCGGGCTTCGGCCACCGCCTCTCA
CCTGAATACAAGCAGAATGAGATCAACAAGCTGCTGAGTGAGCAGGATGGCTCCCTGAAA
GACATACTGCGCCGCAGCCCTGGCACCGGGGCTGGGCTGGCTGAAAAGAGCGACCGCTGC
TCGGCCTGCTGA

>Hs-GJB2-Cx26-NM_004004
ATGGATTGGGGCACGCTGCAGACGATCCTGGGGGGTGTGAACAAACACTCCACCAGCATT
GGAAAGATCTGGCTCACCGTCCTCTTCATTTTTCGCATTATGATCCTCGTTGTGGCTGCA
AAGGAGGTGTGGGGAGATGAGCAGGCCGACTTTGTCTGCAACACCCTGCAGCCAGGCTGC
AAGAACGTGTGCTACGATCACTACTTCCCCATCTCCCACATCCGGCTATGGGCCCTGCAG
CTGATCTTCGTGTCCACGCCAGCGCTCCTAGTGGCCATGCACGTGGCCTACCGGAGACAT
GAGAAGAAGAGGAAGTTCATCAAGGGGGAGATAAAGAGTGAATTTAAGGACATCGAGGAG
ATCAAAACCCAGAAGGTCCGCATCGAAGGCTCCCTGTGGTGGACCTACACAAGCAGCATC
TTCTTCCGGGTCATCTTCGAAGCCGCCTTCATGTACGTCTTCTATGTCATGTACGACGGC
TTCTCCATGCAGCGGCTGGTGAAGTGCAACGCCTGGCCTTGTCCCAACACTGTGGACTGC
TTTGTGTCCCGGCCCACGGAGAAGACTGTCTTCACAGTGTTCATGATTGCAGTGTCTGGA
ATTTGCATCCTGCTGAATGTCACTGAATTGTGTTATTTGCTAATTAGATATTGTTCTGGG
AAGTCAAAAAAGCCAGTTTAA

>Hs-GJB3-Cx31-NM_024009
ATGGACTGGAAGACACTCCAGGCCCTACTGAGCGGTGTGAACAAGTACTCCACAGCGTTC
GGGCGCATCTGGCTGTCCGTGGTGTTCGTCTTCCGGGTGCTGGTATACGTGGTGGCTGCA
GAGCGCGTGTGGGGGGATGAGCAGAAGGACTTTGACTGCAACACCAAGCAGCCCGGCTGC
ACCAACGTCTGCTACGACAACTACTTCCCCATCTCCAACATCCGCCTCTGGGCCCTGCAG
CTCATCTTCGTCACATGCCCCTCGCTGCTGGTCATCCTGCACGTGGCCTACCGTGAGGAG
CGGGAGCGCCGGCACCGCCAGAAACACGGGGACCAGTGCGCCAAGCTGTACGACAACGCA
GGCAAGAAGCACGGAGGCCTGTGGTGGACCTACCTGTTCAGCCTCATCTTCAAGCTCATC
ATTGAGTTCCTCTTCCTCTACCTGCTGCACACTCTCTGGCATGGCTTCAATATGCCGCGC
CTGGTGCAGTGTGCCAACGTGGCCCCCTGCCCCAACATCGTGGACTGCTACATTGCCCGA
CCTACCGAGAAGAAAATCTTCACCTACTTCATGGTGGGCGCCTCCGCCGTCTGCATCGTA
CTCACCATCTGTGAGCTCTGCTACCTCATCTGCCACAGGGTCCTGCGAGGCCTGCACAAG
GACAAGCCTCGAGGGGGTTGCAGCCCCTCGTCCTCCGCCAGCCGAGCTTCCACCTGCCGC
TGCCACCACAAGCTGGTGGAGGCTGGGGAGGTGGATCCAGACCCAGGCAATAACAAGCTG
CAGGCTTCAGCACCCAACCTGACCCCCATCTGA

>Hs-GJB4-Cx30.3-NM_153212
ATGAACTGGGCATTTCTGCAGGGCCTGCTGAGTGGCGTGAACAAGTACTCCACAGTGCTG
AGCCGCATCTGGCTGTCTGTGGTGTTCATCTTTCGTGTGCTGGTGTACGTGGTGGCAGCG
GAGGAGGTGTGGGACGATGAGCAGAAGGACTTTGTCTGCAACACCAAGCAGCCCGGCTGC
CCCAACGTCTGCTATGACGAGTTCTTCCCCGTGTCCCACGTGCGCCTCTGGGCCCTACAG
CTCATCCTGGTCACGTGCCCCTCACTGCTCGTGGTCATGCACGTGGCCTACCGCGAGGAA
CGCGAGCGCAAGCACCACCTGAAACACGGGCCCAATGCCCCGTCCCTGTACGACAACCTG
AGCAAGAAGCGGGGCGGACTGTGGTGGACGTACTTGCTGAGCCTCATCTTCAAGGCCGCC
GTGGATGCTGGCTTCCTCTATATCTTCCACCGCCTCTACAAGGATTATGACATGCCCCGC
GTGGTGGCCTGCTCCGTGGAGCCTTGCCCCCACACTGTGGACTGTTACATCTCCCGGCCC
ACGGAGAAGAAGGTCTTCACCTACTTCATGGTGACCACAGCTGCCATCTGCATCCTGCTC
AACCTCAGTGAAGTCTTCTACCTGGTGGGCAAGAGGTGCATGGAGATCTTCGGCCCCAGG
CACCGGCGGCCTCGGTGCCGGGAATGCCTACCCGATACGTGCCCACCATATGTCCTCTCC
CAGGGAGGGCACCCTGAGGATGGGAACTCTGTCCTAATGAAGGCTGGGTCGGCCCCAGTG
GATGCAGGTGGGTATCCATAA

>Hs-GJB5-Cx31.1-NM_005268
ATGAACTGGAGTATCTTTGAGGGACTCCTGAGTGGGGTCAACAAGTACTCCACAGCCTTT
GGGCGCATCTGGCTGTCTCTGGTCTTCATCTTCCGCGTGCTGGTGTACCTGGTGACGGCC
GAGCGTGTGTGGAGTGATGACCACAAGGACTTCGACTGCAATACTCGCCAGCCCGGCTGC
TCCAACGTCTGCTTTGATGAGTTCTTCCCTGTGTCCCATGTGCGCCTCTGGGCCCTGCAG
CTTATCCTGGTGACATGCCCCTCACTGCTCGTGGTCATGCACGTGGCCTACCGGGAGGTT
CAGGAGAAGAGGCACCGAGAAGCCCATGGGGAGAACAGTGGGCGCCTCTACCTGAACCCC

GGCAAGAAGCGGGGTGGGCTCTGGTGGACATATGTCTGCAGCCTAGTGTTCAAGGCGAGC
GTGGACATCGCCTTTCTCTATGTGTTCCACTCATTCTACCCCAAATATATCCTCCCTCCT
GTGGTCAAGTGCCACGCAGATCCATGTCCCAATATAGTGGACTGCTTCATCTCCAAGCCC
TCAGAGAAGAACATTTTCACCCTCTTCATGGTGGCCACAGCTGCCATCTGCATCCTGCTC
AACCTCGTGGAGCTCATCTACCTGGTGAGCAAGAGATGCCACGAGTGCCTGGCAGCAAGG
AAAGCTCAAGCCATGTGCACAGGTCATCACCCCCACGGTACCACCTCTTCCTGCAAACAA
GACGACCTCCTTTCGGGTGACCTCATCTTTCTGGGCTCAGACAGTCATCCTCCTCTCTTA
CCAGACCGCCCCCGAGACCATGTGAAGAAAACCATCTTGTGA

>Hs-GJB6-Cx30-NM_001110219
ATGGATTGGGGGACGCTGCACACTTTCATCGGGGGTGTCAACAAACACTCCACCAGCATC
GGGAAGGTGTGGATCACAGTCATCTTTATTTTCCGAGTCATGATCCTCGTGGTGGCTGCC
CAGGAAGTGTGGGGTGACGAGCAAGAGGACTTCGTCTGCAACACACTGCAACCGGGATGC
AAAAATGTGTGCTATGACCACTTTTTCCCGGTGTCCCACATCCGGCTGTGGGCCCTCCAG
CTGATCTTCGTCTCCACCCCAGCGCTGCTGGTGGCCATGCATGTGGCCTACTACAGGCAC
GAAACCACTCGCAAGTTCAGGCGAGGAGAGAAGAGGAATGATTTCAAAGACATAGAGGAC
ATTAAAAAGCAGAAGGTTCGGATAGAGGGGTCGCTGTGGTGGACGTACACCAGCAGCATC
TTTTTCCGAATCATCTTTGAAGCAGCCTTTATGTATGTGTTTTACTTCCTTTACAATGGG
TACCACCTGCCCTGGGTGTTGAAATGTGGGATTGACCCCTGCCCCAACCTTGTTGACTGC
TTTATTTCTAGGCCAACAGAGAAGACCGTGTTTACCATTTTTATGATTTCTGCGTCTGTG
ATTTGCATGCTGCTTAACGTGGCAGAGTTGTGCTACCTGCTGCTGAAAGTGTGTTTTAGG
AGATCAAAGAGAGCACAGACGCAAAAAAATCACCCCAATCATGCCCTAAAGGAGAGTAAG
CAGAATGAAATGAATGAGCTGATTTCAGATAGTGGTCAAAATGCAATCACAGGTTTCCCA
AGCTAA

>Hs-GJB7-Cx25-NM_198568
ATGAGTTGGATGTTCCTCAGAGATCTCCTGAGTGGAGTAAATAAATACTCCACTGGGACT
GGATGGATTTGGCTGGCTGTCGTGTTTGTCTTCCGTTTGCTGGTCTACATGGTGGCAGCA
GAGCACGTGTGGAAAGATGAGCAGAAAGAGTTTGAGTGCAACAGTAGACAGCCCGGTTGC
AAAAATGTGTGTTTTGATGACTTCTTCCCCATTTCCCAAGTCAGACTTTGGGCCTTACAA
CTGATAATGGTCTCCACACCTTCACTTCTGGTGGTTTTACATGTAGCCTATCATGAGGGT
AGAGAGAAAAGGCACAGAAAGAAACTCTATGTCAGCCCAGGTACAATGGATGGGGGCCTA
TGGTACGCTTATCTTATCAGCCTCATTGTTAAAACTGGTTTTGAAATTGGCTTCCTTGTT
TTATTTTATAAGCTATATGATGGCTTTAGTGTTCCCTACCTTATAAAGTGTGATTTGAAG
CCTTGTCCCAACACTGTGGACTGCTTCATCTCCAAACCCACTGAGAAGACGATCTTCATC
CTCTTCTTGGTCATCACCTCATGCTTGTGTATTGTGTTGAATTTCATTGAACTGAGTTTT
TTGGTTCTCAAGTGCTTTATTAAGTGCTGTCTCCAAAAAATATTTAAAAAAACCTCAAGTC
CTCAGTGTGTGA

>Hs-GJC1-Cx45-NM_005497
ATGAGTTGGAGCTTCCTGACTCGCCTGCTAGAGGAGATTCACAACCATTCCACATTTGTG
GGGAAGATCTGGCTCACTGTTCTGATTGTCTTCCGGATCGTCCTTACAGCTGTAGGAGGA
GAATCCATCTATTACGATGAGCAAAGCAAATTTGTGTGCAACACAGAACAGCCGGGCTGT
GAGAATGTCTGTTATGATGCGTTTGCACCTCTCTCCCATGTACGCTTCTGGGTGTTCCAG
ATCATCCTGGTGGCAACTCCCTCTGTGATGTACCTGGGCTATGCTATCCACAAGATTGCC
AAAATGGAGCACGGTGAAGCAGACAAGAAGGCAGCTCGGAGCAAGCCCTATGCAATGCGC
TGGAAACAACACCGGGCTCTGGAAGAAACGGAGGAGGACAACGAAGAGGATCCTATGATG
TATCCAGAGATGGAGTTAGAAAGTGATAAGGAAAATAAAGAGCAGAGCCAACCCAAACCT
AAGCATGATGGCCGACGACGGATTCGGGAAGATGGGCTCATGAAAATCTATGTGCTGCAG
TTGCTGGCAAGGACCGTGTTTGAGGTGGGTTTTCTGATAGGGCAGTATTTTCTGTATGGC
TTCCAAGTCCACCCGTTTTATGTGTGCAGCAGACTTCCTTGTCCTCATAAGATAGACTGC
TTTATTTCTAGACCCACTGAAAAGACCATCTTCCTTCTGATAATGTATGGTGTTACAGGC
CTTTGCCTCTTGCTTAACATTTGGGAGATGCTTCATTTAGGGTTTGGGACCATTCGAGAC
TCACTAAACAGTAAAAGGAGGGAACTTGAGGATCCGGGTGCTTATAATTATCCTTTCACT
TGGAATACACCATCTGCTCCCCCTGGCTATAACATTGCTGTCAAACCAGATCAAATCCAG
TACACCGAACTGTCCAATGCTAAGATCGCCTACAAGCAAAACAAGGCCAACACAGCCCAG
GAACAGCAGTATGGCAGCCATGAGGAGAACCTCCCAGCTGACCTGGAGGCTCTGCAGCGG
GAGATCAGGATGGCTCAGGAACGCTTGGATCTGGCAGTTCAGGCCTACAGTCACCAAAAC
AACCCTCATGGTCCCCGGGAGAAGAAGGCCAAAGTGGGGTCCAAAGCTGGGTCCAACAAA
AGCACTGCCAGTAGCAAATCAGGGGATGGGAAGACCTCCGTCTGGATTTAA

>Hs-GJC2-Cx47-NM_020435
ATGAGCTGGAGCTTCCTGACGCGGCTGCTGGAGGAGATCCACAACCACTCCACCTTCGTG
GGCAAGGTGTGGCTCACGGTGCTGGTGGTCTTCCGCATCGTGCTGACGGCTGTGGGCGGC
GAGGCCATCTACTCGGACGAGCAGGCCAAGTTCACTTGCAACACGCGGCAGCCAGGCTGC
GACAACGTCTGCTATGACGCCTTCGCGCCCCTGTCGCACGTGCGCTTCTGGGTCTTCCAG
ATTGTGGTCATCTCCACGCCCTCGGTCATGTACCTGGGCTACGCCGTGCACCGCCTGGCC
CGTGCGTCTGAGCAGGAGCGGCGCCGCGCCCTCCGCCGCCGCCCGGGGCCACGCCGCGCG

CCCCGAGCGCACCTGCCGCCCCCGCACGCCGGCTGGCCTGAGCCCGCCGACCTGGGCGAG
GAGGAGCCCATGCTGGGCCTGGGCGAGGAGGAGGAGGAGGAGGAGACGGGGGCAGCCGAG
GGCGCCGGCGAGGAAGCGGAGGAGGCAGGCGCGGAGGAGGCGTGCACTAAGGCGGTCGGC
GCTGACGGCAAGGCGGCAGGGACCCCGGGCCCGACCGGGCAACACGATGGGCGGAGGCGC
ATCCAGCGGGAGGGCCTGATGCGCGTGTACGTGGCCCAGCTGGTGGCCAGGGCAGCTTTC
GAGGTGGCCTTCCTGGTGGGCCAGTACCTGCTGTACGGCTTCGAGGTGCGACCGTTCTTT
CCCTGCAGCCGCCAGCCCTGCCCGCACGTGGTGGACTGCTTCGTGTCGCGCCCTACTGAA
AAGACGGTCTTCCTGCTGGTTATGTACGTGGTCAGCTGCCTGTGCCTGCTGCTCAACCTC
TGTGAGATGGCCCACCTGGGCTTGGGCAGCGCGCAGGACGCGGTGCGCGGCCGCCGCGGC
CCCCCGGCCTCCGCCCCCGCCCCCGCGCCGCGGCCCCCGCCCTGCGCCTTCCCTGCGGCG
GCCGCTGGCTTGGCCTGCCCGCCCGACTACAGCCTGGTGGTGCGGGCGGCCGAGCGCGCT
CGGGCGCATGACCAGAACCTGGCAAACCTGGCCCTGCAGGCGCTGCGCGACGGGGCAGCG
GCTGGGGACCGCGACCGGGACAGTTCGCCGTGCGTCGGCCTCCCTGCGGCCTCCCGGGGG
CCCCCCAGAGCAGGCGCCCCCGCGTCCCGGACGGGCAGTGCTACCTCTGCGGGCACTGTC
GGGGAGCAGGGCCGGCCCGGCACCCACGAGCGGCCAGGAGCCAAGCCCAGGGCTGGCTCC
GAGAAGGGCAGTGCCAGCAGCAGGGACGGGAAGACCACCGTGTGGATCTGA

>Hs-GJC3-Cx31.3-NM_181538 Splice site.
ATGTGTGGCAGGTTCCTGCGGCGGCTGCTGGCGGAGGAGAGCCGGCGCTCCACCCCCGTG
GGGCGCCTCTTGCTTCCCGTGCTCCTGGGATTCCGCCTTGTGCTGCTGGCTGCCAGTGGG
CCTGGAGTCTATGGTGATGAGCAGAGTGAATTCGTGTGTCACACCCAGCAGCCGGGCTGC
AAGGCTGCCTGCTTCGATGCCTTCCACCCCCTCTCCCCGCTGCGTTTCTGGGTCTTCCAG
GTCATCTTGGTGGCTGTACCCAGCGCCCTCTATATGGGTTTCACTCTGTATCACGTGATC
TGGCACTGGGAATTATCAGGAAAGGGGAAGGAGGAGGAGACCCTGATCCAGGGACGGGAG
GGCAACACAGATGTCCCAGGGGCTGGAAGCCTCAGGCTGCTCTGGGCTTATGTGGCTCAG
CTGGGGGCTCGGCTTGTCCTGGAGGGGGCAGCCCTGGGGTTGCAGTACCACCTGTATGGG
TTCCAGATGCCCAGCTCCTTTGCATGTCGCCGAGAACCTTGCCTTGGTAGTATAACCTGC
AATCTGTCCCGCCCCTCTGAGAAGACCATTTTCCTAAAGACCATGTTTGGAGTCAGCGGT
TTCTGTCTCTTGTTTACTTTTTTGGAGCTTGTGCTTCTGGGTTTGGGGAGATGGTGGAGG
ACCTGGAAGCACAAATCTTCCTCTTCTAAATACTTCCTAACTTCAGAGAGCACCAGAAGA
CACAAGAAAGCAACCGATAGCCTCCCAGTGGTGGAAACCAAAGAGCAATTTCAAGAAGCA
GTTCCAGGAAGAAGCTTAGCCCAGGAAAAACAAAGACCAGTTGGACCCAGAGATGCCTGA

>Hs-GJD2-Cx36-NM_020660 Splice site.
ATGGGGGAATGGACCATCTTGGAGAGGCTGCTAGAAGCCGCGGTGCAGCAGCACTCCACT
ATGATCGGGAGGATCCTGTTGACTGTGGTGGTGATCTTCCGGATCCTCATTGTGGCCATT
GTGGGGGAGACGGTGTACGATGATGAGCAGACCATGTTTGTGTGCAACACCCTGCAGCCC
GGCTGTAACCAGGCCTGCTATGACCGCGCCTTCCCCATCTCCCACATACGTTACTGGGTC
TTCCAGATCATAATGGTGTGTACCCCCAGTCTTTGCTTCATCACCTACTCTGTGCACCAG
TCCGCCAAGCAGCGAGAACGCCGCTACTCTACAGTCTTCCTAGCCCTGGACAGAGACCCC
CCTGAGTCCATAGGAGGTCCTGGAGGAACTGGGGGTGGGGGCAGTGGTGGGGGCAAACGA
GAAGATAAGAAGTTGCAAAATGCTATTGTGAATGGGGTGCTGCAGAACACAGAGAACACC
AGTAAGGAGACAGAGCCAGATTGTTTAGAGGTTAAGGAGCTGACTCCACACCCATCAGGT
CTACGCACTGCATCAAAATCCAAGCTCAGAAGGCAGGAAGGCATCTCCCGCTTCTACATT
ATCCAAGTGGTGTTCCGAAATGCCCTGGAAATTGGGTTCCTGGTTGGCCAATATTTTCTC
TATGGCTTTAGTGTCCCAGGGTTGTATGAGTGTAACCGCTACCCCTGCATCAAGGAGGTG
GAATGTTATGTGTCCCGGCCAACTGAGAAGACTGTCTTTCTAGTGTTCATGTTTGCTGTA
AGTGGCATCTGTGTTGTGCTCAACCTGGCTGAACTCAACCACCTGGGATGGCGCAAGATC
AAGCTGGCTGTGCGAGGGGCTCAGGCCAAGAGAAAGTCAATCTATGAGATTCGTAACAAG
GACCTGCCAAGGGTCAGTGTTCCCAATTTTGGCAGGACTCAGTCCAGTGACTCTGCCTAT
GTGTGA

>Hs-“GJA4P”-39.2P-NG_026166 This sequence is in reality Cx39.2P (“GJD2like”)
ATGAGCGACTGGTCATTCCTGGGCTGGCTCCTGACCCGAGTGCAGAACGATTCCACCGTG
GTTGGCAAGGTATGGCTCACTGTCCTGGTCTTACACATCCTGCTTGTCGCCCTGCTGGGA
AGTGCTGTCTGTGGGATGAGCACTGCAAGTTCATCTGCAATACCCTGCGGCCTGGCTGCA
CCAATGACCACTTCTCCCACTTCCGCTGGGGCTTTCCAGATTGTGCTGGTGGCCGTACCC
TCCATCTTCTTTGTTGTCTGTGTGCTGCACTAGATGGTGAATGGGAGACAGTGGATGTGG
AGAGGGGGTACCTGCTGGAAACCGTGCAAGAGCTGGCAGCTGGAGGGGCTCTCCCTGGAC
CCAGGCTGGGGCCCCTTGGGGCTTCTTTCTTTCTAGAGGGGCAGCTCTTAGTAGGAGAGG
AGGTTTTTCCCCAAATGCCTTGGGGCTGCCACCTGGTACCCCAGCCTGCAGTCATACAGG
GTCCTGGCTGTCTGCACTGCCCACGTGGTGCTGCGGGCCTGCATGGAGCTGGCCTTCCTG
GTGGGGTCTACTCTCTGGGTGTGATATGCCATGGTTGCTTCACTGCCACTCCTCCCCTGT
CCCTCCAGTCCTGACTGCTTTGTGTCCAGAGCCATGAGGAAGAAAATCTTCCTGAACTTC
ATGTGCAGGTGGGGTTGGGCTGCTTCCTCCTGAACCCGATGGAGTTGTGCTACCTGGGCT
GGGTCTTCCCTTGCCAGGCACGCTCTGTGGCCTGCACCAGCTAGTGCTACTTCTGCTCCA
CTGTGATGAGGAAGGACCGTGCTCCAGGTGCCCTCC

>Hs-GJD3-Cx31.9-NM_152219
ATGGGGGAGTGGGCGTTCCTGGGCTCGCTGCTGGACGCCGTGCAGCTGCAGTCGCCGCTC
GTGGGCCGCCTCTGGCTGGTGGTCATGCTGATCTTCCGCATCCTGGTGCTGGCCACGGTG
GGCGGCGCCGTGTTCGAGGACGAGCAAGAGGAGTTCGTGTGCAACACGCTGCAGCCGGGC
TGTCGCCAGACCTGCTACGACCGCGCCTTCCCGGTCTCCCACTACCGCTTCTGGCTCTTC
CACATCCTGCTGCTCTCGGCGCCCCCGGTGCTGTTCGTCGTCTACTCCATGCACCGGGCA
GGCAAGGAGGCGGGCGGCGCTGAGGCGGCGGCGCAGTGCGCCCCCGGACTGCCCGAGGCC
CAGTGCGCGCCGTGCGCCCTGCGCGCCCGCCGCGCGCGCCGCTGCTACCTGCTGAGCGTG
GCGCTGCGCCTGCTGGCCGAGCTGACCTTCCTGGGCGGCCAGGCGCTGCTCTACGGCTTC
CGCGTGGCCCCGCACTTCGCGTGCGCCGGTCCGCCCTGCCCGCACACGGTCGACTGCTTC
GTGAGCCGGCCCACCGAGAAGACCGTCTTCGTGCTCTTCTATTTCGCGGTGGGGCTGCTG
TCGGCGCTGCTCAGCGTAGCCGAGCTGGGCCACCTGCTCTGGAAGGGCCGCCCGCGCGCC
GGGGAGCGTGACAACCGCTGCAACCGTGCACACGAAGAGGCGCAGAAGCTGCTCCCGCCG
CCGCCGCCGCCACCTCCGCCACCGGCCCTGCCCTCCCGGCGCCCCGGCCCCGAGCCGTGC
GCCCCGCCGGCCTATGCGCACCCGGCGCCGGCCAGCCTCCGCGAGTGCGGCAGCGGCCGC
GGCAAGGCGTCACCGGCCACCGGCCGCCGAGATCTGGCCATCTAG

>Hs-GJD4-Cx40.1-NM_153368 Splice site.
ATGGAAGGCGTGGACTTGCTAGGGTTTCTCATCATCACATTAAACTGCAACGTGACCATG
GTGGGAAAGCTCTGGTTCGTCCTCACGATGCTGCTGCGGATGCTGGTGATTGTCTTGGCG
GGGCGACCCGTCTACCAGGACGAGCAGGAGAGGTTTGTCTGCAACACGCTGCAGCCGGGA
TGCGCCAATGTTTGCTACGACGTCTTCTCCCCCGTGTCTCACCTGCGGTTCTGGCTGATC
CAGGGCGTGTGCGTCCTCCTCCCCTCCGCCGTCTTCAGCGTCTATGTCCTGCACCGAGGA
GCCACGCTCGCCGCGCTGGGCCCCCGCCGCTGCCCCGACCCCCGGGAGCCGGCCTCCGGG
CAGAGACGCTGCCCGCGGCCATTCGGGGAGCGCGGCGGCCTCCAGGTGCCCGACTTTTCG
GCCGGCTACATCATCCACCTCCTCCTCCGGACCCTGCTGGAGGCAGCCTTCGGGGCCTTG
CACTACTTTCTCTTTGGATTCCTGGCCCCGAAGAAGTTCCCTTGCACGCGCCCTCCGTGC
ACGGGCGTGGTGGACTGCTACGTGTCGCGGCCCACAGAGAAGTCCCTGCTGATGCTGTTC
CTCTGGGCGGTCAGCGCGCTGTCTTTTCTGCTGGGCCTCGCCGACCTGGTCTGCAGCCTG
CGGCGGCGGATGCGCAGGAGGCCGGGACCCCCCACAAGCCCCTCCATCCGGAAGCAGAGC
GGAGCCTCAGGCCACGCGGAGGGACGCCGGACTGACGAGGAGGGTGGGCGGGAGGAAGAG
GGGGCACCGGCGCCCCCGGGTGCACGCGCCGGAGGGGAGGGGGCTGGCAGCCCCAGGCGT
ACATCCAGGGTGTCAGGGCACACGAAGATTCCGGATGAGGATGAGAGTGAGGTGACATCC
TCCGCCAGCGAAAAGCTGGGCAGACAGCCCCGGGGCAGGCCCCACCGAGAGGCCGCCCAG
GACCCCAGGGGCTCAGGATCCGAGGAGCAGCCCTCAGCAGCCCCCAGCCGCCTGGCCGCG
CCCCCTTCCTGCAGCAGCCTGCAGCCCCCTGACCCGCCTGCCAGCTCCAGTGGTGCTCCC
CACCTGAGAGCCAGGAAGTCTGAGTGGGTGTGA

>Hs-GJE1-Cx23-NM_001358410 Splice sites
ATGTCTCTAAATTACATCAAAAACTTCTATGAAGGATGTGTTAAACCTCCAACTGTGATT
GGTCAATTCCACACCCTTTTCTTTGGATCGATCCGAATATTCTTCCTCGGGGTGCTAGGC
TTTGCAGTTTATGGGAATGAGGCCTTGCACTTCATTTGCGATCCAGACAAAAGAGAAGTA
AACCTCTTCTGTTACAATCAGTTCAGGCCAATCACTCCACAAGTAAGTTTTTCTGCATTA
CAACTAGTTATTGTCCTGGTTCCTGGAGCTCTTTTCCACCTTTATGCTGCATGTAAAAGC
ATCAATCAAGAATGCATTCTTCAAAAGCCTATCTACACTATAATTTATATACTCTCTGTT
TTATTAAGAATTAGTCTAGCGGCAATAGCATTCTGGCTTCAGATTTACCTCTTTGGTTTC
CAAGTAAAATCTCTTTACCTGTGTGATGCTAGATCTCTTGGGGAAAACATGATTATAAGA
TGCATGGTTCCAGAACACTTTGAAAAAACCATTTTTCTCATTGCAATAAATACATTTACA
ACAATTACAATTTTATTATTTGTTGCTGAGATTTTTGAGATCATATTTAGAAGATTATAC
TTTCCATTCAGACAATGA

Suppl. Fig. 2. Mouse (*Mus musculus*) connexins.

The chronology of the sequences are according to the Greek nomenclature. Both the Greek nomenclature and the size nomenclature are indicated, and the GenBank accession number is given for each entry.

Yellow: Conserved domains as defined by Cruciani and Mikalsen (2007)
Green: Conserved cysteine codons (cysteine signature)
Grey: 15 nt added at the ends of the conserved domains
Turquoise: Splice site.
Other colors are explained where necessary.

```
>Mm-gja1-NM_010288
ATGGGTGACTGGAGCGCCTTGGGGAAGCTGCTGGACAAGGTCCAAGCCTACTCCACGGCC
GGAGGGAAGGTGTGGCTGTCGGTGCTCTTCATTTTCAGAATCCTGCTCCTGGGGACAGCG
GTTGAGTCAGCTTGGGGTGATGAACAGTCTGCCTTTCGCTGTAACACTCAACAACCCGGT
TGTGAAAATGTCTGCTATGACAAGTCCTTCCCCATCTCTCACGTGCGCTTCTGGGTCCTT
CAGATCATATTCGTGTCTGTGCCCACACTCCTGTACTTGGCTCACGTGTTCTATGTGATG
AGAAAGGAAGAGAAGCTGAACAAGAAAGAAGAGGAGCTCAAAGTGGCGCAGACCGACGGG
GTCAACGTGGAGATGCACCTGAAGCAGATTGAAATCAAGAAGTTCAAGTATGGGATTGAA
GAACACGGCAAGGTGAAGATGAGAGGTGGCCTGCTGAGAACCTACATCATCAGCATCCTC
TTCAAGTCTGTCTTCGAGGTGGCCTTCCTGCTGATCCAGTGGTACATCTATGGGTTCAGC
CTGAGTGCGGTCTACACCTGCAAGAGAGATCCCTGCCCCCACCAGGTGGACTGCTTCCTC
TCACGTCCCACGGAGAAAACCATCTTCATCATCTTCATGCTGGTGGTGTCCTTGGTGTCT
CTCGCTCTGAATATCATTGAGCTCTTCTATGTCTTCTTCAAGGGCGTTAAGGATCGCGTG
AAGGGAAGAAGCGATCCTTACCACGCCACCACCGGCCCACTGAGCCCATCCAAAGACTGC
GGATCTCCAAAATATGCTTACTTCAATGGCTGCTCCTCACCAACGGCCCCACTCTCACCT
ATGTCTCCTCCTGGGTACAAGCTGGTCACTGGTGACAGAAACAATTCCTCCTGCCGCAAT
TACAACAAGCAAGCCAGCGAGCAAAACTGGGCGAATTACAGCGCAGAGCAAAATCGAATG
GGGCAGGCCGGAAGCACCATCTCCAACTCCCACGCCCAGCCGTTTGATTTCCCTGACGAC
AGCCAAAATGCCAAAAAAGTTGCTGCTGGACACGAACTCCAGCCCTTAGCTATCGTGGAT
CAGCGACCTTCCAGCAGAGCCAGCAGCCGCGCCAGCAGCAGACCTCGGCCTGATGACCTG
GAGATTTAA

>Mm-gja3-NM_016975
ATGGGCGACTGGAGCTTCCTGGGGCGGCTGCTGGAGAACGCACAGGAGCACTCTACAGTC
ATCGGCAAAGTGTGGCTGACCGTGCTGTTCATCTTCCGCATTCTGGTGTTAGGGGCGGCA
GCCGAGGAGGTGTGGGGCGACGAGCAATCGGACTTCACCTGCAACACACAGCAGCCAGGC
TGTGAGAACGTCTGCTACGACCGCGCTTTCCCCATTTCGCACATCCGCTTCTGGGCGCTG
CAAATCATCTTCGTGTCTACGCCCACCCTCATCTATCTGGGCCACGTGCTACACATCGTG
CGCATGGAGGAGAAGAAGAAAGAGCGGGAGGAAGAGCTGCTGAGGAGAGACAACCCTCAG
CACGGCCGTGGTCGCGAGCCAATGCGTACAGGGAGCCCGCGGGACCCTCCACTACGCGAT
GACCGTGGCAAGGTGCGCATCGCAGGTGCGCTGCTGCGGACCTACGTCTTCAACATCATC
TTCAAGACACTCTTCGAAGTGGGGTTCATCGCGGGCCAGTACTTTCTATACGGCTTCCAG
CTGCAGCCACTTTACCGCTGCGACCGCTGGCCCTGCCCCAACACTGTGGACTGTTTCATC
TCCAGGCCCACAGAGAAGACCATCTTTGTCATCTTCATGCTGGCTGTGGCCTGTGCGTCA
CTGGTACTCAACATGCTGGAGATTTACCACCTGGGCTGGAAGAAGCTCAAGCAGGGAGTT
ACTAACCACTTCAACCCAGATGCCTCAGAAGCCAGGCACAAGCCCTTGGACCCCCTACCC
ACGGCCACCAGCTCTGGCCCGCCCAGCGTCTCCATCGGGTTCCCACCTTATTACACACAC
CCTGCCTGTCCCACAGTACAGGCAAAGGCCATAGGGTTTCCTGGGGCCCCACTATCACCA
GCAGACTTCACAGTGGTGACTCTAAACGATGCTCAAGGCAGAAACCACCCAGTCAAACAC
TGCAATGGCCACCACCTGACGACAGAGCAGAACTGGACCAGGCAAGTGGCAGAGCAGCAG
ACTCCAGCCAGCAAGCCCTCTTCAGCAGCATCCAGCCCTGATGGCCGCAAGGGGCTCATT
GACAGCAGTGGCAGCAGCTTACAGGAGAGTGCCTTGGTAGTGACGCCAGAGGAGGGGGAA
CAGGCTTTGGCCACCACAGTGGAGATGCACTCGCCACCGTTGGTCCTCCTGGACCCAGGA
AGGTCCAGCAAGTCCAGCAACGGACGTGCCAGACCAGGTGACTTGGCCATCTAG

>Mm-gja4-NM_008120
ATGGGCGACTGGGGCTTCCTGGAGAAGTTGCTAGACCAGGTCCAGGAACACTCGACCGTG
GTGGGCAAGATCTGGTTAACGGTGCTCTTCATCTTCCGCATCCTCATCCTGGGGCTGGCT
GGCGAGTCGGTGTGGGGCGACGAGCAGTCTGATTTTGAGTGTAACACAGCCCAGCCGGGC
TGCACCAACGTCTGCTATGACCAGGCCTTCCCCATCTCCCACATCCGATACTGGGTGCTG
CAGTTCCTCTTCGTCAGCACACCCACCCTGATCTACCTGGGCCACGTCATCTACCTGTCT
CGGCGGGAAGAGCGGTTGCGGCAGAAAGAGGGAGAGCTCCGGGCGCTGCCATCCAAGGAC
```

CTACATGTAGAGCGGGCACTGGCTGCCATCGAACATCAGATGGCCAAGATCTCGGTGGCA
GAGGACGGTCGTCTTCGGATTCGTGGGGCGCTCATGGGTACCTATGTGGTCAGCGTGCTG
TGTAAGAGTGTGCTGGAGGCAGGCTTCCTCTATGGCCAGTGGCGCCTCTATGGCTGGACC
ATGGAGCCGGTGTTTGTGTGCCAGCGTGCGCCCTGCCCCCACATCGTGGACTGCTATGTC
TCTCGACCCACTGAGAAGACTATCTTCATCATCTTCATGCTGGTGGTAGGAGTCATCTCC
CTGGTGCTCAACCTGCTGGAGCTGGTTCACCTGCTGTGTCGGTGTGTCAGCCGGGAGATA
AAGGCACGAAGGGACCACGACGCCCGCCCGGCCCAGGGCAGTGCCTCAGACCCTTACCCT
GAACAGGTTTTCTTCTACCTCCCCATGGGCGAGGGACCCTCTTCCCCACCGTGTCCCACC
TACAACGGGCTCTCATCCACTGAGCAGAACTGGGCCAACTTGACCACAGAGGAGAGACTG
ACCTCTTCCAGACCTCCCCCATTTGTAAACACAGCTCCCCAGGGTGGCCGAAAGTCCCCT
AGCCGCCCCAACAGCTCTGCATCCAAGAAGCAGTATGTGTAG

>Mm-gja5-NM_001271628
ATGGGTGACTGGAGCTTCCTGGGGGAGTTCCTGGAGGAGGTCCACAAGCACTCCACAGTC
ATCGGCAAGGTCTGGCTCACTGTCCTGTTCATTTTCCGCATGCTGGTCCTGGGCACCGCT
GCTGAGTCCTCCTGGGGAGATGAGCAGGCCGACTTCCGGTGCGATACCATTCAGCCTGGT
TGCCAAAATGTCTGCTATGACCAAGCCTTCCCCATCTCCCACATTCGTTATTGGGTACTG
CAGATCATCTTTGTGTCCACGCCTTCTCTAGTGTACATGGGCCATGCCATGCACACTGTG
CGCATGCAGGAAAAGCAGAAATTGCGGGATGCTGAGAAAGCTAAAGAGGCCCACCGCACT
GGTGCCTATGAGTACCCAGTAGCCGAAAAGGCCGAGCTGTCCTGCTGGAAAGAAGTAGAT
GGGAAGATTGTCCTCCAGGGCACCCTACTCAACACCTATGTCTGCACCATTCTGATCCGC
ACCACCATGGAGGTGGCCTTCATCGTAGGCCAGTACCTCCTCTATGGGATCTTCCTGGAT
ACCCTGCATGTCTGCCGCAGGAGTCCCTGTCCCCACCCAGTCAACTGTTATGTTTCGAGG
CCCACGGAGAAGAATGTCTTCATTGTCTTTATGATGGCTGTGGCTGGACTGTCTCTGTTT
CTCAGCCTGGCTGAACTCTACCACCTGGGCTGGAAGAAGATCCGACAGCGCTTTGGCAAG
TCACGGCAGGGTGTGGACAAGCACCAGCTGCCTGGCCCTCCCACCAGCCTCGTCCAGAGC
CTCACTCCTCCCCCTGACTTCAATCAGTGCCTAAAGAACAGCTCCGGAGAGAAATTCTTC
AGCGACTTCAGTAATAACATGGGCTCCCGGAAGAATCCAGACGCTCTGGCCACTGGGGAA
GTGCCAAACCAGGAGCAGATTCCAGGGGAAGGCTTCATCCACATGCACTATAGCCAGAAG
CCAGAGTACGCCAGTGGAGCCTCTGCGGGCCACCGCCTTCCTCAGGGCTACCATAGTGAC
AAACGGCGCCTTAGTAAGGCCAGCAGCAAAGCAAGGTCAGATGACCTGTCAGTGTGA

>Mm-gja6-NM_001001496 (corresponds to human gja6p/gja1pX/cx43pX)
ATGAGTGATTGGAGTGCCTTACACCAGCTCCTAGAAAAGGTTCAACCCTACTCCACAGCT
GGAGGAAAGGTATGGATCAAGGTTCTTTTCATTTTCCGCATCCTGCTCCTGGGCACTGCT
ATCGAGTCGGCTTGGAGTGACGAGCAGTTTGAGTTCCATTGCAACACTCAGCAGCCTGGT
TGTGAAAATGTCTGCTATGACCATGCCTTCCCAATCTCTCACGTGCGCCTCTGGGTCCTC
CAGGTCATTTTCGTATCTGTGCCTATTCTCTTATACCTGGCACATGTGTACTATGTGGTT
CGACAGAATAAGAAGTTGAACAAGCAAGAGGAAGAACTGGAAGCTGCTCATTTTAATGAG
GCCAGCGTGGAAAGGCACTTGGAGACAATTGCAGGAGAGCAGTTCAAGTGTGGCAGTGAA
GAACAGAGTAAGGTGAAAATGAGAGGCAGATTGCTGCTAACCTACATGGCCAGCATCTTC
TTCAAGTCTGTCTTCGAGATGGCCTTCCTCCTGATCCAGTGGTACATTTATGGATTTACT
CTGAGTGCCCTTTACATCTGTGAGCAGTCTCCTTGCCCACGTCGGGTGGACTGCTTCCTC
TCTCGCCCCACCGAGAAAACCATCTTCATCCTCTTCATGTTTGTGGTGTCAGTGGTGTCT
TTTGTCTTGGATATCATTGAGCTGTTCTATGTCTTATTTAAGGCTATTAAGAATCGTATG
AGAAAAGCGGAGGATGAGGTTTACTGTGATGAGCTACCATGCCCTTCCCATGTCTCTTCA
TCAACTGTTCTCACCACCATAGATTCTAGTGAGCAGGCGGTTCCAGTGGAACTTTCTTCA
GTTTGTATTTAA

>Mm-gja8-NM_008123
ATGGGCGACTGGAGTTTCCTGGGAAACATCTTGGAAGAGGTGAATGAGCACTCCACTGTC
ATCGGCAGAGTCTGGCTCACAGTGCTCTTCATCTTCCGCATCCTCATCCTCGGGACAGCA
GCGGAGTTTGTGTGGGGCGATGAGCAATCTGATTTTGTATGCAACACCCAGCAGCCAGGC
TGTGAGAATGTCTGCTACGATGAGGCCTTTCCCATCTCACACATCCGCCTCTGGGTGCTG
CAGATCATCTTCGTCTCCACTCCATCGCTGATGTACGTGGGGCACGCGGTACACCACGTT
CGCATGGAGGAGAAGCGAAAGGACCGTGAAGCTGAGGAGCTCTGTCAGCAGTCGCGCAGC
AACGGGGGTGAGAGGGTACCAATCGCCCCAGACCAGGCCAGCATCCGGAAGAGCAGCAGC
AGTAGCAAAGGCACCAAGAAGTTCCGGCTGGAGGGCACACTGCTAAGGACCTATGTCTGC
CACATCATCTTCAAGACCCTCTTTGAGGTGGGCTTCATCGTGGGCCATTACTTCCTGTAT
GGTTTCCGCATCCTGCCCCTCTATCGCTGCAGCCGGTGGCCCTGCCCCAATGTGGTAGAC
TGCTTTGTATCCCGGCCTACTGAGAAGACCATCTTCATCCTCTTCATGTTATCAGTCGCT
TTTGTGTCACTCTTCCTCAACATCATGGAGATGAGCCACCTGGGCATGAAAGGAATCCGG
TCTGCCTTCAAGAGGCCTGTAGAGCAACCACTGGGGGAGATTGCTGAGAAGTCCCTCCAC
TCCATTGCAGTTTCCTCCATCCAGAAAGCCAAGGGCTACCAGCTTCTAGAAGAAGAGAAG
ATCGTATCACACTATTTCCCTTTGACAGAGGTTGGAATGGTGGAGACCAGCCCTCTTTCG
GCCAAGCCTTTTAGTCAGTTTGAGGAGAAGATCGGCACAGGACCCCTGGCAGATATGTCA
CGGAGTTACCAAGAAACCCTGCCTTCTTATGCTCAGGTGGGGGTCCAGGAAGTGGAGCGG

GAAGAGCCGCCTATAGAAGAGGCTGTGGAACCGGAAGTGGGAGAGAAGAAGCAAGAAGCA
GAGAAGGTGGCCCCAGAAGGGCAGGAGACAGTTGCAGTGCCAGACAGGGAGAGAGTAGAG
ACCCCTGGAGTGGGGAAGGAGGATGAGAAAGAAGAGCTGCAAGCTGAAAAGGTAACCAAG
CAAGGGCTGTCTGCTGAGAAGGCACCCTCACTCTGTCCGGAGCTGACAACCGATGACAAT
CGGCCCTTGAGCAGGCTGAGTAAAGCCAGCAGCAGGGCCAGGTCAGATGATCTCACCATA
TGA

>Mm-gja10-NM_010289
ATGGGAGATTGGAATTTACTGGGTGGCATCCTAGAGGAAGTCCACTCCCACTCCACTATA
GTGGGGAAGATCTGGCTGACCATCCTCTTCATCTTCCGAATGCTGGTACTTGGTGTCGCT
GCTGAGGACGTCTGGGATGATGAGCAGTCCGCCTTTGCCTGCAACACCCAGCAGCCCGGT
TGCAACAATATCTGTTACGATGATGCTTTCCCCATCTCTTTGATCAGATTCTGGGTTTTG
CAGATCATCTTTGTGTCTTCCCCTTCTTTGGTGTATATGGGCCATGCCCTTTATAGACTC
AGGGACTTTGAGAAGCAGAGGCAGAAGAAGAAGTTATACCTTAGAGCCCAGATGGAGAAT
CCAGAGCTCGACCTGGAGGAGCAACAAAGGGTAGATAAAGAGCTGAGGAGACTCGAGGAG
CAGAAGAGGATTCATAAAGTCCCTCTGAAAGGATGTCTGCTGCGCACCTATGTCTTACAC
ATCCTGACCAGATCAGTGCTAGAAGTAGGGTTCATGATAGGCCAATATATTCTCTATGGG
TTTCAAATGCACCCCATTTACAAGTGCACCCAAGCCCCCTGCCCCAATTCAGTGGACTGC
TTTGTTTCCAGGCCCACAGAGAAGACCATTTTCATGCTTTTCATGCACAGCATTGCAGCC
ATCTCCTTGTTACTCAATATCCTGGAAATATTTCATCTCGGCATCAGGAAAATCATGAGG
GCACTCGATGGCAAATCCAGCAGTGGGAACACTGAGAACGAAACAGGCCCTCCATTCCAT
TCAACAAACTACTCAGGGACCCAGCAGTGTATGATCTGTTCTTCTTTACCTGAAAGAATC
TCACTACTTCAAGCCAACAATAAACAGCAAGTCATCCGAGTCAATATACCACGGTCTAAA
AGCATGTGGCAAATTCCACACCCCAGGCAACTTGAGGTAGATGTATCCTGTGGCAAAAGA
GACTGGGCTGAGAAAATTGAGAGCTGTGCACAGCTCCACGTCCACAGCCCCTGCCCACAT
GACCGCAGTGCCAGAATTCAGCACCCTGGACAGCAACCGTGCCATTCTGTCTTTGGCCCC
AAGAATGCAATGTCTCAGTCTTGGTTCGGTACAATGACGGCTTCTCAACACCGTCCATCA
TCTGCGTTAGAAACCTGGGAGCGATCCCAGGGCCCAGAAGCTTCAGGGAGATCTCTCACA
GATCGCCAGAGTCACTTCCAAGGCAGTGACGGCAGTGCAAGAGAGAGTGGGGTTTGGACA
GACAGATTAGGCCCAGGAAGTCGCAAGGCCAGCTTTCTATCGAGGCTGATGTCAGAAAAG
GGACAACGGCATAGTGACTCAGGAAGCTCACGGTCTCTGAATAGTTCCTGCTTGGATTTT
TCACACGGAGAAAATAGCCCATCACCTCTGCCGTCTGCCACTGGGCACAGAGCATCGATG
GTAAGTAAAAGCAGCCATGTTGATTCACCTCCTCACTCTTCTTTCATCATACATGAGACA
TATGTATATGTGTATTAA

>Mm-gjb1-NM_008124
ATGAACTGGACAGGTCTATACACCTTGCTCAGTGGCGTGAATCGGCACTCTACAGCCATT
GGCCGAGTATGGCTGTCTGTCATCTTCATCTTCAGAATCATGGTGCTGGTGGTGGCTGCT
GAGAGCGTGTGGGGGGATGAGAAGTCCTCTTTCATCTGTAACACCCTCCAGCCGGGCTGC
AACAGCGTCTGCTATGACCATTTTTTCCCCATCTCCCACGTGCGCCTATGGTCCCTGCAG
CTTATCTTGGTTTCCACCCCAGCTCTCCTCGTGGCAATGCACGTAGCTCACCAACAGCAC
ATAGAAAAGAAAATGCTACGGCTTGAGGGGCATGGGGACCCCCTTCACCTGGAAGAGGTA
AAGAGACACAAGGTGCACATCTCAGGGACACTGTGGTGGACCTATGTCATCAGTGTGGTG
TTCCGGCTGCTGTTCGAGGCTGTCTTCATGTATGTCTTCTATCTGCTCTACCCCGGCTAT
GCCATGGTGCGGCTGGTCAAGTGTGAAGCCTTCCCCTGCCCCAACACAGTGGACTGCTTC
GTGTCCCGCCCCACCGAGAAAACCGTCTTCACTGTCTTTATGCTCGCAGCCTCCGGCATC
TGCATTATCCTCAACGTGGCGGAGGTGGTGTACCTCATCATCCGGGCCTGTGCCCGCCGT
GCTCAGCGCCGCTCCAATCCGCCCTCCCGCAAGGGCTCGGGCTTCGGCCACCGCCTCTCA
CCTGAATACAAGCAGAATGAGATCAACAAGCTGCTGAGCGAGCAGGATGGCTCTCTGAAA
GACATACTGCGCCGCAGCCCTGGCACAGGGGCCGGGCTCGCTGAAAAGAGCGACCGATGC
TCAGCCTGCTGA

>Mm-gjb2-NM_008125
ATGGATTGGGGCACACTCCAGAGCATCCTCGGGGGTGTCAACAAACACTCCACCAGCATT
GGAAAGATCTGGCTCACGGTCCTCTTCATCTTCCGCATCATGATCCTCGTGGTGGCTGCA
AAGGAGGTGTGGGGAGATGAGCAAGCCGATTTTGTCTGCAACACGCTCCAGCCTGGCTGC
AAGAATGTATGCTACGACCACCACTTCCCCATCTCTCACATCCGGCTCTGGGCTCTGCAG
CTGATCATGGTGTCCACGCCAGCCCTCCTGGTAGCTATGCATGTGGCCTACCGGAGACAT
GAAAAGAAACGGAAGTTCATGAAGGGAGAGATAAAGAACGAGTTTAAGGACATCGAAGAG
ATCAAAACCCAGAAGGTCCGTATCGAAGGGTCCCTGTGGTGGACCTACACCACCAGCATC
TTCTTCCGGGTCATCTTTGAAGCCGTCTTCATGTACGTCTTTTACATCATGTACAATGGC
TTCTTCATGCAACGTCTGGTGAAATGCAACGCTTGGCCCTGCCCCAATACAGTGGACTGC
TTCATTTCCAGGCCCACAGAAAAGACTGTCTTCACCGTGTTTATGATTTCTGTGTCTGGA
ATTTGCATTCTGCTAAATATCACAGAGCTGTGCTATTTGTTCGTTAGGTATTGCTCAGGA
AAGTCCAAAAGACCAGTCTAA

>Mm-gjb3-NM_001160012
ATGGACTGGAAGAAGCTCCAAGACCTATTGAGTGGCGTGAACCAGTACTCCACGGCATTT

GGGCGCATCTGGCTGTCAGTAGTGTTCGTCTTCCGGGTGCTGGTGTATGTGGTGGCTGCC
GAGCGTGTGTGGGGTGACGAGCAAAAAGACTTTGACTGTAACACCAGGCAACCCGGCTGT
ACCAACGTGTGCTATGACAACTTCTTCCCCATCTCCAACATCCGACTCTGGGCCCTGCAG
CTCATCTTCGTCACGTGTCCGTCTATGCTGGTCATCCTGCATGTAGCCTACCGCGAGGAG
CGGGAACGGAAGCATCGCCAGAAGCACGGGGAGCAATGCGCCAAACTGTACAGCCACCCG
GGCAAGAAGCATGGCGGCCTGTGGTGGACCTACTTGTTTAGCCTCATCTTCAAGCTCATC
ATCGAATTGGTCTTCCTGTACGTTCTCCACACGCTCTGGCATGGCTTCACCATGCCGCGT
CTGGTACAGTGCGCCAGCATAGTACCCTGCCCCAACACCGTGGATTGCTACATCGCTCGG
CCCACGGAGAAGAAGGTCTTTACCTACTTCATGGTAGGCGCTTCTGCCGTCTGCATTATT
CTCACCATCTGTGAGATCTGCTACCTCATCTTCCACAGGATCATGCGAGGCATAAGCAAG
GGCAAGTCCACAAAGAGCATCAGCTCCCCGAAGTCCTCCAGCCGGGCCTCCACCTGTCGC
TGTCACCACAAGCTGCTGGAGAGTGGCGATCCGGAAGCAGACCCAGCCAGTGAAAAGCTG
CAGGCTTCAGCGCCCAGCCTGACCCCCATTTGA

>Mm-gjb4-NM_008127
ATGAACTGGGGATTTCTCCAGGGAATCCTGAGTGGTGTGAACAAGTACTCCACGGCACTG
GGCCGCATCTGGCTGTCTGTGGTCTTCATCTTCCGGGTGCTGGTGTATGTGGTGGCGGCA
GAGGAGGTGTGGGACGACGATCAAAAGGATTTCATCTGCAATACCAAGCAGCCAGGCTGC
CCCAACGTCTGCTATGATGAGTTCTTCCCCGTGTCCCACGTGCGCCTCTGGGCCCTGCAG
CTCATCCTGGTCACCTGTCCTTCCCTGTTAGTGGTCATGCATGTGGCCTATCGTGAAGAG
CGAGAAAGGAAACATCGCCTCAAACATGGGCCCAATGCCCCAGCCCTGTACAGCAACCTG
AGCAAGAAGAGGGGTGGCCTGTGGTGGACATACCTGCTGAGTCTCATCTTCAAGGCTGCT
GTGGACTCTGGCTTTCTCTACATCTTCCATTGCATTTACAAGGACTATGACATGCCCCGA
GTGGTAGCTTGCTCTGTGACTCCCTGCCCCCACACTGTGGACTGTTACATCGCCCGACCC
ACAGAGAAGAAGGTCTTCACCTACTTCATGGTAGTCACGGCAGCCATTTGTATTCTACTC
AACCTCAGTGAGGTCGTCTACCTGGTGGGCAAGAGATGCATGGAGGTCTTCCGTCCCCGG
CGCCGGAAAGCTTCCAGGAGGCACCAACTGCCAGATACGTGCCCACCGTATGTGATCTCC
AAAGGAGGTCACCCTCAAGATGAGAGCGTGATCCTAACAAAGGCCGGGATGGCCACGGTG
GATGCAGGTGTGTATCCATGA

>Mm-gjb5-NM_010291
ATGAACTGGAGTGTTTTTGAGGGACTCCTGAGCGGAGTCAACAAGTACTCCACAGCCTTT
GGTCGCATCTGGCTGTCTCTGGTCTTCGTCTTCCGTGTGCTGGTGTACCTGGTGACAGCT
GAGCGCGTGTGGGGAGACGACCAGAAGGATTTTGACTGCAACACCAGGCAACCGGGCTGT
ACCAATGTCTGCTACGATGAGTTCTTCCCCGTGTCTCACGTGCGCCTCTGGGCTCTGCAG
CTCATCCTGGTCACCTGCCCCTCTTTGCTTGTGGTCATGCATGTGGCCTATCGAAAGGCT
CGAGAGAAGAAGTACCAGGAAAAGATTGGTGAAGGTTACCTTTACCCGAATCCCGGCAAG
AAGCGGGGTGGACTCTGGTGGACATACGTCTTCAGCCTCTCGTTCAAGGCCACCATAGAC
ATTATCTTCCTCTACCTCTTCCACGCATTCTATCCCAGATATACCCTCCCTTCTATGGTC
AAGTGCCATGCGGAGCCGTGTCCCAACACAGTGGACTGCTTCATTGCCAAGCCCTCCGAG
AAAAACATCTTCATTGTCTTCATGGTGGTCACGGCCGTCATCTGCATCCTGCTTAACCTT
GTGGAGCTGATCTACCTAGTGATTAAGCGGTGTTCTGAGTGTGCGCAGCTGAGGAGACCA
CCCACTGCACATGCAAAGAATGACCCAAACTGGGCCAACTCTCCTAGCAAAGAGAAGGAC
TTCCTCTCATGCGACCTCATCTTTCTGGGCTCGGACGCTCACCCGCCTCTGTTACCAGAC
CGCCCTCGAGCCCACGTGAAGAAAACCATTCTGTGAA

>Mm-gjb6-NM_001010937
ATGGACTGGGGGACCCTGCACACCGTCATCGGTGGCGTGAACAAGCACTCTACCAGCATA
GGGAAGGTGTGGATCACGGTCATCTTTATTTTCCGAGTCATGATCCTAGTGGTGGCTGCC
CAGGAAGTGTGGGGTGATGAGCAGGAGGACTTTGTCTGCAACACTCTGCAGCCAGGGTGC
AAGAACGTCTGCTATGACCATTTCTTCCCGGTGTCTCACATCCGGCTCTGGGCCCTGCAG
CTGATCTTTGTGTCTACCCCAGCCCTGTTGGTGGCCATGCACGTGGCCTACTACAGACAT
GAAACTGCCCGAAAGTTTATACGTGGGGAGAAGAGAAACGAGTTTAAAGACCTGGAGGAC
ATCAAACGGCAGAAGGTGCGCATTGAGGGCTCCCTGTGGTGGACGTACACCAGCAGCATT
TTCTTCCGCATCATCTTCGAAGCCGCCTTCATGTATGTGTTCTACTTCCTCTACAATGGG
TACCACCTACCCTGGGTACTGAAATGTGGCATTGACCCCTGCCCCAATCTCGTGGACTGC
TTCATTTCGAGGCCAACTGAGAAAACGGTGTTCACTGTTTTTATGATTTCCGCATCCGTG
ATTTGCATGCTGCTCAATGTGGCCGAGTTGTGTTACCTGCTGCTTAAATTGTGCTTTAGG
AGATCCAAAAGAACACAGGCGCAGAGAAACCACCCCAACCATGCCCTGAAAGAGAGCAAG
CAGAATGAAATGAATGAGCTGATCTCAGATAGTGGCCAGAATGCAATCACAAGTTTCCCA
AGTTAA

>Mm-gjc1-NM_008122
ATGAGTTGGAGCTTCCTGACTCGCCTGCTAGAGGAGATCCACAACCATTCGACATTTGTA
GGGAAGATCTGGCTCACTGTGCTGATTGTCTTTCGAATTGTCCTAACTGCTGTAGGAGGA
GAGTCCATCTACTATGATGAACAAAGCAAATTTGTGTGCAACACAGAGCAGCCGGGCTGT

GAGAATGTCTGCTATGATGCCTTTGCCCCGCTCTCCCACGTGCGCTTCTGGGTATTCCAG
ATCATCCTGGTTGCAACTCCCTCTGTGATGTACCTGGGATATGCTATTCATAAGATTGCC
AAAATGGAGCATGGCGAGGCAGACAAGAAGGCAGCTCGGAGCAAACCCTATGCCATGCGT
TGGAAACAGCACCGGGCTCTGGAAGAAACGGAAGAGGACCATGAAGAGGATCCTATGATG
TATCCAGAGATGGAGTTAGAAAGCGAAAAAGAAAATAAAGAGCAGAGCCAACCAAAACCT
AAGCATGATGGCCGACGACGAATTCGAGAGGATGGGCTCATGAAAATCTATGTGTTGCAG
CTGCTGGCCAGGACTGTGTTTGAGGTGGGCTTTCTAATAGGGCAGTATTTCCTGTATGGC
TTCCAAGTCCACCCATTTTATGTGTGCAGCAGACTTCCTTGCCCTCATAAGATAGACTGC
TTTATTTCTAGACCCACTGAAAAGACCATCTTCCTTCTGATAATGTATGGTGTCACAGGC
CTCTGCCTATTGCTTAACATTTGGGAGATGCTTCACTTAGGGTTTGGGACAATTCGAGAC
TCACTAAACAGTAAAAGGAGGGAACTTGATGATCCGGGTGCTTATAATTATCCTTTCACT
TGGAACACACCCTCTGCTCCCCCTGGCTATAACATTGCTGTCAAACCAGATCAGATCCAG
TACACTGAGCTGTCCAATGCTAAGATTGCCTACAAGCAAAACAAAGCCAATATTGCCCAG
GAACAGCAGTACGGCAGCCACGAGGAACACCTCCCGGCTGATCTGGAGACTCTGCAGCGG
GAGATCAGAATGGCTCAGGAACGCTTGGACCTAGCAATCCAGGCCTACCATCACCAAAAC
AACCCCCATGGTCCTCGGGAAAAGAAGGCCAAAGTGGGGTCCAAATCTGGGTCCAACAAA
AGCAGTATTAGTAGCAAATCAGGGGATGGGAAGACCTCCGTCTGGATTTAAT

>Mm-gjc2-NM_080454
ATGAGCTGGAGCTTCCTGACGCGGCTGCTGGAGGAGATCCACAATCATTCCACCTTCGTG
GGCAAAGTTTGGCTCACTGTGCTGGTGGTCTTCCGCATTGTGCTGACAGCCGTCGGTGGT
GAGTCCATCTATTCAGATGAGCAATCCAAGTTCACCTGCAACACGCGGCAACCGGGTTGT
GACAACGTCTGCTATGACGCCTTTGCGCCCCTGTCTCATGTGCGCTTCTGGGTCTTCCAG
ATAGTGGTCATCTCCACACCTTCTGTCATGTACCTGGGCTATGCAGTCCACCGCTTGGCG
CGGGCCTCGGAACAGGAGCGCAGACGCGCTCTCCGACGTCGCCCTGGCACCCGGCGCTTG
CCCAGGGCGCAGCTGCCACCGCCGCCACCTGGCTGGCCGGACACCACCGATCTGGGAGAG
GCGGAGCCCATATTGGCTCTAGAGGAGGATGAGGACGAGGAGCCGGGGGCGCCCGAGGGC
CCCGGAGAAGACACGGAGGAGGAGCGAGCGGAGGATGTGGCTGCCAAAGGGGGCGGAGGT
GATGGCAAGACGGTGGTCACTCCTGGCCCGGCCGGGCAGCACGATGGGCGGCGGCGCATC
CAGAGGGAGGGCCTGATGCGTGTGTACGTGGCTCAGCTGGTGGTTAGGGCGGCCTTCGAG
GTGGCCTTTCTGGTGGGCCAGTACCTACTGTACGGCTTCGAGGTGCCACCCTTCTTTGCC
TGCAGCCGCCAGCCTTGCCCCCACGTAGTGGATTGCTTCGTGTCGCGGCCGACCGAGAAG
ACGGTCTTCTTGCTGGTCATGTACGTGGTTAGCTGTCTATGCTTGTTGCTCAACCTCTGT
GAGATGGCGCACCTGGGTCTCGGCAGTGCGCAGGATGCTGTGCGCGGCCGTCGGGGAGCC
TCAGCGGCGGGGCCTGGCCCCACGCCGCGCCCACCGCCCTGCGCTTTCCCGGCCGCGGCC
GCCGGCCTGGCTTGCCCTCCAGACTACAGCCTGGTGGTGCGTGCAGCTGAGCGCGCGCGA
GCGCACGGCCAGAACTTGGCGAACCTAGCGCTGCAGGCGTTGCGCGATGGGGCGGCGGTG
GCGGCGGTTTCCGCGGACCGCGACAGTCCGCCGTGCGCTGGGCTCAATGCAACCTCTCGG
GGGGCACCCAGGGTGGGCGGCCTAGCTTCCGGAACCGGCAGCGCCACGTCGGGGGGCACC
GTTGGGGAGCAGAGCCGGCCGGGAGCTCAGGAACAACTGGCCACTAAGCCCAGGGCTGGC
TCTGAAAAGGGCAGTACAGGCAGCAGAGACGGCAAGGCCACCGTGTGGATCTGA

>Mm-gjc3-NM_080450
ATGTGCGGCAGGTTCCTGAGACAGCTATTGGCTCAGGAGAGCCAGCACTCCACCCCTGTG
GGGCGCTTCCTTCTTCCCATGCTCATGGGATTCCGTCTCCTGATCTTGGTTTCCAGTGGA
CCTGGGGTTTTCGGCAATGATGAGAATGAATTCATATGTCATTTAGGGCAGCCAGGCTGC
AAGACCATTTGCTATGATGTCTTCCGCCCTCTCTCTCCATTGCGCTTCTGGGCCTTCCAA
GTCATTCTGATGGCTGTACCCAGTGCCATTTATGTGGCTTTCACTCTGTATCATGTGATT
GGATACTGGGAGGTACCAGGAAAGGAAAACAAGGAGCAAGAGACCCAGATTAGCAAAGGG
GATCATAGCAAGGATGTCTCAGGGGCTAAAAGCCTCAAGCTTCTCTGGGCCTATGTGGCA
CACCTTGGGGTACGGCTGGCCCTTGAGGGAGCAGCTCTAGGTGTTCAGTACAATCTGTAT
GGTTTCAAGATGTCCAGCACTTTTATATGTCGTGAGGATCCTTGTATTGGCAGCACAACC
TGTTTCCAGTCTCACCCCTCTGAGAAGACCATCTTCCTCAACATCATGTTTGGGATCAGC
GGGGCCTGTTTCTTATTTATTTTCTTGGAGCTTGCGCTTTTGGGTTTAGGGAGGTTTTGG
AGGATATACAAGCACAAACTTTCCTTCTTAAAGAAGTTGCCAACTTCAGAGAGCTCTGTA
AGATCCAAGGACACAACCGATGAATTGTCAGTGGTGGAGGCAAAAGAGCCATTTTGA

>Mm-gjd2-NM_010290 Splice site
ATGGGGGAATGGACCATCTTGGAGAGGCTGCTGGAAGCCGCGGTGCAGCAGCACTCCACT
ATGATTGGGAGGATCCTGTTGACTGTGGTGGTGATCTTCCGGATACTCATTGTGGCCATT
GTAGGGGAGACGGTGTACGATGATGAGCAGACCATGTTTGTGTGCAACACCCTACAGCCC
GGCTGTAACCAGGCCTGCTATGACCGCGCCTTTCCCATCTCCCATATACGTTACTGGGTC
TTCCAGATCATAATGGTGTGCACCCCCAGTCTCTGTTTTATCACCTATTCTGTGCACCAA
TCCGCCAAGCAGCGAGAACGCCGGTACTCTACTGTCTTCCTAGCCCTGGACAGAGACCCT
GCTGAGTCTATAGGGGGACCTGGAGGAACTGGGGGTGGGGGCAGCGGAGGGAGCAAACGA
GAAGATAAGAAGTTGCAAAATGCCATTGTCAATGGGGTGCTGCAGAACACAGAGACCACC
AGTAAGGAGACAGAACCAGATTGCTTAGAGGTTAAAGAGCTGACTCCACATCCATCTGGG
CTGCGCACAGCAGCAAGGTCCAAGCTCCGAAGACAGGAAGGTATCTCCCGCTTCTACATC

ATCCAAGTGGTGTTTCGAAATGCTCTGGAGATTGGGTTTCTGGTGGGCCAGTACTTTCTA
TATGGCTTCAGTGTTCCAGGGTTGTATGAGTGCAACCGTTACCCCTGCATCAAGGAGGTA
GAATGTTATGTGTCTAGACCTACCGAGAAGACAGTCTTTCTGGTGTTCATGTTTGCTGTG
AGCGGCATTTGTGTGGTGCTCAATCTGGCTGAACTTAACCATCTGGGATGGCGGAAGATC
AAACTGGCTGTCCGGGGAGCCCAGGCCAAGAGGAAGTCAGTCTATGAGATACGTAACAAA
GATCTGCCTCGAGTCAGTGTTCCCAATTTCGGCAGGACTCAGTCCAGTGACTCTGCCTAT
GTGTGA

>Mm-gjd3-NM_178596
ATGGGGGAGTGGGCGTTCCTAGGCTCCCTGCTGGACGCGGTGCAGCTACAGTCGCCGCTC
GTGGGTCGTCTCTGGCTGGTGATCATGCTGATCTTCCGCATCCTGGTGCTGGCCACGGTG
GGAGGTGCGGTGTTCGAGGACGAGCAGGAGGAGTTCGTGTGTAACACGTTGCAGCCCGGC
TGTCGCCAGACCTGCTACGATCGCGCCTTCCCGGTGTCCCACTACCGCTTCTGGCTCTTC
CACATCCTGCTGCTGTCGGCGCCGCCGGTGCTGTTCGTCATCTACTCCATGCACCAGGCC
AGCAAGGAGGCGGGTGGTGCGCAGCTGGCCCCGCCGTGCGCGCGCGGGCGTGCCGAGGCG
CCGTGCTCCCCGTGCGCCCTGCGCGCTCGCCGCGCGCGCCGCTGCTACCTGCTGAGCGTG
GCTCTGCGCCTGCTCGCCGAGCTGGCTTTCCTGGGCGGCCAGGCGCTGCTCTACGGCTTC
CGCGTGGACCCGCACTACGCGTGCGCCGGGCCACCTTGTCCGCACACGGTCGACTGTTTC
GTGAGCCGGCCCACCGAGAAGACCGTCTTCGTGGTCTTCTACTTCGCCGTCGGGCTGCTG
TCGGCGCTGCTCAGCGTGGCGGAGCTGGGTCACCTGCTCTGGAAGGGTCGCCAGCGCGCC
AAGCTGCTCCCGCCGCCGCCGCCGTCGCCCTCTTTGCCATCGCAGCGCGGGGACCCCGAC
CCTTTCGGCCCGCCAGCCTACGCGCACCGCTCACCGGCAGGCGACAGCGAGGGCGAAGGC
GGCAGCGGCCACAGCAAAGCGTCGCTGGCTACCGTGCGCCAGGACCTGGCCATCTAG

>Mm-gjd4-NM_153086 Splice site
ATGGAGAAGTTGAACTTGTTGGGATTCCTCATCATCACCTTAAACTGTAACGTGACCATC
ATGGGCATGATCTGGCTGATCGTGGAGGTCTTGCTGAGGATGCTAGTGGTGGTCTTGGCA
GGGTCACCTATCTATGAGGATGAACAAGAGAGGTTTATTTGCAACACACTGCAACCAGGA
TGTGCCAACGTTTGCTACGACCTCTTTTCCCCAGTGTCACCGCTGCGATTCTGGCTAGTG
CAGAGCCTGGCCTTGCTTCTGCCTTCGGTGGTCTTTGGCACTTACACCCTACACCGCGGT
GCGAAGCTGGCTGCAGTGGGGGGAGCCTGCAGGCCCCAGGTGCCCGACCTGTCTACTGCC
TACCTGGTGCACCTACTGCTGCGCATGCTGCTGGAGGCCGGGCTGGCCTTCCTGCACTAC
TTTCTCTTTGGCTTTTCTGTGCCCGCCCGCGTGTCTTGCTCGCATGTACCCTGCTCAGGG
GCTGTGGACTGCTACGTGTCGCGGCCCACGGAGAAGTCACTCCTGATACTATTCTTTTGG
GCAGTGAGTGCGCTATCCTTCCTGCTCAGCTTGGCTGACCTGCTTTGGATCCTGCCGAGG
AGAAAGACACTGAGGACCACGCAGTGGGTGAATGGAGAGGCTAGACCAGTCTGTGAAGTA
CCTGCACCTCCCCCTTGCCTCTTACAAAACCCCCAGGGCTATCTTAGCCAAGGTCAGGTG
GACCAAGAGGACAGACAGGAGGAACAAGTTGTGCCTGAGTTCCCCTGCATGTGGACAGCA
GGGCAGAGTGACAACAGCAATGTTGGTCAGGCCTGTGTGTCGGGGCTGCTGGAACATTCA
GACCAAGATGCTAGTGAGGCCACTTCCTCAGCTGGTGACAGGCTAACAGTGGCTCACACA
GCACATGAGCTCAGATTCCACAGAGAGACTTCACTGGACCTGGGGGGCAAAAACACCCAG
GCAGATGAACTCTCCTTGGCTACCCAGAGCCACCTGGCCAGACACAGTTCAGCCAGCAAG
CCTCAAGCTCCATGCCGGCTGACCACCTCAGGCAGTGCTCCCCATTTGAGAACCAAAAAA
TCTGAGTGGGTGTGA

>Mm-gje1-NM_029722 Splice sites
ATGTCTCTAAATTACATCAAGAACTTCTATGAAGGATGTGTTAAGCCTCCAACTGTGATC
GGCCAGTTCCACACTCTCTTCTTCGGCTCAGTGCGGATGTTCTTCCTCGGAGTGCTGGGC
TTTGCTGTCTACGGGAATGAGGCGTTGCACTTCAGCTGTGACCCAGACAAGCGAGAGATA
AACCTGTTCTGTTACAATCAGTTCCGGCCAATAACTCCCCAAGTGTTCTGGGCATTGCAG
CTAGTGATTGTCCTGCTTCCTGGAGCTATTTTCCACCTGTATGCTGCATGCAAAAGCATC
AATCAAGACTGCATTCTTCAGAAGCCCGTGTACACTGTGATTTACGTCCTCTCGGTCTTG
TTAAGAATCAGCCTGGAGGTGTTCGCATTCTGGCTTCAGATTCACCTCTTCGGCTTCCAA
GTGAAGCCGATATACTTGTGTGATACTGAATCTCTTGGTAAAAAACCAAATATTCTAAAA
TGCATGGTTCCAGAGCACTTTGAAAAGACTATTTTCCTCATTGCAATGTACACATTTACT
GTGATCACGATGGTATTATGTGTTGCTGAGGTTTTTGAGATCATATTTAGAAGATCATGT
TTTCTCTTTAAACGATGA

Suppl. Fig. 3. Opossum (*Monodelphis domestica*) connexins.
Yellow: Conserved domains as defined by Cruciani and Mikalsen (2007)
Green: Conserved cysteine codons (cysteine signature)
Grey: 15 nt added at the ends of the conserved domains
Turquoise: Splice site.
Other colors are explained where necessary.

>Md-GJA1-XM_007484502
ATGGGGGATTGGAGTGCCTTAGGCAAACTCCTTGACAAAGTACAGGCTTATTCTACTGCT
GGAGGGAAGGTGTGGCTCTCCGTCCTCTTCATTTTCCGAATCTTGCTATTGGGAACCGCG
GTGGAATCAGCTTGGGGTGATGAACAGTCTGCCTTTCGATGTAACACTCAGCAGCCAGGT
TGTGAAAATGTATGCTATGACAAATCCTTCCCAATCTCTCACGTGCGATTCTGGGTCCTG
CAAATCATCTTTGTGTCTGTGCCAACCCTCCTGTACCTGGCACACGTGTTCTATGTGATG
CGTAAAGAAGAGAAGCTGAACAAGAAAGAAGAAGAGCTCAAAGTCGCCCAAACGGACGGT
GCCAATGTGGATATGCACTTGAAACAAATTGAAATCAAGAAATTCAAATATGGAATTGAA
GAACATGGCAAAGTGAAAATGCGTGGAGGGTTACTGCGTACCTACATCATCAGCATCCTT
TTTAAGTCTGTGTTCGAGGTGGCCTTCCTCCTGATTCAGTGGTACATCTATGGCTTCAGC
TTGAGCGCGGTCTATACTTGCAAGCGGGATCCCTGCCCTCATCAAGTGGACTGCTTCCTC
TCCCGTCCCACCGAGAAAACGATCTTCATTATCTTCATGCTGGTTGTGTCCTTGGTGTCT
CTTGCCTTAAATATCATTGAGCTCTTCTATGTGTTCTTCAAGGGTGTCAAGGATCGCGTG
AAGGGAAAAAGCGACCCTTACCACACTACAGCCGGCCCACTGAGTCCCAGCAAAGATTGC
GGTTCTCCTAAATATGCTTATTTCAATGGCTGTTCCTCCCCAACCGCCCCCTTGTCACCC
ATGTCTCCTCCAGGGTACAAGCTCGTGACCGGAGATCGAAATAATTCTTCTTGCCGTAAC
TACAACAAGCAAGCCAGTGAGCAAAACTGGGCCAACTATAGTGCTGAACAGAATAGAATG
GGGCAGGCTGGAAGCACCATCTCCAATTCCCATGCTCAGCCTTTTGATTTTCCAGATGAT
AACCAGAATTCAAAAAAACTAGCCGCCGGCCATGAGCTACAGCCACTTGCCATTGTGGAC
CAAAGGCCTTCCAGTAGAGCCAGCAGTAGGGCCAGCAGCCGACCTCGACCCGATGACTTG
GAGATCTAA

>Md-GJA3-XM_007495190
ATGGGTGACTGGAGCTTTCTGGGGAGATTATTAGAGAATGCGCAAGAACACTCTACCGTG
ATTGGCAAAGTTTGGCTGACTGTTCTGTTCATCTTCAGAATCCTGGTGCTAGGTGCCGCC
GCAGAAGAAGTCTGGGGAGATGAACAGTCCGATTTTACGTGTAACACTCAGCAACCAGGT
TGTGAGAATGTCTGCTATGACAAGGCTTTTCCTATTTCCCACATCCGCTTCTGGGTCCTG
CAGATCATTTTCGTCTCTACCCCCACCCTCATTTATCTGGGCCATGTGCTGCACATTGTA
CGCATGGAGGAAAAAAAGAAGGAAAAAGAAGATCTCCTTAAGAAAGACAACCTGCACCAG
GGGGTAGAGCTCAGTGGCCCCAACAGCCATCGAGAACCACTCAGCAAAAAGGAGAGCGCC
AGGATGGAAAAGAAAGAGAGACCCCCAATCCGAGATGATCGAGGCAAAGTCCGGATAGCT
GGTGCCCTGCTCCGCACCTACGTCTTTAACATCATCTTCAAGACTCTGTTTGAAGTGGGC
TTCATTGTGGGGCAATATTTCCTCTACGGGTTTGAGTTAAAGCCACTCTACCGATGTGAC
CGCTGGCCCTGCCCCAACACAGTGGATTGCTTTATCTCCAGACCCACAGAAAAGACCATA
TTCATTATTTTCATGTTAGTGGTGGCTTGTGTGTCTCTCTTGCTCAACATGCTGGAGATC
TATCACCTGGGCTGGAAAAAGCTTAAACAGGGTATGACCAACCATTACAATAGCCCAGAT
TCTCCAGAAGCCAAAGTCCTGCCCTCCAAATGTAGTAGCATTGGCCCACTTCTGCTCTCT
CCCCATTCTGCCCCTCCTGCCGTTGGATTCCCACCATACTATACTCAGTCTGCCTCTTCC
CTAGGACAGGCAACCACTACAGGTTATCCTGGGGCTCCTCCACAACCCACAGAATTCAGA
ATGGTAACCCTCCCTGAGGAGCGCAGTAAGGCAGCCCCAGCCAAATATTATGGTGGCAAC
CACCACCACATCTTAATGACTGAGCAGAACTGGGCCAACCAGGAGGCAGAGCAACAGACT
TTTGAAAGGAAGTCTTCCCCAAGCAATACCACCCCTGGCACTCCCACCACCCCAAGCAGT
GTTCGGCAGCTCCTCCAAGAGCGAGGAGACAGTGGTGGGGGGAGCAAAGTGCCCTTGCTG
GTGATTAATGGGAGTAGCAGCAGTTTAGGGGCCACTAAATCAGAGGTAACCTCTGAAGGG
GAGAAACAGCCAGGTACCACCACAGTTGAGATGCATGCACCGCCATTGCTTCTCGTTGAT
TCAAGACGGTTAAGCAAGGCTAGCAAGGCTAGCAGTGGCAGAGCTCGATCGGATGACTTA
GCCATCTAA

>Md-GJA4-XM_007492764
ATGGGTGACTGGGGCTTCCTAGAGAAACTGCTAGACCAAGTCCAGGAGCACTCCACAGTG
ATAGGCAAGATCTGGCTGACCGTACTGTTCATCTTCCGGATCCTCATCCTGGGGCTGGCC
GGTGAGTCTGTCTGGGGAGACGAGCAATCAGACTTCGAATGTAACACGGCCCAGCCGGGC
TGCACCAACGTGTGCTATGACCAGGCCTTCCCCATCTCCCACATTCGCTACTGGGTCCTG
CAGTTCCTCTTTGTCAGCACGCCCACCCTGGTCTACCTGGGCCACGTCATCTACCTGTCC
CGCCGAGAAGAGAAGCTGCGGCAGAAGGAGAGCGAGCTGCGGGCGCTTCAAGCCAAGGAC
CCACGAGTGGAGCAGGCGCTGGCCACGGTGGAGCGGCAGATGGCAAAGATCTCTGTGGCC
GAGGATGGGCATCTTCGCATCAGGGGAGCCCTGATGGGCACCTACGTGGCCAGCGTGGTC
TGTAAGAGCCTGCTGGAGGCTGGCTTCCTCTATGGACAGTGGCGCCTGTACGGCTGGATG

ATGGAGCCCGTGTACGTGTGCCGACGCTTCCCCTGCCCACACCTGGTGGACTGCTTTGTG
TCCCGCCCCACAGAAAAGACCATCTTCATCATCTTCATGCTGGTCGTGGGGATGATTTCC
CTAGTACTCAACCTCCTGGAGCTGGCACATCTTGGCTTTCGGTGTATGGGGCACAAGCTG
AGGGCCCGGCAGACCCGGGCTCGGCTGGGGCCTTACTCTGCTGCCCTGGGGGATGGGGCT
GGTATGGGTAGTTCTGGGGACCCCTATTCGGATCGGATGTTCTTCTACCTCCCCATGAAC
GAGACACCTACGTCGCCTCCCTGCCCCTCTTACAACAAGCTGTCTAGTGAACAGAACTGG
GCTAACCTGAATACAGAAGAGAGCCTGGCCACCCAGAAGCAGGGCATGCTCCCTGGGCCG
GGGCCCCTGCTGACTCATCCCGAGGGCCCCTACCTGGCTCAGCCCCCCCAGAACGGAGAC
AATGCCCTGAGTCGTCCTAGTAGCTCAGCTTCCAAGAAACAGTATGTGTAA

>Md-GJA5-XM_007485330
ATGGGTGACTGGAGCTTCCTCGGGGAGTTCCTGGAAGAGGTCCACAAGCATTCCACAGTG
ATTGGCAAGGTTTGGCTGACTGTCCTCTTCATCTTCCGTATGCTAGTGCTGGGCACAGCG
GCGGAGTCCTCGTGGGGGGACGAGCAAGCCGACTTCCAGTGTGACACGCTACAGCCTGGT
TGTGAGAACGTCTGCTATGACCAAGCCTTCCCCATCTCCCACATCCGATACTGGGTGCTG
CAGATCATCTTCGTCTCCACTCCATCCCTGGTGTACATGGGCCACGCGATGCACACCGTG
CGCATGGAGGAGAAGAGGAAGCTGAAGGAGGCTGAGAGGGCCAAGGATTCCAAGGGTGCA
GACACCTATGAGTATCAGGCAGAGAAGGCTGAGCTTTCCTGCCGGGAAGAGCTGAGTGGA
AGGATTATCCTTCAAGGCACCCTTCTCAACACCTATGTCTGCAGCATTCTCATTCGCACG
GCCATGGAGGTGGCTTTCATCGTGGGGCAGTACCTCCTCTACGGGGTCTTCCTGGAGACC
CTATACATCTGTCGCCGAAAACCCTGCCCTCACCCGGTCAACTGCTATGTGTCTCGGCCC
ACTGAGAAGAATGTTTTTATCGTATTCATGCTGGCTGTGGGGGGTCTGTCCCTCTGCCTC
AGTCTGGCTGAACTCTACCATCTGGGCTGGAAGAAAGCCAAGCGGCACTTCAACCAGAAC
TGCCCAGGCAGGGTGGAGGCCAGCCTGCCCACAGCAGGGGTGGCCCAGAACTGCACGCCA
CCCCCAGACTTCAATGAGTGTGTAGAGGGCAGCCCCAATCAGAAGTTCTACAGCAGTCAT
TTCAGCAATAATATTGCCTCCCGGCAGAATACAGACAACTTGGCCACAGAGCAGGTCCAA
GGCCAGGAGGAGGCTGTAGGGAGGGCCTTTTTGCACATGCAGTATGCAGAGGGGCCCGAG
GTGGCTAACGGGATGTCCAATGCACATCGGTTCCCCCATAGTTACCATGCTGACAAGCGC
CGCCTGAGCAAGGCTAGCAGCAAAGCCAGGTCAGATGACTTGTCGGTGTGA

>Md-GJA8-XM_001363421
ATGGGTGATTGGAGTTTCCTGGGGAATATCTTGGAGGAGGTAAATGAGCACTCCACGGTC
ATCGGCAGGGTCTGGCTCACCGTCCTCTTCATCTTCCGGATTCTGATCCTGGGCACAGCA
GCAGAATTTGTGTGGGGTGACGAGCAGTCGGACTTTGTATGTAACACCCAACAACCAGGT
TGTGAGAATGTCTGCTACGATGAGGCCTTCCCCATCTCCCACATCCGTCTCTGGGTCTTA
CAGATCATCTTCGTGTCCACTCCATCCCTGGTGTATGTGGGCCATGCTGTGCACCATGTA
CGTATGGAAGAAAAGCGGAAGGAGAGGGAAGCTGAGGAGGTATGCCAGCAGTCTGGGGGC
AATGGGGAGAGGCTGCCATTAGCCCTGGATCAAGGAAGTACCAAGAAGAGCAGCAACAGT
ACCAAAGGTACCAAAAAGTTCCGACTGGAAGGAACCCTCTTGAGGACCTACATCTGCCAC
ATCATCTTCAAGACTCTCTTCGAGGTGGGCTTCATCGTGGGCCATTACTTCTTGTATGGC
TTTCGGATCCTGCCCTTGTATCAGTGCAGCCGTTGGCCTTGCCCCAATGTGGTGGACTGC
TTCGTGTCTAGGCCCACCGAGAAGACCATTTTTATCCTTTTCATGCTCTCGGTGGCCTCT
GTGTCCCTCTTCCTCAATATCATGGAAATCAGCCACCTGGGCCTGAAGAGAATTCGATCT
GCTTTCAATAGACCTGCTGAGCAGCCACTGGGGGAGATCCCTGAGAAATCCCTCCACTCC
ATTGCTGTCTCGTCCATCCAAAAGGCCAAGGGCTACCAGCTCCTAGAAGAAGAGAAAATC
GTGTCCCACTACTTCCCCCTGACCGAGGTTGGGGTGGTGGAGACAAGACCACTTGCTGCC
ACCCCTTTCAGCCGTTTTGAAGAGAAGATCAGCACTGGGCCTCTGGGAGATCTGTCCCGG
GCTTACACGGAAACGCTGCCCTCCTATGCTCAGGTGGGAGAACAAGAAGTAGAGGCAGAA
CGTGAAGAAGAGGAGGCAGAGGCCCCGCCAGAGGAGGAAGAGAAGAGGCAGGAATCAGAG
ACAGGGATCCCGGAGGGGCAGGAAGCCCCTCCTGTAGGGCAGGAGGAAGAGAAGGAGAAA
GCAGGGACCCCCGTTGAAATGAAGGCAGGAGACAAGCAAGAGCTGCCAGTGGAGAAGATA
CCCCTGTGCCCAGAGCTGGCAGCGGATGACACCCGACCCCTCAGCCGGCTAAGCAAAGCC
AGCAGTCGAGCAAGGTCAGATGATTTGACTGTATGA

>Md-GJA10-XM_007484323
ATGGGAGACTGGAATTTGCTGGGCAGCATTCTAGAGGAAGTCCACTTCCATTCAACCTTG
GTGGGGAAGATCTGGCTGACCATGCTATTCATATTCCGTATGCTGGTGCTTGGAGTGGCA
GCAGAGGATGTTTGGGATGATGAACAGTCAGCATTTATCTGTAACACCCAACAGCCTGGC
TGCAGCAACATTTGTTATGATGATGCTTTCCCCATTTCTTTGATCAGGTACTGGGTTTTA
CAGATTATCTTTGTATCTTCTCCCTCTCTTGTATACATGGGCCATGCACTTTATAGACTC
AGGGCCTTTGAAAAGGAGAGGCAGAGGAAGAAAGTACAACTTCGTGCCCTGCTGGAAGAA
CCTGAGCATGACCAAGAGGAGCACCAAAGAATTGAGAAGGAACTGAGGAGATTAGAGGAA
CAGAGGAAGGTACACAAGGCACCCCTGAAAGGATGCTTGCTACGTACTTATATATTGCAT
ATCTTGACCAGATCTGTATTGGAAGTAGGGTTTATGACAGGGCAGTATATTCTCTATGGG
TTTCAAATGCACCCCCTTTACAAATGCAGTCGATTTCCTTGCCCCAATTCAGTGGACTGC
TTTGTGTCCAGGCCCACAGAGAAGACCATTTTCATGTTGTTCATGCACACCATAGCAGCT
ATCTCCTTGTTCTTAAACATTTTGGAAATATCTCACTTGGGAATCAGGAAAATCACCAAG
GCATTATATGGTGGGTCTAGTAGTGAGAGTGAAAGGGAACTGTATCACTCAAAGAAAAAT

TCAGTGACCCAGCCCTGTGTAACTCACTCTTTATTACATGAGAGCATCCCTTTGTCCCAG
CCTCCTAACCACTTTTGGCTAGAGAAACAAGTAATTGGAACTGGTAATACAGAGTATAAA
ACAGCGTGGCAACCCAACCATCACAACCAGACTGAGGGAGCACCTCCACCTGGCAAGAAG
AACTGGTCCAAGAGGGATCGGCATCATAGAGTGTTTCAATTTAGCTCTCATTGTCCTCAG
GATCTTGATTATGGTGTCCAGCACTTACAACACTGCAAACATCAGAACCATGGACAGCAC
CCACGTTCTTCCTTCAGTAAAAGGGGCATTCTTTCCAAATCGCATCAGGCCAATCTTACA
AATTGCTCTTCTTGCACACTAAGTCCAGGGGAACAGCCCTACAGTCTTTGGAGCCCAAGC
AGGTCCTCAATAGACTTGACTGCTCACTGCAAGACAAGTGATAACATCAAGTCAGAGTGT
TTTGACTCGGCAGAGAAAGGGTCTCACCCAGGTAGCCGTAAAGCCAGTTTCCTATCTAGG
CTTTTGTTTGAGAAGGGCCAGTTATCCAAAGCTTCAGAAAGCTCTGATTCCCAGCATAGC
TCTATCTTGGACTTCCAGCACTGGGGTGAAGATGACCATGCCTCCAGCTCTCCGCCTCCA
GGCACTGGGCGCAGAATGTCAA**TGAA**AATGCTCTTAAAACTTTCATCTATCATGAAAAAA
TAA

>Md-GJB1-XM_007507588
ATGAACTGGACAGGCCTGTACGCCTTACTCAGTGGTGTAAACCGGCACTCTACCGCCATC
GGGCGCGTCTGGCTCTCAGTCATCTTCATCTTTCGCATCATGGTGCTGGTGGTGGCCGCT
GAGAGCGTGTGGGGAGATGAGAAGTCCTCCTTCATCTGCAACACCATGCAGCCTGGCTGT
AACAGTGTGTGCTATGACCACTTCTTTCCCATCTCCCACGTGCGCCTGTGGGCCCTGCAG
CTCATCCTGGTGTCCACACCGGCCCTGCTTGTGGCCATGCATGTGGCCCACCAGCAGCAT
ATGGAGAAGAAGCTGTTGCGACTTGAGGGCCACGGGGACCCCCTGAGCCTGGAAGAGGTC
AAACGGCACAAGGTGCACATCTCAGGCACACTGTGGTGGACCTATGTCATCAGTGTGCTC
TTTCGCCTGCTCTTTGAGGCTGTCTTCATGTATGTCTTCTACCTGCTCTACCCAGGCTAC
GCCATGGTGCGCCTGGTCAAGTGTGACAGCTACCCTTGCCCCAATGTGGTGGACTGCTTC
GTGTCACGGCCCACTGAGAAGACCGTCTTCACCGTCTTCATGCTGGCTGCCTCAGGCATC
TGCATTGTGCTCAACGTGGCAGAGCTGGTGTACCTCGTTGTCCGTGCCTGTGCCAGGCGG
GCCCAGCACCGCTCCAACCCACCCTCACGCAAGGGTTCGGGATTTGGCCACCGGCTGTCC
CACGAGTGCAAACAGAACGAGATCAACAAGCTGCTCAGTGACCAAGATGGCTCCCTCAAA
GACATACTGCGGCGCAGCCCGGGCACTGGAGCTGGCCTCACTGAGAAGAGTGACCGCTGC
TCTGCCTGTTGA

>Md-GJB2-XM_007495197
ATGGACTGGAGTACTCTACAGACTATTTTGGGGGGTGTCAACAAACACTCCACCAGCATA
GGCAAAATCTGGCTCACTGTCCTCTTCATTTTCCGCATTATGATCCTGGTCGTGGCTGCT
AAGGAGGTGTGGGGAGACGAGCAGGCTGATTTTGTCTGCAACACTCTCCAGCCCGGATGT
AAAAATGTGTGCTACGACCACTTTTTCCCCATCTCGCACATCCGCCTCTGGGCTCTGCAG
CTGATCTTTGTGTCGACCCCCGCGCTCTTGGTGGCCATGCACATTGCTTACCGGAGGCAC
GAGAAAAAGAGAAAGTTCATCAAGGGAGAGATAAAGTCTGAATACAAAGACATAGAAGAA
ATCAAGAAACAAAAGGTTCGCATTGAAGGAGCCTTGTGGTGGACCTACACAAGCAGCATT
TTCTTCCGAGTCGTCTTTGAAGCGGTCTTCATGTACGTGTTCTATTTTATGTACAACGGA
TTCTCCATGACTCGAATGGTGAAATGTAATGCTTGGCCCTGTCCCAATACTGTGGACTGC
TTTATTTCCCGACCCACTGAAAAGACAGTGTTCACTGTGTTCATGATTTCGGTGTCTGGA
ATTTGCATACTGCTAAATGTCATTGAATTGTCTTATCTGCTGATAAGATATTGTTCTGGG
AAGTCCAAGAAGCCAGTTTAA

>Md-GJB3-XM_016422482
ATGGACTGGAAGACTCTGCAGTCACTCCTGAGTGGTGTCAACAAGTACTCCACAGCGTTC
GGCCGGATCTGGCTGTCGGTGGTGTTCGTCTTTCGCTTGCTGGTGTACGTGGTGGCGGCG
GAGCGCGTGTGGGGGGATGAGCAGAAGGATTTTGACTGCAATACACGCCAGCCCGGCTGC
ACCAATGTCTGCTACGATCACTTCTTCCCCATCTCCAACATCCGCCTGTGGGCCCTGCAG
CTCATCTTCGTCACCTGCCCCTCGCTGCTGGTCATCATGCACGTGGCCTACCGGGAGGAG
CGGGAGCGGCGGCACCGGGAAAAGCACGGGGACAAATGCGCCCGGCTCTACGAAAACGCC
AGCAAGAAGCATGGCGGGCTCTGGTGGACCTACCTGCTGAGCCTCTTCTTCAAGTTCATC
GTGGAAGTCGTCTTCCTCTACATTCTGCACACGCTGTGGTACGGTTTCTTCATGCCCCGC
CTGGTGCAGTGTGCCGGCGTGAGCCCCTGCCCCAACACCGTGGACTGCTACATCGCCAGG
CCCACCGAGAAGAAGATCTTCACCTACTTCATGGTGGGGGCCTCAGCCATCTGCATTGTC
CTGACCATCTCTGAGATCTGCTACCTCATCTCTAAGAGACTATCCCGGAGCTTCTGCCAA
AAGAAGCACAAGCGCTCTGCCCTGCACTCTCCCTCCTCCAGCAGGGCATCCACCTGCCGC
TGCCACCATCTGCTGACCCACCATGGGGGCGAAGAGGGCATGGTCCAAGGCAAGGGGGCA
GAGGCCCTTCGGGCCTCCGCACCCAACCTGACTCTCATCTGA

>Md-GJB4-XM_016422483
ATGAACTGGGCATTCCTCCAGGGCCTCCTGAGTGGGGTCAACAAGTACTCCACGGTTCTT
GGCCGAGTCTGGCTGTCAGTGGTACTGATATTTCGAGTACTGGTATACGTAGTGGCAGCA
GAGGAGGTGTGGGACGATGAACAAAAGGACTTCGACTGCAACACTCGACAGCCAGGTTGT
GCGAATGTTTGCTACGACCACGTCTTCCCCATTTCCCACGTCCGCTTGTGGGCCCTTCAG
CTCATTCTGGTCACATGTCCCTCGCTGCTCGTCGTCATGCACGTAGCCTATCGAGAGGAG
CGGGAGCGGAGGCACCGGATGAAGCATGGCCCCCAGGCCAGGCCCCTCTATGGCAACCCA

GGGAAAAAACGTGGAGGTCTCTGGTGGACCTACCTGCTAAGCCTTATATTCAAGGCTGGC
GTTGATGCCACCTTCCTGTACATCTTCCATCGCCTCTATAATAACTATGACATGCCCCGT
GTGGTGCACTGCTCCGTGGACCCCTGCCCCAATGTGGTAGATTGCTTCATCTCCCGGCCC
ACGGAAAAGAAGGTCTTTTCCTACTTCATGGTGGCCACAGCTGTCATTTGCATCCTGCTC
AATCTAGGGGAAGTGTCCTACCTGATCTGTAAGAGAGCCCAGGAGCTCCTAGGGCCACAG
AACTCGAAACAGCCTCGGCGGCACCATCGGAGGCATGGCCACCATGGCCCCTTGGGGACC
CTGCAGGATGCCTGCCCCCCTTATGCTCCTGCCCAGGCTTTGTCCCAAGGTGACCCCACC
AAGGAGGCTACATTGCCTTGCTAA

>Md-GJB5-XM_007492760
ATGAACTGGGGAATCTTCGAGGATCTGCTGAGCGGCGTCAATAAGTATTCTACAGCTTTC
GGCCGCATCTGGCTCTCCCTGGTCTTCATCTTCCGCGTGCTGGTCTACCTAGTGACCTCC
GAGAAGGTGTGGAGCGATGACCACAAAGACTTCGACTGCAACACGCGCCAGCCGGGCTGC
TCCAACGTCTGCTACGACCACTTCTTCCCCATCTCCCACGTCCGCCTGTGGGCGCTGCAG
CTCATCCTGGTCACGTGCCCCTCGTTGCTCGTCATCATGCACGTGGCGTACCGGCAGGCC
CGGGAGCTGAGACACCTGGAGCAGGTGGGCGAAGGAGGCGGGCGCCTCTATCCAAACGTA
GGCAAGAAGCGCGGAGGGCTCTGGTGGACCTACGTCTTCAGTCTGGTCTTCAAGGCCAGC
GTGGATTCAATCTTCCTCTACGCGTTCTACCGCCTCTATCAAAACTACCTGCTCCCGCAC
GTGGTCTTCTGCAGCGAGGACCCCTGCCCCCATACCGTGGACTGCTTCATCTCCAAGCCC
ACGGAGAAGAACATTTTCACACTCTTCATGGTGGCTACCGCCATCGTCTGCATCTTGCTC
AACCTGGTAGAACTGGGTTACTTGGTCAGCAAGAGGTGCTGGGAGTGCCGGGAGGTTGGG
AGAATGGATACCAAGAAGGATTTGCTGTCCGGGGGCGATCTCATCTTCCTGGGTACCGAC
CCCAAGCCACCGCTGCTGCCTTTTTCTCCTGACTCCCCCCGAGACCAGGTGAAGAAAACC
ATGGTATAA

>Md-GJB6-XM_007495198
ATGGACTGGAGTACACTGCATACTTTCATTGGAGGCGTAAATAAACACTCCACCAGCATA
GGGAAGGTTTGGATCACCGTCCTCTTCATTTTTCGAGTCATGATCCTTGTCGTAGCTGCT
CAAGAAGTATGGGGAGATGAGCAAGAAGATTTTGTCTGTAACACACTGCAGCCAGGATGC
AAAAATGTGTGCTATGACCACTTCTTCCCTGTTTCTCATATCAGACTTTGGGCTCTTCAA
CTAATCTTTGTCTCCACTCCAGCACTTCTGGTAGCCATGCATGTAACTTACAATAGACAT
GAGAAGGAAAGACAGTTTAGGAAAGGGGAGAAAGGGATTGAATTCAAAGACTTAGAAGAA
ATTAAAAAACAAAGGGTACGAATTGAGGGGTCTTTGTGGTGGACTTACACTAGCAGTATT
TTCTTTAGGATTATCTTTGAAGCCTCCTTTATGTATGTGTTTTACTTTCTTTACAATGGC
TATAACCTGCCCTGGGTGGTGAAATGCAGTATTGATCCTTGTCCCAATATTGTGGACTGC
TTTATTTCAAGACCCACTGAAAAGTCTGTTTTTACCATTTTCATGATTGCTGCATCTGTG
ATTTGCATGCTGTTAAATGTGGCTGAATTATGTTACTTGCTCATGAAACTGTGCTTCAGA
AGATCCAGAAGAGCACAGGTTCAAAGAAATCACCCTAATCATGCCATAAAAGAAAGCAAA
CAGAATGAAATGAATGAGCTGATTTCAGACAGTGGACAAAATGCAATCACAGGTTTCCCA
AGTTAA

>Md-GJB7-XM_007484297
ATGACTTGGATGCTCCTCAGAGATCTCCTAAGTGGAGTAAATAAATATTCAACAGGAATT
GGTCGAATCTGGCTGGCTGTCATCTTTATGTTCCGTTTGCTGGTCTACATGGTAGCTGCA
GAACATGTTTGGAAAGATGAACAGAAGGAATTTGAATGTAACATTAGGCAGCCTGGTTGT
GAAAATGTCTGTTTTGACTACTTCTTCCCCATCTCCCAGGCTAGGCTTTGGGCCTTGCAG
CTGATCATGGTCTCTACTCCTTCTCTTCTGGTTGCTTTGCATGTGGCCTACCGTTTGGGC
CGTGAAAAAAGGCACAATAAGAAATTTTATGTTAGTCCAGGTAGCAAGGATGGGGGCCTA
TGGTACACTTATATCATTAGCCTTGTTGTCAAAACTGGTTTTGAAATTGGCTTCCTGGCT
TTGTTTTACAAGTTATATGATGGATTTAGAGTACCCTACCTTGTGAAATGTGATATAAGA
CCTTGCCCTAACACTGTGGATTGCTTTATCTCCAAACCTACTGAGAAGAAAATCTTCCTT
TACTTCTTGTTAGTCACATCATGTCTGTGCATTGTTTTGAATATCGTTGAGTTAGGTTAT
CTGGTTCTCAAGAGTTTTGTAAAGTGCTGCCTTCAACGATATGCTCAGAATTTCAAATCT
TCAGCTTATAAGTGTCATAACCTTGATTATGCCATGTGCAATGAGATTGTCCCGAAACTG
CACCAGAAGCACAACTCTGACTGTTCCAGAAGCATTCCTCAAAAACTTGATAGAAGTGAT
TTGCAAGAATGGTGA

>Md-GJC1-XM_007482452
ATGAGTTGGAGCTTCCTGACTCGACTGCTAGAGGAGATCCACAACCATTCCACATTTGTG
GGGAAGATTTGGCTCACCATATTGATAGTTTTCCGGATAGTCCTCACTGCTGTGGGTGGG
GAGTCTATCTACTATGACGAGCAGAGCAAATTTGTATGCAACACAGAACAGCCCGGCTGC
GAGAATGTCTGCTATGACGCTTTTGCCCCACTGTCCCATGTCCGCTTCTGGGTATTCCAG
ATCATCCTTGTGGCCACCCCCTCGGTGATGTATCTTGGCTATGCCATCCACAAGATTGCC
AAGATGGAACATGGTGAGGCAGACAAGAAGGCATCAAGAAGCAAGCCCTACGCAATGCGC
TGGAAGCAGCACCGGGCCCTGGAAGAAACTGAGGAGGACCACGAGGAGGACCCCATGATG
TATCCGGAGATGGAGTTGGAGAGTGAGAAAGAGAACAAGGAGCAGAGTCAGCCTAAACCC
AAGCACGATGGTCGGCGGCGGATTCGGGAAGATGGCCTTATGAAAATCTACGTGCTACAG

TTGCTAGCAAGGACTTTGTTTGAGGTGGGCTTCCTGGTGGGGCAGTATCTTCTCTATGGC
TTCCAGGTCCGCCCATCTTATGTGTGCCGCAGAATCCCCTGCCCTCATGAAATAGACTGC
TTCATTTCTAGGCCCACCGAAAAGACCATCTTCCTGCTAATAATGTACGGTGTGACGGGC
CTCTGTCTGATCCTCAACATTTGGGAGATGCTCCATTTGGGGTTTGGGACTATCCGTGAC
TCACTAAATAGCAAAAAGAGAGAACTGGAAGATTCGGGTGCTTATAACTATCCTTTCACT
TGGAATACTCCATCCGCTCCACCTGGCTATAACATTGTGGTCAAACCAGATCAGATCCAG
TACACCGAACTGTCCAATGCAAAGATCGCCTACAAGCAGAACAAGGCCAACATTGCTCAG
GAGCAGCAATACGGGAGCAATGAGGAGAACCTTCCCGCAGACCTCGAGATGCTGCAGCGG
GAAATCAAAGTGGCCCAGGAACGCCTGGATCTGGCCATCCAGGCCTACAACCACCAGAAC
AACCGCCACGGCCCCCGGGAAAAAAAGTCCAAAGCTGGGTCCAAAGCTGGGTCCAACAAA
AGCAGTGCCAGTAGCAAATCTGGGGATGGGAAGAATTCTGTCTGGATTTAA

>Md-GJC2-XM_007499765
ATGAGCTGGAGCTTTCTGACGAGGCTGTTGGAGGAGATCCACAACCATTCCACCTTTGTG
GGCAAGGTTTGGCTGACCGTGCTGATCGTCTTCCGGATAGTGCTGACCGCAGTAGGGGGG
GAGTCCATCTACTCGGACGAGCAGAGCAAATTCACCTGTAACACCCGCCAGCCGGGCTGC
GACAACGTGTGCTATGACGCCTTTGCCCCCCTCTCCCACGTTCGCTTCTGGGTCTTCCAG
ATCGTGGTCATCTCTACGCCGTCCGTCATGTACCTTGGCTATGCCATCCATCGTCTGGCG
AGAGCCTCGGAGGAGGAGCGGCGCCGGGCCAGGCGCGGGCGCCAGGGTGGCCGCGGGGGC
CGCAGGCGTCCCCAGAGGAGGAGGCTGCCGCCGATGGCCCACCCGGGCTGGGCAGACACC
CCGAATGGCGGAGAGGAGGAGCCCATGATTGGCCTTGGGGCTGGCATGGGGGAAGAAGAG
GAGGCTGGCGATGGGAGCAGGGAGGACAGAGAGCAAGAAGAAGAAGAAAAGGAGGCCTTG
GCGGGGGAGAAAGGCAAGGGGGCTCCGGAGGCCGGGCCTGCCAACCAGAAGCATGATGGG
AGGCGAAGGATCCAGCAGGAGGGGCTGATGAAGATCTACGTGTTCCAGCTGTTGGCTCGG
GCCTCCTTTGAGATCTGCTTCCTGGTCGGGCAGTACCTCCTGTATGGCTTCGAGGTGCAA
CCTGTCTTCCGCTGCAGCCGAGATCCGTGCCCTCACACCGTTGACTGCTTTGTGTCTCGG
CCCACAGAGAAGACTGTCTTCCTCCTGGTGATGTACGTGGTCAGCTGCCTGTGCCTCATC
CTGAATCTCTGTGAGATGGCCCACCTGGGCCTGGGCAGCTTGCAGGATGCGGTGCGGAGC
CGCAGGGTCGGGGGCCGCGACCAGGGTTCGGCCGGCTATCCTTCCCCCCCACCCGCGCCC
CGGCAGCTACCCCATGGCTACCTGTACGCTCGCAACATCTCCTGCCCTCCTGAGTACAAC
ATGGTGGTCAGGAATGAGAGGGCAGCGGCAGCCGGGCGCCTCGTGCCTGGAGGCCTCCTG
GCCCACGAGCAGAACCTGGCCAATGGCGTCCTGCAGGAGCTGCAAGAGCTTCAGGGCCCT
GGCTCAGAGGAGAATCCTCCACCCATGGATCTGGCTGCTGCCTTCCGAGCTACTCATCAT
CGGGCTGGCACCCAGGACCCTGCCCCTGGGGTCAGGAGCAACGGCTTTCCTGCTTATACA
GCTCAGGTCGGGCCACTACCTTCAAGGACTGACAGCCCAGCCTCGGCAGGCACCATTGTG
GAGCAGAATCATTCCGATGGTGCCCCAGGGCAGCAAGGGGCCAAAGCCAAATCCAACTCG
GAGAAAGGCAGCAGCGTCAGCAGCAAAGATGGCAAGACATCTGTATGGATATGA

>Md-GJC1like-XM_007499115
ATGAGCTGGGCGTTCCTGACGAGATTGCTGGAGGCTGTGACCCAGCACTCCACTCTGGTG
GGGAAGCTCTGGCTCTCCGTGCTGGTCGTTTTCCGACTAGTGCTCCTGGCCGTGGGTGGG
GAGGCCATTTACCGGGACGAGCTCAGTGGATTCGTGTGTAACACTCGGCAGCCTGGATGC
CAAAACGTCTGCTACGACACCTTCGCACCCCTCTCGCATGTGCGCTTCTGGGTATTCCAG
ATCATTCTCGTCACCGCGCCCACCGTGTTCTATTTGGGCTATGCCGTGCACCATCTGTCC
CGGCGCCAGATGACCCAGAAGCAAGAGAAGGAAGAGGAGGAAGGCGAGAAGGAGCCTATG
ATCAAAGAGAAGAAGCCTCAGATGTCCCCTGACGATGGGGCCCATGACGGTCGCAGGAGG
ATCCGCAGGGATGGGCTCCTAGGGGCCTATGTTGCGCAGTTGCTGGTGCGGACTGCCTTA
GAGGTGGCCTTCCTAGCGGGCCAGTATCTGCTCTTTGGCCTGAAAGTGCCTCCCGACTAT
GACTGCAAGCAGAATCCTTGCCCTCATGTCGTCGTCTGCTATATATCTCGTCCCACGGAG
AAGACCATCTTCCTGCTGGTCATGTATGCAGTCAGTGGCCTCTGCCTCCTGCTCAATCTG
ATCGAGTTGCTGCACCTGGGCCTGGGGAGCGTGAAAAATGGTGGAAGCCGCCCTGACCCA
GCGCCCCATTCCAAGAGTCTCAAGCTGGAGCTGCAGCAAATGCAGAAACAACTGCACCTG
GTCCAGAAGCAAATGGACATGGGACTGCATCTGGGCACACCTTTGGTCACAGTGCCTACC
TGTTCAGGGCAGCCTCCCACCTACAATCTCTCTACTCAGCAAAATCGGCACAACCAAGCC
CAGAAAGAGACCTGGCTCTCCAAGACAGGTACGTAG

>Md-GJC2like-XM_001370819
ATGAGCTGGGCGTTCCTGACCAAGTTGCTGGAGGCTGTGACCCAGCACTCCACCCTGGTG
GGGAAGCTCTGGCTCTCCGTGCTGGTCGTTTTCCGACTAGTGCTCCTGGCCGTGGGTGGG
GAGGCCATTTACCGGGACGAACTCAGTGGCTTCTCCTGCAACACAGCACAACCTGGCTGC
CTAAATGTTTGCTACGATACCTTCGCGCCTCTCTCCCACGTGCGCTTCTGGGTATTCCAG
ATCGTCCTGGTCACTGCGCCCACCGTGTTTTATCTGGGCTATGCCGTGCACCATCTGTCC
CGGCGCCAGATGACCCAGAAGCTAGAGGAGGAGGAGTCTCTGATCCCGGGAAAGAAGTCC
TGTAAGGATGGGGCCCACGATGGTCACAGGAGGATCCGCAGGGATGGGCTCCTAGGGGCC
TATGTCGCCCAGTTGCTGGTGCGGACTGCCTTAGAGGTGGCCTTCCTAGCGGGCCAGTAT
CTGCTCTTTGGCCTGAAAGTGCCTCCCGACTATGACTGCAAGCAGAATCCTTGCCCTCAT
GTCGTCGTTTGCTATATATCTCGTCCCACGGAGAAGACCATCTTCCTGCTGGTCATGTAT

GCAGTCAGTGGCCTCTGCCTCCTGCTCAATCTGATCGAGTTGCTGCACCTGGGCCTGGGG
AGCATAAGAAATGGTAGAAGCCTGAAAAATGGTGGACATCACCCTGCCCCATCTCCTCAT
TCCAAGGCTGGCAAGCTAGAACAAATGCAGAGACAGTTGAATCTGGTCCAGGAGCAAATG
GGCATGGTATTCAATCAGGGCCCTCTTTTGGCTACAGAACCTACCTCTACAGGGCAGTCT
CCAGACTACAGTTTATTTGCTCAGCAAAACTGGCATAACCAAGCCCAGAAATAG

>Md-GJD2-XM_003340035 Splice site
ATGGGGGAATGGACCATCTTAGAGAGGCTGCTGGAAGCCGCCGTACAGCAACATTCCACT
ATGATTGGGAGGATCCTGCTGACAGTGGTGGTGATCTTCCGGATCCTTATCGTGGCCATA
GTGGGTGAGACAGTGTATGATGATGAGCAGACCATGTTTGTATGCAATACCCTGCAGCCA
GGCTGCAACCAAGCCTGCTATGACCGAGCTTTCCCTATCTCTCATATCCGCTACTGGGTC
TTCNAAATCATCATGGTGTGCACTCCTAGCCTCTGCTTCATCACCTACTCAGTTCATCAA
TCTGCCAAGCGAGCCGGGGGTGGGAGCAGTGGTGGGGGCAAGCGGTTAGACAATAAGAAG
CTGCAAAATGCCATTGTAAATGGAGTACTACAAAATACAGAGAATACTAGCAAGGAGACT
GAGCCAGACTGTCTAGAGGTTAAGGAGTTGGCCCCACACCCATCTGGGTTACGTACAGCA
GCCCGTTCTAAGCTCCGTCGGCAAGAGGGCATCTCCCGCTTCTACATTATTCAGGTTGTT
TTCCGTAATGCCCTAGAAATTGGGTTCCTGGTAGGCCAATACTTCCTTTATGGCTTCAGT
GTCCCTGGACTGTATGAGTGTGACCGCTATCCCTGTATCAAGGAGGTAGAATGCTATGTT
TCCCGGCCCACAGAGAAGACAGTCTTCCTCGTGTTCATGTTCGCTGTGAGTGGCATTTGT
GTTGTCCTCAATCTGGCTGAACTCAATCACCTGGGCTGGCGCAAGATTAAGCTGGCTGTG
CGTGGTGCACAAGCCAAGAGGAAGTCAGTCTATGAGATCCGCAACAAGGACCTGCCTCGG
GTCAGTGTACCGAATTTTGGCAGGACTCAGTCAAGTGACTCTGCCTATGTGTGA

>Md-GJD2like-39.2-XM_001376506
ATGGGTGACTGGTCATTTCTGGGCCGGCTTCTGAATGAAGTCCAGAACCATTCCACTGTC
ATCGGTAAGATCTGGCTCACCGCCCTCCTCATCTTTCGGATTCTCCTGGTCACATTGGTG
GGCGATGCCATCTACGGAGATGAGCAGTCTAAGTTCACCTGTAACACCCTCCAGCCGGGC
TGCACCAACGTCTGCTACAATAGTTTTGCTCCTATCTCCCACCTTCGCTTCTGGATCTTC
CAGATTGTTCTGGTGGCCACACCATCCATTTTCTACATCGTGTGTGTGTTGCATCAGGTG
GCCTTGGAGGAGAGGATGGATGTGGAGAGGGACCGTCTGCTGGAGCTGTGGCGAAGACTG
GTGCCAGCCACTGGGGAAGTCCTTCCTGGAGTGGGGTCTGGGGTCCTAGTGCCCTCTAGC
TCCTTTGAAGGCCACAGTCTGGATGAGGAGGAGGTCCTTCCAAAGCACCTCCAGTCCACC
TCTCAGGACCCCATCTACCTGGCCAACCGGGCATTGATCATTTACATCGCCCACGTGGTG
CTGAGGGCCTTCCTGGAGTTGGGATTCCTAGTGGGGCAATACTACCTGTTTGGGTTTGAT
GTGCCTCATTTGTATCGCTGTGAAACCTACCCATGTCCCACCAAGACGGACTGCTTTGTC
TCCAGGGCGACAGAGAAAATGATCTTTCTGAATTTTATGTTCGGCGTTGGGCTTGGCTGT
TTTCTTCTGAACTTGGCAGAATTGCACTACCTGGGATGGCTCTTTACCTTCCGGATGCTC
TTCAAGGCTTGTGTCAATTGCTGCCAATATCTAGGCAAAGCACCCCCATCCCACAGGTCC
CGACTGCTGCCTCTTCTGGACTCAAGTCAGGAGAGGATCCTTCTGGAGGCCTCCTTGCTG
CCTGCGTGGGGGTCAGGGCATCTCAGGGCCCAGCACACTGTGATACCTCTGACAATCGAT
GCAGCCCAGGCCACTTTGGACCACAAATCTCAACAGGAAAAGTCCTCCAGGATGCCCCCC
AGCAAGAAATCTTGGCTCTGA

>Md-GJD3-XM_001365802
ATGGGGGAATGGGCCTTCCTGGGCTCGCTGCTGGACTCGGTGCAGCTGCAGTCCCCCCTG
GTGGGCCGCCTGTGGCTGGTGGTCATGCTGATTTTCCGGATCCTGGTGTTGGCCACGGTC
GGGGGGGCCGTGTTCGAGGATGAGCAGGAGGAGTTCGTGTGTAATACGCTGCAGCCTGGG
TGTCGTCAGGCCTGCTATGACCGGGCCTTCCCCATCTCCCATTACCGCTTTTGGCTTTTC
CACATCTTGCTGCTCTCTGCTCCTCCCGTGCTGTTCATCATCTACTCCATGCACCAGGCC
AGCAAAGGCCCCAGACCCCAAGGAGATGGGATGAAGGAGCAGGAGGAGTGGAGCGGCAGC
CTGGGCCAGCGCCTGGCAGGGCCCGGGGCTCGCCGCTGCTACCTGCTGAGCGTGGCCCTG
CGTCTGCTGGCTGAGCTGGGCTTCCTGGTAGGGCAGACCCTGCTGTACGGCTTCCGGGTG
GCCGCCCGTTTTCCCTGCACCCAGACCCCGTGCCCGCACGTGGTGGACTGCTTTGTCAGC
CGCCCAACGGAGAAGACGGTCTTTGTGATTTTCTACTTTGCTGTGGGGCTTCTCTCTACC
CTCCTCAGCGTGGTCGAGCTGGGCCATCTCTTCTGGAAGGGGCGCCGAAGTCAGAACAAG
GGTGCCCTTTGGCTAGGCAAGGTGGTCACAGGAGAGAAAGACAACCACTGCAACCAGGAG
CAGGAAGAGGCCCGGAAGCTTCTCCTGCCACTCTCCCCACCAAAAAGGGCCCCTCCACCC
AGCCCCATGCAGGGCGCCCCACCTGCCTATGCCCACAAGTTGCCACAGGGTGGCAGCGAG
GGCAGCAGCAGCAGGAGCAAGTGCTCGCTGTCCACAGCCAGAGAGGACCTCTCCATCTAA

>Md-GJD4-XM_0013474328 Splice site
ATGGAACGTTGGGATTTGCTGGGGTTTCTGATCATCACATTGAACTGCAATGTGACAATT
GTGGGGAAGATCTGGCTCATCATCATGATAATGCTGAGGATGGTGGTAATTATTTTGGCA
GGCTATCCAATCTACCAAGATGAACAAGAGAGATTTATTTGCAATACTTTGCAACCAGGA
TGCTCCAATGTATGCTATGACATCTTCTCTCCTGTATCTCACTTCCGATTTTGGCTGATC
CAGAACGTATCTGTTCTCCTACCTTATGCCATGTTCAGTGTTTATGTCTTTCACAAAGGA
GCCCTACGTGCTGCAATGGGGGCCCGCCAACCAGATTGCTGTAAAGGAAGGAATATTCTC
CCTGACCAGAGAGGAGTGAAGGGGTTTGGCTATTCTCATCTAAATTTCCCTGACTTTTCC

ACTGCATATATAATACAACTTCTTCTGAGGATCCTGACCGAAGCTGCTTTTGGTGGTGCC
CAATACTATCTCTTTGGATTCTGGGTTCCAAAGCAATTCTCCTGCTACCATTCTCCTTGT
ACAAGTGTGGTGGATTGCTATATCTCCCGGCCCACAGAGAAATCAATAATGATGCTTTTT
ATTTGGGGAGTCAGTGCTCTATCCTTTCTTCTAAGTTTTGTTGACCTAATTTGTTGCATG
CAGAGATGGCTGAGACGGAAGCATTTGGCTAAACGGATGATGAAAAATGTTTGTGTCAGT
GAAGAGCATGGCTCCCCATACATCCCTCCTGGTCAAGCCAAGCACTGTTTGACCTCAGAG
GTAAGGCAGGAACCGCCACAGCTGGTGAAAAGGTTCAGTCAGATTAGTGAGAATGCTGGG
TGGCCTGAGTCTAGAGGGGAAGAAGATATATCTCTTCATCCTATGGTGTGGCCCAAAGAT
GTAGCTTCCAGGTCAAACCTTAATAGCCCAGGTCATAAGTCTTGCTCATCTGGAAGAATG
ACTTTTCCAGATGAAGATGGCAGCGAAGTGATGTCCAATGGCAGTGAACAGCAAGGGATA
GCTTCTAAAGAGATGCAAAGCAGGCCTTTCAAAGAGGTTCCTAGGTCCAAGGACAGCTCC
CAGCTTGGAGAGTTTGCCTCTGCCCCTCGAAGCCGACTTGGAGGACATTATTCATCCAGT
GAGCTGAAACCTTCTGCCTCTCAGGTGAGCTGTGGCAGCCCTAGCTACCTAAGGGCCAAA
AAGTCTGAATGGGTATGA

>Md-GJE1-XM_001380882 Splice sites
ATGTCTCTAAATTACATCAAAAACTTCTACGAAGGATGTCTCAGACCTCCTACAGTGATC
GGTCAGTTCCACACGCTTTTCTTTGGCTCAGTTCGAATGTTCTTCCTTGGAGTGTTGGGC
TTTGCAGTCTATGGGAATGAAGCCTTGCATTTTAGCTGTGATCCAGACAAGAGGGAAATC
AATCTCTTCTGTTACAACCAGTTCAGGCCAATAACTCCACAGGTATTCTGGGCATTACAG
CTAGTGACTGTACTGGTTCCTGGAGCTGCTTTTCATCTTTATGCTGCATGTAAAAGCATC
GATCAAGAAAGCATACTTCAGAGACCCATCTACACTGTCTTTTATATCCTCTCTGCCTTA
TTAAGAATAATTCTTGAGGTGATAGCCTTTTGGCTACAAAGTCATCTTTTTGGTTTTCAA
GTAAAATCTCTTTATCATTGTGATGCTAGTTTGCTTGAAAAAAGATTGGGTATCATAAGA
TGCCTGGTCCCGGAACATTTTGAAAAAACCATATTTCTCATTGCCATGTACACATTTACT
GCAGTCACAGTGGCATTGGGATTTGCTGAAGTTTTTGAGATCTTATGTAGAAGATTAGGG
TTTTTAAGTGGATAG

Suppl. Fig. 4. *GJC1like* and *GJA9* connexin sequences from other marsupials and platypus
Yellow: Conserved domains as defined by Cruciani and Mikalsen (2007)
Green: Conserved cysteine codons (cysteine signature)
Grey: 15 nt added at the ends of the conserved domains
Turquoise: Splice site.
Other colors are explained where necessary.

>Sh-GJC1like-XM_003761914 Sarcophilus harrisii
ATGAGCTGGGCGTTCCTGACGAAGTTGCTGGAAGCTGTGACCCAGCATTCTACCTTGGTG
GGGAAACTGTGGTTGTCCGTGCTGGTTGTGTTTCGCCTAGTGCTGCTGGCGGTGGGTGGA
GAGGCCATTTACCGGGACGAGTTAAGTGGCTTCTCCTGTAATACAGCGCAACCGGGTTGC
CAAAATGTTTGCTATGACGCCTTCGCACCCCTCTCCCACGTTCGCTTCTGGGTGTTCCAG
ATCATCCTGGTCACTGCGCCCACCGTATTCTACTTGGGCTATGCGGTGCACCACCTGTCC
CGGCGCCGGAGGATCCAGAAGCAAGAAGAGGAAGAGGAGCCCATGCTCAAAAAGAAGTCC
GAGAAGTCCCATGATGATGGAGCCCACGATGGTCGCAAGAGGATCCGCAGGGATGGGCTC
CTGGGGCCCTATGTTGGGCAGTTACTGGTGCGGACTGCCTTGGAGGTGGCCTTCCTACTA
GGCCAGTACCTGCTCTATGGCCTGGAGGTGCCTCCCTCCTATGTCTGCGTGCGTAAGCCT
TGCCCCCACACCGTGGATTGCTTTGTTTCTCGTCCCACGGAGAAGACCATCTTCCTGCTG
GTCATGTATGCAGTAAGTGGCCTCTGTCTCCTGCTCAACCTGATCGAGTTGCTGCATCTG
GGTCTGGGAAGTTTGAAGAAGGATAGAAGTCACAATGCTCCACTTTCTCATTCCAAGAAT
TTCCCACTGGAGCAGATGCAGAAACAGCTGCATCTGGTCCAGGAGCACCTGGACATGGCA
TTGCATATGAGCACACCTTTGGTCACAGCGCCTACCTATGCAGGCCAGTCTCCTTCCTAC
AACATATATGCTCAGCAAAATCTGCATAATCAAAGCGAAAAAGAGCCTCATTTCTCCAAA
ACAGATCAGTGA

>Koala-GJC1like-XM_020995466 Phascolarctos cinereus
ATGAGCTGGGCCTTCCTGACGAGGTTGCTGGAGGCTGTGACCCAGCACTCCACCCTGGTG
GGGAAGCTCTGGCTGTCCGTCCTGGTGGTGTTCCGCCTGGTACTCCTGGCCGTGGGCGGG
GAGGCCATTTACCGGGACGAGCTAAGTGGCTTCTCCTGCAACACAGCACAGCCAGGCTGC
CAAAATGTTTGCTACGACGCCTTCGCACCCCTCTCCCACGTACGCTTCTGGGTGTTCCAG

ATCATCCTAGTCACTGCACCCACCGTGTTCTACCTGGGCTATGCCGTGCACCATTTGTCC
CGGCGCCAAATGACCCAGGAACAAGAGGAGGAAGAGAGGGAGCCCATGATCAAAGAGAAG
TCCAAGAAGTCCGCTGAGGATGAAGCCCACGATGGTCGCCGGAGGATCCGCAGGGACGGC
CTCCTGGGAGCCTATGTGGGGCAGTTGCTGATCCGATCTGCCTTAGAGGTGGCCTTCCTG
ATGGGCCAGTACCTGCTCTACGGCCTGGAAGTGCCTCCCTCCTATATCTGCCAGCGCAGC
CCTTGCCCCCACACCGTGGACTGCTTTGTATCTCGTCCTACTGAGAAGACCATCTTCCTG
CTGGTCATGTATGCAGTCAGTGGCCTCTGCCTCCTGCTCAACCTGATTGAGTTGCTGCAC
CTGGGCCTGGGAAGCATGAAGAAGGGTAGAAACCACCCTGCCCCAACTCGTCATTCCAAG
AGTCTCCAGTTGGAGCAAATGCAAAAACAGTTGCATCTGGTCCAGGAGCACCTGGACTTG
GCGGTGCATTTGAGCACACCTTTGGCCACGGTGCCTGCCTATTCAGGCCAGTCTCCATCC
TACAGCACATATGTTCAGCAAAACCGGCACATCCAAGCCCAAAAAGAGGCTCTATTGTCT
AAGACAGGTATATTTGGGGTAGATTAA

>Oa-GJA9-XM_001512804 Ornithorhynchus anatinus (platypus)
ATGGGGGACTGGAATTTCCTGGGAGGCATTCTGGAGGAGGTCCACATCCACTCCACCATC
ATCGGAAAGATCTGGCTCACCATCCTCTTCATCTTCCGGATGCTCGTCCTTGGAGTGGCA
GCTGAAGACGTCTGGAACGACGAGCAGTCCGGGTTCGTCTGCAACACCGAGCAACCGGGC
TGTAGAAATGTCTGCTACGATCGGGCCTTTCCCATCTCCCTCATCAGATACTGGGTCCTG
CAAGTCATATTTGTGTCTTCCCCATCTCTGGTGTACATGGGCCATGCTTTGTACAGACTG
AGGGCCCTGGAGAAGGACAGGCAAAGGAAGAAAGCTCAACTCAAAGGGGAACTGGAGGCC
ACTGAGTTTGAAATGGTCGAGGATCGGAGAAAGCTGGAGCGAGAACTCCGTCAGCTCGAG
CAAAGGAAACTCAATAAAGCACCACTGAGAGGGGCCTTGCTTTGTACCTACGTGATACAC
ATTTTAACTCGGTCTGCAGTTGAAATTGGATTCATGATTGGCCAGTATCTTCTCTATGGA
TTTCGGCTCGATCCTCTCTTTAAGTGTCATAGAGATCCATGTCCAAATACAGTCGACTGC
TTTGTATCGAGGCCGACAGAGAAGACGGTCTTCCTGTTATTTATGCAATCCATAGCGGCT
GCCTCTCTTCTTTTAAATGTTCTAGAAATTGTCCATCTCGGTTTAAAAAGAATCAAAAAG
GGATTTGAGAGACCCAAATATAAATATAAAATGAATGATGAATGTGAGGACTCTGATTTG
AGCGAGGCAAAAAGAATTTCTGCAGCACAACCACCTTGTCTGGGCACGGCTACCAATCCA
CCCAAGACGCTCCCTTCTGCGCCTATTGGCTACACCGTGTTAGGGGAAAAACAAATGAAT
CCCACAGTGTACCCCGGCTTCAGTTCACCTTCAGTACTGCAGGCCCTTCAGGAAACTGGC
AAGAAAAGTCCGGCTAGTGACGAGAGAAATAAACCGTCTGACGAGACGTGCCTGAGTAGC
ATCGTGGAGGGTTATCCTGCGAACGCCAACTTGAGTCACAGCGAAGGGATAATCAGCGTG
GTGAGGGCAGAGATCGGTGGCACTCACAAGCAAGAGAACACCACCACCGGCCACCAGGCT
AATTTCGAAGCTGCCGTCCATCTGGGCTATCGGCCGGAGATGCCATTGGGGGCTGCCCTA
TACCCTTCACTGCAACCCGAAATGACTTTCTCCCCGCCAACCGATTGCACCCGCACAGCA
CGGAGCCTAAGCGACCCCTGGAACGGTTCGACGGGGGGTCTTCAGAGCAGAGGGTCACCT
CCCAGAGGTAACCTCAGGAGACAGAGCCGAGGGAGCACCGGCAGACCCCGAGCCCTCTCC
CGGGCGGACTCCCGACCACCCAGTAGGTCAAACAGCTCAGACTCTCCGGGGGAGATGAGC
TCGGGATCCAAACCCAGCAAAAGCTGTGACAGTCCTAGGGTTTTCCCACTTTCTCGGCGA
ATCTCTCTGGCGACTTGCAGCGTTAGCAGCAGGCGGGCCCCGACCGACCTTCAGATCTGA

Suppl. Fig. 5. Zebrafish (*Danio rerio*) connexins.
Yellow: Conserved domains as defined by Cruciani and Mikalsen (2007)
Green: Conserved cysteine codons (cysteine signature)
Grey: 15 nt added at the ends of the conserved domains
Turquoise: Splice site.
Other colors are explained where necessary.

```
>Dr-cx43-NM_131038
ATGGGTGACTGGAGTGCGTTGGGAAGGCTTCTTGACAAGGTGCAGGCCTACTCCACGGCC
GGAGGGAAGGTCTGGCTCTCTGTGCTCTTCATCTTCCGGATCCTTGTTCTGGGAACAGCA
GTGGAATCGGCCTGGGGTGACGAGCAGTCAGCTTTCAAGTGCAATACCCAGCAGCCTGGT
TGCGAGAATGTCTGCTATGACAAATCGTTCCCCATCTCGCACGTGCGCTTCTGGGTGCTT
CAGATCATCTTCGTGTCCACGCCGACGCTCCTGTACCTGGCGCATGTCTTCTACCTGATG
CGAAAGGAGGAGAAACTCAACCGTAAAGAAGAGGAGCTGAAGGCCGTGCAGAACGACGGC
GGCGACGTTGAGCTCCATCTCAAGAAAATCGAGCTCAAGAAGTTTAAGCATGGCCTAGAG
GAGCACGGCAAGGTGAAGATGAAGGGTAGCCTGCTGCGCACCTACATCTTCAGCATCATT
TTCAAGTCCATCTGTGAGGTGGTCTTCCTGGTCATCCAATGGTACCTCTACGGCTTCAGC
CTCTCTGCCGTGTACACATGCGAACGCACGCCTTGCCCTCATAGGGTGGACTGTTTCCTT
TCTCGGCCCACCGAGAAGACCATCTTCATCATCTTCATGCTAGTGGTTTCGCTCTTCTCG
CTTTTGCTCAACATCATCGAGCTCTTCTACGTGCTCTTCAAACGAATCAAGGACCGCGTC
AAAAGCCGACAAAACACACAGTTTCCCACTGGCACTTTGAGCCCCACGCCGAAGGAACTG
TCTACGACCAAATACGCGTACTACAATGGTTGCTCCTCACCAACTGCACCGCTCTCACCA
ATGTCACCTCCAGGCTACAAACTGGCCACCGGCGAAAGGACCAACTCTTGCCGCAATTAC
AACAAGCAGGCTAATGAGCAGAATTGGGCCAACTACAGCACAGAACAGAATCGCTTGGGC
CAGAATGGCAGCACCATCTCCAATTCACATGCACAAGCCTTCGACTACCCTGATGATACA
CATGAGCACAAGAAACTGACGCCAGGGCATGAGTTGCAGCCATTGGCGTTGATAGATGCA
CGGCCGTGCAGCCGTGCCAGCAGCCGCATGAGCAGTCGAGCGAGGCCTGATGACCTGGAC
GTCTAG

>Dr-gja1like-XM_688906
ATGGGTGACTGGAGCGCACTGGGGAAACTTCTTGACAAGGTCCAGGCGTACTCCACTGCT
GGAGGCAAAGTCTGGCTCTCCGTCCTCTTCATCTTCCGGATCCTGGTGTTGGGGACGGCG
GTGGAGTCCGCCTGGGGAGACGAGCAGTCGGCCTTCAAATGCAACACGCTGCAACCTGGA
TGTGAGAACGTGTGCTATGATAAGTCCTTCCCCATCTCCCACGTGCGCTTCTGGGTGCTG
CAGATTATATTTGTGTCCATGCCGACCCTCTTATATCTCAGCCATGTGGTGTTCCTTATG
AACAAAGAGGAGAAACTGAATAAAAAAGAGGACAAACTACGAGACATCCAAAGCAAAGGC
GGAGATGTGGACGTGCTCCTGCGCAAAATCGAAACGAGGAAGTTCAAGTACGGATTGGAG
GATCACGGGAAGATCAAGATGAGGGGAGGGATATTTTACACGTATATAGTGAGCATCGTG
TTGAAGTCCGTATTTGAAATTGTCTTCCTTTTAATACAGTGGCATCTTTACGGATTCAAG
CTGTCGGCTGTTTATACGTGCGAGAAGTTCCCTTGTCCGCATAAGGTGGACTGTTTTCTG
TCCCGTCCCACAGAGAAGACAGTTTTCATCATCTTCATGCTGGTCGTCTCGCTGGTCTCT
CTGGCTCTCAACGTATTTGAGTTTTTTATGTGATTTTTAAGAGAATGAAAGACCAAATT
AGGGAGTCTGAGAAGAAATTTGACAGTGCCTGCAATATCAAGCCCTGTCCGAGGAATCTG
TCCGGCTATGAGTATTACAATGACTGCTCGGCCCCCGTCCCAAATCTAGGCTACAATCTA
GACACTGTCGATAAATCCAACTCCTCTGATAATTACGACAAGCAGGCTAATGAGCAGAAC
TGGACTAATTACAGCACAGAACAGAACCAGTTGGGTCACAGCCAGCGCTTTCCTTACCCG
GAGAAAGTGACTCTAGGGAAGGATCTTCTGCTGCTAAAAGAGCTTGAACCTCGACCCAGT
AGTCGAGCGAGCAGTCGAGCCAGGCCGGATGATCTTGACATCTAG

>Dr-gja3-NM_207642
ATGGGTGACTGGAGCTTTCTTGGGCGGCTCTTGGAAAATGCGCAGGAACACTCGACAGTG
ATCGGCAAAGTCTGGCTGACGGTACTCTTCATTTTTAGGATTCTGGTGTTGGGAGCGGCA
GCTGAGGAGGTCTGGGGCGATGAACAGTCGGACTTCACCTGCAACACTCAGCAGCCTGGC
TGTGAGAACGTCTGCTACGATGAGGCCTTCCCCATCTCCCACATCCGCTTCTGGGTGCTC
CAGATCATCTTCGTGTCCACGCCGACGCTCATCTACCTGGGCCACGTCCTGCACATCGTT
CGTATGGAGGAGAAGCGGAAAGAGCGTGAGGAGGAGTTGCGAAAGGCCAGCCGGCTCCAG
GAGGAGAAAGAACTCCTGTATAGAAATGGAGGGGGAGGGGAGCCTGGTGGACGGGGTGGG
GGCGGCAAAAAGGAAAAGCCGCCAATCAGAGACGAGCATGGCAAAATCCGCATTAGAGGT
GCCTTGTTGCGCACCTACGTGTTCAACATCATTTTCAAGACCCTGTTTGAAGTGGGGTTC
ATTTTAGGTCAGTATTTCCTCTATGGTTTCCAGTTGCGGCCCCTGTATAAGTGTGCGCGG
TGGCCTTGCCCCAACACGGTGGACTGCTTCATTTCCCGGCCTACAGAAAAGACCATCTTC
ATCATATTTATGCTTGTGGTGGCTTGCGTGTCCCTTTTGCTGAATTTGTTAGAAATCTAT
CACCTCGGATGGAAGAAGGTCAAACAGGGCATGACCAATGAGTTTGCCCCGGACCGTGAA
TCGCTGCCTGAGGCGGACGAAGCTGAGCCCGAGTCCCCCAGAACTGCGCCTCCAACCCTC
AGCTACCCGCCAGACTACACGGAAGTGGCGGTGGCGGGTGGCGCGTTCCTCCAGCCTGTG
TCAGCGCCCTCCACCGCAGAGTTTAAGATGGACCCTTTGCGCGAGGAGCTTGAGGAGTCC
```

TCACCTTTCTACATCAGCAACAACAACAACCACAGGCTAGCTGCTGAGCAGAACTGGGCC
AACCTGGCCACCGAGCAGCAGACTCGGGAGATGAACGCCACCTCCCCCTGCTCTTCCTCC
TCATCTTGCTCCTCTGATAACGTACGGCAATCCAAAGATGCCGCTCAGCTTGCCAGCACC
CCCTCCTCTGCTGGTGGTGGTTTAAGCACTGGGCCGGAGGAGGGGCACGTCACCACCACG
GTGGAGATGCACGAGCCGCCCGTCATTTTCACTGACGCTCGACGACTGAGCAGGGCTAGT
AAAGCCAGCAGTGTGAGAGCGAGGCCCAATGATCTGGCGGTGTAG

>Dr-cx39.9-NM_212826
ATGGGAGACTTTAGCTCTCTTGGGAAGCTTTTAGAAAGCGCCCAGGAACATTCTACAGTG
GTGGGCAAAGTCTGGCTCACCGTCCTATTCATCTTCCGTATCTTGGTGCTCAGTGCTGCC
GCAGAGAAGGTTTGGGGTGACGAGCAGTCTGGCTTCACCTGTGACACCAAACAACCTGGT
TGTCAGAATGTGTGTTACGATGTAACCTTCCCCATCTCTCACATCCGATTCTGGGTGCTC
CAGATCATCTTCGTCTCAACCCCAACACTGATCTATCTGGGCCATATCCTCCACCTGGTG
CGCATGGAGCAAAAACTAAAAAGCAATGAAACAAGCGGAGCAGACAAACAAGCACTTCTG
GGCCACAAACCAAAAGGTCCCATACGTGATGAACAGGGGAAGATCTGTCTGAAGGGAGTC
TTGCTGCGCACGTATGTCTTCAACATCATCTTCAAAACACTGTTTGAGGTGGGCTTCATT
GTGGCACAGTATTTCCTCTATGGATTTGAGCTCAAGCCTCTGTACACCTGCAGCAGGTGG
CCTTGCCCAAACACTGTCAACTGCTACATCTCGAGACCGACAGAAAAAACCATCTTCATT
ATTTTCATGCTGGCCGTAGCCTGTGTCTCTCTGCTGCTCAACCTGGTGGAGATGTATCAC
CTGGGCTTTACCAAGTGCAGACAGGGTCTCCGTTACCGGCGTGCTCACTCAGTCTGCGAC
ACTGAGTCTAAAGTGCCTAGTGAGGACGTTGTTGTTCCTTTTGTGCAAAATTACCCTTAT
TTTCCTGCTCACGCACCTCCTCCGGCTTCCTTTCCCACAGAGCCGCACTTCAACCTCTCA
GAACCTGATGGGACCTTTCCGGTTCATAACAGCCGCTCTGTTTACAAGCAAAACCGAGAG
AACATGGCTGTAGAGCGCAACGGCAAACCTGACACTGCTGATGTTAAGATCAGCAAAACA
GTGAGCTCTGTACCCGGATCACCATCAAGCCAGCAGCGCAGGCCCAGCCATTCGAGTCGC
TATAGCAGCAACAAGACCAGGATGGATGACCTCAAAATCTGA

>Dr-cx39.4-NM_001044823
ATGTCCAGAGCTGACTGGGGGTTTCTGGAGCACCTGCTAGAGGAAGGCCAGGAGTACTCG
ACGGGCGTAGGACGCGTGTGGCTGACCGTCCTCTTCCTCTTCCGCATGCTTGTGCTTGGC
ACGGCTGTGGAATCTGCATGGGACGACGAGCAGTCTGACTTCGTCTGCAACACCAAACAG
CCCGGCTGTGAGTCCGTCTGCTATGACAAGGCCTTCCCCATATCCCACTTCCGTTACTTT
GTCCTTCAGGTCATCTTCGTCTCAACTCCGACCATTTTCTACTTCGGCTACGTGGCCTTG
AGGGCTAGAAATGAGAAGAGGCCAGAGGAAAAGCTGGAGGAAGATGGCAGAAGGCATAGA
CATCGAAAGACCAACGCCTGTATATTAGAAGTAATAAAAGAGGAAGACGAAGATGGGAGT
GAAACTGAAAAGAACCGCAAAGCGCTGGAACCTCCAAAGCTTAAAGGGAAACTGCTGTGC
GCTTACGCGGCGAGCATAATAGTGAAAGTTCTCATCGAAGTTGGCTTCATCCTGGGTTTA
TGGATACTTTATGGGTTTGTGATCGAGGCCAAGTACGTGTGTGAGAGGCTTCCCTGTCCT
CACACGGTTGACTGTTTCGTCTCACGACCAACAGAAAAAAACATCTTCACCATATACACA
CAAGTCATCGCCGTAGTGTCGATTCTCCTCAACGTTGTGGAGCTTTTCCATCTGCTTCAG
TTGGTGATAACACGTCGACTAGAGAAGAAATATCAGGCAGAGGTGCAGATTCATAATAGA
GTTAGAACAGCACCATCTAAAGCTCAACAACCATCATTTGAGGAAAGGAACCATCTCTTT
CTTCCTGTAGCACATGGTGGATACCCTACCGAAGGGTTGGATTGGGAAAAAAGAGACCCT
TCCTTGGCAGAAGACATGCTTCCAAGCTACTCAAACTGCATTAGGAATATGAAGCCTGCA
ATAAACAAAAACAATCTTCCTAAAAAGCACCTCAAGGCTGATGACAAACCGAGACATTAT
GTTTGA

>Dr-gja5a-NM_001007213
ATGGGGGACTGGAGTCTCTTGGGTAATTTCCTCGAAGAAGTGCAGGAGCACTCTACGTCG
GTTGGGAAGGTGTGGTTAACCGTGCTGTTCATCTTCCGTATCCTGGTGTTAGGCACAGCG
GCAGAGTCATCATGGGGCGACGAGCAGTCCGACTTTATGTGTGATACTCTACAACCTGGT
TGCACCAATGTCTGTTATGACCGAGCTTTCCCCATTGCTCATATCCGCTACTGGGTGCTG
CAGATCGTCTTCGTATCGACACCCTCCCTCATCTACATGGGCCATGCCATGCACACCGTC
CGCATGGAGGAGAAGAGAAAACAAAAGGAGCAGGAGGAGAAGGCAGAGGCGGGAAAAGGA
GAGAAGGAGTATCTGGAACATAAAGAGAAATTCGAAAATACAAAGACAAAAATCCACCTG
AAGGGGGCACTGCTGCAGACATATGTTCTGAGCATTGTGATCCGCCTGGTCATGGAAGTG
ACCTTCATTGTGATTCAGTACATGATGTACGGGATCTTCCTGGATGCTCTGTATCCATGT
TCAATGCTTCCCTGCCCCAACCCTGTGAACTGTTACATGTCCCGTCCAACTGAGAAAAAT
GTCTTTATTGTGTTCATGCTGGTGGTTGCAGCTGTTTCGCTCCTCCTCAGCGTCATAGAG
TTATATCACCTTGGATGGAAACAGTGCAAAAAATGCCTTAGGAAACATGCTGACAAGCAT
GCCAATGACAAAATTCAAAACGTCAAAGCTGTTTCTGCAATTGAACCGATCAGGACAAGC
ATTCCAATGGATCTGGCTGAGAACATCCAGCCTCGTCTTTCTCAAACCTGCACTCCACCT
CCAGATTTCAACCAGTGCTTAAGATCAAACCAAGGTCCAACATCTCCTCCACATCTTCAT
TCTCACCATCTTCATCACATCCACCAAACCTGCCAGCCCTTCACCAACCATCTGGCACAC
CAGCAGAACTCCGTCAACATGGCCGCCGAGCGGCATCACCACAGCCATGATGGCCTGGAG
CCAGCCGTGGACTTCCTGCAGATGCATTATGGGAGTCCTGAGGCTCGGGTTCGAAGTGAA
ATGACACCCAGTACCCCTTCCACACCATCCTCCCATCCAGGGTTCTTCAGAGACAAGCGC
CGGCTTAGCAAGACCAGTGGTACTAGTAGTAACCGACTCAGACCAAGTGATCTGGCCGTG

TAG

>Dr-gja5b-NM_001034988
ATGGCCGACTGGAGTCTGCTGGGGAGCTTTCTAGAAGAAGTCCAGGAGCATTCAACCTCG
GTGGGAAAGGTGTGGCTCACTATTCTTTTCATCTTCCGGATCTTGGTACTAGGCACGGCT
GCTGAATCCTCGTGGGGCGACGAGCAGGAAGACTTCACCTGCGACACAGAGCAGCCCGGC
TGCGAGAACGTTTGTTACGACCGAGCCTTTCCTATAGCGCATATACGCTTCTGGGTGCTC
CAGATCGTGTTCGTGTCTACACCTTCTCTGATCTACATGGGGCACGCAATGCATATCGTC
CGCCGAGAGGAGAAGAAGAGGAAAGAGCTGGATGATGAAGGAGCGCAGAGAGATGGAGAA
AAGTACCCAGAAGATGACAAGAACAAGGAGGACGAAGGTGGAGGTAGGAGGGTACGATTG
AAGGGTGCGTTGCTGCAAACATACGTCCTCAGCATCCTCATCCGCACTGTGATGGAAGTG
ATCTTCATCATAATCCAGTACCTGATCTACGGAGTCTTCCTTAGTGCACTCTATGTGTGT
AAAGCCCCTCCGTGTCCACATCCGGTCAACTGCTACATCTCCAGACCAACAGAGAAGAAC
GTGTTCATTGTCTTCATGCTAGCAGTAGCAGCGGTGTCACTGCTGCTTAGTATCGTGGAA
CTGTATCATTTGGCATGGAAGCAGTTGAGGAAGTATGTGCACGGATACAAGGCTTCCAAA
CAACGACCAAACACGCCGTCCACCATGCCTGCACTTTCACCAAATCCGTCCACCCCAAAC
CGAGCCTGCACCCCACCTCCAGACTTCAACCAATGCTTGACCTCGCCACCATCTTCTCCT
ACTTTACAGACACACTCGCTTTTACATCCGACCTGCCCTCCATTTCACGACCGACTGGCG
CACCAGCAGAACTCTGCAAACATGGTCACTGAAAGGCACAGAGGACAAGACTACTTAGGG
GTCAACTTCTTGAGCTTCTCACAGACACCTACAGAGACTCCCAACTCCTGTGCCTCACCT
TCATTCCTGAGCAGTGATTTTGAGGACAAGCGAAGGTTTAGTAAGAGCAGCGGGACCAGC
AGCCGCATGAGACCGGACGACCTTGCGGTATAG

>Dr-gja8b-NM_131809
ATGGGTGACTGGAGTTTCTTGGGCAACATCCTCGAGGAAGTAAATGAGCATTCGACGGTA
ATCGGTAGGGTGTGGCTCACGGTCCTCTTCATCTTCCGAATCCTCATCTTGGGCACAGCC
GCAGAGTTTGTGTGGGGCGACGAGCAATCGGATTATGTGTGCAACACGCAGCAGCCGGGT
TGCGAGAACGTTTGCTACGACGAGGCCTTTCCCATCTCGCACATTCGCCTCTGGGTGCTC
CAGATCATCTTTGTTTCCACACCCTCATTAGTGTACGTGGGCCACGCCGTGCACCATGTA
CACATGGAGGAGAAGCGTAAGGAACGGGAGGAGGCTGAGCTCAACCGGCAGCAAGAGAAC
GAGGAGAGGCTGCCGCTGGCGCCTGATCAGGGAAGCGTCCGTACGGCCAAGGAGACGAGC
ACAAAGGGCAGCAAGAAGTTTCGTCTGGAGGGCACTCTCCTGAGGACCTATATCTGCCAC
ATTATCTTCAAGACTCTCTTTGAGATCGGCTTTGTGGTGGGTCAATACTACTTGTACGGC
TTCCGAATCTTGCCACTCTACAAGTGCAGCCGTTGGCCGTGCCCAAACACGGTTGACTGC
TTCGTCTCAAGACCAACCGAGAAAACCGTTTTCATCATCTTCATGTTAGCTGTGGCCTGC
GTCTCACTGTTCCTCAATTTTGTGGAAATCAGCCATTTAGGCTTAAAAAAGATCCACTTT
GTGTTTCGTAAGCCGGTGCGGCCGCAAGTCGAGGGACCAGGAGCAGCCGAAAAGGCATTG
CCTTCCATAGCTGCCTCATCGATCCAGAAAGCCAAAGGTTACAAGTTGTTGGAGGAGGAC
AAATCCACGTCGCACTTCTTTCCTCTGACTGAGGTTGGGGGGATGGAGGCTGGACGCCTG
CCGGCTTCATATGAGCCATTTGAGGAGAAATCTGACGAGGCCATGGCACCTAAGAAAGAC
ATGTCTAAGATGTATGACGAAACGCTGCCCTCTTACGCCCAGACGACCGTGATTGGACCG
AGTGCATCGTCAGGAATTCTGCGCAGGGATGAAGATGAGGACGAGTTGGCTGTGGAGGCA
GACATGGAGGCCAGCGAGACGATAGAAGATACACGACCGCTCAGCAGCCTGAGCAAGGCC
AGCAGTCGCGCAAGGTCAGATGACTTGACGGTATAA

>Dr-gja8a-NM_001128350
ATGGGAGACTGGAGCTTCTTGGGTAATATTTTGGAGGAAGTAAATGAACATTCAACTGTG
ATTGGTCGTGTTTGGCTCACAGTACTATTCATTTTCCGAATTTTAATTCTGGGCACGGCT
GCTGAGTTTGTCTGGGGAGACGAGCAGTCCGATTACGTGTGCAACACTCAGCAGCCAGGA
TGCGAGAATGTGTGCTATGATGAGGCCTTTCCAATCTCTCACATTCGCCTATGGGTGTTA
CAGATCATCTTTGTATCCACACCTTCACTTGTATACGTGGGCCATGCTGTCCATTACATC
CACATGGAGGAGAAACGAAAGGAGCGGGAAGAAGCTGAGGTCAGCCACCAGCAAGAACTT
TGCGAGGAGCGCCAGGCAATGGATCAAGGAAGCGTTCGCACTGCCAAGGAGACCAGCACG
AAGGGGAGCAAGAAGTTTAGACTTGAAGGAACCCTGCTGTGCACCTACATCTGCCATATC
ATCTTCAAAGCTCTGTTTGAAATAGGCTTTGTAGTGGGACAATATTTCCTTTATGGGTTC
CGCATCCTGCCTTTGTACAAGTGCAGTCGTTGGCCATGCCCTAACACAGTGGACTGCTAC
GTCTCCCGGCCCACCGAAAAGACCATCTTCATCATTTTCATGCTTGCAGTGGCTTGTGTT
TCACTGTTCCTCAACTTTGTGGAGATTAGCCACCTCGGCTTGAAGAAGATCCGCTTTGTG
TTCCACCGACCAGCTCCAGCACAGTTAGAGTCGCTTGGACCTCCTGAGAGGAGTTTACCG
TTTCTTCTAACCACCCCTGTCCAAAAAGCCAAAGGCTACAGGCGCCTCGAAGAGGAGAAG
AAAGACGAGGTGGCTCATATCTATCCACTAGCTGAGGTTGGGATGGAAGAGGGACAGTTC
TTCTTACCTCAGCTGGAGAAGGAGCAGAAAAGCAGTCAGGAGGCAATTCTGCCAACAGCG
CCACCTGTAGAGGAGACAATTATATGCGATGAGACTCAACCCTCCTTCCTTCAGGTCACA
GAGACATTACCAGAGCTCCCAACTGAAGAGCCGCCTAGGGAAGGAGATGAGGTAGACAGT
CTAAAAACTCCAACAGTGCTCCCAGAAGTACTAGAGGAGCATTCAGAAGGGGAAAGCGTG
GAGGAAACATATTTAATTTCACTTGAGGAGAATTTGGATGTAGATGTAGGGAAAGTGGTG
ACTGAAGAAAAGTTGCTAAGAGAGAGTAGCTTAGTAGATACTGAGCCAAAACAAGAGGAA
AACCTTTCAGGAGATGGAGAGGAGAAGGAAACCTCACAGGAAATTGGGGAAACAGAGGAT

AGCAACTTTCCAACCAAAAGGCTGAAGGAGGAAGAGGGTTTACCTGATGTGGTTGAAGAA
GCATCTGATGAAGAGAGAGAACTTTGTGAGCTTGAGCCTTTTGTGAATGAGGACACTGTG
GAGGAGGTGAAAACTTTAGATGATGTGAATCCTGACGATCTGGATATGTCCAAAATATCT
GAAGGAGAACAAGAGTCTGAGGCTTTAGCGGAAAATGTAGCATCAAATCTTCCTGACATA
GCGCCTATTGGGGATGAAGTGGATATGGGAAGGGACAAAGCTCAGAGTGATGTAGATGAT
AAAGAAGATGGAGTTCAAAGTGATGTAGTAGACTCAGGAGTTTTAGAAGATTTAGTGGAG
ATTAAAAAGGTCAGAGCTCTAGATGATCTACAAGATGCAGAAGATTGTTGTCTAGATATA
GATAAGAACGATCCTTTAGAAAATGAGGATATACCTCTTGACACTGCAGTCAATACAAGT
GGAGAGGATCTAGATAAACCTCTAGGACTTTCAGGTGACACTAACCAGTTGAAAACTTTG
AGGAAGGAAGAGGTATCATTGGGGATAGAAGATCCACAGGAGGTCAAAGGCTCTGAGTTG
GAAGAGTCAAGACAAGAAGATGAGTCTGTGAAAACTGGAGACCTTGAGAAGGAAGAATAT
TCTGAAGAATCAAAAGCCTTGGAGGCTTTAGAGGAGATGATAGATGCTCCACTTGTAGCT
CTAGATTTAGAACCAACAGAGGAAACAAGATCTTCAAGTCGTCTCAGCAAAGGTAGCAGC
AGAGCCAGGTCAGATGATCTAACTATATGA

>Dr-cx55.5-XM_021466745
ATGGGAGACTGGAACTTTCTTGGTGGGATTTTGGAAGAAGTGCATATCCACTCTACCATG
GTGGGAAAAATCTGGCTCACAATCCTCTTCATATTTCGCATGCTGGTGCTTGGTGTGGCA
GCGGAGGATGTGTGGAATGATGAACAGGCTGACTTTATCTGCAACACCGAGCAGCCTGGT
TGCCGCAATGTGTGCTATGATAAAGCTTTCCCCATTTCCCTAATCCGTTACTGGGTTCTG
CAGGTCATATTTGTGTCCTCTCCCTCACTGGTGTACATGGGCCATGCCCTCTACCGCCTC
CGTGCTCTTGAGAAGGAGCGTCAGCGCAAAAAGATGGCACTGAGACGAGAACTTGAGGGT
GTGGATGTGGAGATGGCGGAGGTACGGCGCAAAATAGAACGTGAGCTTCGGCAGATCGAC
CAGGGGAAATTAAACAAGGCTCCATTGAGGGGGTCTCTTCTGCGCACATACGTGGCTCAC
ATAGTCACCCGCTCAGCTGTAGAAGTATTCTTCATGACAGGACAATATGTTCTGTATGGG
TTTCAACTGAATACACTGTACAAATGTGAACGGGAGCCTTGTCCCAATGCAGTGGATTGC
TTTGTATCTCGACCCACTGAGAAAAGCGTCTTCATGGTGTTCATGCAATGCATAGCTGGC
ATTTCATTGTTCCTCAACATTCTGGAGATCCTGCACTTGGGGTACAAGAAACTTAAAAAG
GTCATTCTGAACTACTATGCACAGCTGAGGGATGATCCCAATGACAGCTACTATCCCAAC
AAAGTGAAGAAAGATTCTGTTGTGCATCAGACATGCATTGGCACCTCCACTGGCCGCAAG
GCCACCATTGCTTCTGCACCCAGTGGATACAACCTTCATCTTGATCGACCACCTGATGGA
GCTGCCTATCCTCCTTTGATTAACCCATCCTCTGCTTTCTTGCCTGTTCAGGGTGATTTA
CCAGCTAAAAACGGTGCTGATGAACCAAAGTACTTGCAGAACAGTCCCACAGAGCACAAC
AGCAATTCAAACAATACCAGCAGTGACTCGCACTCACCACCTTGCAACTCTGTCACTCCA
CCCAAGCAAGACGAAGGGGAAGATTCTGTCCAAACTTTACCACTGCACAAGAAAGGGCAG
GAGTCTAAGTTATCAGAGTCATCGAGCCACACCAGAGAATCATCTCATGCCTCATCCAGC
ATGGTAAAGAAACCTTGGAAGGGTAGTGCTCCCTGGAATTGCTCAACAGTCGTAGAAGGT
AATGGCTCAGACTCAGATTCCCTGGAAGGCTCCAAAGCTCGTTGTCCTTATTCTGCTGTG
CGGGCACGTACCTCATCCAGGTCTGATACCAAGCTGAGCAGGCCCACCTCCCCTGATTCA
GTCGAAGAATCGAGCTCTGAGTCACGGCATAGTCCACGAGCGTCACCAAGCCATCGTGCC
TCATTGGCCAGCAGTTCCAGCAGCAGACGAGCAGCTCCCACAGACTTACAAATTTAA

>Dr-cx52.9-NM_207093
ATGGGGGACTGGAACTTCCTGGGGGGGATTCTGGAGGAGGTGCACATCCACTCCACTATG
GTGGGAAAGATCTGGCTCACCATCCTCTTCATCTTCCGCATGCTGGTGCTGGGCGTTGCG
GCGGAGGACGTGTGGAACGATGAGCAGTCCGACTTCATCTGCAACACTGAGCAGCCCGGC
TGCCGCAACGTCTGCTATGACCAGGCCTTCCCAGTGTCCCTGATCCGCTACTGGGTGCTG
CAGGTTATCTTCGTGTCCTCACCCTCGCTGGTCTACATGGGCCATGCCATCTACAAGCTG
CGCGCCCTGGAGAAGCAGCGCCACTGCCAGCGTGTGACCCTGCGGCGGGAGCTGGAGACG
GTGGAGCCGGAGCTGATGGAGACGCGGCGGCGCATCGAACGGGAGCTGCGGCAGCTGGAG
CAGGGCAAGCTGAACAAGGCTCCGCTGCGGGGCTCGCTGCTGCGCACATATCTGGCCCAC
GTGCTGACCCGCTGCGTGCTGGAGGTCTGCTTCATGATGTGCCAGTATCTGCTATATGGA
CACCGGCTGCAGCCGCTCTACAAGTGCGACCGGCAGCCGTGCCCAAACGTGGTGGACTGC
TTTGTGTCTCGGCCCACGGAGAAAGCCTGTTCATGGTGTTCATGCAGGGCATCGCGGCG
GTGTCGCTGTTCCTGAGCCTGCTGGAGCTCCTGCATCTGGCCTATAAGAAGCTGAAAAAG
GGGCTGCGGGACCACTGCCCTGCCCTCAGAGACGGGCCAGCGGACTCCAGTGCCCCCAAT
AGGAACTCTGTGGGACAGCAGGCTCGCAAGGCCACCATGCCCATGGCACTCAGTGGGTAC
ACCGCACTGCTGGAGAAACAGGGCAATGGCCCCACGTACCCCTCAATCATACACCCGCTG
TCTGCCTTTGTGCCGATCCAGGGCCCGTCAGCACCAGACATGGAAAACCGGGATGCCCTG
CGCAGCCCGCTGGAACACAACAGCTCCAACAACACCAGCAGCGGGTCCCGGTCTCCATTG
GTGCAGGAGCGTGGTCCAGCTGCGTCCCCCAGCGACTACCACACGCTCCCCCAGACAGAC
TCCTTGCCCCTGATAGCCACACTCCCCCTGACGGACCCCCTGCCCCAGATGGATTTGTTC
CCCTTGAGGGATGCAAGCTCTTGTCCAGCTGTCCTGCACAAGCAGCGGCGGGTCAGTCCG
CCCTGGAACTGCAGCACTGTGATGGAGAGCACCGGCTCGGACAGCGGAGACTCCAGCAGT
GGTGGGCTCAGTCAGGGAAGGAGGACTCGCTGTGGCCGGTCCGTGTCCCGCTCAGACCTG
CGGCTCACGCCGGACTCACAGCCTCACTGTGCTGCAGAAAGCCCCTCACTCTCACCCCAG
CGGCAGCGGCCAGCGATAGGCAGCAGCAGACGGGCAGACCTGCAGATCTGA

>Dr-cx52.6-NM_212819
ATGGGAGATTGGAACTTGCTTGGGAGTATCTTAGAGGAGGTTCACATTCACTCCACCATT
GTGGGCAAAATCTGGCTTACCATTCTTTTCATTTTCCGCATGCTCGTACTTGGGGTTGCT
GCGGAAGACGTATGGGATGATGAGCAAAGTGAGTTTGTGTGCAACACAGAACAGCCTGGA
TGCAAGAATGTCTGTTATGACCAAGCGTTCCCGATATCCCTCATCCGATACTGGGTCTTG
CAGATCATCTTTGTCTCTTCGCCATCTTTGGTGTACATGGGACACGCACTCTACCGGCTC
CGAGCTCTGGAGAAAGAGCGGCACAAGAAGAAAGTCCAGCTGAAGGTGGAGCTGGAAGAG
AGTGAGGCTTTGGAAGAACACAAAAGAATTGAAAAAGAGCTTCGGAAGCTGGAGGAGCAG
AAGAAAGTGAGGAAGGCCCCTCTGAGGGGTTCCTTGCTGCGCACATATGTGTTCCATATC
TTAACCAGGTCAGTGGTGGAGGTGGGATTCATAGTGGGGCAGTACATGCTCTACGGTATC
GGACTGACGCCGCTGTACAAGTGTGAGCGTGATCCTTGCCCCAACAGTGTAGACTGCTTT
GTTTCTCGGCCGACAGAGAAGAACATCTTCATGATTTTCATGCTGGTCATATCAGGAGTG
TCCTTGTTCCTCAATCTCCTGGAGATTTTCCACCTTGGTGTGAAAAAGATCAAGCAGACC
ATATATGGATCCATGTACAGCGACGACGACAGCATTTGCAGGTCAAAGAAAAACTCCATG
GTCCAACAAGTGTGCTTCCTTACAAATTCCTCACCACAAAAACAGTTGCATTTGACACAC
ACTTCCCTTGCCATGGCACCTGATGGACAGATGGTACCTTTGCCTATCTATATGCAGACA
GCTGGTCATGTAGTGTCCAACATTAACCCAAATGGATCTGTGCAGCCTCTGAGACAGGAC
CGTCTTCCAAGCCAACCCGAAATTCAAGTTCTTCAACAACTGGGAATCAAAGAACGAAGG
TCCATTCCAGACAACCGTCTGCAATCCTGCAGCAGTGAGGATTCTGGCCCCAAAGGTTCC
GAACCACCGAAATATTCTCAGCAGCCCCGAGCTTCATTCAGAGCCAGTCATATAGAAATA
CCAGCAGCATTAAGGAAACACAGCCGTGTTAGTCAGTGTAAAGACTTCAGTGAGGAGAGT
GATTCGGTGGAAAGTGGGAACTATCCTACTGCCAGGAAAGCCAGCTTCATGTCAAGGGGA
CTTTCAGAGAGCCCGTCTGAGAGTGCAGCCTCCAAGAGTGGATCAGACACAGAGGCCAAC
CGTATCACTCAAGGGGAGAGTCCCGCTATGACACCACCCCCTGCAGCAGGACGTAGAATG
TCAATGGTAAGAAAATTCTGA

>Dr-cx52.7-XM_021467222
ATGGGGGACTGGAACTTGTTGGGGAGCATTTTAGAGGAGGTTCACATTCACTCCACCATC
GTGGGTAAAATCTGGCTGACCATCCTGTTCATTTTCCGGATGTTGGTTCTTGGTGTTGCG
GCAGAGGACGTTTGGGTGGACGAGCAGAGCGAGTTCGTCTGCAACACGGACCAGCCTGGA
TGCAAGAACGTCTGCTACGACCAGGCATTCCCAATATCGCTCATTCGCTTCTGGGTATTG
CAGATCATTTTTGTTTCCTCGCCTTCGCTGGTGTACATGGGACATGCTCTGTACCAGCTA
AGGTCTTTAGAGAAAGAACGGCACAGGAGGAAAATCCAGCTGCGAGCAGAGCTGGAAGAG
ACCGAGCCCCTCTTGGAGGAGCACAGAAAGTTGGAGAAGGAACTGAGGAGGTTGGAAGAG
CAGAAGAAGATGAAGAAGGCTCCTCTAAGGGGCTCCTTGCTTCGTACATATATTATTCAT
ATCCTCACCAGATCAGTGGTGGAAGTGGCGTTCATTGTCGGGCAGTATATCTTATATGGC
ATTGGACTGGATCCTTTGTACAAGTGTGAGAGGGTGCCTTGCCCGAACAGCGTGGACTGT
TATGTTTCCAGGCCAACAGAGAAAACCATCTTCATGGTTTTCATGATCGTCATCGCAGGA
GTTTCGTTGTTCCTGAACCTTTTGGAAATATCCCACTTGGGGGTGAGAAAGATTAAACAG
ACTCTGAGCGGACTGCAGTTTGTCGAGGAGGACAGTCTTTGCAAACCCAAGCATTCGACA
ATTCAGCAGCTCTGCGTGATGACGGAGTATTCGCCTCACAAAAACCCACAATTGAAAACG
TTTATCCCGCAGGGACAAATGGACAAACATCTGTTCAGCTCTTCGAGCAATGATATTCTG
CGGCACAACAGTTTAGCAGCGTCCACCCTACCGGTGTCCTGCATTACCCAGCAGCCTCGC
CAAATGCGCCAGCCCAGCCAGGGAATGATTCATGAACTGCACTCCCAGGGGTCACTGAGG
CTCCTGGAGGACCAGGAAAACCAGCATCCAGATAGCAGTAACTGCTCAGAAAGGGACATT
AGGCCTTTTAACTCGGGCCATCCGGGTTCTGAGGGCCATACCGAGATACCAGCCTGCCTT
CGCAATGCTCTGCACAGGCCCAGCCGCTTGGCGGACCTAGCAGATGATGCTATGGAGTCC
TCCGAGAGCGACTTCTGTCCACCCAACAGGAAAGCCAGTTTCATGGTTCGGATGCCCTCT
GAAAGCATGTCCGGCAGTCCGTCCTGTCCCTCCACCAGGAGTTCAGAGTCTGAGCTGGGA
TCCCTTAACGACCTGCCCATGAACCCACCACCAGGGGGAGGACGACGGATGTCTATGGCA
AGTAGATGGAAATGA

>Dr-cx34.5-NM_001030200
ATGGGCGAGTGGGATTTCCTCGGACGACTGCTGGATAGAGTCCAGACACACTCCACCGTA
GTGGGAAAAATCTGGCTCACCGTCCTGTTTGTCTTCAGGATTCTAGTCCTTGGAGCCGGC
GCTGAGAGAGTGTGGGGCGATGAGCAGTCCGACTTCATCTGCAACACAGAGCAACCGGGA
TGCGAAAACGTCTGCTACGACCGCGCGTTCCCAATCTCGCACGTCCGCTACTGGGTGCTT
CAGATCATCTCTGTGTCCACGCCAACTCTGGCCTACCTGGGCCACGTCGTCCACGTCATA
CACGCCGAAAAGAAAGTGCGAGAGATGATGAAGAAAGAGCTTCAGAATGAGCAAATCAAC
CTCTTCCTCAAGAAAGGCTACAAAGTTCCCAAGTACAGCCGGGAAAACGGGAAGGTCAAC
ATCCGTGGACGTCTTCTGAGAAGCTACATTCTGAGTTTGCTTTGTAAGATACTACTGGAG
GTGGGTTTCATTTTGGGCCAATACTATCTTTATGGCTTCACTCTTAGGGCTCAATATGTC
TGCAGCTATTTCCCGTGTCCTCACAAGGTGGACTGTTTTTTGTCGAGGCCCACTGAGAAA
ACCATCTTCATTTGGTTTATGCTGGTGGTGGCTTGCATTTCCTTGCTCCTAAATGTGATT
GAGATCTTATATCTTTGCGCTAAGAAGATCAGCGAGTGTCTCAGCCGCAAAAAGGACTAC
ACCATCACTCCGGTGACCCCTGTGGTGAGCAAAAAGAACTTTAAAAATACAGATCAGGTG
ATACAGAATTGGATGAACCGCGAGTTGGAGCTTCAGAGGAGGGAACCTGGCAATGAGGCG
ACCAAGAGCTTGGCTTCAGAGGGTCGCAGTGCTGACATGCAAGAGGTTCATATCTGA

```
>Dr-cx32.2-NM_001030210
ATGGGAGACTGGGGGTTTCTCTCAGCCTTACTGGACAAAGTACAGTCTCACTCCACTGTC
ATCGGGAAGATATGGATGAGCGTCCTATTCATCTTCCGCATCTTGGTGTTGGGAGCAGGA
GCCGAGAATGTTTGGGGCGACGAAAGATCCAACTTAGTGTGCAACACCAACACCCCTGGC
TGCGATAACCTGTGCTACGACTGGCAGTTCCCCATTTCGCACATCCGCTTCTGGGTCATG
CAAATCATCTTCATTTCCACTCCAACTTTAGTGTATCTGGGGCACGTGGTGCACATCATC
CACCAGGAGAACAAACAGAGAGAACTTCTCAAAAGCAATCCCATGGCAAAGTCGCCGAAA
TACACTGACGAAAACGGAAAGGTCGAAATTAAAGGAAGTATGTTGGGTAGCTACTTGACG
CAACTGTTCATTAAGATCATTTTAGAGGTGGCCTTCATCGTCGGACAGTATTATCTGTTT
GGATTCATCATTGACCACAAGTTCATCTGTGAAAGGTCACCCTGTATGAGGGCTGAGTGT
TTCGTGTCCAGACCCACGGAGAAAAGCATCTTCATTATCTTCATGCTGGTGGTGGCTTGC
GTGTCTCTGGCCTTAAATGTTCTGGAGATATTTTATTTGCTTTGTAGGAGGATCAGTCGG
AGAAGTAAGAAGTGTAGACAAGCAATGTATAATGGTGAATCTCGTTATCCGGGACATTTC
ACAACAGAACTCGAGTCTATGAATGGGATGAGGCATAATGAGTTTAATGTGGCCTTTCAG
AACAAGTGGAGTCAAAGAAAAGGCAGTCTGGACGCAGCCAAACCTGAGGCTTAA
```

```
>Dr-cx32.3-NM_199612
ATGGGAGACTGGGGATTTCTCTCATCGTTATTAGACAAAGTACAGTCTCACTCCACCGTT
GTTGGCAAAATATGGATGAGCGTGCTTTTCATCTTTCGGATCCTGGTGTTGGGAGCAGCG
GCGGAGAGCGTTTGGGGTGACGAACAATCAAGTTTGGTTTGCAACACCTTGCAACCTGGT
TGTGAAAACGTGTGCTACGACTGGCAGTTCCCCATCTCACACATCCGCTTCTGGGTCCTG
CAGATCATATTTGTCTCCACTCCGACTTTGGTGTACCTCGGCCATGCGGTGCAGGTCATT
CACAATGAGAACAAACTTAGGGAGAAGAAAAAAATCCTTGGTGATGGCCACATGTTGAAG
GAACCCAAATACACCGACAGCCAAGGCCACGTCAAGATTAAAGGAAACCTCCTCGGCAGC
TATCTAACGCAGTTGTTTTTAAAATCATCCTTGAAATCGCGTTCATTGTTGGACAGTAT
TATTTATACGGCTTTATTATGGTCGCCAAGTTTACATGCTCCCGTTCCCCTTGCCCTTAC
ACTGTTGAATGTTTCATGTCCCGTCCCACCGAAAAGACCATCTTCATTATATTTATGCTA
GCGGTGGCCTGCGTATCTCTGTTACTGAATGTCATAGAGGTGTTTTACCTGCTGTTCACC
AGAGTGGGATGTCGGAAGAGACGATCACATACTGTTACTACGGCTAAAAACCCGGCCAGT
TTGTCTTCCTCCTGGCAGATGAACTCTGAAGACGCTCTGAAGCAAAACAAACTCAACAAG
CAGTTTGAGAGCGGACAGAGCCTTGGAGGAAGCCTGGATGGGGCGAAAGAAGACATGCAA
TTGATGGAAGATCACTAG
```

```
>Dr-cx28.9-NM_001007324
ATGGGAGAATGGGGATTTCTCTCCAAGCTGCTGGACAAAGTGCAGTCTCACTCCACAGTG
GTTGGGAAGGTGTGGCTCACGGTCCTGTTTGTCTTCAGGATCATGGTTCTCGGGGATGGT
GCTGAAAAGGTGTGGAGCGACGAACAATCAAAAATGATCTGCAACACGAAACAGCCTGGT
TGCACGAACGTATGCTACGATCACACCTTTCCCATCTCCCATATTCGCTTCTGGGTTCTT
CAAATCATCTTCGTGTCCACGCCAACACTTCTATACTTCGGCCACGTCCTGCATGTCCTC
CACAAAGAAAAGAAACTGCGACACGAGATCGAATCCCATGCTGAAAAACAAGGCCTCAAA
CAGCCGAAATATATAGACGATTACGGCAAAGTCATAATCAAGGGCCAATTATTGGGTAGT
TACCTATCCAGCCTGTTTGTGAAGATCTTGCTAGAGGCCGCGTTTATCGTTGGCCAGTAT
TATATTTACGGTTTCATAATGATCCCGAAGATCGAATGCTCCCAGTCTCCTTGCCCTCAT
ACAGTTGAGTGCTACATGTCCCGTCCCACAGAGAAGACCATCTTCATCATCTTCATGCTG
GTGGTGGCGTGCATCTCTCTGCTTCTGAACGTGGTTGAGATGTTCTACCTGATATGCCGC
AGGTCAAAGAGACACCGCGCCGCAAAGATGACTTCATTTCATAAAGGTTTAAACGGATCC
AAGGTGTACATTTCAGGAACTTCAAAGTCTAGCAAATCTTAA
```

```
>Dr-cx28.1-XM_005170194
ATGGGCGACTGGGGATTCCTCTCCAAACTTTTGGACAAAGTGCAGTCTCACTCGACCAGC
ATTGGAAAGGTTTGGCTGACAGTTCTGCTGATCTTCAGAATAATGGTTCTAGGTGCCGGA
CTGGATAAAGTCTGGGGAGACGAACAGTCCAGAATGGTCTGCAACATCAACACTCCTGGT
TGCCTGAACGCCTGTTACGACCACATCTTCCCCATATCTCACATGCGATTCTGGGTGCTC
CAAATCATCTTCGTGGCCACTCCGAATCTGGTCTACCTCTTTTATGTTCTGCATGTCATC
CATAGAGAAAACAAACTGAGGCAGCGTTTAGAAAATCAGGCAGAGAAGCACGGTGTCAAG
CTACCGAAATACACAGACGGCAATGGGAAGGTTTATTATAAAGGGAACCTTCTCGGTTGT
TATATGTTTAGCCTCATTGTGACTATTTTGTTGGAGGCTGGCTTTCTTGTAGGCCAGTAT
TTTTTAATTGGCCTTTTGATGCCCATGCAGCTTGACTGTAATGTAGAGCCATGTCCTAGT
GTTGGTCTGCATTGTTTTACGTCCCGTCCAACTGAAAAGAGCATCTTCATTGTGTTCATG
CTCATTGTGGCTTGCGTGTCTTTAGCTCTGAATATTGGAGAGATTTTTTATCTGATTGGT
CGCAGGAATGTGTATAAAGCAAGGACTCGTTCGAATGCTGTGGATGAGATGCACAAATTG
AGCCCCACTGAAACGTTTTGCTGA
```

```
>Dr-cx27.5-NM_131811
ATGAACTGGGCGTCATTTTATGCCGTGATCAGCGGCGTGAACCGACATTCCACCGGCATT
GGGCGGATTTGGCTGTCTGTCCTCTTCATTTTCCGGATCCTGGTTCTGGTGGTGGCGGCG
GAGAGCGTGTGGGGCGACGAGAAAGCGCATTTCATCTGCAACACCCAACAGCCGGGATGC
```

AACAGCGTGTGCTACGACCACTTCTTCCCGATCTCCCACATCCGACTGTGGGCCCTGCAG
CTCATCATGGTCTCCACCCCCGCCCTGCTGGTCGCCATGCACATTGCACACCGTCGGCAC
ATCGACAAAAAGTTGTATCGCCAGGCTGGCCGCACCAGCCCGAAAGACTTGGAGGCGATA
AAGAACCAGAAGATGAAGATTACCGGCGCCCTCTGGTGGACATATATGATCAGCCTGCTG
TTCCGTGTGTTGTTCGAGTCCGCCTTTATGTATCTGTTTTACATGATTTACCCGGCTAT
AAGATGTTCCGGCTGGTGAAGTGTGACTCGTATCCGTGCCCAAACATTGTCGACTGTTTC
GTGTCCAGGCCGACAGAGAAAACAGTCTTCACTATATTTATGCTGGCGGTGTCCGGCGTC
TGTATCCTGCTCAACATCGCCGAAATCGTCTTTCTTGTGGCGAGAGCAACCAGTCGACAT
CTCAATAACTCCAAAGATTCCGCTGTGGGAGCCTGGATCTCCCAAAAACTCTGCTCCTTC
TAG

>Dr-cx31.7-XM_001921588
ATGAATTGGGCATCCTTTTATGCTGTGATCAGTGGTGTGAACAGGCATTCAACAGGCATT
GGACGCATCTGGCTGTCAGTCATCTTCATCTTCCGTATCTTGGTGCTAGTAGTGGCAGCC
GAGAGCGTGTGGGGAGATGAAAAGTCAGGCTTTACCTGCAATACTCAGCAACCCGGCTGC
AACAGCGTGTGTTATGACCAGTTCTTTCCAATCTCACACATCCGCCTTTGGATTTTGCAG
CTCATTCTGGTGTCCACACCAGCCCTACTGGTCACTATGCATGTTGCACATCGGCGACAC
GTTGAGAAAAAGATCCTCAAGATATCTGGTCAGGGAACTGAAAAGGACTTCGAGAGCATT
AAAACCCGAAAGTTCAAAATTGTTGGTGCACTATGGTGGACTTACATGATAAGTATCATA
TTTCGCATAATTTTTGAAGTGGTTTTCTTGTACATTTTCTACTTAATCTATCCAGATATC
ACTATGGTTCGTCTTGTGAAATGTGACTCATATCCATGTCCAAATACAGTAGACTGTTTT
GTGTCTCGTCCTACAGAGAAGACCATTTTTACTGTCTTTATGCTGGTGGTGTCTGGACTT
TGTGTCTTGCTAAATATCACAGAGGTTATGTATTTAATAACTCGGGCATGTATCAAATAT
TTTCAAGGAGCAGTACATCAAACTAAAGGACCTTGGCTCACTCATAAACTGGGAACCTAT
AAGCAGAATGAAATAAATAATTTGATATCAGAGCATTCATTTAAACCTAGATTTAATGTT
GGGCGGAAACCTCCAGTGCTGAAAAATGAGCGCTGCTCAGCTTTCTAG

>Dr-cx30.3-NM_212825 (a cx30.3*1 sequence)
ATGAGTTGGGGAGCACTTTATGCTCAGCTGGGAGGAGTGAATAAACACTCCACCAGCTTG
GGGAAGATCTGGCTGTCTGTCCTCTTCATCTTCCGCATTTGCATCCTGGTCATAGCAGCA
GAGACGGTCTGGGGAGACGAACAGTCAGACTTCACCTGCAACACACAACAGCCTGGTTGC
AAAAACGTTTGCTATGACCACTTCTTTCCAGTCTCGCACATACGTTTCTGGTGTCTGCAG
CTCATCTTTGTGTCCACACCGGCTTTACTGGTGGCTATGCATGTGGCATATCGCAAGCGC
AACATGAAAAAGAAAAGCATTTTAGCCAAGCGTGGAGGTAATGGTAAAGGAGATGACCTG
GAGAGCTTGAAGAACCGGCGTCTACCCATCACTGGGCCACTGTGGTGGACCTACACATCC
AGCCTGTTCTTCAGACTTCTTTTCGAGGCCGGATTCATGTATGCTCTCTATTACGTCTAT
GATGGCTTTCAGATGGCACGCCTTGTGAAGTGTGAGCAATGGCCTTGTCCCAATAAAGTT
GACTGTTTCATCTCAAGGCCGACAGAGAAGACGGTCTTCACCATCTTTATGGTGGGATCT
TCTGCTATCTGCATTGTGCTCAATGTGGCTGAACTGGCCTATCTGATTGTCAAAGCATTG
CTCAGGTGCTCAGCCAGAGCCAAAGGGAGGCGCTCATTTGTACACCAAGAGAAAATGTCC
ACAGAAAAGGCGCACCTACAGAATGAAAAAAACGCAAGGTTGCTGTCATCAGCTTCGGAC
TCATCGAGCAATAAGACTGTTTAA

>Dr-cx35.4-NM_001017685
ATGGACTGGAAGACTTTTCAAGCCCTGCTCAGCGGGGTGAACAAATACTCCACTGCATTC
GGCCGGATATGGCTCTCAGTGGTTTTTGTGTTCAGGGTCATGGTTTATGTCGTAGCGGCA
GAAAGAGTTTGGGGTGATGAGCAGAAAGACTTTGACTGCAACACCAAGCAGCCGGGCTGC
GCAAACGTCTGCTATGATTTCTACTTCCCCATTTCCCACATAAGACTATGGGCTCTGCAG
CTCATCTTCGTCACGTGTCCATCACTAATGGTGGTCATGCACGTGAAATACCGTGAGGAA
CGTGAACGCAAAGCCAAAGCAAAACTCTACGCCAACACGGGAAAGAAGCACGGTGGACTG
TGGTGGACGTATCTGATCAGCCTTTTTGCTAAGACTGGCATTGAGATCACCTTCCTGTAC
ATCCTCCACCACATCTACGACAGCTTCTACCTGCCAAGGCTGGTGAAGTGTGATGTCCAG
CCATGTCCCAATGTTGTGGACTGTTACATTGGCCGGCCCACAGAGAAAAGAGTCTTCACT
TATTTCATGGTGGGAGCGTCAGCGCTCTGCATAGTGCTCAGTGTCTGCGAGATCATCTAT
CTGATCGCCAAACGCATCAGCCGCTGCGCTAACAAATACAAGCAGCATGACAAAAGAAGC
ACACCGATTAATCAGCGATATCGAGATGAGGACAGCAACTGCACTATTCCTTTGCACGAG
CTGGAGAGCAAGCCCGAGTATAAACCAGAGACTAAAGAAGACTTTAGGTCTGAATATAAA
CCTGGCCCTAAACCGCAGTTTAAATCTGAAGTCAAGCCCACTTTTAAGCCAGCCTACAGG
TTGAGTGTGGACATGAGAGCGTCTGCTCCAAATCTCTCAGCACCAATGTACAAAATACAG
TCTGGTATCATCTAA

>Dr-cx34.4-NM_001130636
ATGAATTGGGCTTTTCTTCAGGGTCTCCTGAGCGGGGTCAACAAGTACTCCACAGCGTTC
GGCCGTGTCTGGCTCTCGATAGTCTTCCTTTTCAGAGTCATGGTTTTTGTAGTCGCGGCT
GAAAAAGTGTGGGGCGACGAGCAGAAAGACTTTGCGTGTAACACCGCCCAGCCGGGATGC
CATAATGTATGCTATGACCACTTCTTCCCCGTGTCCCACATCCGCCTCTGGGCTCTGCAA
CTCATCTTCGTCACTTGTCCGTCATTCATGGTGGTTTTACATGTGGCATATCGTGATGAA
CGTGAGCGGAAAAACCGTCTCAAATATGGTGAAGGATGTAAACGTTTGTACGACAACACC

GGAAAGAAACGTGGTGGTCTTTGGTGGACGTACGTGCTCTCGCTGGTTTTTAAAATGGGA
GTGGATGCGACTTTTGTGTATCTGCTGTACCACATCTACGAGGGCTACGATTTTCCAGTT
CTGGTGAAATGTTCTGAAGCTCCATGCCCAAACATTGTGGACTGCTTCATCTCGCGGCCC
ACAGAGAAGCGAATCTTCACCATCTTTATGGTGGTGACCAGTCTGGTGTGCATCCTGCTG
TCTCTCTTCGAGATCCTCTATCTGGTGGGCAAACGCTGCTTTGAATGCATCAATAGGGTG
CAGAGCTCACGACATGTGAACAGAGAGAGATCCATGGCTAATATGACAAACTTGAATGCT
CATTTAGAGTCAAACAACAAAAAACTGGCAAGCGAAGACCAGCCGGCACCAGCATACAGT
GTGGTCATGTCAGCCAAAAAGAAAACCAGCTTGGAAAATACTTTCAATCCGAGTTGGACC
TTGAGAGATGACAAATGTTCAACTTCACATTCACTTGGAGGTGAATGCGAACGTGACTGA

>Dr-cx28.6-NM_001007212
ATGAACTGGTCGGGATTGCAGTCCCTTCTGAGCGGGGTCAATCAATATTCGACCGTGTTT
GGTCGAGTGTGGCTATCCGTGGTGTTTGTGTTTCGCGTCCTGGTGTTTGTAGTGGCAGCT
CAACGCGTTTGGGGTGACGAAAACTTAGTGTGCAACACCAGGCAACCCGGCTGTGCCAAC
GTCTGCACCGACACCACATTCCCCATCTCTCACACCCATCTGTGGGCATTACAGCTCATC
TTCGTCACATGTCCGTCGCTCATGGTCATAGCCCACGTCAAACTCAGAGAAGACAAAAAC
AAGAAGTACACAGACGTCCATGAGGGAGAGCATTTATACGCCAACCCTGGAAAAAAGCGT
GGTGGTCTTTGGTGGACTTACCTGCTGAGCTTGCTCATTAAAGTCATTGTCGACGCTGGT
TTTCTGTATATTCTTCATTACTTGTATAACGGCTTTGATCTTCCTCGCCTTGTCAAGTGC
TCGCTGGATCCTTGTCCAAATACAGTGGACTGTTTCATCTCTCGTCCCACAGAGAAGAAG
ATCTTCATACTCTTCATGGTGATTTCCAGTGTGGTCTGCATCTTCATGTGCATCTGTGAA
ATGGCTTATCTCATTGGAAAGCGAGTATCCAATAACTGTATGATGGTAAAAGGACCAGCA
CAAAGATCCAAAACACATGATCCAACTCTTTCAAGCAGACAGAACTTAATTTCACAAAAA
ATAAAAGAGAAAAAAATAGACAATACAGCTCTTTAA

>Dr-cx30.9-NM_001007288
ATGAACTGGTTGTCCCTAGAAGTCCTGCTTGGCGGGGTTAGCCAATACTCCACTGTGTTT
GGCCGTGTCTATCTCTCCGTGGTGTTCATCTTCCGAATCCTGGTGTTTGTGGTGTCTGTC
CAGCAAGTCTGGAACGACGAACAGAAAGACTTCATCTGCAACACGGCCCAGCCAGGCTGC
ACCAATGTCTGCTACGACCAGTTCTTCCCCATATCCCACATCCGTCTATGGGCTCTCCAG
CTTATTTTCGTCACTTGTCCATCTCTCATGGTGGTCGCTCACGTCAAATATCGACAAATG
AAGAATGTGAAGTACAACACTGCCCGCAATGGTGAAAACATGTATGCAAACCCAGGAAGA
AAGCGTGGAGGCCTGTGGTACACCTATATCCTCAGCCTGTTATTCAAAGCCGGCTTTGAT
GCAGCATTCTTGTATATTTTGTACTACCTTTATAAATTCGACATGCCAAATGTTACCAAA
TGCACTGCGGAACCTTGTCCAAATACAGTGGACTGCTATATCTCCCGTCCCACAGAGAAG
AAAATCTTCACTCTTTTTATGGTGGTCTCCTCCTCTGTGTGCATCTTCATGTGTATCTGT
GAGATGGTGTATCTGATTACAAAGAAAGCTGGCAAATTTCTGCACAAAAAGAGTGAGGAG
AATAGAAACTACATAAGCAGAAGGGTGGATCCGACGGCCATGTCCAACCAGAATCTCAAC
AATCTTAAAATGGCACAGGCTGCAGAAGAACTAAAAAATTCTGAGATGATCCATTCCGAG
TTGAACAAATTATATGAACGCTGA

>Dr-cx28.8-NM_001045239
ATGAACTGGGGTTTCCTGGAGAACGTGTTGAGCGGGGTGAACCGCTACTCCACCGTGGTG
GGCCGGGTCTGGCTCTCCATCCTCTTCATCTTCCGCATCCTGGTGTTCGTGGCGGCCGCC
GAGCAGGTGTGGAAGGACGAGTTCAAGGACTTCGTCTGCAACACGCAGCAGCCGGGCTGC
GAGCAGGTGTGCTTCGACCACTTCTTCCCCATCTCTCAGGTGCGTCTGTGGGCGCTGCAG
CTCATCATGGTGTCCACGCCGTCGCTGCTGGTGGCTCTGCACGTGGCCTACCGAGAGCAC
CGCGAGCGCAAACACAAGCGCAGGCTCTACCAGGACAAGGGCAGCATTGACGGCGGCCTG
CTGTTCACGTACATCACCAGCCTGGTCCTCAAGACGTCTTTCGAGGTGGGCACGCTGCTG
GCCTTCTACCTGCTGTACAGCGGTTTCCACGTGCCGCGGCTGCTGCGCTGCGCCGAGAGC
CCCTGCCCCAACAGCGTGGACTGCTACATCGCCAGAGCCACCGAGAAGAAGATCTTCCTC
TACATCATGGGCTGCACCTCCATCCTGTGCATCGCGCTCAACCTGCTGGAGATGGGCTAC
ATCGTGTCCAAGCAGTGCTGGAAGAGCTTCAGCAAGAGATACACTCCGGTGCGGGATGGG
GCCACTCGCCCGCCCTCCACCTTTACTCTCGGCCCACCGCAACCCTCATGCACAGCCAAG
GAGGAGGGCGATCAGTCTGCGCCGGCGGGACAGGAGACAGCCTGA

>Dr-gjc1like-XM_679922
ATGAGCTGGAGCTTCCTGACGCGTCTGCTGGAGGAGATCCAGCACCATTCCACGTCGGTG
GGGAAACTCTGGCTCACCACGCTAGTTGTGTTTCGCATCGTGCTGACCGCGGTGGGCGGC
GAGTCTATATACTACGACGAGCAGAGCAAGTTCATCTGCAACTCTGCACAGCCGGGATGT
GAGAACGTCTGCTACGATGCCTTCGCGCCGCTGTCCCACGTGCGCTTTTGGGTCTTTCAG
ATCATCCTTTCCTCCCTGCCCTCTCTGCTGTACATGGGCTATGCCGCGAATAAGATCTCA
CACAGAGAGGATTTACGGGGCGGCTCGGGGGCCGGGGCTCCTTCCACAGGAGATTCGGCA
GGGGGCGGATACACTCAACGCAGGCCGAGAAAGATGTACTTTGGGGCACGGCAGCATCGG
CCAGGACATGAGGATGGGGAGGAAGAGCGAGAAGATGACCCCATGATCTACGAAGTGCCT
GAGATAGACACCACACGTCGGGAATTAGTGCCACCGCGACCTAAACCCAAAGTGCGTCAC
GATGGGCGTAGACGCATTCAAAATGATGGCCTGATGCGAGTTTACGTGCTACAGCTGTTG
ACACGATTTGTTCTGGAGGCCATTTTTCTTGCAGGACAGTATCTGTTGTATGGCTTCCGC

GTGGAACCTGTTTTCGTGTGCACGGATGTTCCCTGCCCGCACCGGGTGGACTGCTTCATC
TCACGGCCCACTGAAAAAACCATCTTCCTTAGAATCATGTATGGCGTCAGCTGCCTGTGC
CTGCTGCTAAACCTGTGGGAAATGATTCATCTCGGCGTGGGAACCATCAGCGATGTCTTG
CGCAAACGAAACGCGGCAGCTAGCGATGATGAGTATCAGCTCGGCCTGCTGGCATCCGGC
GGCGTTTCGGTGGGAGTCGGCGGGCCTTCACTTAGTGAAGGAGAACCTGTAGGTGGAGTC
GGCGGTGGTGTTCGAGAAGCGGATTACGTTGGTTATCCTTTCTCCTGGAACACCCCATCT
GCACCACCTGGGTATAATATCGTGGTGAAACCTGAAACGATGCCCTACACAGACTTGAGC
AATGCTAAAATGGCGTGCAAGCAGAACCGCGCCAACATCGCCCAGGAGGAGCAGCAGCAA
TACGGCTCCAACGAGGACAACTTCCCATCTGCAGGTGAAACACGGCCGCCACCTATTAAC
AAAGATGTGATACAGTTGGAGGCGGCCATTCAGGCTTATACCTTGCAGCACCATGCTAGC
AATAACCACGATGAACTGGACGACACTAATGATATTGATGAGAAGCCTCAGAGCAATATC
ACCACAGCGCCACAGAAGGAGCGAAAGCAACGGTCCAAGCATGGGAAATCCGGGAGCGCT
GGGAGCAGCAGCAGCAGCAAATCAGGGGAGGGAAAGCCATCTGTCTGGATCTGA

>Dr-cx47.1-NM_001004574
ATGAGCTGGAGCTTTCTCACTCGACTCTTGGAGGAAATCCACAACCACTCCACATTTGTG
GGGAAGGTCTGGCTGACGGTCTTGATCATCTTCCGGATCGTTCTGACCGCAGTCGGGGGC
GAGTCGATCTACTCGGATGAGCAGACAAAGTTCACCTGCAACACAAAGCAGCCCGGCTGT
GACAACGTCTGCTACGACGCCTTCGCACCGCTCTCACACGTCCGCTTCTGGGTCTTCCAG
ATAATCATGATTTCCACACCCTCCGTCATGTATCTGGGATATGCCATCCATAAGATCGCC
AAAACCTCAGAGGAGGAACGACACAAGAACCAGATTTACCAGAAGAGGAGGCACCACAGT
CGCTGGAGAAACGGACACCATCTAGAGGACGCTTTAGAGGAGGAAGATGAGGACGCGGAG
CCAATGATCTACGAAGAAGATGCACGAGAGATCAAAGCAGAGACTGTCCGAGATCCCCTA
AAACACGATGGCCGCCGCAGGATCATGCAAGAAGGTTTAATGAGGATGTATGTTCTTCAA
CTTTTATCCCGCGCCATCTTCGAGGTGGGATTCCTCACGGGTCAGTATCTCCTCTACGGC
TTCCGCGTCAACCCTTCGTACGTCTGCAACAAGATCCCATGCCCACACAGGGTGGACTGC
TTTGTTTCAAGACCCACCGAGAAGACCATCTTTTTGCTCATCATGTATGTGGTGAGCTGT
CTATGTCTGCTGCTCAATGTTTGCGAGATGTTTCACTTGGGGATCGGTGCCTTTCGAGAC
ACTCTTCGCAAACGTCGAAACCAGAATCAGCGACCTTCCTATGGCTACCCTTACTCCAGG
AATATTTCCAGTTCTCCGCCAGGATACAACTTAGTTGTTAAATCCGACAAACCCGGTCGC
ATTCCCAACAGCATCGTCCTGCCTGATCAGAACATGGATAGAGAGATCGCAGAACAACAC
TGCACAAGTCCTGATGAGAACATCCCCACTGACCTAGCAACCTTGCACCACCATTTACGA
GTAGCTCAGGAGCAGCTTGACATGGCTTTTCAGACATACAACACAAAAACCACTCATATT
TCAAGAGCCAGCAGCCCCGTTTCTGGTGGCACAACGACAGAGCAGAACCGCATCAACATG
GCTCAGGAGAAGCAGGGCGCTCGGCCCAAAGCAAGCACCGAGAGAGCTGGGACACTAGTA
AAAAATGGAAAAACTTCGGTGTGGATTTAA

>Dr-cx44.2-NM_131810
ATGAGTTGGAGCTTCTTGACACGTTTGCTTGATGAAATCTCCAACCATTCTACTTTTGTG
GGAAAGATCTGGCTCACTCTCCTCATTATCTTCCGAATCGTCCTGACAGTGGTGGGCGGT
GAGACCATCTATCAGGATGAACAGAGCAAGTTTGTATGCAATACACAACAGCCTGGTTGT
GAGAACGTGTGCTATGATGCTTTTGCCCCTTTATCGCATGTTAGATTTTGGGTTTTTCAA
ATTATTGTGATAACCACTCCATCCATTATGTATCTCGGCTTTGCCATGCACAAAATTGCT
CGAATGGCCGATGATGAATATCGACCACGCAAACGCAAAATGCTGTCTATGGTTCATCGA
GGTATGAGCCGTGACTATGACATGGTTGACGAGATGAGTGAAGAAGTTCCCATGATCCCA
GAAGAGATTGAGCCCTCAGAGAAAAACAACAAATCAGCAGCTTCAACCAAGACTACCGCT
GCTTCTGATGCTGCCGTGAAACATGATGGTCGACGCCGCATCAAGAGAGATGGTCTCATG
AAGGTGTACGTGTGTTACAGTTGATCTCTCGTGTTGCCTTTGAGATAGCTTTCCTCTTTGGC
CAATATATTCTTTATGGTTTTGAGGTCTCTCCGTCCTACATTTGCACCCGAAGCCCTTGC
CCACACACTGTGGATTGCTTTGTCTCACGTCCCACTGAAAAAACCATCTTCCTGGTCATC
ATGTATGTTGTCAGTGTACTCTGTCTGGCACTGACTGTATTGGAAATCCTGCATCTGGGA
ATTGGTGGCTTGAGGGACTCTCTTCGTAATCGAGCAAATCGGAGACTCCCTGTTCATAGG
CCATCCACGTCCACCATCTGTCACCGCCTTCCCAGTGCTCCACCTGGATACCAGGCTGTC
CTAAAAAAGTACTCCTCAGGCAAGCTGAAGGCTGAGTTTCTAGCAGACTCGGGACGGGAT
TCAATGGGTGGTGACAATACTACTCGTGATCTAGACCGTCTGCGGAGGCATCTGAAAATT
GCACAGCAACACCTGGACCAGGCCTACCACACTGAGGAAGTAGGGGCTTCACACAACAGC
GGGCCTGACTCTAAAAGCATCGCTGCTGAGCAAAACCGACTCAACCAAGCGCAGGAAGGC
TTTGGCAGCACTGAGGAGAAAGGTTAG

>Dr-cx43.4-NM_131069
ATGAGCTGGAGTTTTCTTACGCGGTTGTTGGATGAAATCTCCAACCACTCCACCTTCGTG
GGCAAGATATGGCTCACGTTATTCATCATCTTCCGCATTGTTTTGACTGTTGTGGGGGGA
GAATCGATATACTACGATGAACAGAGCAAATTTGTGTGTAATACCCAGCAACCTGGTTGT
GAGAACGTTTGCTACGATGCATTTGCACCACTCTCTCATGTCCGGTTCTGGGTTTTCCAG
ATCATTTTGATCACAACCCCCACTATCATGTACTTGGGATTTGCTATGCACAAGATCGCT
CGGTCAAATGATGTGGAGTACAGGCCAGTCAACAGGAAACGCATGCCAATGATCAACCGC
GGAGCCAACCGGGATTATGAGGAGGCCGAAGACAACGGTGAGGAAGATCCTATGATTATG
GAAGAGATCGTGCCTGAGAAAGAAAAGGCTCCAGAGAAGTCTGCTGTTAAACATGACGGC

CGGCGGAGAATAAAGCGAGATGGGCTCATGAAGGTGTACATCCTGCAGCTTCTGTCGAGG
ATTATTTTCGAGGTGGGCTTTCTCTTTGGCCAGTATATCCTGTATGGTTTCGAGGTCGCC
CCGTCATACGTGTGCACTCGCAGTCCCTGCCCGCACACCGTAGACTGCTTTGTGTCACGT
CCGACAGAGAAAACCATCTTTCTGCTGATTATGTATGCCGTGAGCTGTCTCTGCTTGTCT
CTTACGGTGCTGGAGATACTTCATTTGGGCCTCAGCGGAATTCGTGATGCTTTTCGACGA
CGTGCACGCCATCAAAGTGTTCAGCGCCCACGTGCCCCCATATGCAGACAGGTGCCCACT
GCCCCGCCAGGGTACCACACTGCCCTGAAAAAAGACAAGCTGTCTTTGGGAATGAAACCT
GAGTATAACTTGGACTCCGGTCGGGAGTCTTTTGGTGACGAGTCGTCATCGCGAGACATT
GACCGCCTGCGCAGGCACCTGAAACTGGCTCAGCAACATTTAGATTTGGCCTATCAGAAT
GGCGAGAGCAGTCCTTCACGCAGCAGCAGCCCAGAGTCCAACGGCACTGCTGTCGAGCAG
AACAGACTTAACTTTGCTCAGGAGAAGCAGGGGAGCAAATGTGAAAAAGGGATCCATGCT
TGA

>Dr-NN-gjd2-G67999
AGGATCCTCCTAACTGTGGTGGTGATCTTCCGGATCCTGATCGTAGCCATAGTAGGAGAG
ACGGTGTATGATGACGAGCAGACCATGTTCGTCTGTAACGCCTTGCAACCGGGTTGCAAC
CAGGCGTGCTACGACAAAGCCTTCCCGATCTCGCACATCAGATACTGGGTGTTTCAGATC
ATCATGGTGTGCACGCCGAGCCTCTGCTTCATCACGTACTCGGTCCATCAGTCTGCTAAG
CAGAAGGAGCGGCGCTACTCCACTGCCACCGTCTTCCTGACAGTGGACAGCAAGGAGCAG
GACTCGCTGAAGCGAGAGGAGGCCAAAAACCAGAAGATCAAGAACACCATCATGAACGGA
GTACTTCAGAACACGGAGAACTCCACCAAAGAAGCCGAACCTGATTGCCTGGAGTCCAAA
GAGCTGGTCAGCTCCAACACCAAGCCGGCAAAGTCCAAAATGCGGCGGCAGGAGGGCATC
TCCAGGTTTTACATCATCCAGGTGGTGTTCAGAAACGCTCTAGAGATCGGCTTCCTAGTG
GGCCAGTATTTCCTGTACGGATTCAACGTGCCGGCGGTGTACGAGTGCGACCGCTACCCA
TGCATCAAGGAGGTGGAGTGCTACGTGTCCAGGCCCACGGAGAAAACCGTGTTCCTAGTC
TTCATGTTCGCGGTCAGCGGCTTTTGCGTGATTCTCAATCTAGCCGAGCTCAATCATCTA
GGCTGGCGGAAGATCAAAACGGCGGTGAGGGGCGTGCAGGCCCGCAGGAAGTCCATCTAT
GAGATCAGGAACAAGGATTTGCCGCGGATGAGTATGCCCAATTTCGGCCGCACTCAGTCC
AGTGACTCGGCCTACGTGTAA

>Dr-gjd2b-NM_194420 Splice site.
ATGGGGGAATGGACAATTCTCGAGCGTCTCCTGGAGGCGGCTGTCCAACAGCACTCTACT
ATGATTGGGAGGATCCTGCTAACTGTTGTGGTGATCTTCCGGATTCTAATTGTGGCGATT
GTTGGAGAGACCGTGTACGACGACGAGCAGTCAATGTTTGTGTGTAACACTCTGCAGCCA
GGCTGTAACCAAGCTTGCTATGACAAAGCGTTCCCTATATCTCACATCAGATACTGGGTT
TTCCAGATCATCATGGTTTGCACACCCAGTCTCTGCTTCATCACATACTCTGTGCATCAG
TCGGCCAAACAGAAGGAGCGGAGGTATTCTACCATCTACCTGTCCCTCGACAAAGACCCC
GATACGATGAGGCGAGACGACAGCAAAAGATCAAAAACACCATTGTGAACGGAGTACTT
CAAAACACGGAGAACTCCACCAAAGAGTCCGAGCCTGACTGTCTAGAGGTCAAAGAGATC
CCCAATTCAGCCATGAGAACTACCAAATCTAAAATGAGAAGACAAGAGGGCATCTCCAGG
TTTTACATCATTCAGGTGGTGTTCAGAAACGCACTGGAAATTGGCTTCCTGGTGGGCCAG
TATTTCTTGTACGGATTCAACGTGCCCGCCGTGTACGAGTGCGACCGCTATCCTTGCATC
AAAGACGTCGAATGCTACGTATCAAGACCTACGGAGAAAACCGTGTTCCTCGTCTTCATG
TTTGCAGTCAGTGGGATTTGCGTGGTGCTCAACCTGGCTGAACTCAATCACCTGGGCTGG
AGGAAAATTAAAACAGCGGTGAGGGGGGTTCAGGCCAGGAGGAAGTCCATCTATGAGATC
AGAAACAAGGACTTACCGCGGATGAGCATGCCGAATTTCGGAAGAACCCAGTCCAGTGAC
TCTGCCTACGTTTAA

>Dr-gjd1a-NM_001128766 (a gjd2*2 sequence) Splice site
ATGGGAGAATGGACTATATTGGAGAGGTTGCTGGAGGCTGCGGTACAGCAGCACTCTACT
ATGATCGGCAGGATCCTGCTGACAGTAGTGGTGATATTCCGGATCCTGATCGTGGGTATA
GTGGGAGAGAAGGTGTATGAGGACGAACAAATTATGTTTATATGTAACACACTGCAACCG
GGTTGCAACCAGGCCTGCTACGATAAAGCCTTCCCAATCTCCCATATCCGTTACTGGGTT
TTCCAGATCATCCTGGTTTGCACGCCCAGCCTCTGCTTTATCACATACTCTGTGCACCAG
TCTGCCAAGCACAAAGACCAGCGTTACACACTTCTGCATGGCCCTTACATCGACCACGGT
CATGGGCCGAGCCGCAAGCTCCGCAACATCAACGGCATCCTGGTGCACCCGGAAAGCAAA
GACGACCGCGAATGTCTGGATCTGAAAGACATTCCCAACATCCCGGCGGGGGTGACATAC
TCCAAAAGTGCCAAGATCCGCCAGCAGGAAGGCATCTCCCGCTTCTATGTCATCCAAGTG
GTGTTCCGGAACGTTCTGGAAATCGGCTTTCTAGCCGGCCAGTACTTCCTCTACGGATTC
AATGTTCCCGCCATGTTTGAGTGCGACCGCTACCCTTGCGTGAAGGAGGTTGAATGTTAC
GTGTCGCGTCCCACAGAGAAGACAGTTTTCCTGGTGTTCATGTTCGCAGTTAGCGGAATC
TGCGTGGTGCTAAATTTGGCTGAGCTCAACCACCTTGGCTGGCGCAAGATTAAGACGGCC
ATCCGTGGCGTCCAAGCTCGTAGAAAGTCTATTTGTGAGATCCGAAAAAAGGATGTGTCT
CACTTGTCCTCCGTGCCCAACTTGGGCCGCACCCAGTCTAGCGAATCAGCTTATGTCTGA

>Dr-gjd2like-XM_009291479 (a gjd2*2 sequence) Splice site.
ATGGGAGAGTGGACCATTTTAGAGCGCCTCCTGGAGGCCGCTGTGCAGCAGCACTCTACT
ATGATCGGAAGGATTTTGCTGACAGTTGTGGTGATATTCCGTATCCTAATCGTGGCCATT

GTTGGTGAAACCGTCTATGAGGATGAGCAGACCATGTTTATCTGCAACACCCTCCAACCG
GGCTGCAACCAGGCCTGCTACGACAAAGCCTTCCCCATTTCCCACATCCGATACTGGGTC
TTCCAGATTATCCTTGTCTGTACACCAAGCCTGTGCTTCATCACCTATTCAGTACACCAG
TCAGCCAAACAGCGTGACCGCCGCTACTCCTTCCTCTACCCAATAATGGAAAAGGACTAC
AGTCGTGAGGGTACGCGGAAACTACGCAACATTAACGGCATTTTGGTGCAGCACTCTGAA
AGTGGCGGTGGAAAGGATGAACCTGATTGCTTAGAGGTAAAGGAGATCCCAAATGCTCCA
AGAGGCCTCTTGCATGGCAAGAGTTCAAAGGTACGCCGACAAGAGGGGATTTCTCGCTTC
TACATAATCCAGGTAGTGTTCCGCAATGCACTGGAGATAGGTTTCTTAGCAGGGCAGTAC
TTTCTATATGGCTTCAGCGTTCCTGGCATCTTCGAGTGTGACCGATACCCCTGCCTGAAG
GAAGTGGAGTGCTACGTATCCCGACCCACTGAGAAGACGGTCTTCCTAGTGTTCATGTTT
GCAGTGAGTGGCATCTGCGTTGTGCTGAACCTCGCTGAACTCAACCACCTCGGCTGGAGA
AAAATCAAAGCTGCCATTCGAGGTGTCCAAGCCCGCAGGAAGTCCATCTGTGAGATCCGC
AAAAAGGACATGGCCCACCTGTCACAACCGCCAAACCTGGGCAGGACCCAGTCCAGCGAG
TCTGCTTATGTTTGA

>Dr-cx36.7-NM_001103197
ATGACAGAATGGACGCTGCTGAAGCGGCTGCTGGACGCCGTGCACCAGCACTCCACCATG
ATCGGACGCCTCTGGCTCACAATAATGGTGATTTTCAGATTGCTGATCGTCGCAGTGGCG
ACCGAAGACGTCTACACTGATGAGCAGGAGATGTTCGTGTGCAATACTCTCCAACCGGGA
TGTCCGAACGTCTGCTATGATGCATTCGCGCCAATATCGCAACCACGTTTTGGGTCTTC
CAGATCATCACGGTCTCCACGCCGTCGCTTTGTTTTATTATCTACACCTGGCACAACTTG
TCCAAACAACCCGAAGGTGAGCAGATAAAGGAAGCGCTTGAGAGGAGCTGCGACTCGGAG
AGTTGCTCCATTAAATCGCATAAACACATAAATCCAAGCCTTGAAGGAGTCACCAACCAG
AAACCATCGCAAGCATCGAAAACCTCTTCGGGAGTCCTTTCGAAGTATTACATCTTCCAC
GTTTGCTTTCGTACCATTCTGGAAGTGGCCTTTGTAGTGGCCCAGTGGCTGCTTTTCGGC
TTCCGCGTCCCAGCTCATTTCGTTTGCACGTCTTCCCCTTGCATGCAAAGCGTCGACTGC
TACGTTTCTCGTCCCACGGAGAAAACCGTTTTTCTTATCTTTATGTTCTGCGTTGGAGTT
TTCTGCATCTTCTTGAACTTCTTAGAGCTCAATCATTTGGCTTGGAAGATGATCAAGAGG
TCTGTGCTGGTCAAGGACGGATCCTGGAATGGATACGGCGCTATAAACCAAGACTCGCAG
TCGATCGCTTCTTTGACGTTTCGAGATGTTACCAGCACTACATCACTACCGACTCTTGAT
CTAGTTGTGGATCACCAACCTGACTGGACATGCGCTGCAAACTGCTCGACAAAGAAGGAC
AATAGAGGAACACAGAGTAAACCCAAGACAAACAGAAAAGCAAAACAGAGGAGCACTGAG
GTTTGGATATAA

>Dr-gjd2like-XM_009291771 (a cx39.2 sequence)
ATGGGTGATTGGTCAATTCTGGGCCGCTTTCTAACCGAGGTTCAGAATCATTCAACAGTC
ATCGGCAAAATCTGGCTGACGGTGCTACTGATCTTCCGCATACTGCTGGTCACCCTGGTG
GGAGATGCAGTGTACAGCGACGAGCAGTCCAAATTCACCTGCAACACCCTTCAGCCGGGC
TGCAACAACGTCTGCTATGATACATTCGCCCCCGTCTCACACTTACGCTTCTGGGTCTTC
CAGATCGTTCTGGTCTCAACGCCCTCCATCTTCTACATCATCTATGTGCTGCACAAAATC
ACCAAAGATGAGAAGATGGAGACGGAGAGGATCCACGCAGAGGCCAGTCACCCTAGTCGA
ATACAAGGAGATGGTTCCAGACTGGCCTACGGAGCCCAGGGAGAAGAATGGGGTGGTCAG
GACGAGGGAAGCGTTGAGCAAAGTCTCCTGCAGGAAGATTTCGGTGAGCTCGGCAAAGAT
CCAACCACACTTTCCAGTCAGGTTCTACTCATTTATATTGTTCACGTCCTGATCCGCTCC
GTCCTGGAGATCACCTTTCTTGTCGGTCAGTATTACTTGTTTGGATTCGAGGTGCCTCAT
TTGTTCCGCTGCCAAACGTACCCTTGCCCAACACGGACTGACTGTTTTGTGTCTCGAGCC
ACCGAAAAGACTATTTTCCTTAATTTTATGTTCAGCATCAGCTTGGGTTGTTTCCTCCTG
AACATTGTGGAGCTTCACTACCTGGGTTGGGTCTACATTTTCCGTATGCTCTGCGCCGCC
TGCTTCTTGTGCTGCAAGTCAGAGAGGGATTTGTATGCTCAACGAAACCCACTGTTGCTT
CGCCTCAGACACTCGATGCAGAGCAGGCTGGTCCTGCAGTCTTCAACAACCACTCTGTCT
CAGGAGAAGACCGGGACGGCTTTGCTTTCACATGGTCCTGTCATCTCTTTTGAGACCGAC
TCGACTCTTGAAAGCTCCTCAAAGAGGAACCCTGAGGAGAGGGAACGCATGAGGGTCAAA
TTGGCCAACATGGTTAGATTTACTGGTAAAAAGTCTTGGCTGTGA

>Dr-gjd4-XM_021470260 Splice site.
ATGGCCAAACAAGCTACATCAGAAGTTATCTTCATAACGCTGAACCACAACATCACCCTC
ACAGGGAAAGCTTGGCTCGTCCTGGTGGTATTTCTAAGGATCCTGGTGTTGCTGTTTGCT
GGTTATCCTCTCTACCAGGATGAGCAGGAACGATTTGTGTGTAACACCATTCAGCCCGGT
TGTGCCAATGTGTGCTATGACATGTTTGCCCCTCTGTCCCTCTTCCGCTTCTGGCTTGTA
CAACTGACCACTCTGTGCCTCCCCTACATAATGTTCATCATCTATGTGATCCACAAAGTG
AGTTCTGGCTTAGCTACCGATACCGGAACTTCCGAGTCCATAAAAGCGGACTCCATCTAC
AAGATCCATCAAGAATCATTCAGGAAAGCATCTCTTTGTAAGATGGTCATGAAGGCTGAG
AAGGGAAGGGTGCAGTACTTCACGGGAGCCTACATCTTGCATCTTCTGCTTCGGATAATG
GTAGAAGCTGGATTTGGAGCTGCCCATTATTACTTGTTCGGCTTTCACATCCCCAGACGC
TTTATGTGTCAGCAGGCACCCTGCACAACAATGGTGGACTGCTACATCTCTAGACCCACT
GAGAAAACCGTCATGCTGAACTTCATGTTAGGGGCAGCCGCTTTGTCCCTGCTATTAAAC
ATCTGCGACCTGATTTGTGCAATCAAGCGCTCTGTGAGGCAAAAAAACAAACGAAAGATG
CTAGTGCAAAAGATGTATGCAGAGGAGCAGTACTACGTGTCAGGGAATGGAAATCAAGGT

GTGGACGCTAGCAGCCCTCCAAATCAAGATGTGATGAGTCCAGGAGTGTTTCGCAAAAGA
GGGACCAGAAACTCAAGTGGCGATGAAGCTGCTTCTGTGCTTTTGGATGATGACCCTCCG
CCATCCTTACCTCAAGAAGGAAAGCCTACAATTTCAGGAATGCCTGGTTGCAGAAGCAAT
GATGACAGCAGCAGTTACCAACCCACCCAAGAAGGGGGGATGGTAAGAGAGGGCAGTGAA
GTGGCCCTGTGCCCCAGTGAGCCCTTGGGAACCCCTAGATCCATCCGAGTAAGCAAACGT
AGTCGGCTGAAACCTCCACCACCTCCTCGAAGGGATAAACTTGCCGTGCAAGGTGCAATT
GATGGCTCTGGAGCGACAGCATTGTGTACCAGAAGAGTAGGGCAATATACTCTGGTAGAA
ATGACCACTGGTGAAGACATAACAACTTGCGGTGGAGATGGGAAAGAGAAAAAGTCAGAG
TGGGTTTGA

>Dr-cx23-NM_001013546 Splice site.
ATGTCATTAAATTACATCAAAAACTTTTATGAAGGATGCCTCAGGCCTCCGACAGTGATA
GGCCAGTTCCACACGCTGTTTTTTGGCTCTGTGCGTACCTTTTTTCTTGGAGTCCTTGGA
TTTGCTGTCTACGGCAATGAGGCCCTGCACTTCAGCTGTGATCCGGACAAGAGGGAATTA
AACCTTTACTGTTACAACCAGTTCAGGCCTATAACACCTCAGGTTTTCTGGGCGTTACAG
CTAGTCACTGTCTTGGTACCTGGAGCAGTGTTTCATCTTTATGCTGCCTGTAAGAATATA
GACCAGGAGGAGATCCTTCATCGGCCGATGTCCACAGTCTTTTACATCATCTCTGTCCTG
TTAAGAATAATTCTAGAAGTCTTAGCCTTTTGGCTACAGAGCCACCTTTTTGGTTTCCTG
GTTGATCCTATTTTCATGTGCGATGTCACCGGCCTTGGAAAGATCCTCAACGTCTCAAAG
TGCATGGTTCCTGAACACTTTGAGAAGACCATCTTCCTCAGTGCAATGTACACCTTCACC
ATCATCACCATACTGCTCTGTATCGCTGAGATTTTTGAGATTTTGTTCCGAAGACTTGGC
TATTTAAACCAGCCAATGACTTAG

>Dr-gje1like-XM_021473060 Grey font: regarded as intron, although the GenBank entry claims it to be a part of the exon. Splice site.
TTTGTGCAGCTCCGGCCTCCGACTGTGATTGGTCAGTTCCACACGCTGTTCTTCGGCTCA
GTGCGCATGTTCTTTCTGGGGGTTCTGGGATTTGCAGTTTACGGGAATGAAGCGCTTCAC
TTCAGCTGTGATCCGGATAGGAGGGAGATCAACTTATTCTGCTACAACCAGTTTAGGCCC
GTCACACCGCAGGTGTTTTGGGCGCTCCAGCTCGTGACGGTCCTCGTTCCCGGTGCAGTT
TTTCATCTTTATGCGGCGTATAAAAACATCGACCAGGAGGAGATTCTGGAGCGGCGCTCA
TTTACTGTGTTTTACATCATCTCTGTACTCCTGCGGATCCTTCTGGAGGTTGCGGCTTTC
TGGCTCCAGAGTCGTCTGTTCGGTTTTTTGGTTCACCCGTTGTATTCCTGCGACTCCAGA
CCTCTGGACAGCAGGCTCAACTTCACCAAATGTATGGTTCCCGAACACTTTGAGAAAACC
ATCTTCCTCAGCGCCATGTACACCTTCACCATCATCACCATGATATTGTGCGTGGCGGAG
ATTTTCGAGATCCTCTGCAGAAGGCTGGGGTATTTAACACATCAGTGA

Suppl. Fig. 6. Japanese pufferfish (Fugu; *Takifugu rubripes*) connexins.
Yellow: Conserved domains as defined by Cruciani and Mikalsen (2007)
Green: Conserved cysteine codons (cysteine signature)
Grey: 15 nt added at the ends of the conserved domains
Turquoise: Splice site.
Other colors are explained where necessary.

```
>Fr-gja1-43-XM_011618634
ATGGGTGACTGGAGTGCTCTGGGTCGGTTGTTGGACAAGGTTCAGGCCTACTCCACTGCT
GGAGGGAAGGTGTGGCTCTCTGTCCTCTTCATATTCCGGATCCTTGTTCTGGGCACTGCT
GTGGAATCAGCGTGGGGAGATGAGCAGTCTGCCTTCAAATGCAACACCCAGCAGCCTGGT
TGCGAGAATGTCTGCTATGACAAGTCTTTCCCTATCTCCCATGTTCGCTTCTGGGTCCTA
CAGATCATCTTCGTGTCAACACCTACCCTCCTGTACTTGGCTCATGTTTTCTACCTGAAT
AGGAAAGAACAGAAATTCAGCAAGATTGAAGAGGTGCTCAAAGCTGTACAAAATGACGGA
GGCGACGTTGATGTCCCACTGAAGAAGATTGAGATGAAAAAGCTTAAATATGGCATCGAG
GAACATGGGAAAGTGAAGATGAAAGGAGCCCTGCTGAGAACTTACATAGTCAGCATCTTC
TTCAAGTCACTTTTTGAGGTGGGCTTCCTGGTGATTCAGTGGTACATCTATGGCTTCAGT
TTGTCTGCTGTCTACACCTGTGAGAGGTCCCCATGTCCACACAGGGTGGACTGTTTCTTG
TCCCGTCCCACTGAGAAGACGGTCTTCATTATTTTCATGTTGGTGGTCTCGCTCGTATCC
CTGGTACTTAATATTATTGAGCTCTTCTATGTGCTTTTCAAGAATATCAAAGATCGTGTG
AAGGGCAAACAGCAGCCCACGCTCTACCCCAGCGCTGGCACCCTCAGCCCTATGCCCAAA
GAGCTGTCCACTACCAAGTATGCCTACTACAACGGTTGTTCCTCGCCAACTGCACCACTT
TCACCCATGTCGCCGCCAGGTTATAAGATGGCCACAGGGGAGCGGGGGGCCGGATCGTGC
CGTAATTATAATAAGCACGCTAGCGAGCAGAATTGGGCCAACTACTCCACAGAGCAGAAG
CGACTCGGACAGAATGGAGGAGGAAGCACAATTTCAAATTCCCACGCCCAAGCGTTTGAC
TTCCCGGATGATACCCAAGAGCACAAGAAAATGTCCTCATTGGCAGCTCATGAGCTGCAA
CCGTTAGCGCTGATGGACGCTCGTCCTTGCAGCCGGGCAAGCAGCCGATTGAGCAGTCGA
GCACGGCCAGATGACCTAGATGTTTGA

>Fr-gja3-46-XM_003962226
ATGGGCGACTGGAGCTTTCTGGGGCGGCTGTTGGAGAACGCTCAGGAGCATTCTACGGTC
ATCGGCAAAGTCTGGCTGACTGTCCTCTTCATCTTCAGGATCCTGGTGCTGGGGGCAGCC
GCCGAGGAGGTCTGGGGGGACGAGCAGTCTGATTTCACCTGCAACACCCAGCAGCCCGGT
TGCGAGAATGTCTGCTACGACGAGGCCTTCCCCATTTCGCACATCCGCTTCTGGGTGCTG
CAGATCATCTTCGTGTCGACGCCGACCCTCATCTACCTGGGCCACGTGCTGCACATCGTC
CGCATGGAGGAGAAGCGGAAGGAGAAGGAGGAGGAGCTGCGGAAAGCAAACCGGTTACAG
GAGGAGAAAGAACTCCTTTACAGAAACGGGGGAGACGCAGGAGGAGGCGGCAAGAAGGAG
AAGCCGCCCATCAGGGATGAGCACGGCAAAATCCGCATCAGAGGTGCACTGCTGCGTACC
TATGTGTTCAACATTATATTCAAAACCCTGTTTGAAGTGGGATTCATTTTGGCCAGTAT
TTCCTCTACGGCTTCCAGCTGAGGCCCCTGTACAAGTGTGGACGTTGGCCCTGCCCCAAC
ACTGTAGACTGCTTCATTTCCAGACCTACTGAAAAGACAATTTTTATTATATTTATGCTT
GTGGTGGCTTGCGTGTCTCTTTTGCTGAATTTGTTAGAGATCTATCACCTCGGATGGAAG
AAAGTTAAACAGGGCGTGACAAACCAGTTTGTCCCCGACCGCGAATCAATGCGCCGGGTC
AACATTGCAGAGCCCGAGTGTTTGGCCTTGGCCTCCAGAACTGCCCCATCCAGTTACCCC
CCCAACTACACTGATGTGACGGCGGGCAGTGGGGCGTTCCTTCAGCCCGTGGCGCCGCCG
GCCGTGCCTTCGACCACGGAGTTCAAGACCGACGACCTCCAGCGGGAGCCGCCTCGCCAC
CAGCCCTCCGCCTCTCACTACTACATCAGCAACAACAACAACCACAGGCTGGCCACGCAG
CAGAACTGGGCCAACCTGGCCACTGAGCAGCAGACTCGGGAGATGAAGGCCACCTCCTCC
TCCTCCTCCTCCACCAATGACGAGCAGCAGCAGCCCGTCGATGCGGAGCTGCTCCCTTCC
GCCAGCAGCAACATCACCAACACAACCACCACCGTCACCGCGTCAGGTAGCAGTAGCCCG
GGCTCGGCCTCCAACACGGGCAGCTGGGGCGGAGGAAGGAAGGAGCGGGGGGAAAACGGC
GTCTCCACCACCAGGGTGGAGATGCACGAGCCTCCGGCGACGGCCGGCGTGGACCCTCGG
CGACTTAGCCGAGCCAGCAAGAGCAGCAGCGTCAGAGCGAGGCCAAGCGACCTGGCTGTC
TAA

>Fr-gja3like-XM_003966473
ATGGGTGACTGGAGCTTTCTAGGGCGGCTGCTGGAGAATGCTCAAGAACACTCCACTGTG
ATTGGAAAGGTTTGGCTGACTGTCCTGTTTATCTTCCGCATCTTGGTGCTGGGTGCAGCA
GCCGAAGAAGTTTGGGGTGATGAGCAGTCTGATTTCACCTGTAACACGCAGCAGCCTGGT
TGTGAGAACGTCTGCTATGATGAAGCCTTCCCTATCTCCCACATTCGCTTCTGGGTGCTG
CAGATCATTTTTGTCTCCACTCCAACACTCATCTACCTGGGCCACGTCCTTCACATTGTC
CGCATGGAGGAGAAGAGGAGAGAGAGGGAGGAGGACCTCCGAAAGGCAGGACGGCACCAG
GAGGACCATGATCCTCTCTATCATAATGGAGTTAGCAATGGAGGAAGCAGAGGTGGTGGC
AAAAAAGAAAAGCCACCTATTCGTGATGAACACGGGAAGATTCGCATCCGTGGGGCGTTA
CTGAGGACCTACATCTTTAACATCATCTTCAAGACTCTGTTTGAAGTGGGTTTCATCCTG
GGGCAGTACTTCCTCTATGGCTTCCATCTGAGGCCGCTCTACAAATGTGGCCGCTGGCCC
```

TGCCCAAACACCGTGGACTGCTTCATCTCCAGGCCCACTGAAAAGACGATCTTCATCATC
TTTATGCTGGTGGTCGCATGCATCTCGTTGGCCCTCAACCTGTTGGAGATCTACCACCTG
GGATGGAAGAAGGTCAAGCAGGGAGTCACCAATGAGTTTGTCCCCGATGGCGAGTTGCTG
CCGCAGAGTGCAGACGAGCACAGAGACATGGAGAAGATCCACGAGCAGACTTCTCCATCG
GCACTTGAATGTTTGTCGACATATTCCAGCATGAATATGGCAGGACGCGTAGCTGAAGAA
GGAGGAACCTACAGTCCACCCGAGGCCTCTCTAGCAGTAATGTCTTCACCTACCGGTCTC
AAGATGGACGGCACAGTGTTCCACCCAGATGACTTCCTGTTGGAGGCACTGCCTCCTTCT
TTTTGCAGCAGTAATGACAAAGTGAGCCATGGGCAGCTAACAGAAGTGGAGCAAAACTGG
AGCAACATGGCACTGGAGCTCCGCACTCTAAACGGGAAAAACTCCTCCTACCCTCCTACT
CTTCCTTCCCCTCCCAACTCCTCCTCTTCTTCTCTTCAGGAGGAGACAAACCCCCCGCTT
CCCCAAGGGGAGCAACACTCCATGTTCCCCACTCTGCCTCGTCATACTCCTCTCTATGCT
CTCACTCCAAAGGAGGCCCTGGATGAACCCCCTGTTCCCTCGTGTAAGGTTCCGCATGAT
GATGTCACCGTGGTTACCAAGGCAGAGATGCATTGGCCTCCTGCTTCTGCTACAACAGAT
ATCCGGAAGCCAAGTCGGGCAAGCAAGTGCAGCGTCAGAGCACGTCCCGATGACCTGGCG
GTGTAG

>Fr-gja3like-XM_003971206
ATGGGGGACTGGAACTTGTTGGGGAAGCTGCTGGAGAGTGCCCAGGAACACTCCACTGTT
GTGGGAAAAGTCTGGCTGACAGTGCTGTTCATCTTCCGTATCCTGGTGCTGGGAACTGCC
GCGGAGAAGGTGTGGGGGGATGAACAGTCCGGCTTCACATGCGACACCAAGCAGCCCGGT
TGTCAGAACGTTTGCTACGACAAGACTTTCCCCATTTCTCACATCCGCTTCTGGGTGATG
CAGATCATTTTTGTCTCCACGCCCACCCTTATCTATTTGGGCCACATCCTTCATTTGGTT
CGCATGGAGGAAAAACAGAAGCAGAAAGAGAAGGACCTCGCAGCCCTGTCTGAAAAGCAG
GAGCAGTTGCTTGGCAACAAGCCAAAGAAGGCCTCAATTAAAGACAACCAGGGCCACGTG
CGTTTGCAAGGAGCCCTGCTGCGAACTTACGTCTTCAACATTATCTTCAAGACCCTGTTT
GAAGTGGCCTTTATTGTAGCTCAGTACTTCCTCTATGGTTTTGAGCTAAAGCCGATGTAC
ACCTGCGACCGCTGGCCTTGTCCCAATATGGTGAATTGCTACATTTCGCGGCCCACCGAG
AAGACAGTCTTCATCCTCTTCATGCTGGCTGTGGCCTCCATCTCGCTGCTGCTCAACCTG
GTGGAAATGTACCACCTGGGTTTCACTAAGTGCCATCAGGGTCTTCGATACAGGCGATCA
AGGGTCAAAAACCTGCCTCCCAAGGCCCTGCCCGAGTCCGTCGTGCCCTTTGCCCCCAGC
TACAACTACTTCTCCGGTCATCCCGCGGTGCCGGAGCCGTTTTCATCTAACTCAAAATAC
AGCGTGACAGAGCCCAGCTCCGCTTACAGCCCCTACAGCAGTAAGGCTGTCTACAAGCAG
AACAGAGACAATCTGGCCGTGGAGAGGAAAGGAAAATCCGAGGACGAGATCGTGATGGAG
AGGAAACCCTCCTCTCCTGCTTTGGAGATGTCCGTCGACAATCAGCGCCGAAACAGTCAG
TCAAGCAAGCACAGCAAGAGCAGACTGGACGACCTAAAGATCTAA

>Fr-gja3like-XM_003970457
This record has been removed from GenBank as a result of genome annotation process (July 2019). This sequence gives a 100% identical hit with gjb1like-XM_029847578, which is an erroneous identification (remark as of Oct 31, 2019). The reason for the erroneous identification could be that "gja3like" and "gjb1like" are closely located in the genome.
ATGGGGGACTGGAACCTGCTGGGAAAACTTCTGGAAAAAGCCCAGGAGCACTCCACCGTC
GTGGGCAAAGTGTGGCTCACCGTCCTCTTCATTTTCCGTATCCTGATCCTCAGCGCTGCC
ACCGAGAAGGTGTGGGGCGACGAGCAGTCGGGCTTCACCTGCGACACCAGGCAGCCCGGT
TGCGAGAACGTCTGCTACGACATCACATTCCCCATCTCCCACGTCCGTTTCTGGGTGCTG
CAGATCATCTTCGTGTCGACGCCGTCGCTGATTTACCTGGGACACATTCTCCACCTGGTG
CGGATGGAGGAGAAGCAGAAGGAGAAAGAGCGGGTGCGACTGTCGCGGAAGCAGGGCCTG
CTGGCGTCCAAGCACAGAAAGCCCCTGGTGAGGGACGAGAAGGGCCGAGTGCGCCTGCAG
GGGGAGCTTCTGCGCACGTACGTCTTTAACGTGATCTTCAAAACTCTGTTTGAGGTGGGC
TTCATCGTGGCTCAGTATTTGCTGTATGGCTTTGAGCTGAAGCCCATGTACACATGTAAC
AGACCCCCCTGCCCCAACGTGGTCAACTGCTACATTTCCCGGCCCACAGAGAAGACCATC
TTCATCATCTTCATGCTGGGAGTGGCCAGCATCTCCCTGCTCCTAAATCTCATTGAGGTC
TATCACCTGGGCTTCACCAAGTGCCGCCAGGGTCTCACCTTTAGGAGGCAGCACCAGCTC
TCCGAGGGGGATTCTCAAGGAGCCCAGCGAGGCCTCGGTGCCCTTTGCGCCCAGCTATGGC
GAGTACTTCCAAGGACACCACCCGGTGCAGCCGACCTACCCCCCCGTGCCCAGCTACAAC
CTCTCCCCGCTGCCTGACGGCACCGAGTCGTCCTTCCATCCTTACAACAGCAAGGCGGCC
TATAAACAGAACAAGGACAACCTGCTGGTGGAGCGGGGCGGCAGCAAGCCAGAGGAGCAC
GATCTGAAAGGAAAGAAGGAGCCGGGTTCGGCCCCCGAGTCACCTACGCAGGTCACGTTG
AGCCGCGGCGCCAAACACGCCAGCAACAAGACTAGAATAGACGATCTGAAGATATGA

>Fr-gja4-37-XM_011609056
ATGTCAAGAGGTGATTGGTCCTTCCTGGAGAACCTGCTGGAGGAGGGCCAGGAGTACTCT
ACGGGCATCGGCCGCGTCTGGCTCACGGTGCTCTTCCTGTTTCGCATGCTCGTGCTGGGG
GCATCGGCAGAGTCCGCCTGGGATGACGAGCAAGCCAATTTCATCTGCAACACGCATCAG
CCCGGCTGCACCAACGTGTGCTACGACAAAGCCTTCCCCATCTCCCACTTCCGCTACTTT
GTCCTCCAGATCATCTTTGTTTCCACGCCGACCATCTTCTACTTCGGATACGTCGCTTTG

CGGGTCAGGAAGATCAATAAAGACGTGGAGGGCAGCTCTGATGAAGGTCAGAGAGGAGGG
ATGGCGAAGGAGACGGACAGTAACTCTGCAACGAAACGCAGCTCAGAGGAGAAGAAACTA
GAGGAAGTGAGGAAAAGCAGAAAAGCTGATAAGGAACTTCCTGAGGCACCTAAGCTGAAA
GGCAGACTGCTGTGTGCGTACACCCTCAGCATCCTCTTAAAGGTCCTCCTAGAAGGTGGC
TTCATGACAGGCCTGTATTTCCTGTACAACGGCTTCTACATCGCAGCAAAGTTCGAGTGT
CAAAGGAACCCTTGTCCCCACACGGTGGACTGCTTCGTCTCGCGGCCCACGGAGAAGACC
ATCTTTGTGTTATACACTCAGGTCATCTCTGGCGTCTCCCTGCTCCTCAACCTGGTGGAG
CTCCTCCACCTTCTGCAGCTAGCCGTCGCTCACCGGCTGGAGAAAAGCCACGGTCACCGC
GGCGTCTACCTGCCTCCCGCTGAGCAGGCGACCGTGGAGGCCGCGCGAATCCAAATGGAG
GTGTTACAGTCCAGTAAAGCAGGGAGCAACGGCGACCTTCCAACCCGGCATGAGGTGGGG
CGTCACACCAATCCCTGCGAGAGTTCCGGGGAACCAGGGATCGAGGTGAACAGGGGACCC
GGAGAGCCTGGGGACGACCTCCTCCCTAGTTATGTGACTTGCGTTGAAGCCACGAGGGCT
ATGCTTTCACCCAGAGTCCATTATAAGAAGAACACAGTCCAGAGTCCGAAAAGCACCAAG
GCAGCTCAGAAAGGACATTCAAAACAGAAACATTATGTATGA

>Fr-gja5-40-XM_003961811
ATGGGTGACTGGAGCCTCCTGGGAAACTTTCTAGAAGAGGTCCAGGAACACTCCACCTCG
GTGGGAAAGGTCTGGCTCACCGTCTTGTTCATCTTCCGGATCTTGGTGCTGGGCACGGCC
GCCGAGTCCTCCTGGGGTGACGAGCAGAGCGACTTCCTGTGCGACACGCAGCAGCCAGGT
TGCACCAACGTCTGCTACGACAGCGCCTTCCCCATCGCCCATATCCGCTACTGGGTGCTG
CAGATTGTGTTTGTCTCCACGCCGTCCCTCATCTACATGGGTCACGCCATGCACACCGTG
CGCCGAGAGGAGAAACAGCGCAGGAGGGAGCAAGAGGAGAGGGAAGCGAGGGCGGAAAGT
GGAGGAAGCCTGGAGGAGAAGGAATTCCTCCAACAGAAGGAGAGCGAAAGAGCCCCGGCG
TCGGGCGGGACCAGCCGGGTTCAACTGAGAGGAGCCCTGCTGCAAACGTACATACTCAGC
ATCATGATCCGCACGGTGATGGAGGTGACGTTTATTGTGGTGCAGTACCTGATGTACGGG
GTGTTCCTCAATGCGTTGTACCTGTGCAAGGCCTGGCCCTGTCCAAACCCGGTCAACTGC
TACATGTCCAGGACCACAGAGAAGAACGTCTTCATCGTCTTCATGCTGGTGGTGGCGGGT
GTGTCTCTGCTGCTCTCCGTGTTGGAGCTCTACCACCTCGGCTGGAGGAGCGTCAGAAGA
CACCTACGCAATAAGATGAGCGAAAAGAGCAACCACAGAACTGTGACAGTGGCTGTGTCC
ACGGCCTTGGAGCCCAACAGTCCACCACAGCCTTCGCTTTCCTGCACCCCACCCCCAGAT
TTCAGCCAGTGCCTTGCAGCCTCTGGATCCATGAATGCCATAACGTCCATGGCCGCTCAC
CCCTTCAACAACAGGATGGCGCTGCAGCAGAACTCGGTCAACCTGGCCACCGAACAGCAT
CACAGCTGCGACAACCTGGAGGACGAGTCAGACTTCCTGAGGATCAGATACGACCAACTA
CCCATGGAGCTGCCCCAAAGCTGCTCGCCATCCCCCCTCCTGCAGTCCAGCTACACGAGG
GACAAACGGCGCCTGAGCAAGACCAGCGGGAGCAGCAGCAGACCTCGTCCCGATGATCTT
GCGGTGTAG

>Fr-gja5like-XM_011603067 Modified. The prediction and/or sequencing/assembly does not seem to be correct in cysteine-encoding signature area of the second extracellular loop. Underlined+italics: 13 nucleotides removed. N: added nucleotides to keep the open reading frame to follow the general pattern of connexins. without these Ns, the sequence must probably be regarded as a pseudogene. Splice sites.
ATGGCAGACTGGAGCTTACTGGGGAACTTCCTGGAGGAGGTGCAGGAGCATTCCACCTCT
GTTGGAAAGGTGTGGTTGACCATCCTCTTTATCTTCCGGATCCTTGTGCTGGGGACCGCC
GCCGAGTCCTCATGGGGAGACGAGCAAGAAGATTTCAACTGTGACACAGAACAGCCAGGC
TGCGAGAACGTTTGTTATGACCGAGCCTTCCCAATAGCACATATACGATACTGGGTGCTG
CAGATTGTGTTTGTGTCCACGCCCAGCCTGATCTACATGGGTCACGCCATGCACAGGGTC
CGCAGGGAGGAGAAGAGGAGGAGCCGGGAGGAGGGAGGCGGGGAGGGGAGAGGGGGAGAG
GAGGACCCGGGCGGCGGCGGACGAGGAAATGACAGTGGGGAAGAAGATGAGAAAGGTGGG
AGAGAAGTGGAGAAGCACGGAGAGAAAGAGGGTGGAGGTCGCTTGCGTTTGAGGGGAGCG
CTACTACAGACCTATGTGCTGAGTATACTGATACGAAGCGTCATGGAGGTGGTGTTTCTC
ACTCTCCAGTATTTAATGTACGGGATCTTCCTTAATCCTCTGTATGTCTGCAA*GG*CTTGN
CCGTGCCCTCAGCCGGGNAACTGTNATGTCTCCAGGCCAACAGAGAANAATGTCTTTATT
GTGTTCATGCTGGCCGTTTCCGGTGTTTCTCTGGTCCTCAGCGTGCTAGAGCTGCAACAC
CTGGCGTGGAGGCACTGCTGTAGAAAGACGGCGGCGGCTAACAAGGCCTCGCTAGGCCGA
CAGGTCTCTCTGTCCCCTCCACCACAGTCCACCCCACCTCCAGAATTCAGCCAGTGCATG
ATGGGCTCCACACACTTCCTTCCTCTTGCGTTCCCCAACCACCACCTGGCACACCAACAG
AACTCAGAGAACATGGCCACCGAGAAGCACAAAATAGCCGCCGCCGTAGAGGAGGCCACC
CTCCTGCAGATGGGCTGCTACTCACACGGATGGCAGAAGACCAACCCCAGTCAGATCCAG
GACGACCCCTACCTGAGGAACGACAACAGTCGCTACGGTCCCGGCAGCAGGGAGATCAGC
TGTTCACAGATCCAAAATGGAGGCTCCGACAGGCTCTTGCTTTGCCCCAGCGGCGCTCAC
AATCAGAAAGACAAGCGGAGATTCAGCAGAACCAGCGGCACCAGCAGCCGAACAAGAGCG
GACGACCTGTCCGTTTAA

>Fr-gja8-50-XM_003961810
ATGGGTGACTGGAGCTTTCTGGGTAATATTTTAGAGGAAGTTAACGAGCACTCTACGGTG
ATCGGCCGGGTGTGGCTCACGGTCCTCTTCATCTTCCGTATCCTCATCCTGGGCACGGCG

GCGGAGTTTGTATGGGGCGATGAACAGTCAGACTATGTCTGCAACACAAAGCAGCCTGGT
TGCGAGAACGTGTGCTACGACGAGGCCTTCCCGATCTCCCACATCCGCCTGTGGGTGCTG
CAGATCATCTTTGTGTCCACACCATCTCTGGTGTACGTGGGTCATGCTGTGCACCACGTC
CACATGGAGGAGAAACGCAAGGAGAGAGAGGAGGCAGAACTCAGCCGGCAGCAGGAGCTG
AGCGAGGAACGTCTCCCTTTGGCGCCCGATCAGGGTAGTGTCCGCACCACTAAGGAGACC
AGCACAAAGGGAAGCAAGAAGTTCAGGCTGGAGGGCACCCTGCTGAGGACCTACATCTGC
CACATCATCTTCAAAACACTGTTTGAAGTGGGGTTTGTGGTGGGCCAGTACTTCCTGTAT
GGCTTTCGCATTCTGCCACTGTACAAATGCAGCCGCTGGCCTTGCCCTAACACGGTGGAC
TGCTTCGTGTCTCGTCCCACTGAGAAGACTGTCTTCATCATCTTCATGCTGGCCGTGGCC
TGTGTCTCTCTCTTCCTCAACTTTGTGGAGATTAGTCACTTGGGCCTGAAGAAGATTCGC
TTCGTCTTTCGCAAGCCCGTGCCGGCCCCAGCCCAAGGCGAGGGCTCGGCCCCGCTTCCA
GCACCAGGGAAGAGCCTGCCTTCCCTAGCTGTGCCCTCCCTGCAGAGAGTGAAAGGTTAC
CGGCTGCTGGAGGAGGAGAAAGCTCCCCCAACTCATCTCTACCCTCTGGCTGAGGTGGGC
ATGGAGGCCGGCAGAGGGACCCCACCCTTCCAGGGGCTAGAGGAGAAGTCCCAGGAGGTG
CTACCCATGGAGGACATCTCTAAGGTGTATGACGAGACTCTGCCCTCCTACACCCAGACC
ACTGAGACTGGGGGGGTGGTGGTGGTGAGAGAGGAGGCAGAGGAAGTGGTGAACGTGGAG
GAGGTAGCTGAAGCGGAGGCCACGGATACGATAGAAGACACCAGACCGTTGAGCCGACTG
AGTAAAGCCAGCAGCAGGGCCAGGTCAGACGATCTCACAGTATGA

>Fr-gja9-59-XM_003965660
ATGGGAGACTGGAACTTCCTCGGAGGGATTTTGGAGGAGGTGCACATTCACTCCACCATG
GTGGGCAAGATCTGGCTCACCCTTCTCTTCGTTTTCCGCATGCTGGTCCTCGGAGTGGCG
GCCGAAGACGTGTGGAACGACGAGCAGGCTGACTTCATTTGCAACACCGAGCAGCCGGGA
TGCAGGAACGTTTGCTACGACCTGGCTTTTCCCATCTCCCTCATCCGCTACTGGGTGCTG
CAAGTTATCTTCGTGTCCTCCCCCTCGCTGGTTTACATGGGCCACGCTCTGTACAGACTG
CGGGCCCTGGAGAAAGCACGGCAGAGAAAGAAAGTCCTGCTGAGGAAGGAGCTGGAGTTG
GTGGACGTGGATCTGGCCGAAGCCAGGAAGAGGATTGAACGGGAGGTGAAGCAGCTCGAC
CAGGGCAAGCTGAACAAAGCTCCGCTCCGGGGATCCCTGTTGCGCACTTATGTGGCACAT
GTGGTCACCCGGTCCGTTGTTGAAGTGGGCTTCATGACGGGCCAATATGTCCTTTATGGG
TTTCACCTCTACCCACTTTTCAAGTGCGAGCGGGATCCTTGTCCTAATGCTGTGGACTGT
TACGTCTCCAGGCCGACGGAGAAAAGCGTCTTCATGGTCTTCATGCAATTCATCGCCGCA
ATTTCCCTCTTCCTTAACATTTTGGAGATGGCGTATCTTGGCTACAAGTGGATTAAACAG
GGCATCTTGGATCTTTACCCGCAATTACAGGATGAGCTCGATGATGACTTTATCTCTAAG
GGGGGAAAGGAATCTGTTGCGCAACTCTGCGCCAGTGCGGGCCGGAAGATGACGATTACA
TTTTCCCCAATTGATGGCAACCTAATGCAAGGAGCCGTTGGTCCAGCAGCGGCTCTTCCT
CTCCTGAGTGACCTGTCCAATCAACCACATCTGGGGGGTTCCACATGTTTGGCCCAAAGC
CCAAAAGGGCGCAGCACGATCCATGCGAAGCTTTCTCTCCCTCACAGCCAGGAAAAAGAG
CGCAGTGACAGTGGCAGCCCAGACTATCCAAAGTGCCAACAGAAACCAGCCCCCCCGCTG
CCAGTGAGCGTACCGCGGAGGCCTTGGAGGGCTCATTCTTTCAAATGCGCCACGGTGCCG
GAGGGGAAGAGTTCTGACACGGATTCAAACGAGGAGCTGTGCGCTCAGACGTCGCCAAAC
CAGCGCGTCCGCCATCTCAGCCGTAGCTCCACGGCGGAGTCTCTGCATGGCTCCAGCTCG
GGCTGCGCGCACAGCCCCACGCTGCCCCCCTCTTACTGCAAAACATCATCACCGAGCAAA
AGCAGCAGCAGTCGGGAGCCAGACCTGCAAATTTAA

>Fr-gja9like-XM_003968854
ATGGGAGACTGGAATTTCCTTGGAGGAATCTTAGAGGAGGTGCATATCCACTCCACCATG
GTGGGCAAGATCTGGCTGACCATCCTGTTCATATTTCGGATGTTAGTACTGGGAGTTGCT
GCGGAGGATGTGTGGAATGACGAACAGTCTGATTTCATCTGCAACACCGACCAGCCTGGT
TGTCGAAATGTCTGTTATGACCAGGCTTTCCCCATCTCCCTCATTCGATACTGGGTGCTT
CAGGTGATTTTCGTGTCCTCCCCCTCCTTGGTCTACATGGGCCATGCCATTTATCAACTG
CGAGCTCTGGAGAAGGAACGCCACTGTAAGAAGGTGGCATTACGCCGGGAGATGGAAGCA
GTGGATGTGGAATTGGTGGAGGTAAGGAAGAGAATTGAAAAAGAGATGAGGCAGCTAGAG
CAGGGCAAACTCAACAAGGCACCACTGAGAGGGTCTCTATTGTGTACTTATGTGGCCCAC
ATTGTGACTCGCTCGTTGGTAGAGGTCAGCTTCATGATGGGTCAGTACATCTTGTATGGA
CACCACCTGAAACCTCTTTACAAGTGTGAGCGAGAGCCGTGCCCAAATGTGGTGGACTGC
TTTGTGTCCAGACCCACAGAGAAAACAGTTTTTATGATGTTCATGCAAGCCATTGCCTGC
CTCTCACTCTTTCTCAGTCTTCTTGAGATTATCCACCTGGGATTTAAGAAGCTTAAGAAG
TGTATCTTGAACTTCTTCCCACACCTGAAAGATGATCCTGATGAATTTTACATTAGCAAG
TCAAAAAAGAACTCAGTCGTGCATCAGGTGTGTGCTGGAACATCTGTAGCTGGAAAGACA
ACTATTCCCACAGCGCCATGTGGATACACGTTGCTGATGGAGAAGCAGGGCAATGGGCCC
AACTACTCGCTTCTCAATGCCTCCTCTGCTTTCATTCCAATACAAGGGGACCCTGGTGCA
AAGTCAGATCGGCGTAAGGATGGCAAGGAGGGAATTCCAAGTCCTACAGAACAAAACAGT
AATTCCAACAACACCAGCAGCGACACACATTCTCTTCCTGTGGATAAACATGAGGAGCCA
GAGGAGCCCCTGGTGACCTCTGAATATCCTACGCTCCCTGTTGCCGACGCCACCTCCTGC
CCAACCCTGTCAGGCATTACCAGGAAGTCACGGAGGATCAGCCCACCTTGGAACTGCTCC
ACTCTACCAGAAGGGAATGGCTCAGACAGTGGGGATTCCTACCTGGGGGGCAACAGCATC
AAGCAACGCAGCAGCTGTGTTGGGCCCCGTGCAAGGATTCTCTCCAAATCAGACACTAAA
AAGCCTGGCAGACCACAAAGCCCGGACTCAGCAGGTGAGCTGAGTTCGGCGTCTCGTCAC

AGCAATGAGAGTAACAGCCCCACAGCTTCACCCCCAAACCGCAGAGTGTCAGCAGCAAGT
AGTGCCAGCAGCCGGCGAGCTCCGACTGACCTACAGATATAA

>Fr-gja10-62-XM_003971382
ATGGGGGACTGGAACTTATTAGGAAGTATTTTAGAAGAAGTCCACATTCATTCCACCATT
GTGGGCAAAATCTGGCTCACCATCCTCTTCATCTTCCGGATGCTTGTGCTTGGGGTTGCG
GCCGAGGATGTTTGGGACGATGAGCAGAGTGAATTTGTTTGCAACACGGAGCAACCTGGG
TGCAAGAACGTCTGCTACGACCAGGCTTTCCCCGTCTCCCTGATCCGTTATTGGGTCCTG
CAGATTATTTTTGTATCCTCTCCATCACTGGTCTACATGGGACATGCACTGTATCGCCTG
AGGACCCTTGAGAAAGAGCGGCACAGGAGGAAAGTCTGCCTGAAAGCTGAGCTGGAGGGT
ACAGACCCCATCCAGGAGGATCACAAGAGGATCGAGCGAGAACTCAGGAAACTAGATGAA
CAGAAGAGAGTGAGGAAGGCCCCTCTAAGAGGCTCCTTGCTTCGCACATATGTTCTCCAT
ATCTTAACTAGGTCCGCAGTAGAGGTGGGTTTTATCGTAGGACAATGTGCTCTGTACGGC
CTTGGACTGTCTCCCTTGTACAAATGTGCCAGACTGCCGTGTCCCAACAGCGTCGACTGT
TTCGTCTCTCGGCCTACAGAAAAGAACATTTTCATGGTCTTCATGCTAGTCATTGCTGGT
GTTTCGTTGTTCCTCAACATTCTGGAGATTTTTCATCTGGGTGTGAAGAGGATTAAACAA
AGTTTGTATGGATATAAATACCGAGATGACGAGAGCGTGTACCGCTCAAAGAAGAACTCC
ACGGTACAGCAAGTGTGTGTTCTCACAAATTCGTCACCACAGAGGCTGGTGCAGCTCACA
CAGATGACTTGTTCGGCTCTGCCTGACACTCACGGGGAGACTCTGGCAATAAATGTGTCC
CACCAGAACCAGGAAGGATCTGGCGCCACCAACCAGCATCCGTCACACATTGGCATGCCT
GCCCAGGGTCTTCATCAGGTGCCACCTGTCGAGCAGCAATGCGCTGTAGGTACAAGGAAG
CCGTCGTATAGCAGCGAAGAATCCAGCGAACCTCACGTGAGGCCACAATATGCAGGACCC
AGAGCCACCCTCGTCGCCAGCCACATGGAGATCCCAGCAGCCCTGAGGAACCCACAGAGG
AAAATGAGCAGAGTAAGTGTTTATAAGGACCTTAGCGACATGAGTGACTCGGCAGAGAGC
GAGCCCCACCCCACGGTCCGGAAGTGCAGCTTTATGTCCCGGGGTCTGTCCGATGGAAAG
CTGTCCTCCCCGTCCGACAGCACCGACAGCCACAGCGGAAGTGATGCTGAAGCCCAGCAT
CTCAACCAAGCCGAGGGTTCAGTGGTGACCCCCCCACCACCGGCCAGCGGGAGGAGGATG
TCCATGGTTAGTAGACAATTTTCACAGTCCACAACAAAACTTCACAAACCTGATTCTGGT
GTAGATAGTTAG

>Fr-gja10like-XM_011619942 Extended according to ENSTRUT00000004551 (underlined)
ATGGGGGACTGGAACCTGCTTGGCAGCATCCTAGAAGAGGTTCACATACATTCCACCATC
GTGGGCAAAATATGGCTGACCATACTCTTCATTTTCCGCATGCTGATATTGGGAGCAGCT
GCTGAAGATGTGTGGGATGATGAGCTGTCTGAGTTCATCTGTAACACTGACCAACCAGGA
TGCAAAGCTGTCTGCTATGACCGTGCCTTCCCTATCTCGCTTATTCGCTTCTGGGTCCTG
CAGGTTATCTTTGTCTCTGCACCCTCTTTGGTCTATATGGGCCATGCCCTTTATTGCATC
CGAGCTCTTGAGAAAGAGCGCCACCGCAGGCGTATCCAGCTAAAGGAGGAGTTGGATGAG
GCTGAATTAGCAATGGAGGAACAGAGGCGTGCAGAGAGAGAATTGAGAAGGCTGGATGAA
CAGAAGAAAGTGAAGAAGGCCCCTCTTAAAGGTTCTTTGTTGAGAACTTACATTATCCAT
ATCCTTACTCGCTCTGTGGTGGAAATCTGTTTCCTTCTTGGCCAGTATTTCCTCTACGGT
GTTCAATTGGACCCACTTTATAAGTGTGAGAGGATGCCCTGTCCCAACAGTGTAGACTGT
TACATCTCTAGGCCCACAGAGAAGAGCATTTTCATGGTCTTCATGATTGCCATTGCTGGT
ATTTCACTTTTACTCAACATTTTTGAAATATCACACCTAGGCATAAGGAAAATTAAAGGG
ATACTATATGGAGAGCTATACAGAGACGATGACAGTTTGATTTTCAAGTCCAAGAAGAAA
GCCTCCTTACCACAACTTTGTGTCATTAGCAGTGTATCACCTCACAATGGGCCTTTGACT
CAAACACTGAAAGTGATTCCAGAGGTAGCCATGAAATTTTCTTATTGTAATGCTGGCCTT
AAATCCAGCCAGGACATACAAAGACCCAACCGTAGCCTGCAACCTAAGCCAACTGGGTGT
GTTGAGAACTTACAAATTCAAGCCCCCCTAATCAGTGAGGAAATGAACACTTTCAAGGCA
GAAAACAACCCAGAGTGGATCTCTTCTTTTTCAATTGGTGGAGGTAGTACAACAAACCAC
GAAGATGATACACGTGATGTAGGACTCCCTCTTTCTAACCATTCGGAAACTCAGTTATTA
CATAACATCCTAAGGTCAAGGGATGCACAAAAAGAGGAGCGAAAGGATTCAGTGATGAAT
GAAGTCCTCATACCAAACCCCAGGAAGACGAGCTTTTTGAACAGACCACCATCAGAGAGC
TTGTCTTCTATCAGTAACTCTACAAGTCCATCCTTACATACCTCAGAGGAATCTGATGAA
CTGGGCTCATTACAGGGAGACATGCCAATAATGCCACCGGCTGGCCGAAGAATGTCTATG
GCAAGTATAGCGTCATTGGTTTCTCATGGGTTGAATAGCTCAAACACATTTTTCACATTT
TTGGTATGA

>Fr-32.7like-XM_003976250 Splice site.
ATGGGCGAGTGGGATTTGTTGGGCCGCCTGTTGGATAAAGTGCAGAGTCACTCCACAGTT
CTGGGCAAGGTTTGGCTCACCGTGCTTTTTGTCTTCCGCATCCTGGTGCTGCAGACCGCC
GCCGACAAGGTGTGGGGTGATGAGCAGTCTGACTTTGTCTGCAACACTCAGCAGCCGGGC
TGTGAGAACGTCTGCTACGACCTCGCGTTCCCCATCTCTCACGTGCGCTTCTGGTTTCTT
CAGATTATTGCCATAGCGACGCCGAAGCTGCTCTACCTCGGCCACGTCCTCCACGTGATC
CACATTGAGAAGAAGGAGAAGGAAAAGATGAAGAAACAGGCCGAGTTGGATGCTCAGGCG
TGTCTGTTCCTCAGGACCTACAAAGTTCCCAAGTACATCAAAAGCTCTGGCAAGATCAGC
ATCCGCGGCCGCCTCCTCCGCAGTTACACCTTCCACCTGCTGGCCAAGATCCTCCTGGAA
GTTGTCTTCATCGCCGGCCAGTACTTCCTCTTTGGCTTCACCCTGGACTCTCGCTACGTC
TGCCAGCGCCACCCCTGCCCCCACAAGGTGGACTGCTTCCTGTCCAGGCCTACGGAGAAG

TCGGTCATCATCTGGTTCATGCTGGTGGCGGCAGTCGTCTCCCTGGCCCTCAGCCTGGTG
GAGCTGTTCTACCTGTGCGTGAAAGCGACGAAGGAGTGCATGGCGAGGAGGCAGGACTAC
ACGGTGACGCCCGTGACGCCCCCGGTTTCGGGAAGGAAAGCTTTCAAAATCTCCGATGAG
ATGATCCAAAATTGTATCAACCTGGAGCTGGAGCAGCATAAAGAGCAGAGGGGGGGGAAG
AGGATCACCGGCGGGGCCAACGAGGTCCCCAGTATCATCTCACCTGACAGCAAGAGCAAG
GGGGAGGTCCGTATCTGA

>Fr-32.2like-XM_003976251
ATGGGTGAGTGGAGCTTTCTGTCCTCTCTGCTGGACAAGGTCCAGTCTCATTCCTCTGTC
ATCGGGAAGGTCTGGCTCAGCGTGGTCTTCATCTTCAGGATCATGATTATTGGAGCTGGA
GCTGATAAGGTTTGGGGCGATGAGCAGTCCAATATGATCTGTAACACCAAGCAGCCCGGC
TGTAAGAACGTCTGCTACGATCACGCCTTCCCGATCTCGCACATTCGATTCTGGGTCCTC
CAGATCATCTTCGTCACCACACCCACGCTGGTCTACCTGGGACACGTCCTGCACGTCATC
CACAAAGAGAATAAGATGAGAGAATACATGAAGACTCACAGTCAGAGCAACCTTGCCAAG
TACCCCAAGTACTCTGATGAGAAAGGCCACGTGGAGCTGAAGGGCAACCTTCTGGGCACC
TACATAACCTCCATATTTTTCCGAATCATCCTGGAGATCGCCTTCATCGTGGGGCAGTAT
TACCTGTACGGGTTCATTATGGACCCTAAGGTGGTCTGCTCCCGGGCCCCCTGCCCCTTC
ACCGTGGAGTGTTTTATGTCCCGTCCCACCGAGAAGACCATCTTCATCCTCTTCATGCTC
GCAGTCTCTTGTGCTTCGCTTTTACTGAATGTAGCAGAACTCTTTTACTTGTTGCATTTC
CGCTTAAAGAAAAGGTCCAAAAGTCTTCCGGCTTTGTCTCTCGCCATTCACCCACACTTC
AACAGTGAGAGCAAGGCCTAG

>Fr-32.2like-XM_011617171
ATGGGAGACTGGGGATTCTTATCGTCCTTGCTGGACAAAGTCCAGTCCCACTCCACGGTC
ATCGGAAAGATCTGGATGAGCGTCCTCTTCCTGTTCAGGATCATGGTTTTGGGCGCCGGC
GCCGAGAGCGTTTGGGGCGACGAGCAGTCGGGTTTCATCTGCAACACTCAGCAACCCGGT
TGCGAGAACGTCTGCTACGACTGGACCTTCCCAATTTCGCACATTCGCTTCTGGGTCCTC
CAGATCATCTTTGTGTCCACGCCGACGCTGGTGTACCTGGGCCACGCCATGCACATCATC
CACAAGGAGAACAAGCTGAGGGAGAAGCTGCTGAGCCCCGGCGGGCCCCGCCTTGCTAAG
GTGCCCAAGTACACTGACGAAAAAGGGAAGGTGAAGATCAAAGGAAACCTGCTGGGGAGC
TACCTGACCCAGCTCGTGTTCAAGATCCTCATCGAGGCGGCCTTCATCGTGGGCCAGTAC
TACCTGTACGGCTTCATCATGGTGCCAATGTTCCCTTGTTCCAGGGAGCCCTGCCCCTTC
ACCGTGGAGTGCTACATGTCCCGTCCCACCGAGAAGACCATCTTCATCATCTTCATGTTG
GTGGTGGGCTGCGTCTCCCTGCTCCTCAACGTGGTCGAGGTGCTCTACCTCCTGTGCACC
AGGCTCAAATGTGCCTCCAGGTCCCGCGCACAGAAGCTCACGTCGGCGGAACATCCCGCC
ACCCTGCCCGCTCCCAAATGGCCGACGGTGGACGATGCGCTCATGCAGAACAAGATAAAC
CTGGAGAAAGAACGCGGTCAGAGCATCGGCGGGAACCTGGATGGCGCCAAGGAGGAGACG
CAGCTGCTGCGCCATTAA

>Fr-gjb1like-XM_011610767
ATGAACTGGGGAACCTTTTACGCCCTGATCAGCGGCGTAAACAGGCACTCGACCGGCATC
GGGAGGGTTTGGCTCTCCGTCATCTTCGTCTTCCGAATCCTGGTGTTGGTGGTGGCTGCT
GAGAGCGTTTGGGGAGACGAGAAGTCGGGCTTCACCTGCAACACCCAGCAGCCTGGCTGC
AACAGCGTCTGCTACGACCAGTTCTTCCCCATCTCGCACATCCGCCTTTGGGCTCTGCAG
CTGATCCTGGTCTCCACCCCGGCCCTGCTGGTGGCCATGCACGTAGCCCACAGACGGCAC
ATCGACAAGAAGATCCTGAAGAGGGCCGGCCGGGGCACCCCCAAAGACCTGGAGCAGATC
AAGAACCAGAGGTTCCAGATCACCGGAGCTCTGTGGTGGACGTACATGATCAGCATCATC
TTCAGGATCGTCTTTGAGGTGGCTTTTCTCTACATCTTCTACCTGATCTATCCAGGTTTC
AAAATGGTGCGTTTGGTCAAGTGCGACTCGTACCCCTGCCCCAACACCGTGGACTGTTTT
GTGTCCAGACCCACAGAGAAGACCATATTTACCGTGTTCATGCTGGGGGTCTCGGGGGTG
TGTGTGCTTCTGAACTTGGCTGAGATGGTCTACCTCATCGGCCGGGCCTGCAGGCAGTGC
ATCAGAGGCTCGGAAGAAACCTCCAAAGTCCCCTGGATCAGTCAAAAATTGTCCTCTTAC
AGGCAAAATGAGATTAACGAACTGATATTGGACCATCCCCTCAGGTCAAAGTTCGGCGTG
ACCAAAAAGAAGCCCAGCTGA

>Fr-gjb1like-XM_003971205
ATGAACTGGGCATCATTTTACGCCGTCATCAGCGGTGTGAACAGACACTCCACGGGCATC
GGCCGCATCTGGCTTTCTGTGCTCTTTATTTTCCGCATCCTGGTCCTGGTGGTTGCTGCG
GAGAGCGTGTGGGGAGACGAGAAGTCGGGCTTCACCTGCAACACCCAGCAGCCGGGCTGC
AACAGCGTCTGCTACGATCACTTCTTCCCGATCTCCCACATCCGCCTCTGGGCACTCCAG
CTCATCCTGGTCTCCACCCCTGCCCTGCTGGTGGCTATGCATGTGGCTCATCGCCGCCAC
ATCGACAAGAGGCTCTACAAACTGTCAGGGCGGACCAACCCCAAAGATCTGGAGCAGATT
AAGACCCAGAAAATGAAAATCACAGGCGCGCTGTGGTGGACGTACGTCATCAGCCTGCTC
TTTCGCGTTATCTTCGAGGTGACCTTTATGTACCTATTTTACATGATCTACCCCGGTTAC
AAGATGATCCGGCTGGTGAAGTGTGACTCGTACCCCTGTCCCAACACAGTGGACTGCTTT
GTCTCCAGGCCCACAGAGAAGACGGTTTTCACCGTCTTCATGCTGGCTGTGTCAGGGGTC
TGTATTCTGCTCAACATTGCAGAGGTGGTGTTCTTGGTGGGGAAGGCCTGCGGTAAACAT
TTACACCATGCTGGAGACTCAGCCATGGGGGCTTGGATCCAACAAAAGCTCTGCTTCCTC

TAG

>Fr-gjb2like-XM_003962228 97% identical to XM_003962227 below
ATGTCTTGGGCCACGCTTTACAGTCAGCTGGGTGGTGTCAACAAACACTCCACCAGCCTG
GGAAAGATCTGGCTTTCTGTCCTCTTCATCTTCCGCGTCACCATTCTGGTTCTGGCCGCT
GAGAAAGTCTGGGGCGACGAACAGTCCGACTTTAAATGCAACACGCAGCAGCCAGGTTGC
AAAAATGTCTGCTACGATCATTTCTTTCCCGTTTCGCACATCCGCCTGTGGTGCCTGCAG
CTGATCTTTGTGTCCACCCCGGCCCTTCTGGTGGCCATGTATGTGGCCTACAGAAAACGT
GGAGAACAGAGAACCGTTATGGCCTCCGGAGGCGATGAGAAGGTGAAGGAGACCGACCTG
CAGATACTGAGGACGAAGCGCCTGCACATCACGGGCCCTCTGTGGTGGACCTACACCTGC
AGCTTGTTCTTCAGATTGCTGTTTGAGGGTGGCTTCATGTACGCTCTGTACTTTATCTAC
GATGGCTTCCAGATGCCGCGACTGGTCAAGTGCGAGCAGTGGCCTTGCCCCAACAAGGTC
GACTGCTTCATCTCCAGGCCAACAGAGAAAACCGTCTTCACCATCTTCATGGTGGTCTCG
TCGGCCATTTGTATGGTTCTCAATGTTGCTGAGCTCTTCTACCTTTTTGCCAAGGCCCTC
ATGCGGTTATCAGCCAGGTCAAAGCAGCGTAAGCGGAGATATACCAGCGAATCAAACTTC
AACCAGGACACGCTTCTGGACAACAGGAGGAATGAAACTTTGTAG

>Fr-gjb2like-XM_003962227 See XM_003962228 above. Either recently duplicated gene, or an assembly error.
ATGTCTTGGGCCACGCTTTACAATCAGCTGGGTGGGGTCAACAAACACTCCACCAGCCTG
GGAAAGATCTGGCTTTCTGTCCTCTTCATCTTCCGCGTCACCATTCTGGTTCTGGCCGCT
GAGAAAGTCTGGGGCGACGAACAGTCCGACTTTAAATGCAACACGCAGCAGCCAGGTTGC
AAAAATGTCTGCTACGATCATTTCTTTCCCGTTTCGCACATCCGCCTGTGGTGCCTGCAG
CTGATCTTTGTGTCCACCCCGGCCCTTCTGGTGGCCATGTATGTGGCCTACAGAAAACGT
GGAGATAAGAGAACCGTTATGGCCTCCGGAGGCGATGAGAAGGTGAAGGAGACCGACCTG
CAGATACTGAGGACGAAGCGCCTGCACATCACGGGCCCTCTGTGGTGGACCTACACCTGC
AGCTTGTTCTTCAGATTGCTGTTTGAGGGTGGCTTCATGTACGCTCTGTACTTTATCTAC
GATGGCTTCCAGATGCCGCGACTGGTCAAGTGCGAGCAGTGGCCTTGCCCCAACAAGGTC
GACTGCTTCATCTCCAGGCCAACAGAGAAAACCGTCTTCACCATCTTCATGGTGGTCTCG
TCGGCCATTTGTATGGTTCTCAATGTTGCTGAGCTCTTCTACCTGATTGCCAAGGCCCTC
ATGCGGTTATCAGCCAGGTCAAAGCAGCGAAAGCAGAGATACAACCGAGAAAACTTCCAC
CGGGACAACACGCTTCTGGAGAACAAGAAGAATGAGAACATGTTCTCTTCAGACTCCACC
AGCAACAGGACCGTGTGCTGA

>Fr-gjb3like-XM_003962552
ATGGACTGGAAGACCTTCCAAGCCCTCCTCAGTGGGGTGAATAAATACTCCACGGCGTTC
GGGAGGGTCTGGCTGTCGGTGGTGTTCGTGTTCAGGGTGATGGTGTACGTGGTGGCGGCA
GAGCGCGTGTGGGGCGACGAGCAGAAGGACTTTGACTGCAACACCAAGCAGCCGGGCTGC
GCTAACGTCTGCTACGACTTCTTCTTCCCCATCTCCCACATCCGCCTGTGGGCCCTGCAG
CTCATCTTCGTCACGTGCCCGTCCTTCATGGTGGTCATGCACGTGGCGTACCGTGACGAC
CGCGAGCGCAAATTCAGGGCCAAGCACGGCGACGGGAAGAAGCTGTACAACAACACGGGC
AAGAAGCACGGCGGCCTGTGGTGGACGTATATGCTGAGCCTGTTCGTGAAGACGGGCATC
GAGGTCGCCTTCCTCTACATCCTCCACCACGTCTACGACAGTTTCTACCTGCCGAGGCTG
GTCAAGTGCGAGGTGTCGCCCTGCCCCAACCAGGTGGACTGCTACATCGGCCACCCCACC
GAGAAGAAGGTCTTCACCTACTTCATGGTTGGAGCCTCGGCCCTCTGCATCATCCTCAAC
ATTTGCGAGATCATTTACCTCATCGCCAAGCGCGTCGTGCGGTGCGCCAACAAGGTCAAG
AGGCACCATCGCAACAGAGCCCCGTGCCCTCCGGAGAACTACAGCGACGACCCCTTCAAC
AACTGCAACGTGACGATGGCGAAGCCGGAGCTGAAGGACAACCCCCCGTCCTTCAGGACC
GCGTGCAAGTCTACATATAAGCTGGACAGTCTTCGGATGAACGACAAGATCCGGGCCTCT
GCCCCCAATCTGTCTGCATGGCCGGTTGCGGGGCCCAGTGTAGGCATAAACGGGCCGACA
GTGGTTGGACTGGAACCCAGCAGGAAACCCCTGAACGCAGACCAGGTGCTGGACTTCAGG
TCGTTGTGTAAGAACCTGGAAGCGGAGTCATCCCGGCTGGACTCTTAA

>Fr-gjb3like-XM_003969117
ATGGATTGGAAGTTTCTTGAGGGTCTCCTCAGCGGAGTCAACAAGTACTCCACTGGCTTC
GGACGCATCTGGCTGTCGGTGGTCTTCGTCTTCCGCGTGCTGGTCTTCGTCGTGGCTGCC
GAGCGGGTCTGGAGCGACGACCAGGGACACTTTGAGTGCAACACCCGTCAGCCAGGCTGC
ACCAACATCTGCTATGACTACTTCTTCCCCATCTCCCACATTCGCCTGTGGGCGCTCCAG
CTGATCTTCATCACCTGCCCCTCCTTCATGGTGGTGCTGCACGTGGCCTACAGGGAAGAA
CGGGAACGCAAGTACAAAGCCAAGCACGGCGAGGACGCCCGGCTGTACGACAACCCAGGC
CAGAAGCACGGCGGTCTGTGGTGGACGTACTTGCTGAGCCTCTTCACCAAGACCACCTTC
GAGATGCTGTTCCTCTACCTGCTCAACTACATCTACGACAGCTTCAAACTGCCCAGGAAA
GTCCAGTGTGACGCGAGTCCCTGCCCCAACCTGGTGGACTGCTACATATCCCGGCCCACT
GAGAAGACGGTTTTCACCTACTTCATGGTGGGTGCCTCGGTGCTGTGCGTGGTGCTCAAC
ATCTGTGAGATCCTCTATCTGATTGCTGCTCGTGTGGTGAATCGGAAGTATCGGGGAAGC
AACCGTGCGTCCTCTAGGAAGGTCCACGCGGCCGCCAGCGCGGGCTGCGATGGTTGCAAG
TCCTCTCTTGTGCATGATTAG

>Fr-gjb4like-XM_011614516 Splice site.
ATGAACTGGTCGGGGCTGGAGAGCCTTCTCAGTGGAGTCAACAAATACTCCACGGCCTTT
GGGAGAATCTGGCTGTCCATGGTGTTTGTGTTCCGTGTGCTGGTGTTTGTGGTGGCAGCA
CAGAGGGTCTGGGGTGATGAGAGCAAGGATTTCGTGTGCAACACTCGACAGCCGGGTTGT
ACCAACATCTGCTACGACCACATCTTCCCCATCTCCCACATCCGTCTCTGGGCTCTGCAG
CTGATCTTCGTCACCTGCCCGTCCTTGATAGTGATGGCTCACGTCAAATTCCGTGAAGGG
AAGGATGCCAAATACGTGGAGCAGCACCACGGCTCTCACCTATACAGCAACCCCGGCAAG
AAGAGAGGGGGGCTGTGGTGGACCTATCTGCTGAGTCTGATCCTCAAAGCTGGATTTGAC
GCCTCGTTTCTTTATATTCTGTACAAGATATATGATGGTTATGACTTGCCCAGGTTGTCG
AAATGTTCGCTGGATCCGTGTCCCAACACGGTCGACTGCTTCATCAGTCGCCCGACAGAG
AAAAAGATCTTCATGTTGTTCATGGTCGTGTCCAGTGCGCTTTGCATTTTCATGTGCCTC
TGCGAAATGCTCTATCTTGTTGGGAAGCGCATCGCCAAACTGGTAAAGATCCGCCACCAG
AACGAACAGATCCTATTTGCTGAGCAGCACGAACTCACCGACATGGTCCCACCCAGATCC
CAGTATCACAAGACTGACCCAACCCTGACGGACAGTCAGCTCAGTTTAAACAGAAAGGAG
AAGGTCAGAGAAGGTACTGTGACGACCACACTGTAA

>Fr-gjb4like-XM_011609061 Splice site.
ATGAACTGGTCTGCACTGGAGGCCCTGATCAGCGGGGTCAACAAGTACTCCACCGTGTTC
GGACGCGTCTGGCTGTCCATGGTCTTCGTTTTCCGAGTGATGGTGTTTGTGGTTGCGGCT
CAGCGGGTGTGGGGCGACGACAGCAAGGACTTTGTCTGCAACACGGCCCAGCCGGGCTGC
AACAACGTGTGCTACGACAGCATCTTCCCCATCTCACACATCCGCCTGTGGGCCCTGCAG
CTCATTTTCGTCACTTGCCCGTCGCTGATGGTGGTGGGCCACGTCAAGTATCGGGAGAAG
AAAGACTCCCAGTACACCACCTCGCACCACGGGAAACACCTGTACGCCAATCCTGGAAAG
AAGCGTGGAGGGTTGTGGTGGACCTACCTGGCGAGTCTGATTTTCAAGGCCGGCTTTGAC
GCTGGTTTCCTGTACATCCTCTATCACGTCTACGACGGTTATGACATGCCCCGCCTCTCT
AAGTGCTCCCTGGAGCCGTGCCCCAACACAGTGGACTGCTTCATCTCGCGGCCCACTGAG
AAGAAGATCTTCACCCTCTTCATGGTGATCTCTTCTGCCGTCTGCATCCTGATGTGCCTC
TGTGAGATGATCTACCTCATCTGCAAGCGCGTTCACAAACTCATTAAGCGAAGGAACGAG
GTGGAGAGAAGGTTGTTCGCCGAGAGTCATGAGATGGCCCCTCTGGCAGCACCAAGGTCC
GAGCTGAGGTCCAAATCATCGATCAGGGTGGATCCAACCGCCTCTGTCCAGGACCTCACC
GAAGAGAAGCGGCCACCTGAGAAACAAAAGATCGCAGCATAG

>Fr-gjb4like-XM_003962551
ATGAACTGGGCATTCCTCCAGGGCCTCCTCAGCGGGGTGAACAAGTACTCCACCGCCTTT
GGCCGAGTGTGGCTCTCCATTGTCTTCCTCTTCAGGGTCATGGTGTTCGTGGTGGCGGCT
GAGAAGGTGTGGGGCGACGAGCAGAAAGACTTCAAATGCAACACGGCTCAGCCCGGCTGC
CACAACGTCTGCTACGACCACTTCTTCCCCGTTTCCCACGTCCGGCTGTGGGCGCTGCAG
CTCATCTTCGTCACCTGCCCGTCTCTCCTGGTGGTGATGCACGTCGCCTACAGGGAGGAC
AGGGAGCGGAAAAACAGGCTTAAATATGGCGACGACTGCCGCCGTCTCTACCAGAACACC
GGGAAGAAGCGCGGAGGCCTGTGGTGGACCTACGTCCTCACGTTGGTCTTCAAAATCGGC
GTCGACGCCACCTTTGTCTACCTTCTCTACCACATCTACGAGGGTTACGACTTCCCCTCG
CTCATCAAGTGCCAGCAGAAGCCCTGCCCGAACACGGTGGACTGCTTCATCGCGCGGCCC
ACCGAGAAGCGGATCTTCACCATCTTCATGGTGGTCACCAGCCTGGTCTGCATCTTCCTC
TCCATCATCGAAATCCTCTACTTGGTGGGCAAACGCTGCCGTGAGTGTTTGACGGCCGGT
CACCACACTCACCACCCCATGACCAACAACATCTCGAGCGGAAGCCACCTGATGGAGTCC
AGCACTCTAAAGAGGGTTCCAAAGTGCACCCCCGAAACGCCGGCACCTTCGTACAGCTCT
GCCATATCCTGA

>Fr-gjb4like-XM_003969116
ATGAACTGGGCCTTCCTCGAGGGCCTCCTCAGCGGGGTGAACAAGTACTCCACAGCGTTC
GGCCGTATCTGGCTCGCCATCGTTTTCATCTTCAGGCTCCTGGTCTTCCTGGTGGCCTGT
GAGAAGGTCTGGGGCGACGAGCAGAAGGACTTTGACTGCAACACCCTGCAGCCCGGGTGT
CACAACGTCTGTTACGACTACTACTTCCCCGTCTCTTACACCCGACTCTGGTCCCTGCAG
CTGATCTTCGTCACCTGCCCGTCCCTTCTGGTCACGCTTCACGTGTCCTACAGGAAGGAT
CGTGAACGTAAACATCGGCTGAAGCACGGAGAAAACAGCCCCCCTCTGTATGACAACACA
GGGAAGAAGCGAGGAGGTCTTTGGTGGACCTACTTCTTCAGCCTGCTGTTTAAGATAACG
GTGGACGTGGTGTTTACTCTCCTGTTGTTCTACATCTACGAGGCCACCTTCTTCCCACCG
CTGGTGAAATGCGAGGAAGACCCGTGTCCCAACGTGGTGGACTGCTACATTGCCAGGCCG
ACGGAGAAGAAAATATTCACCATCTTCATGGTGGTCACCAGCTTCGTGTGCATCTGCCTC
ACGGTTTGCGAGGTTTTCTACCTGTGCGGGAAGAGGATCTGGGAGTGCAGCAGGGGCGGG
TGCCACCCTGACAGAGAGGACTCCTTCCTGGTGAGGGTTCCTCTGGACGCGAGGAACGCT
GTGAACAAAGGCTCGGTGGCGGCTGAGGCTGCGGCGCTCGACAGAGACGGAGAAGCCTTC
AGTCCCGCCCCAGCGTACGCCATCGCCGTCTCGTCAAGTCTGATGTGTGACGATTGA

>Fr-gjb6like-XM_011606139
ATGTCTTGGACCACTCTGTACGCTCAGCTGGCTGGAGTAAACCGTCACTCCACCAGCTTG
GGTAAAGTCTGGCTCTCTGTGCTCTTTATTTTTCGAGTTATGGTTTTGGTCGTGGCGGCC
GAGAGTGTTTGGGGAGATGAACAGTCTGACTTCACCTGTAACACCCTACAGCCTGGTTGT

GAGAATGTCTGCTATGATCAGTTCTTCCCCGTCTCCCACATCCGGCTCTGGTGTCTTCAG
CTTGTCTTTGTCTCCACTCCAGCTCTCCTGGTTGCGATGTACGTGGCCTACCGGAACCAC
GGCGACAAGAAAAAGCTCCTCCAGGTGTTCACTTTCAACAAAGGTCAGGAGGAAGAGTTG
GAGAGCCTCAGGAACAGGAGACTGCCTATATCTGGTGCCCTCTGGTGGACATACGCCTTC
AGCCTTCTGTTCAGGCTCTTGTTTGAAGGAGGATTCATGTACGCCTTGTATGTGATTTAT
GATGGCTTCCGGATGCCGCGCCTGGTGCAGTGCGACCAGTGGCCATGCCCAAACCTAGTG
GACTGTTTCATCTCACGGCCAACAGAGAAAACAGTCTTCACCATTTTCATGGCCACCTCA
TCCTCCATCTGCATGCTCCTTAACATGGCAGAGCTTGCATATCTTGTTGCCAAGGGAGTC
ACGAGGTAG

>Fr-gjb7-25-XM_003977315
ATGAACTGGGGCTTTCTGGAGAACATCCTCAGCGGAGTGAACAAATACTCCACGGTGATC
GGGCGCATCTGGCTCTCCGTCGTCTTCCTCTTCAGAATCCTGGTGTATGTCGCAGCAGCC
GAGCAAGTGTGGAAGGACGAGATGAAGGAGTTTGTGTGCAACACCCGTCAGCCTGGCTGC
GAGACTGCCTGCTTCAACCACTTCTTCCCCATCTCGCAGGTGCGCCTCTGGGCCATGCAG
CTCATCCTGGTATCAACCCCATCCCTGCTGGTGGCCCTGCATGTGGCCTACAGGGAGCAC
CGTGAGGCCAAGCACAAGAAACAACTGTACAAGGACAAAGCAACCATTGACGGAGGATTG
TTTTTAACCTACATCGCCAGTCTGGTTTTTAAGACTGCTTTTGAGGTCGGCTCCCTGCTC
ATCTTCTACTTTGTTTACAACGGTTTCGAGCTCCCTGTGTTGCTCCGCTGCAACCAGAGT
CCCTGTCCAAACACAGTGGACTGTTTCATTGGCAAAGCCACCGAAAAGAAGATTTTCCTC
TACATCATGGCCTGCACTTCTGTACTTTGCATCTTTCTCAATTCGGTGGAGCTCCTCTAC
ATTATATGGAAACAATTAGTCAAATGCGTCATCCGGCATCACGTTCCTGTGGAGAAGAGA
CCGTCCTCTCGCTACCACTCACAAGGGTCCAACATCAACAGATATGTCTCTGTTGAGCCT
GGTGTCATTAACGAAGGTGGCCCCATAAAGGCTAAAGCTGAAAACTTCCCAGTTCATTCT
GGGACTCATTAA

>Fr-gjc1-45-XM_003964814 Modified according to prediction in Ensembl (which has omitted other parts of the sequence).
In July 2019 this sequence was made obsolete, and replaced by XM_029836267 (and other transcription variants). There is only one nucleotide difference between our modification of XM_003964814 and the new XM_029836267, marked in purple. As we have used the accession number XM_003964814 in all analyses (which were done before July 2019), for the purpose of this manuscript we keep the obsolete accession number.
ATGAGCTGGAGCTTCCTCACGCGGCTGCTGGAGGAGATCCACAACCACTCCACCTTCGTG
GGGAAGCTGTGGCTCACCGTGCTCATCGTCTTCCGCATCGTTCTCACTGCCGTTGGGGGA
GAGTCCATCTACTACGATGAGCAGAGCAAGTTCGTGTGCAACTCGGGACAGCCGGGCTGC
GAGAACGTTTGCTACGACGCCTTTGCCCCTCTGTCTCACGTCCGCTTCTGGGTATTCCAG
ATTATCCTGGTGGCGATGCCCTCTCTCATGTACATGGGCTACGCCATCAACAAGATCGCT
AGATTAGATGAAGCCAAAGGAGGTGGAACCTCCACTGCTGTTAGAACGGGAGGGGGGGGC
TACACGCACAGGAAGCCCAGGAAAATCTGCTTTGGAGCGCGGCAGCACCGGGGTATCGAG
GAGACCGAGGAGGACCAGGAGGACGATCCCATGATCTACGAGGTACCGGAGATCGAGCCC
CCCAAGAGGCCGAGGGATCCGCTGCAGCCCGCTCCGAGACCCAAAGTCCGGCACGATGGA
CGCAAGCGCATCAGAGACGAGGGGCTGATGCGGGTTTACGTTCTGCAGCTGGTGACCCGT
ACGGTGCTGGAAGCCTGCTTCCTCGCCGGCCAGTATTTACTGTACGGGTTCCGTGTGATG
CCCGTGTTCGTGTGCTCGGGGAAACCGTGCCCCCACAACGTTGACTGCTTCGTCTCACGA
CCCACAGAGAAGACCATCTTCCTGCGCATCATGTACGGGGTCACAGTCCTTTGCCTCATT
CTCAACATTTGGGAGATGCTTCATTTAGGGATCGGCTCCATATACGACATCCTCCGCCGG
CGGCGCAGCCCACCCCAGGATGATGAGTACCAGCTGGGCTTGTTGGGTACCAGTGGAGCT
GTAGAGGGGCCCGTAGGGGGTACAGCCCCTGAGGCGGGCTCTGAAGGAGGGGTCGGCGGT
GACGGGGCTGCCGATTATGTCGGCTACCCTTTCTCGTGGAACACGCCGTCGGCTCCGCCT
GGCTACAACATTGTGGTAAAGCCCGAGCAGATGCCCTACACAGACCTCAGCAACACCAAG
ATGGCGTGCAAGCAAAACCGGGCAAACATTGCCCAAGAAGAGCAACAGCAGTTTGGTAGT
AACGAAGACAACTTCCCCACCGGAGGAGAAGCCCGCGTGGCTTTGAACAAAGACATGATC
CAGCAGGCTCACGAGCAGCTGGAGGCGGCCATCCAGGCCTACAGCCAGCAGCACCAGGCT
GAGGTGCAGCTCGGGGAGAACCAGGACGACAAACCCCAGAGTAACATCATTCAGGCTCAA
CCGCAGCTGCAGCCTCAGCCCCATAAGGAGCGCAAACACAGATTCAAGCACGGCAAAGGA
GGCAGCAGTGCAGGAGGCAGCAGCAGCAACAGCAGCAGCAGCAAATCGGGAGAGGGGAAG
CCCTCCGTGTGGATTTAA

>Fr-gjc1like-XM_003961198
ATGAGTTGGAGTTTCCTGACACGCCTGTTGGAAGAAATTCACAACCATTCTACGTTTGTG
GGCAAGATATGGCTGACTGTCCTTATTGTCTTCCGCATCGTGTTGACGGCTGTTGGCGGG
GAGTCCATCTACTACGATGAGCAGAGCAAGTTTGTGTGCAACTCGGGCCAGCCGGGCTGC
GAGAATGTCTGCTACGATGCCTTCGCTCCACTGTCACACGTCCGCTTTTGGGTGTTCCAA
ATCATTCTGGTGGCCACCCCATCGCTCATGTATCTGGGATACGCTGTCAACAAAATTGCT
CGTGCCGAAGAGCGGGCAGGTGGGAAGGGGGCGCGAGGCTATTCGCAGAGGAAACTCAAG
AGGAAGCTGTATCTGGCAGACAGGAGGCAGCACAGAGGCATCGAAGAAGCTGAGGATGAC
CAAGAGGAAGACCCTATGATCTACGAAACAGCAGACATTGGCAGTGAAGACGCAAAAGGG

AGCGTCACTAAGGGAAAAGATAAGGTCAAGGTGCGCCACGACGGACGCCAGCGTATTAAA
GAGGATGGCTTGATGCGGATTTATGTCCTTCAGCTCTTGGCCCGCTCCCTGCTGGAGGTG
GCTTTCCTGTGTGGGCAGTACACCCTGTATGGATTCGCTGTTCCCCCCACCTATGTCTGC
TCTCAGCTGCCATGCCCCCACAGCGTGGACTGCTTTGTGTCTCGGCCCACTGAAAAAACC
ATCTTCCTCCTCATTATGTACACAGTCTCCCTGCTCTGTCTGATGCTGAATATCTGGGAG
ATGCTTCACCTAGGCATCGGCACCATCTGCGAGATCATCCGTTCCCACCAACTCCCTGAT
GAGGAGCTGTACGGACTGACACAATCAAAAGGAGCCCACGCTGACGCTGGATTGAGCCGA
GAGGAGTACAGCAGCTACGCTTTCTCTTGGAATGCCCCATCAGCTCCGCCTGGGTACAAC
ATCGCAATCAAACCCCCTCTGGTAACAGCAGGACACCGTGATCAACCCCTGCCTGTCACT
GATCTCACCAACGCGAAGATGGCGTGCCGGCAAAATCACGCAAACATCGCTCACGAGGAG
CAGCAACAGTACAGCAATAACGACGAAAACCTATGCAAAGCCGGGATGGGCGATGACCAC
ATGCGTGCCAATCACTCTCAGAACAGACTGGAGGCGGACAGCGCAGCTCACAGCCAGCTG
GAGGGTCAAAGCAACAAGGCTCATCGTGACCGCAAACAGCGACAGGCCTCCAAACACACG
TCCAGCAAGGATGACACCGACCGGGGCAGCACCAGCACCAGCAATACCAGCAAATATGGC
GTCATCAAAGGTTCAGAGTGGATATGA

>Fr-gjc1like-XM_003978839 Modified according to ENSTRUT00000007687 (underlined sequence added). Splice sites.
ATGAGCTGGAGTTTCCTGACGCGTTTGCTGGACGAGATCTCCAACCACTCCACCTTCGTG
GGGAAGATCTGGCTGACCGTCCTGATCATCTTCCGCATCGTGCTGACCGCCGTTGGCGGC
GAAACCATCTACTACGATGAGCAAAGTAAATTTGTTTGCAACACGCAGCAGCCTGGATGT
GAGAACGTCTGCTACGATGCCTTCGCCCCGCTCTCACACGTACGATTCTGGATCTTTCAG
GTGATTTTGATAACCACCCCCACCATAATGTACCTGGGCTTCGCCATGCACAAGATCGCA
CGCATGGATGACAGCGAGTACCGCATCGTCCGGAAACCCAAAAAGAGGATGCCCATCGTT
AGCCGCGGAGCTGTTAGGGACTATGAGGAGGCAGAGGACAATGGAGAGGAAGACCCCATG
ATTGCCGAGGAGATTGAACAAGAAAAGCCTGACAAAACGGAGAAGGATCCTAACTGGTAT
CTGTGTCATCCAGGCACAGAGAAGAAGCACGACGGCCGGCGGCGAATCCAGCGCGACGGC
CTAATGAAGGTCTACGTGTGCCAGCTGCTGTGGCGCTCATCCTTTGAGGTTGCGTTCCTT
TTCGGCCAGTACATCCTCTACGGTTTTGAGGTTTTTCCGTCCTTTGTGTGCACCCGCTCA
CCATGCCCCCACACCGTGGACTGCTTCGTGTCGCGCCCCACAGAGAAGACCATCTTCCTG
CTGGTCATGTACGTCGTGTCTTTCCTCTGCCTGCTCCTCACCGTTTTTGAAATGATCCAT
TTGGGGATAGGAGGTGTCCACGACACCTTTCGGAGACGGGCCACTCTCAACCCACGCGCC
CCTCGTCCGTCCACCACACGCAGCATACCCACAGCCCCGCCAGGATACCACGCCACTATG
AAGAAGGAGAAACTGAAAGGACAGCTGAGGGACTCGCCGATAGGGGACTCTGGGCGGGAG
AGCTTCGGTGATGAGGGTCCGTCATCCAGGGAACTGGAGCGCTTGAGGAGGCACCTGAAG
CTGGCCCAGCAACACCTGGATTTGGCCTACCAGGTCGAGGAAGGAAACCCCTCACGGAGC
AGCAGCCCCGAGGTGAACACGGCTGCACAGACGGCTGCCGAGCAGAACCGACTCAACTTT
GCCCAGGAGAAGCAGGGAGAAACAACCGAAAAAGGTAAACCGGCGAGAAACTGGCTGAAA
TGTGCTATAATGTTATCCTGA

>Fr-gjc1like-XM_003962095
ATGAGCTGGAGTTTCCTCACGCGCCTGTTGGACGAGATCTCCAACCACTCGACCTTCGTG
GGCAAAATCTGGCTCACCCTCCTCATCGTCTTCCGCATCGTGCTGACGGCCGTCGGGGGC
GAGTCTATATACTATGATGAACAGAGTAAGTTTGTGTGCAACACAAACCAGCCTGGTTGC
GAGAACGTGTGCTACGACGCCTTCGCGCCGCTGTCGCACATTCGCTTCTGGGTTTTCCAG
GTGATTATGATCACCGCCCCCACCATCATGTACCTCGGCTTCGCCATGCACAAAATCGCC
CGGATGAACGACGACGACTATCGACCCCGCAGCAGGAAGAAGATGCCCATCGTCAGCCGG
GGAGCCAACCGGGACTACGAGGAAGCGGAGGACAACGGCGAGGAGGACCCGATGATCCTG
GAAGAGATCGAACCGGAAAAGGAGAAGGAGAAGGAGACCACGGAGAAGCCGTGCAAGAAA
CACGACGGGCGGCGTCGGATCAAGCGCGATGGCCTGATGAAGGTCTACGTGTTCCAGCTG
CTATCGCGGGCCATCTTCGAAGTCTCCTTCCTGTTTGGACAGTACATCCTCTACGGGCTG
GAAGTCGCACCCTCGTACGTTTGCACGCGCTCCCCTTGCCCGCACACGGTGGACTGTTTT
GTTTCCCGTCCCACGGAGAAAACCATCTTCCTGCTCATCATGTATGCGGTCAGCGGTCTT
TGCTTGCTCTTCACCCTGCTGGAGATCATCCACCTCGGCATCAGCGGTCTTCGGGACTGC
TTCTGCGCCCCCCGGCCTCGCCCTCCCACCCCGCGCCACTCGGCTCTCGCCAGCCAGAGG
TCCTCCATCTCCCGCCAGCCGTCCGCTCCGCCGGGCTACCACACGGCTCTGAAAAAGGAC
CCTTCGGGAAAGATGGGCTTTAGGGACAACCTGGGAGACTCCGGCCGGGAGTCTTTTGGT
GACGAGACTTCATCGCGGGAACTGGAGAGGCTGCGTAAACACCTGAAACTGGCGCAGCAG
CACCTGGACATGGCCTACCAGAACGGGGAAAGCAGCCCGTCGCGCAGCAGCAGCCCCGAG
TCCAACGGCACGGCGGTGGAGCAGAACCGGCTGAACTTTGCTCAGGAGAAGCAGAGTGAC
AAAGGTCAGACCCTAATTTTATTGTTGGCGCATCTTTGCCAGGGTTTTGATGGAATTCTA
GATCTGTAA

>Fr-gjc2-47-XM_003975332
ATGAGCTGGAGCTTCCTCACACGTCTGCTGGAAGAGATCCACAATCATTCCACATTTGTG
GGGAAAGTGTGGCTCACTGTGCTCATTATCTTCCGCATTGTGCTCACGGCAGTTGGAGGC
GAATCCATCTACTCGGATGAGCAGACAAAGTTCACCTGCAACACAAAGCAGCCGGGTTGT
GACAACGTATGCTACGATGCCTTTGCCCCTCTCTCGCATGTCCGTTTCTGGGTCTTCCAG

ATCATCATGATCTCCACTCCTTCCATCATGTACATGGGCTATGCCATTCACAAGATTGCT
CGGAGTACAGATGAGGAGCGCAGGAAACTCCACAGGCTTCGCAAAAAGCCTCCCCCGCAT
TCCAGATGGAGAGAGAGCCATCACCTGCAGGGCGTCTTAGAGGAGGACGAAGATGACGAC
GCTGAGCCCATGATCTATGAGGATACACTGGAGGTGCAAGATGCCAAACCAGAACCGGGG
AACAGCACGGGCAAAGACCCACCAAAATACGACGGCCGTCGAAAAATCATGCAGGAAGGC
CTGATGAGGATCTACGTCCTTCAGCTGATGTCAAGAGCTGTTTTTGAAATTGCCTTCCTT
GCTGGACAGTACCTTCTCTATGGTTTTCGTGTTAGTCCATCCTATGTATGCAACAGGATC
CCCTGCCCACACAGAGTGGACTGTTTCATCTCGAGACCCACAGAAAAAACAATCTTCCTC
CTTATCATGTACGTGGTGAGCTGTCTTTGTCTTGTGCTAAACATCTGCGAGATGCTTCAC
CTGGGAATGGGAACATTTCGGGACACCCTTCGCATGAAGAGGAGCAGGGGCAGACAGTCA
TCCTACGGCTACCCTTTTTCTCGCAATATTACAGCTTCCCCTCCAGGGTACAACCTCGTA
ATGAAGACAGACAAACCCAGCAGGATTCCCAACAGCCTCATTGCCCATGGGCAGAACGTA
GCCAACGTGGCTCAGGAGCATCAGTGCATCAGCCCGGACGAGAACATCCCCTCTGATCTC
GCAAGCCTACACCGGCACCTAAGAGTTGCTCAAGAGCAACTTGATATGGCATTTCAGACC
TACCAGACCAAACAAAACCAGCAAACCTCCAGAACCAGCAGTCCAGTGTCTGGAGGCACC
ATCGCAGAGCAGAACAGAGTCAACGCAGTTCAAGAGAAGCAGGGCGCAAGGCCGAAATCA
GCCACAGAGAAGGCTGCAACTATTGTAAAAAATGGAAAGAGCTCCGTTTGGATCTAG

>Fr-gjd2-36-XM_003962518 Splice site.
ATGGGGGAATGGACTATACTAGAGAGGCTCCTGGAGGCTGCTGTCCAGCAGCACTCTACT
ATGATAGGAAGGATCCTACTAACAGTGGTGGTCATCTTCCGGATTCTAATCGTGGCGATA
GTTGGAGAGACTGTCTATGATGATGAGCAGACCATGTTTGTTTGTAACACCTTACAGCCG
GGCTGCAACCAAGCGTGCTACGACAAAGCATTCCCCATTTCACACATTAGATATTGGGTT
TTTCAGATTATCATGGTGTGCACGCCGAGCCTTTGTTTCATCACGTACTCGGTGCACCAG
TCGGCCAAGCAGAAGGAGCGGCGCTACTCCACAGTCTATCTGACACTAGATAAGGATCAA
GATTCACTCAAACGAGATGAGAGCAAAAAGATAAAGAACACCATCGTCAACGGAGTCCTT
CAGAACACGGAGAACTCCACCAAAGAAGCCGAACCGGACTGTTTAGAAGTGAAAGAGATC
CCCAATTCGGCCATGAGAACTGCAAAGTCCAAAATGAGGCGCCAGGAAGGCATCTCCAGG
TTTTACATCATCCAGGTGGTTTTCAGAAACGCGTTGGAAATCGGCTTTTTGGTGGGCCAG
TACTTTCTGTACGGATTCAACGTCCCGTCGGTGTACGAGTGCGACCGCTACCCCTGCATA
AAAGACGTCGAGTGCTACGTCTCAAGACCCACGGAGAAGACAGTGTTCCTGGTGTTCATG
TTCGCCGTCAGCGGCTTTTGCGTGGTGCTGAACCTGGCGGAGCTCAATCACCTGGGCTGG
AGGAAAATCAAGACGGCCGTGCGGGGCGTGCAGGCTCGGCGGAAGTCCATCTATGAGATC
AGAAACAAGGACTTGCCGAGGATGAGCGTGCCCAATTTCGGGCGCACTCAGTCCAGTGAC
TCTGCGTACGTGTAG

>Fr-gjd2like-XM_003971111 Splice site.
ATGGGGGAATGGACCATCTTGGAGCGTCTGCTGGAGGCGGCTGTCCAGCAGCACTCCACT
ATGATTGGAAGGATCCTGCTGACAGTGGTGGTGATCTTCCGCATCCTAATAGTCGGCATA
GTGGGTGAGAAGGTGTACGAGGACGAGCAGATCATGTTCATCTGCAATACCATGCAGCCC
GGCTGCAACCAGGCCTGCTACGACAAGGCCTTCCCCATCTCACACATCCGCTACTGGGTC
TTTCAGATCATCTTGGTGTGCACGCCGAGCCTGTGCTTCATCACGTATTCCGTGCACCAG
TCTGCCAAAGCACGCGACCGAAGCTACTCCCTCCTGCATCCGTACATGGATCACCATGGC
CATGGTCACCACGGTCGCCATCACGACCATCACGCTCGCAAAATCCACTCGCGTTACATC
AACGGTATCCTGGTGCATCCTGAGGGCAGTAAAGAAGACCACGACTGCCTGGAGGTCAAG
GAAATCCCCAATGGACCCCGGGGACTGCCTCCAACACACAAGAGCGCCAAGGTTCGGCGG
CAGGAAGGTATTTCCCGTTTCTACGTCATCCAGGTGGTGTTCCGTAATGCGCTGGAGATA
GGCTTCTTGGCAGGCCAGTACTTCCTGTATGGCTTCAACGTTCCAGGGATGTTTGAGTGC
GATCGCTACCCCTGCGTGAAGGAAGTCGAGTGTTACGTATCTCGTCCCACAGAGAAGACT
GTGTTTTTGGTCTTTATGTTCGCGGTCAGCGGCATATGTGTGCTGCTCAACCTGGCTGAG
CTCAACCACATCGGCTGGAGGAAGATAAAGACGGCCATCCGAGGGGTGCAGGCTCGGAGG
AAGTCCATCTGCGAACTGCGCAAGAAGGACGTGTCTCACCTGTCTCAGGCCCCAAACCTG
GGCAGGACCCAGTCCAGCGAGTCCGCCTACGTCTGA

>Fr-gjd2like-XM_003968741 Splice site.
ATGGGAGAATGGACCATCCTAGAGCGCCTCCTGGAGGCTGCAGTGCAGCAGCATTCCACT
ATGATTGGGAGGATCCTGCTGACAGTGGTGGTGATCTTCCGGATCCTGATCGTGGCCATC
GTCGGGGAAACGGTGTACGAGGACGAGCAGACCATGTTCATCTGTAACACCCTGCAGCCG
GGCTGCAACCAGGCCTGCTACGACAAAGCGTTCCCCATCTCCCACATCCGCTACTGGGTC
TTCCAGATCATCCTGGTGTGCACCCCCAGTCTCTGCTTCATCACTTACTCAGTCCACCAG
TCAGCCAAGCAGAAGGACCGTCGGTACTCCTTCCTCTATCCCATCATGGAGAGGGACTAC
GGGGGGAGGGACGGCACGCGGAAGCTCCGCAACATCAATGGGATTCTGGTTCAACACGGC
GGCGATGGTGGAGGAGGGAAGGAAGAACCAGACTGTCTGGAGGTGAAGGAGATCCCCAAC
GCCCCGCGGGGCCTCACTCATGGCAAGAGCTCCAAGGTCCGGCGCCAAGAAGGGATCTCC
CGCTTCTACGTCATTCAAGTGGTTTTCCGGAACGCTCTGGAGATCGGATTCCTGGCCGGC
CAATACTTCCTCTACGGCTTCAGCGTGCCTGGGATTTTCGAATGTGACCGCTACCCGTGT
CTGAAGGAGGTGGAGTGCTACGTGTCCCGGCCCACCGAGAAAACCGTGTTCCTGGTGTTC
ATGTTCGCGGTGAGCGGCATCTGCGTGGTGCTCAACCTGGCGGAGCTCAACCATCTGGGG

TGGCGCAAGATAAAGGCCGCCATCAGGGGGGTCCAGGCCCGCAGGAAGTCCATCTGCGAA
ATCCGGAAGAAGGACATGGCTCATCTCTCCCAGCCCCCCAACCTGGGACGCACGCAGTCT
AGTGAGTCGGCCTACGTGTGA

>Fr-gjd2like-XM_011617194
ATGACTGAATGGACGCTGCTCAAACGCCTCCTGGACGCCGTCCACCAGCACTCCACCATG
ATTGGCCGTCTGTGGCTGACCGTTATGGTCATCTTCAGGCTGCTGGTTGTCGCCGTGGCG
ACCGAGGACGTGTACGCCGACGAGCAGGAGATGTTTGTGTGCAACACCTTGCAGCCGGGA
TGCTCCACCGTCTGCTACGACGCCTTCGCTCCCATCTCGCAGCCACGCTTCTGGGTGTTC
CACATCATCAGCGTCTCCACGCCATCGCTCTGCTTCATCGTCTACACGTGGCACAACCTG
TCCAAGTCCCCCCACGCCGCTCTGGGGCGGCGCCGCGGCGTTAAGGAGGGAGGGCAGGTG
GGCGGCGGACAGGAGGGGGGTCCTCCCAGCTGCAGCTCGGACAGCTGCTCCGTCCTCTCC
CATCAGCACCTGGGCCACAGCCTGGCGGACATCTTAGAGGGCGGAGGCCTGGTGACCTCC
CGTCACGTCCCGGCAGGAAGTTCTGAGGGCCTGGCGGTCTCTGGAGGAGTCCTGTCCAAA
TGTTACATCTTTCACGTGTGTTTACGAGCCGCTCTGGAGGTGGGCTTTGTCCTGGCCCAG
TGGAAGCTGTTCGGTTTGCAGGTTCCGGTCCTCTTCGTGTGCAGCTCCTCGCCCTGCAAC
CAGCCCGTGGACTGCTACGTCTCCAGGCCCACAGAGAAGACCATATTCCTGATTTTCATG
TTTTGTGTTGGTCTTTTCTGCATCTTCCTCAATCTGCTGGAACTCAATCACCTGGGCTGG
AAGAAGATCCGGCAGGCGGTGCAGCTGAAGGAGGAGCCGTCCTGGCCAGGCTGCACAGGT
GGACGACAGGGCTATGAAGCCCTTCCTCCAGTCAGTCCTTCACCCAAGTCCTCCTTAGGC
CTGAAGGACATCAGCTCCACCCCTCTGCCCACCCTGGATGTGGTGATGGCTCACCGCCCC
GAGTGGAGCTGCGTGCTGAACTGTGGCAAGAAGAGAGAGTTCCAGAAGGTCCAGGAGATC
CGCTTAGAGGTGTCCAAGAGAGCCGACCCCCACAGAGGACAGAGGCCACCTGTGAAGAAC
GCGGAGACCAGAGGCTCCAAGCAGAGCTGCACCGAAGTCTGGATTTAA

>Fr-gjd2like-XM_003971197
ATGGGAGACTGGTCCATTCTTGGCCGCTTCTTAACAGAAGTTCAGAATCATTCCACAGTC
ATTGGCAAGATATGGCTGACAATGCTGCTCATCTTCCGCATCTTGCTGGTGGCGTTGGTG
GGCGACGCGGTGTACAGTGACGAGCAGTCTAAGTTTACCTGCAACACCCTACAGCCTGGA
TGCAACAACGTCTGCTACGACACCTTTGCTCCTGTGTCACACTTGCGCTTCTGGGTCTTT
CAGATTGTCCTCGTCTCCACACCTTCGATTTTCTACATCGTCTACGTCTTGCAAAAGATC
ACCAAGAATGAAAAGTTAGAGGTGAAGAAGGTGGTCGTGGTACCCAGGTCTCCCACACCG
CTCAGAGGGGAGAAGGATCCGGGGGGAGATAAAGAGGCAATGCTGGAGGGGGGTAGTTAT
AACACCACCTATAACAACGAAGAGTGGAGCTCTCAGGAGGATGAGTGTGAGGAGAGGAGC
CAGCTGAACGAGGAAATGAAAGAGGTCGGAAAGGACCCGACCCAGCTCTCCAGTCAAGTG
TTGCTCATCTACATCATCCATGTTCTCCTGCGCTCCATCATGGAGCTCATCTTCCTGATC
GGACAATATTACCTCTTTGGATTTGAAGTGCCGCATCTTTTCCGCTGTGACACCTACCCG
TGTCCAAACCAAACCGACTGCTTTGTGTCCCGAGCCACAGAGAAGACCATCTTCCTGAAC
TTCATGTTCAGCGTCAGTCTGGGATGCTTCATCCTGAACATTGTGGAGCTGCATTATCTC
GGCTGGATTTATATCTTCAGGGTGTTGTTCTCTGCATGCTGCACGTGCTGCAAGTCAGAC
AGAGACCCCGTTCGGCAGGTGGAGTTGTATTCGGACAACAACCCGCTGCTGCTGGAGCTC
AAGCATTCACTGCGGGGCAGGGTCGTGCTGCAGGCCACCTCTGCTGTGTCACGGGACAAG
AGCAGCAGCGTCCCAAATCAAGCCCCGGCCATCTCTTTTGAAACAGACTCTACGCTGGAG
TGCACGTCAAAGAGGAACCTAGATGAGAAGGAACGCGCGAAAACAAGACTACATAAAATG
GGAAGAGGCAAAAAGTCATGGCTGTAA

>Fr-gjd3-31.9-XM_003961468
ATGGGGGAATGGGGCTTCCTCGGTGGACTCTTCGACAGCCTCCAGGCTCACTCGCCCATG
CTCGGCCGCTTCTGGCTCCTGCTCATGCTCATCTTTCGGATAGTGATCCTCGGAACTGTG
GCCAGCGACCTGTTTGAGGACGAACAGGAGGAATTTGCCTGCAACACCCTCCAGCCGGGC
TGCAAACAGGTGTGTTACGACATGGCCTTTCCCATCTCGCAGTACAGATTCTGGGTGTTT
CACATCGTCCTCATCGCCACGCCATCGCTGCTTTTTCTGGTTTACACCATGCATCACCAC
AATAAGAAGAACTCCAAATTCAATCAGAGGTACAATGAAGACATCCGTTTAAGGAGGCTT
TACATCGTCAACGTGGTGTTTCGCATCCTGGCAGAAGTTGGGTTTCTCGTGGGTCAGTGG
CTGCTCTATGGCTTCAAGGTGGAGGCCCAGTTCCCCTGCAGCCGCTTCCCCTGCCCCTAC
ACCGTGGACTGCTTCACCTCCCGCCCGGCGGAGAAAACCGTCTTCCTCTGCTTCTACTTT
GTCGTCGGGGCGATTGCAGCCCTTTTCAGCTGTGCTGAGCTCTTCCACAGCTCCATAAAG
TGGTTCTGCTGCAGCACGGAGGTAGGAAGGCAGAAACAGAACTTGCCCTGTATCAGCGAC
AACCTCTTCAACTTCAAGCAGGAAGAAGAGACAGCAAAGGAGAAGCGGCAGATGAAGAAT
CCGCACGCACCCAACAGCGTGAGGCAGAAGAGAGGATCAGCGAAGAGCATCTCTAGGAAG
AGCTCCAGTGGCGTCCACAGGCACATCGGTGGGAAAGTGGTGAGCACCAGGACATTCATG
GTGTGA

>Fr-gjd4-40.1-XM_003967849 Fr161792 Splice site.
ATGGAGGGATCAAATGCCTGTGAGGTCATCTTTATCTCTGTCAATCACAGCATCACACTG
ATGGGTAAAGTGTGGCTCATAGTGATGATCTTTCTCCGTGTCCTGACGCTCCTCTTTGCT
GGATACCCCCTCTACCAGGACGAGCAGGAGCGATTTGTGTGCAACACCATCCAGCCTGGG
TGTGCCAACGTCTGCTACGACTTGTACTCTCCGATTTCACTCTTCCGCTTCTGGCTGGTC

CAGCTCCTCACTTTGTGTCTTCCTTACATCATCTTTGTTGTCTACATCATCCACGAGGTC
TCAAATGACCTCTGTGTGCACCCGAACCCCCCGGGCCACGTCAAAACCTCACAACTTTTC
CAGATCCAACAAGACTCTTTCAGGAAGGCACCGGGCAGCAAGATGGCGACCGCAAGGAGA
TCGGATCGATGCTTCTCAGGAGCATATGTCCTCCACCTGATGTTCAGAACCTTGCTGGAA
GGAGGGTTTGGAGCAGCGCATTACTATCTCTTTGGTTTCTACATCCCCAGGAGGTTCCTG
TGCCAACATCCGCCATGCACCACACAGGTGGACTGCTACATTTCCAGACCCACTGAGAAG
ACTGTGATGCTCAACTTCATGCTTGGTATGGCCATCCTGTCTCTTTTTTTAAACGTGTTG
GATTTTATAAGCTCCATCAAACGCTCTGTGACCAAGAAGGGCAGAAAGAAGATGGCGGTC
GAGAAGAATTATGAAGAAGAGCAGTGCTCTAGTTCAACTGGTGTAGCCTTCAGATCAACA
GACCCAAACGCCCCGTTGACACAGGACCTGGACGTGGAGGTTCCTCAAGCAGGAAGTTTC
CGAAAAAGGCGCAACAGCAAGGGTTCTTGTGGAGGGCCAGATCCGTCCTCTCTCGACCGT
TCTTCATCTTTTCCACGTTCACTAGGACCTCAAGGGTGCAACACAAATGGGAACAATGGC
TACTCAGTTCCACAGGAAGATGTTCTGGAAAACAACGGCAGCGACGTGGCTCTTTGCCCT
CCAGACTCCATGGGGACGCCTAGATCTATTCGCGTTAGCAAACGAGGGCGATTAAAACCT
CCTCCTCCCCCTAGACGAGACCTTGGTTCCTGTCCAAGGGGCCCGGCGGGGTCCACCGGG
GATATATCAGCGATTTGTACCAAAAAGGTTGGCCAGTTCACAATGCTAGAGCAGCTACAG
ACCAATGATGATGGGCAAGACAAAAGGTCAGAGTGGGTCTGA

>Fr-gjd4like-XM_011616749
ATGGGAGCCAGCGACGTTCTCTTCATCACGCTCAGCCACAGCGTCTCCTTCCTGGGGAAG
GCCTGGTGGACCCTGATGCTGGCCCTCCGCCTGCTCCTGCTGCTGCTGGCCGGCTTCCCC
CTCTTCAGCGACGAGCAGGAGCGCTTCGTCTGCAACACCATCCAGCCGGGCTGCTCCAAC
GTCTGCTTCGACGCCTTCGCTCCCGTGTCCGTCTTCCGCCTCTGGCTCTTCCACCTCATC
CTTCTCGCCCTTCCCCACCTGCTCTTCGCCACCTACGTCGCGCACAAGGTCTTCGCGCAC
CCGGGTCCCGGAGGGTTCTACTGCGCCGGGAGCCGTGGAGGTTCCCCTGTCGGCCTGGAG
AACCGCGGCTCGTCCAGAGAACTGTCCCTGCTCAAGAGCCGGGTCCAGGAGCCCAGAGGA
CCACGCTTCTACTGCGCATACGTGCTGGTGGTGGTGCTCAGGATCCTTCTGGAGGCTGCT
TTTGGAGCGGGCCAGTTCTACCTCTTTGGTCTGTCCTTTCCAAAGAGCTTCCTGTGCTAC
GAGGCCCCCTGCACCTCCGGGGTGGAATGCTACGTCTCCAGGCGCACGGAGAAGTCTTTA
ATGATGAGCTTCATGTTGGGCGTCTCCTCGCTCTCTGTCCTGCTGAGCTTGGTTGATCTG
ATGAGCTCCATGAAGGCGCTGGTGAGGTGGAGGAGCAGGAGGGAGGTGCTCGCGGAGGAG
CTGATCAGAGGAGAACAAAGCAGCGTGTTGACGGCCACAACCATGGCTGAAGACGGAGAT
AAAAGCCCGCGATCAAAGAACCATCCTGACAGCAAAGATCCTCAGGTGGACACGCCTCCC
ACTCCCAGGAGCACCCCAGCACCGTCTCAGGATGCCCTCAACAGCCACCCCAGACCCCCG
CTGTCCCCTCGGCCTGACAGAGAGCCATCATCAAAGCTGAGGGCCCCGGCACCAGTGGGG
GGGGGGGACACGGGTCAGCACGGTCCAGCCAGGACAAACTCAGGCCAACAGTCCGACAGC
AGTGACTCTCAAGAACGGCGAGCCTGGGTGTGA

>Fr-gje1-XM_011611785 This prediction is probably erroneous with regard to the first exon. We have replaced this with the more likely first exon, which is separated from exon 2 by a approx 235 nt intron. Red font: Our suggested modification for exon 1. Splice site
ATGTCCTTAAACTACATCAAAAACTTCTATGAAGGATGCCTCAGGCCTCCTACTGTGATA
GGCCAGTTCCACACCTTGTTCTTCGGCTCAGTACGGATGTTCTTCCTGGGCGTTCTCGGC
TTTGCCGTCTACGGGAATGAGGCGCTGCACTTCAGCTGTGACCCTGACCGCCGAGAACTC
AACTTGTACTGCTACAACCAGTTCAGACCCATCACGCCTCAGGTGTTCTGGGCTCTACAG
TTAGTGACAGTATTGGTTCCTGGAGCTGTGTTTCACCTCTATGCAGCCTGTAAGAACATT
GACCAGGAGGAGATCCTGGAACGGCCCATCTACACCGTCTTCTACATAATTTCTGTTCTT
CTGCGTATCATTCTGGAAGTCATTGCCTTCTGGCTGCAGAGCCACCTTTTTGGCTTCCAG
GTCCACCCTCTGTACATGTGTGACGCGAGTGCTCTGGAAAAGACCTTTAATGTGACCAAG
TGCATGGTTCCTGAACACTTTGAGAAGACCATCTTCCTCAGTGCCATGTACACCTTCACT
GTCATCACCATACTTCTCTGTGTCGCTGAGATCTTTGAGATACTCTGTCGGCGGCTCGGT
TATCTCAACAACCAGTGA

Suppl. Fig. 7. Green spotted pufferfish (*Tetraodon nigroviridis*) connexins.

Green spotted pufferfish, *Tetraodon nigroviridis* (Tn)
Assembly: TETRAODON 8.0, March 2007.
Genebuild: May 2010.
Database version: 98.8

As far as possible, the names of the sequences are taken from the Ensembl predictions. Where there is a prediction (although we might have modified it) without a name, we include NN (no name) as a prescript, use the most common name of the ortholog sequence (usually from zebrafish), and end the name with an abbreviated Ensembl gene prediction number. Where there is no prediction in Ensembl and no predicted (or experimentally found) sequences in GenBank with a name, we include NP (not predicted) as a prescript, and use the most common name of the ortholog sequence (usually from zebrafish).
The Ensembl gene abbreviation is done as follows: ENSNIG00000015676 = G15676.

Yellow: Conserved domains as defined by Cruciani and Mikalsen (2007)
Green: Conserved cysteine codons (cysteine signature)
Grey: 15 nt added at the ends of the conserved domains
Turquoise: Splice site.
Other colors are explained where necessary.

```
>Tn-NP-gja1 This sequence is predicted by  Ensembl as an intron in the gene
ENSTNIG00000007253 enah. Note that this sequence was included in our previous
analyses (Cruciani and Mikalsen, 2007) as Tn13946001.
ATGGGTGACTGGAGTGCTCTGGGTCGTCTGCTGGACAAGGTGCAGGCCTACTCCACCGCT
GGAGGGAAGGTGTGGCTCTCTGTGCTCTTCATCTTCCGGATCCTGGTGCTGGGGACTGCG
GTGGAATCGGCGTGGGGGGACGAGCAGTCTGCCTTCAAGTGCAACACGCAGCAGCCGGGC
TGTGAGAACGTCTGCTACGACAAGTCCTTCCCCATCTCCCACGTGCGCTTCTGGGTGCTC
CAGATCATCTTCGTGTCCACACCGACCCTCCTGTACTTGGCTCATGTCTTCTACCTGAAC
AGGAAAGAACAGAAATTCAGCAAGATCGAGGAGGTGCTGAAGGCGGTCCAAAACGATGGA
GGCGACGTGGACGTCCCGCTGAAGAAAATTGAGATGAAGAAGCTGAAGTATGGCATTGAG
GAGCACGGGAAGGTGAAGATGAAGGGAGCCCTGCTGAGAACCTACATTGTCAGCATCTTC
TTCAAGTCGCTCTTTGAGGTGGGCTTCCTGGTGATCCAGTGGTACATGTACGGTTTCAGC
CTGTCCGCCGTCTACACCTGTGAGCGGTCCCCATGTCCACACCGGGTGGACTGTTTCCTG
TCCCGTCCCACCGAGAAGACAGTCTTCATCATTTTCATGCTGGTGGTGTCGCTGGTGTCC
CTGCTGCTCAACATCATTGAGCTCTTCTACGTGCTCTTCAAGAGGATCAAGGACCGGGTG
AAGGGCAAGCAGCAGCCGGCGCTCTACCCCAGCGCCGGCACCCTGAGCCCTGGGCCCAAG
GAGCTGTCCACCACCAAGTACGCCTACTACAACGGCTGCTCCTCACCCACCGCTCCGCTC
TCACCCATGTCCCCCCCGGGCTACAAGACGGCCACGGGGGAGCGGGGGACCGGCTCCTGC
CGGAACTACAACAAGCACGCCAGCGAGCAGAACTGGGCCAACTACTCCACCGAGCAGAAG
CGGCTGGGCCACACCGGCGCAGGAAGCACCATCTCCAACTCCCACGCCCAGGCCTTCGAC
TTCCCCGACGACACCCAGGAGCACAAGAAGATGTCCTCGCTGGCGGCCCACGAGCTGCAG
CCGCTGGCGCTGCTGGATGCTCGGCCCTGCAGCCGCGCCANCAGCAGGCTGAGCAGCCGC
GCCCGGCCTGACGACCTGGACGTCTGA

>Tn-gja3-G15676 Bold+italics: Two nucleotides removed to avoid spurious(?) stop
codon and keep reading frame. Underlined: Sequence not included in Ensembl
transcript prediction, but we consider it as a likely part of cds.
ATGGGCGACTGGATCTTTCTGGGGCGGCTGCTGGAGAACGCTCAGGAGCATTCCACCGTC
ATCGGCAAAGTCTGGCTGACCGTCCTCTTCATCTTCAGGATCCTGGTGCTGGGCGCGGCC
GCCGAAGAGGTGTGGGGCGACGAGCAGTCGGACTTCACCTGCAACACCCAGCAGCCCGGC
TGCGAGAACGTCTGCTACGACGAGGCCTTCCCCATCTCGCACATCCGCTTCTGGGTGCTG
CAGATCATCTTCGTGTCCACGCCCACGCTCATCTACCTGGGCCACGTGCTGCACATCGTC
CGCATGGAGGAGAAGCGCAAGGAGAAGGAGGAGGAGATGCGCAAAGCCAACCGCTTCCAG
GAGGAGAAGGAACTCCTTTACCGAAACGGGGGGGACGCAGGAGGCGGCGGCAGGAAGGAG
AAGCCGCCCATCAGGGACGAGCACGGCAAAATCCGCATCAGAGGCGCGCTGCTGCGGACC
TACGTCTTCAACATCATATTCAAAACCCTGTTTGAGGTGGGATTCATTCTGGGCCAGTAT
TTCCTGTACGGCTTCCAGCTGAGGCCCCTGTACAAGTGTGCGCGTTGGCCCTGCCCCAAC
ACGGTGGACTGCTTCATCTCCCGGCCCACCGAAAAGACCATTTTCATTCTCTTTATGCTT
GTGGTGGCTTGCGTGTCTCTTTGCTGAATTTGTTAGAGATCTATCACCTCGGGTGGAAG
```

AAGGTCAAACAGGGCGTGACCAACCAGTTTGTCCCCGACGGCGAGTCGCTGCGCCGGGTC
AACATCGCGGAGCCCGAGTGTTTGGCCCCGCCCCCCAGAACTGCCCCGTCCAGTTACCCC
CCCGACTACACGGACGTGACGGCAGGCAGCGGGGCCTTTCTGCAGCCCGTGGCGCCGCCG
GCCGTGCCTTCGGGCGCCGCGTTCAAGATGGACGACCTCCAGCGGAGCCAGCCTCCCCAC
CAGCCCCCCTCCTCCTCCTCCTCCTCTTCCTCCCCTCACTACTACATCAGCAACAACAAC
AACCACAGGTTGGCCGCGCAGCAGAACTGGGCCAACCTGGCCACCGAGCAGCAGACTCGG
GAGATGAAGGCCACCTCCCCCTCGCCCTCCTCCTCCAACACCTCCCATGATGAGCAGCAG
CAGCAGCCTGTTGATGCGGAGCTGCTCCCTCCCGCCACCAACACCAACACCATCGCCGCC
GCCGATGCTCCAGGGAGCAGCAGCCCGGGCTCCGCCTCCAACGCAGGCAGCTGGGGTGGA
GGAACCAACGAGCAGGAAGGAAGGCGCGTCTCCACCACCAGGGTGGAGATGCACGACCCT
CCGCCCGCCCCCGGCGTGGACCCTCGGCGACTCAGCCGAGCCAGTAAGAGCAGCAGCGTC
AGAGCGAGGCCGAGCGACCTGGCCGTCTGA

>Tn-GJA3-G10339 Underlined: Sequence not included in Ensembl transcript prediction (ENSTNIT00000002769), but we consider it as a likely part of cds. Splice sites.
ATGGGTGACTGGAGCTTTCTAGGGCGGCTGCTGGAGAATGCTCAAGAACACTCCACTGTG
ATTGGAAAGGTTTGGCTGACTGTCCTCTTTATCTTCCGCATCCTGGTGCTGGGCGCAGCC
GCTGAGGAGGTTTGGGGTGATGAGCAGTCCGATTTCACCTGTAACACGCAGCAGCCCGGT
TGCGAAAACGTCTGCTACGACGAGGCCTTCCCCATCTCCCACATTCGCTTCTGGGTGCTG
CAGATCATTTTTGTCTCCACGCCAACCCTCATCTACCTGGGCCACGTGCTGCACATTGTC
CGCATGGAGGAGAAGAGGAGAGAGAGGGAAGAGGAGCTCCGGAAGGCAGGGCGGCACCAG
GAGGACCACGATCCTCTTTTTCATAATGGAGTTAGCAACGGAGGAGGCAGAGGTGGCGGG
AAAAAAGAGAAGCCGCCTATTCGGGATGAACACGGGAAGATCCGTATCCGCGGGGCGTTA
CTGAGGACCTACATCTTCAACATCATCTTCAAGACTCTATTTGAGGTGGGCTTCATCCTG
GGGCAGTACTTCCTCTATGGCTTCCACCTGAGGCCGCTCTATAAATGTGGCCGCTGGCCC
TGCCCGAACACTGTGGACTGCTTCATCTCCAGGCCCACCGAAAAGACAATTTTTATCATC
TTCATGCTGGTGGTTGCGTGCATCTCCTTGGCCCTCAACCTGTTGGAAATCTACCACCTG
GGATGGAAGAAGGTCAAGCAGGGAGTCACCAATGAGTTTGTCCCCGACGGTGAGTTGCTG
TTGAGGAGTGCCAACAAGCACAGAGACGCGGAGAAGATCCGTGAGCAGGCTTCTCCATCG
GTGCTTGAATGTTTGTCGACTTACTCCAGCATGAATGTGGCAGGAAGCGGAGGGAATGAA
GGAAGATCCTACAGTCCGCCCGAGGCCTCTCTGGCTGTGATGTCATCACCTGCCAGTCTC
AAGATGGACGGCAGCGCGTTCCACCCGGACGACCTCTTGTTGGAGGCCCTGCCTGCTTCT
TTTTGCGGCAGTAGTGACAAAGTGAGCCACGGGCAGCTGACAGAAGTGGAGCAAAACTGG
AGCAACATGGCACTGGAGCTCCAGAATCTCAATGGGAAAAACTCCTCCTACCCTCCTCCC
CTTCCCTCCCCTCCCAACTCCTCCTCCTCCTCCTCCTCCTCCTCTTCTCTTCACGAGGAG
ACAAACCCTCCGCTTCCTCAAGGGGAGCAACACTCCATGTTCCCCACGCTGCCTCGTCAT
GATCCCCTCTACGCTCTCACTCCAAAGGAGACCATGGAGGAGCCCTCTACTGCCTCATGT
GACGTCCCACCCGACGATGTCACCGTGGTTACCAAGGCAGAGATGCACTGGCCTCCTGCT
TCTGCTGCCACAGACATCCGGAAGCCAAGTCGGGCGAGCAGGAGCAGCGTCAGAGCACGC
CCCGATGACCTGGCAGTGTAG

>Tn-NN-cx39.9-G08981 Underlined: Sequence not included in Ensembl transcript prediction, but we consider it as a likely part of cds.
ATGGGGGACTGGAACCTGCTGGGGAAACTTCTGGAAAAAGCCCAGGAGCACTCGACTGTG
GTGGGCAAAGTGTGGCTGACCGTCCTCTTCATTTTCCGCATCCTGATCCTGAGCGCCGCC
ACTGAGAAGGTGTGGGGCGACGAGCAGTCGGGCTTCACCTGTGACACCAAACAGCCTGGT
TGCGAGAACGTCTGCTATGACATCACATTCCCGATCTCCCACGTGCGTTTCTGGGTGCTG
CAGATCATCTTTGTGTCGACGCCGACGCTGATTTACCTGGGACACATTCTCCATCTGGTG
CGGATGGAGGAGAAGCAGAAGGAGAAGGAAAAGGAGCACGCAAGACTGTCAGCAAAGCAG
GGTCTGCTGGTCTCCAAGCACAAAAAGCCCCTGGTGAGGGACGAGAAGGGCAGAGTGCGC
CTGCAGGGGGAGCTATTGCGCACATACGTCTTTAACGTCGTCTTTAAAACGCTGTTTGAG
GTGGGCTTCATCGTGGCTCAGTATTTCCTTTATGGCTTTGAGCTGAAGCCGATGTATACA
TGCAACAGAGCCCCCTGCTCCAATGTGGTCAACTGCTATATTTCCCGGCCCACGGAGAAG
ACCATCTTCATCATCTTCATGCTGGGCGTGGCCAGTGTGTCTCTGCTCCTGAATCTCATT
GAGATCTATCACCTGGGCTTCACCAAGTGCCGCCAGGGTCTCACCTTCAGGAGGCGGGAC
CTTCCCTCCGAGGGGATTCTCAAGGACCCCAGCGTGGCCTCTGTGCCCTTTGCGCCCAGT
TACGATGAGTACTTCCACGGACACCACCCGGTGCAGCCGGCCTACCCGCCCGTGCCCGGC
TACAACCTCTCCCCGCTGTCTGACGGCACCGAGTCGTCTTTCCATCCTTACAACAGCAAG
GCAGCCTACAAGCAGAATAAGGACAACCTGCTGGTGGAAAGGAGCAGCAGCAAGCCGGAG
GAATGCGACCTGAAAGGAGAGAAGGATCCGGGTTCTGCCCCCGAGTCACCTACGCAGGTC
AGGTCTAGCCGCAGCGCCAAACACGGCAACAACAAGACTAGAATAGACGATCTGAAGATA
TGA

>Tn-NN-cx39.9-G11824
ATGGGGGACTGGAACTTGTTGGGGAAGCTGCTGGAGAGTGCCCAGGAACACTCCACCGTT
GTGGGCAAAGTCTGGCTGACAGTGCTGTTCATCTTCCGTATCCTGGTGCTGGGAACTGCC
GCTGAGAAGGTGTGGGGAGATGAGCAGTCCGGCTTTACGTGCGACACCAAGCAGCCCGGT
TGTCAGAACGTTTGCTACGACAAGACCTTTCCCATTTCCCACATCCGCTTCTGGGTGATG

CAGATCATTTTCGTCTCCACGCCCACCCTCATCTATTTGGGCCACATCCTTCATCTGGTT
CGCATGGAGGAAAAAGAGAAACAGAAAGAGAAGGAGCTGGCAGCCCAGAGTGAAAAACAG
CAGCAGTTGCTTGGCAACAAGCCGAAAAAAGCCCCAATTAAAGACAACCAGGGTCACGTG
CGTTTGCAAGGCGCCCTGCTGCGAACTTACGTCTTCAACATCATCTTCAAGACCCTGTTT
GAAGTGGCCTTTATTGTAGCTCAGTACTTCCTCTATGGTTTCGAGCTCAAGCCGATGTAC
ACCTGCGACCGCTGGCCTTGCCCCAACATGGTGAACTGCTACATCTCTCGACCCACTGAG
AAGACGGTCTTCATCCTCTTCATGCTGGCGGTGGCTTGCATCTCTCTGCTGCTCAACCTG
GTGGAAATGTACCATCTGGGATTCACCAAGTGCCACCAGGGCCTTCGGTACAGGCGATCA
AAGACCAGAAAACAGTCTCCCAAGGCCCTCCACGAGCCCGTCATGCCCTTTGTTCCCAGT
TACAACTACTACACCGGTCACCCTGCAGTGCCGGAGCCGTTTCCGACCGACTCCAAGTAC
AGCGTGACAGAGCCCGGCTCCGCTTACAGCCCCTACAGCAATAAGGTCGTCTACAAGCAG
AACAGGGACAACATGGCTGTGGAGAGGAAGGGAAAACCCGAGGACGAGGTCGTGATGGAG
AGGAAACCCACCTGCCCTGCCTTTGAGGGGTCTGCTGACAGTCAGCGCAGAAACAGTCAG
TCAAGCAAGCACAGCAAGAGCAGACTGGATGACCTAAAGATCTAA

>Tn-cx39.4-G09223 Underlined: Sequence not included in Ensembl transcript prediction, but we consider likely as a part of cds. Red font: One nucleotide is removed here relative to the genomic sequence. Ensembl predicts a 4 nt long intron here (cgagcgaAGCCaacttt), where upper case letter is the predicted Ensembl intron, and g is the nucleotide that we have removed.

ATGTCAAGAGGTGACTGGTCCTTCCTGGAGAACCTGCTGGAGGAGGGCCAGGAGTACTCG
ACAGGCATCGGCCGTGTCTGGCTCACCGTGCTCTTCCTCTTTCGCATGCTTGTGCTGGGA
GCATCTGCAGAGTCGGCCTGGGATGACGAGCAAGCCAACTTTGTCTGCAACACGAATCAG
CCTGGCTGCACCAGCGTGTGCTACGACAAAGCCTTCCCCATCTCCCACTTCCGCTACTTT
GTCCTCCAGGTCATATTTGTGTCCACGCCGACCATTTTCTACTTCGGATACGTCGCTTTG
AGAGTCAGAAGGATCAAAAAAGACGCAGAGGAAGGTTTTGATAGAGGAACTGTAAAAAAG
ACAAACAGTCACTCAGAGGAAGCGAGGAAAAGCGGGAGGGCTGAAGAGGAAGCTCCCGAG
GCACCTCGGCTGAAGGGCAGACTGCTGTGTGCATACGCCCTCAGCATCTTCTTAAAGGTC
CTCCTGGAGGTTGGCTTCATGTCAGGCCTGTATTTTCTCTACAATGGCTTCTACATCGCA
GCAAAGTTCGAGTGTCACAGGAACCCTTGTCCTCACACGGTGGACTGCTTCGTCTCACGG
CCCACGGAGAAGACCATCTTCGTGGTATACACTCAGGTCGTCTCCGGCGTCTCCCTGCTC
CTCAACCTGCTGGAGCTGCTCCACCTTCTCCAGCTTGCCATCACTCACCGGCTGGAGAAA
CATTACCACGGTCGTCATGGAGACTACCTCCCCCCCGCAGAGCAGGGGACTGCGGAAGCT
GCACGAATCCAAATGGAGGCCTCGCAGTCCGGTAAGACAGGGAGCGACACTCACCTTCCA
ACCCAGTGTGAGATGGAGGAGTCTGCCAATCCCTGCCAGAGTTTCGGAGAAGCAGGCATA
GAACCAGGCATGAACCGCTCATCTGGAGAGACTGGGAACAGCCTCCTCCCCAGTTATGTG
ACCTGCATCAAAGCCTCGAGGATGATGCATTCACCCAGAGCCCATCACAAAAAAACCACA
GTCCACACCTCCAAAAACACCAAGGCCGCTCAGAAGGGACATTCCAAACTCAGGCATTAC
GTCTGA

>Tn-GJA5-G02166 Underlined: Sequence not included in Ensembl transcript prediction, but we consider it as a likely part of cds.

ATGGGTGACTGGAGCTTCCTGGGGAACTTTCTAGAAGACGTCCAGGAACACTCCACCTCG
GTCGGGAAGGTCTGGCTCACCGTCCTCTTCATCTTCCGGATCCTGGTGCTGGGCACGGCC
GCCGAGTCGTCCTGGGGCGACGAGCAGAGCGACTTCCTGTGCGACACCCAGCAGCCCGGT
TGCACCAACGTCTGCTACGACAGCGCCTTCCCCATCGCCCACATCCGCTACTGGGTGCTG
CAGATCGTCTTCGTCTCCACGCCCTCCCTCATCTACATGGGTCACGCCATGCACACCGTG
CGCCGGGAGGAGAAACAGCGGCGGAGGGAGCAGGAGGAGAGGGAGGCGAGGGGGGAGCGC
GGAGACAGCTTGGAGGAGAAGGAGTTCCTCCAGCAGAAGGAGAGCGAAAAGGCTCCGGCG
TCCGAAGGGAGCAGCCGCCTGCGCCTGAGAGGAGCCCTGCTGCAGACCTACATACTCAGC
ATCCTGATCCGCACGGTGATGGAGGTGACCTTCATTGTGGTGCAGTACCTGATGTACGGG
GTCTTCCTCAACGCCCTGTACCTGTGCAAGGCCTGGCCCTGTCCCAACCCTGTCAACTGC
TACATGTCCAGGCCCACGGAGAAGAACGTCTTCATCGTCTTCATGCTGGTCGTGGCCGGC
GTGTCCCTGCTGCTCTCCGTGCTGGAGCTCTACCACCTCGGCTGGAAGAGCCTCAAAAGG
TGTCTGCGCCAAAAGCTGATGGAAAAGAGCAGCCCGCAGGACTGTGGCGGTGGCGGTGTCG
GCGGCCCTGGAGCCCAACAGTCCGCCTCAGCCTTCTGTTTCCTGCACGCCGCCCCCAGAT
TTCAGCCAGTGCCTGGCAGTCTCAGGTTCCATCAACGCCATCGCCTCCATGGCCTCCCAC
CCCTTCAGCAACAGGATGGCGCTGCAGCAGAACTCGGCCAACTTGGCCACCGAGCGGCAT
CACAGCTCCGACAACCTGGAGGACGAGGCGGACTTCCTGAGGATCCGATACGACCAGCTG
CCCTCGGAGCTGCCCCGGAGCTGCTCGCCGTGCCCCCTCCTGCAGTCTGGCTTCATCAGG
GACAAACGGCGCCTGAGCAAGACCAGCGGGAGCAGCAGCAGACCTCGCCACGATGACCTT
GCAGTGTAA

>Tn-NN-gja5-G09857 Underlined: Sequence not included in Ensembl transcript prediction, but we consider it as a likely part of cds.

ATGGCAGACTGGAGCCTACTGGGAAACTTCCTGGAGGAGGTGCAGGAGCACTCCACCTCT
GTTGGAAAGGTGTGGCTGACCATCCTGTTTATCTTCCGTATCCTCGTGCTTGGACCGCC
GCCGAGTCATCCTGGGGAGACGAGCAAGAAGATTTCAACTGTGACACCGAACAGCCAGGC

TGCGAGAACGTTTGTTACGACCGAGCCTTCCCAATAGCGCATATACGATACTGGGTGCTG
CAGATTGTGTTTGTGTCCACGCCCAGCCTGATCTACATGGCCACGCCATGCACAGGGTT
CGCAGGGAGGAGAAGAGGAGGAACAGGGAGGAGGAAGGTGGGGAGGGGAGAGGTGGAGAG
GAGGACCCAGGAGGAGGAGGAAGAGGAGGAGATGACGGCAGAGAAGAAGATAAGAAAGGA
GGGAAAGAAGTGGCGGAGCAAGGAGAGAAGGAGAGCGGAGGTCGTGTGCGCTTGAGGGGA
GCGCTGCTGCAGACCTATGTACTGAGTATACTGATACGAAGCATCATGGAGGTGGTGTTT
CTCAGTCTCCAGTATTTCCTGTACGGGATCTTCCTCACTCCCCTGTATGTCTGCGAGGCC
TGGCCGTGTCCACATCCGGTGAACTGTTATGTCTCCAGGCCAACAGAGAAAAACGTGTTT
ATTGTGTTCATGCTGGCTGTTTCTGCCGTCTCTCTGGTTCTCAGCGTGCTCGAGCTGCAA
CACCTGGCCTGGAGGCACTGCTGCAGGAAGGCGGTAGCTGCTAATGAGGCCTCTCTGGGC
CGACAGCTCTCCTTGTCTCCTCCACCACCATCAACCCCACCTCCAGACTTCAGCCAGTGC
ATGATGGGCTCGACACACTTCCTACCTCTGGCTTTCCCCAGCCACCACCTGGTGCACCAA
CAGAACTCCGAGAACATGGCCACCGAGAAGCACAAAATCGCCGCCGCCGTCGAAGAGGCC
ACCCTCCTCCAGATGGGCTGCTACTCGCACGGATGGCAAAAGAGCAATCCCAGCCAGATC
CAGGAGGACGCCTACCTCAGGAAGGACAATAACTGCTACGGGCCCGGAGGCAGGAAGATG
AGCTGTCCGCAGATTCAGAATGGGGGCTCCGACAGGCTGCTGCTTTGCCCCGGCGGGGCT
CTCAGTCAGAAGGACAAGCGGAGGTTCAGCAAAACCAGCGGCACCAGCAGCCGAACAAGA
GCGGACGACCTGTCGGTTTAA

>Tn-gja8a-G13937 Underlined: Sequence not included in Ensembl transcript prediction, but we consider it as a likely part of cds.
ATGGGTGACTGGAGCTTTCTGGGTAATATTTTAGAGGAAGTGAACGAGCACTCTACGGTG
ATCGGCCGGGTGTGGCTCACGGTCCTCTTCATCTTCCGCATCCTCATCCTGGGCACGGCG
GCAGAGTTTGTGTGGGGGGACGAACAGTCAGACTATGTCTGCAACACGCAGCAGCCTGGC
TGTGAGAATGTGTGCTATGATGAGGCCTTCCCCATCTCCCACATCCGCCTGTGGGTGCTG
CAGATCATCTTTGTGTCCACGCCGTCTCTGGTGTACGTGGGTCACGCTGTGCACCACGTC
CACATGGAGGAGAAGCGCAAGGAGCGGGAGGAGGCAGAGCTCAGCCGGCAGCAGGAGCTG
AGCGAGGAGCGCCTCCCCTTGGCCCCCGACCAGGGTAGCGTCCGCACCACCAAGGAGACC
AGCACCAAGGGGAGCAAGAAGTTCCGGCTGGAGGGCACCCTGCTGAGGACCTACATCTGC
CACATCATCTTCAAGACGCTGTTTGAAGTGGGCTTCGTGGTGGGCCAGTACTTCCTGTAC
GGCTTTCGCATTCTGCCGCTGTACAAATGCAGCCGCTGGCCCTGCCCCAACACGGTGGAC
TGCTTCGTGTCCCGACCCACCGAGAAGACCGTCTTCATCATCTTCATGCTGGCTGTGGCC
TGCGTCTCTCTCTTCCTCAACTTTGTGGAGATTAGTCACTTGGGCCTGAAGAAGATTCGC
TTTGTCTTTCGCAAGCCGGTGCCGGCCCCGGCCCAGGGCGAGGGCTCGGCCCCGCTCCCG
GCCCCGGGCAAGAGTCTGCCCTCCCTCGCCGTGCCCTCCATGCAGAGAGTGAAGGGGTAC
AGGCTGCTGGAGGAGGAGAAAGCTCCCCCAATAACTCACCTCTACCCACTGGCCGAGGTG
GGCATGGAGGCCGGCAGAGGGAGCCCCCCCTTCCAGGGACTAGAGGAGAAGCCGGAGGAG
GTGCTGCCCATGGAGGACATCTCCAAGGTGTACGACGAGACTCTGCCCTCCTACACCCAG
ACCACCGAGACTGGGGGGGTGACACTACACGAGGAGGAAGAGGTAGAGGTAGAGGAGGAG
CAGCCAGCCGAAGCAGAGAAGGAGGAGGTGGTTGTGAGGGAGGAGGCAGAGGAGGTGGTG
AATGTGGAGGGGCCCAGAGCCGCGGAGGCCCCGGATACGATAGAAGACACCCGACCGCTG
AGCCGACTGAGCAAAGCCAGCAGCAGAGCCAGGTCAGATGATCTGACGGTATGA

>Tn-GJA9-G06130 As predicted by Ensembl
ATGGGAGACTGGAACTTCCTCGGAGGGATTTTGGAGGAGGTGCACATTCACTCCACCATG
GTGGGGAAGATCTGGCTCACCATTCTGTTCATTTTCCGCATGCTAGTCCTCGGCGTGGCG
GCGGAGGACGTGTGGAACGACGAGCAGGCTGACTTCATCTGCAACACCGAGCAGCCGGGA
TGCAGGAACGTGTGCTACGACCGGGCTTTTCCCATCTCCCTCATTCGCTACTGGGTGCTG
CAGGTTATTTTCGTGTCCTCTCCCTCGCTGGTTTACATGGGCCACGCTCTGTACAGACTG
CGGGCCCTGGAGAAAGCGCGGCAGAGGAAGAGAGCGCTGCTCCGGAAGGAGCTGGAGATG
GTGGGCGTGGATTTGGCCGAAGCTAGGAAGAGGATGGAGTGTGAGGTGAAGCAGCTGGAC
CAGGCCAGGCTGAACAAAGCCCCGCTCAGGGGATCCCTGTTACGCACGTACGTGGCCCAC
GTTCTCACTCGCTCCGTTGTCGAAGTGGCCTTCATGACGGGCCAGTACCTTCTTTACGGA
TTTCACCTCTACCCGCTTTTCAAGTGCGAGCGGGATCCTTGTCCTAATGCCGTGGACTGT
TATGTCTCCAGGCCCACAGAGAAAAGCCTTTTCATGGTCTTCATGCAATGCATCGCCGCC
ATTTCCCTCTTCCTGAACATTTTGGAGATCGTGCATCTGGGTTACAAGAAGATTAAACGG
AGCATCTTGGATCTTTGCCCGTTACGGGATGAACTGGAGGACGACTTTGCTGTTAAGGAC
AAAAGAGAATCTGTCGCACAGTTGTGCACCGCTGCGGCCCGGAAGATGACCATTACGTTT
TCACCGGCGGATGACAACGTGCTGCAGGGAACGGGGCGTCCAAACAATATCGCGCCAACG
GTTCTTCCTCTTCTGAGCGAGGCGTCCACTCAACTGGATCTGGAGGAATCCAGATGTGCG
TCCCAGCGTCCAAAAGACTGCAGCTGCGTGCTGACCGCGGCGGCTGGTGAGCGCCGCTCG
CCCTCCGTCGTTCCTCCAGAGCGGGAAAAGCAGAGCAGCGGAAGTGGCAGCCCGGATTGT
CCAAGACGCCCACCAAAGCCGGCACACGGATCCACCTTCCCCGCGCTGCCGGCGAACGCC
CCCAGGAGGCCTTGGAGGCCTCGTTCCTTTCAATGCGCCACAGTCCTGGAGGGGAAAAGC
TCTGACACCGACTCACGCGAGAGCGCCAGGGAGAGCAGCGAACCGCAGGGAAAACCTGGC
GCCTGCCGCCACAGCCTCAGCTCTGCGGCAGAGTCACCGGACGACTCCAGCGCGGGGTCC
GTGCACAGCCCCAGGCTGCCTTCCTCTTGCTGCAAAACATCAATAACAAGCAAAACCAGC
AGCGGTCGGGCTCCAGATCTGCAAATTTAA

>Tn-cx52.9-G05726 Underlined: Predicted as intron in Ensembl transcript. If this intron is used, the 3’-tail is extended.

ATGGGAGACTGGAACTTCCTTGGAGGAATCTTGGAGGAGGTCCACATTCACTCCACCATG
GTGGGCAAGATCTGGCTGACCATCCTGTTCATCTTCCGGATGCTGGTGCTGGGGGTCGCC
GCAGAGGACGTGTGGAATGACGAGCAGTCCGATTTCATCTGCAACACGGAGCAGCCCGGC
TGTCGCAACGTGTGTTACGACCAGGCCTTCCCCATCTCCCTCATCCGATACTGGGTGCTC
CAGGTGATTTTTGTGTCCTCCCCTTCTTTGGTCTACATGGGTCACGCCATTTATCAGCTA
CGAGCTCTGGAGAAGGAGCGCCACTGCAAGAAGGTGGCGTTGCGCCGGGAGATGGAAGCG
GTGGATGCGGAACTGGTGGAGGCGAGGAAGAGAATCGAGAAAGAGATGAGGCAGCTGGAG
CAGGGCAAACTCAACAAAGCCCCCCTGAGGGGCTCCCTGCTGTGTACTTACGTGGCCCAC
ATCGTAACTCGCTCGGTGGTGGAGGTCAGCTTCATGATGGGTCAGTACATCCTGTACGGA
CACCGCCTGAAACCTCTTTACAAGTGCGAGAGAGAGCCGTGCCCGAACGTGGTGGACTGC
TTCGTGTCCAGACCCACGGAGAAGACGGTTTTCATGATGTTCATGCAAGCCATTGCTTGC
ATCTCCCTCTTCCTCAGTCTCCTTGAGATTATCCACCTGGGATTTAAGAAGGGTGAAGAA
GGGCATCTTGGACTTTTACCCGCATCTGAAAGAGGACCCGGATGA

>Tn-cx52.6-G03863 Underlined: Not included in Ensembl prediction

ATGGGGGACTGGAATTTATTAGGAAGCATTTTAGAAGAAGTCCACATTCATTCGACCATC
GTGGGGAAAATCTGGCTGACCATTCTCTTCATTTTCCGAATGCTTGTTCTTGGCGTTGCG
GCTGAGGACGTCTGGGACGACGAGCAAAGCGAATTTGTTTGCAATACGGAGCAGCCCGGG
TGCAAAAACGTGTGCTACGACCAGGCCTTCCCCGTCTCCCTGATCCGTTACTGGGTCTTG
CAAATCATCTTCGTGTCCTCCCCGTCACTAGTCTACATGGGACATGCGCTGTATCGCCTG
AGGACTCTCGAGAAAGAGAGACACAAGAGGAAAGCCTGCCTGAAGGCTGAGCTGGAGGGC
ACAGACCCTGTCCAGGAGGACCACAGGAGGATTGAGCGAGAACTCAGAAAGCTAGATGAA
CAGAAGAGGGTGAGGAAAGCTCCTCTAAGGGGCTCCTTGCTGCGCACATACGTCCTCCAT
ATCTTAACCAGATCTGTTGTGGAGGTGGCTTTCATTATAGGACAATGTGCTCTGTACGGG
CTCGGGCTGTCGCCCCTGTACCGATGTACCAGACCGCCATGCCCCAACACCGTCGACTGC
TTTGTCTCTCGGCCTACAGAGAAGAATGTTTTCATGGTTTTCATGCTGGTTATCGCTGGC
GTTTCGTTGGCGCTCAACATTCTGGAGATCTTGCATCTGGGTGTGAAAAGGATTAAACAA
AGTTTGTATGGATATAAATACAGAGACGACGAGAGCGTGTGTCGCTCCAAGAAAAACTCC
ACCGTGCAGCAAGTTTGCCTTCTTGCTAGTTCTTCCCCTCAGAGGCTGGTGCAGCTGACC
CAAGTCACTTGCTCCGCTCTGCCCAACACTAATGCGACGAGTCTGTCCCATCAGAACCAG
GAAGGGTCCGGAAACGCCAACCAGCATCCCTCACACGCGTGCGTGCCCATCCAGGGTGTC
CAGCAGGTGGCACCGGCCGAGCAGCATCGCCTTTCGGGACTGAGGAAGCCGTCGTGCAGC
AGCGAGGAATCCAGCGAGCCTCACGTGAAGCCCCAGTACGCCGGCCCTCGAGCCACGCTC
GTGGCCAGCCACATGGAGATCCCGGCGGCCCTAAAGAACCCAGCACGGAAGCAGAGCAGA
GTCAGCATTTATAAAGAGCTCAGTGACATGAGCGACTCTGCCGAGAGCGAGCCCCACCTC
CCAGCCCGCAAATGTAGCTTTATGTCTCGGGGCCTGTCGGACGGAAAGCTGTCCAACCCA
TCCGACAGCGCCGACAGCCGCAGCGGAACAGACTCGGAAGCCCAGCACCTCAACCAATCA
GAGAGCTCAGTGGTGACCCCACCGCCTCCAGCCAGTGGCAGAAGGATGTCCATGGTTAGT
GGGCCGGGTTCATTTTTCCACCACAAAACTAGACACACGTGA

>Tn-cx34.5-G19149 Underlined+italics: Ensembl ENSTNIT00000022585 predicts a 63 nt long insert here. Splice sites.

ATGGGGGAATGGGATTTGCTGGGCCGCCTGCTGGATAAAGTGCAGACTCACTCCACGGTT
CTGGGCAAGATTTGGCTCACGGTGCTCTTCGTCTTCCGCATCCTGGTGCTGCAGACGGCT
GCCGACAAGGTGTGGGGGGACGAGCAATCGGACTTCATCTGCAACACTCAGCAGCCAGGC
TGCGAGAACGTCTGCTACGACCTGGCTTTCCCCATCTCCCACGTCCGCTTCTGGTTCCTT
CAGATCATTGCCATAGCGACGCCCAAGCTGCTCTACCTCGGCCACGTCCTTCA*CG*CGTGT
GTGTTCCTCAGGAGCTACAAAGTTCCCAAGTACGTCAAAGGCTCGGGCAAGATCAGCATC
CGCGGGCGCCTCCTCCGCAGTTACACCTTCCACCTGATGGCCATGATCATCCTGGAAGGC
GCCTTCATCGCCAGCCAGTACCTCCTCTTTGGCTTCGCTCTGGAGACGCGCTACGTGTGT
GAGCGCCACCCCTGCCCCCACAAGGTGGACTGCTTCCTGTCCAGGCCCACGGAGAAGTCG
GTCATCATCTGGTTCATGCTGGTGGCGGCCGTGGTCTCCCTGGCCCTCAGCCTGGCCGAG
CTCTTCTACCTGGGCCTCAAAGCCACCAGGGAGTGCATGGCCAGGAGGCAGGACTACACG
GTGACGCCCGTGACGCCGCCCGTTTCGGGGAGAAAAGCCTTCAAAATCTCCGATGAGATG
ATCCAGAACTGCATCAACCTGGAGCTGGAGCAGCTTAAAGAAAAGAAGGTCCGGAGGGTC
GCCGGGGGGCCCGAGGAGGTGCCCAGTGTCGCCCCGCCCGGCAACAGGAACAAGGGAGAG
GTCCACATCTGA

>Tn-cx32.3-G19150 No modifications. This sequence corresponds to the predicted transcript ENSTNIT00000002345.

ATGGGAGACTGGGGATTCTTGTCGTCCTTGCTGGACAAAGTCCAGTCCCACTCCACCATC
ATCGGGAAGATCTGGATGAGCGTCCTCTTCCTGTTCAGGATCATGGTCCTGGGCGCCGGC
GCCGAGAGCGTCTGGGGCGACGAGCAGTCCGGGTTCATCTGCAACACTCAGCAGCCCGGT
TGCGAGAACGTCTGCTACGACTGGACCTTCCCCATTTCCCACATCCGCTTCTGGGTCCTC
CAGATCATCTTCGTGTCCACGCCGACGCTGGTGTACCTGGGCCACGCCATGCATGTCATC

CACCAGGAGAATAAGCTGAGGGAGAAGCTGCAGAGCCCCGGCGGGAGCCGCTTGCTCAAG
GTGCCCAAGTACACCGACGAGAAGGGGAAGGTGAAGATCAAGGGGAACCTGCTGGGGAGC
TACCTGACCCAGCTGGTCTTCAAGATCCTCATCGAGGCGGCCTTCATCGTGGGCCAGTAC
TACCTGTATGGCTTCATCATGGTGCCGATGTTCCCTTGCTCCAAGAAGCCCTGTCCCTTC
ACCGTGGAGTGCTACATGTCCCGCCCCACCGAGAAGACCATCTTCATCATCTTCATGCTG
GTGGTGGCCTGCGTCTCGCTGCTCCTCAACGTCATCGAGATGCTCTACCTCCTGTGCACC
AGGCTCAAATGCGCCTCCAGATCGCGGACGCAGAAGCTGACGTCGGCCCAGAGCCCCGCC
GGCCTGCTGGCCCCGAAATGGCCGACGGCGGAGGACGCGCTCCATCAGAACCGGATCAAC
CTGGAGCAGGAGCGCTGCCAGAGCGTCGGCGGGAGCCTGGACGGCGCCAAGGAGGAGATG
AAGCTTCTGCATCACAACTGA

>Tn-NN-gjb1-G08980 Note that our sequence is only a minor part (the 3’-end) of the predicted Ensembl transcript
ATGAACTGGGGAACCTTTTACGCCCTCATCAGCGGCGTGAACAGGCACTCAACGGGCATC
GGAAGGGTTTGGCTCTCCGTCATCTTCGTCTTCCGAATCCTGGTGTTGGTGGTGGCTGCC
GAGAGCGTCTGGGGCGATGAGAAGTCGGGCTTCACCTGCAACACCCAGCAGCCCGGCTGC
AACAGTGTCTGCTACGACCAGTTCTTCCCCATCTCGCACATCCGCCTGTGGGCTCTGCAG
CTGATCTTGGTCTCCACCCCGGCCCTGCTGGTGGCCATGCACGTAGCCCACAGACGCCAC
ATCGACAAGAAGATCCTGAAGAGGGCCGGCCGTGGCACACCCAAAGACCTGGAGCAGATA
AAGAACCAGAGGTTCCAGATCACTGGAGCTCTGTGGTGGACGTACATGATCAGCATCATC
TTCAGGATCGTCTTTGAGGTGGCTTTCCTCTACATCTTCTACCTGATTTATCCAGGTTTC
AAAATGGTGCGTCTGGTCAAGTGTGACTCTTACCCTTGCCCCAACACCGTGGATTGTTTT
GTGTCCAGACCCACTGAGAAAACCATATTTACAGTGTTCATGCTGGGGGTCTCGGGGGTG
TGCGTGCTTCTGAACCTGGCGGAGGTGGTCTACCTCATCGGCCAGGCCTGCAGACAGTGC
ATCCGAGGCTCTGAAGAAACCTCCAAAGTCCCCTGGATCAGTCAAAAATTGTCCTCTTAC
AGGCAAAATGAGATCAACGAACTGATACTGGACCATCCCCTCAGGTCAAAGTTCAGTGTG
ACCAAAAAGAAGCCCAGCTGA

>Tn-cx27.5-G11825 Note that the predicted Ensembl transcript extends another 42 nucleotides on the 5’-end
ATGAACTGGGCATCGTTCTACGCCGTCGTCAGCGGCGTGAACAGACACTCCACGGGCATC
GGCCGCATCTGGCTCTCCGTGCTGTTCATTTTCCGCATCCTGGTCCTGGTGGTTGCTGCA
GAGAGCGTGTGGGGAGACGAGAAGTCGGGCTTCACCTGCAACACCCAGCAGCCGGGCTGC
AACAGCGTCTGCTACGATCACTTCTTCCCCATCTCCCACATCCGCCTCTGGGCTCTCCAG
CTCATCCTGGTCTCCACTCCGGCCCTGCTGGTGGCCATGCACGTGGCTCACCGCCGCCAC
ATCGACAAGAGGCTCTACAAGCTGTCAGGGCGCACCAATCCCAAAGACCTGGAGCAGATT
AAGACCCAGAAAATGAAAATCACAGGCGCACTCTGGTGGACATACGTCATCAGCCTGCTT
TTCCGCGTTATCTTTGAGGTGACCTTTATGTACCTGTTCTACATGATCTATCCTGGTTAC
AAGATGATCCGGCTGGTGAAGTGTGACTCGTACCCCTGTCCCAACACGGTGGACTGCTTT
GTCTCCAGGCCCACAGAGAAGACTGTTTTCACCGTCTTCATGCTGGCTGTGTCAGGGGTT
TGTATTTTGCTCAACATTGCGGAGGTGGTCTTCTTGGTGGGGAAGGCCTGCGGTAGGCAT
TTACAGCACGCTGGAGACTCCGCTGTGGGAACCTGGATCCAGCAAAAACTCTGCTTCCTT
TAG

>Tn-NN-cx28.9-G19153 Splice site. Two 100% identical predictions are located approx. 10000 nt apart (G19153 and G19151).
ATGGGAGAGTGGGGTTTTCTGTCCTCTCTGCTGGACAAGGTCCAGTCTCATTCCTCCGTC
ATCGGGAAGGTCTGGCTCAGCGTGCTCTTCATCTTCAGGATCATGGTTCTGGGAGCTGGA
GCTGATAAGGTTTGGGGCGACGAGCAGTCCAATATGATTTGTAACACCAAGCAGCCCGGC
TGTAAAAACGTCTGCTACGACCACGCCTTCCCCATCTCGCACATTCGTTTCTGGGTCCTC
CAGATCATCTTCGTCTCCACGCCCACGCTGGTCTACCTGGGACACGTCCTGCACGTCATC
CACAAGGAAAACAAGATGAGAGAGTACATGAAGACGCACACTCAGAGCAACCTGGCCAAA
TACCCCAAGTACACCGACGAGAAAGGCCACGTGGAGATCCGGGGCAACCTCCTGGGCACC
TACATGACCTCCATCGTTTTCCGCATCCTTCTGGAGATCGCCTTCATCGTGGGCCAGTAC
TACCTGTACGGCTTCATCATGGACCCCAAAGTGGTCTGCTCCCGGGCCCCCTGCCCCTTC
ACCGTCGAGTGCTACATGTCTCGTCCCACCGAGAAGACCATCTTCATCCTCTTCATGCTC
GCGGTCTCTTGCGCGTCGCTGCTGCTTAACGTGGTGGAGATCTGCTACTTGGTGTGCTCC
CGCTCGAAGAAAAGAGCCAAGACGCCGCCGGCGTCCGCGCTCGTCATTCACCCACGGTTC
ACCAGCGAAAGCAAAGCCTGA

>Tn-NP-cx30.3 Note that this sequence was included in our previous analyses (Cruciani and Mikalsen, 2007) as Tn29165001.
ATGTCTTGGGCCGCACTTATTAGTAAGCTGGGTGGTGTCCACGAATACTCCACCAGCCTG
GGGAAGGTCTGGCTTTCTGTCCTCTTCATCTTCCGCATCGGTATTCTGGTTGTGGCCACC
GAGAAAGTCTGGGGAGACGAACAGTCCAGCTTTACGTGCAACACACAGCAGCCGGGCTGC
AAAAACGTCTGCTACGATCACTTCTTCCCGGTTTCACACATCCGCTTGTGGTGCCTGCAG
CTGATCTTTGTGTCGACCCCGGCCCTTCTGGTGACCATGTACGTGGCCTACAGAAAACAT
AAAGATGAGAAAAATTGTTTAGACTCCAAGGACAGTGAGACGGGGAAGGAGGAAGAGACG

GGAAAAAAGGTTAAAAAAGAGGAACAAAAAAAGCGTCTGCCCATCACAGGTCCTATGTGG
TGGATCTACACCAGCAGCTTGTTCTTCAGACTGCTCTTTGAGGGGGGCTTCATGTACGCT
CTGTACTTCATCTATGATGGCTTCCAGATGCCACGTCTGCTCAAGTGTGAGCAGTGGCCT
TGCCCCAACAAGGTGGACTGCTTCGTCTCCAGGCCGACGGAGAAAACGGTCTTCACCATC
TTCATGGTGGCCTCGTCCGTCATTTGTATGGTTCTTAATTTTGCCGAACTCATCTACCTA
ATTGGCAAGGCCCTCTTTAAGAGAAGTAGTGGGGGGAAAAAATGCACTATAGAATTAAAC
TCCAACCAGAATACCATGCTTTTGGAGAAAAAATTAA

>Tn-cx30.3-G01258 Underlined: Sequence not included in Ensembl transcript prediction, but we consider likely as a part of cds.

ATGTCTTGGGCCGAACTTTATAAGCGGCTGGGCGGCGTCAACAAACACTCCACCAGCCTG
GGGAAGGTCTGGCTTTCTGTCCTCTTCATCTTCCGCGTCTCTATTCTGGTTCTGGCCGCC
GAGAAAGTCTGGGGAGACGAACAGTCCGACTTTACGTGCAACACACAGCAGCCGGGCTGC
AAAAACGTCTGCTACGATCACTTCTTCCCGGTTTCACACATCCGCTTGTGGTGCCTGCAG
CTGATCTTTGTGTCGACCCCGGCCCTTCTGGTGACCATGTACGTGGCCTACAGAAAACAA
ACAGATTGGAAAAAAGGTTTAGCCTCCAAGGACAGTGAGACGGGGAAGGAGGAAAAAGTG
GAGAAGGGCGGTGAGAAGAATGAGGAGGAAATACTCATATTGCTTGAAGAGGGGGAAAGT
AATTCAGTCCCCGAGAACAGTAAGAAAGTGAAGGAGGAAAATACGACAGAGTTAAAGAAT
GATAAGGACAATGAGAAGAAAAAGGGGAAAAAAGAGGAACAAAAAAATGAGCGTCTACCC
ATCACAGGCCCTCTGTGGTGGATCTACACCAGCAGCTTGTTCTTCAGACTGCTCTTTGAG
GGGGGCTTCATGTACGCTCTGTACTTCATCTATGATGGCTTCCAGATGCCACGTCTGGTC
AAGTGTGAGCAGTGGCCTTGCCCCAACAAGGTGGACTGCTTCGTCTCCAGGCCAACGGAG
AAAACGGTCTTCACCATCTTCATGGTGGCCTCGTCCGTCATTTGTGTGGTTCTTATTTTT
GCCGAACTTTTTTACCTTATTGACAAGGCCCTCTTTAAGAGAAGTACCATAGAATTAAAC
TCCAACCAGAATACCATGCTTTTGGAGAAAAAATAAAAATAAAGTTCTGTAA

>Tn-cx30.3-G15674 Underlined: Sequence not included in Ensembl transcript prediction, but we consider likely as a part of cds. N:Nucleotide added to keep reading frame.

ATGTCTTGGGCCACACTTTACAGTCAGCTGGGCGGCGTCAACAAACACTCCACCAGNCTG
GGGAAGGTCTGGCTGTCTGTCCTCTTCATCTTCCGCGTCACTATTCTGGTTCTGGCCGCC
GAGAAAGTCTGGGGAGATGAACAGTCCGACTTTACGTGCAACACACAGCAGCCGGGCTGC
AAAAACGTCTGCTACGATCACTTCTTCCCGGTTTCACACATCCGCTTGTGGTGCCTGCAG
CTGATCTTGGTGTCGACCCCGGCCCTTCTGGTGGCCATGTACGTGGCCTACAGAAAACGT
GGAGATAAGAGAACTGTCCTGGCCTCCGGCGGGGATGAGAAGGTGAAGGAGGCAGACCTG
CAGACCCTGAAGACGAAGCGTCTGCACATCACAGGCCCTCTGTGGTGGACCTACACCAGC
AGCTTGTTCTTCAGACTGCTCTTTGAGGGGGGCTTCATGTACGCTCTGTACTTCATCTAT
GATGGCTTCCAGATGCCACGTCTGGTCAAGTGTGAGCAGTGGCCTTGCCCCAACAAGGTG
GACTGCTTCATCTCCAGGCCGACGGAGAAAACGGTCTTCACCATCTTCATGGCGGCCTCG
TCCGCCCTTGTATGGTTCTTAATATTGCTGAACTCGTCTACCTGATTGTCAAGGCTCTC
GTGCGGTTATCAGCCAGGTCCAAGCAGCGGAAACAGAGATACGCTCGGGAGAACTTCCAC
CGGGATCACATGCTTCTGGAGAACAAGAAGAATGAGAACATGTTTTCCTCAGACCCAACA
AGCAACAGAACCATGTGTTGA

>Tn-NN-cx30.3-G10340 Underlined: Sequence not included in Ensembl transcript prediction, but we consider it as a likely part of cds. Splice sites.

ATGTCTTGGGCTGCTCTGTACAGCCAGCTGGCTGGAGTAAACCGCCACTCCACCAGTCTG
GGGAAAGTCTGGCTCTCTGTGCTCTTTATTTTCCGAGTCATGGTTTTGGTTGTGGCTGCT
GAGAGCGTTTGGGGGGATGAACAGTCGGACTTCACCTGTAACACCCTACAGCCTGGCTGT
GAGAACGTCTGCTACGATCAGTTCTTCCCTGTCTCCCACATCCGGCTCTGGTGTCTTCAG
CTTGTCTTTGTTTCCACCCCAGCGCTCCTGGTGGCGATGTACGTGGCCTACCGGAACCAC
GGCGATAAGAAGAAGCTCCTACAGAATTCTGGAAGAGTTGGGATCTTGAGCACGGAAGGT
CCAGAGGAGCAGCTGGAGAGCCTCAGGAGCAGGAGGCTGCCCATATCTGGCGCTCTCTGG
TGGACGTACGCCTTCAGCCTTCTGTTCAGGCTCCTGTTTGAAGGAGGTTTCATGTACGCC
CTCTACGTGATTTACGACGGTTTCCGGATGCCACGCTTGGTGCGGTGCGACCAGTGGCCG
TGCCCCAACCTAGTGGACTGTTTCATCTCACGGCCGACAGAGAAAACAGTCTTCACCGTT
TTCATGGCCACCTCATCATCCATCTGCATGCTCCTCAACGTGGCAGAGCTCGCATATCTT
GTTGGCAAGGCTGTCACAAGCCCACCAGCAGGAGTAAAAGACGTGAAGAAGAGAAGTGAG
AGAAGACTGGTGCTGTTTGACAGGAGAGCGGTAAGCAGGGTGGAGGCTGCCTGGACTAAT
GCTGCTGCTGAGGTGGGAGGAGGAATTAGGAGGAGGGATTCTCATCCTTCCACCTGCTGG
ATTATGAGCTTCTCTCATGGTGCGTTCACGTCCATGTGGGAAAGACTGCAAACAGGAAAA
CTGGCATATTTCAGAATGATTATAGTCAGTATTGTTGTAAAATTGTTGTAA

>Tn-cx35.4-G16899 As predicted by Ensembl.

ATGGACTGGAAGACCTTCCAAGCCCTCCTCAGCGGGGTGAACAAGTACTCCACCGCGTTT
GGGAGGGTCTGGCTGTCGGTGGTGTTCGTGTTCAGGGTGATGGTGTACGTGGTGGCGGCG
GAACGCGTGTGGGGCGATGAGCAGAAAGACTTCGATTGCAACACCAAGCAGCCGGGCTGC
GCCAACGTCTGCTACGACTACTTCTTCCCCATCTCCCACATCCGCCTGTGGGCCCTGCAG

CTCATCTTCGTCACCTGCCCGTCCTTCCTGGTGGTCATGCACGTGGCGTACCGGGATGAG
CGCGAGCGCAAGTACAGGGCCAAGCACGGCGATGAAAGCAAGCTGTACAACAACACGGCC
AAGAAACACGGCGGCCTGTGGTGGACCTATCTGCTGAGCCTGTTTGTGAAGACGGGCATC
GAGGTGGCCTTCCTCTACATCCTCCACCTCGTCTACGACAGCTTCTACCTGCCGAGGCTG
GTCAAGTGCGAGGTGGCGCCCTGCCCCAACCAGGTGGACTGCTACATCGGACACCCCACT
GAGAAGAAGGTCTTCACATACTTCATGGTTGGCGCCTCGGCCCTCTGCATCGTCCTCAAC
ATTTGCGAGATCATTTATCTCATCTCCAAGCGCATTGCGCGGTGCGCCAACAAGCACAGG
AGGCACCATCGCAATCCGGCCGTACACCCTCCAGACGAAGAACACAACATGGACGACCCC
TTCAGCAACCACAAAGCCATGGAGCCCAAGCCAGGGCTGAAGGAAAGGCCCCCGTCCTTT
AACACCGCCTCCAAGTTCCCATATAACATGGACAGCTTCCGGATGGCCGACAAGATCCGA
GCCTCCGCCCCCAATCTGTCTTCATGA

>Tn-cx28.6-G08925 Grey font: Intron? Underlined: Sequence not included in Ensembl transcript prediction, but we consider likely as a part of cds. Splice site.
ACTTTCCAGCCGGGCTGCACCAACATATGCTACGACCACATCTTCCCCATCTCCCACATC
CGCCTGTGGGCGCTGCAGCTGATTTTCGTCACCTGCCCGTCGCTGATCGTGATGGCTCAC
GTCAAATTCCGCGAAGGGAAAGACGCCAAGTACGTGGAGCAGCACCACGGCTCCCACCTC
TACAGCAACCCTGGCAAGAAGAGAGGCGGGCTGTGGTGGACCTATCTCCTCAGTCTGATC
CTCAAAGCCGGATTTGATGCTTCCTTTCTTTACCTCCTGTACAGAATCTATCACGGTTAT
GATCTGCCCAGATTATCGAAATGTTCGCTGGAACCGTGTCCCAACACGGTGGACTGCTTC
ATCAGCCGTCCCACGGAGAAAAAGATCTTCATGCTCTTCATGGTCGTCTCCAGCGCTCTT
TGCATTTTCATGTGCATCTGCGAAATGTTCTATCTCGTCGGAAAACGCATCGCCAAACGG
GTGCAGAGCCACCGCGAGAACAAGCAGATTCTGTTTGCTGACCAGCACGAACTGACCAAC
ATGGTTCCACCCAGATCCCAGTATCGGGAGACTGACCCCACCCTGACGGGCAGTCAGCTG
AGCCTGGGCAGAAGGGACAAGGTCAGGGAGGAGGCCGTGACAACGACCCTGTAA

>Tn-cx30.9-G09221 Underlined: Sequence not included in Ensembl transcript prediction, but we consider likely as a part of cds. Splice site.
ATGAACTGGTCTGCCCTGGAGGCCCTGCTCAGTGGGGTCAACAAGTATTCCACTGTGTTC
GGACGCGTCTGGCTGTCCATGGTCTTCGTCTTCCGCGTGATGGTGTTTGTGGTGGCGGCT
CAGAGGGTGTGGGGTGACGAGAGCAAGGACTTTGTCTGCAACACAGCGCAGCCGGGCTGC
AACAACGTCTGCTACGACAGCATCTTCCCCATCTCACACATCCGCCTGTGGGCCCTGCAG
CTCATTTTCGTCACGTGCCCGTCGCTGATGGTGGTGGGGCACGTCAAGTATCGGGAGAAG
AAAGATTCCCAGTACAGCACCTCACACCACGGGAAGCATCTGTACGCCAATCCTGGAAAG
AAGCGCGGAGGGCTGTGGTGGACCTACCTGGTGAGTCTGATTTTCAAGGCCAGCTTTGAC
GCTGGTTTCCTGTACATCCTCTACCACATCTACGATGGTTACGACATGCCCCGCCTGTCC
AAGTGTTCCCTGGAGCCGTGCCCCAACACGGTGGACTGCTTCATATCCCGGCCCACCGAG
AAGAAGATCTTCACCCTCTTCATGGTGGTCTCCTCTGCCATCTGCATCCTGATGTGCATC
TGTGAGATGATCTACCTCATCTGCAAGCGCATCAGTAAACTCATTAAGAGAAGGAACGAG
GCAGAGAGGAGGCTGTTTGCTCAGCAGCACGAGATGACGCCGCTGGCACCACCGAGGTCA
GAGCTGAGGTCCAAATCGCCGATCAGGGTGGATCCAACAGCCTCAATCCAAGATCTTGGC
GCCATCGCTGAGGACAAGCAACCGCCTGAGAAACAACAGGTCACAGCGTAG

>Tn-cx34.4-G16900 Underlined: Sequence not included in Ensembl transcript prediction, but we consider likely as a part of cds.
ATGAACTGGGCATTCCTCCAGGGCCTCCTCAGCGGGGTGAACAAGTACTCCACCGCCTTC
GGCCGAGTGTGGCTCTCCATCGTCTTCCTCTTCAGGGTCATGGTGTTCGTGGTGGCGGCG
GAGAAGGTCTGGGGCGACGAGCAGAAGGACTTCAAATGCAACACGGCTCAGCCTGGTTGC
CACAACGTCTGCTACGACTACTTCTTCCCCGTGTCCCACATCCGGCTGTGGGCCCTGCAG
CTCATCTTCGTCACCTGCCCCTCTCTCCTGGTGGTGATGCACGTGGCGTACCGGGAGGAC
AGGGAACGGAAACACAGGCTAAAATTCGGCGAAAACTGCCACCGTATTTACCAGAACACT
GGGAAGAAGCGCGGAGGCCTGTGGTGGACCTACGTCCTCACTTTGGTCTTCAAAATCGCC
GTAGACGCCGTCTTCGTCTACCTTCTCTACCACATCTATGAGGGCTACGACTTCCCCTTG
CTCATCAAGTGCCAGCAGAAGCCCTGCCCCAACATAGTGGACTGCTTCATCGCTCGCCCC
ACCGAGAAGCGCATCTTCACCATCTTCATGGTGGTCACCAGCCTGGTCTGCATCTTCCTC
TCCCTCCTGGAGATCCTCTACCTGGTGGGCAAACGCTGCCACGAGTGTTTCAAGGCCGTT
CACGACTCCCACCGCATCGTGACCGCCGCCATCTCCAGCGGAACCAACATGATGGAGTCC
CGGGTCGCAAAAACCAGCCCCGAAAGTCTGGCGCCCTTGTACAACTCTGCCAAAGCGGAC
AGCCGGACAGCCAGGGACTCTGCCCAGACTCTGAAGTTCACCTGA

>Tn-cx28.8-G19193 Underlined: Sequence extended in 3'-direction until stop codon.
ATGAACTGGAGCTTTCTGGAGAACATCCTCAGTGGGGTGAACAAATACTCCACAGTCATT
GGGCGCGTCTGGCTCTTTGTGGTGTTCCTCTTCAGGATCTTGGTGTACGTCGTAGCGGCC
GAACAAGTGTGGAAGGAAGAGACGAAGGAGTTCGTGTGCAACACCCGTCAGCCCGGCTGC
GAGACCACCTGCTTCAACCACTTCTTCCCCTGTTTCGCAGGCGCGTCTCTGGGCCATGCAA
CTCATCCTGGTGTCTACCCCATCTCTGCTAGTGGCCCCTGCATGTGGCATATAGGGAACAC
CGAGAGGCCAAGCACAAGAAAAAACTGTACCAGGACAAGGCGACCATTGACGGGGGATTG
CTTTTCACTTACATCGCCAGTCTCATTTTTAAGACGGCGTTCGAGGTGGGCACCCCTGCTG

GTGTTCTACTACGTCTACAACGGTTTTGAGCTTCCGGCGCTGCTCCGCTGCGACCAGAGT
CCCTGCCCAAACGTAGTGGACTGTTTCATCGGCAAAGCCACCGAAAAGAAGATTTTCCTC
TACATCATGGCCTGCACATCTATCCTCTGCATCGTTTTGAATGTCGTTGAGCTTATTTAC
ATCGTATGGAAGCAAGTCGTTAAATACGTCATCCAGCATTACAATCCTGTGGAGAAGAGA
CCTCCCTCTGGCAACCAGACACAAGTGTCCAACGTCAATGGATATGTCTCTGTTGACCAC
GTTGGAAATGAAGAAGACAGCCCAAAAAGACTAAACCATTAG

>Tn-NN-gjc1-G00149 Underlined: Sequence not included in Ensembl transcript prediction, but we consider likely as a part of cds.
ATGAGCTGGAGTTTCCTGACTCGCCTGTTGGAAGAAATTCACAACCATTCCACGTTTGTG
GGCAAGATCTGGCTCACTGTCCTGGTTGTCTTCCGCATCGTGCTGACGGCCGTTGGCGGC
GAGTCCATCTACTACGATGAGCAGAGCAAGTTTGTGTGCAACTCGGGCCAGCCGGGCTGT
GAGAACGTCTGCTACGACGCCTTTGCTCCGCTGTCGCATGTCCGCTTTTGGGTGTTCCAG
ATCATTCTGGTGGCCACCCCTTCGCTCATGTACCTGGGATATGCCGTCAACAAAATCGCT
CGCACAGAGGAGCAGGTGGGTGGGATGGGAGTGAGGGGATGTTCGCAGAGGAAGCTCAAG
AGAAAGCTGTATCTGGCAGACAGAAAGCAGCACAGAGGCATTGAAGAAGCTGAGGATGAC
CAAGAGGAAGACCCTATGATCTATGAAATGGCAGAAGTGGGGAGCGACTGCAGTGAAGAA
ACAAAAGGCAATGTTGTTGGAAAAGATAAGGTCAAGGTCCGCCACGATGGACGCCAGCGT
ATCAAAGAGGATGGTCTGATGCGTATTTATGTCCTTCAGCTCCTGGCCCGCTGCTTGCTG
GAGGTGGCTTTCTTGTGCGGGCAGTACGCCCTGTACGGATTCGCTGTTCCCCCTACCTAT
GTCTGTTCTCAGCTGCCCTGCCCCCACAGCGTGGACTGCTTCGTGTCCCGGCCCACTGAG
AAGACCGTCTTCCTCATCATTATGTACATCGTCTCCCTGCTCTGTCTGATGCTCAACATC
TGGGAGATGCTTCACCTGGGCATCGGCACCATCTGCGAGATCATTCGTTCCCGGCGGGTC
CCCGAGGAGGAGCTGTACGGGCTGACACAAGCGAAAGAGCCCCACGCCAGAGAGGATTAC
AGCAGCTACCCTTTCTCCTGGAACGCGCCATCAGCTCCGCCTGGGTACAACATCACAATC
AAGCCCCCAATGGTGCCGGCAGAACGCCACGATCAACCCCTACCGGTCACCGACCTCACC
AGCGCGAAGATGGCATGCCGACAAAACCACGCTAACATCGCGCACGAGGAGCAGCAACAG
TACAACAATAACGACGAAAACCTTCGCAGAGCCGGGATGGGAGATGACCGCACGCGTTCC
CATCACTCTCAGAACAGACTGGAGATGGACGCGTCAGCCCACAGCCAGCCGCAGGGCCAA
AACAACAACAAGCCTCACCGCGACCGCAAACACCGCCAGGCCTCCAAACATGCGTCCGGC
AAGGCTGACGCAGACCGAGGCGGCAGCAGCACCAGCAACACCAGCAAATACGGAGTCATC
AAAGGCTCCGAGTGGATCTGA

>Tn-NN-gjc1-G05345 Underlined: Sequence not included in Ensembl transcript prediction, but we consider likely as a part of cds.
ATGAGCTGGAGCTTCCTCACGCGGCTGCTGGAGGAGATCCACAACCACTCCACCTTCGTG
GGGAAGCTGTGGCTCACCGTGCTCATCGTCTTCCGCATCGTCCTCACCGCCGTCGGGGGA
GAGTCCATCTACTACGACGAGCAGAGCAAGTTCGTGTGCAACTCGGGACAGCCGGGCTGC
GAGAACGTCTGCTACGACGCCTTCGCGCCGCTGTCCCACGTCCGCTTCTGGGTTTTCCAG
ATTATCCTGGTGGCCATGCCCTCCCTCATGTACATGGGCTACGCCATCAACAAGATCGCC
AGGCTGGAGGAAGCCAAAGGAGGCGGGGCCTCGGCGGCCATCAGGACGGGAGGCGGAGGC
TACACGCACAGAAAGCCCAGGAAGATCTGTTTCGGAGCGCGGCAGCACCGGGGCATCGAG
GAGACCGAGGAGGACCAGGAGGACGACCCCATGATCTACGAGGTCCCGGAGGTGGAGCCC
CCCAAGAGGCCCCGGGACCCGCTGCAGCCCACGCCCAGACCCAAAGTCCGGCACGACGGG
CGCAAGCGGATCAGAGACGAGGGCCTGATGCGGGTTTACGTGCTGCAGCTGGTGACCCGT
ACCGTGCTGGAGGCGGGCTTCCTCGCCGGCCAGTATCTGCTCTACGGTTTCCGCGTGATG
CCCGTGTTCGTGTGCTCGGGGAGACCGTGCCCCCACAGCGTGGACTGCTTCGTGTCGCGC
CCCACGGAGAAGACCATCTTCCTGCGCATCATGTACGGCGTCACCGTCCTTTGCCTCGTC
CTCAACGTCTGGGAGATGCTCCATTTAGGGGTGGGCTCCATCTACGACATCCTCCGCCGC
CGGCGCGCCCCGCCCCAGGACGATGAGTACCAGCTGGGCTTGCTGGGCGCCAACGGAGCC
GTGGAGGGCTCCGTCGGGGGCACGGCCCCCGAGGCGGGTTCCGAAGGAGGGGTGGGCGGC
GACGGGGCCGCGGACTACGTGGGCTACCCTTTCTCGTGGAACACGCCGTCGGCTCCGCCC
GGCTACAACATCGTGGTCAAACCCGAGCAGATGCCCTACACGGACCTGAGCAACGCCAAG
ATGGCGTGCAAGCAGAACCGGGCCAACATCGCCCAGGAGGAGCAGCAGCAGTTTGGTAGC
AACGAAGACAACTTCCCCACGGGGGGAGAAGCCCGCGTGGCTTTGAACAAAGACATGATC
CAGCAGGCTCACGAGCAGCTGGAGGCGGCCATCCAGGCCTACAGCCAGCAGCACCAGGCC
GAGGTGCAGCTGGGGGACAACCAGGACGACAAACCCCAAAGCAACATCATCCAGGCGCAG
CCGCTGCTGCAGCCGCAGCCTCAGAAGGAGCGCAAGCATAGATTCAAGCACGGGAAAGGA
GGCAGCAGCGCAGGAGGCAGCAGCAGCAACAGCAGCAGCAGCAAGTCGGGGGAGGGGAAG
CCCTCCGTGTGGATTTAG

>Tn-cx47.1-G08482 As predicted by Ensembl
ATGAGCTGGAGCTTCCTCACACGTCTCCTGGAAGAGATCCACAATCATTCCACATTTGTG
GGGAAAGTGTGGCTGACAGTGCTCATCATCTTCCGCATTGTGCTCACGGCAGTCGGAGGC
GAATCCATCTACTCGGACGAGCAGACGAAGTTCACCTGCAACACCAAGCAGCCGGGCTGT
GATAACGTATGCTACGATGCCTTCGCCCCTCTCTCGCACGTCCGTTTTTGGGTTTTCCAG
ATCATCATGATCTCCACCCCTTCCGTCATGTACATGGGCTATGCTATTCATAAGATAGCG
CGGAGTTCGGATGAAGAGCGCAGAAAGCTCCACAGGCTTCGCAAAAAGCCCCCCACCACAT

TCCAGATGGAGAGAGAACCATCACCTGCAGGGCGTCTTAGAGGAGGACGAAGACGACGAC
GCTGAGCCCATGATCTATGAGGATACGCTAGAGGTTCAAGATGCCAAACCAGGACCAGGG
AACAGCGGTAGCAAAAACCCACCGAAATATGACGGCCGTCGAAAAATTATGCAGGAAGGT
CTAATGAGGATCTATGTCCTTCAGCTGATGTCAAGAGCTGTTTTTGAAATTGCCTTCCTT
GCTGGACAGTACCTCCTGTATGGTTTTCGTGTCAGTCCATCATATGTATGCAACAGGATC
CCGTGCCCACACAGGGTGGACTGTTTCATCTCAAGACCCACAGAAAAACTATTTTCCTC
CTGATTATGTACGTGGTGAGCTGTCTCTGCCTCGTGCTAAACGTCTGTGAGATGCTTCAC
TTGGGAATCGGTACTTTCCGGGACACCCTCCGCCTGAAGAGGAACAGGGGCCGACAGTCA
TCCTACGGCTACGCTTTTTCTCGCAATATCCCAGCGTCTCCTCCAGGGTACAACCTTGTG
ATGAAAACAGACAAACCAAGCAGGATTCCCAACAGCCTTATTGCCCATGAGCAGAACGTG
GCCAATGTAGCTCAGGAGCACCAGTGCATCAGCCCAGACGAGAACATCCCCTCTGACCTT
GCGAGCCTACACCGGCACCTAAGAGTTGCTCAAGAACAGCTCGATATGGCTTTTCAGACT
TACCAAACCAAACAAAACCAGCAGACGTCCAGAACCAGTAGTCCAGTGTCTGGAGGCACC
ATGGCAGAACAAAACAGAGTCAATGCAGTTCAAGAGAAGCAGGGCGCAAGGCCAAAATCA
GCCACAGAGAAGGCCACGACCGTGGTAAAAAATGGAAAGAGCTCTGTCTGGATTTAG

>Tn-NN-cx43.4-G02041 Nearly identical to G02430/G02447. Splice site.
ATGAGCTGGAGTTTCCTGACGCGTCTGCTGGACGAGATCTCCAACCACTCCACCTTCGTG
GGGAAGATCTGGCTGACCATTTTGATCATCTTCCGCATCGTGCTGACGGCCGTCGGCGGT
GAGACCATCTACTACGATGAGCAGAGCAAATTTGTTTGCAACACGCAGCAGCCCGGATGC
GAGAACGTGTGCTACGACGCCTTCGCCCCGCTCTCCCACGTACGATTCTGGATCTTCCAG
GTGATCCTGATCACCACCCCCACCATCATGTACCTGGGCTTCGCCATGCACAAGATCGCA
CGCATGAACGACAGCGAGTACCGCGTCGTCCGGAAAGCCAAGAAGAAGATGCCCATAGTG
AACCGCGGACCGCGGGACTACGAGGAGGCGGAGGACAACGGCGAGGAAGACCCCATGATC
GCCGAGGAGATTGAACAAGAGAAGCCTGACAAAGCGGAGAAGGGCCCGGAGAAAAAGCAT
GATGGCCGACGGCGAATCCAGCGTGACGGCCTGATGAAGGTCTACGTGTGCCAGCTGCTG
TGGCGCTCTTCCTTCGAGGTCGCCTTCCTCTTTGGCCAGTACGTCCTCTACGGCTTCGAA
GTGCACGCGTCCTACGTGTGCACCCGCTCGCCGTGCCCCCACACGGTGGACTGCTTCGTG
TCGCGCCCCACAGAGAAGACCATCTTCCTGCTGGTCATGTATGTGGTGTCCTTCCTCTGC
CTGCTCCTCACCCTCTTTGAAATGCTCCACTTGGGGATCGGCGGCGTCCGCGACACCTTC
CGCAGGGCGTCCGCTCTCAACCAGCGGGCCCCTCGTCTGACGGCCCCACGTAGCATCGCC
ACGGCGCCGCCGGGCTACCACGCTACCATGAAGAAGGAGAAATTGAAAGGACGGCTGAGG
GACTCGCCCATGGGCGACTCCGGGAGGGAGAGCTTCGGTGACGAGGGTCCCTCATCCCGG
GAACTGGAGCGGCTGAGGAGGCACCTGAAGCTGGCCCAGCAACACCTGGACCAGGCCTAC
CAGGTTGAGGACAGGAGCCCCTCGCGGAGCAGCAGCCCCGAGGTGAACACGGCCGCGCAG
ACGGCCGCCGAGCAGAACCGACTCAACTTTGCCCAGGAGAAGCAAGGAGAACCCAGCGAG
AAAGGTAAAGAAATGCTCAGGCGCCATCAGATGGAGGTGCGGCGACTATTACAGGTGGTG
GTTTTGGCCAGCAGGCAGCGTTTTCAGAGGCGTCTCTGTCGCTGTTAG

>Tn-cx43.4-G08887 Underlined: Sequence not included in Ensembl transcript prediction, but we consider likely as a part of cds.
ATGAGTTGGAGCTTCCTCACCCGCCTGTTGGACGAGATCTCCAACCACTCCACCTTCGTG
GGCAAAATCTGGCTCACCCTCCTCATCGTCTTCCGCATCGTCCTGACGGCCGTCGGCGGC
GAGTCCATATACTACGATGAACAGAGCAAATTTGTGTGCAACACAAACCAGCCCGGTTGC
GAGAACGTGTGCTACGACGCGTTTGCGCCGCTGTCGCACATCCGCTTCTGGGTGTTCCAG
GTGATCATGATCACCACGCCCACCATCATGTACCTCGGCTTTGCCATGCACAAGATCGCC
CGGATGGACGACAACGACTACCGGCCCCGCGCCAGGAAGAGGATGCCAATCGTCAGCCGC
GGCGCCAACCGGGACTACGAGGAGGCGGAGGACAACGGCGAAGAAGACCCGATGATTCTA
GAAGAGATCGAGCCAGAAAAGGAGAAGGAGACCGCGGAGAAGCCGGGCAAAAAGCACGAC
GGCCGGCGTCGGATCAAGCGCGACGGTCTGATGAAAGTTTACGTGTTCCAGCTGCTGTCG
CGCGCCATCTTTGAAGCCTCCTTCCTGTTCGGGCAGTACATCCTCTACGGGCTGGAGGTG
GCGCCCTCGTACGTTTGCACGCGCTCCCCCTGCCCCCACACGGTGGACTGCTTTGTTTCC
CGTCCCACCGAGAAAACCATCTTCCTGCTCATCATGTACGCCGTCAGCGCGCTCTGCCTG
CTCTTCACCGTGCTGGAGATCCTCCACCTCGGCATCAGCGGCCTCCGGGACTGCTTCTGC
GCCCCGCGGCCCCGCCCGCCCACCCCCCGTCACTCGGCCCTGGCCAGCCAGAGGTCCTCC
ATCTGCCGCCAGCCGTCCGCTCCTCCGGGGTACCACACGGCCCTGAAGAAGGACCCCTTC
GGGAAAGCTGGGCTTCAGGGACAACCTGGGGGACTCCGGCCGGGAATCCTTCGGGGACGA
AGCTTCGTCCCGGGAACTGGAGAGGCTGCGCAAGCACCTGAAACTGGCGCAGCAGCACCT
GGACATGGCCTACCAGAACGAGGAAAGCAGCCCCTCGCGCAGCAGCAGCCCGGAGTCCAA
CGGCACCGCGGCCGAGCAGAACCGACTGAATTTCGCCCAGGAGAAGCAGAGCGACAAAGG
TGA

>Tn-NN-gjd2*2-G11801 Splice site. As predicted by Ensembl.
ATGGGGGAATGGACCATTTTGGAGCGTTTGCTGGAAGCGGCTGTCCAGCAGCACTCCACT
ATGATCGGAAGGATCCTGCTGACAGTGGTGGTGATCTTCCGCATCCTAATAGTGGGCATA
GTGGGTGAGAAGGTGTACGAGGACGAGCAGATCATGTTCATCTGCAACACCATGCAGCCC
GGCTGCAACCAGGCCTGCTACGACAAGGCCTTCCCCATCTCACACATCCGCTACTGGGTT
TTCCAGATCATCTTGGTGTGTACGCCAAGCCTGTGCTTCATCACGTATTCCGTTCACCAG

TCTGCCAAAGCGCGTGACCGAAGCTACTCCCTGCTGCATCCGTACATGGATCACCATGGT
CACGGTCACCACGGTCGCCATCACGACCACCACGCTCGCAAGATCCACTCGCGCTACATA
AATGGTATTCTGGTGCATCCTGAGAGCAGTAAGGAAGACCACGACTGCCTGGAGGTCAAG
GAAATCCCCAATGGACCCCGGGGACTCCCTCCGACACACAAGAGTGCCAAAGTCCGGCGG
CAGGAAGGTATTTCCCGTTTCTACGTCATCCAGGTGGTGTTCCGCAATGCGCTGGAGATA
GGCTTCTTGGCAGGCCAATACTTTCTGTATGGCTTCAACGTTCCAGGGATGTTTGAGTGC
GATCGCTACCCCTGTGTGAAGGAGGTCGAGTGTTACGTATCCCGTCCCACAGAAAAGACT
GTGTTTCTGGTCTTTATGTTTGCCGTCAGTGGCATTTGTGTGCTGCTCAACCTGGCGGAG
CTCAACCACATCGGCTGGAGGAAGATAAAGACGGCCATCCGAGGGGTGCAGGCCCGGAGG
AAGTCCATCTGTGAACTGCGTAAGAAGGATGTGTCTCACCTGTCCCAGGCCCCGAACCTG
GGCAGGACCCAGTCCAGTGAGTCAGCCTACGTCTGA

>Tn-gjd2b-G17236 Splice site. As predicted by Ensembl.
ATGGGGGAATGGACTATACTAGAGAGGCTCCTGGAGGCTGCTGTCCAGCAGCACTCTACT
ATGATAGGAAGGATCCTACTAACCGTGGTGGTCATCTTCCGGATTCTAATCGTGGCGATA
GTTGGAGAGACTGTCTATGATGATGAGCAGACCATGTTTGTTTGTAACACCTTACAGCCG
GGCTGCAACCAGGCGTGCTACGACAAGGCGTTCCCCATCTCGCACATTAGATACTGGGTG
TTTCAGATCATCATGGTGTGCACGCCGAGCCTGTGCTTCATCACCTACTCGGTGCACCAG
TCGGCCAAGCAGAAGGAGCGGCGCTACTCCACCGTCTATCTGACCCTCGATAAGGATCAA
GATTCACTGAAACGCGACGAGAGCAAAAAGATAAAGAACACCATTGTGAACGGAGTACTT
CAGAACACGGAGAACTCCACCAAAGAAGCCGAACCGGACTGTTTGGAGGTGAAAGAGATC
CCCAATTCGGCCATGAGAACTGCAAAGTCCAAAATGAGGCGCCAGGAAGGCATCTCCAGG
TTCTACATCATCCAGGTGGTCTTCAGAAACGCGCTGGAGATCGGCTTCCTGGTGGGCCAG
TACTTTCTGTACGGATTCAACGTGCCGTCGGTGTACGAGTGCGACCGCTACCCCTGCATC
AAAGACGTCGAGTGCTACGTCTCCAGACCCACGGAGAAGACGGTGTTCCTGGTGTTCATG
TTCGCCGTCAGCGGCTTCTGCGTGGTGCTGAACCTGGCCGAGCTCAATCACCTGGGCTGG
AGGAAAATCAAGACGGCCGTGCGGGGCGTGCAGGCCCGGCGGAAGTCCATTTACGAGATC
CGAAACAAGGACCTGCCGAGGATGAGCGTGCCCAACTTCGGACGCACTCAGTCCAGTGAC
TCCGCGTACGTGTAG

>Tn-NN-gjd2-G14329 Corresponds to transcript ENSTNIT00000017564. Splice site.
ATGGGAGAATGGACCATCCTAGAGCGCCTCCTGGAGGCTGCGGTGCAGCAGCATTCTACT
ATGATTGGGAGGATCCTGCTGACAGTGGTGGTGATCTTCCGTATCCTGATCGTGGCGATC
GTTGGGGAGACGGTGTACGAGGATGAGCAGACCATGTTCATCTGCAACACTCTGCAACCA
GGCTGCAACCAGGCCTGCTACGACAAGGCCTTCCCCATCTCCCACATCCGCTACTGGGTC
TTCCAGATCATCCTGGTGTGCACTCCCAGTCTCTGCTTTATCACTTACTCCGTCCACCAG
TCAGCTAAGCAAAAGGACCGTCGCTACTCCTTCCTCTATCCCATTATGGAAAGGGACTAC
GGGGGAAGGGACGGGACACGAAAGCTCCGCAACATCAATGGAATTCTAGTCCAACATGGC
GGCGATGGCGGAGGAGGAAAGGAAGAACCAGACTGCCTGGAGGTGAAGGAGATCCCCAAC
GCCCCGCGGGGCCTCACTCATGGCAAGAGCTCCAAGGTTCGCCGCCAGGAAGGCATCTCC
CGCTTCTACGTCATTCAAGTGGTCTTCCGAAACGCCCTGGAGATCGGATTCTTGGCAGGC
CAGTACTTCCTCTACGGCTTCAGCGTGCCTGGGATTTTCGAGTGCGACCGCTACCCGTGT
CTGAAGGAGGTGGAGTGCTACGTGTCCCGGCCCACCGAGAAGACGGTGTTCCTGGTGTTC
ATGTTTGCGGTGAGCGGCATCTGCGTGGTGCTAAACCTGGCTGAGCTCAACCATCTGGGG
TGGCGCAAGATCAAGGCGGCCATCAGGGGTGTCCAGGCCCGCAGGAAGTCCATCTGCGAG
ATCCGGAAGAAGGACATGGCGCACCTCTCCCAGCCGCCCAACCTGGGCCGTACGCAGTCC
AGCGAGTCGGCCTACGTGTGA

>Tn-cx36.7-G03401 Underlined: Sequence not included in Ensembl transcript prediction, but we consider likely as a part of cds
ATGACGGAATGGACCCTGCTGAAACGCCTCCTGGATGCCGTCCATCAGCACTCCACCATG
ATTGGCCGCCTGTGGCTGACTGTGATGGTCATCTTTAGGCTGCTGGTAGTTGCTGTGGCA
ACGGAGGACGTGTACACGGACGAACAGGAGATGTTTGTGTGCAACACGCTGCAGCCGGGG
TGCTCGACCGTCTGCTACGACGCCTTTGCGCCCATCTCGCAGCCGCGCTTCTGGGTGTTC
CACATCATCAGCGTGTCCACGCCGTCGCTCTGCTTCATCATCTACACGTGGCACAACCTG
TCCAAGGTCCACAGCGCTGCTCAAAGGCACCCACGCGGCGCTGGCCAGCCGCTCGGCCAA
GACGTCGGCCAAGCGGCAAAGGAGGTCCTGGGAAAGGAGGCGGGGAAGGCAGGTGGCGAC
CGGGAGGTGTACCCTCCAAGCTGCAGCTCGGACAGCTGCTCCGTCCTCTCCCACAAGCAC
CTCGGCCACAGCCTGGTGGACATTTTAGACGGCGTCGCCGCCCGTAGTTTGCGAAACGGA
GACCCGGCGACCTCCAGGCCCATCCAAGCTTACACCTTTAAAGACGGAAGCTCGGAGGGT
CTGGCGGTCTCTGGAGGAGTTCTGTCCAAATGCTACATCTTCCACGTGTGTCTACGCGCA
GCTCTGGAGGTGGGATTTGTCGCCGCCCAGTGGAAACTGTTCGGATTGCAGGTGCCTGTC
CGCTTTTTGTGTACCTCCTCGCCCTGCAACCAGCCCGTGGACTGCTACGTCTCCAGGCCC
ACGGAGAAGACCATATTCCTGATCTTCATGTTTTGTGTTGGCGTCTTCTGTATCTTCCTC
AACCTGCTGGAACTCAATCACCTGGGCTGGAAGAAGATCAGGCAGGCGGTGCGGCTGAAG
GAGGACGAGGCGCCCTGGCAGGCCTGCGCGGGGATAGGACGCGGATACCAGACCATCCCT
CCGGTCAGCCCTTCGCCCAAGTCTTCGGGTATGAACGGCACCGCCCCTCCGCCCACTTTG
GACGTGGCGATGGGCCACAAACCGGAGTGGGGCTGCGTGGTCAACTGTGGCGGCACCCGA

GGGTGCGGAAAGGTCAAAGGAGAGAGGAAACACAAGGAGCTGAGAGGGTTCAAGCAGAGC
AGCGCAGAAGTCTGGATCTGA

>Tn-GJD3-G12849 Underlined: Sequence not included in Ensembl transcript prediction, but we consider likely as a part of cds.

ATGGGGAGTGGGGCTTCCTCGGTGGACTCTTCGACAGCCTCCAGGCTCACTCGCCCATG
CTGGGTCGGTTCTGGCTCCTGCTCATGCTCATCTTTCGGATAGTGATCCTCGGAACTGTG
GCCAGCGACCTGTTTGAGGACGAGCAGGAGGAGTTTGCCTGCAACACTCTCCAGCCGGGC
TGCAAGCAGGTGTGTTACGACATGGCCTTCCCCATCTCGCAGTACAGATTCTGGGTCTTT
CACATCGTGCTCATCGCCACGCCTTCGCTACTCTTCCTCGTTTACACCATGCATCATCAC
AACAAGAGCAACTGCAAATTCAATCCCAGGTACAGGGAAGACGTGCGTTTGAGGAGGCTC
TATATTCTCAACGTGGTGTTTCGGATTCTGGCCGAGGCCGCCTTCCTGGTGGGCCAGTGG
CTGCTCTATGGCTTCAAGGTGGAGGCCCAGTTCCCCTGCAGCCGCTTCCCCTGCCCCTAC
ACCGTGGACTGCTTCACCTCGCGTCCTGCAGAGAAGACCATCTTCCTCTGCTTCTACTTC
GTCATCGGGGCCATAGCCGCCCTCTTCAGCTGTGCGGAGCTCTTCCACATCTCTGTGAAG
TGGTTCTGCGCCGGCCCGGAGCCCTCGAAGACAGAGGACTCGGGCATCAGCGATAACCTT
CTCAACGTGAAGCAGGAGGAAGGGATCAAAAAGGAGAAGCAGCAGGAGAAGAAGCAGCAA
GCTCCCGACAGCAGAAGGCTGAAGAGAGGATCGGTGAGGAGCAGCTCCAGCAGGAAGAGC
TCCGGCGGCCTCCACAGGCACATCAGTGGCAAATACGTGAGCAGCAGGACTTTGATGGTG
TGA

>Tn-NN-cx39.2-G01238 Underlined: Sequence not included in Ensembl transcript prediction, but we consider likely as a part of cds.

ATGGGAGACTGGTCCATTCTTGGCCGCTTCTTAACCGAAGTTCAAAATCATTCCACGGTC
ATTGGCAAGATATGGCTGACCATGCTGCTCATCTTCCGCATCTTGCTGGTAGCACTGGTG
GGCGACGCGGTGTACAGTGACGAGCAGTCTAAGTTTACCTGCAACACCCTCCAGCCTGGA
TGTAACAACGTCTGCTATGACACCTTTGCTCCCGTCTCGCACTTGCGCTTCTGGGTCTTT
CAGATTGTTCTTGTCTCCACACCTTCTATTTTCTACATCGTCTACGTCTTACAAAAGATC
ACCAAGAATGAAAAGTTAGAGGTGAAAAAGGTTGTAGTGATACCACGGTCTCCTACACCA
TTCAAAGGGGGGGAGGATCGAGGAGGAGATAAAGAGGCAATGCTGGAGACTGGTGGCCCT
TATAACCCAACCTATAACAATGAGGAGTGGAGCTCTCAGGAGGATGAGTGTGAGGAGAGG
AGCCAGCTGAATGAGGAAATGAAAGAGGTCGGAAAAGACCCGACCCAGCTCTCCAGTCAA
GTGTTACTCATTTACATCATCCATGTTCTGCTGCGCTCCATCATGGAGATCATCTTCCTC
ATTGGACAGTATTACCTCTTTGGATTTGAGGTGCCACATCTTTTCCGCTGCGACACCTAC
CCGTGTCCAAACAGAACCGACTGCTTTGTCTCTCGAGCCACGGAGAAGACCATCTTCCTG
AACTTCATGTTTAGCGTCAGTCTTGGGTGCTTCATCTTGAACATCGTGGAGCTGCATTAT
CTCGGCTGGATTTATATTTTCAGAGTGCTGCTCTCTGCATGCTGCACGTGCTGCAAGTCC
AACAGAGACCCGGTTCAGCAGGTGGAGTTGTATTCGGACAACAACCCACTGCTGCTGGAG
CTCAAGCATTCACTGCGGGGCAGGGTCGTCCTGCAGGCCACCTCTGCCGTGACACGGGAC
AAAAGCAGCAGCGTCCCAAATCAGGCCCCAGCTATCTCTTTTGAAACAGACTCCACACTG
GAGTGCACTTCGAAGCGGAACCCAGATGAAAAGGAACGCACTAAGGCAAGACTGCACAAA
ATCGGAAGAGGCAAAAAGTCATGGCTGTAA

>Tn-NN-gjd4-G07977 Ensembl prediction as pseudogene. Underlined: Sequence not included in Ensembl transcript prediction, but we consider likely as a part of cds. Exon 1 is missing.

GGTAAGACCTGGTGGACTCTGCTGCTGGGTTTGCGCCTGAGCGTCCTGCTGCTGCTGGGC
TTCAGCCTCTTCAGCGACGAGCAGGAGCGCTTCGTCTGCAACACCATCCAGCCGGGCTGC
TCCAACGTGTGCTTCGACGCCTTCGCTCCCGTGTCCGTCTTCCGCCTCTGGCTCCTCCAC
CTCGTCCTCCTGGCTCTTCCCCATCTGCTCTTTGCCACCTACGTGATGCACCGGCTTCTG
ACCGCTCCGGGTTCCCTCTGGCTCGTCCCAGGGAACTGTCCCTTGCGCGGGAGCCAGCTC
CAGGAGCCCGGAGGAGCGCGCTTCTACTGCGCGTACGTCCCGGTGGTGGTGGTCCGGATC
CTTCTGGAAGTTGTTTTCGGGGCCGGCCAGTTTCACCTCTTCGGTTTGTCCTTTCCAAAG
AGCTTCCTGTGCTACGAGGCCCCCTGCACCTCGGGGGTGGAGTGCTACATCTCCAGACGC
ACCGAGAAGTCCCTCATGCTCAGTTTCATGTTGGGCGTGGCCTCGCTCTCCATCCTGCTG
AGTTTGTTTGATCTGCTGGGCTCCGTGAAGGCGATGGTGAGCTGGAGGAGGAGGAGGGAG
ATGCTGGCGGAGGAGATGATCAAAGGAGAACAAAGCAGCGTGATTACGGCGACGACCATG
GCTGAAGACAGCGATAAAAGCCCCGAGTCCAACAGTCCCGACAGCAGAGATGCTCAGGTG
GACACACCTCCCACTCCCACCAGCACTCCAGCACCTCCTCGGTCGGTCCTCCACAGCCGG
GTCGGACCCCCGCTGTCCCCTCGGCCTGACAGGGAACCACTGAGGGACCCAGCACCAGTG
GGGGGGAGGAAGCCGGCCCAGTACGGTCCAGCCGGGACAACCTCGGGCCAACAGTCTGAC
GGCGAGGCTCCAGACAGACGAGCCTGGGTTTGA

>Tn-GJD4-G08724 Ensembl predicts another exon 1 and another splice site. Underlined: Sequence not included in Ensembl transcript prediction, but we consider likely as a part of cds. Splice site.

ATGGCGGGATCAAGTACCTGTGAGGTCATCTTCATCTCTGTCAATCACAGCATCCCGCTG
ATGGGGAAAGTGTGGCTCATAGTGATGATCTTTCTCCGTATCCTGACCCTCCTTTTTGCC
GGATACCCCCTCTACCAGGACGAGCAGGACCGATTCGTGTGCAACACCATCCAGCCTGGA
TGTGCCAACGTCTGCTACGACCTGTACTCCCCCGTCTCCCTCTTCCGCTTCTGGCTGGTC
CAACTCATCACTTTGTGTCTTCCCTACATCGTCTTTGTCATCTACATCATCCACAAGGTC
TCAAATGACCTCTGCGCACACCTGAACTCCTCGGGCCAGGTCAGAACCTCGCGGCTGTTC
CAGATCCAGCAAGAGGCACCTGGTGAGAAGATGGCGCCTGAGAGGGGATCGGCTCGGTGC
TTCACAGGAGCCTATATCCTCCACCTGATGTTCCGAACCTTGCTGGAGGCAGGATTTGGA
GCTGCTCATTACTATCTCTTCGGTTTCAACATCCCCCGGAGGTTCCTGTGTCAACACCCA
CCGTGCACCACCCAGGTGGACTGCTACGTGTCCAGACCCACCGAGAAGACTGTGATGCTC
AGCTTCATGCTCGGCGTGGCCGTCCTGTCCCTTTTTTTAAACGTTTTGGATTTTATTAGC
GCCATCAAGCGCTCTGTCACCAAGAAGGGCAAAAAGAAGTTGATGGTAGGGAAGATTTAT
GAGGAGGAGCAGTGCTTCCTGTCAACGGGTGCGGCCTCCGGACCAACAGACCCAAACCAC
TCGGTGGGTAAACAGAATCTAGAGGTGGAGGCTCAGGCGGGCGGTTTCCGGAAGAGGCAC
AACAGCAAGGGTTCTTGCGCAGGGGTTGCTGTCCCTGTAGGGCAAGATCCACCCTCTCTC
GACCGTTCTTCATCCTTTCCACGTTCACTTGGACCTCCAGGGTCCAACACAAATGGGAAC
AATGGCTACTCCCTTCCACAGGAGGATGTTTCAGAAAACAATGGCAGCGACGTGGCCCTC
TGCCCCCCAGAGTCCATGGGGACACCTAGATCCATTCGAGTTAGCAAACGAGGTCGATTA
AAACCTCCTCCTCCACCTAGGCGAGATCTGGGTTCGTCTCCAAGTGGGCCGGCGGGGCCC
CCTGGGGACATTTCAGCAATCTGTACCAGAAGGGTCGGCCAATTCACACTGGTAGAGCTG
TCCAACGCAGAGCTACGGACCAGTGAAGACGGGCAAGACAAAAGGTCAGAGTGGGTCTGA

Suppl. Fig. 8. Three-spined stickleback (*Gasterosteus aculeatus*) connexins.
Stickleback, *Gasterosteus aculeatus* (Ga)
Assembly: BROAD S1, Feb 2006.
Genebuild last updated: May 2010.
Database version: 98.1.

As far as possible, the names of the sequences are taken from the Ensembl predictions. Where there is a prediction (although we might have modified it) without a name, we include NN (no name) as a prescript, use the most common name of the ortholog sequence (usually from zebrafish), and end the name with an abbreviated Ensembl gene prediction number. Where there is no prediction in Ensembl and no predicted (or experimentally found) sequences in GenBank with a name, we include NP (not predicted) as a prescript, and use the most common name of the ortholog sequence (usually from zebrafish).
The Ensembl gene number abbreviation is done as follows: ENSGACG00000004089 = G04089.

Yellow: Conserved domains as defined by Cruciani and Mikalsen (2007)
Green: Conserved cysteine codons (cysteine signature)
Grey: 15 nt added at the ends of the conserved domains
Turquoise: Splice site.
Other colors are explained where necessary.

>Ga-cx43-G04089 As predicted by Ensembl, but 1 A exchanged with N to avoid unexpected stop codon)
ATGGGGGACTGGAGCGCTCTGGGCCGTCTCCTGGACAAGGTCCAGGCCTACTCCACCGCC
GGCGGCAAAGTCTGGCTGTCGGTCCTCTTCATCTTCCGCATCCTGGTCCTGGGCACGGCG
GTGGAATCCGCCTGGGGAGACGAGCAGTCGGCCTTCAAATGCAACACCCAGCAGCCCGGT
TGTGAGAACGTGTGCTACGACAAATCCTTCCCCATCTCCCACGTCCGCTTCTGGGTCCTC
CAGATCATCTTCGTGTCCACGCCCACGCTCCTCTACCTGGCTCACGTCTTCTACTTGAAC
CGGAAGGAGCAGAAGTTCAACAGGAAGGAGGAGGAGCTCAAGGCCGTGCAAAACGATGGC
GGCGACGTTGACATCCCGCTGAAGAAGATCGAGATGAAGAAGCTGAAGTACGGCATCGAG
GAGCACGGCAAGGTGAAGATGAAAGGGGCCCTGCTCAGAACCTACATAGTCAGCATTTTC
TTCAAGTCCATGTTCGAGGTGGGCTTCCTGGTGATCCAGTGGTACATCTACGGGTTCAGC
CTCTCTGCGGTCTACACCTGCGAGAGGGACCCGTGCCCACACCGGGTAGACTGCTTCCTG
TCGCGTCCCACGGAGAAGACGGTGTTCATCATCTTCATGCTGGTGGTGTCCCTGGTGTCC
CTGATGCTCAACCTCATTGAGCTTTNATACGTCTTTTCAAGAATATCAAAGATCGCGTGA
AGNNNNN

>Ga-GJA3-G01367 Our modification. Underlined: Introns predicted by Ensembl are included as part of exon.
ATGGGCGACTGGAGCTCTCTGGGCCGCCTGCTGGAGAACGCTCAGGAGCACTCGACGGTG
GTCGGCAAGGTGTGGCTGACGGTCCTCTTCATCTTCAGGATCCTGGTGCTGGGCGCGGCG
GCCGAGGAGGTGTGGGGCGACGAGCAGTCCGACTTCACCTGCAACACGCAGCAGCCCGGC
TGCGAGAACGTCTGCTACGACGAGGCCTTCCCCATCTCGCACATCCGCTTCTGGGTGCTG
CAGATCATCTTCGTGTCCACGCCCACCCTCATCTACCTGGGCCACGTGCTGCACATCGTC
CGCATGGAGGAGAAGCGCAAGGAGAAGGAGGAGGACGCGCGCAAGGCCAGCAGGATCCGA
GAGGAGAAAGAACTCCTTTGTAGGAACGGTGCGGACTCGGGAGGAGGCGGGGGACGGGGC
GCCAAGAAGGAGAAGCCGCCAATCAGGGACGAGCACGGCAAGATCCGCATCCGGGGCGCG
TTGCTGCGCACCTACGTGTTGAACATCATCTTCAAGACCCTGTTTGAGGTGGGCTTCATC
CTGGGGCAGTACTTCCTGTACGGCTTCCAGCTCAGGCCGCTGTACAAGTGCGCCCGCTGG
CCGTGCCCCAACGCGGTGGACTGCTTCATATCCCGGCCCACCGAGAAGACCATCTTCATC
GTGTTCATGCTGGTGGTGGCCTGCGTGTCTCTCCTGCTGAACTTGTTGGAGATCTATCAC
CTGGGCTGGAAGAAGGTCAAGCAGGGCATGAGCAGCGAGTCCCCGCCCCTCCACGAGTCG
CCGCGCCGCGTCAACCTCGCGCAGCCCGAGTGCTCCCGGACTGCCCCCCGCGGCCTCGGC
CGCCCCCCCGACTACACGGACGTGACGGCGGGCAGCGCCGCCTTCCTGCCGCCCGCGGGC
CTGGCGGCGGCGGCGGAGTTCAAGGCGGGCGGACTCCGGCGGGAGGAGCCGCTCCGCCGC
CCCCCCACCTCCGCCCACTACTACGTCAGCAGCAACAACAACAACCACCACCGGCTGGCC
ACGCAGCAGAACTGGGCCAACCTGGCCACCGAGCAGCAGACCCGGGAGATGAAGGCCGCC
GCCGCCGCCGCCCCCTCCTCCTCGTCCTCCAGCAGCAGCGGCAAAGAGCGGCAGCAGCGG
CCCGTGGATGCCGCGGCGCCCCCCCCCAGCAGCAACGTCTGCAGCAACGTCGACTCCACC
GCCGCCGCCTCCAGCAGCAGCAGCAGCAGCAGCATCGCGTCCAGCGCGGGCAGCTGGAGG

GGGGGCAAGAGCGGGCAGGAGGAAGGTCACGCCACCAACACCCTCCACCACCACCGTGGA
GATGCACGAGCCCCCGCTGACCGACCATCGGCGGCTCAGCCGGGCCAGCAGACCAGCAGC
GTCAGGGCGAGGCCGAGCGACCTGGCCGTCTGA

>Ga-NN-gja3-G14074(2) Our modification. There are two connexins (wrongly) fused into one Ensembl prediction. The other sequence is Ga-NN-cx30.3-G14074(1). Underlined: Exons predicted by us. Splice site.
ATGGGTGACTGGAGCTTTCTTGGGCGGCTGCTGGAGAACGCTCAGGAACACTCCACTGTG
ATTGGAAA**GG**TGTGGCTGACCGTTCTCTTCATCTTCCGCATCTTGGTGCTTGGCGCAGCG
GCCGAAGAGGTTTGGGGCGACGAGCAGTCCGACTTCACCTGTAACACCCAGCAACCCGGT
TGCGAGAACGTCTGCTACGACGAGGCCTTCCCCATCTCCCACATCCGCTTTTGGGTGCTG
CAGATCATCTTTGTCTCCACGCCGACCCTCATCTACCTGGGCCATGTGCTGCACATCGTC
CGCATGGAGGAGAAGCGGAGGGAGAGGGAGGAGGAGCTCCGGAAGGCCGGGCGGCACCAG
GAGGACCACGATCCGCTCTATCACCTCGGAGCCGCTGATGGGGGAGGAAAGAAAGAGAAG
CCGCCAATCCGCGATGAGCACGGGAAGATCCGCATTCGCGGGCACTGCTGAGGACCTAC
ATCTTCAACATCATCTTCAAGACTCTGTTTGAGGTTGGCTTCATCCTGGGACAGTACTTC
CTGTACGGCTTCCGCCTGAGGCCGCTCTACAAGTGTGGCCGCTGGCCCTGCCCCAACACC
GTGGACTGCTTCATCTCCA**GG**CCCACTGAAAAGACAATCTTCATCATCTTCATGCTGGTG
GTGGCCTGTGTCTCCCTGCTCCTCAACCTGCTGGAGATCTACCACCTGGGCTGGAAGAAG
GTCAAGCAGGGGGTCTCCAACGAGTTCGCCCCCGGCGGGGAGTCTCCGGCGCTGATCGGG
GACGAGCCCGGGGACCCGGAGACGATCCGCGAGCAGACGTACCCGCGGACGCTCGACTGT
TTGCCGGTGTACGCCACCGTGAACGTGGCAGGGGTCGGGGCTGAAGAGGGAGGAGCCTAC
AGTTCGACCGAAGCCTGCGCACCAGTGGTGCCCGCCAGATTCAAGATGGACGCCGCGTTG
TTCCACCCAGACGACTTCCTGTTGGACTCGCTGCCTACGTCTTTTCACGCCGGGAAAGGG
AGTGACGGGTGCCGCGAGCAGCGGATGGAGACGGAGCAGAACTGGAGCAACATGTCGCTG
GAGCTCCACAATCGGGAAGGGAAGGAATCCTCCTCCTCCGCCTCCTACCCGTCTCCCTCT
CCTCCCACATCCGCCTCTTCCTCTCCCCGAGAGGATACCGCCCCGCCACTTCCACACGGG
GAGCAACACTCCACGTTTCCTACACTTCCGCGTCACACCCCCCTGTCTCCGCTCGCACCT
GAAGAGGCGGCGGCGGACGAGGACACGTCCCCTCATACGGCCCTCCACGACAACTTCACC
GTGGTTATCAAGGCGGAGATGCATCCGCCTCCCACTTCTGCCACGAGAGACGTCCCAAAG
CCCAGTCGGTCCGGCAAGAGCGGCGGTGTCCGAGCTCGCCCCGACGACCTGGCAGTGTAG

>Ga-NN-cx39.9-G20329 Our modification. Underlined: Intron predicted by Ensembl is included as part of exon.
ATGGGGGACTGGAATTCGCTGGGGAAGCTGCTGGAGAGCGCCCAGGAGCACTCAACCGTT
GTGGGCAAAGTTTGGCTGACAGTCTTGTTCATTTTCCGCATCCTGGTGCTGGGATCCGCC
GCTGAGAAGGTTTGGGGCGACGAGCAGTCGGGCTTCACCTGCGACACCAAGCAGCCCGGT
TGTCAGAACGTCTGCTACGACAAGACCTTCCCCATTTCCCATATCCGCTTCTGGGTGTTG
CAGATCATCTTTGTCTCCACGCCAACGCTCATTTATCTGGGCCACATCCTTCACCTGGTC
CGCATGGAGGAAAAGGAGGTGCAGAAAGAAAAGGACCTCGCCACCGACGAGGAAATGCAC
GAGCAGTTACACGCGACCAAAGCCAAGAAGGCCTCGGTCAAAGACAAACAGGGCCACGTG
CGCTTAAAAGGGGCACTTTTGCGAACCTACGTCTTCAACGTCATTTTTAAGACCCTGTTT
GAGGTGGCTTTTATCGTCGCCCAGTACTTCCTGTACGGTTTTGAGCTAAAGCCCATGTAC
ACCTGCGACCGCTGGCCTTGCCCTAACATGGTGAACTGCTACATCTCTCGACCCACCGAG
AAGACCATATTCATCCTGTTCATGCTGGCCGTGGCCTGCGTCTCCTTGCTGCTCAACCTG
GTGGAAATGTACCATCTAGGCTTCACAAAGTGCCACCAGGGCCTCAGTTACAGACGGGCG
CGGGCTGCTCGCGAGGCTCCGAAGGCCTTAAACGAGGCTGTCGTGCCGTACGTCACCGAC
TACAGCTTCTTTTCGGGTCACGCCGCGGTGCCTAGTCCTTTCCCCGTGGACTCAAAGTAC
AGCGCGGCAGCGCCCAACGCCGCCTACAGCCCCTACAACAGCAAAGCGGTTCACAAGCAG
AACAGAGACAACATGGCCGTGGAGAGAAAAGGCAAACCAGAGGGAGACGAGGCGAAGGAG
AGCAAAATCTCAGGCCCCGTTTCTGAGTTGCCCGGCGAACATCAGCGCAGAAACAGTCAG
TCAAGCAAACACAGCAACAACAAGAGCAGGCTGGATGACCTGAAGATCTAG

>Ga-NP-cx39.9 This sequence is predicted by Ensembl as a part of an intron in vma21 (vacuolar H+-ATPAse homolog) (G18298).
ATGGGCGACTGGAACCTGCTGGGAAAGCTTCTGGAAAAGGCCCAGGAGCACTCCACCGTG
GTGGGGAAGGTGTGGCTCACCGTGCTGTTCATCTTCCGTATCCTGGTCCTCAGTGCCGCC
ACAGAGAAGGTGTGGGGCGACGAGCTGTCGGGCTTCACCTGCGACACGAAGCAGCCGGGC
TGTGAGAACGTGTGCTACGACGTCACTTTCCCCATCTCTCACGTCCGGTTTTGGGTGCTG
CAGATCATCTTCGTGTCCACGCCGACGCTGATCTACCTGGGGCACATCCTGCACCTGGTG
CGGATGGAAGAAAAGGACCAGCAGAAAGAGCTCGCTCAGCATTCGGACAAGCAGGCCCTT
GTTGCGGATGGTAAGCAGAAGAAAGCTCTGGTGAGGGACAATAAGGGTCGAGTGCGCCTG
CAGGGGGAGCTCTTGCGTACATATGTGTTTAATGTGGTCTTCAAAACCCTGTTTGAAGTG
GGTTTCATCGTGGCCCAGTACCTCTTGTACGGCTTCGAGCTGAAGCCCATGTACACGTGT
GACAGACCGCCCTGCCCCAATGTGGTCAACTGCTACATTTCACGTCCCACGGAGAAAACC
ATCTTCATCATCTTCATGCTGGGAGTGGCTAGCGTGTCTCTGCTCCTCAACCTCGTAGAG
ATCTACCACCTGGGCTTCACCAAGTGTCGCCAGGGCATCACCTTCAGGCGACGCCATCGG
TTCTCCAGGGGGCTCCCCAAGGAGCCCAGCGGGGCCGCGGTGCCGTACGCGCCGAGTTAC

GACGACTACTTCCACCAAGTCCAGCCGGCCTACCCGCCCGTACCCAGCTACGACCTCCAC
CCTCTGTCCGAGGGCACCGACCCGCCCTTCCACCCCTACCACAGCAAGGCGGCCTACAAG
CAGAACTCTGACAACTTGGCGGTGGAAAGGAGCGGTGGCAAACCAGAGGAAAGTGACCCA
AAGGGTAAAAAGGGAGCCGGGTTGGCCCCCGGGTCGCCCCCCGGGTCGGCCCCCGGGTCC
CCTACGCAGGCCAGGCCAGGCCGCAGCGGCAAACACAGCAACAACAAGACTAGAATAGAC
GATCTTCAGATATGA

>Ga-cx39.4-G07433 Our modification. Underlined: Exon extended in both direction until initiation and stop codon.

ATGTCCAAAGCTGACTGGTCCTACCTACAGCACCTGCTGGAGGAGGGCCAGGAGTATTCG
ACGGGCATCAGCCGCGTCTGGCTTACCGTGCTCTTCCTGTTTCGCATGCTGGTCCTGGGC
ACCGCCGCTGAATCCGCCTGGGACGACGAGCAAGCCGACTTTGTCTGCAACACGCTGCAA
CCCGGCTGCACAGCTGTGTGCTACGACAGGGCCTTCCCCATCTCCCACTTTCGCTACTTT
GTCCTCCAAGTCATCTTCGTCTCCACGCCGACCATCTTCTACTTTGGATATGTGGCCATA
ATGGCCGGGAAAGACAAGCAGAAAGAAGAAGAAGATGGGAAGGAGGCGGAGGAAGGCGGT
GGTGGAGGTAGAAGTGGAGGGAGAGCATCAGAAAGGGACGATGACAATGTGACCAGAGAC
AATGCGCCAGAGAAGGAGAAACTAGGGGGCGGTGGCAGAGGTAGGCGAGCTGAGAGGGAC
CCACCTGCCGCTCCTAAACTGAAAGGAAGGTTGCTGTGCGCATATGTGTTCAGCATCCTG
TCCAAAGTGCTCCTGGAGGTCGGCTTCATCGTAGGGTTGTGGTTCCTCTACGATGGCTTC
TACATTGCAGCGAAGTTTGAGTGCACCTGGTCCCCCTGTCCCCACACCGTGGACTGCTTT
GTGTCCAGGCCCACGGAGAAGACCATCTTCACCATCTACACCCAGGTGATTGCTGGCATC
TCCCTGCTCCTCAACCTCGCCGAGCTCCTTCAGCTCGCCGTCTCCCACCGGCTGGCGAAG
TACTACCGTACCCAGACCCAGGACCACCTTCCTCGATCCAAGCAGGTACCGGCTAGACAG
GAGGCGGCTTCCGAACCTCCGCCGGATTCATCCAGGCCTTACAATGCAGGGGGTCATGTC
AACCCCCCCGGGCCGGGGGAGGCTCCATGCTACGCCACACCTTGTGAGAGCTACGGGGAC
CTGGGGATCGAGGTCGGCTGGGGTCCCAGGGAGGTCGGGAGTGACCTGCTTCCCAGTTAT
GTGAACTGCATAGGGGCTATGAAGACCCACTGCCCAAAAGTCCATTATAAGGCACACCCA
AAGCTCCCTGGGAAAAAGACTAAGGGTGTCCATAAGGAACACTCGGGGAAGAAGCATTAC
GTATGA

>Ga-GJA5-G03669 Our modification. Underlined: Introns predicted by Ensembl are included as part of exon.

ATGGGGGATTGGAGTCTCCTGGGGAATTTCCTAGAGGAGGTCCAGGAACACTCTACCTCG
GTCGGGAAGGTCTGGCTCACCGTCCTCTTCATCTTCCGCATCCTGGTGCTGGGCACGGCG
GCCGAGTCGTCCTGGGGCGACGAGCAGAGCGATTTCCTGTGCGACACCCAGCAGCCCGGC
TGCACCAACGTGTGCTACGACAGCGCCTTCCCCATCGCCCACATCCGCTACTGGGTGCTG
CAGATTGTTTTTGTCTCCACGCCGTCCCTCATCTACATGGGCCACGCCATGCACATTGTG
CGCCGGGAGGAGAAGCAGCGGAGGGTGGAGCAGGAGGAGAGGGAGGAGAGGGGGGAAGGG
GGAGAAGACCTGGGGGGGGAGAAGGAGTACCTCCAGCAGAAGGTGAGCGGGAGAATGGTG
GCGTCTGACGGGACCGGCCGTGTTCGCCTGAAAGGGGCGCTGCTGCAGACGTACATCCTG
AGCATCATGATCCGCACGGTGATGGAGGTGACATTTGTCGTGGTGCAGTACATGATCTAC
GGGGTGTTCCTCAGGGCGTTGTACCTGTGCAAGTCTTGGCCCTGCCCCAACCCCGTCAAC
TGCTACATGTCCCGGCCCACGGAGAAGAATGTCTTCATCGTCTTTATGCTGGTGGTGGCC
GGCGTGTCCCTGCTGCTCTCCGTGCTGGAGCTCTACCACCTCAGCTGGAAGGGCGCCAGG
AGGTGTTTACGCAAGAAGAGGATGGAGAAGAGCAGCCACAAAGCTGTGACGGCGGCCGTC
TCCGCGGCCTTGGAGCCCAACAGCCCCCCACTGCCCCCGGCCTCCTGCACCCCGCCTCCT
GACTTCAGCCAATGCCTGGCGGCCTCGAGCTCCATGGACCCCATGACCTCTATGGCCTCG
CACCCCTTCAGCAACAGGATGGCGCTGCAACAAAACTCCGCCAACCTGGCCACGGAGCGG
CACCACAGCTGCGACGACCTGGAGGATGAGAAAGACTTCCAGAGGATGCGATTCGACCAG
GCGCCCCCAGAGGTGCCCACCAGCTGCTCTCCCTCGCCGCTGCTGCACTCCGGCTACACG
AAGGACAAACGCCGCCTGAGCAAGACCAGCGGCACCAGCAGCCGGGCTCGGCAGGACGAC
CTGGCAGTGTAG

>Ga-NN-gja5-G11699 Our modified prediction, including the removal of two nt in first conserved domain (between lower case letters). Underlined: Introns predicted by Ensembl are included as part of exon. Note that also a part of an Ensembl predicted exon is considered as intron by us (at splice site 2). Splice sites.

ATGGCGGACTGGAGCCTGCTGGGAAACTTCCTGGAGGAAGTGCAGGAGCACTCGACCTCC
GTTGGCAAGGTGTGGCTGACCGTCCTCTTCATCTTCCGCATCCTGGTGCTCGGGACGGCC
GCCGAGTCTTCCTGGGGAGACGAGCAGGAGGACTTCAACTGCGACACCGAGCAGCCGGgc
TGCGAGAACGTTTGCTACGACCGAGCTTTTCTCATCGCCCACATACGATACTGGGTGCTG
CAGATcgTGTTCGTGTCCACTCCCAGCCTCATCTACATGGGCCACGCCATGCACACCGTC
CGCATGGAGGAGAAGAGGAGGAGCCGGGAGGAGGAGGACGGGGACGGGGGGGAGAGGCAG
GAGGACCCGGGAGGGGGCGGAGGGGATGGAGGAGGAGAGAAACACGGGAGGAAAGGAGAG
AAGGACAGAGGAAAGGAGGAAAGCAGAGACGGTCAGGCGGCAGGTCGAGTGCGTCTGAGG
GGCGCGCTGCTGCAGACGTACGTCCTGAGCATCCTGCTGCGGAGCGTCATGGAGGTGGTG
TTCCTGCTCCTCCAGTACTTCATGTACGGCGTCTTCCTCAACCCTCTGTATGTCTGCAAG
GCCTGGCCGTGTCCTCATCCAGTGAACTGTTACGTCTCCAGGCCGACGGAGAAGAACGTC

TTCATAGTGTTCATGATGACCGTGTCCGCCGTCTCCCTGCTCCTCAGCGTGCTCGAGCTG
CATCACCTGGCGTGGAGACACTGCTGCAGGTACGCGCGCTCACCAGCGAAGGCCGTCACT
CTAGCCAACGCCTCGCTGGCCCGTCAGCTCTCTGTGCCCCCCCCGCTGCCGCCCACCCCT
CCCCCGGACTTCAACCAGTGCGTGATGGGCTCCTCCCACTTCCTGCCGCTCCCTTTCCCC
AACCACCGCCTCGCCGACCAGCAGAACTCCGACAACATGGCCGCCGAGAAGAACAAAATG
GCCGCTGCCGCCGCAGAGGAGGTGACCCTCCTCCAGATGAGCCGCTACTCACCCGCGTGG
CCCGCGGCGGGCGGCGGTCAGATCCAAGATGGCGGATACCTGAGGACCGGCGACGGGGAG
ACGGGCGGGGGTCACAGGGACCGACGGAGGTTCAGCAGGACGAGCGGAACGAGCAGCCGG
ACCCGAGCCGACGACCTCTCGGTTTAG

>Ga-gja8a-G03667 No modifications.
ATGGGTGACTGGAGCTTTCTGGGTAATATTTTAGAGGAAGTTAACGAGCACTCTACGGTG
ATCGGCCGGGTGTGGCTCACGGTGCTCTTCATCTTCCGTATCCTCATCCTGGGCACGGCG
GCGGAGTTTGTGTGGGGCGATGAGCAGTCTGACTATGTCTGCAACACGCAGCAACCGGGA
TGTGAAAACGTGTGCTACGACGAGGCCTTCCCCATCTCCCACATCCGCCTGTGGGTGCTG
CAGATCATCTTCGTGTCCACGCCGTCTCTGGTGTACGTGGGTCACGCCGTGCACCATGTG
CACATGGAGGAGAAGCGCAAGGAGCGCGAGGAGGCGGAACTCAGCCGGCAGCAGGAGCTG
AGCGAGGAGCGTCTCCCCCTGGCACCCGACCAGGGCAGCGTCCGCACCACCAAGGAGACC
AGCACCAAGGGCAGCAAGAAGTTCAGGCTGGAGGGCACCCTGCTGAGGACCTACATTTGC
CACATCATCTTCAAAACACTGTTTGAGGTGGGCTTCGTGGTGGGACAGTACTTCCTGTAC
GGCTTCCGCATCCTGCCGCTGTATAAATGCAGCCGCTGGCCCTGCCCCAACACGGTGGAC
TGTTTTGTGTCCCGGCCCACGGAGAAGACCGTCTTCATCATCTTCATGTTGGCCGTTGCC
TGCGTCTCGCTCTTCCTCAACTTTGTGGAGATCAGTCACCTGGGCCTGAAGAAGATCCGC
TTCGTTTTCCGCAAGCCGGCCCCGGCCCCGGCTCAAGGGGAGGGCACGGCCCCCCTGCCG
CCACCAGGAAAGAACTTGCCCCCTCTGGCTATGCCAGCCCTTCAAAGAGCGAAGGGTTAC
AGGCTGCTGGAGGAGGAGAAAGCTCCCATGACTCAGCTCTACCCGCTCACCGAGGTGGGC
ATGGAGGCTGGCAGAGGGCCCCCACCCTTCCTGGGGCTGGAGGAGAAAGCGGAGGAGGTG
CTGCCAATGGGGGGCATCTCTAAGGCGTACGACGAGACTCTGCCCTCCTACGCCCAGACC
ACCGAGACGGCGGGGGTGACGCTACGCCAGGAGGCCGAGGAGGTGCAGCCGGCAGAGGCA
GAAGCAGAGAGAGTGGAGAAGGGTGCGGATGAGGATCTGGAGGTAGAGGAAGCGGGGAAC
GGGGAGGGGGTAAATCCAGAGGAGACAAGGATGGAGGTCACGGATACGATAGAAGACACC
AGACCGCTGAGCCGACTGAGCAAAGCCAGCAGCAGGGCCAGGTCAGACGATCTTAACGTA
TGA

>Ga-GJA9-G13675 Our modification. Underlined: Intron predicted by Ensembl is included as part of exon.
ATGGGCGACTGGAACTTCCTCGGCGGGGTTTTGGAGGAGGTGCACATCCATTCCACCATG
GTGGGCAAGATCTGGCTCACCATCCTCTTCATCTTCCGCATGCTGGTCCTCGGCGTGGCG
GCGGAGGACGTGTGGAACGACGAGCAGGCGGACTTCGTGTGCAACACCGAGCAGCCCGGC
TGCAGGAACGTGTGCTACGACCACGCGTTCCCCATCTCCCTCATCCGCTTCTGGGTGCTG
CAGGTCATCTTTGTGTCCTCTCCCTCGCTGGTGTACATGGGCCACGCGCTCTACAGGCTG
CGGGCGCTGGAGAAGGCCCGGCAGAAGAAGAAGGCGCTGCTGCGGAAGGAGCTGGAGCTG
GTCGACGCGGAGTCGGCGGAGGCCAGGAAGAGGATCGAGCGGGAAGTGAAGCAGCTCGAC
CAGGGCAAGCTGAACAAAGCCCCCCTGAGGGGCTCGCTGCTGCGAACCTACGTGGCGCAC
GTCTTCACCCGCTCGGTTGTGGAAGTGGCCTTCATGACAGGCCAGTACGTCCTTTACGGC
TTTCACCTCCACCCGCTCTTCAAGTGCGAGCGGGACCCTTGTCCCAATGCCGTGGACTGT
TATGTGTCCAGACCGTCAGAGAAAAGTGTCTTCATGGTGTTCATGCAATGCATCGCGGCC
ATATCCCTCTTCCTGAACCTCTTGGAGCTCACGTACCTGGGCTACAAGAAGGTCATGCAG
GGCATCTTGGACCTTTACCCTCACTTGCAGGACGAACCCGATGACTACTGCGCCAACAAG
TGCAAAAAAGAATCTGTTGTGCAAATATGCACCAGCGTACCCCGAAGGGTGACGGTTGCC
TCCGCACCCTGTGACTACAACCTCTTGTTGGAGAGGTACCCGAACCTCCTCAGACCTCCA
TCTTTTCTCCCTCATCGGAGCGAGCAGACCCACCAGCAGTATTTGGAGGATCCCCCGCAC
GGCAAAGAGGACGGAGGGAATCCAGACTCTCCAAAGAAGGCCGACTCAAACTCCAGCCCG
GAGACACCGGAAGAGTCCAGCTCAGGATCCAGGGACAACCCCAGGCCGCCTTCTTCCAAC
GGCAAGACGCCAGTGGACAGACGGCCGGCAGACCTGCAGATCTGA

>Ga-cx52.9-G02230 Our modification. Underlined: Intron predicted by Ensembl is included as part of exon.
ATGGGAGACTGGAACTTCCTTGGAGGGATCTTGGAGGAGGTGCACATCCACTCCACCATG
GTCGGCAAGATCTGGCTCACCATCCTGTTCATATTCCGGATGCTGGTGCTGGGCGTCGCG
GCGGAAGACGTGTGGAACGACGAGCAGTCCGACTTCGTCTGCAACACCGACCAGCCGGGC
TGCCGCAACGTCTGCTACGACCAGGCCTTCCCCATCTCCCTCATCCGCTACTGGGTGCTC
CAGGTCATTTTCGTGTCCTCGCCCTCCCTGGTGTACATGGGCCACGCCATCTACCAGCTG
CGGGCTCTGGAGAAGGAGCGGCACTGCAAGAAGGTGGCCCTCCGTCGCGAGCTGGAGGCG
GTGGACGCGGAGCTGGTGGAGGTGCGGCGGAGGATTGAGAGGGAGATGAAGCTGCTGGAG
CAGGGGAAGCTCAACAAGGCTCCTCTGAGGGGCTCTCTGCTGTGCACCTACCTGGTCCAC
ATCGTCACGCGCTCAGTGGTGGAGGTCAGCTTCATGGTGTGTCAGTACTTCCTCTACGGA
CACCGGCTGAACCCGCTCTACAAGTGTGAGCGGGAGCCGTGCCCCAACGTGGTCGACTGC

TTCGTCTCCAGGCCCACCGAGAAGACGGTGTTCATGGTGTTCATGCAGGGGATCGCCTGC
ATCTCGCTCTTCCTCAGCCTCCTGGAGATCATGCACCTGGGATTCAAGAAGCTCAAGAGG
GGCATCCTGGACTACTACCCGCACCTGAAGGCCGACCTCGACGAGTACTACGTGGACAAG
TCGAAGAAGGACTCGGTGGTGCATCAGGTGTGCGTGGGCACGTCCGTGGGTCGCAAGACC
ACCATCCCCACGGCGCCGTGTGGGTACACGTTGCTGTTGGAGAAGCAGGGCAACGGGCCC
GCCTACCCTCTCCTCAACGCCTCCTCTGCCTTCGTCCCGATCAAAGGGGACCCTGTCGCA
AAGCCGGACCTCCACAAGGACGGCAAGGAGGGCGTCCCGAGCCCCACGGAGCAGAACAGC
AACTCCAACAACACGAGCAGCGAGACGCGCTCCCCTCCTTCAGACAAACAGGAGGAGCCG
GAGGAGCAGTCTTCGCCCCCTCTGGAACGCATGGGGTGCACCAGCTCCGAGTATCCGACC
CTCCCCGTGGCCTCATCGTGCGCAACGATGTCAGGAGCTGCGAGGAAGTCGCGGAGGGTC
AGTCCACCGTGGAACTGCTCCACGCTGGTGGAAGGCAACGGGTCGGACAGCGGAGACTCC
TATCAAGGGAACAACGGCGGGAAGCCGCGTGGCGGCTGCGTCGGACCCCGAGCGAGGGTG
CTCTCCAAATCAGACACGAAGAGGCCGAGCAGGCCTCAGAGCCCGGACTCCGCAGGGGAG
CTGAGCTCAGTGTCTCGACACAGCCGCGAGAGCAACAGCCCCGTCCCAGCCTCTCCCAGC
CGCCGCGTGTCAGCGGTGAGCAGCAACGGCAGCAGAAGGGCCCCAACTGATCTGCAGATA
TGA

>Ga-cx52.6-G06243 Our modification. Underlined: Intron predicted by Ensembl is included as part of exon.

ATGGGCGATTGGAACTTATTAGGGAGCATCTTAGAAGAGGTCCACATTCACTCCACCATC
GTGGGAAAGATCTGGCTCACCATCCTCTTCATCTTCCGCATGCTGGTGCTCGGCGTGGCC
GCCGAGGACGTGTGGGACGACGAGCAGACCGAGTTCGTCTGCAACACGGAGCAGCCCGGC
TGCAAGACCGTCTGCTACGACCAGGCTTTCCCCATCTCCCTCATCCGCTACTGGGTGCTG
CAGGTCATCTTCGTGTCCTCCCCGTCCCTGGTCTACATGGGCCACGCGCTGTACCGCCTG
AGGACCCTGGATAAGGAGAGGCACAGGAAGAAGGCCTCCCTAAAAGCCGAGCTGGAGGGG
ACGGACCCCGTCCAGGAGGACCACCGTAGGATCGAGCGGGAGCTCAGGAAGCTGGACGAG
CAGAAGAAGGTGAGGAAGGCGCCCCTCAGGGGCTCGCTGCTGCGCACCTACGTTTTCCAC
ATCCTGACCAGGTCCGTCGTGGAGGTGGGCTTCATCATTGGCCAGTGCGCTCTGTACGGC
ATCGGGCTGTCTCCCCTCTACAAATGCGAGCGGTTGCCTTGCCCCAACAGCGTGGATTGT
TTCGTGTCGCGGCCGACGGAGAAGAACATTTTCATGGTTTTCATGCTGGTCATCGCCGGG
GTCTCTTTGTTCCTCAACCTCCTGGAGATCTTCCACCTGGGGGTGAAGAAGATCAAACAG
AGCCTGTACGGATACAAATACGGGGACGACGACAGCGTGTGCAGGTCGAAGAAGAACTCC
ACGGTGCAGCAGGCGTGCGTGCTCAACAACTCCTCGCCGCAGAGGCTGATGCAGCTCACG
CACATGTCCTGCCCCCGGGTGTCGGACGCTCACAGGAAGCCTTCGGACCCCCAGTGCAAG
GAGGGCCCGGCCCACCGGGCGCCCTCGTGCAGCAGCGACGAGTCCACCGGAGGCCGAGGA
GCCCCGGGCCGGCCGCAGTACGCGGGGCCCCGGCCCACCCTGAGTGCCGGCCACATGGAG
ATCCCGGCTGCCCTGAGGAACCCGCAGAGGAAGCACAGCAAGGTGAGCGCCTGCAAGGAG
CTGAGCGACATGAGCGATTCGCCCGAGAGCGACTACCACCCCACGGGCAGGAAGTGCAGC
TTCATGTCCCGCGGGATGTCGGAGAGCAAGCTGGCCTCCTCGTCCGACAGCGCCGACTCC
CGCAGCTTAGGGGACGTCGAGGCCCAGCACTTCAACCAGGGGGAGAGTCCGGCGGTGACA
CCGCCACCTCCGTCGAGCGGGAGGAGGATGTCCATGGTGAGGGCAAAAAAAACAAAAACA
AATGTCTTCCAGTCATGTTTTGTCTGGTGGCCCCTGATCAAGGATACTAAATGGGCCTCG
CAAAGGGGGGCTGAGTTATGGGCCTTTTTTCATTTTTTCAAACATTCAAAAGGCTTATAA

>Ga-NP-gja10

ATGGGTGACTGGAACCTGCTGGGCAGCATCCTAGAAGAGGTCCACATTCATTCTACTATC
GTGGGCAAGATCTGGCTTACCATACTTTTCATCTTCCGCATGCTGATCCTGGGTGCGGCC
GCTGAGGACGTATGGGATGATGAGCAGTCCGAGTTTGTCTGCAACACTGACCAGCCAGGC
TGCAAGGCGGTCTGCTACGACCGTGCCTTCCCCATCTCCCTCATACGTTTCTGGGTCTTG
CAGGTGATCTTTGTCTCTGCGCCATCGTTAGTCTACATGGGCCATGCCCTCTACTGCATG
CGAGCACTTGAGAAGGAGCGCCACCGCAGACGGGCCCAGCTGAAGGAGGAGCTGGATGAG
GTGGAGTTGGCGCTGGACGAACATAAGCGCATGGAGAGGGAACTGAGGAGGCTAGACGAG
CAGAGGAGGGTGAAGAAGGCTCCTCTCAGAGGCTCTCTATTGAGAACGTACATCATCCAT
ATCCTTACACGCTCTCTGGTGGAGGTCTGCTTCATTTTCGGCCAGCATATACTTTATGGT
GTCCAACTAGAGCCCCTCTATAAGTGTGATAGGCTACCTTGCCCCAACAGTGTAGATTGT
TACATCTCCAGGCCCACGGAGAAGACAATATTCATGGTTTTCATGATTGTCATTGCTGGT
GTGTCACTGTTCCTCAACATACTGGAAATATCCCACTTGGGAATCAGGAAAATCAAACAG
ACACTGTATGGAGAGAGGTACACGGAAGATGACAGTTTGATTTACAAGGCTAAGAAGAAG
TCGTTACCACACCTTTGTGTAATGAGTAATGTATCACCTCACAACGGGCCTTTGACTCAG
ACCTTCAAAGTGATTCCAGAGGCAGATATGAAGCCTCCATATTACAATACTGTGCTCAAA
GCCAACCAGGAGGCACCAAGACACAACAGCTTGGCCTATATGGGACACAGTCAGACCAGC
TATATCTGTCCCGAACCTAGGATGCCGCCCGGGTCTGGCAGGAACTTTGCAATTCAAGCC
CCCAAAACCCATGAAGGTCCAGAAATCCGCACAGCTATGGTGGACCATCATCTGGCCTGG
GCTGCTGTATCAACTGTGGAGGGAAACGCAACAAACCATCATCCAGATCCCCATGAGGGA
GAGTGCCCTCACTCTACCCATTTGGAAGCTCTGCTGTCCACTAGCACCTTAAGGCCCAGC
GCTATCAGAGACCTGGATGAGAATCATCGAAGGGAGTCAAATGAGAGTGAAGTCCTGCTA
TCCAACCCCAGGAAGACCAGCTTCATGATTAGGCCACCATCTGACAGCTTGTCTTCTATC

AGTGACTCCACCAGCCCTTCCTTGCATACCTCAGAGGAGTCAGATGAACTGGGCTCCCTG
CAGGGAGACATGCCAATGATGCCGCCTGCTGGAGGCCGAAGAATGTCAATGGCAAGTAAA
GAGTGGAGATCTTCTAATGTGCTGGAATTTGCTTTTACCTGCCTTTGGTGTCTAGAGCCT
AATAGTCTATATATGTTATATGTATGA

>Ga-cx28.9-G06833 Our modification. Underlined: Exon extended until stop codon. Splice site.
ATGGGAGAGTGGGGTTTCCTGTCCTCTCTACTGGACAAGGTCCAGTCCCACTCCACCGTC
ATCGGGAAGGTCTGGCTCATTGTTCTTTTCATCTTCAGGATCATGATCCTCGGAGCTGGA
GCAGAGAAGGTGTGGGGTGATGAGCAGTCAAATATGGTTTGCAACACCAAACAGCCTGGT
TGCAAGAACGTCTGCTATGACCAAGCCTTCCCAATCTCACACATTCGATTCTGGGTCCTC
CAGATTATCTTTGTGTCAACTCCAACGCTGATCTACCTCGGCCACGTCCTCCACATTATC
CACAAAGAAAATAAGATGAGAGAACAGACGCTGACCTACTCCAAGACCGGAATTGTCAAA
GTTCCAAAGTACTCCGACGACAAAGGCCACGTCAAAATTAAAGGCGACCTGCTGGGAAAC
TACATGACCTCTATTTTCTTCAGAATCCTCCTGGAGGTAGCGTTCATTGTTGGGCAGTAT
TATCTCTATGGGTTCATCATGGACCCAAGAGTGGTCTGCACCCGAGCCCCTTGTCCATTT
ACCGTGGAGTGCTACATGTCTCGGCCAACAGAGAAGACCATCTTCATTATCTTCATGCTG
GTGGTGTCCATCATCTCTCTCGTACTGAACGTAGCGGAGATCTTCTACCTGGGGTGTACT
CGCTCAATCAGGCAAAGGTCTAAAACACATAAAGCATCAATTGCCATTCACACTCGTTTA
AACGGGGACACTCTTATGTAA

>Ga-cx32.3-G06829 Our modification. Underlined: Intron predicted by Ensembl is included as part of exon.
ATGGGAGACTGGGGTTTCCTGTCCGGCCTACTGGACAAGGTCCAGTCCCACTCTACGGTC
ATCGGCAAGATCTGGATGAGCGTGCTCTTCCTCTTCAGAATCATGGTCTTGGGTGCGGGC
GCGGAGAGCGTCTGGGGCGACGAGCAGTCGGGTTTCGTCTGCAACACCCAGCAGCCTGGT
TGTGAGAACGTCTGCTACGATTGGACCTTCCCCATCTCGCACATGCGTTTCTGGGTCCTT
CAGATCATCTTCGTCTCAACTCCGACGCTGGTGTACCTGGGCCACGCCGTGCACGTCATC
CACCAGGAGAACAAGATGAGGGAGCAGCTGTTGAGCGCAGCTGGGTCCCGGCTGTGCAAA
CAGCCCAAGTACACCAACGAAAGGGGAAAGGTGATGATCAAGGGGAACCTGCTGGGGAGC
TACATGACCCAACTCGTGTTCAAGATCTTCATCGAGGCCGGCTTCATCGTGGGCCAGTAC
TACCTTTACGGCTTCGTCATGGTGCCCATGTTCCCCTGCTCTCAGACACCCTGTCCCTTC
ACCGTGGAGTGCTACATGTCCCGACCCACAGAGAAGACCATCTTCATCATTTTCATGCTG
GTGGTGGCCTGTGTCTCCCTGTTCCTCAACTTCCTCGAGATGTTCTACCTGATTTGTACC
AGGGTCCGGTGTGGGTCCAGGGCTCGCTCTCGCAAGATCACTACGGCGGATAACCCTGCG
AGCCTGTCGACTCCCCGATGGCCGACGGCAGACGACGCGCTCAGGCACAACAAGGTGAAC
ATTGAGCTGGAGGGCAGCCAGAGCGTCGGTGGGAGCCTGGATGGAGCCAAAGAGGAGAAA
CGACTACTGAGTGGTCATTAA

>Ga-cx31.7-G18314 As predicted by Ensembl (transcript ENSGACT00000024257) and in GenBank AAY27079.1.1 (Q50D51_GASAC.1)
ATGAACTGGGGGAGCTTTTACGCCGTGATCAGCGGCGTAAACAGGCATTCCACCGGCATC
GGGCGCGTCTGGCTCTCCGTCATCTTCATCTTCCGCATCCTGGTCCTGGTGGTCGCTGCC
GAGAGCGTCTGGGGAGACGAGAAGTCCGGCTTCGTCTGCAACACCCAGCAGCCCGGCTGC
AACAGCGTCTGCTACGACCAGTTCTTCCCCATCTCGCACATCCGCCTGTGGGCGCTGCAG
CTCATCCTGGTCTCCACCCCCGCCCTGCTGGTGGCCATGCACGTGGCCCACCGACGCCAC
GTTGACAAGAAGGTCCTGAAGAAGACGGGCCGCGGCGGGCCCAAGGAGCTGGAGCTCATC
AAGAACCAGAAGTTCCAGATCACCGGAGCGCTGTGGTGGACGTACATGATCAGCATCGTC
TTCAGGATCGTCTTGGAGGTGGCTTTTCTCTACATTTTCTACTTGATCTATCCGGGCTTC
AAGATGGTGCGCTTGGTGAAGTGTGCGTCGTACCCGTGCCCCAACACGGTGGACTGCTTC
GTCTCCAGACCGACAGAAAAGACCATATTCACTGTGTTCATGCTGGCGGTGTCCGGGCTG
TGTGTGCTGCTCAACCTGGCCGAGGTGGCCTACCTCATATTCAGGGCCTGCAAGCGGTGC
CTCCGAGGCTCCGAGGAAGAGTCCAAAGTCGCTTGGATAAGTGGAAGATTCTCCACTTAT
AAGCAAAATGAAATCAATCAGCTGATAGCGGAGCAGGCGCTCAAGTCTAAGTTCGCTGTG
AGCAAAAAGAGCCCGACCGAGAAGGGAGAAAGGTGTTCGGCATTCTGA

>Ga-cx27.5-G20330 As predicted by Ensembl
ATGAACTGGGCGTCGTTTTACGCTGTCATCAGCGGTGTGAACAGACACTCGACAGGCATC
GGTCGCATCTGGCTCTCTGTCCTGTTCATTTTCCGTATCCTGGTCCTGGTGGTTGCAGCG
GAGAGTGTGTGGGGCGACGAGAAGTCCGGCTTCACCTGCAACACCCAGCAGCCGGGCTGC
AACAGCGTCTGCTACGACCACTTCTTCCCCATCTCCCACATCCGCCTGTGGGCGCTTCAA
CTCATCCTGGTGTCCACCCCCGCCCTGCTGGTCGCCATGCACGTGGCCCACCGGCGCCAC
GTCGACAAGAGGCTCTACAGACTTTCGGGGAGGACCAATCCCAAAGACCTGGAGCAGATA
AAGACCCAGAAGATGAAAATCTCTGGGGCTCTGTGGTGGACGTACGTCATCAGCCTGTTG
TTCCGCATCGTCTTTGAGGTGACCTTCATGTATCTGTTTTATATGATCTACCCTGGTTAC
AAGATGATCCGGCTGGTGAAGTGCGACTCGTACCCGTGTCCCAACACGGTGGACTGCTTC
GTGTCGAGGCCCACGGAGAAGACTGTCTTCACCGTGTTCATGCTGGCTGTATCGGGGGTT
TGTATTCTGCTCAACATTGCGGAGGTGATCTTCTTGGTGGGGAAGGCCTGCAGTAAGCAT

CTGCACGCTGCTGGAGACTCGACTGTCGGGGCTTGGATCCAACAAAAGCTCTGCTCATAC
TAA

>Ga-cx30.3-G01368 Our modification. Underlined: Intron predicted by Ensembl is included as exon.

ATGTCTTGGGGGGTGCTCTACGCCCAGCTGGGCGGAGTCAACAAACACTCCACCAGCCTG
GGGAAGATCTGGCTCTCCGTCCTCTTCATCTTCCGCATCACCATCCTGGTCCTGGCCGCC
GAGAGCGTCTGGGGCGACGAGCAGTCCGACTTCACCTGCAACACGCAGCAGCCCGGCTGC
AAGAACGTCTGCTACGACCACTTCTTCCCCGTGTCTCACATCCGCCTGTGGTGCCTGCAG
CTGATCTTCGTGTCCACGCCGGCGCTGCTGGTGGCCATGCACGTGGCCTACAGGAAGCGC
GGGGACAAGAGGACCATGCTGGCCTCCAACGGCGCCGAGCGGACGACGGACAACGAGCTG
GAGACGCTGAAGAGGAGGCGCCTGCCCATCGCGGGCCCGCTGTGGTGGACCTACACCTGC
AGCCTCTTCTTCCGCCTCGTCTTCGAGGGCGGCTTCATGTACGCGCTGTACTTCGTGTAC
GGCGGCTTCCAGATGCCGCGGCTGGTGAAGTGCGAGCAGTGGCCCTGCCCCAACAAGGTG
GACTGCTTCATCTCCAGGCCCACGGAGAAGACCGTCTTCACCATCTTCATGGTGTCCTCG
TCCACCATCTGCATGGTGCTGAACGTGGCCGAGCTGGGCTACCTCATCGCGAAGGCGGCG
CTGAGGTGCTCGGCCCGGTCCAACCGGAGGAACCGCCCGTACGGCCACGCGGACGGCGTG
CCGCAGGACAACAGCCACCTTCAGAACGTGAAGAACGAGCTGCTGTCTGCCGACTCGCGC
GCCAGCAGGACGTGCTGA

>Ga-NN-cx30.3-G14074(1) There are two connexins (wrongly) fused into one Ensembl prediction. For the present connexin, Ensembl only predicts (correctly) the first exon. Underlined: Exons predicted by us. Splice sites. The second sequence is Ga-NN-gja3-G14074(2)

ATGTCTTGGCCGGCTCTGTACTCTCAGTTGGTCGGGGGGAACCGACACTCCACCAGCTTG
GGTAAAATCTGGCTCTCGGTGCTCTTCATTTTCCGGGTCATGGTGCTGGTTGTCGCTGCT
GAGAGCGTCTGGGGGGACGAACAGTCTGACTTCACTTGCAACACACTACA**GC**CTGGCTGT
GAAAACGTCTGCTACGACCAGTTCTTCCCTGTCTCCCACATCCGTCTATGGTGTCTCCAG
CTTGTCTTTGTCTCCACGCCAACACTCCTGGTTGCAATGCATGTGGCCTATCGGAACCAC
AGTGACAAAAAGAGGCTCCTACA**GG**GTTCAGGTAGAGCGGGTTTCCTCACCAGCAAAGGC
CAGGAGGAAGACCTGGAGACTCTGAGGAGAAGGAGACTCCCAATAGCTGGCGCCCTCTGG
TGGACGTACGCCTGCAGCCTGGTGTGCAGGTTACTGTTTGAAGGAGGCTTCA**TG**TATGCC
CTGTACGTGGTGTACGACGGGTTCCAGATGCCTCGCTTGGTGCAGTGTGACCAGTGGCCG
TGTCCAAACCTGGTGGACTGCTTCATCTCTCGCCCCACCGAGAAAACCATCTTCACCGTC
TTCATGGCCACCGCCTCCTCTATCTGCATGGTCCTTAACATGGCGGAACTTGCATATCTT
GTTGCCAAGGCTGTCACTAGGTAG

>Ga-cx34.5-G06828 Our suggested modification Our modification. Underlined: Intron predicted by Ensembl is included as part of exon. Splice sites. At first splice site, a part of the exon predicted by Ensembl is now considered as a part of the intron.

ATGGGCGAATGGGACGTGCTGGGCCGCCTTCTGGATAAAGTGCAGAGTCACTCCACCGTG
ATCGGCAAGGTCTGGCTCACCGTGCTGTTTGTCTTCCGCATCCTGGTCCTGCGCACCGGC
GCCGACAAGGTGTGGGGCGATGAGCAGTCCGACTTTGTCTGCAACACCCTGCAGCCCGGC
TGTGAGAACGTCTGCTACGACATGGCCTTCCCCATCTCTCACGTGCGCTTCTGGGTCCTT
CAGATCATCGCTGTGGCCACTCCAAAGTTGCTGTACCTCGGTCACGTCCTCCATGTGATC
CACCTTGAGAAGAAGATGAAGGAGAGGATGAAGAGGCATGCTGACTTGGACAACCAGATC
AGTCTGCTCCTTAGAAGGGCCTACAAAGTTCCCAAGTACACCAAGAGCACCGGCAAGATC
AGCATCCGCGGGACTCTGCTTCGCAGTTATGTCCTCCACCTCGTGGCCAAGATTGTCCTG
GAAGTCCTGTTCATCGTGGGTCAGTACTATCTGTACGGCTTCACCCTCAAGGAGCGCTAC
GTCTGCGCCCGCTCTCCGTGCCCCCACCAGGTGGACTGCTTCCTGTCGAGGCCGACGGAG
AAGTCGGTCATCATCTGGTTCATGCTGGTGTCGGCGGTTGTCTCTCTCTTCCTCAGCCTG
ATTGAGCTGCTCTACCTGTGTGTGAAAGCTGTGAGGGAGTGTATGACCAGGAGGCAGGAC
TACACCGTGACCCCGGTGACCCCTCCGCCTTTGGAGAGGAAAGCTTTTAAAAGCCGCGAC
GAGATGCTCCAGAATGGTGTCAACCTGGAGCTGGAGCTCCGTGGAGGAAAGCCAGGGGCG
AACGGGGCCGGAGGCGGGTCCACCGAGGCTGCTGTGGATGTGTCGCTGGAGAGCAACAAC
ACGGGAGGGGAGGTGCACATCTGA

>Ga-NN-cx35.4-G09240 Our modification. Underlined: Exon extended until stop codon.

ATGGATTGGAAATTCCTCGAGGGGCTCCTCAGCGGAGTCAACAAGTACTCCACTGCCTTC
GGACGCATCTGGCTCTCGGTGGTCTTTGTCTTCCGCGTGCTGGTCTTCGTGGTGGCCGCC
GAGCGGGTCTGGAGCGACGACCAGGGACACTTCGACTGCAACACCCGCCAGCCGGGTTGC
ACCAACCTCTGCTTCGACTACTTCTTCCCCATCTCCCACATTCGCCTCTGGGCTCTGCAG
CTCATCTTCGTCACCTGCCCCTCCTTCATGGTGGTGCTGCACGTGTTCTACCGGAAGAAG
CGGGACCGCGAGTACCGAGCCAAGCACGGCGAGGACACCGGGCTGTACGACAACCCGGGC
CAAAAGCACGGCGGCCTGTGGTGGACGTACCTGATGAGCCTCTTCATCAAGACCTTCTTC
GAGATCACTTTCCTCTACCTGCTGCACTACGTGTACGAAAGCTTCCGGCTGCCCAGGAAG
GTGCAGTGCGACGTCAAGCCCTGTCCCAACCTGGTGGACTGCTACATATCCCGGCCCACC

GAAAAGACCGTCTTCACCTACTTCATGGTGGGGGCGTCCGTCGTGTGCGTGGTGCTCAAC
GTTTGCGAGATCTTCTATCTGGTTGCTTTCCGGATGGTGACCTTGAAGAGGACAGGCAGC
GTCCACACCTCGTCCAGGAAGGTGCACCCGAACGCGAACGACTGCAAGTCCTCTCTCGCC
TCTTAA

>Ga-cx35.4-G07158 Our modification. Underlined: Exon extended until stop codon.
ATGGACTGGAAGACCTTCCAGGCCCTCCTCAGCGGGGTGAACAAATACTCCACGGCGTTC
GGCCGGGTATGGCTGTCCGTGGTGTTCGTGTTCAGGGTGATGGTGTACGTGGTGGCGGCG
GAGCGGGTGTGGGGCGACGAGCAGAAGGACTTTGACTGCAACACCAAGCAGCCCGGCTGC
GCCAACGTCTGCTACGACCACTTCTTTCCCATCTCCCACATCCGCCTGTGGGCCCTGCAG
CTCATCTTCGTCACCTGCCCGTCCTTCATGGTGGTCATGCACGTGGCGTACCGCGACGAC
CGCGAGCGCAAGTACAAGGCGAAGCACGGAAACACCACCCAGCTGTACCAGAACACGGGC
AAGAAGCACGGCGGCTTGTGGTGGACCTATCTGATCAGCCTCTTCGTGAAGACGGGCATC
GAGGTCTCCTTCCTCTACATCCTCCACCACATCTACGACAGTTTCTACCTGCCGAGGTTG
GTCAAGTGCGGTGTGTCGCCCTGCCCCAACCTGGTGGACTGCTACATCGGCCACCCCACC
GAGAAGAAGGTCTTCACCTACTTCATGGTCGGAGCTTCGGGCCTCTGCATCGTCCTGAAC
ATCTGCGAGGTCATTTATCTCATCTCCAAACGCGTTGTTCGGATAGCGAGGAAGGCCAGG
ACTCACCGCCGCACCCCACCCCTGCCTCTGGACGTGCCTCTGGACGTGTACAAGGACCAC
TTTGACAACGCCGACAAGATCATGTCGAGGAGGTACCTGAAAGATCAGCCTCCGTCCTTT
AAGACGGCAACCAAATCTCCGCCCAGGATATCTGGGCTCAAAATGGAAGACAGGTTTCGG
GCCTCTGCTCCTAATCTGTCCATTTCTTAG

>Ga-cx30.9-G07404 Our modification: Underlined: Sequence is extended in 3’-direction to reach a stop codon.
ATGAACTGGTCTGCTTTGGAGTCCCTCATCAGTGGGGTCAACAAGTACTCCACCGTGTTC
GGGCGCATCTGGCTCTCCATGGTCTTCATCTTCCGGGTGTTGGTGTTCGTGGTGGCGGCC
CAGCGGGTGTGGGGCGACGATAACAAGGACTTTGTCTGCAACACCATCCAGCCGGGCTGC
ACCAACGTGTGCTACGACCACATCTTCCCCATCTCCCACATCCGCCTGTGGGCCCTGCAG
CTCATCTTCGTCACCTGCCCGTCCCTGATGGTGGTGGGCCACGTCAAGCTTCGCGAGAAG
AAGGACATGCAGTACACCGCCTCGCACATGGGCGCCCATCTGTACGCGCACCCCGGGAAG
AAACGAGGGGGCCTGTGGTGGACGTACCTGGCGAGTCTGATTTTCAAGGCAGGCTTTGAT
GCTGGCTTCCTCTACATCCTGTACTACGTCTACGAAGGCTACGACATGCCCCGCCTGTCC
AAGTGCTCCCTGCAGCCCTGCCCCAACATGGTGGACTGCTACATATCGCGTCCCACTGAG
AAGAGGATCTTCACCATCTTCATGGTGGTCTCCTCTGCGCTGTGCATCCTAATGTGCATC
TGCGAGATAGTTTACCTCATCGGCAAACAAATCCAGAAACGCATCAAGAAGAAGTACAAC
GCAGACAGGATACTGTCCAGAGTCGATCCTACGGCCTCCACTCAAAATCTCAGTAACGCC
AAGAAAGAGAAGGCGCCTATTAGGGAGAAAATTATGCACGATAGAGCAATGAAGCTTTTT
GAAAAAGAAATGTGGAAAAGCATTCATCAGAGCCAATGA

>Ga-cx28.6-G07635 Our modification. Underlined: Intron predicted by Ensembl is included as part of exon. Splice site. At first splice site, parts of exons predicted by Ensembl are now considered as introns.
ATGAACTGGTCAGGACTTGAGAGCTTGTTGAGCGGAGTCAACAAATACTCCACTGCGTTC
GGGAGGATTTGGCTTTCCATGGTGTTTGTGTTCCGTGTCATGGTGTTTGTGGTGGCAGCG
CAGAAGGTTTGGGGCGACGAAAACAAAGACTTTGTGTGTAACACGCGGCAGCCCGGCTGC
ACCAACATCTGCTATGACCACATCTTCCCCATCTCCCACATCCGCCTGTGGGCGCTGCAG
CTGATCTTTGTCACGTGCCCGTCCCTGATGGTGATGGCTCATGTCAAATTCCGCGAAGGA
AAGGACAAGAAATACGAGGAGCAGCACCACGGCTCCCACCTGTACGCCAACCCCGGCAAG
AAGAGAGGGGGCCTGTGGTGGACCTACCTGCTGAGCTTGGTCTTGAAGGCTGGATTCGAC
ATGTCCTTTCTGTACATTCTGTACCGGATATACCACGGATATGACTTGCCCAGGCTATCC
AAGTGTTCGCTGGAACCGTGCCCCAACACCGTGGACTGCTTCATCAGCCGCCCCACGGAG
AAGAAGATCTTCATGTTGTTCATGGTCGTTTCCAGCGCGGTGTGCATCTTGATGTGCTTC
TGCGAGATGATTTACCTCATCGGCAAGCGCGTCGTCAAAGAGATGAGGCTCCGCAAGAAC
AACGAGATGCTCCGGTTTGCTGAGGAGCACGAGCTCACCAACATGGCCCCACCCAGGTCG
CAGTATCGCAGGGTCGATCCAACGCTGACAGACAGCCAGCTGAGTTTAAACAAGGACAGC
CAGCTGAGTTTAAACAAGGCGGACAGGGTCAGAAACAGTGCTATGAGTACGAACTTGTAG

>Ga-cx34.4-G07159 Our modification. Underlined: Intron predicted by Ensemble is included as part of exon.
ATGAACTGGGCATTCCTCCAGGGCCTCCTCAGTGGGGTGAACAAGTACTCCACGGCCTTC
GGCCGCGTTTGGCTCTCCATCGTTTTCCTCTTCAGGGTCATGGTATTCGTGGTCGCGGCG
GAGAAGGTGTGGGGAGACGAGCAGAAAGACTTTCTGTGCAACACGGCTCAGCCCGGGTGC
CACAACGTTTGCTACGACCACTTCTTCCCCGTGTCCCACGTCCGGCTGTGGGCCCTGCAG
CTCATCTTCGTCACCTGCCCGTCTCTCCTGGTGGTGATGCACGTGGCCTACAGGGACGAC
CGGGAACGAAAGAACCGGCTCAAGTACGGCGAGAACTGCCGCAGCCTCTACAAGAACACG
GGGAAGAAGCGCGGCGGCCTGTGGTGGACCTACGTGCTCACTCTGGTCTTCAAAATAGCC
GTGGACGCCACCTTCGTCTACCTCCTGTACCACATCTACGAGGGCTACGACTTCCCCTCG
CTCATCAAGTGCGAAGAGAAGCCCTGCCCCAACGTGGTGGACTGCTTCATCGCTCGGCCC

ACCGAGAAACGGATCTTCACCATCTTCATGGTGGTCACCAGCCTGGCCTGCATCCTCCTC
TCCATCTTTGAAATCCTCTACCTGGTGGGGAAACGCTGCTGCGAATGCGCCACCGCGGGA
AGAAGCTCTCGCCACGTCGACACCAGCACGCTGTCCAGCGGCGCGACCCTGATGGATTCG
AACACCCTTAAGGTGGCGGGCAAATCCCCCCCCGGGACGCCGGCTCCTTCGTACAGCGCG
GCCGTGTCTTGA

>Ga-NN-cx34.4-G09234 Our modification. Underlined: Intron predicted by Ensembl is included as part of exon. Splice site.

ATGAACTGGGCCTTCCTCGAGGGCCTCCTCAGCGGGGTGAACAAATACTCCACAGCGTTC
GGCCGCATCTGGCTCGCCATCGTCTTCATTTTCCGGCTCCTGGTCTTCCTGGTGGCCTGC
GAGAAGGTCTGGGGCGACGAGCAGAAGGACTTCGACTGCAACACCCGGCAGCCCGGCTGT
CACAACGTGTGCTACGACCACTACTACCCCGTCGCCTACACGCGCCTCTGGGCCCTGCAG
CTGATCTTCGTCACCTGCCCGTCCCTCCTCGTGACGCTGCACGTCTCCTACCGGGAGGAA
CGGGAGCGCAAACACCGGCTGAAGCACGGGGAGGACTGCCCCCCCCTGTACGACCACACG
GGGAAGAAGCGGGGGGGCCTGTGGTGGACCTACTTCTTCAGTCTGCTGTTTAAGATACTG
GTGGACGGCGTGTTCGTCTTCCTGCTGTTCTACATCTACGAAGCCACGTTCTTCCCGCCG
CTGGTGAAGTGCAACGAGGAGCCGTGTCCCAACGTGGTGGACTGCTACATCGCCAGGCCC
ACGGAAAAGAAGATCTTCACCATCTTCATGGTGGTGACCAGCTTTGTGTGCATCCTCCTC
ACTCTCATCGAGGTCTTCTACCTGTGCGGTAAGAGGCTCCGGGAGTGCTGCCGAGGAGGA
GGTCGCCCGGCGAGAGGGAACTCCTTCAAGATGGTCCGAACTCCTCTGAGCGGGAAGGAG
AACTCGGCCTACAAGGAGCCGTTCGCGCAGAAGGATAAGGCGGTGGACAAGGAGAGTTCG
GCGCCAGCGTACAGCGTCGCCATCTCCTGA

>Ga-cx28.8-G12273 Our modification Underlined: Extended until stop codon

ATGAACTGGGGCTTCTTGGAGAACATCCTCAGCGGGGTGAACAAGTACTCCACGGTGATC
GGCCGCATCTGGCTCTCCGTGGTCTTCCTCTTCCGGATCCTGGTGTACGTGGCGGCGGCC
GAGCACGTGTGGAAGGACGATCAGAAGGACTTTGTGTGCAACACCCGGCAGCCCGGCTGC
GAGAACGCCTGCTACGACCACTTCTTCCCCATCTCGCAGGCGCGCCTGTGGGCCCTGCAG
CTCATCGCGGTGTCCACGCCGTCCCTGCTGGTGGCCCTGCACGTGGCCTACCGGGAGCAC
CGCGAGGAGAAGCACCACCGCCGGCTGTACCGGGACAAAGGCAGCCTGGACGGGGGGCTG
TTCGCCACCTACGTGCTCAGCCTGGTCTTCAAGGTGAGCTTCGAGGCCGGCTCCCTGCTG
GCCTTCTACTACGTGTACGGCGGCTTCGCGGTGCCCACGCGCCTGCGCTGCAGCCAGAGC
CCGTGTCCCAACACGGAGGACTGCTTCATCGCCAGGGCCACGGAGAAGAAGGTCTTCCTC
TACATCATGGGCGCCACCTCCCTGCTGTGCATCGTCCTCAACCTGGCGGAGCTGGCCTAC
ATCGTGTGGAAGCACCTGTGGAAGTGCTTCACGCGGCGGTACGTGCCCGCGGGGGGCCGG
GCCCGCGCGCGCGTCTCCAGAGGCGACGGGTTCGCGTCCGCGGAGCCCGCGATGGGCCCG
CAGGTGGAGGACAGGATGGACCCGCAGGTGGAGGAGAGGGTGGACCCGCAGGTGGAGGAG
GGGAGGGGCGCGCAGGCCCCCCCCGCCCACTGA

>Ga-NN-gjc1-G09243 Our modification. Underlined: Introns predicted by Ensembl are included as part of exon.

ATGAGCTGGAGCTTCCTCACGCGGCTGCTGGAGGAGATCCACAACCACTCCACCTTCGTG
GGGAAGCTGTGGCTCACCGTGCTCATCGTCTTCCGCATCGTGCTCACCGCTGTTGGGGGA
GAGTCGATCTACTACGACGAGCAGAGCAAGTTTGTCTGCAACTCGGGCCAGCCGGGCTGC
GAGAATGTCTGCTACGACGGCTTCGCACCTCTGTCTCACGTTCGCTTTTGGGTCTTCCAG
ATCATCTTAGTGGCGATGCCTTCTCTCATGTACATGGGCTACGCTGTCAATAAGATCGCA
CGAATAGATGAGGCCAAAGGGGGCGGTGGATCTGCTGCAGTTAGAACAGGAGGAGGAGGC
TATACGCACAGGAAGCCCAGGAAAATCTGCTTTGGAGCGCGGCAGCACCGGGGCATTGAG
GAGACCGAGGACGACCAAGAGGATGATCCTATGATCTATGAAGTGCCAGAAATCGAGCCC
CCAAAAAGACCGCGGGATCCATTGCAACCCGCACCCAGACCCAAAATCAGGCATGATGGG
CGCAAGCGCATCAGAGACGAGGGGCTGATGCGGGTCTACGTTTTGCAACTGGTGACTCGT
ACGCTGCTTGAAGCAGGCTTCCTTGCAGGCCAGTATTTGCTGTACGGGTTTCGTGTGACG
CCCGTGTTTGTGTGCTCGAGGAATCCTTGTCCCCACAGCGTTGACTGCTTTGTGTCGCGT
CCCACGGAGAAGACCATCTTCCTGCGCATCATGTATGGTGTCACCGTCCTTTGCCTCACG
CTCAACGTTTGGGAGATGCTCCATCTGGGTATCGGCACCATTTGTGACATTCTGCGCCGC
CGGCGCCACCAACCTCAGGAAGATGAGTACCAGTTGGGATTGCTGGGCAACAGTGGAGGT
GTGGAGGGCTCAGTAGGCGCTGCAGGGCCCGAGGCAGGCTCCGAGGGAGGGGTGGGTGGA
GATGGGGCTGCAGACTATGTCGGTTACCCCTTCTCGTGGAACACCCCGTCTGCTCCACCT
GGCTACAACATTGTGGTAAAGCCAGAGCAGATGCCGTACACAGACCTTAGCAACGCAAAG
ATGGCGTGCAAGCAGAACCGGGAAAACATTGCCCAAGAGGAGCAGCAGCAATTTGGTAGC
AATGAGGATAACTTTCCCACCGGCGGGGAGGCCCGCGTAGCTTTGAACAAAGACATGATC
CAGCAAGCTCATGAGCAGCTGGAGGCGGCCATCCAGGCCTACAGTCAGCAACACCGAGCC
GAGGAACAGCTCGGGGACAACCGGGACGACAAGCCCCAAAGCAACATCATCCTGGCCCAA
CCTCAGCCTCAGCCTCAGCCTCAGAAAGAGCGCAAGCATAGATTCAAACACGGGAAAGGA
GGCAGCAGTGGAGGAGGCAGCAGCAGCAACAGCAGCAGCAGTAAGTCAGGAGAGGGTAAG
CCCTCCGTGTGGATTTAA

>Ga-NN-gjc1-G06369 Our modification. Underlined: Introns predicted by Ensembl are included as part of exon.

ATGAGTTGGAGTTTCCTGACTCGCCTGCTGGAGGAAATCCACAACCACTCCACGTTCGTG
GGCAAGATCTGGCTCACCGTCCTCATCGTGTTCCGCATCGTGCTGACGGCCGTGGGAGGC
GAGTCCATCTACTCTGATGAGCAGAGCAAGTTCGTCTGCAACACGGGCCAGCCGGGCTGT
GAGAACGTCTGCTACGACGCCTTCGCGCCGCTCTCGCACGTCCGCTTCTGGGTTTTCCAA
ATCATTCTGTTGACCACCCCCTCGCTCTTGTACCTGGGCTACGCCGTCAACAAGATCGCT
CGGGCAGATGAGCGGACCGGCTGCGGGGAGAGGAGGCCCGGGAAGCCGTATCTGGCGGGC
AGAAGGCAGCACCGCGGCGTCGAGGAGGCGGAGGATGACCAAGAAGAAGACCCGATGATT
TCCGAAACGGCGGAGGGGGAGGGCGATGGCGACGGACCGGTGAAAGGACGCAGCGATCCG
ACAAAGGTCCCGGTGCGCCACGACGGCCGCCAGCGCATCCAGGAGGACGGACTGATGCGC
ATATATGTCCTTCAGCTCTTGATCCGCGCCGCGCTGGAGGTGGCTTTCCTGTTCGGACAG
TATGCCTTGTACGGCTTCGCTGTGCCCCACACCTACGTGTGCTCGGCCCAGCCCTGCCCC
CACAGCGTGGACTGCTTTGTGTCGAGGCCCACTGAGAAAACCATCTTCCTCATCATCATG
TACACGGTCTCCCTGCTCTGCCTGGCGCTCAACATATGGGAGGTGCTTCACCTCGGCATC
GGCACCATCTGCGAGATTGTGCGCTCGCGCCAGGTGCAGCACCCCGACGACGAGCAGCGT
GGGCTGATGGGGGCACAGGTAGCTCCTCATGAGGAAAGGCTGGGGGCAGATGATTACAGG
CACTACTCTTTTTCTCGGAATGCCCCATCGGCCCACCCGGGTACAACGCCGCCATCGAGC
CTCTTCTGGTTACGACAAAGCACCGCGACAAGCCGCTACCCAACTCTGACATCAGCGACA
CCAAGACAGCGTCCCGGCAGAACCACGTGA

>Ga-cx47.1-G17416 Our modification. Underlined: Intron predicted by Ensembl is included as part of exon.

ATGAGCTGGAGCTTCCTCACTCGTCTCCTGGAAGAGATCCACAACCACTCCACCTTTGTG
GGGAAAGTGTGGCTGACGGTGCTCATCATCTTCCGCATCGTGCTCACGGCCGTCGGAGGC
GAGTCCATCTACTCGGACGAGCAGACCAAGTTCACCTGCAACACCAAGCAGCCGGGCTGC
GACAACGTCTGCTACGATGCGTTCGCCCCTCTCTCGCACGTCCGCTTCTGGGTCTTCCAG
ATCATCATGATCTCCACTCCCTCCATCATGTACATGGGCTACGCTATCCACAAGATAGCC
CGAACCACAGAGGAGGATCGCAGGAAGCGCCAGAGGCTCCGCAAGAAGCACACTCCTCAC
TCCAGATGGAGGGAGGGCCACCATCTGGAAGATGTCTTGGAGGAGGAGGAAGATGACGAT
GCTGAGCCCATGATCTACGAGGATCCGCTGGGGGAGCAAGAGGCCAAGCCCGAACCGATG
ACCAGCTTAGGCAAAGATCCGCCGAAACACGACGGCCGCCGAAGGATAATGCAGGAAGGC
CTGATGAGAATCTATGTGCTGCAGCTCATGTCCAGAGCTATTTTTGAAATCGCTTTCCTC
GCCGGACAGTATCTCCTCTACGGCTTTCGAGTTAGTCCATCGTATGTATGCAACAGGATC
CCCTGTCCACACAGGGTGGACTGCTTCATCTCCAGGCCCACAGAGAAAACAATCTTCCTC
CTCATCATGTATGTGGTGAGCTGCCTTTGTCTGGTGCTGAATGTGTGCGAGATGCTCCAC
ATCGGAATCGGCACTTTCCGGGACACCCTGCGCATGAAGAGGAACCGGGGCATGAGGACG
TCCTACGGCTACCCGTTTTCTCGAAACATCCCAGCCTCTCCTCCGGGGTACAACCTTGTG
ATGAAGACAGACAAACCCGGCAGGATCCCCAACAGCCTCATCACCCACGAGCAGAACATG
GCCAACGTGGCCCAGGAGCAGCAGTGCGCCAGCCCGGACGAGAACATCCCGTCTGATTTG
GCGAGCCTGCACCGGCACCTACGGGTGGCCCAGGAACAGCTCGATATGGCCTTTCAAACA
TATCAAACCAAAACCAACCAGCAAACATCGCGGACCAGTAGTCCTGTATCTGCGGGCACC
GTGGCAGAACAAAATCGGGTCAATACAGTCCAAGAGAAACAAGGAGCGAGGCCCAAATCT
GCCACAGAGAAGGCTGCCACCATCGTCAAGAATGGAAAGACCTCTGTTTGGATCTAG

>Ga-NN-cx43.4-G14294 Our modification. Underlined: Introns predicted by Ensembl are included as part of exon. Splice sites. At the first splice site, parts of the exons predicted by Ensembl have been removed

ATGAGTTGGAGCTTCCTCACTCGTCTGCTGGATGAGATCTCCAACCACTCCACCTTTGTG
GGGAAGATCTGGCTGACTATGCTCATCGTCTTCCGCATCGTGCTGACTGCCGTCGGCGGG
GAATCCATCTACTACGACGAGCAGAGTAAATTTGTCTGCAACACGCAGCAGCCCGGCTGC
GAGAACGTGTGCTACGACGCGTTTGCGCCGCTCTCGCATGTTCGATTCTGGATCTTTCAG
GTGATTCTGATCACCACCCCCACCATTATGTACCTGGGCTTTGCCATGCACAAGATCGCC
CGCACGGAGGACAGCGAGTACTGCCCCCCCCGGACCCAGAAGAAGAGGATACCCATCGTG
AGCCGGGGGGCAGTTCGGGACTACGAGGAGGCCGAAAACAACGGCGAGGAGGACCCCATG
ATCGCTGAAGAGGTCGAACTAGAGAAACTCAACAAGGCAGAAAAAAGCTCTGGGAAGAAG
CACGACGGCCGTCGGCGGATCATGCGCGACGGCCTGATGAAAGTCTATGTGTGCCAGCTG
CTTTGGCGCACCTCCTTCGAGGTGGCCTTCCTCTTTGGCCAGTATGTCCTCTACGGCTTT
GAGGTGATGGCCTCCTACGTCTGCACTCGCTCGCCGTGCCCGCACACTGTGGACTGCTTT
GTGTCGCGCCCCACCGAGAAGACCATCTTCCTGCTGGTGATGTACGTTGTGTCTTTCCTC
TGCCTGCTCCTCACAGTCTTTGAAATCATCCACTTGGGGGTCGGAGGCATCCAGGACACC
TTCCGCAGGCGGGCCACGCTCTGCTCTCGCACCCCCCCTCCGTCGTCCTCTCGCCCCGGG
CACGCTGCTCCGCCGGGATACCACGCCACCATGAAGAAGGAGAAACTGAAGGGAGAGCCG
AGGAACTCCCCAATGGGGGACTCTGGGCGGGAGAGTTTCAGGGACGAGTTGCCCTCATCC
AGGGAGCTGGAGCGGTTGAGGGGTCACCTGAAGCTGGCGCAGCAGCACCTGGACCTGGCC
TACCAGGCCGATGAGGGAAGCCCTTCCCGGAGCAGCAGCCCGGAGGGCAACGCGGCTGCG
CAGATGGCCGCCGAGCAGAACCGCCTCAACTTCGCCCAGGAAAAGCAGGGGGAAGCGAGC
GAGAAAGGTAACAAGCTTTTCGCAGGGATTTCAACTTAG

>Ga-cx43.4-G02384 Our modification. Underlined: Introns predicted by Ensembl are included as part of exon. cc (2 locations in the sequence): Both places, a third C has been removed to keep the reading frame. The number of Ns has been adjusted to fit reading frame and pattern expected in the second conserved domain.
ATGAGCTGGAGCTTCCTCACGCGTCTGCTGGACGAGATCTCCAACCACTCCACCTTCGTG
GGCAAAATCTGGCTCACCCTCCTCATCGTCTTCCGCATCGTGCTGACGGCCGTCGGGGGC
GAGTCCATCTACTACGATGAGCAGAGCAAGTTCGCGTGCAACACGCAGCAGCCCGGCTGC
GAGAACGTGTGCTACGACGCGTTTGCGCCGCTGTCGCACATCCGCTTCTGGGTGTTCCAG
GTGATCATGATCACCATCCCCACCATCATGTACCTCGGCTTCGCCATGCACAAGATCGCC
CGCATGGACGACAGCGACTACCGGCCCCGCAAGCGGATGCCGATAGTGAGCCGCGGCGCC
AACCGCGACTACGAGGAGGCGGAGGACAACGGCGAGGAGGACCCCATGATCCTGGAGGAG
ATTGAGCCGGAGAAGAAGGAGAAGGAGGCGCCGGAGAAGAAGCCGAGCAACAAGCACGAC
GGACGGCGGCGCATCAAGCGCGACGGCCTGATGAAGGTCTACGTGTTCCAGCTGCTGTCG
CGCGCCATCTGCGAGGCCTCGTCCTCNNNNNNNNNNNNNNNNNNNNNNNNNNNNNNNNNN
NNNNNNNNNNNNNNNNNNNNNNNNTTCGCCCTGCccGCACACGGTGAACTGCTTCGTGTCG
CGCCCCACAGAGAAGAccATCTTCCTGCGCATCATGTACGGCGTGAGCGCCCTGTGTCTG
CTCTTCACCCTGCTGGAGATCCTGCACCTCGGCATCAGCGGCATCCGGGACTGCTGTTGC
AGGCCGCGGACGCCGACCCCCCGGCGCACGGCCCCGCCCAGCCAGCGGTCCTCCATCAGC
CGGCAGCCGTCGGCGCCGCCGGGCTACCACACGGCGCTGAAGAAGTACCCGTCGGGGAAG
ATGGCCTTCCGGGACAACCTGGTGGACTCGGGGCGCGAGTCGCTGGGGGACGAGGCTTCG
TCGCGGGAGCTGGAGAGGCTGCGCAGGCACCTGAAGCTGGCCCAGCAGCATCTGGACCTG
GCCTACCAGAACGGGGAGAGCAGCCCGTCGCGCAGCAGCAGCCCCGAGTCCAACGGCACC
GCGGTGGAGCAGAACCGACTCAACTTTGCCCAGGAGAAGCAGAGTGCTACCTGCGAGAAA
GGTGAGGCTTCTTGA

>Ga-NN-gjd2*1-G05651 No modifications. Splice site.
ATGGGGGAGTGGACCATATTGGAGCGCCTCTTGGAGGCGGCTGTCCAACAACATTCTACA
ATGATCGGAAGGATCCTGCTGACCGTGGTCGTGATCTTCCGTATCTTGATCGTGGCCATC
GTGGGAGAGACCGTGTACAACGACGAGCAGTCCATGTTTGTCTGTAACACCTTACAGCCA
GGCTGCAACCAGGCATGCTACGACAAGGCGTTCCCAATCTCCCACATCAGGTACTGGGTG
TTCCAGACCATCATGGTGTGCTGCCCCATCCTCTGCTTCATCACTTACTCAGTGCACCAG
TCTACCAAGCAGAAGGACCGGCGCTACTCCACAGTTTTCCTCTCCTTGGACAAAGGCATG
GATTTTATGAGGAGAGACAACAGACGGCTCAAGAATACCATTGTGAACGGGATGCTACAG
AACACAGAAAACTCTAACAAGGAAGTAGAGCCGGACTACACTGAGGTAAAGGAAATTCAC
AACTCAGCCATGCAAACTACTAAGTCAAAGATGAGAAGGCAGGAGGGCATCTCCAGGTTC
TACATCATCCAGGTCGTGTTCAGAAACGCACTAGAGATAGGGTTTCTGGTGGGTCAATAC
GTCCTGTACGGATTCTATGTCCCTGGGGTGTATGAATGTGATCGATACCCTTGCATGAAA
GATGTAGAGTGCTATGTTTCACGGCCAACAGAGAAAACAGTGTTTCTCGTCTTCATGTTT
GCGGTCAGTGGTATTTGTGTACTGCTGAACTTGGCAGAGCTCAATCATCTTGGCTGGACA
AAGATAAAAACTGCTGTCAGAGGAGTGCAGGCTAGGAGGAAGTCCACTTATGAGATCCGG
AACAAAGACTGCCCAAGGATGAGTATGCCCAATCTTGGGCACACCCATTCAGGTGACTCT
GCATATGTGTAA

>Ga-gjd1a-G20357 Our modification. Underlined: Intron predicted by Ensembl is included as part of exon. Splice site.
ATGGGGGAATGGACCATTTTGGAGAGGCTGCTGGAGGCGGCTGTCCAGCAGCACTCGACC
ATGATCGGAAGGATCCTGCTGACAGTGGTGGTGATTTTCCGTATTCTAATAGTAGGCATA
GTGGGGGAGAAGGTGTACGAGGACGAGCAAATCATGTTCATCTGTAACACCATGCAGCCC
GGCTGCAACCAGGCTTGTTACGACAAGGCCTTCCCCATCTCGCACATCCGCTACTGGGTC
TTTCAGATAATCCTGGTGTGCACGCCGAGCCTGTGCTTCATCACATATTCTGTTCACCAG
TCTGCCAAAGCGCGTGACCGAAGCTACTCTCTCCTGCATCCTTACATGGACAGCCACGGC
CACGGTGGCCACCACGGGCGCCATCACGACCACCACGCCCGCAAGCTTCACTCTCGCAAC
ATCAACGGCATTCTGGTGCACCCCGACAGCAGCAAGGAGGATCACGACTGCCTGGAGGTC
AAGGAGATCCCCAACGGACCCCGGGGACTCCCGCAAACACACAAGAATGCTAAAGTGCGC
CGGCAGGAAGGCATCTCCCGTTTCTACGTCATCCAGGTGGTGTTCCGCAACGCACTGGAG
ATCGGCTTCCTGGCCGGCCAGTACTTCCTGTACGGCTTCAACGTGCCAGGGATGTTTGAG
TGTGATCGCTACCCGTGCGTGAAGGAGGTGGAGTGTTACGTGTCTCGACCCACGGAAAAG
ACCGTGTTTCTGGTCTTCATGTTTGCCGTGAGCGGCGTTTGTGTGCTGCTCAACCTGGCT
GAGCTCAACCACCTCGGCTGGAGGAAGATAAAGACGGCCATCCGAGGGGTGCAGGCCAGA
AGGAAGTCAATCTGTGAGGTCCGCAAGAAGGATGTTTCACACCTGTCCCAGACCCCAAAC
CTGGGCAGGACGCAGTCTAGTGAGTCAGCCTACGTCTGA

>Ga-gjd2b-G10416 Our modification. Underlined: Intron predicted by Ensembl is included as part of exon. Splice site.
ATGGGGGAATGGACTATACTAGAGAGGCTCCTGGAGGCTGCTGTCCAGCAGCACTCTACT
ATGATAGGAAGGATCCTACTAACAGTGGTGGTCATCTTCCGGATTCTAATCGTAGCAATA
GTTGGAGAGACTGTCTATGATGACGAGCAGACCATGTTTGTTTGTAACACCTTACAACCG

GGCTGCAACCAGGCATGCTACGACAAGGCATTCCCCATTTCACACATTAGATATTGGGTT
TTTCAAATCATCATGGTGTGCACCCCGAGCCTTTGTTTTATCACGTACTCGGTGCATCAA
TCGGCCAAGCAGAAGGAGCGGCGGTACTCAACAGTCTATCTGACGCTAGATAAGGATCAA
GATTCACTGAAGCGAGACGAGAGCAAAAAGATAAAGAACACCATTGTTAACGGAGTACTT
CAGAACACAGAGAACTCCACCAAAGAAGCCGAGCCGGACTGTTTAGAAGTCAAGGAAATC
CCAAATTCGGCAATGAGAACTACAAAGTCCAAAATGAGGCGACAAGAGGGCATCTCCCGC
TTTTACATCATCCAGGTGGTTTTCAGAAACGCGCTGGAGATCGGCTTCTTGGTGGGTCAG
TACTTCTTGTACGGATTCAACGTCCCGTCGGTGTATGAATGTGATCGCTACCCCTGCATT
AAAGATGTCGAGTGCTACGTCTCCAGACCTACGGAGAAGACCGTGTTCCTGGTCTTCATG
TTCGCGGTCAGCGGCTTTTGCGTGGTGTTGAACCTGGCGGAACTCAATCATTTGGGCTGG
AGGAAAATCAAAACCGCCGTGCGCGGTGTGCAGGCTCGGCGGAAGTCCATTTATGAGATC
AGAAATAAGGACTTGCCGAGGATGAGTGTGCCTAATTTCGGCCGCACTCAGTCCAGTGAC
TCTGCTTATGTGTAA

>Ga-NN-gjd2*2-G05764 Our modification. Underlined: Intron predicted by Ensembl is included as part of exon. Splice site.
ATGGGAGAATGGACCATCCTGGAGCGCCTCCTGGAGGCCGCCGTGCAGCAGCACTCGACT
ATGATCGGAAGGATCCTGCTGACTGTAGTGGTCATCTTCCGCATCCTGATCGTGGCCATC
GTCGGCGAGACCGTCTATGAGGACGAGCAGACCATGTTCGTCTGCAACACCCTGCAGCCA
GGCTGCAACCAGGCCTGCTACGACAAGGCCTTCCCCATCTCCCACATCCGCTACTGGGTC
TTCCAGATCATCCTGGTGTGCACGCCCAGCCTCTGCTTCATCACCTACTCCGTCCACCAG
TCGGCCAAGCAGAAAGACCGCCGCTACTCCTTCCTCTACCCCATCATGGAGAGGGACTAC
GGGGGAAGGGAAGGGACGCGGAAGATCCGCAACATCAACGGGATTCTGGTGCAGCACGGC
GGCGGCGGCGACGGCGGCGGCGGGGGGAAGGAGGAAGCCGACTGCCTGGAGGTGAAGGAG
ATCCCCAACGCGCCGCGGGGCCTCACCCACGGGAAGAGCTCCAAGGTTCGCCGGCAGGAA
GGGATCTCCCGCTTTTACATCATCCAGGTGGTGTTCAGAAACGCGCTGGAGATCGGCTTC
CTGGCGGGCCAGTACTTCCTGTACGGCTTCAGCGTGCCGGGGATTTTCGAGTGCGACCGC
TACCCGTGTCTGAAGGAGGTGGAGTGCTACGTGTCCCGTCCCACGGAGAAAACGGTTTTC
CTGGTCTTCATGTTCGCGGTGAGCGGCATCTGCGTGGTGCTCAACCTGGCGGAGCTCAAC
CACCTGGGCTGGCGCAAGATCAAGGCCGCCATCAGGGGCGTGCAGGCCCGCAGGAAGTCC
ATCTGCGAGATCCGGAAGAAGGACATGGCGCATCTGTCCCAGCCGCCCAACCTGGGCCGC
ACGCAGTCCAGCGAGTCCGCCTACGTCTGA

>Ga-GJD3-G08497 Our modification. Underlined: Exon extended until stop codon.
ATGGGGGAATGGAGCTTCCTCAGCGAGCTCTTCGACAGCCTCCGGGCCTACTCCACCATG
CTCGGCCGTTTCTGGCTCCTGGTCACCGTCATCTTCCGGATGCTGATCCTGGGAACCGTG
GCGAGCGACCTGTTTGAAGACGAGCAGGAGGAGTTCACCTGCAACACCCTGCAGCCGGGC
TGCAAGCAGGTGTGCTACGACATGGCCTTCCCCATCTCCCAGTACAGGTTCTGGGTGTTC
AACATCGTCCTCATCGCCACGCCAGCCCTGGTCTTCCTCGTGTACGCCGTGCATCACCAC
AACAAGAGGGCCGACGGCGGCCAAAGCAGCGGCCGGGACGACCTGGCAGATCTCCACTTG
AGGAAGTTCTACGTCGTCAACGTGGTCTTTCGCATACTGGCGGAGGTGGGCTTCCTCGTG
TTCCAGTGGACGCTGTACGGCTTCACGGTGGAGGCCCACTTCCCCTGCAGCCGCTTCCCC
TGCCCCCACGTGGTGGACTGCTTCACCTCCCGCCCCTCGGAGAAAACCGTGTTCCTCCGC
TTCTACTTCGGGGTGGGGCTGGTGTCGGCGGCCTCCAGCTGCGCCGAGCTTTTCTACAGC
TCCGTGAAGTGGTTCTGCTGCTCGAGGAGGCCGCTCTTCGCCCCGGACCGCCGCCACCAC
GACGACGACGACGAGGAGGCGGGGGGCGGCGGCGGCGGCGGGTCGGAGGAGAAACCGCGA
GGGGGCGCGGGAAGGGGCGGTGGCGCGAGCCACAAGGGAGGAGCGCGAGCGGGAAGGTCT
CCGTCCGCGAGCAGAGTGAGGAAGGGCGGGAAGTACGCGGCGGCGGGGCGCTCGTGGCGT
GAGGCCGCCCTGCTTTCGAAGCAGGCAGCGGGCGCTCAGGGTCACATGAGTGACTGA

>Ga-cx36.7-G14369 Our modification. Underlined: Introns predicted by Ensembl are included as part of exon.
ATGACGGAGTGGACACTGCTCAAGAGACTCCTGGACGCTGTGCACCAACACTCCACCATG
ATCGGTCGCCTGTGGCTCACCGTCATGGTCATTTTCCGGCTGCTCATCGTTGCTGTGGCC
ACTGAGGACGTGTACACCGACGAACAGGAGATGTTTGTGTGCAACACGCTGCAGCCGGGA
TGCTCCACCGTCTGCTACGACGCCTTTGCGCCAATCTCGCAACCTCGCTTCTGGGTCTTC
CACATCATCAGCGTCTCCACGCCATCCCTCTGCTTCATCATCTACACGTGGCACAACCTG
TCCAAGCTGCCCCACAATGGCACCCAGAGGCAACGGCTGGGACAAGGAGGTATCCCAAGG
CCGGGAGGCCCGGATGTCGGGCAAGGCAGTGGACGCGAGGTGTACGATCGGAGCTGTGAC
TCAGACAGCTGCTCAATTCGCTCCCATAAGCACTTGGGTCACAGTTTGGCGGATGTGCTA
GAGGGCGTTACAGCTCACAACCTCCGGAGGGGAGACCACAACAAAGCGATGTCCTTGAGT
CCTGCTCGAGGTTACGCCGTCCTAGAGGCGACTCAAGAGGCCTCTGGAGGCGTTCTGTCT
AAATGTTACATCTTCCATGTGTGTTTGCGGGCTGTTCTGGAGGTGAGTTTTGTGGTGGCC
CAGTGGAAGCTGTTTGGCTTCCAGGTACCTGTCCATTTCCTTTGTACGTCTGTCCCCTGC
AGCCAGCCGGTGGACTGCTACGTCTCCAGGCCCACGGAGAAGACCATTTTCTTGCTCTTC
ATGTTTTGTGTGGGGATTTTCTGCATCCTGCTCAACCTGCTGGAGCTCAACCACTTGGGC
TGGAAGAAGATCAGACAGTCTGTGAGGCTGAAGGAGAGGGCGTCCTGGGGAGGCTGTCCA
GGTATGAAAGGGGGGTATGAAACCTTCCCCCCAGACAGCCCCTGTCTCACAACCTCGTTA

GGCTACAGGGACGTGACCAGCACCACTTCCCTGCCCACTTTGGACCTGGTGGTGGGGCAC
CAGCCTGACTGGACCTGCGCTGTGAACTGTGGCAGGATGAGGGAGCATGAGGAGGTCAGA
GAGACGAGACCAGATCAAACAAAGTCACAGGACTCCCATAAAGGAGAGAGGCAGCCTCTG
AAGAGTAAGACGGCGATCAGAGAGTACAAACAGAGGAGTGCTGAGGTCTGGATATAG

>Ga-NP-cx39.2 Not predicted by Ensembl
ATGGGAGACTGGTCCATTCTTGGCCGCTTCCTAACGGAAGTCCAGAACCATTCCACGGTC
ATCGGCAAGATATGGCTGACTATGCTGCTAATCTTCCGCATCTTGCTTGTGGCTCTGGTG
GGGGATGCTGTCTACAGCGACGAGCAGTCCAAGTTTACCTGCAACACCCTGCAGCCCGGC
TGCAACAACGTCTGCTACGACACCTTCGCCCCCGTCTCACACCTGCGCTTTTGGGTCTTT
CAAATTGTGCTGGTCTCCACACCCTCTATCTTCTACATCGTCTACGTCTTGCAAAACATT
ACCAAACATGAAAAACTGGAGGACAGGAAGCTTCAAGTGCTGCCCGGGGCCTCACTTTCA
CTTGAACGGGACAAACACACTGTTAGAGAACAAGAGAGCACACTGGAGGCCCGCAGTCCT
CGTGAAGGGGAATGTGATGGTAAGAGCCAACTGGAGGAGGATGCGAGAGAGGTAGAAAAG
GACCCGACCCAGCTCTCCAGCCAAGTGCTACTTATCTACATCATCCACGTTTTTCTGCGC
TCCATCATGGAGATAATCTTTCTCGTTGGACAGTACTGCCTCTTCGGATTTGAGGTCCCA
AACCTTTTCCGCTGTGAGACCTACCCATGCCCAAACAGGACCGACTGCTTTGTGTCCCGA
GCAACAGAGAAGACCATCTTTCTCAACTTCATGTTCAGCGTCAGTCTAGGCTGCTTTGTC
TTGAACATTGTGGAGCTGCATTATGTGGGCTGGATTTACATTTTCAGGGTGTTGTTCTCT
GCATGCTGTCCGTGCTGTGAAACAGGTAGAAACCCTGGGCAGCAGGGGGACTTGTACTCT
GACAACAGGCAGGGTTGCCCTGCAGACCACCTTGTTGACCCGTGTTGA

>Ga-NN-cx39.2-G00420 Our modification. Underlined: Extension of exon to reach initiation codon. N: One nucleotide added to keep reading frame.
ATGGGTGACTGGTCCATACTTGGTCGCTTCCTGTCAGAAGTCCAAAACCACTCTACAGTG
ATAGGCAAGATCTGGCTCACCATGCGGCTGATTTTCCGTATTCTGCTCGTGGCCTTAGTG
GGTGACGCTGTCTACAGTGATGAGCAGTCNACTCATGTTACATTCACATGTAACACCCAG
CAGCCTGGATGTAACAACGTCTGTTATGACACTTTTGCTCCTGTTTTGCATCTTCGGTTT
TGGGTCTTCCAAATTGTGCTGGTCTCCACTCCATCCATCTTCTACATAGTTTTTATTCTA
CATAAGATTGCCAAAGATGAAAAGCTGAATGGCCAAAGGGCACAGATGGTAGCCCAGAGG
TATCCCCAATCGAGATGTGGGCGCATTGGGAAGGATGGCATGGAGGTCTTAAAGGTTGAC
ATGCCCACCTACTGTCATTACAGGGAAGATTGGGATGCAAAAGAGAGAGAGAAGGAGTAGAG
CAAAGCTCTCTGGAGGAGGATGGTGGCAAAGTAGGAGAGGACCCTACGCAGCAGTCCAAT
CGGGTTCTGCTCATCTACATTCTTCATGTGTTACTACGATCAGTCATGGAGATTACCTTT
CTGGTGGGCCAATTCTTCTTGTTCGGGTTCACGGTGCCTCAACTGTACCGCTGTGAGACC
TACCCTTGTCCCACACGTACAGACTGCTTTGTGTCGCGTGCCACTGAGAAAACCATCTTC
TTGAACTTCATGTTCAGTATAGGTTTTAGCTGCTTTCTACTCAACATAGCAGAGCTCCAC
TACCTTGGCTGGGTCTACATTTTCCGCATCCTCTGCTCGGCCTGTTTCACCTGCTGCAGT
CATGAGAGGGACACTATCAGACTCTACTCAAACCACAACGCCCTCCTGCTGCAGCTAAGG
CATTCTCTTAGGAGCCAGTTGGCTCTGTAG

>Ga-NP-gjd4(1) Not predicted by Ensembl. Splice site
ATGACGGGAATGAGTGGCTCTGAGGTCATCTTCATCTCTGTCAATCACAACATCACTTTG
ATGGGCAAGGTTTGGCTCGTCGTGATGATCTTCCTCCGTATCCTGATCCTCCTCCTGGCT
GGTTATCCTCTCTACCAGGACGAGCAGGAGCGATTCGTGTGTAACACCATTCAGCCCGGC
TGTGCCAACGTGTGCTATGATCTGTTTTCTCCCATCTCACTCTTCCGCTTCTGGCTGGTG
CAGCTCGTCACCTTGTGTCTCCCCTACTTCATCTTCATTATCTACGTGGTCCACAAGGTC
TCGAAGGGCCTCACAGTGGACCCGTACCCCTCGGGCCGCACCAAAGCCCCACCTTTGCTC
GAGAACCACCAGGAGCCGTTCACCAAGACATCTGTAAACAAGACGGCTGAGCACAGGGGG
GCTCGGTGCTTCACGGGAGCCTACATCCTCCATCTGTTGTTCAGAACGTTGCTGGAGGCA
GGGTTTGGGGCAGCTCACTACTATCTGTTTGGTTTCCACATCCCGAGGAGGTTCCTGTGC
CAGCATCCGCCGTGCACCACCCAGGTGGACTGCTACATCTCTAGGCCCACTGAAAAGACC
GTGATGCTCAACTTCATGCTCGGCGCGGCTGCTTTGTCCCTTTTCCTCAACGTGCTGGAT
TTCTTCTGTGCCATCAAGCGGTCGGTGAAACAGAAAAGCAAGAGCAGGATGACGGTAGTG
GAGAAGATATATGAGGAAGAGCAATGTTTCCTTTCGGCTAAGCAGGAGAAAGAAGGGGCT
GGTCTCCCGGGGAGCTTCCGGAAAAGGCGAGGCAGCAAGGGCTCTAGTGCAGGGCTTGCT
TTAGGTCAGGAAACACCCGGCATGGAGCGCTCTTCTCTTCCACACTCTCCAGGACATCCT
GGCTGCAACACCAACGGAAACAACGGCTACTCTGTTTCCCAGGAGGAAGCTCTGGAAAGG
AATGGCAGCGAGGTGGCTCTTTGCCCCCCAGAGGCGATGAGGACACCTAGATCAATCCGT
GTTAGCAAACGGAGTCGACACAAACCCCCACCTCCGCCCAGACGGGACCTCGGCGCGCCC
CCCAGGGAGCCAGCGGTTCCCTTCGGAGACGTTTCCACAGCAATTTGTACCAGACGTGTG
GGTCAGTACACGCTGGTTGAACTGGGTAGCGGCGCAGAGCCACAGACCAATGATGAAGAG
ATGAGATCTGAGTGGGTGTGA

>Ga-NP-gjd4(2) Not predicted by Ensembl. Exon 1 not found; Splice site
NGCAAAACGTGGTGGATGTTAATGCTGCCCCTCCGCCTGCTGGTCCTCCTGCTGGCCGGC
TCCACCCTCTTCAGCGACGAGCTGGATCGCTTCACCTGCAACACCGTCCAGCCGTGCTGC
TCCACCGTGTGCTTCGACGCCTTCTCCCCCGTGTCCGCCTTCCGCCTCTGGCTCTTCCAC

CTCGTCCTGCTGTGCGTCCCCGACGCGCTGTTCGCCACCTACGTCGTGCACAAAGTGGCG
TCGCCTCCCCACGGAGGGTTCTGCTGCGACGGCGGTCGAGGAGGGTCCCCCGTCGCCCTC
GGGGACTCCGGCTCCTCGAGGCTTCCCCGCCTGCGGCCTGCGCGGGAAGCGCCGCGCTTC
CACTGCGCTTACTTCCTGGCTGTGATGCTGCGCGTCCTGCTGGAGGTGGTTTTCGCCGGC
GGGCAGTTCTTCCTCTTCGGTTCGTCCGTTCCCCGGAGCTTCCGCTGCCACGAGGCTCCC
TGCACATTTGGCGTGGAGTGCTACGTCTCCAGACCCACGGAGAAGACCATGATGCTCCAT
CTCATGCTGGGACTGGCCTCCCTGTCCGTCCTGCTGGGTCTGGCGGACCTGGCGACCTCC
ACAAAGGCCGCGGTGACCTGGAGGAGGAGGAGGAGGAGGAGGAGGGAGGCGTCGACGGAA
GAGAAGAGCGCCGCGCGTTCTACGACAACCTCAACGGAGGACAGCGGAGTCCTTTCGACC
AGAAGACTCGGCCTCGCGGTGGCGACGCCTCGGACCCCCAGTCCCTTTGGCACTCCAGTG
CCCGCCCACTTCGTCCTCCACAGCCGGCTGAGACCTCCCCTGCCCCCCCGCCCCGACAGA
GGGCCGAACCCCAGGACGCCGACACCCAACGGGTGGGAAAAAGCCGGACCGGGACACTTC
AGTCGGGGCGAACTGGGGCCAACAGTCTGA

>Ga-GJE1-G13559 No modifications. Splice sites.
ATGTCTCTGAACTACATCCGAAACTTCTACGAAGGCTGCCTCAGGCCTCCCACGGTGATC
GGACAGTTCCACACCTTGTTCTTCGGCTCGGTGCGGATGTTCTTCCTCGGCGTCCTCGGC
TTCGCTGTCTACGGAAACGAGGCGCTGCACTTCAGCTGCGACCCGGATCGCCGAGAGCTC
AACCTGTACTGCTACAACCAGTTCAGACCCATAACCCCCCAGGTTTTTGGGCGTTACAA
CTGGTGACGGTGCTGGTCCCGGGCGCGGTCTTTCACCTCTACGCAGCCTGTAAGAACATC
GACCAGGAAGAGATCCTGGAGCGCCCCCTCTACACCGTGTACTACATCATTTCTGTTCTT
CTACGCATCATCCTGGAAGTCATCGCCTTCTGGCTGCAAAGTCACCTCTTTGGCTTCCAG
GTCCACCCGGTGTTCATGTGTGACGCCAGTTCTCTGGAAAAGACCTTTAACGTGACGAGG
TGCATGGTGCCAGAACACTTTGAGAAAACCATCTTCCTCAGTGCCATGTACACCTTCACC
GTCATCACCATTCTCCTCTGTGTCGCCGAGATATTCGAGATACTGTGCCGACGTCTCGGT
TACCTCAACAACCAGTGA

Suppl. Fig. 9. Atlantic herring (*Clupea harengus*) connexins.

Yellow: Conserved domains as defined by Cruciani and Mikalsen (2007)
Green: Conserved cysteine codons (cysteine signature)
Grey: 15 nt added at the ends of the conserved domains
Other colors are explained where necessary.

```
>Ch-gja1-cx43-XM_012829211
ATGGGTGACTGGAGTGCTTTGGGAAGACTCCTGGACAAAGTCCAGGCTTACTCCACGGCC
GGTGGAAAAGTCTGGCTCTCCGTGCTTTTCATCTTTCGCATCCTGGTGCTGGGGACGGCA
GTGGAGTCGGCGTGGGGCGATGAACAGTCGGCCTTCAAATGCAACACGCAGCAGCCCGGT
TGCGAGAACGTCTGCTACGACAAGTCCTTTCCTATCTCGCACGTGCGCTACTGGGTGCTA
CAGATTATCTTCGTGTCCACGCCCACGCTGCTCTACCTGGCTCACGTCTTCTACCTGATG
CGCAAGGAGGAGAAGCTCAACCGCAAGAAAGAGGAGCTGAAGTTGGTGGGCAACGACGGT
GGTGACGTGGAGATCCCGCTGCAGAAGATCGAGATGAAGAAGCTCAAGCACGGGCTGCAG
GAGCACGGCAAGGTCAAGATGAAGGGCGCCCTCTTGCGCACCTACATCTTTAGCATCCTC
TTCAAGTCGCTCTTCGAGGTGGGCTTCCTGGTCATCCAGTGGTACTTGTACGGCTTCACG
CTTGCGGCAGTGTACACTTGCGAGCGCGACCCCTGCCCGCACCGCGTGGACTGCTTTCTC
TCCCGGCCCACGGAGAAGACTGTCTTCATCATCTTCATGCTGGTGGTTTCGCTGGTGTCC
CTGGGGCTCAACGTAGTCGAGCTGTTCTACGTCTTCTACAAGCGTATCAAGGACCGCGTC
AAGGGGAATCAGGGTAACCTCTACCCCATCGCTGGCACCATGAGCACCACGCCCAAGGAC
ATGTCCACCACCAAGTACGCCTACTACAACGGATGCTCATCGCCCACCGCCCCACTCTCC
CCCATGTCACCGCCAGGCTACAAGCTGGCCACGGGGGAGAGGACCAACTCCTGTCGCATT
TATAACAAGCAGGCCAACGAGCAGAACTGGGCCAACTACAGCACGGAGCAGAACCGGCTG
GGCCAGAACGGCAGCACCATCTCCAACTCGCATGCCCAGGCCTTTGACTTCCCAGATGGC
ACCCAGGAGCACCAGAAACTGCCGCCGGGCCACGAGCTTCAGCCACTGGCGCTCCTGGAC
CCAAGGCCTTGCAGCCGGGCCAGCAGCCGCATCAGCAGCCGACCACGGCCGGACGATCTA
GACGTCTAG

>Ch-gja1like-XM_012836783
ATGGGCGACTGGAGTGCTTTAGGGAAACTTCTTGACAAGGTCCAGGCGTACTCCACAGCG
GGAGGCAAGGTCTGGCTCTCTGTCCTCTTCATCTTCCGGATACTGGTGCTGGGAACGGCG
GTGGAATCCGCGTGGGGTGACGAACAGTCGGCCTTCAAGTGCAACACGCAGCAGCCCGGC
TGTGAGAACGTCTGTTACGACAAGTCGTTCCCCATCTCTCACGTGCGCTTCTGGGTGCTA
CAGATCATTTTCGTTTCCATACCAACTCTCCTTTACCTCAGTCACGTCCTCTACCTCATA
CACAAGGAGGATAAACTCCTCAAGAAAGAGGAGAACCTGAGAGCGATTCAGAGTCAAGGT
GGAGATGTGGACGTGCTACTTCAGAAAATTCAGCAGAGGAAGTTCAAGTATGGGCTGGAG
CAGCATGGGAAGATTAAAATGAGAGGGGGCCTTCTATACACTTACATTTTGAGCATTATT
CTCAAGTCTGTTTTTGAGGTGGCCTTCTTGCTGATGCAGTGGTATATTTACGGATTCCAC
CTCTCTGCCATCTACACTTGTGAAAGGGTTCCCTGTCCTCACCAGGTTGACTGTTTCCTC
TCCCGCCCGACTGAGAAGACCATCTTCATAATCTTCATGTTGGTAGTTTCGCTAGTCTCT
CTAGGTCTTAATGTCATTGAGTTCTTTTATGTAATATATAAGAGAATGAAAGATAAGGTA
AAGGAGAAGGCCGCAAATCAACTGCACCATAACGTTCACCTGAAGCCTTGCATGGGAGAG
ATGCCGCCCTCTAGTTATGTCTACTACAACGACTGCTCGGCCCCCATGTCTAACCTAGAG
TACAACCTAAACACGGCAGACAGGAACAACTCTTGCGAGAGCTACAGCAAGCAGGCCAAG
GCTCAGAACTGGACCAATTACAGTACGGAGCAGAACCAGCTGGGCCGGAAAGTGCCCACG
TACCCCTGCTGCCACACCCAGGATTTTCACTACCCAGAAAAAGTGACACCGGGTACGGAC
ATTGCTCTCCTCAAGCAGCTTGACCCTCGACCCAGTAGCCGGGCGAGCAGTCGAGCGAGG
CCAGATGATCTTGACATCTAG

>Ch-gja3like-XM_012842347
ATGGGTGACTGGAGTTTTCTGGGGCGGCTGTTGGAGAATGCGCAGGAACACTCAACGGTG
ATCGGCAAGGTCTGGCTGACTGTCCTCTTCATCTTTAGGATCCTGGTGCTGGGGGCCGCT
GCTGAGGAGGTGTGGGGGGACGAGCAGTCGGACTTCACCTGCAACACGCAGCAGCCCGGT
TGCGAGAACGTGTGCTACGACGAGGCCTTCCCCATCTCACACATCCGCTTCTGGGTGCTA
CAAATCATCTTCGTGTCCACGCCCACTCTCATATACCTGGGCCATGTACTGCACATTGTT
CGTATGGAGGAGAAGCGGAAAGAGAAGGAGGAGGAACTGCGAAAGGCCACTAGACTCCAG
GAGGAGAAAGAACTCCTTTACAGAAACGGAGGGGGAGGGGGGGCGAGAGGAGGGGGTGGC
AGACCTGTGAAAAAGGAGAAGCCACCAATCAGAGACGAGCACGGGAAAATCCGTATTAGG
GGTGCGCTGTTACGCACCTATGTGTTCAACATCATATTTAAAACCCTTTTTGAGGTGGGG
TTTATTTTAGGTCAGTATTTCCTCTATGGCTTTCAGCTGACGCCCCTGTATAAGTGTGCG
CGGTGGCCTTGCCCCAACATCGTGGACTGCTTCATCTCCAGACCCACAGAAAAGACCATC
TTCATCATATTTATGCTTGTGGTGGCTTGCGTGTCTCTTTTGCTGAATTTGTTAGAGATT
TATCACCTTGGATGGAAAAAAGTCAAGCAAGGCATGATTACGAATTACGACCACGAGTTA
CTACCACTCAGGGAGGGTGCTGGGCCCGAGCCTGTGAGCTCTGGTCCCAGGACTGCTCCT
CCCACCCTTAGCTACCCGCCGACCTACACAGACGTGACGTCAGGCAACTCGGCCTTCTTG
```

CAGCCTGCGGTGGCGCCACCCTCGGCCGACTTCGCTATGGATCCGCTCCACGAGGAGCTG
CGCGAGCCCTCGCCTTTCTACATCAGCAACAACAACAACCACAGGCTGGCCGCTGAGCAG
AACTGGGCCAACTTGGCCACCGAGCAGCAGACTCGGGAGATGAAGGCCACCTCCCCTTCC
CCCTCCTCCTCCGCCTCCGCCTCCTCGTCCTCCTCCTCCTCCTCCTCCTCTTCTGCTGAC
CACGAGCGGCAGCCCAAAGAGGCGGCCCTGCCCACCACCAGCCCCAGCTCGAGCGGCGGC
GGCAGCTTGAGTGACGGCAAGAGTGAGCCGGAAGAAGGCCACGTCACCACCATGGTGGAG
ATGCACGAGCCGCCCCTCACGTTCACAGACCCGCGCCGGCTCAGCAGAGCCAGCAAGACC
AGCAGTGTTAGAGCCAGGCCCAACGACCTGGCAGTTTAG

>Ch-gja3like-XM_012840585
ATGGGTGACTGGAGCTTTCTGGGGCGTCTGTTGGAGAACGCGCAGGAGCACTCAACAGTG
ATCGGCAAGGTGTGGCTGACCGTCCTCTTCATCTTCAGGATCCTGGTGCTGGGGGCCGCC
GCTGAGGAGGTGTGGGGGGACGAGCAGTCGGACTTCACCTGCAACACACAGCAGCCCGGT
TGCGAGAACGTCTGCTATGACGAGGCCTTCCCCATCTCGCACATCCGTTTCTGGGTGCTG
CAGATCATCTTCGTGTCCACGCCGACGCTCATCTACCTGGGCCACGTCCTGCACATCGTC
CGCATGGAGGAGAAAAGGAAGGAGAAGGAGGAGGAGATGCGCAAGGCACTCCGCTTCCAG
GAGGAGAAGGATCTCTACCGGAATGGTGGGGAGGGTGGAGGAGGGGGTGGTGGAGGTGGG
AAAAAGGAGAAGCCCCCAATTCGAGACGAGCATGGAAAGATCCGGATCCGGGGGGCCCTG
CTCCGCACCTACATCTTCAACATCATCTTCAAAACCCTGTTTGAGGTGGGCTTCATCCTG
GGTCAGTACTTCCTCTACGGGTTCCAGCTCAGGCCCCTGTATAAGTGTGCCCGATGGCCC
TGCCCAAACTCAGTGGACTGCTTCATCTCCAGGCCCACGGAAAAGACCATTTTTATCATT
TTCATGCTTGTGGTGGCTTGCGTGTCCCTTTTGTTGAACCTCTTAGAGATCTACCACCTG
GGCTGGAAGAAAGTCAAGCAGGGCATGACCAACGAGTTTGCGCCGGAGTGGTCAATGGGC
AGGGAGGCAGATAACATGACGGTCCTGCCCGAGACCAGAACATCCGCTCCCCTCCCCAAC
CCACCCAACTACACAGACCTGACGGTCGCAGGGAGAGGTGCCTTCATGCCCCAATCCCAC
TCCATGGCCATAGTAGCCACTCCTGAGGCAGAGCGCAAGTCAGACCCTCTCCATGACCCC
CTCCACTCCTCCATCTTCTCCAGCAACAACAGCAACAACTTCCGGCTACCCACGGGGCAG
AATTGGGCCAACATGGCGGCCGAGCTGCAGACTAGCGAAGCGAAGCCGAGCGCCCCTTGC
TTGTCCTCCTCCTCCTCCTCGTGCTCTCTGAGTAACATGGCGCACCCCAAGGAGCCCCCG
TTGGTTTCCTCCGGCGCGGTGACCGACGGCGATGCTGATGGCGATGAATCCAGTGGAGGG
AAGAGTGACGTGGGTCGCGACATCACGACCACCACGGTGGAGATGCACGAACCACCGGTG
GTCCCGATTGATGTCCGTAGGCTAAGCCGGGTGAGCAAGACCAGCAGCATCAGAGCCAGG
CCTGATGACCTCGCTGTCTAG

>Ch-gja3like-cx39.9-XM_012834366
ATGGGAGACTGGAGCTTACTGGGGAAGCTTCTGGAGAGTGCCCAGGAGCACTCCACCGTG
GTGGGCAAGGTCTGGCTGACGGTCCTTTTCATCTTCCGCATCCTAGTGCTGGGTACGGCC
GCCGAGAAGGTGTGGGGGGACGAGCAGTCGGGCTTCACCTGCGACACCAAGCAACCCGGT
TGCCAGAACGTCTGCTACGACAAGACCTTCCCCATCTCGCACATCCGCTTCTGGGTACTG
CAGATCATCTTCGTCTCCACGCCCACGCTCATCTACCTGGGCCACATCCTGCACCTGGTG
CGCATGGAGGAGAAGCAGAAGCTCAGGGAGAAACAGCTACTGAACCATCAACACGCTACG
GACAATCAGCTGGTCATTGCTGACGTGAAGACCAAGAAGGCGCCCGTGCGAGACGAGCAG
GGTCACATCCGCCTGCAGGGCGACATCCTGCGCACCTATATCTTCAACATCATCTGCAAA
ACCTTCTTCGAGGTGGGCTTCATTGTGGGCCAGTACATCCTGTACGGCTTCGAGCTGAAG
CCCCTCTACACGTGCGACAGGCCCCCATGCCCTAACCGCGTGAACTGCTACATCTCTCGG
CCCACAGAAAAGACCATCTTTATCCTCTTCATGCTAGGTGTAGCCTGCTTGTCGCTGCTG
CTCAACCTGGTGGAGATGTACCACTTGGGCTTCACCAAGTGCAAACAGGGACTCCGTTAC
CGACAGGTGGAACTTGCATCGGAGGCACCCTCCAAGACGCCAAGTGAGGCTACAGCTGTA
CCTTTCGTGCCCAGCTACAGTTACTACCACCCAGACCCTACGCCCTACCCTCCTGCCCCG
GGGTACAGCATCACGCCTATGAATGGATCGGACTCATCCTTCCACCCATACAACAGCAAG
GCGGCATACAAGCAAAACAAGGACAACCTTGCCATGGAGAGAAATAGCAAACCTGAGGAA
TGCGACCTGAAAACGAAAAAGGGTTCTGGGTCCGTCCCGGGATCGCCCGCTTCCATCCGT
CAGGGCAAGTCAACTCGAACCCCTCGGCCCACCCCCCACAAGACCAGAATAGATGACCTC
AAAATCTGA

>Ch-gja3like-cx39.9-XM_012819598
ATGGGGGACTTTAGTTCATTGGGAAAGCTGCTGGAGAATGCCCAGGAGCACAGCACCGTG
GTGGGGAAGGTCTGGCTCACCGTCCTCTTCATCTTCCGCATCCTGGTGCTCAGTGCCGCC
GCCGAGAAGGTGTGGGGCGACGAGATGTCGGGCTTCACCTGCGACACCAAGCAGCCCGGT
TGCCAGAACGTCTGCTACGACATAACCTTTCCCATCTCGCACATCCGCTTCTGGGTGCTG
CAGATCATCTTCATCTCCACGCCCACGCTCATCTACCTGGGCCACATCCTGCACCTGGTG
CGCATGGAGGAGAAGCACAAGCAGAAGGAGAAGGACCGGGCCGAGCAGCTGGAGCAGGCG
CAGCTCTCCGGCGCCCCCGACAAACAGCAGCTGCTGCTGATGTCCCGCATCAAGTGCCCC
AAGGCGTCGGTGCGCGACGAGCAGGGCCGCATCCGGCTGCATGGCGTGCTGCTGCGCACC
TACGTCTTCAACATCATCTTCAAGACGCTGTTCGAGGTGGGCTTCATCGTGGGCCAGTAC
CTCCTGTACGGCTTCGAGCTGAAGCCGCTCTACACGTGCGACCGGTCGCCCTGTCCCAAC
GTGGTCAACTGCTACATCTCGCGGCCCACGGAGAAGACCATCTTCATCATCTTCATGTTG
GCGGTGGCCTGCATCTCCCTGCTGCTCAACCTGGTGGAGATGTACCACCTGGGCTTCACC

AAGTGCCGCCAGGGGCTGAGGTACCGCCGCTCGAAGTCGATCTTGGGCAGCGCCTCAAAG
TTGCCCAGGGAGACGGCGGTGGTGCCCTTCGCCGTGCCCAGCTACAGCTACTTCCCCCAG
CCGCCCACGGCACCCGAGGCATACCGCTCAGAGTCCCACTACAACCTGACGGAGGCCGAC
TCGGGGTTCCAGCCCTACAGCAGCAAAGTGGCCTACAAGCAGAACAGGGACAACCTGGCG
GTGGAGCGCAGCAACAAGCCCGACAACAACACGGAGCTGAAGGGCATGAAAGGCTCGTGC
TCGGCCCCTGGCTCCCCCATGCAGAACAAGCGCAGGCCCAGCCACTCCAGTCGCAGCAGC
AACAACAAAACACGGCTGGACGACCTCAAGATCTGA

>Ch-gja6like-XM_012822071 (in reality gja4)
ATGTCAAGAGCTGACTGGAGTTTCCTGGAGCATCTACTCGAGGAGGGCCAGGAGCACTCG
ACTGGTGTCGGCCGCGTGTGGCTGACCGTCCTCTTCCTCTTCCGCATCCTGGTGCTGGGA
ACGGCCGCCGAGTCCGCCTGGAACGACGAGCAGTCCGACTTCATCTGCAACACCAAGCAG
CCCGGCTGCGAGGCCGTCTGCTACGACAAGGCCTTCCCCGTCTCCCACTTCCGCTACTTT
GTGCTCCAGATCATCTTCGTCTCCACGCCCACCATCTTCTACTTCGGTATCGTGGCCATG
GAGGTGGGGAAGAAGGCCAAGAGGCAAGAGGAGAAGAAGAGGAAGAAACAGGAGAGGGAG
AGCCGGGGAGGAGAGGCGTCGTCGGGGAGGCCCAGTCTGGAGGTGATTCAGGAGAAGGAC
GAAGATGCGGAGGAGGAGGAGAGAAAGGTGGAGCGAGGAGGAGGAGGGGTGAAGCGAGGC
GCGGGAGAGCCTCTGAGGCTGAAGGGCAAGCTGCTGTGTGCCTACGCCGTCAGCATCCTC
CTGAAGCTGTCCCTCGAGGTGGGCTTCATCGTGGGCCTCTGGGTCCTCTACGGGTTCGTC
ATCCCGGCCCGGTACGAGTGCCAGCGCGACCCGTGCCCGCACACGGTGGACTGCTACGTG
TCGCGGCCCACCGAGAAGACCATCTTCACCATCTACATCCAGACCATCGCCGCCATCTCC
GTGCTGCTCAACGTGCTGGAGCTCTTCAGCCTGCTGCAGCTAGCCATCAAAAACCACCTG
GAGAAGCGCTACCGGCGGCAGTGTCAGGGGCCCGTCGTCAGGGCGCGGGAGATTGCCAGG
GCCCCGTCTCGCCTGGAGATCGGCGGGGGGAAACACCCCCCCCCGCCCCTGCCTACGA
GGAGAGGGGGGATCAGTACCTCCCCGCCGAAGAGGGGGGGCTCCCTCAGCTGCCCAGCTA
TCTGAACTGCATCAGCAGTATGAGGCCCTTGGCAAACAGGGGCCACCACAACAGCAAACA
CCATCCGCAGCAACAGAGCAAGCGCAATAA

>Ch-gja5like-XM_012816449 Underlined: predicted as intron in this entry. We have included this part of the sequence as exon.
ATGGGTGACTGGAGCCTGCTGGGCAACATCCTGGAGGAGGTTCAGGAGCACTCCACGTCC
GTGGGCAAGGTGTGGCTCACCATCCTCTTCATCTTCCGCATCCTGGTGCTGGGCACGGCG
GCCGAGTCCAGCTGGGGCGACGAGCAGTCGGACTTCATGTGCGACACGCTGCAGCCGGGC
TGCGAGAACGTCTGCTACGACAGCGCCTTCCCCATCGCGCACATCCGCTACTGGGTGCTG
CAGATCGTCTTCGTGTCCACGCCCTCGCTCATCTACATGGGCCACGCCATGCACACCGTG
CGCATGGAGGAGAAGAGGAAGTGCAGGGAGCAGGAGGAGAGGGAGCGCGCCGACGCCGAC
GCCGACGACGCGGAGGGAGACGCGGGGGAGAAGGAGTACCTGGAGCAGAAGGAGCGGGAC
GCGAGCGGAGGAGGATGGGTAGGGGGCGTGCCGCCGGACGCTTCCAGAAAGATCCGTCTG
CGCGGGGCACTGCTGCAGACGTACGTGCTGAGCATCCTGATCCGCACGGTGATGGAGGTG
GGCTTCGTCACGGTGCAGTACCTCATCTACGGCATCTTCCTCAAGGCCGAGTACAAGTGC
ACCACGCCGCCCTGCAAGAACATGGTGGACTGCTACATGTCCCGGCCCACGGAGAAGAAC
ATCTTCATCGTCTTCATGCTGGCCGTGGCCGGGGTGTCCCTCTTCCTCAGCGTGGTGGAG
CTCTACCACCTGGGCTGGAAGCAGGTGAGGGGCTGCCTCCGGAGGTACGCCGCCAAGCAG
GCCTTCCACGGCGCCGGCGTGGCCAGCGCCAAGCACAAGAGTGCTGCTGCCATAGCCATC
GCCACGGCGACCGGCTCCATGGGGATGGAGAACGTGGACACGCCCGGCTCCCGCCCCACC
CCCGGGTGCACACCGCCTCCGGACTTCCACCAGTGCCTGGCGGCGTCGCGCGGCTCCCCG
GCGTCTGGCCGCCATCACCATCATCATCACCATCATCCTCCTCCTCCTCCTCCTCACTCC
CACCTTCATCCGTCGGCACAGGCGAACACACACACACACACACACTCGTCCCCCTCCTGC
CAGCCCTTCAGCACCCGTCTGGCTCTGCAGCAGAACTCCGCCAACATGGCCACCGAGCGA
CACATGGGGCCCGCACACAGCCCCGACTTCCTGCGCATGTCCTACCAGCATCCGCTCGCC
AACGGCCTGCCCAATGGCTGCCCTTCCCCCGGCTCTAGTCCCGCCCCCAGCCCCGCCCTG
CTCCACGCGGCGCTGCTGAAGGACAAGCGGCGACTCAGCAAGGCCAGCGCATCCAGCAGC
GGACGCGTGCGACAGGACGACCTGGCCGTGTGA

>Ch-gja5like-XM_012840593
ATGGCAGACTGGAGCCTTCTAGGTAACTTCCTGGATGAAGTGCATGAGCACTCCACGTCA
GTGGGCAAGGTGTGGCTGACGGTGCTGTTTATCTTCCGCATCCTGGTGCTGGGCACGGCG
GCTGAGTCCAGCTGGCTGGACGAGCAGGAAGACTTCATGTGTGACACGCAGCAGCCCGGC
TGCGAGAACGTCTGCTATGACCACGCCTTCCCCATCGCGCACATCCGGTACTGGGTGCTC
CAGATCGTCTTCGTTTCCACGCCCTCGCTGGTCTACATGGGCCATGCCATGCACACGGTC
CGCATGGAGGAGAAGAAAAGGAGGAAAGAACAGGAGGACCAGGGAGGAGGTGAGGGAGGA
GGAGGAGGAGAGGAGAAGAAGTACCCGCAGGAGGAAGAGAGGGATTGCGGGAAGGGCCAT
GAGGGTCCTGCAAAGATCCGTCTGAAGGGGGCGCTGTTGCGCACCTACATCCTGAGCATC
CTGGTGCGGTTGGTGATGGAGGTGATGTTCATCGTGGTGCAGTACCTCATCTACGGAATC
TTCCTGAATCCACGCTTCCTGTGTGAAGCCAAACCATGCCCACACATGGTGGACTGCTAC
ATCTCGCGGCCCACCGAGAAGAACATCTTCATCGTGTTCATGCTGGGCGTGGGGGTCCTC
TCTCTGCTGCTCAGCGTGATCGAGCTCTACCACCTGGCCTGGAAGCAGTGCAGACGCTAC
ATGAAGAGGTACGAGGCCAACCGACAACTGCAACAGCAGGAAGAGCAGCGACCTTTGACC

CCGTCCACTATTACCGCCACTTCCCCAGAGAAACCCCACCGCGCCCTGCCACTGCCACCC
TGCTCGCCCCCACCCGACTTCAGCCAATGCATGCCCCCGCCGTCACCCGTCCACGCCCAC
AGCCACGCCCCCAGCTGCCCCTCCTACAGCGACCGGCTGGCCAATCAGCAGAACTCCGTC
AACATGGCCGCTGAGCGTCACCGTGTTCTGGACGCCGGGGAGGACTTCCTGGGGAGGCAG
GTCTTCCTGACAGCGACAACGACAGCTCCGCCTCCGTCAGAGGAGGGCGGGGCCTGTCCC
CAGGTGATGACAAATGGTTTCCTGAAGGACAAGCGGCGTCTCAGCAAGACCAGCGGAGCC
AGCATGCGCATGCGTCCAGACGACATCGCTGTGTAA

>Ch-gja8-cx50-XM_012840595
ATGGGGGACTGGAGCTTCTTGGGCAACATTTTAGAGGAAGTTAACGAGCACTCGACGGTT
ATTGGTCGGGTGTGGCTCACCGTTCTCTTCATCTTCCGCATCCTCATCCTGGGCACGGCG
GCGGAGTTCGTGTGGGGCGACGAGCAGTCGGATTACGTGTGCAACACCCAGCAGCCCGGT
TGTGAGAACGTCTGCTACGACGAGGCCTTCCCCATCTCGCACATTCGCCTGTGGGTGCTG
CAGATCATCTTCGTGTCGACACCGTCGCTAGTGTATGTGGCCATGCCGTGCACCACGTG
CACATGGAGGAGAAGCGCAAGGAGCGCGAGGAGGCCGAGCTCAGCCGCCAACAGGAGGCC
AATGAGGAGCGGCTGCCCCTGGCGCCCGACCAGGGCAGTGTCCGCACCACCAAGGAGACT
AGCACCAAGGGCAGCAAGAAGTTCCGGCTGGAGGGGACCCTGCTGAGGACCTACATCTGC
CACATCATCTTCAAGACGCTGTTCGAGGTGGGCTTCGTGGTGGGCCAGTATTTCCTGTAC
GGCTTCCGCATCCTGCCGCTGTACAAGTGCAGCCGCTGGCCCTGCCCCAACACGGTGGAC
TGCTTCGTGTCTCGGCCCACTGAGAAGACGGTCTTCATCATCTTCATGCTGGCTGTGGCG
TGCGTCTCACTCTTCCTCAACTTCGTGGAGATCAGCCACCTGGGACTGAAGAAGATCCAC
TTCGTGTTCCGGAAGCCTCCGCAGGCGCAGGTGGAGGGCCGCGGCTCGCCGGAGAAGGGG
CTGCCTGTGGGCGTGTCCTCACTGCAGAAGGCCAAGGGCTACAAGCTGCTGGAGGAAGAC
AAGGCCACCGCCCACTTCCTGCCGCTGACGGAAGTGGGCATGGAGGCCGGACGGCTGCCC
TACCAGCAGGCAGTCGCCCCGCCGGGGGACGAGTCCAAGGTGTACGACGAGACATTGCCC
TCCTACGCGGCAACCACTGGGGGCGCGGCGGCGGCGATGGTGTCAGTTACGAATCAGGAC
GAGGAGGATCTGGATTCACCGATGGATGCCGAGGCCACGGATACGATAGAGGACACGCGA
CCCCTCAGCAGCCTGAGTCGGGCGAGCAGCCGCGCACGCTCCGACGACCTGACCGTATGA

>Ch-NP-gja8-XM_012816450 (predicted as KAT6B) Modified by truncation, potential intron in 3’end of sequence?
ATGGGTGACTGGAGCTTCTTGGGAAACATTCTAGAGGAAGTGAATGAGCACTCCACTGTG
ATTGGTCGGGTGTGGCTGACCGTGCTCTTCATCTTCCGCATCCTCATCCTGGGCACAGCG
GCGGAGTTCGTGTGGGGCGATGAGCAGTCGGACTTCGTGTGCAACACGCAGCAGCCCGGA
TGCGAGAACGTCTGCTACGACGAGGCCTTCCCCATCTCGCACATCCGCCTGTGGGTGCTG
CAGATCATCTTCGTGTCGACGCCGTCGCTGGTCTATGTGGCCACGCCGTGCACCACCTG
CACATGGAGGAGAAGCGCAAGGAGCGCGAGGAGGCCGCCGAGCTCCTGAGCCGCCCGATG
GAGGCCACGGAGGACCGCCCTCCCTTGGCGCCCGACCAGGGAAGCGTCCGCACCACCAAG
GAGACCAGCGCCAAGGGCAGCAAGAAGTTCCGGCTGGAAGGCACGCTACTGCGCACCTAC
ATCTGCCACATCATCTTCAAGACGCTGTTCGAGGTGGGCTTCGTGGTGGGCCAGTATTTC
CTGTACGGCTTCCGCATCCTGCCGCTGTACAAGTGCAGCCGCTGGCCCTGCCCCAACACG
GTGGACTGCTTCGTGTCTCGGCCCACTGAGAAAACCGTCTTCATCCTCTTCATGCTGGCC
GTGGCCTGCGTCTCACTCTTCCTCAACTTTGTGGAGATCAGCCACCTGGGCCTGAAGAAG
ATCCGCCTGGTCTTCCGGAAGCCTCCGCGGGGCCAGGGGGAAGGGGAGGGGGATGGAGAG
GGTGGACCGCTGACCCAGAGGGGCCTGCCCTCCATCGCCTCCCCCATCCTGCGGTCCAAA
GGGTACCGGCTGCTGGAGGAGGAGAGGGCCACGCCTCACTACTACCCCCTGACGGATGTG
GGTATGGAGGCAGGGAGGGTGCCAACATCTCTGCTGCTGCTGGAGAGAGCCTGCCAGGCT
ACCACATCAGTGCCCACTGAAGACGTCTCCAAAGTCTACAACGAAACACTGTCGTCTTAT
GCCCAGACCACTGAGTTATTTGAAGAGATCCTGGAGGAGGAAGAGGAAGAGGAGGATGAG
GAGGAGCAGGTGGCACAGGCAGAGGGTTTAGGAGGCATGGCAGCAGAAGGACAGGAGGGA
GGGGAGGTGCCAGTG

>Ch-gja9like-XM_012824682
ATGGGGGACTGGAACTTCCTTGGTGGGATCTTGGAGGAGGTCCACATCCACTCCACTATG
GTGGGGAAGATCTGGCTCACCATCCTCTTCATCTTCCGCATGCTGGTCCTGGGGGTGGCG
GCCGAGGACGTGTGGAACGACGAGCAGTCTGACTTCATCTGCAACACGGAGCAGCCCGGG
TGCCGCAACGTCTGCTACGACCAGGCCTTCCCCATCTCCCTCATCCGCTACTGGGTGCTG
CAGGTCATCTTTGTCTCGTCGCCCTCGCTCGTCTACATGGGCCACGCGCTCTACCGGCTG
CGCGCGCTAGAGAAGGAGCGCCAGCGCAAGAAGGTCGCACTGCGTCGCGAGCTGGAGGAG
GTGAACGCGGAGCTGGTGGAGCTGCGGCGGCGGATCGAGCGAGAGATGCGTCAGCTGGAC
CAGGGCAAGGTGAACAAGGCGCCGCTGAGAGGCTCTCTGCTGCGCACCTACGTGGCCCAC
ATCATCACGCGCTCCGCCGTGGAGGTGGGCTTCATAACGGGCCAGTACGCGCTCTACGGC
TTCCAGCTGGACCCGCTCTTCAAGTGCGAGCGCGAGCCGTGCCCCAACGTGGTGGACTGC
TTCGTGTCGCGGCCCACGGAGAAGAGCGTCTTCATGGTGTTCATGCAGTGCATCGCCTTC
ATCTCGCTCTCCCTCAACATCCTGGAGATCATGCACCTGGGCTACAAGGGGCTCAAGGAG
GGCATCCTGGACATCTACCCGCACCTTAGGGATGATCTGGAAGACAGCTACTACCCCACC
AAGGGCAAGAAGGATTCTGTGGTCCCTCAAGTCGGGATGGCCACTGGACGAAAGGCCACT
CTACCCTCCGCACCGGGTGGCTACAATCTGCTCATGGAGAAACCTCCCGACGGCCCTACC

TACCCCCCTCTCATCAACCCCTCATCTGCCTTCGTTCCTGTTCAGGGGGACGTGCCCCCT
AAAGGTGGGGCAAACGCCCTCAAGGAGTCGGCGCACAGTCCCACGGAGTACAACAGCAAC
TCCAACAACACCAGCAGTGAGACCCGTTCAGGGCCCAGCAACTCCGTCACTCCACCCAAG
CCAGACGAGGTGGAGGACCAGGCCCATCTTCCCCCACATGACGAGGAGCTGGAGTTGGAG
AGCCCTGATTCCCCCTGCCTGCCCAGAGACTTCTCTCACTCCTCGTGCCCCACACTGCCG
GTGAGCGCGGTGAAGAAGCCCTGGAAGGTCACCGCGCCTTGGCATTGCTCCACGGTGGTC
GAGGGCAACACCTCGGAAGAGGCCTCGCATGGGAGCGCCAAGGGCCGCAGCGGGGGTGCC
GCCAGCAGTAGTGGCGCAGCCTACGTGACCGCCCGTTCCCGCTCGGGCTCGAAGTCCAAG
AGACCCAGTCGGCCCAGCACGCCCGACTCCATCGAGGAAACGAGCTCGGAGTCGAGGGCC
AGCCCCAGGACCTCGTCTCCAGTCCGTCGCGCATCATTGTCGAGCAGCGCAAGCAGCCGA
CGAGCTGCCCCGACAGACCTGACGATATAA

>Ch-gja9like-XM_012816385 Underlined:sequence extends into previously suggested intron
ATGGGGGACTGGAACTTCCTGGGGGGCATTTTGGAGGAGGTGCACATCCACTCTACGATG
GTGGGCAAGATCTGGCTGACCATCCTCTTCATCTTCCGCATGCTGGTGCTGGGAGTGGCG
GCGGAGGACGTGTGGAACGACGAGCAGACCGACTTCATCTGCAACACGGAGCAGCCCGGG
TGCCGAAACGTCTGCTACGACCGCGCCTTCCCCATCTCGCTCATCCGCTTCTGGGTGCTG
CAGGTCATCTTCGTCTCCTCGCCCTCGCTCGTTTACATGGGCCACGCCATCTACCAGCTG
CGGGCGCTGGAGAAGGAACGCCACGGCAAGCGGACGGCCCTGCGGCGCGAGCTGGAGATG
GTGGACGTGGAGCTGACGGAGGTGCGGAGGCGCATCGAGCGCGAGCTCAAGCAGGTGGAG
CAGGGCAAGCTGAACAAGGCCCCGCTGCGGGGGTCGCTGCTCAGGACCTACGTGGCCAAC
ATCATCACGCGCTCGCTGGTGGAGGTGGGCTTTATGACGGGCCAGTACCTGCTCTACGGC
GTCCACCTGGACCCGCTCTTCAAGTGCGAGCGCGAGCCGTGCCCCAACGTGGTGGACTGC
TTCGTGTCGCGGCCCACGGAGAAGAGCGTCTTCATGGTGTTCATGCAGGGCATCGCCGCC
GTCTCGCTCTTCCTCAGCCTTCTGGAGATGATGCACCTCGGCTACAAGAAGCTGAAGAAG
GGCATTCTGGGATACTACCCCAACATCAAGGAGGAGCTTGACGACTCCTACATCAGCAAG
TCCAAAAAGAACTCTGTGGTGCAAACGGTGTGCATGAGCTCCGCTGGTCGCAAGGCAACC
ATCCCTACGACGACCAGCGGGTACACGCTTCTAATGGAGAAGCAAGGCAACGGCCCTACT
TACCCCATCCTCAACGCCACTTCCACCTTCATGCCTATCCAGGGCAACCCTGCCGGGCAG
CCGGGACTGGACATGCCCAGGGACCCCACAGACGTGGTGTTGAGCCCAATGGAGCGCAAC
AGTAACTCCAACAACACCAGCAGCGAGACGCGCTC

>Ch-gja10-cx62-XM_012821374 Splice site
ATGGGGGATTGGAACCTGCTGGGGAGCATCTTAGAGGAGGTCCATGTACACTCCACCATT
GTGGGGAAGATCTGGCTCACCATCCTCTTCATCTTCCGAATGCTTGTTCTCGGTGTGGCT
GCTGAGGACGTTTGGGACGACGAGCAGAGTGAATTTGTTTGCAACACAGAACAGCCTGGA
TGCAAGAATGTGTGCTACGACCAGGCGTTCCCCATCTCCCTTATAAGATACTGGGTATTA
CAGATCATTTTTGTGTCGTCACCCTCCCTGGTGTATATGGGACATGCCTTGTATCGTCTG
CGGACTCTGGAGAAGGAGCGACACAAGAAGAAAGTCCTGCTGAAGCTGGAGCTGGAGATG
ACTGAAGGTCTGGTGGAGGAGCACAGGCGGGTGGAGAGAGAGCTGAAGAAGCTGGAGGAG
CAGAAGAAGGTGAGGAAAGCTCCACTCCGCGGCTCCTTACTGCGAACGTACGTCTTCCAT
ATCTTGACCAGATCAGTTGTTGAGGTTGGTTTCTTAGTTGGGCAGTATTCCCTTTATGGT
ATTGGCTTGAAGCCGTTATACAAGTGTGAGAGGTTACCCTGCCCAAATACCGTGGATTGC
TTTGTGTCAAGGCCAACGGAGAAGAACATTTTCATGATCTTTATGTTGGTCATTTCGGGC
GTTTCCCTGTTTCTCAATCTACTGGAGATATTTCACCTTGGGGTGAAAAAGATTAAACAA
GGCATATATGGCGGCAAAGGTCTGGATGAGGATAGCATATGCAGGTCTAAGAAGAACTCA
ATGGTCCAGCAGGTCTGTATCCTGTCCAACTCCTCTCCTCAGAAACTGATACACGTGACT
CATTCGACCTGCGCAGTCGTTCCAGATGGGCGAGTGGAGTCTTCACCCTTTGGACTCCCT
CAGCCTAGGCAGGAGGTGAACAACAACGACATCACCAATGGCTCGGACCATAACGCCAGA
CAGAGTCGCCTGCCCAGCCATGCCGACCTCCCTGCCCTGAGGCAACTAGGGGCAACGGAG
CGCCGTCTCACTCTTGATACCCGGCAGGCGTCTTGCAGCAGTGATGATTCTAACGGGCCC
AAGGGCTCAGCACCCTCCAGAAGTGTGGGGGCGGCGCCGCAGCCGCGGGCCATGCGCAAG
CAGAGCCGGGTCAGCATTTTGAGCGAGGACCTGAGTGACTCTCCAGACAGTGCCACCTAT
CCCGCCGCAAGAAAAATGAGTTTCATGTCGCGCGGGCTCTCCGAGAGTCCTTCTGACAGC
CCTGATTCGAAGGCAGGCTCTGATGCTGAGGCCAAACGGATCGCTGAGGGGGAGAGTCCC
CCTGCAACACCACCCCCTGCCAGTGGAAGAAGAATGTCAATGGCAAAGTATGATTCTGGA
ACTGTCTTCAATCATGAAAAAATGAGCTCGGACTGGGGCGGCAGACACAGCAGACACAGC
AGACTGGGCTGTCCACAGATGCCAGCAGCGAGCCACGCTGTTCTACCACCCTCTGCTTAA

>Ch-gja10like-XM_012836705
ATGGGGGACTGGAACCTGCTGGGAGGCATTCTAGAGGAAGTCCACGTTCACTCCACCATA
GTGGGCAAGATCTGGCTGACCATCCTCTTCATCTTCCGCATGCTGGTGCTTGGCGTGGCG
GCGGAGGATGTGTGGGTCGACGAGCAGAGCCAGTTCGTGTGCAACACGGAGCAGCCGGGA
TGCAAGAACGTCTGCTACGACAGCGCCTTCCCCATCTCGCTCATCCGCTTCTGGGTCATG
CAGATCATCTTCGTCTCCTCGCCCTCGCTCGTCTACATGGGCCACGCGCTCTACCGCCTG
CGCTCGCTGGAGAAGGAACGCCACCGCAGGAAGGTGCAGCTGCGGGCAGAGCTGGAGGAT
GTCGAGCCCCTGCTGGAGGAGCACAGGAAACTGGAGAAGGAGCTGAAGAAGCTGGAGGAG

CAGAAGAGGGTGAAGAAGGCTCCTCTGCAGGGGTCCCTGTTGTGTACATATGTCATTCAT
ATCCTAACCCGATCAGTGGTGGAGGTGGGCTTCATAGTGGCTCAATATATCTTATACGGC
ATTGGCCTAGATCCCTTGTACAAGTGTGAGACTTTGCCTTGTCCCAACATGGTGGACTGT
TACGTCTCCAGGCCGACGGAGAAAACTATCTTCATGGTGTTCATGATTGTTATTGCGTGC
GTGTCGCTGTTTCTGAACCTGCTGGAGATATCGCACCTGGGCGTGAGGAAAATCAAGCAG
ACGCTGACGGGGCTGCGACCCGCCGACGACAGCGACAGCCTGGGCAACCTGCCCCGCAAG
CCCAATCTCCAGCAGCTGTGCGTGGTCACCAACATGTCGCCGCAGAAGAAAAACCCCATG
CTGGTGCAGACCAGCTTCTTCCCCGAGGGCCACGGCGACCCTCCCCCGCTCTATTTGGCA
GCCATGGATGTGTTGCCGAGTGGCGATGCACAGAGGGACAACAGCATCAACGAGGGCGGC
GGCGGTGGTGGCCACCTCGTCTCTTGTTTTCCCCAGCAGACCCGGCAGCTGCGTCTGGCC
AGCCAGGGTCGCATCCAGGGTCTGCGCTTCCAGATGCCTCTGGAGCAGCCACAGCTAGCG
CTTCAGCACGGCATCTCAGAGAGCCAACCCCATCTGCAGCAACACACACAGCAACACTCT
CAGTTTGCTGCTAACGAGTTTCTTGGGGAGGCTCACAGGAGGCTGTCATGTTTGGCCCAG
CAAACCCACGAGGGCCAGTACCCCGGCGGGCCCTACCAGCCCAATCACATGGCGGCGGCA
CCGGTCTCAATGCCCCACCTGGCCACTCATCTGGCCCAGCACCGGCCCAGCCGGGTCCTG
GAACTAGAGGCTCGGCGGGATTCGTCAGACAGCGACGTGCCGTACCCGCCGCACCCGCCG
CGCAAGGCCAGCTTCATGGCGCGGCTGCCCTCGGACAGTGACTCATCCAACGTGTCCAGC
TGCCAGACCAGCCAGAGCTCAGGGTCGGAGCTGGGCTCGCTCAACAACATGGTCATGAAC
CCGCCGCCAGGACGGAGAATGTCAATGGCAAGTAAAGCGCTGAGACTAAAGGCTTCTGAC
CTACTGATTTAG

>Ch-cx32.7like-XM_012829360
ATGGGTGAGTGGGACTTCCTTGGCCGGCTGCTAGATAAAGTGCAGTCCCATTCCACGGTG
ATTGGGAAAATCTGGCTGACTGTCCTTTTCGTCTTCCGCATCCTGGTCCTAGGGGCCGGC
GCAGAGAAGGTCTGGGGCGATGAGCAGTCGGACTTTGTCTGCAACACGGAACAGCCGGGC
TGCGAGAATGTGTGCTATGACGACGCCTTCCCCATCTCGCATGTGCGCTTCTGGGTGCTG
CAGATCCTGTCCGTCTCCACGCCCACGCTGGTCTACCTCGGCCACGTGCTGCACGTGGTC
CACATTGAGAAGAAGGTCCGCGCCCAGATGAGCAAGCAGATCCCAGATCAGCAGATGAAC
ATGTTCCTCATGAAGAGCTACAAGGTGCCCAAGTACAGCAAGGACAACGGGAAGGTGAGC
ATCCGCGGACGCCTCCTGAGGAGCTACATCATCAGCCTTTTCATCAAGATCCTGCTGGAG
GTGGCCTTCATCCTGGGCCAATACTACCTTTACGGCTTCACCCTAGATGCCCGCTATGTC
TGCAGCAAGTCCCCCTGCCCGCATCAGGTGGACTGCTTCCTGTCCAGGCCCACAGAGAAG
TCCGTCTTCATCTGGTTCATGCTGGTGGTGGCCTGCGTTTCGCTGCTTCTCAACGTGGTC
GAGATGGGCTACCTGACCGTCAAGAAGGTCAAGGAGTGTTTGAACCGGCGGCAGGACTAC
ACGGTCACGCCTATCACTCCAGTTTTGGAGCACCGGGATTTCAAGGCCAAGGACGAGGTG
ATCGAGAACTGGCTGAACAGGGAGGGGGAGCTGCAGAAGAAGGAGCAGGTGACCAGGAGT
GTGGCGTCTGAGGACAACAGCGCTAACATGGAGGAGGTACACATCTGA

>Ch-cx32.2like-XM_012829221
ATGGGAGAATGGGGATTCCTGTCAAATTTATTGGAAAAGGTGCAATCCCACTCCACCGTC
ATCGGGAAGGTTTGGATGACCGTTCTGTTCGTCTTCAGGATCATGGTGCTGGGGGCTGGC
GCAGAGAAAGTCTGGGGTGACGAGCAGTCCAAGATGATTTGCAACACAAAACAGCCTGGT
TGCAAAAATGTGTGCTATGATCAGGCCTTCCCTATCTCCCACATTCGCCTCTGGGTGATG
CAGATAATCTTTGTGTCGACCCCGACCTTGATATACCTGGGTCACGTCATACACATTGTG
CACAAGGAGGACAAACTCAGGGAGAGGTTACAGAATGAAGCCGGGAGGCAAGGGTTGAAG
ATGCCCAAATATACGGATGACAAAGGAAAAGTTCACATCAGAGGCAGCCTCTTGGGCAGC
TACATGACCAGCCTGGTGTTTAAGATTATTCTAGAGGTTGCGTTCATCGTGGGTCAGTAT
TACGTCTACGGCTTTGTGTTCGTGCCCCGGATAGAGTGCGAAGGGGAGCCTTGCCCCTTC
AAGGTGGAGTGCTTCATGTCACGGCCCACAGAGAAGACCATCTTCATCATCTTCATGCTG
GCGGTGTCCTGTGTGTCTCTGCTGCTGACGGTGGTGGAGATCTTCTACCTGCTGTGCAGA
AATTGCAAGAAGAGGCCCAACTACAGTGGAGCGCAGCAGATGATCACTATGTCAGGTTAC
AGTGCAGGGAAAATGTAA

>Ch-cx32.2like-XM_012829260
ATGGGAGATCTAGGATTCCTTTCAAAGCTGCTGGAACAAGTCAATTTCCACTCCACAGTC
GTCGGGAAAGTATGGATGACCGTTCTCTTTTTGTTCCGGATCATGGTTCTAGGAGCCGCG
GCAGAGAGTGTGTGGTCGGATGAACACTCTAACATGGTGTGCAACACGAACCAACCTGGT
TGCGAGAACGTGTGCTATGACTGGCAGTTCCCCATTTCCCACATCCGTTTCTGGGTGCTG
CAGATCCTCTTTGTGTCCACCCCGACCCTGATGTACCTCGGCCACGCCATGCACATCATC
TCCAAGGAGAACAAGCTGAGGGACCGGATCCAGAGGCATGAGGAGAACGTGAAGGCGCCC
AAGTACACAAACGACAAAGGGAAAGTGAGTATCAGGGGACAGCTGCTGGGCAGCTACCTC
ACGCAGCTCTTTTTCAAGATCCTCCTGGAGATCGGCTTCATCGTGGGCCAGTACTACCTC
TACGGCTTCATCATGGTGCCCATGTTCTCCTGCTCCAGGGATCCCTGCCCATTCACGGTG
GCCTGCTACATGTCCCGGCCGACTGAGAAGACCATCTTCATCATCTTCATGCTGGCTGTG
GCCGGCTTGTCCCTGCTGCTCAACGTGGTGGAGCTCTTCTACCTGCTCTGCTCCAAGTGT
GCCCGTGGCCGCCGTAACCAACGCCTCCGCAACACCACCCCCCCACCCAGCTGGAGCCCT
CATGCGGATGTGGACACCGTGGCACAGAACAACATTAACACGCACTTTACTGACGGCCAG
AGCCTGGGAGGGAGCCTGGATGGGGCCAGGGAGGAGAAGAGGCTGATGGAGCGTCACTGA

>Ch-cx32.2like-XM_012828709
ATGGGAGACTTTGGGTTCCTCTCCAAGTTGCTGAACAAAGTGCAGACGCACTCCACAGTG
GTAGGGAAGGTCTGGATGAGCGTCCTCTTCCTTTTCCGTATCATGGTCCTGGGGGCCGGA
GTGGAGAGCGTGTGGGGTGACGAGCGGTCCAACATGATATGCGACACCAAGCGGGTCGGC
TGTGACAACGTCTGCTACGACTGGAAGTTCCCCATCTCGCACGTGCGCTTCTGGGTGCTG
CAGATCATCTTCGTGTCCACGCCCACGCTGCTCTACCTGGGCCACGCCGTGCACGTCATC
CACAGGGAGAAGAAGCTGCACGAGCAGATTAGGAAGCCCGTGGAGGGCGTGGTGTTCAAG
GGGCCCAAGTACACCGACGACCGGGGCCGGGTGCAGATCAAAGGCGTCCTCCTGCGCAGC
TACATGGCCCAGCTTTTCTTCAAGATCCTCCTGGAGGTGGCGTTCATCGTGGGTCAGTAC
TACCTGTACGGCTTCTTCATGGACCCTAGGTTCGAGTGTGAGCGCTACCCCTGCTTTCAT
AAGGTGGAGTGCTTCATGTCAAGGCCCACGGAGAAAACCATATTCATCCTCTTCCAGCTA
GTAGTGGCCTGCGTGTCCCTGTTATCCTGGAGCCTGGAGGGATTCTACCTCCTCTGCAAG
CAATTGAAGAGGAAAGATCGCCACGTACGCCAGCCCAGCAGCATTCCAATGAGCCACGTG
CAACGTGCAGACATGGCAGACGCCGTGAACCAGAACAAAGCCAATATGTCCTACGAGGGG
GAAAAGCAACTTTGA

>Ch-gjb1like-XM_012819602
ATGAACTGGGCATCCTTTTATGCCGTGCTCAGCGGCATAAACAGGCATTCTACCGGCATT
GGCCGCATCTGGCTCTCTGTCCTCTTCATCTTCCGTCTCCTGGTGCTGGTGGTGGCGGCT
GAGAGCGTGTGGGGCGACGAGAAGGCCCACTTCATCTGCAACACGCAGCAGCCCGGCTGC
AACAGCGTCTGCTATGACCACTTCTTCCCTATCTCACACATCCGCCTGTGGGCCCTGCAG
CTCATCCTGGTGTCCACCCCGGCCCTTCTGGTGGCCATGCACATCGCGCACAGACGCCAC
ATCGACAAGCGGCTGTACCGGCAGGCTGGACGCTCGAGCCCCAAGGACCTTGAACTAATC
AAGACCCAGAAGATGAAGATCACGGGCGCCCTGTGGTGGACCTACATCATCAGCTTGATT
TTCCGGGTGCTATTTGAGTCGGCCTTCATGTACCTGTTCTACATGATCTATCCTGGTTAC
AAGATGTTCCGGTTAGTGAAGTGCGACTCGTACCCCTGTCCCAACACGGTGGACTGCTTC
GTCTCGCGGCCGACCGAGAAGACGGTGTTCACGGTCTTCATGCTGACCGTCTCCGGCATC
TGTATTCTGCTCAACATCGCTGAGGCCATGTACCTGGTAGCACGAGCCTACAGCAGACAT
TTTAACAATGCTAAAGACTCACCTATTGGAGCCTGGATCACTCAGAAACTGTGTTCCTTT
TAA

>Ch-gjb2like-XM_012834339
ATGAACTGGGGCACCTTTTATGCCGTGATCAGCGGCGTAAATAGGCACTCCACGGGCATC
GGCCGCGTCTGGCTCTCGGTCATCTTCATCTTCCGTATCCTGGTGCTGGTGGTGGCAGCA
GAGAGTGTCTGGGGTGACGAGAAGGCAATGTTCATCTGCAACACCCAGCAGCCTGGCTGC
AACAGCGTCTGCTACGACCACTTCTTCCCAATCTCACACATCCGCCTCTGGGTGCTGCAG
GTCATCCTTGTCTCCACGCCGGCTCTGCTGGTCGCAATGCACGTGGCGCACCGTCGCCAT
GTCGACAAGAGGATCCTCAGGATGTCAGGCCGCGGAAGCAACGCCAAAGATCTGGAGCAG
ATAAAGAACCAGAAGTTCAAAATCACCGGTGGTTTGTGGTGGACCTATACGATCAGCATC
CTCTTCCGCATCATCTTTGAAGTGGGTTTCCTCTTCATCTTCTATCTCATCTACCCTGGC
TTCACCATGTTGCGTCTGGTGAAATGTGACTCGTACCCGTGTCCCAACACTGTGGACTGC
TTCATCTCCCGGCCTACAGAGAAGACTATCTTCACCGTCTTCATGCTGGCAGTCTCTGGC
GTTTGCCTCCTGCTCAACATTGCAGAGCTGCTGTATCTGGTGGGCAAGGCATGCAGGAGG
TTCTGCCAAGGGTCCGACAAGGACGTCAGAGGCGCCTGGATCACGCAGAAGCTCTCCTCC
TACAAACAGAACGAGATCAATCAGCTGATATCAGAGCACTCTTTCAAGGGCAAATTCTCC
GTGGGTCGGAAGAGCCCAGCAGAGAAGGAGGAGAGGTGTTCTGCCTGCTAG

>Ch-gjb2like-XM_012842299
ATGAGCTGGGGGGCGTTGTATGCCCAGCTGGGCGGCGTGAACAAGCACTCCACCAGCCTT
GGCAAGATATGGCTGTCCGTCCTCTTCATCTTCCGCATCACCATCCTGGTGCTGGCCGCC
GAGAGCGTCTGGGGAGACGAGCAGGCGGACTTCACCTGCAACACGCAGCAGCCCGGCTGC
AAGAACGTGTGCTACGACCACTTCTTCCCCGTCTCGCACATACGCCTCTGGTGCCTGCAG
CTGATCTTCGTGTCCACGCCGGCGCTGCTGGTGGCCATGCACGTAACCTACCGCAAGCGC
GGCGTCAAGAAGGACCTCATGGCCGCGCGGGGAGACAAAGCCAACGAGGGCGACCTGGAG
AGCCTGAAGAGGAGGAGGCTGCCCATCACTGGCCCCCTCTGGTGGACGTACACCAGCAGC
TTGTTCTTCCGTCTGATCTTTGAGGCCGGCTTCATGTACGCCCTCTACTTTCTCTACGAT
GGCTTCCACATGCCCAGGCTGGTGAAGTGCGAGCAGTGGCCCTGTCCCAACAAGGTGGAC
TGCTTCATCTCGCGCCCCACGGAGAAGACCGTGTTCACCATCTTTATGGTGGGCTCCTCG
TCCATCTGCATAGTGCTTAACGTGGCGGAGCTGGGCTATCTGATCGTCAAGGCCCTGATG
AGGTGCTCGGCACGCATGGCACGGAAGAAGCACGCCTACACTCACCCAGAAAATGCATCC
AAAGACAAGGCTTACTTGCAGAACAAAAAGAATGAGATGTTACTGTCATCCTCCACTGAC
TCCAGCACTGGCAAGGCGGTCTAA

>Ch-gjb2like-XM_012820173
ATGAGCTGGGGCGAGCTGTACACCCAGCTGGCCGGCGTCAACCGCCAGTCCACCGGCCTG
GGCAAGGTGTGGCTCTCTTTCCTGTTCATTTTCCGCGTCACCATCCTGGTCCTGGCGGCC
GAGAAAGTGTGGGGGGACGAGCAGTCCGACTTCAAGTGCAACACGCTGCAGCCGGGCTGT

GAGAATGTCTGCTACGACCACTTCTTCCCCATCTCGCACGTGCGCCTCTGGTGCTTGCAG
CTGGTGTTTGCCTCCACACCACCCCTGCTGGTGGCCATGCACGTAGCCCATCGCAAGCGC
AGCAGCAAGTCCTCCGCACGGGCCAGGCAACAGGAGGAGGAGCTGAAGAGCATTCGCCAA
AGGAGGCTGCCCATCACGGGGACGCTGTGGTGGACCTACGCCCTCAGCCTGGTTTTCAGG
CTAGTGTTCGAGGCGGTGTTTGTCTATGCCATGTACGCCATCTACGGGAGCTTCTGGATC
CCTCGCCTGGTGCGCTGCGAGCAGTGGCCTTGCCCCAACGAGGTGGACTGCTTCGTATCA
CGGCCCACTGAGAAGACGGTGTTCACCATGTTCATGGTGGCGGCGTCGGGTGCATGCATG
GTGCTCAACGCGACCGAGCTCGCCTACCTCATAGCCAAAATGATGATGAAGTGCTCCAGG
CCAGGCGCCAGGAGAGATGCCTGCTCCTCCCGCTGCTCCAACCGCCCGCCAGTGGAGCAG
AACCAGAGGAATGAGTGTTTAACCTCCTTGACAACTCCTTTCTGA

>Ch-gjb2like-XM_012840586
ATGAGCTGGGGCACCCTGTACACCCAGCTGGCCGGGGTCAACCGCCAGTCCACCAGCCTG
GGCAAGGTGTGGCTCTCTGTCCTCTTCATCTTCCGCGTCACCATCCTGGCCCTGGCGGCC
GAGACAGTGTGGGGGGATGAGCAGTCCGACTTCACGTGCAACACGCTGCAGCCGGGCTGT
GAGAATGTCTGCTACGACCACTTCTTCCCCATCTCGCACGTGCGCCTCTGGTGCTTGCAG
CTGGTGTTTGCCTCCACACCACCCCTGCTGGTGGCCATGCACGTAGCCTATCGCAAGCGC
GACGACAAGCGCAGCATCCTGCGGCGCAACAACAAGTCAGCGGCCGCGTCCTCCGCACGG
GCCAAGCAGCAGGAGGAGGAGCTGGAGAGCATTCGGCAAAGGAGGCTGCCCATCACAGGG
ACGTTGTGGTGGACCTACGCCCTCAGCCTGGTTTTCAGGCTGGTGTTCGAGGTGGCGTTT
GTCTATGCCATGTACGCCATCTACGGGAGCTTCTGGATCCCTCGCATGGTGCGCTGCGAG
CAGTGGCCTTGCCCCAACGAGGTGGACTGCTTCGTATCACGGCCCACCGAGAAGACGGTG
TTCACCATGTTCATGGCGTCGGCGTCGGGTGCATGCGTGGTGCTCAACGCGACCGAGCTC
GCCTACCTCATAGCCAAAGTGATGGTGAAGTGCCCCAGGCCAGGCGCCAGGAGAGGTGCC
CGCACCTCCGTTGCCGCCAACTGCTCGCCCAAAGACAAGGGCCTGGTGCAGAACAAGAAG
AATGAGTCTTTGCTGTCCTCTGTGTCCTCCATGACATCCAGTGTCAAGGCTGTGTGA

>Ch-gjb3like-XM_012822385 (100% identical to XM_012822374 and XM_012822365, all mapping to NW_012217989)
ATGGACTGGAAAACTCTCCAGGCCCTGTTGAGTGGAGTGAACAAGTACTCCACCGCGTTT
GGCCGGATCTGGCTCTCCGTGGTGTTCGTGTTCCGGGTGATGGTCTATGTTGTGGCTGCC
GAGCGGGTGTGGGGTGATGACTCGAAGGACTTTGACTGCAACATCAAGCAGCCTGGCTGC
CCCAACGTCTGCTATGACCACTTCTTCCCCATCTCCCACATCCGCCTGTGGGCCTTACAG
CTCATCTTTGTCACCTGCCCTTCCTTCATGGTGGTGATGCACGTGGCGTACCGTAATGAA
CGTGAGCGTAAGCACCGGGTCAAGTACGGGGAAGAGACCGCCAAGCTTTATGCCAACACA
GGAAAGAAGCATGGCGGCCTTTGGTGGACCTACCTGCTGAGTCTCTTCGCCAAGACCTTC
ATTGAGATTGGCTTCCTGTACCTCCTCCACCACATCTATGACAGCTTCTACCTGCCTCGA
CTGGTCAAGTGTGACATCAAACCCTGCCCCAATGTGGTGGACTGCTACATCGGCCGGCCC
ACAGAGAAGAAGGTCTTCACCTATTTCATGGTGGGGGCTTCAGCCCTCTGCATTGTTCTC
AGTGTCTGTGAGATTATTTACCTAATCTCCAAGCGCATAGTCCGCTGCACCAACAAGATG
AATGCCCAAGAGAGAATCCGTCGTCACCGCAACAGGGAAGATGACAGTAAAAGCACCCTA
CCCGTTACAGACATGGACCACCACCCTGATTATAAACCAGAGACCAAGCCGGATTTTAAG
CCCGACTTCAAGGCTACTCTTAAGCCGCCTCCAAGGTCGTCAAGGTCCATTCGTGCATCA
GCCCCAAATTTGTTCTTTTCTGCCTCATAA

>Ch-gjb3like-XM_012818491 (100% identical to XM_012818489, both mapping to NW_012219726)
ATGGATTGGAAGGGTCTGGAAGGCCTCCTTAGTGGAGTGAATAAGTACTCTACAGGCTTT
GGCCGAATCTGGCTGGCGCTGGTGTTTGTTTTCCGTGTCATGGTGTTCGTGGTGGCAGCT
GAACGTGTGTGGAGCGATGACCAAAAGGATTTCGACTGTAACACACTAATGCCTGGGTGC
GCCAACGCATGCTACAACTACACCTTTCCCATCTCACACATCCGCCTGTGGGCTCTGCAA
CTCATTTTTGTCACCTGTCCTTCTTTCATGGTGGTGATGCACGTGTGGTACCGCGAAGAT
CGGGAGCGCAAATACCGTGCCAAGCATGGTGATGGTGTGCGCCTTTATAACAATCCAGGA
CAGAAGCACGGCGGTCTGTGGTGGACTTACTTCCTCAGCTTGTTCTTCAAGACGGGCATT
GAGGTGCTTTTTCTTTATCTGCTGCATTACATTTACGCAAACTTCGACATGCCCCGCAAG
GTGACCTGTGACATGTGGCCATGCCAACACAATGTGGACTGCTACATCTCCCGTCCGACC
GAAAAGCGCATCTTCACATACTTTATGGTGGGTGCCTCAGCTGTTTGTATTGTGCTCAAC
ATCTGTGAGATCTTCTACCTCATGGCCATGCGTGCGCTGCGCCGCAGTCACAGGGGCAAC
ATGGCCGCCAGGAAGAAGACCTGTGGAGAGCCGTACTGCACTGACTGTAGCCTACCTATG
GCCACCTACACACCAGCCAAGGAAATGAAACCAGAATGA

>Ch-gjb4like-XM_012822073
ATGAACTGGGGCGCGTTGGAGTCCCTGCTCACCGGGGTGAATAAATACTCCACGGTGTTC
GGCCGCATCTGGCTCTCCATGGTCTTCGTCTTCCGGGTCCTGGTGTTCGTAGTGGCGGCT
CAGCGTGTCTGGGGTGACGAGAACAAGGACTTTGTGTGCAACACCCTACAGCCGGGCTGC
GCCAACGTCTGCTACGACCACTACTTCCCCATCTCCCACATCCGCCTGTGGGCGCTGCAG
CTCATCTTCGTCACCTGCCCGTCCCTGTTGGTGGTGGGCCACGTCAAGTGGCGCGAGCAG
AAGGACCTGAGGTACACCACCTGCCACAAGGGGGCGCACCTGTACGCCAACCCGGGGAAG

AAGCGTGGCGGCCTGTGGTGGACCTACCTGCTCAGCCTGATCCTGAAGGTCAGCTTCGAC
ATAGGCTTCCTCTACATCCTCTACCACATCTATGACGGATACGATATGCCCAAGCTCTCC
AAGTGTGAGCTGGATCCATGTCCGAACATAGTGGACTGCTACATCTCACGTCCCACTGAG
AAAAAGATCTTCACCATCTTCATGGTGGTGTCTGCCTGTGTCTGCGTCGTCATGTGCTTC
TGCGAAATGGGCTACCTGATCTGCAAGAAGATCCACAAAAAGCTCAACTTGCACAAGAAG
AACCGTCAGCAGATGTTTGCTGAGAGCCACGAGCTTGGTGAGCTCGTTCCGCCCAGAAGC
TTGCAGTACAATCGGATCGACCCAACTGCCTCCAGGCCCGCTTCGAGAGCCCCGTCCAGA
GCCCCGTCCAGAACCTCAATCCACAATCTCCACAACAGCAAGAAGGAGGAGGCTGCCGCG
GCAGAGAGAGGGAAAAGCTAA

>Ch-gjb4like-XM_012826764
ATGAACTGGTCTGCACTGGAGAGCCTCCTCAGTGGGGTGAACAAGTACTCCACCGCCTTT
GGCCGCGTCTGGCTCTCAATGGTCTTTGTGTTCCGCGTCATGGTCTTCGTGGTGGCGGCC
CAGCGGGTGTGGGGCGACGAGAGCAAGGACTTCGTCTGCAACACGCGGCAGCCCGGCTGC
AGCAACGTCTGCTATGACAGCATCTTCCCCATTTCCCACATCCGCCTGTGGGCCCTGCAG
CTCATCTTCGTCACCTGCCCGTCACTCATGGTGATGGCGCACGTCAAGTACCGCGAGGAG
AAGGACAGGAAGTACATCGTTTCCCACACGGACGGCACGCACCTCTACGCCAACCCCGGA
AAGAAGCGCGGTGGCCTGTGGTGGACCTACATGCTCAGCCTGATCTTCAAAGCTGGTCTG
GATGCAGGTTTCCTCTACCTCCTTCACCATATCTACCACGGCTACGACATGCCCCGGCTC
GCCAAATGCAGCCTGGAGCCGTGCCCCAACACGGTGGACTGCTACATCTCTCGGCCCACT
GAGAAGCGGATCTTCACCCTCTTCATGGTGGTGTCCTCAGCATGTTGCATCTTCATGTGC
ATCTGTGAGATGTTCTACCTGATCTGCAAAAAGCTGCATAAGATTTTCAGGGTCCGGCAC
ACCCACGAGATGGAGCAGTTTGCTCAGACTCATGAGCTGAACAACATTGCGCCACCTCGA
TCGCAGTACAGGAGGGTGGACCCCACACTGTCCAGCACCCAGAACCTCAACAGGGAAAAG
ACAAGGGAAATAGCCACGTCCAAGTTGTAA

>Ch-gjb4like-XM_012822396
ATGAACTGGTCAGCTCTACAAGGCCTCATTAGTGGGGTCAACAAATACTCTACGGCATTT
GGCCGTGTTTGGCTGTCCATCGTTTTTATCTTCCGAATCATGGTATTCGTGGTCGCAGCC
GAGAAGGTTTGGGGTGATGACCAGAAAGACTTCAAGTGCAACACGGCACAGCCCGGCTGC
CACAATGTCTGCTACGACCACTTCTTCCCTGTGTCCCACATCCGGCTGTGGGCCTTACAG
CTCATCTTTGTCACCTGCCCCTCCTTCTTGGTGATGATGCATGTGCAATATCGAGAGGAA
CGTGAACGAAAGAACCGTCTCAAGTACGGCGAGGACGTCAAGCGTCTCTACCAGAACACG
GGCAAGAAGCGCGGAGGCCTGTGGTGGACCTACGTCCTCACCCTCGTCTTCAAGATGGCA
GTAGACGCCACCTTCGTCTACCTGCTCTACCACATCTACGAGGGCTACGACTTCCCGTCG
CTAATCAAGTGCTCGCAGGCGCCATGCCCCAACCTAGTGGACTGCTTCATCTCTCGGCCC
ACAGAGAAGCGCATCTTCACCCTCTTCATGGTGATCTCCAGCCTGGTGTGCATCATACTC
TGCTTAATTGAGACCATCTACCTGGTGGGCAAGCGCTGTGTGAAGATTGGCAGCCGGATG
CAATCCTCTCGGAAAATGCAGATGACGGCCTCCATGATGAATGTCAGGAACTCGAACATG
TTGGTGTTGGAGCCCCTCAGTGACAAGCGGCCCAACAAAGAAGCTGTTAGTCCAGCACCA
TCCTACAGTGTAGCCATGTCCAAGACATGA

>Ch-gjb4like-XM_012818492 (Red T: Only difference to XM_012818490; both mapping to NW_012219726) (a cx34.4 sequence)
ATGAACTGGGCCTTTCTGCAGGGCCTCCTCAGTGGGGTCAACAAATACTCCACAGCGTTC
GGCCGCATTTGGCTCTCGGTCGTGTTCATCTTCAGATTGATGGTGTTCCTCGTGGCCGCT
GAGAAGGTGTGGGGGGATGAGCAGGGGAACTTTGACTGTGACACGAGGCAGCCAGGTTGT
AAGAACGTCTGTTATGATCACTTCTTCCCCATTTCCTATTCACGGCTCTGGTCTCTGCAG
CTGATCTTTGTCACCTGCCCTTCACTGCTGGTGTTGCTGCATGTGGCCTACCGCGACGAC
CGGGAGCGAAAGCACGAGCTGAAGCATGGTGATGGCTGCACAAAGCTCTACGAAGACACA
GGAAAAAAGCGTGGTGGACTCTGGTGGACCTATCTATTCAGCCTGCTCTTCAAGTTGGCA
GTGGATGGTGTTTTCATCTTCCTGGTCTTCTACATCTATGAAGCCAACTTCTTTCCACTG
GCGGTGAAGTGCAAGGAAGCACCTTGCCCCCAGGCTGTAAACTGCTTCATCAGCCGGCCC
ACAGAGAAGCGCATCTTCACCGTTTTCATGGTGATCACCAGTGGTGTTTGTATCCTACTC
ACATTGCTTGAGATGGCCTACCTGGTGGGAAAGCGTTGTAAGGAGTTGGCGACCACTCGC
CCTCGGCACAGATATCCGGCAGCCATAACATCTGTAGTGAATCCCCAGGAACAGAACGCT
CATAATGAGTCCATACTGAATGACCATCGGGTTGATGAAAGCGCCCCTGTCTATAAGGCC
TGA

>Ch-gjb7-cx25-XM_012823856
ATGAACTGGGGCTTTCTGGAGAATGTGCTCAGCGGGGTCAACAAATACTCCACTGTGATT
GGACGAGTGTGGCTGTCCATCCTTTTTGTCTTCCGTATCCTGGTGTATGTGGCGGCAGCC
GAGCAAGTCTGGAAGGATGAGACCAAGGACTTTATCTGCAACACCCGGCAGCCTGGCTGT
GAGACCGTGTGCTACGACCATTTCTTCCCCATCTCCCAAGTACGCCTGTGGGCCCTCCAG
CTCATCATGGTGTCGACGCCGTCCTTGCTGGTGGCTCTGCATGTGGGCTACCGTGAGCAC
CGAGAGGCCAAATATGGAAAGAAGCTTTACGATAACAAGGGCAGGCTTGATGGAGGTCTA
CTTGCCACCTACATTATGAGCCTCGTCTTCAAAACTACGTTTGAGGTTGGGTCTCTGATC
GCCTTCTACCTCCTGTACAATGGCTTCACCGTCCCTAGGCTGCTCCAGTGCAGCCAAGAT

CCTTGTCCCAACACGGTGGACTGCTACATCGCAAGGCCCACAGAGAAGATGATTTTCCTC
TACATCATGGGCTGCACATCCATCTTATGCATCTGTCTTAATGTCATAGAGATGATGTAC
ATTATCTCCAAACAGTGTTGGAAGTGTTTCAGCAAACGCTATGTGCCTATAGAAGAGAGG
AGACGTTGTCACTGTGGCAAAGCTCACGCACTGCTAGCAGACTCAGTAGGAGCACTGGTA
TTGCCTCAGGCCAAAGAGGTGAGCTCACAGTTGGAGTTAAAACAGGAAAGTCCTTCCTGA

>Ch-gjc1-cx45-XM_012816830 Underlined: previously predicted introns are included as part of the cds.
ATGAGCTGGAGCTTCCTGACGCGCTTGCTGGAGGAGATCCAGAACCACTCCACCTTCGTG
GGGAAGCTGTGGCTCACTGTCCTCATCGTCTTCCGCATCGTGCTGACGGCCGTGGGCGGC
GAGAGCATTTACTACGACGAGCAGAGCAAGTTTGTGTGCAACTCCGGCCAGCCAGGCTGC
GAAAATGTCTGCTACGACGCCTTTGCCCCCCTTTCCCATGTTCGCTTCTGGGTCTTCCAG
ATCATCCTGTCCGCCATGCCTTCACTCATGTACATGGGCTACGCCGCCAACAAGATTGCC
AAGATGGAGGACACACGAGGGGTCGCGTCGGGAGGGAGCAGTGGCACGGGGACGGGCACC
AGAGGTGGAGGCTACACTCACCGGCGGCCGAGGAAGATGTACTTTGGGGCGCGGCAGCAC
CAGAGCGGCCTGGACGAAGGGGATGAGGAGCAGGAGGACGACCCCATGATCTACGAGGTG
CCCGAGCCGGACACCACACGGCGAGACCTCTTGCCACCGCGGCCCAAGCCCAAGGTACGG
CACGACGGGCGGAGGCGTATCCGTGACGACGGCCTGATGCGCATCTACGTGCTCCAGCTG
GTGACGCGCACGGCGCTGGAGGCCGGGTTCCTGGCAGGGCAGTACTTGCTGTACGGCTTC
CGCGTGGCGCCCGTGTTCGTGTGTTCGGGCAAACCGTGCCCGCACAATGTGGACTGCTTC
GTGTCTCGGCCCACCGAGAAGACCATCTTCCTGCGGATCATGTACGGCGTCACGTGCCTC
TGCCTCACCCTCAACGTCTGGGAGATGCTGCACTTGGGCATCGGCACCATCACTGACATC
ATACGCCGGCGACGGGCCACGCCCCCCGACGACGAGTACCAGCTGGGGCTGCTGGGGACC
GGGGGAGTGTCCGTCGGAGTCGGAGGCACCGGGGGACCGCTCAGTGAGGGGGAAGGCACG
GGCGGCGTGGGGGGAGCTGTTGGTGCGGACTACGTGGGCTACCCGTTCTCCTGGAACACG
CCGTCGGCCCCGCCGGGATACAACATAGTGGTGAAGCCGGAGGCCATGCCCTACACGGAT
CTGAGCAACGCCAAGATGGCCTGCAAGCAGAACCGGGAGAACATTGCCCAGGAGCAGCAG
CAGTACGGCTCAAACGAGGACAACTTCCCCACGGGGGCCGAACCGCGGGCCCCTCCCATC
AACAAGGACGTCATCCAGCAGGCGCAGGAGCAGCTGGAGGCCGCCATACAGGCCTACAGC
CAGCACCACGGCAACAATCATCATGACGACCCTCACCGCGGCGACGACGATGACAAGCCG
CAGAGCAACATCACCCCGGCGCAGAAAGAGCACAAACACCACCACCACCGGGCCAAGGCC
GGCAGGGGTGGGGGCAGCGCTGGGAGTGGGGGGGGGAGCAGTAGCAACAGCAGTAGCAGC
AAATCAGGAGAGGGCAAGCCATCTGTGTGGATCTGA

>Ch-gjc1like-XM_012817598
ATGAGCTGGAGTTTCCTGACCCGGCTGCTGGAGGAGATCCATAACCACTCGACGTTCGTG
GGGAAGATCTGGCTGACCGTGCTCATCGTGTTCCGGATCGTTCTGACGGCGGTCGGGGGC
GAGAGCATCTACTACGACGAGCAGAGCAAGTTCGTGTGCAACTCGCTGCAGCCGGGGTGC
GAGAACGTGTGTTACGACGCCTTCGCCCCGCTCTCGCACGTCCGCTTCTGGGTCTTCCAG
ATCATTCTGGTGGCCGCGCCCTCCCTCATGTACCTGGGCTTCGCAGCCAATAGGATCGCT
CGTCTGGAAGAGGGGCGGAGCTCGAGCAGGAAGCAGCGTAAGCTGTGCAGCGGTGGGCGG
CGGCCTCAGCGGGGCCTAGAGGAGGCGGAGGAAGACCAGGAGGAGGAGCCAATGATTTGT
GAGACGCTGGAGGAGGAGGAGGAAGAGGAGGAGACCGGCAGCGCGGGGCGGGCAAAGGCG
ACGCGGCACGACGGGCGCCGGCGTATCTGCAGGGACGGACTGATGCGCGTGTACGTGCTG
CAGCTGCTGACGCGGGCGGCGCTGGAGCTGGCCTTCCTCCTGGGCCAGTACGCCCTCTAC
GGGCTGGTGGTGCCCGCGCGCTACGTCTGCTCCGGCCCGCCCTGCCCCCACAGCGTGGAC
TGCTTCGTGTCGCGGCCCACGGAGAAGACCATCTTCCTCTTCGTCATGTACGGCGTGTCG
CTGCTGTGCCTGGCGCTCACCCTGTGGGAGGTGCTGCACCTGGGCCTTGGCTCCATCCTG
GACATCCTGCACGTGAGGCGCCGCCATCGTCATCGCCCGCCGCCACCCGACCACGCCATG
CCCCTCGGCCCTCTGGGAGGGGGCGTGCCTGTTAGCGAGGCGGGAGGTGGTGGGGGTGGG
GAGGGATACGGCAGCTACCCCTTCTGGAGCTCCGCCGCCCCCCTGCCCCCCCCCGGCTAT
AGCCTGAAGCCGGAGCAGCTGCCAATCAGTGAGCTGAACAGCCAGGCCAAGATGGCGGCG
AGGCAGAACAGAGCCAACCTCGCGCAGGAGGAGCAGTACGGGGGAGGGGGAGGGGCTGCG
CAGGACCAGCAGCAGCAGCAGCCGATCAGATGA

>Ch-gjc1like-XM_012821065 Splice site
ATGAGCTGGAGCTTCCTGACGCGTCTGCTAGAGGAGATCTCCAACCACTCCACCTTCGTG
GGCAAGGTGTGGCTCACACTGCTCATCGTCTTCCGCATCGTGCTGACGGTCGTGGGCGGC
GAGACCATCTACCACGACGAGCAGAGCAAGTTCGTGTGCAACACGCAGCAGCCCGGCTGC
GACAACGTCTGCTACGACGCCTTTGCGCCGCTCTCGCACGTCCGCTTCTGGGTCTTTCAA
ATCATCATCATCACCACGCCCTCCATCATGTACCTGGGCTACGCCATGCACCGGATCGCC
CGTGCGGCTGACGATGAGTACCATCCGCGCCGCAAGCGGGCACCGGTCGTCACCCGCGGG
CCCAGCCACGACTACGACGACGTTGACGAGACCGGCGAGGACGTGCCCATGATCACCGAG
GAGTTGGAGGCCGAGCGCGGGGGGAAGGGCGGGGCGGGAGCGGCGCTGGTCGTCAAGGCG
GCGGCTCCCGAGGGGATGACGATGAAGCACGACGGCCGGCGGCGCATCCTGAGAGACGGG
CTGATGAAGGTCTACGTGGTGCAGCTGCTGTCGAGGATCGCCTTCGAGGTGGCCTTCCTG
TTCGGCCAGTACCTGCTGTACGGCCTGGAGGTGGAGCCGTCCTACGTCTGCATGCGGAGC
CCCTGTCCACACACCGTCGACTGCTTCGTCTCCA**GG**CCCACGGAGAAGACCATCTTCCTG

ATCACCATGTACGTCGTGAGCGCACTCTGTCTGCTCCTCACCTTCCTGGAGATCTGCCAC
CTGGGCATCAGTGGTATCCGCGACAACCTGAAGGGCCGCTCAACCGTCCGCCGTTCCCGC
CAGCCCCTCCACTCCTCTGGCCTCCCCAGCCAGCACGCCACCTCCCTGCTCAAGCAAGTC
CCCTCTGCCCCTCCTGGATACCACTCCGTGCTGAAGAAGGACACCCCCGGCCGGCTCCGG
CCCGAGTTCAGGGAACTGAACCTGAAGGACTCCGGTCGGGAGTCACCGGGGGACGAGTTG
GCGGGTCGAGACCTGGAGCGCATGCGGCGGCACCTGAAGATGGCCCAACAGCACCTGGAC
CAGGCCTACCAGAGCGAGGAGCTGGCGCCAGTGTCGCGCAGCAGCAGTCCGGAGTCCAGC
AGCAAAGCCGCTGAGCAGAACCAGCTCAACTACGCCCAGGAGAAGCAGGCCAGCACCAGC
GACAAAGGTGTCCATGCCTGA

>Ch-gjc1like-XM_012836489
ATGAGCTGGAGCTTCCTGACGCGCCTGCTGGAGGAGATCTCCAACCACTCCACCTTTGTG
GGAAAGGTGTGGCTCACCATGCTCATCGTCTTCCGGATCGTGCTGACGGTGGTGGGCGGG
GAGTCCATCTACTACGATGAACAGAGCAAGTTTGTGTGCAACACGCAACAGCCCGGGTGT
GAGAACGTGTGCTACGACGCCTTTGCACCGCTCTCGCACGTGCGATTCTGGATCTTCCAG
ATCATCTCCATCAGCACGCCCACCATAATGTACCTGGGCTTCGCCATGCACCGCATCGCC
CGAATGGGTGACGGCGAGTACCAGCCACGGCCGCGCAAGCGCATGCCCATGGTCCACCGG
GGGGCTGCGCGTGACTACGAGGAGGCAGAGGACAACGGCGAGGAGGACCCCATGATCAAC
GAGGAGATCGAGCTTGAGAAGGACAAGGACAAAGAGACCGAGAAGCCCTGCAAGAAGCAC
GACGGACGCAGGCGGATTAAGAGGGACGGCCTGATGAAGGTGTACGTGATGCAGCTCCTG
TTTCGCACCGGCTTGGAAGTGGCCTTCCTGTTCGGCCAGTACATCCTGTACGGCCTGGAG
GTGATCCCGTCCTACGTGTGCACCCGCAGCCCCTGCCCACACACGGTCGACTGCTTCGTG
TCGCGACCCACCGAGAAGACCATCTTCCTGCTCATCATGTACGGCGTCAGCTGCCTCTGT
CTGCTGCTCACCGTGCTGGAAATCCTACACCTGGGCATCAGCGGGCTCCGCGATGCCTTC
CGCCAGCGCTCGGCGTCCCACAACCGCAGCCAAGTAGCCATGTCCAGCCAGCGGCCCTCC
ATCTGCCGGCAGGTGCCCACTGCTCCGCCAGGCTACCACACAGCCGTCAAGAAGGATGGC
GGAAAGCTGCCCGCCGGCATGAAGCCATCTGACTTCCGCGATAACTTGGTCGACTCGGGC
CGGGAGTCGTTTGATAACGAGACATCGTCCCGAGAGCTGGACCGGCTGCGTCGGCACTTG
AAGCTGGCCCAGCAGCACCTGGACCTGGCCTACCAGAATGGAGAGAGCAGCCCGTCGCGG
AGCAGCAGCCCGGAGTCGAACGGCACGGCCGTGGAACAGAATCGACTCAACTTTGCTCAA
GAGAAACAGGGAGGAACCTGTGAAAAAGGAATCAGAGCCTGA

>Ch-gjc2-cx47-XM_012827872
ATGAGTTGGAGTTTCCTTACACGTCTTCTGGAAGAGATCCACAACCACTCCACCTTTGTG
GGGAAAGTGTGGCTGACTGTTCTTATCATCTTCCGCATTGTGCTGACTGCCGTTGGGGGC
GAGTCCATCTATTCGGACGAGCAAACCAAGTTCACCTGCAACACCAAGCAGCCCGGCTGC
GACAACGTGTGCTATGACTCATTTGCGCCGCTCTCGCACGTCCGGTTTTGGGTCTTTCAG
ATCATCATGATCTCTACGCCTTCTGTCATGTACCTCGGCTACGCCATCCACAAGATTGCT
CGCTCGTCGGAGAACGACCGGCGGAAGTTCCGCAGGTGCCAAAGAAAGAGCCACCGCAGC
CGTTGGCGAGACAGCCACCCACTGGAACAGGTGCTTGAGGAGGAAGACGACGATGACGCC
GAGCCGATGATCTACGAAGATGCTTTGGAAGTGCAGGACGCAAAGCCCGATGTGACCAAC
TGTCCCATCAAGGATCCGCAGAAACACGACGGGCGGCGGAGGATCATGGAGGAGGGGCTG
ATGAAGATCTACGTGATCCAGCTCTTGTCCCGCGCCGTCTTTGAGATCGGCTTCCTCGTG
GGTCAGTACCTCCTGTATGGCTTCCGCGTCAATCCGTCCTACGTGTGCAATAAGATCCCT
TGTCCTCATAAGGTGGACTGCTTCATCTCGCGACCCACGGAGAAGACCATCTTCCTTCTC
ATCATGTACGTGGTCAGCTGCCTGTGTCTGGTGCTCAACGTGTGTGAGATGTTTCACCTG
GGCATGGGCGCCTTCAGAGACACTCTCCGCAGACGGAGAAACAAAGGTCGACAGCCTCCT
TACAGCTACTCTTACTCGAGGAACATCCCGGCATCACCCCCGGGGTACAACCTGGTCATT
AAGTCAGACAAACCTGGAAGGATGCCCAACAGTCTCATATCACACGAGCAGAACATGGCC
AACGGAGGTCAGGAGCAGCACTGTATAAGTCCTGATGAAAACATCCCCACTGACTTGGCC
AGTTTGCATCGCCACCTACGGGTGGCCCAGGAGCAGCTGGATATGGCCTTTCAGACATAC
AACACCAAAACCAACCCCCAGACATCCAGAACCAGCAGCCCAGTTTCGGGGGGCACCATG
GCGGAGCAGAACCGGGTCAACACAGCCCAGGAAAAACAAGGAGCGAGGCCCAAAGCCACC
ACTGAGAAAGCTGGAACAATAGTCAAAAATGGCAAGACATCTGTATGGATCTGA

>Ch-gjd2-cx36-XM_012823340 Splice site
ATGGGGGAATGGACCATACTAGAGAGGCTCCTGGAGGCTGCTGTCCAGCAGCACTCTACT
ATGATAGGAAGGATCCTACTGACAGTGGTGGTGATCTTCCGGATTCTAATCGTAGCGATA
GTTGGAGAGACTGTTTATGATGATGAACAGACCATGTTCATCTGTAATACCTTACAACCG
GGCTGTAACCAAGCATGTTACGATAAGGCATTCCCCATATCCCATATCAGATATTGGGTG
TTTCAGATCATAATGGTGTGCACACCGAGTTTATGCTTTATCACATATTCGGTTCATCAG
TCTGCAAAACAAAAGGAACGACGGTTCTCCACTGTGTTTCTGACGGTGGATAAGGATCAA
GATTCAATGAAACGAGACGACAGCAAAAAGATCAAAAATACAATCGTGAACGGAGTACTT
CAGAACACCGAAAACTCTACAAAAGAAGCCGAGCCCGACTGTTTGGAAGTGAAAGAGATC
CCAAACCCAAATGTGAGAACTCCTAAATCCAAAGCGAAACGGCAGGAGGGCATCTCCAGA
TTTTATATCATTCAAGTGGTTTTCAGAAACGCGCTGGAAATTGGGTTTTTAGTTGGTCAA
TATTTCTTGTACGGATTCAACGTGCCCGCCGTGTATGAGTGTGATCGATATCCCTGCATA
AAAGATGTCGAGTGCTATGTTTCCAGACCCACGGAGAAGACCGTGTTTCTGGTCTTCATG

TTCGCGGTCAGTGGCTTTTGCGTGGCGCTAAATTTGGCAGAACTGAATCACTTGGGGTGG
AGGAAAATCAAAGTGGCCGTAAGAGGAGTACAGGCTAGGAGAAAGTCCGTTTACGAAATC
AGAAATAAGGACTTGCCCCGAATGAGTATGCCTAATTTTGGTCGCACCCAGTCAAGTGAC
TCTGCCTATGTGTAG

>Ch-gjd2-XM_012819299 Splice site
ATGGGGGAATGGACCATACTAGAGCGGCTTTTAGAGGCTGCTGTGCAGCAGCACTCTACT
ATGATCGGAAGGATCCTACTAACAGTAGTGGTGATCTTCCGGATTCTAATAGTCGCTATA
GTGGGGGAGACGGTGTACGACGACGAGCAGTCTATGTTCGTGTGTAACACGCTACAGCCT
GGCTGCAACCAGGCTTGTTATGATAAAGCATTCCCAATTTCCCACATCAGATACTGGGTG
TTCCAGATCATCATGGTGTGCACCCCCAGCCTCTGTTTCATTACCTACTCCGTGCACCAG
TCAGCGAAGCAGAAAGAGCGGAGGTTCTCGACTGTTTACCTTTCCCTGGACAAGGATCAG
GATTCTATGAAAAGAGATGACAGTAAAAAGATCAAGAACACAATTGTGAACGGAGTACTA
CAGAACACGGAGAACTCAACCAAAGAATCCGAGACAGATTGTCTTGAAGTGAAGGAGATG
CCTAGTTCAGCCATGAGAAATACCAAGTCTAAAATGAGACGGCAGGAAGGCATATCAAGA
TTCTACATCATCCAGGTCGTTTTCCGAAACGCGCTAGAGATAGGGTTTTTAGTGGGTCAA
TACTTCCTCTATGGATTCAATGTCCCTGCCGTGTATGAATGTGATCGATATCCCTGCATC
AAAGATGTTGAGTGCTACGTTTCAAGACCAACGGAGAAGACCGTGTTTCTGGTCTTCATG
TTCGCCGTCAGTGGGATATGCGTGGTTCTGAACCTCGCGGAACTCAACCACCTTGGCTGG
AGGAAAATTAAAACAGCCGTGAGAGGTGTGCAGGCTAGGAGAAAGTCAATTTACGAGATC
AGGAACAAAGACTTGCCGCGTATGAGCATGCCCAATTTCGGTCGCACTCAGTCGAGTGAC
TCCGCCTATGTGTAG

>Ch-gjd2like-XM_012828866 Splice site
ATGGGGGAGTGGACCATCCTGGAGCGCCTCCTGGAGGCTGCTGTACAGCAGCACTCCACT
ATGATTGGGAGGATCCTGCTGACAGTGGTGGTTATCTTCAGGATCCTGATCGTGGCCATC
GTTGGGGAGACCGTGTACGAGGATGAGCAGACCATGTTCATCTGCAACACCATGCAGCCC
GGGTGCAACCAGGCGTGCTACGACAAGGCCTTCCCCATCTCACACATCCGCTACTGGGTG
TTCCAGATCATCCTGGTGTGCACGCCCAGCCTGTGCTTCATCACCTACTCTGTGCACCAG
TCGGCCAAGCAGCGCGAGCGCCGCTACTCCTTCCTCTACCCGATGCTGGAGCGGGACTAC
GGCCGGGACGGGGCGCGTAGGTTGCGCAACATCAACGGGATCCTGGTGCAGCACCCAGAT
GGAGGAGGGGGTGGGAAGGAGGAGCCAGACTGCCTGGAGGTGAAGGAGATCCCCAACGCG
CCGCGGGGGCTGACGCAGAGCAAGAGCTCCAAGGTGCGCCGGCAGGAGGGCATCTCACGC
TTCTACATCATCCAGGTGGTGTTCCGCAACGCACTGGAGATCGGCTTCTTGGCCGGCCAG
TACTTCCTGTACGGTTTCAGCGTGCCGGGCATCTTCGAGTGTGACCGGTACCCCTGCCTG
AAGGAGGTGGAGTGCTACGTGTCACGGCCCACTGAGAAGACAGTCTTCCTGGTGTTCATG
TTTGCGGTGAGTGGCATCTGCGTGATCCTCAACCTGGCCGAGCTCAACCACCTCGGCTGG
CGCAAGATCAAGGCGGCCATTAGGGGCGTGCAGGCGCGCCGCAAGTCCATCTGTGAGGTC
CGCAAGAAGGACATGTCCCACCTCTCACAGCCGCCCAACATGGGCAGGACTCAGTCCAGC
GAGTCGGCCTACGTCTGA

>Ch-gjd2like-XM_012817227 Underlined: previously predicted intron is now included as part of exon.
ATGACGGAGTGGACGCTGCTCAAGCGGCTGCTGGACGCCGTCCACCAGCACTCCACCATG
ATCGGCCGCATCTGGCTCACCGTCATGGTCATCTTCCGCCTGCTCATCGTGGCCGTGGCC
ACCGAGGACGTGTACGCCGACGAGCAGGAGATGTTCGTGTGCAACACGCTGCAGCCGGGC
TGCGTGAACGTGTGCTACGACGCGTTCGCCCCCATCTCGCAGCCACGCTTCTGGGTCTTC
CAGATCATCATCGTCTCCACGCCCTCCCTCTGCTTCATCATCTACACCTGGCACAACCTG
TCCAAGCTGCCCGCGGAGGCCGACGCGGGCAAGGAGAGCCACGACGCGTACGCCCGCAGC
TGCGACTCGGACAGCTGCTCCATCAAGTCACACCGGCACCTGGGCCACAGTCTGGCAGAC
GTGCTGGAGGGCATCGCTGCTCAGTGCAACCAGAAGAGCGCCTGCCTCTCACCGCCCAAG
AGCAGGGTCTGCCGGGGCGCTGCCGGGGCGAAGTCTGGGGTCCTCTCCAAGTACTACATC
TTCCATGTGTGCTTCCGTGCCGCACTGGAGATAGGTTTCGTCTTTGCCCAGTGGCTGCTG
TTTGGCTTCCAGGTCCCGGCACACTTTCTCTGCACAGCTTTCCCCTGCTCCCAAAGTGTG
GACTGCTACGTGTCCAGGCCCACCGAGAAGACCATCTTCCTCATCTTCATGTTCAGTGTG
GGTATCTTCTGCATCTTCCTCAACTTCCTGGAGCTCAACCACCTGGGCTGGAAGAAGATC
ACAATGTCGGTGAGACTGAAGGACAGCTCCTGGAAGGGCTACGAGGCCATCAACCAGGAC
AGCCACTCCGTCACCTCCCTCACCTTCAGGGACGTGACCAGCACCACCTCCCTGCCCACT
CTTGATCTGGTGGTGGGCCACAGGCCGGACTGGACCTGTGCTGGGAACTGCACACCGCTG
AAGGAGGAGCAGGAGGACAGCCTGCAGAACCCCACAGGGAATCCGAGAGCAGCACAGTCC
CTGAAGAGCAAGACTCACAAAGGACGGATTTCAAAGCAGAGGAGCACTGAAGTCTGGATT
TAA

>Ch-gjd2like-XM_012838313
ATGGGAGACTGGTCTATTCTCGGCCGCTTCCTCACAGAGGTGCAGAACCACTCCACCGTC
ATCGGCAAGATCTGGCTGACGATGCTGCTGATCTTCCGCATCCTGCTGGTGACGCTGGTG
GGCGACGCCGTCTATAGCGACGAGCAGTCCAAGTTCACCTGCAACACCCTGCAGCCCGGC
TGCAACAACGTCTGCTACGACACCTTCGCCCCCGTCTCACACCTGCGCTTCTGGGTCTTC

CAGATCGTGCTCGTGTCCACGCCGTCCATCTTCTACATCGTCTACGTGCTGCACAAGATT
GCCAAAGACGAGAAGCTGGAGCTGGAGACGGTGCACGTGCAGAACAAGCGCCCCCTCGGT
GATTACCTGGGCCGGCTGGAGAGAGAGAGGGACAGGGAGAGGGAGAGAGGGGAGACCTAT
GGCAAGAGCCCTGGGCTGCCCTACGGGGGTCCGCACTACGAGGAAGAGTGGGCTCCCCAT
GAGGAGGAGTGTGTTGAGCGAATTCTCCTCGAGGACGACTACGGGGAGGTGGGGAAGGAC
CCCACGGAGCTCTCCAGCAAGGTCCTGCTCATCTACATCGTCCACGTGGTGCTGCGGTCC
ATCATGGAGATCACCTTCCTGGTGGGCCAGTACTACCTGTTCGGCTTCGAGGTGCCGCAC
CTGTTCCGCTGCGAGACCTACCCGTGCCCGACGCGCACGGACTGCTTCGTGTCGCGCGCC
ACCGAGAAGACCATCTTCCTCAACTTCATGTTCAGCATCAGCCTGGGCTGCTTCCTGCTC
AACATCGTGGAGCTGCACTACCTGGGCTGGGTGTACATCTTCCGCGTGCTCTGCTCCGCC
TGCTCCGTGTGCTGCCGGCCGGAGAGGGACCCCGTGGAGCACATGGGCCTCTACGCCGAC
CACAACCCGCTGCTGCTGCAGCTGGAGCACTCCCTGCGGGGCCGCCTCATCCTGCAGACG
CCCACGCCCATCGCCCAGGAGAAGGCCGGCGGCGGACTGCTCACCCACGCGCCCGCCATC
TCCTTCGAGACGGACTCCACGGTGGAGTGCACGTCCAAGCGGAGCGCCGAGGAGATGGAG
CGCATGAGGGCCAAACTGACCAACATGGCCTTGCTGGGCCGTACCAAGAAGTCCTGGCTA
TGA

>Ch-NP-cx39.2 previously unidentified
ATGGGAGACTGGTCCATTCTTGGCCGCTTCCTAACGGAGGTGCAGAACCACTCAACCGTC
ATCGGTAAGATCTGGCTAACGGTGCTGTTGATCTTCCGCATCCTGCTGGTGGCGCTGGTG
GGCGACGCCGTGTACAGCGACGAGCAGTCCAAGTTCATCTGCAACACACTCCAGCCCGGT
TGCAACAACGTCTGCTACGACACCTTCGCCCCCGTCTCGCATCTCCGCTTCTGGGTCTTC
CAGATCGTCCTCGTCTCCACGCCGTCCATCTTCTACATAGTCTATGTGCTGCACAAGATT
GCCAAAGACGAGAAGCTGGAGGTGGAGAAGGTGCCGGCGATAGCCAGGTGTCCGCCCTCG
GAGGATCTCTCGGCACAGGGGAAACTGGAGGAAGAAGATGCCTTAGACTCCAGCGCACCT
CCCTTTGGTTCTGCCTCCGAGGAGGAGGCTTGGGGTCCTCCGGTGGTTGAGAGCGTGGAG
CAGAGCCTGCTGGAGGAGGGGGTTCGGGTGGTGAGGAAGGACCCCACCCAGCTCTCCAAC
CAGGTGCTGCTGATCTACGTGGTTCACGTGGTGCTGAGCTCCATCATGGAGATCACCTTC
CTGGTGGGTCAGTATTACCTGTTTGGCTTCGAGGTGCCACAACTCTTTCGGTGCGAGACG
TACCCCTGCCCAAATCGAACTGACTGCTTCGTCTCGCGCGCCACGGAGAAGACCATCTTC
CTCAACTTCATGTTCAGCATCAGCCTGGGCTGCTTCATCCTCAACATCGTGGAGCTCCAC
TACCTGGGCTGGATCTACATCTTCCGCGTGCTGTGCTCCGCCTGCTCCACCTGCTGCACG
CCTCACAGGAGCCCGCTGGAGCGTCTTGGCTTCTACTACGACCACAACCCCCTCCTGCTG
CAGCTGAAGCACTCTCTCCAGAGCAGGGTGGTCCTGCAGGCCCCGTCCTCCATGGTGCAA
GAGAGGACCTGCAGTGTGCCTGCCTACACCCCTGCCATCTCCTTCGAGACGGATTCCACG
CTGCAGTGTACGTCCAGGAGGAGCCTGGACGATAGGGAGCACAGCAAGGTCAAACTGGCT
AAATTAGGCAGGGGTGAAAAATCCTGGTTGTAA

>Ch-gjd3like-XM_012837668 Splice site (98.4% identical to XM_012837669; both mapping to NW_012837669)
ATGGCGGACTGGGGGTTCCTGGGGGGGCTGTTTGAGGCGATGCAGACCCACTCCCCCCTA
CTGGGGCGCCTGTGGCTGCAGATCATGCTGGTGTTCCGCATGCTCATCCTGGGCACCGTG
GCCTCCGACCTGTTCGAGGACGAGCAGGCGGAGTTCGAGTGCAACACGGCGCAGCCGGGC
TGCAAGCAGGTGTGCTACGACCAGGCCTTCCCCATCTCACAGTACCGCTTCTGGGTGTTC
CACATCGTGCTCATCTCCACGCCCGCCCTCCTCTTCATCATGTACGCCATGCACCTGCAC
TCCAAGAGCCAGGCCCGCCAGGAAGGTGCCAGCTCCAAAAGCGGGCAACTGGGCATCAAC
GCCCACACCACTGAGCCACTGATGCAGAAACCGGACGAGGGCGGGTTGAACCCGCGACAG
GACCATAACGTGATGCGTCTGTACATGCTGAACGTGGGGTTCCGCTTCCTGGCTGAGGTG
GCATTCCTGGTGGCTCAGTGGGCGCTGTACGGGTTCCGCGTGGAGGCCCGCTTCCCCTGC
AGCACGTTCCCCTGCCCCTACACGGTGGACTGCTTCACCTCGCGGCCCATGGAGAAGACC
ATCCTGCTGTGCTTCTACTTCGCCGTGGGCCTCCTCTCTGCACTCTTCAGCCTGGCGGAG
CTCATCCACGTCTTCACCAAGTGGAGGCGATGGAGGAGGGCGGCCCGGACGGGGGGACCC
CCGGACGAGAAGACGGCTGGACGGAACCAGAGGGACCTCCAGAAACTGACCCAGGTGGCG
GTGGGTGACGAGGGTGGCTTGGGGTTTCAGAGGGACAGGAGTGGGAGTGGGGGTGGGAAA
AGGGGGCAGTTTTTCTCAGGGCGGGGGAGACATGGGGGCACTAGTGGCAGCAGCAGCAGT
GGGGGGGGGAAGGTGAGGGTGTCGCTAGGCAGGAGCAACTCCAGCGTCGGACACAAGACC
TCCAGATACAGTAGCCAGAAGAGCCGCACACAGGTGGTGTGA

>Ch-gjd3like-XM_012837670 Splice site (95.1% id to full-length Ch-gjd3like-XM_012837668 above)
ATGGGGGAATATGAGGTTCTGGGGGGGCTGATTGGGGAGATGCAGACCCACTCCCCCCTA
CTGGGGCGCCTGTGGCTGCATATCATGCTGGTGTTCCGCATTCTCATCCTGGGCACCGTG
GCCTCCGACCTGTTCGATGACGAGCAGGCGGAGTTCGAGTGCAACACGGCGCAGCCGGGC
TGCAAGCAGGTTTGCTACGACCAGGCCTTCCCCATCTCACAGTACCGCTTCTGGGTGTTC
CACATCGTGCTCATCTCCACGCCCGCCCTCCTCTTCATCATGTACGCCATGCACCTGCAC
TCCAAGAGCCAGGCCCGCCAGGAAGATGCCAACGCCACCACTGAGCCACTGATGCAGAAA
CCGGACAAGGGCGAGTTGAACCCGCGACAGGACCGTAACGTGACGCCTCTGTACATGCTG
AACGTGGGGTTCCGCTTCCTGGCGGAGGTGGCGTTCCTGGTGGCTCAGTGGGCGCTGTAC

GGGTTCCGCGTGGAGGCCCGCTTCCCCTGCAGCACGTTCCCCTGCCCCTACACGGTGGAC
TGCTTCACCTCGCGGCCCATGGAGAAGACCATCCTGCTGTGCTTCTACTTCGCCGTGGGC
CTCCTCTCTGCACTCTTCAGCCTGGCGGAGCTCATCCACGTCTTCACCAAGTGGAGGCGC
CGGAAGAGGGCGGCCATGACGGGGGGACCCCCGGACGAGGAGACGTCTGGACGGAACCAG
AAGGAACTCCAGAAACTGACCCAGGTGGCGGTGGGTGACGAGGGTGGCTTGGGGGTTCAG
AGGGGCAGGAGTGGGAGTGGAGGTGGGAAGAGGGGGCATGGGGGCACTAGTGGCAGCAGC
AGCGGTGGGGGGAAGGGGAGGGTGTCGCTGGGCAGGAGCAACTCCAGCGTCGGACACAAG
ACCTCCAGACACAGCAGCCAGAAGAGCCGCACAGGGTTGTTGTTGTGA

>Ch-gjd4-cx40.1-XM_012823059 Splice site
ATGGGGGGCCAATCTGCGTCTGAAGCCATTTTTATTGCTGTCAACCACAACATCACTCTA
GTAGGGAAACTGTGGCTGCTCATTATGGTGTTCCTGCGCATTTTCATCCTCATCTTCGCT
GGATACCCACTCTATCAGGATGAGCAGGAGCGATTTGTGTGCAACACCATTCAGCCAGGC
TGTTCAAATGTGTGTTACGACCTATTCGCTCCCCTTTCCCTCTTCCGCTTCTGGTTGCTT
CAGCTCACCATCCTCTGCCTGCCATATTTGACGTTCGTTACCTACATCATTCACAAAGTG
CTGTCAGATATCGCTGTTTTCTCTGACGCGTCACACAAGATGAAAGCCAGGTCTCTCATT
GGAATCCAGCAGGGATCTCTCCAGAAAGGAGCCCTAAGCAAGGCACGTCACATCCAGGCA
GAGCTCAGCACGTTGAGGACCTTCACTGGAGCTTACATCACCCAGCTGCTCCTCCGGATT
CTTTTCGAGGCTGGCTTTGGGGCGGCTAACTACTATCTATTTGGCTTCTACATCCCCAAG
CGCTTCCTGTGCCAGCAATCACCTTGTACAACTACAGTAGACTGCTATGTCTCCAGACCC
ACAGAGAAAACTGTTATGCTGAACTTCATGTTAGGGACGGCCGGCCTTTCCCTTCTTCTC
AACGGGTTGGACATGATCTGTGCCATCAAGCGCTCCGTGAGGCAGAAGTCCAAGAGGAAG
ATGCTGGTGCGGAATATGTACGAGGAGGAACAGTTCTACCTCTCCCCTGGAGGGAGCCAG
GGAGCCATCGATGCCAACGTTTCCACAGTGGAGGAGATGGTGGCCTCAGTAGCAAGCGGA
AGTTTCCGGAAGAGAGGGATGAGTAAGTCCAGCAGGGCCGATCTGGAGGACGCTCCGTGT
GGTCGAGGGACACCTCTTGTCCCGGGGATGCTGGGTCCTACAAACGCTCACAGTGAGAAT
AATGTCTATCCAATCCCGGCTCTGGAGGAATGCCCGGACCGAGAGGGCAGTGAGGTGGCA
CTGTGTCCCACAGAGCAAATGGGCACGCCCAGACCCATACGGGTCAGTAAACGGAGCCGC
CTGAAACCACCCCCGCCGCCCAGACGGGACAACCCCCCTGGGGCGGGTTCTGTGGACGTG
GTTCCAGGAGCGACGGCACTGTGCACCAGAAGAGTCGGACACTACACACTGGTGGAGATG
AGTGGCGTTGGCCTGCCCTCGTGCAGTGGGGACAACCAGGAGAAAAGGTCAGAGTGGGTC
TGA

>Ch-gje1like-XM_012822376 Splice sites
ATGTCTTTAAACTACATCAAGAACTTTTATGAAGGATGTCTGCGGCCTCCCACTGTGATT
GGCCAGTTCCACACTTTGTTCTTTGGCTCTGTGCGCATGTTCTTCTTGGGGGTTCTGGGA
TTTGCAGTTTACGGAAATGAAGCCCTTCACTTTAGCTGTGATCCAGACAGCAGGGAACTC
AACCTCTTCTGTTATAACCAGTTTCGACCGATAACTCCGCAGGTTTTCTGGGCCTTGCAA
CTGGTGACTGTTCTCGTACCTGGGGCTGTTTTCCACCTCTATGCTGCTTGCAAGAACATT
GACCAGGAGGACATCTTAGAGCGGCCCATCTACACTGTCTTCTACATCATATCTGTGCTC
CTGCGGATCATTCTAGAAGTGGTGGCCTTCTGGCTTCAAAGCCACCTGTTTGGCTTCCAA
GTGCACCCCCTGTACATGTGTGACGCCAGCGCGCTGGAGAAGGCCTACAACTTCACCAAG
TGCATGGTGCCTGAGCACTTTGAGAAGACCATCTTCCTCAGCGCTATGTACATTTTCACC
ATCATCACCGTTGTGTTGTGTGTCGCTGAGATCTTTGAAATACTTTGCAGGAGACTTGGC
TATTTAAGCAGTCCATGA

Suppl. Fig. 10. Atlantic cod (*Gadus morhua*) connexins.

Atlantic cod, *Gadus morhua* (Gm).
Assembly: gadMor1, Jan 2010.
Genebuild: Aug 2011.
Database version: 98.1.

Yellow: Conserved domains as defined by Cruciani and Mikalsen (2007)
Green: Conserved cysteine codons (cysteine signature)
Grey: 15 nt added at the ends of the conserved domains
Other colors are explained where necessary.

The Ensembl gene abbreviation is as follows: ENSGMOG00000009844 = G09844.

```
>Gm-NN-gja1-G09844 Our modification.Underlined: Predicted as intron by Ensembl; here included as part of cds. Lower case letters: Located on an unplaced contig (in the scaffold, there is just a row of Ns). The chromosomal cod assembly in GenBank (GCF_902167405) and the subsequent gene prediction XM_030362165 confirmed that the lower case letter sequence indeed is a likely part of the cds. In fact, XM_030362165 predicts that the lower case sequence should be extended approx 90 nt in 5´-direction.  Splice site.
ATGGGAGACTGGAGCGCTCTGGGGAAACTGCTGGACAAAGTCCAGGCCTACTCCACAGCC
GGAGGCAAGGTATGGCTCTCCGTCCTCTTCATCTTCCGTATCCTGGTCATCGGTACTGCG
GTGGAGTCTGCGTGGGGCGACGAGCAGTCGGCCTTCAAGTGCAACACCGCCCAGCCGGGC
TGTGAGAACGTCTGCTACGACAGCTCCTTCCCCATCAGCCACGCACGCTTCTGGGTCCTG
CAGATCATCTTCGTCTCCACGCCAACGCTGCTCTACCTCTGCCACATCTTCTACCTCATC
CACAAGGAGGAAAAGatgaagtacggcatcgagaagaacggaaaggtgaagatgaaagga
gctctgctcaggacctacatcttcagcatcctgctcaagtccttctttgagGTGGGCTTC
CTACTGCTGCAGTGGCACATCTACGGCTTCAGCCTGGCGTCGCGCTACGAGTGCGAGGCG
TACCCCTGCCCCCACCGCACCGACTGCTTCCTGTCGCGGCCCACCGAGAAGACCATCTTC
ATCGTCTTCATGCTGGTGGTCTCCCTGGTGTCCCTGCTGCTCAACCTCATCGAGCTCTTC
TACGTCACCTACAAGTGGGTCAAGGACACCATGAGGGCGTCCGAGGGCCAGCAGCTCCAC
CCCCGCCTCCGCCTGCTGCCGGGGGCCGGAGGAGGAGTGGGAGGAGGAGGAGTGGGAGGA
GAAGAAGGAGGAGCGCCGTACCACTACTGCAACGGCTGCCCCCCCCCCTCCGCCCCTGTC
TACAACCTGGATGCCACGGCGACGGTGGCAAGGGGCGACTCGGTGAACCACTACAACAAG
ACGGCGAGCGAACAGAACTGGACCAACTTCAGCACGGAGCAGAACCAGCTGGGCCGCTCC
CCGCCCCGTCGCCACGGCAGCCAGCGCAGTACCGGCAAGAACAACAACAACAACAATAAC
CACAACAACAAGGCCGGCGCCAACGCCAGTGACTGCAACCGCGACAGCCCTGCCTTCCTG
GGCCCCGCCTTCGTAGGCCCCGCCCACGGCCAGCCACCGCCGTCGGACAAGGTGGAGACC
AAGGAGCTTCACCTCCTCCGGGGGCTGGAGCCGCGGCCCCGGCAGCCGCGCCTCCAGCCGC
GCCCGCACTGACGACCTGGACATCTGA
```

```
>Gm-cx43-G20304 Our modification. Extended in 3'-direction. No reasonable stop codon in frame, but in other reading frames, there are translated sequences that become reasonable similar with other GJA1 orthologs. Hence, potential small intron or sequencing error towards 3'-end.
ATGGGTGACTGGAGTGCTCTGGGCCGCCTGCTGGACAAGGTCCAGGCCTACTCCACCGCT
GGGGGGAAGGTGTGGCTCTCCGTCCTCTTCATCTTCAGGATCCTGGTCCTTGGGACGGCC
GTGGAGTCCGCCTGGGGCGACGAGCAGTCGGCCTTCAACTGCAACACTCAGCAGCCCGGC
TGCGAGAACGTATGCTATGACAAATCCTTCCCCATCTCCCATGTGCGCTTCTGGGTGCTG
CAGATCATCTTCGTGTCCACGCCCACGCTGCTGTACCTGGCCCACGTCTTCTACCTGATG
AGGAAGGAGCAGAAGCTGAACAGGAAGGAGGAAATGCTGAAGGCCGTGCAGAACGATGGC
GGCGACGTTGACATCCCGCTGAGGAAGATCGAGATGAAGAAGCTGAAGCACGGCCTGGAG
GAGCACGGCAAGGTGAAGATGAAGGGCGCCCTGCTGAGAACCTACATCGTCAGCATCTTC
TTCAAGTCCATGTTCGAGGTGGGCTTCCTGGTCATCCAGTGGTACATATACGGCTTCAGT
CTGGCAGCGGTGTACACCTGCGAGAGAGAACCCTGTCCCCACAGGGTGGACTGTTTCCTG
TCTCGGCCCACAGAGAAGACGGTGTTCATCATCTTCATGCTGGTGGTGTCGCTGGTGTCC
CTGCTGCTCAACGTCATCGAGCTCTTCTACGTGTTCTTCAAGAGGATCAAGGACCGTGTG
AAGGGCCGCCAGCCGCCCACCCTCTACCCCAGCGCTGGCACCCTGAGCCATACCCCCAAA
GATCTTTCCACAGCCAAGTACGCCTACTACAATGGCTGCTCCTCCCCCACCGCCCCGCTC
TCGCCCATGTCCCCGCCGGGCTACAAGCTGGCCACGGGCGAGCGCGGTACCGGCTCATGT
```

CGCAACTACAACAAGCAAGCCACCGAGCAGAACTGGACCAACTATTCCACGGAGCAGAAC
CAGCTGGGCCAGCACGGCGCGGGCAGCACTATCTCAAACTCCCACGCGCAGGCTTTTGAT
TTCCCCGACGATACGCACGAGCATAAGAAACTGACGTCATCCGCAGCTGCACACGAGATG

>Gm-NN-gja3-G09100-2 Our modification. Splice sites. This Ensembl prediction contains two separate and unique connexins sequences, the present and a cx30.3 sequence.

atgggtgactggagctttctgggacgccttctggagaatgctcaggaacactcaactgtg
atcggcaaggtgtggctgaccgtcctcttcatcttccgcattctggtgctgggcgcggcc
gcagaggaggtgtggggagacgagcagtcggacttcacctgcaacacgcagcagcccggt
tgcgagaacgtctgctacgaccaggccttccccatctcccacgtgcgcttctgggtgctg
cagatcatcttcgtgtccacgcccacgctcatctacctgggccacgtgctgcacatcgtg
cgcatggaggagaagcggcgtgagaaggaggaggagctgcggaaggcgggctggcgcagc
gaggagctcctcgggcaNNNNGGAGGCGGGAAGAAGGAGAGGCCGCCGATCCGCGACGAG
CACGGGAAGATCCGCATCCGCGGGGCGCTGCTCCGGACCTACGTCTTCAACATCATCTTC
AAGACCCTTCTGGAGGTGGGCTTCATCCTGGGCCAGTACTCCCTCTACGGCTTCCGCCTC
AAGCCGCTGTACAAGTGCGGCCGCTGGCCTTGCCCCAACACGGTGGACTGCTTCATCTCC
AGGCCCACTGAGAAAACCATCTTCATCATCTTCATGCTGGTGGTGGCCTGCATCTCCCTG
CTGCTCAACCTGCTAGAGATGTACCACCTGGGCTGGAAGAAGGTCAAACACAGCGTCACC
CACAAGTTCGCGGCTGACTGCGGGTCCCTGCGGCTGGGCCCCGGCGACGACGCCGGCGAC
CCCCGGGCGGTCCCCGAGTGCGCCACCCTGGTTTCGGACCACTGCCTGCAAGGCTACACC
GGCAGGAGCACCATGGAGCGGGTCCGCTACCTGCCCGTCCAGAACTCCTC

>Gm-gja3-G04087 Our modification. Ensembl-predicted introns are included (underlined). There is probably an intron or something wrong in the 3’-end (after the conserved domain), but we have not tried to solve the problem here. In the first conserved domain at the position indicated by lower case “ga”, the Ensembl sequence indicates a row of approx. 100 Ns. “ga” has been found by Blast against GenBank cod wgs.

ATGGGCGACTGGAGCTTTCTGGGCCGGCTTCTTGAGAACGCGCAGGAGCACTCGACGGTG
ATCGGCAAGGTCTGGCTCACCGTCCTCTTCATCTTCCGCATCCTAGTGCTGGGTGCCGCA
GCAGAGGAGGTGTGGGGCgaCGAGCAGTCGGACTTCACCTGCAACACGCAGCAGCCCGGT
TGCGAGAACGTCTGCTATGACCAGGCCTTCCCCATCTCCCACATCCGCTTCTGGGTGCTG
CAGATCATCTTTGTGTCCACTCCCACGCTCATCTACCTGGGCCACGTGCTGCACATCGTG
CGCATGGAGGAGAAGCGCAAGGAGAAGGAGGAGGAGCACCGCAAGGTCAGCGGGTTCCCC
GATGACAAGGAGCTGCCGTACCGGAACGGGGGCGGCGGTAAAAAGGTGAAGCCGCCGATC
AGAGACGAGCACGGCAAAATCCGCATCCGCGGGGCCTTGCTGCGTACCTACGTGTTCAAC
ATCATCTTCAAGACTCTGTTTGAGGTGGGCTTCATCCTGGGCCAGTACTTCCTGTACGGC
TTCTCGCTGCGGCCGCTCTACAAGTGCTCCCGTTGGCCGTGCCCCAACACGGTGGACTGC
TTTATCTCCAGGCCCACGGAGAAGACTATCTTCATCATATTCATGCTTGTTGTGGCTTGT
GTGTCGCTTTTACTCAACCTGCTGGAGATCTACCACCTGGGCTGGAAGAAGCTGAAGCAG
GGCGTGTACCACCCCGACCACCTGCTGCGGGCCGCCGGCCAGCTGGCCACGCCGGAGGGC
GTGGCCTCGCTAGGGGCCCCGGCTCTCCTCAACTACCCCCCCACCTACAGCCACATAGCG
GCCGGCATGGGGTCCCCCACCGACGCCGAGTTCAAGATGGAGGAGCTCCAGCGGGAGGAG
GGGGCGCGGACGCCTCCCCCGACTCCCCCGGCCGCCCACTACTACATCAGCAGCAACAAC
AACCACCGTCTGGCCGCAGAGCAGAACTGGGCCAACCTGGCCACCGAGCAGCACACCCGC
CAGATGAAGGCCACCTCCCCCACCCCCACGTCCTTCTCCTCCTCAAGCAGTGAAGCGGCC
CCGCCCTGCTCAACTAGCCCCACCCCCTTAATGGCAACCCCGGGCAACGCTGCAGCCCCC
GGTGATGTGGCGACCAGCGGCGACGGAGCCGGCCTGACCCCCGAGCCGGGCCAGCGGGAG
GAAGAGGATGTCACCATGGCGACGGTGGAGATGCACCTGGAGGGGGTGTTCCCGGACCCC
CGGCGTCTTAGCAGAGCCAGTAGAAGCAGCATCCGCGCCCGGCACGATGACCTCGCCATC
TGA

>Gm-NN-cx39.9-G20599 Ensembl prediction. No modifications

ATGGCCGACTGGAACCTGCTAGGGAAGCTGCTGGAGGCCGCTCAGACACACTCCACCGTG
GTGGGCAAGGTGTGGCTCACCGTGCTCTTCATCTTCCGCATCCTGGTTTTGGGCACGGCC
GCCGAGAAGGTTTGGGGCGACGAGTCGTCGGGCTTCACCTGCGACACCAAGCAGCCTGGC
TGTCAGAACGTCTGCTACGACCGTACCTTCCCCATCTCCCACATCCGCTTCTGGGTGCTG
CAGATCATCTTCGTGTCCACGCCCACGCTCATCTACCTGGGCCACATCCTGCACCTGGTG
CGCATGGAGGAGAAACAGACGCAGAAGGAGAAGGACCGCGAGGAGGAGGAGCCGCCGGCG
GCCGAAGGTGCAGGAGGAGGAGGAGGAGGAGGAGGAGGAGGCAAGGCCAAGAAGGGGCCG
GTGAAGGACGGCCAGGGCCGGGTGCGTCTACAGGGGGAGCTGCTGCGGACGTACGTCTTC
AACATCATCTTCAAGACGCTGTTCGAGGTGGGCTTCCTCGTGGCCCAGTACATGCTGTAC
GGCTTCCAGCTGAAGGACATGTACACCTGCGACCACTGGCCCTGCCCCAACATGGTGAAC
TGCTACATCTCGCGGGCCACCGAGAAGACCATCTTCATCCTGTTCATGCTGGTGGTGGCC
TGCGTGTCGCTGCTGCTCAACCTGGTGGAGATCTGCCATCTGGGCTTCACCAAGTTCCAA
AAGGGCCTCTGCTTCCTCTGCCAACGCCCGAAGAACGCGGAGCCCAGCGAGACACCCAAC

TACAACGACTACACGGAT

>Gm-cx39.9-G14144 Our modification. Ensembl-predicted introns are included (underlined). Has been extended by Blast into GenBank cod wgs (lower case).
ATGGCCGACTGGAACTTGCTGGGGAAGCTGCTGGAGAGCGCCCAGACGCACTCCACCGTG
GTGGGCAAGGTGTGGCTCACCGTGCTCTTCATCTTCCGCATCCTGGTTTTGGGCGCAGCC
GCCGAGAAGGTTTGGGGCGACGAGTCGTCGGGCTTCACCTGCGACACCAAGCAGCCTGGC
TGTGAGAACGTCTGCTACGACCGCACCTTCCCCATCTCCCACATCCGCTTCTGGGTGCTG
CAGATCATCTTCGTGTCCACGCCCACGCTCATCTACCTGGCCACATCCTGCACCTGGTT
CGCATGGAGGAGAAGCAGCAGCAGAAGGAGAAGGACCGCGAGGAGGAGCACGCACTGCAC
AGCGAGAAGCAGGAGCTGCTGGCGGCCGAAGGAGGAGGTGGAGGCAAG**G**CCAAGAAGGCG
CCGGTGAAGGACGGCCAGGGCCGGGTGCGTCTGCACGGGGTGCTGCTGCGGACGTACGTC
TTCAACGTCATCTTCAAGACGCTGTTCGAAGTGGGCTTCATCGTGGCGCAGTACCTGCTG
TACGGCTTCCAGCTGAAGGCCATGTACACCTGCGACCGTTGGCCCTGCCCCAACACGGTC
AACTGCTATATCTCGCGGCCCACCGAGAAGACCATCTTCATCCTGTTCATGCTGGTGGTG
GCCTTCCTGTCGCTGCTGCTCAACCTGGTGGAGATCTACCACCTGGGCTTCACCAAGTGC
CACCAGGGCCTCCGCTTCCGGCGCTCGCGCCAGAGGAAGGCCGAGCTGACCCGGATGCCC
AGCGAGGCGCCGGCCGTGATGCACTTTGTCCCCAACTACAACTACTACACCGCGCACGCG
CACGCGGCGCGCGgggggcgggcggcggcggtggcggggaggcctttcagggcgactcc
agctacggcctggccgagcccggcggcgccgcctacagcaacccctacagcagcaaagcc
gtgtccaagcagaaccgcgacaacctggcggtggagcggcgggcggagagcgacgacgcc
ggcggcggcggcggcggcgggtccttcgagagaccggcggagaacaagcgacgcaac
agccagacgagcaaacacagcaacactaagacgcgcggcgacgatctgaagatctag

>Gm-NN-cx39.9-G20196 Ensembl prediction. No modification.
ATGGGGGACTGGAACCTGCTCGGCAAGCTCCTGGAGAACGCCCAGGAGCACTCCACCGTG
GTGGGCAAAGTGTGGCTCACCGTCCTCTTCATCTTCCGCATCCTCATCCTCAGTGCGGCC
ACTGAGAAGGTGTGGGGCGACGAGCAGTCGGGCTTCACCTGCGACACCAAGCAGCCCGGC
TGCGAGAACGTGTGCTACGACGTCACCTTCCCCATATCCCATGTGCGCTTCTGGGTCCTG
CAGATCATCTTCGTCTCCACGCCCACGCTCATCTACCTGGGACACATCCTCCACCTGGTG
CGCATGGAGGAGAAGCACCAGCAGAGGGACAAGCTGAAGGAGCAGCCCGGGGAGAAGCAG
GGCCTGATGGAGGTGGCCAAGCCCAAAAAGCTGGTGCGGGACGACCGGGGTCGCGTGCGC
CTGCAGGGGGAGCTGCTGCGCACCTACGTGTTCAACATCATCTTCAAGACCCTGTTCGAG
GTGGCCTTCATCGTGGCGCAGTACCTCCTGTACGGCTTCGAGCTGAGGCCCATGTACACG
TGCGACAAACACCCCTGCCCCAACACGGTCAACTGCTACATCTCCCGGCCCACCGAGAAA
ACCATCTTCATCATCTTCATGCTGGGCGTGGCCAGCGTGTCGCTGCTCCTCAACCTGGTG
GAGATCTACCACCTGGGCTTCACCAAGTGTCGCCAGGGCATCAGCTACCGGAGGGGGCGG
CTGCTGGCCGCCGCAGCCGCCGCGGAGGCTAAGTCCAAGGAGCTCAGCGACGCGGTGGCG
CCCTTCGCGCCCACCTACGACGACTACTTCCACGGGCACCACCAGGTGTCGCCGGCGTAC
CCGCCGGTGCCCGGCTACAACGTCTCCCCCCTGTCGGAGGAGACAGACTCTCCCTTCCAG
CCGTACCACAGCAAGGCCGCCTACAAACAGAACAAGGACAACCTGGCGGTGGAGAGGAGC
GGCAGCAGCAGGCCCGAGGAATGCGACCCGAGAGGTAAGAGTACGGGCGCGGGGTCGGCG
CCGGGGTCACCGGGCCTGGCGAGGTCCAGCGGCGGTGGTCGTCATGGCAAGCACAGCAAC
AACAAGACTAGAATAGACGATCTGAAGGTCTGA

>Gm-cx39.4-G20255 Our modification. Extended in both 5’ and 3’-directions (underlined).
ATGTCCCGGGCCGACTGGTCCTTCCTGGAGCACCTGCTGGAGGAGGGCCAGGAGCACTCG
ACGCGCGTGGGCCGCGTGTGGCTCACCGTGCTCCTCCTGTTCCGCATGCTGGTGCTGGGC
GTGGCCGCCGAGTCGGCCTGGGACGACGAGCAGTGCAACTTTGTGTGCAACACGGAGCAG
CCCGGCTGCGAGTCGGTGTGCTACGACCGCGCCTTCCCCATCTCCCACTTCCGCTACTTC
GTGCTGCAGGTCATCTTCGTCGCCACGCCCACCATCTTCTACTTCGGCTACCTGGCGGTG
CGCACGGCCAAGGACCAGTCCGAGGAGGAGGAGGCGGAGGAGGAGGACGGGGTGGCGGAG
GGGGAGAGGGCGGCGCAACAAGAGGGGCGGGGCCGAAGCGCGGCGGTGGCGTCCTCCGGG
GCGAGGGCGGCTAGGAAGGGCAAGGGACTGGAGGTCATCCAGGAAGAGGGGGGCGAGGAA
GAGGGCTTGCGAGAGAACGCCGGCAAGGCCGCCAAGGAGGCGTCGGAGTCTCCGAAGCTG
AAGGGGAGGCTGCTGGGGGTGTACACCGTCACCATCTCCGTCACCGTGCTCCTGGAGGCG
GGCTTCATTGCCGGCCTGTGGGTGCTGTACGACGGCTTCGTGATCGCCGCGCGCTACGAG
TGCGTGGGGCTGCCCTGCCCCCACACGGTGGACTGCTTCGTGTCGCGGCCCACCGAGAAG
ACCATCTTCACCATCTACACGCAGGCCATCGCCGGCCTCTCCCTGCTGCTGAACCTGCTG
GAGCTCCTCCACCTGCTCCAGCTGGCCGTCTCCCAGCGACTGGAGAAGCGCTACCGCCGC
GGCGCCACCTCGCTGCCGTCGCCGCGGTGGCGGCGGCGGCAACCGGGGGGGAGACCACAC
CCCGATCCAACTTCCGGCGCAAGAAGCCCCTTCGTACTGCCCCGCCGTCCCCGGAGAGGG
CTACCCCGAGCCCCCCGGGAGCTACGTGGTGGTGGACACCCTGAGGTCCGAGGTGAACTG
GGGGGCCGGCGGGGGAGCGGGGAACGGCGAGGGCGACATCCCGCCCAGCTACCTGAACTG
CTTGGGCGGGATGCGCAGCACACATTCCCCCAGGGCCCACGTGAAGAAGCACGTCCACGG
CAACGGGAAACACAGAAAAGACAACCATAA

>Gm-GJA5-G04028 Our modification. Ensembl-predicted introns are included (underlined). Runs into row of Ns.

ATGGGGGATTGGAGCCTGCTGGGTAACTTCTTGGAGGAGGTGCAGGAGCACTCCACGTCA
GTGGGCAAGGTGTGGCTCACCGTGCTCTTCATCTTCCGCATCCTGGTGCTGGGCACGGCC
GCCGAGTCGTCCTGGGGCGACGAGCAGATCGACTTCCTGTGCGACACGCTGCAGCCCGGT
TGCACCAACGTGTGCTACGACAACGCCTTCCCCATCGCCCACATCCGCTACTGGGTGCTG
CAGATCGTGTTCGTGTCCACGCCGTCGCTCATCTACATGGGCCACGCCATGCACATTGTG
AGGCGCGAGGAGAAGCGCCGGCGCCGGCAACAGGAGGGCCAGGAGGAGGAGGAGGAG
GACGACGACGAGGACGGAGGAGGAGGGGGAGGAGGCGGGGGGAAGAGGCACGACGCGGAG
CGCGAGAAGGAGTACCTCCAGCAGAAGGAGAGCGGGAAGGGCGAGGGCATGGGCCGCGTG
CGCTTGAAGGGGGCGCTGCTGCAGACCTACGTCCTCAGCATCCTGATCCGCACGGTGATG
GAGGTGACCTTCATCACCGTGCAGTACCTGATCTACGGCGTGTTCCTGAAGGCGCTGTAC
CTCTGCAAGGCCTGGCCCTGCCCCAACCCCGTCAACTGCTACATGTCGCGGCCCACCGAG
AAGAACGTGTTCATCGTGTTCATGCTGGTGGTGGCGGGCGTGTCCCTGCTGCTGTCCGTG
GTGGAGCTCTACCACCTCGGCTGGAGGCGCGTCCGGAAGTGCCACCGC

>Gm-gja8a-G19707 Ensembl prediction. No modifications.

ATGGGCGACTGGAGCTTTCTGGGGAACATTTTAGAGGAAGTGAACGAGCACTCGACGGTG
ATCGGCCGGGTGTGGCTCACGGTGCTCTTCATCTTCCGCATCCTGATCCTGGGGACGGCG
GCCGAGTTTGTGTGGGGCGACGAGCAGTCCGACTACGTGTGCAACACCAACCAGCCCGGT
TGCGAGAACGTGTGCTACGACGAGGCCTTCCCCATCTCCCACATCCGCCTGTGGGTGCTG
CAGATCATCTTCGTGTCCACGCCGTCGCTGGTCTACGTGGGCCACGCCGTGCACCATGTC
CACATGGAGGAGAAGCGCAAGGAGCGCGAGGAGGCGGAGCTCAGCCGCCAGCAGGAGCTG
AGCGAGGAGCGGCTGCCGCTGGCGCCCGACCAGGGCAGCGTGCGCACCACCAAGGAGACC
AGCACCAAGGGCAGCAAGAAGTTCAGGCTGGAGGGCACCCTGCTGCGGACGTACATCTGC
CACATCATCTTCAAGACGCTGTTCGAGGTGGGCTTTGTGGTGGGCCAGTACTTCCTGTAC
GGCTTCCACATCCTGCCGCTGTACAAGTGCAGCCGCTGGCCCTGCCCCAACATCGTGGAC
TGCTTCGTGTCGCGCCCCACCGAGAAGACCGTCTTCATCATCTTCATGCTGGCCGTGGCG
TGCGTCTCGCTCTTCCTCAACTTCGTGGAGATCAGCCACCTGGGCCTGAAGAAGATCCGC
TTCGTGTTCCGCAAGCCGGCGCCGGCCCCGGCGCCCGGGGAG

>Gm-NN-gja9-G09903 Our modification. Underlined: Extension of sequence into Ensembl-predicted intron. Splice site. Lower case letter: Found by Blast in GenBank cod wgs.

ATGGGGGACTGGAACTTCCTGGGGGGGATCTTGGAGGAGGTCCACATCCACTCCACCACG
GTGGGCAAGATCTGGCTGACCATCCTGTTCATCTTCCGCATGCTGGTCCTGGGCGTGGCG
GCCGAGGACGTGTGGAACGACGAGCAGAGCGCCTTCGTCTGCAACACGGAGCAGCCGGGC
TGCAGGAGCGTCTGCTACGACCGCGCCTTCCCCATCTCCCTGATCCGCTACTGGGTGCTG
CAGgtgatcttcgtgtcggcccccctcgctggtctacatgggccacgccctgtaccgcctg
cgggcgctggagaaggtccgccagcgccgcaaggcctcctccgccgccagctggagctgc
tggacgcggcggcggcgtcggcggcggcgcggcggcggcgcggagcggcaggtgaaGCAG
GTGGAGCAGGGCCGGCTCAACAAGGCGCCGCTGCGGGGGGCGCTGCTGCGCACCTACGTG
GCGCACGTGTTCACGCGCTCGGCCGTGGAGGTGGGCTTCATGGCGGGCCAGTACCTGCTG
TACGGCGCGCGGCTGCGGCCGCTGTACCGCTGCGAGCGCGCCCCCTGCCCCAACGCCGTG
GACTGCTACGTGTCGCGGCCCACCGAGAAGAGCGTGTTCATGGCGTTCATGCAGGCCATC
GCGGGGGTCTCCCTGCTGCTCAACCTGCTGGAGATCCTGCACCTCGGCTACAAGAAGCTC
CGCAAGGTCTTCAGTACGG

>Gm-cx52.9-G20571 Ensembl prediction. Ensembl sequence runs into a row of Ns, and this part has been extended by Blast into GenBank cod wgs (lower case).

ATGGGGGACTGGAACTTCCTGGGCGGGATCCTGGAGGAGGTCCACATCCACTCCACCATG
GTGGGCAAGATCTGGCTCACCATCCTCTTCATCTTCCGCATGCTGGTCCTGGGCGTGGCG
GCGGAGGACGTGTGGAACGACGAGCAGTCCGACTTCATCTGCAACACGGACCAGCCCGGC
TGCCGCAACGTGTGCTACGACCGCGCCTTCCCCATCTCCCTGATCCGCTACTGGGTGCTG
CAGGTCATCTTCGTGTCCTCCCCGTCCCTCGTCTACATGGGCCACGCCATCTACCAGCTG
CGTGCCCTGGAGAAGGAGCGCCACTGCAAGAAGTCGGCGCTGCGGCGCGAGCTGGAGGCG
GTGGAGGCGGAGCACGCGGAGGTGCGGCGCCGGATCGAGCGGGAGATGCGGCAGCTGGAG
CAGGGCAAGCTGAACAAGGCGCCGCTGCGGGGCTCGCTGCTGCGCACCTACGTGGCCCAC
ATCGTGACGCGCGCCCTGGTGGAGGTGGGCTTCATGTCGGGCCAGTACCTGCTCTACGGC
CACCGCCTGGACCCGCTGTTCAAGTGCGAGCGGGAGCCCTGCCCCAACCTGGTGGACTGC
TTCGTGTCGCGGCCCACCGAGAAGACGGTGTTCATGATGTTCATGCAGGCCATCGCCTGC
ATCTCGCTGTTCCTCAGCGTGCTGGAGATCCTGCACCTGGGCTACAGGAGGCTGAAGAAG
GGCCTGCTGGACTACTACCCCCACCTCAAGGACGACCTGGACGAGTACTACGGCAGCAAG
TCCAAGGAGAACTCGACGGCGCACCAGGTGTGCACGGGCGCCTCGGCGGGCCGCAAGCCC
ACCCTCCCCACCGCGCCCAGCGGGTACACGCTGCTGCTGGAGAAGCAGGGCAACGGGCCG
ACCTACCCCCTCCTCAACACCtccccgccttcgtccccgcgctccccccggagctcggc
gggggggcggagccggccccgggagccgcaacgaggcggcgtggcggcggcggtgttgcc
ccggtccacggagcagaacagcaactccaacaacacgggcgtcgagccgcgctccccgcc

cgcggacaagcaggcccggcccgaggggcttctggcccgggggccggccctccccgggga
cacggagtgcgggggctccgagtaccccaccttccccgtcagagacacctcctcgtgccc
ctccctggcggggaaccccgtgaggaagatccgccgcgccagcccgccctggaactgctc
cacggtgcaggagggcaacgtgtccgacagcggggactcgtacccggggaacgtccacgg
gaaagtccggggctcctcctgcgggccccgcacccggaccgtcgccaagtcggaggccaa
gaggccgagccggtcccagagcccggactctgtgggagagctgagctcggcgtcgcggca
cagccgggagagcaacagcccccccgtcgcctcctcccccaaccgccgcacctcgggggc
tag

>Gm-NN-gja10-G02098 Our modification. Sequence extended in 3’-direction (underlined). The very 3´end (lower case letters) is taken from XM_03035622, which otherwise differs only one nucleotide from the present sequence.
ATGGGTGACTGGAACCTGCTGGGCAGCATCCTAGAGGAGGTCCATGTCCATTCCACCATC
GTGGGCAAGATCTGGCTTACCATCCTCTTCATCTTCCGCATGTTGGTGCTGGGGGTGGCT
GCCGAAGATGTGTGGGAGGACGAGCAGACAGAGTTTGTGTGTAACACTGACCAGCCGGGC
TGCAAGGCTGTGTGCTACGACCGTGCCTTCCCCATCTCCCTCGTTCGCTTCTGGGTGCTG
CAGGTCATCTTCGTGTCGGCACCCTCTCTGGTCTACATGGGCCATGCCCTCTACCGCATC
CGCTTCCTGGACAAGGAGCGCCACCGGCGACGTGCCCAGCTGCGCGACGAGCTTGGCGAG
CCGGAGGTGGCCCTGGAAGAGCACCGGCGCCTGGAGAGGGCGCTGCGTCGGCTGGAGGAG
CAGCGCCGGGTGAAGAAGGTGCCCCTCAGAGGCTCCTTGCTGAGGACCTACATCATCCAT
ATCCTCACACGCTCCGTGGTGGAGGTGTGCTTCATCCTGGGCCAGTATGTCCTCTATGGC
CTGCGCCTGGAGCCTCTCTACAAGTGCGAGCGGCTGCCGTGCCCCAACAGCGTGGACTGT
TACATCTCCCGGCCCACCGAGAAGACCATCTTCATGGCCTTCATGTTCGTCATCGCCGCC
GTCTCGCTTTTCCTCAACCTTCTGGAAATATCCCACCTGGGAGTGAGGAAAATCAGGCAG
ACGATATACGGAGATAAGTACTCCGAAGAGGACAGCTTGATTTACAAGCCCAAGAAGAAG
CGCACCTTGCAGCACCTTTGTCTTATGCGCCACTTGTCGCCTCATAACGGACCGTCAACT
CAAACCTTATTTAAGGGGATTCCTGAGGGAGGGACGAACGCAGTGCATCAGAACATAGGG
CTCAGAGCTAACCAGCAGACAACGACAACAACAACAACCAGACATAACAGCCTGGCTCCT
CTTGGACACTATCAGACAAACTGCATCGCACCTGGATCAATGGTACCACAGAACCAATAC
CAGACCGGGCAGGAGGGGGCCACTCAAGGCCTCCAAACCCATGAAGGCCCAGAGACAAAC
TCCAGGGCCTTGATGGACCAACACCCTCTGGTCTGGCCTGCGGTTCCTTGTAATGTAGAA
GGCTGGCCAAAGGAGCAGACTCAATACCCTGAGGAGCTCCTTCGACCCATGGAGCCTCCT
CGAGTGCTCCAGACTCAGGGGTACACAATGGCCCACAGGCCCAGCCTCGCAGCCAGGGAC
ATGGAGGAGGATGTGGAGAGAAAGTACTCGATAGGCAGTGACCTCTTTCATCTGAACCAC
AGGAAGACCAGCTTCATGGTAAAGCCTCCGTCCGAGAGCATGTCCACCATCAGCGGCTCA
AGCAGCCCCTCGATCCATTCGTCTGAGGAATCTGATGAGCTGGGCTCTCTGCAGGGGGAC
ATGCCTATGATGCCCCCTGCTGGAGGGCGCAGGATGtccatgagtgtgttcctggatatc
tcctcaatcatga

>Gm-cx52.6-G05425 Our modification. Ensembl-predicted intron included in sequence (underlined)
ATGGGGGACTGGAACCTACTGGGTAGCATCTTGGAGGAGGTCCACGTCCACTCCACCATC
GTGGGGAAGATCTGGCTGACCATCCTCTTCATCTTCCGCATGCTGGTCCTGGGCGTGGCC
ACGGAGGACGTGTGGGACGACGAGCAGAGCGAGTTCGTCTGCAACACCGAGCAGCCCGGC
TGCAAGAACGTGTGCTACGACCAGGCCTTCCCCATCTCCCTGATCCGCTAC**TGGG**TGCTG
CAGATCATCTTCGTGTCCTCGCCCTCGCTGGTGTACATGGGCCACGCCCTCTACCGCCTC
CGGGCGCTGGACAAGGAGCGCCACCGCAAGAAGGCGTGGCTGAAGGCGGAGCTGGACGGC
GGCGAGCCCCTCCAGGAGGACCAGCACCGCAGGATGGAGCGCGAGCTGCGGCGGCTGGAC
GAGCACCGCAAGGTGAGGAAGGCCCCCCTGCGGGGGGCGCTGCTGCGCACCTACGTCTTC
CACATCCTGACGCGCTCGGTGGTGGAGGTGGGCTTCATCGTGGGCCAGTGCGCGCTCTAC
GGCATCGGCCTGGCGCCGCTCTACAAGTGTGAGCGCGACCCGTGTCCCAACAGCGTGGAC
TGCTTCGTGTCGCGGCCCACCGAGAAGAACATCTTCCTGGTGTTCATGCTGGTGATCGCC
GGGGTCTCCCTGATCCTCAACCTGCTGGAGATCTTCCACCTCGGCCTGAAGAAGATCAAG
GACAGCCTGTACGGCTCCAAGTACGGCGACGAGGACAGCGTCTGCCGCTCCAAGAAGAAC
TCCCTGGCGCACCCCGCCTGCCACCTGTCCAACTCCTCCCCCCCGCGGACGCTGCACCTC
GCCCACACCGCCTCCGGCTGCCTGGCCCACGACGGCCAGCCGGGCGGTCGCCCTTCCACC
CGGGGCGCGGCGGGGGCCCCCAGCACCACCCGGACGGGGCCCCCTTCGACACCAACGCCC
CGGGAGGCGCCTCCGACCAGCGGGCCCGACCGCTCCCCGTCTGCCTGCACCAGCTGGGGG
CGGTGGGGCGCCGCTACACCCTGGATGACCCCCGGAAGCCCTCGTGCAGCAGCGAGGAGT
CGGCCGGGGCCCAGGGCGCGGGGCCCCAGAGGTACGCCGGGGCCCAGCCGAGGGCCACCC
TCACCGAGCTCCCGGCCGCCCTGCGGAGCGCCCAGCGCAAGCAGAGCCGCGTGA

>Gm-cx28.9-G18912 Our modification. Sequence extended in 3’-direction (underlined). Splice site.
ATGGGTGAGTGGAGTTTCCTAGCGTCTCTCCTTGACAAGGTCCAGTCCCACTCCACGGTC
ATCGGGAAGGTCTGGCTCACAGTGCTCTTCATTTTCCGGATCATGGTCCTCGGGGCCGGA
GCAGAAAAGGTGTGGGGTGATGAGCAATCCCAAATGATCTGTAACACCAAGCAGCCAGGC

TGCAAGAACGTGTGCTACGACCACGCCTTCCCCATCTCCCACATCCGCTTCTGGGTGCTG
CAGATCATCTTCGTGTCGAGCCCCACGCTGGTGTACCTCGCTCACGTCCTCCACGTTATC
CACAAGGAGAAAAAGCTGAGGGAGCGTATGCAGACCAGCAGCGAGCCGACCAAGAACCCA
AAATACTCGGACGACAAAGGTCACGTCAAGATCAAAGGGGACCTTCTGGGCAGCTACCTG
GCCACCATCTTCTTCCGGATCCTTCTGGAGGTGGCGTTCATCGTAGGGCAGTACTATCTG
TACGGCTTCGTCATGGACCCCAGAGTGGTGTGCTCCAGAGCACCCTGTCCCTTCACTGTG
GAGTGCTACATGTCCCGGCCGACCGAGAAGACCATCTTCATCATCTTCATGCTGGGGGTG
TCCTGCGTGGCCCTGCTGCTCAACGTTCTGGAGGTCTTCTACCTGCTGTGCAGAGGCAGG
TGCTCCAAGAGACGGCACGTGGCCCCCTTTACCATGCCCAGCCACTCGGCTGTCCTGGAG
ATGAAGCAGGTGCCCCGGACCACCTAA

>Gm-cx32.3-G18903 Our suggested modification. Ensembl-predicted intron included in sequence (underlined)
ATGGGTGATTGGGGCTTTCTATCCTCCCTGCTGGACAAGGTCCAGTCCCACTCCACGGTT
ATCGGGAAGATCTGGATGAGCGTGCTGTTCCTGTTCCGGATCATGGTGCTGGGGGCAGGG
GCGGAGAGCGTCTGGGGCGATGAGCAGTCGGGCTTCCTCTGCAACACTCAGCAGCCCGGC
TGCGAGAACGTGTGCTACGACTGGACCTTCCCCATCTCACACATCCGCTTCTGGGTGCTG
CAGATCATCTTCGTGTCGACACCAACGCTCATCTACCTGGGCCATGTCATGCATGTCACC
CACAAGGAGAACAAGATGAGGGAGAATCTGGCCAGCCCCGGGTGCGCCAGCACGCAGAAG
CACCCCAAGTACACCAACGAGAAGGGCAAGGTGAAGATCAAGGGCAACCTCCTGGGGAGC
TACCTAGCCCAGCTGGGCGCCAAGATCATCATCGAGGCCGCCTTCATCGTGGGCCAGTAC
TATCTGTACGGCTTCATCATGGTCCCCATGTTCCCCTGCTCCAAGAAACCCTGTCCCTTC
ACCGTGGAGTGCTACATGTCCCGGCCCACCGAGAAGACCATCTTCATCATCTTCATGCTG
GTGGTGGCCTGCGTGTCCCTGCTGCTCAACGTTCTGGAGGTCTTCTACCTGCTGGTGAGC
AGGAGCAGATGTTCCCCCAGGAAGCGCTCGCACATGATCACGTCCGCTCGGCACCCGGCA
CAGCTCTCAGGCCCCATGTGGCCAACGGCAGAAGACGCCCGGCAGGCCAACAAGATGAAC
ATGGACTTTGAGAGCGGCCAGAGTACTGCTGGGAGCCTCAACGGGGCCAAGGAGGAGAAG
AAGCTTCTGAGTGGTCACTAG

>Gm-NN-gjb1-G14169 Our modification. Ensembl-predicted intron included in sequence (underlined)
ATGAACTGGGCGTCCTTCTACGCGGTGGTGAGCGGCGTGAACCGTCACTCCACTGGCATC
GGCCGCATCTGGCTGTCGGTGCTCTTTATCTTCCGAATCCTAGTGCTGGTGGTGGCGGCC
GAGAGCGTGTGGGGCGACGAGAAGTCGGGCTTCACCTGCAACACGCAGCAGCCCGGCTGC
AACAGCGTCTGCTACGACCACTTCTTCCCCGTGTCCCACATCCGCCTGTGGGCGCTGCAG
CTCATCCTGGTGTCCACGCCGGCGCTGCTGGTGGCCATGCACGTGGCCCACCGCCGCCAC
GTCGACAAGAAGATCCACAAGCTGGCGGGCCGCCTTGGGCCCAAGGAGCTGGAGCAGATC
AAGAGCCAGAAGATGAAGATCGTGGGCGCGCTGTGGTGGACCTACGTCATCAGCCTGTTC
TTCCGCATCATGCTGGAGGTCATCTTCATGTTCCTCTTCTACATGATCTACCCCGGCTAC
AAGATGATCCGCCTGGTCAAGTGCGACTCGTACCCCTGCCCCAACACGGTGGACTGCTTC
GTGTCGCGGCCCACCGAGAAGACGGTGTTCACCGTGTTCATGCTGGCCGTGTCGGGCGTC
TGCATCCTGCTGAACATCGCCGAGGTCCTCTTCCTGGTGGCGAAGGCCTGCGGGAGGCAG
CTTAGCAACACCAAGGACGGGGGCGGCCTCTGGGGCTGGCTGGCCCACAAGATCTCCTAC
TAG

>Gm-GJB1-G20195 Ensembl prediction. No modifications.
ATGAACTGGGGGTCCTTTTATGCCGTGATCAGCGGCGTAAACAGGCATTCGACGGGCATC
GGGCGAATATGGCTCTCGGTCATATTCATCTTCCGCATCCTGGTGCTGGTGGTGGCGGCC
GAGAGTGTGTGGGGCGACGAGAAGTCGGGCTTCACATGCAACACGCAGCAGCCCGGCTGC
AACAGCGTGTGCTACGACCAGTTCTTCCCCATCTCCCACATCCGCCTGTGGGCCCTGCAG
CTCATCCTGGTGTCCACGCCCGCTCTGCTGGTGGCCATGCACGTGGCCCACCGGCGGCAC
ATCGACAAGAAGATCCTGAAGCGGTCGGGCCGCGGCAGCCCCAAGGAGCTGGAGCACGTC
AAGAGCCAGAAGTTCCAGATCGTGGGCGGGCTGTGGTGGACGTACATGGTCAGCATCGTG
TTCCGCATCGCCCTGGAGGTGGTCTTCCTCTACATCTTCTGGCGGATCTACCCCGACTTC
AAGATGGTGCGGCTGGTCAAGTGCGACTCCTTCCCGTGCCCCAACACGGTGGACTGCTTC
GTGTCGCGGCCCACCGAGAAGACCATCTTCACCGTGTTCATGCTGACCGTGTCGGGCGTC
TGCGTGCTGCTCAACCTGGCCGAGGTGGTCTACCTGGTGGGGAGGGCGTGCCAGCGATGC
GCCCGGGACCCGGAGGAAGACAACAAGGTGGCGTGGATTGGCCAGAAGATGTCCACGTAC
AGGCAGAACGAGATCAATCAGCTCATAGCCGGCCAATCGATCAAGCCCAAGTTCCCCGTG
ACTAGAAAGGGTTCGGCCGATAAAGGCGACCGGTGCTCCGCTTTCTGA

>Gm-NN-cx30.3-G09100-1 Ensembl-predicted intron included in sequence (underlined). Further modified according to XM_030354646 (lower case letters). Suggested Splice sites. This Ensembl prediction contains two separate and unique connexins sequences, the present one and a gja3 sequence.
ATGCCGTCGTGGGGGGCCCTGCTGGCCCAGCTGAGCGGGGTCAACCGCTACTCCACCAGC
TTGGGGAAGGTGTGGCTGTCGGTGCTCTTCATCTTCAGGGTGATGGTGCTGATCGTGGCG
GCCGAGAGCGTGTGGGGAGACGAGCAGACAGACTTCACCTGTAACACCCTGCAGCCGGGC

TGTGAGAACGTCTGCTACGACCACTTCTTCCCCGTCTCCCACATCAGACTCTGGTGTCTC
CAGCTGGTCTTCGTCTCCACGCCTACCCTCCTCGTCGCCATGCACGTGGCCTACCGTAAC
CACGGCGACAAAAAGagactcctacaggccgaggaggcagagctagagaacctgaagagg
cggagactccagctgactggcgccctctggtggacgtacgcctgcagccttgtggtccgg
ctgctgtttgaagcgggcttcatgtacgtcctgtacgcgctctaccgcggcttccagatg
ccgcggctggtgcagtgcgtggagtggccctgccccaacgtggtggactgcttcgtgtcg
cggcccaccgagaagacggtgttcaccgtgttcatggcgtccgcctccagcgtctgcatg
ctcctcaacgtggccgagctggcctacctggtcgtcaaggccgtcactaggaagtcttga

>Gm-cx30.3-G15795 Our modification. Ensembl-predicted introns are included (underlined).

ATGACTTGGGGCGCGCTGTACGCCCAGTTGGGCGGAGTCAACAAGCACTCCACCAGCCTG
GGGAAGATCTGGCTGTCGGTGCTCTTCATCTTCCGCATCACCATCCTCGTCCTGGCGGCC
GAGAGCGTGTGGGGCGACGAGCAGTCGGACTTCACGTGCAACACGCAGCAGCCGGGCTGC
AAGAACGTCTGCTACGACCACTTCTTCCCCGTGTCCCACATCCGCCTGTGGTGCCTGCAG
CTGATCTTCGTGTCCACGCCGGCGCTGCTTGTGGCCATGCACGTGGCCTACCGTAACCGG
GGCGACAAGCGCACCATGCTGCGGTCCAACGGCGGGGAGAAGACCACGGACCTGGAGCTC
GAGGGGCTGAAGCGCCGGAGGCTGCCCATCACGGGGTCCCTGTGGTGGACCTACACCTGC
AGCCTGTTCTTCCGGCTGATCTTCGAGGGCGGGTTCATGTACGCCCTGTACTTCCTGTAC
GGCGGCTTCCAGATGCCGCGGCTGGTCAAGTGCGAGCAGTGGCCGTGCCCCAACAAGGTG
GACTGCTTCATCTCGCGGCCCACGGAGAAGACGGTGTTCACCATCTTCATGGTGTCGTCG
TCCACCATCTGCATGGTGCTGAACGTGGCGGAGCTGGCCTACCTGGTGGCCAAGGCGCTG
TTGCGCTGCTCCAACCGTGCGGCCCGCAGGAAGATGCCCTACGTCCACCACGACGGCGGG
CGGCGAGAGACCTGGCCCTGA

>Gm-cx28.6-G18713 Our suggested modification. Splice sites. Underlined: Predicted by Ensembl as intron, here included as part of exon.

ATGAACTGGTCCGGCCTGGAGAGCTTGATCAGCGGGGTCAACAAGTACTCCACCGTGTTC
GGCCGCTTGTGGCTGTCCATGGTCTTTGTGTTTCGGGTCATGGTCTTTGTGGTTGCAGCT
CAAAGAGTTTGGGGTGACGAAAACAAAGATTTTGTCTGTAATACGAAACAGCCGGGCTGT
ACCAACGTGTGCTATGACAGCATCTTCCCCATCTCCCACATCCGTCTGTGGGCCCTGCAA
CTGATCTTCGTCACCTGCCCGTCCCTCATGGTGGTGGCCCACGTGAAGCTGCGTGAAGAA
AAGGACCTTAAGTACACCGTACTGCACGAGGGCTCCCACCTGTACAGCAACCCGGGCAAG
AAGAGGGGGGGGCTGTGGTGGACCTACCTGCTGAGTCTGGTCTTCAAGGCAGGCTTCGAC
GCCTCGTTTCTCTACGTTTTGTATCGGATATACCACGGATATGACATGCCCAGGTTGTCC
AAGTGCTCCCTGGATCCCTGCCCTAACACCGTGGACTGCTTCATCAGCCGTCCCACAGAG
AAGAAGATCTTCACCCTGTTCATGGTGGTCACCAGCGCCATCTGCATCTTGATGTGCTTG
TTTGAGATGTTGTACCTCATTGGCAAACGGATTCAGAAAGCCCTCAGGGTTCAGAACTCC
ATTAACAGGCTCCTATTCGCCGAGCGGCACGAGATCAAAAACCTGGTCCCGCCCAGATCA
CAAACCCGCCGCCACTATTCCCCTCAGAGCAAAAGTTTAAGCAAGATGGACAAAGCCAAG
GAGACCACGACAACCCTGTAG

>Gm-cx30.9-G07064 Our modification. Splice site. Ensembl-predicted introns included (underlined)

ATGAACTGGTCCTACCTGGAGGGGCTCATCAGCGGGGTCAACAAGTACTCCACGGGCTTC
GGCCGCATCTGGCTCTCCATGGTCCTCATCTTCCGCGTGATGGTGTTCGTGGTGGCCGCG
CAGCGCGTGTGGGGCGACGAGAGCAAGGACTTTGTGTGCAACACCGTCCAGCCGGGCTGC
AACAACGTGTGCTACGACAGCATCTTCCCCATCTCCCACATCCGCCTGTGGGCCATGCAG
CTCATCTTCGTCACCTGCCCGTCGCTGATGGTGGTGGGCCACGTGAAGTACCGCAAGAAG
AAAGACCTGCAGTACACCACCTCTCATGAGGGCCATCACCTCTACGCCAACCCGGGGAAG
AAGCGCGGGGGGCTTTGGTGGACGTATCTGCTCAGTCTGATCTTCAAGGCAGGATTCGAC
GCCGCCTTCCTCTACATCCTCTACTACATCTACGAGGGCTACGACATGCCCCGCCTCTCG
AAGTGCAACCTGGCGCCCTGCCCCAACGTGGTCGACTGCTACATCTCCCGGCCCACCGAG
AAGAAGATCTTCACCCTGTTCATGGTGATCTCCTCCAGCTTGTGCGTGCTCATGTGCATC
TGTGAGATGGTGTACCTCATCTTCAAGCGCATCCAAAAGCTACTGGTGAAGAAGAGGGAG
GCGGACAGTAGGTTGTTCGCCGAACGCCACGAGATGAAGCCGCTGGCCCGGCCGCGGTCG
GACTTCAGGTCGAAGATGTCCATCCGGGTGGACCCCACGAACACGGCGTCCATACAGAAC
CTGAGCAACACAAAACGAGAGGAGGCGCCCATACAGAACCTGAGCGAGGATTTGTTGAAA
AAGAAGAGAGTAAACTGCAAAATAGATGGATAG

>Gm-cx34.5-G18894 Exon 1 suggested by Ensembl is here shortened by 12 nt. Underlined: Sequence not included in Ensembl transcript prediction, but we consider likely as a part of cds.

ATGGGTGAATGGGACCTGTTAAGTCGCCTGCTGGACCAGGTCCAGACCCACTCCACCGTC
ATCGGCAAGGTGTGGCTCACCGTGCTGTTCGTGTTCCGCATCCTGGTGCTGAGCACCGCC
ACAGAGAAGGTGTGGGGTGATGAGCAGTCCGACTTTGTGTGCAACACCAACCAGCCGGGG
TGTAAGAACGTGTGCTACGACCACGCCTTCCCCATATCACACGTCCGCTTCTGGGT**G**CTG
CAGATCATCTCGGTGGCCACGCCAACCCTGGTGTACCTGGGCCACGTCCTCCACGTCATC

CACGCTGAGCGCAAGGTGAGACTGAAGATCCAGAGGCAGGCGGAGCTGGACGAGGACGCC
CACCTGTTCCTGAAGAAGGGCTACAAGGTCCCCAAGTACAGCCACAGCAACGGCAAGATC
AACCTGCGGGGGAGCATCCTGCGCAGCTACCTGCTCAACCTGGTGGCCAGGATCCTGTTG
GAGCTGGGCTTCATCCTGGGCCAGTACTTCCTGTACGGCTTCACACTGCAGGCCCGCTAC
GTCTGCAGCATGTCGCCCTGCCCGCACAAGGTGGACTGCTTCCTCTCCAGACCCACGGAG
AAGTCCATGTTCATATGGTTCATGCTGGTGGTGGCCTGCGTGTCTCTCCTCCTCAGCATC
GTGGAGCTGCTTCATCTGTGTGTGAAAGTGGCGGGCGAGTGCATAGCTCGGAGGCAAGAC
TACACCGTCACCCCCGTCACCCCGCCGCTCTTGGAGAGGAAGGCCTTCAAGAACCGAGAG
CAGAGGATCCAGGACCATTACAACCTGGAG

>Gm-NN-cx35.4-G04675 Our modification. Sequence extended in 3'-direction (underlined) until a stop codon.
ATGGATTGGAAGAGCCTTGAGGGTCTGCTCAGTGGGGCCAACAAGTACTCCACCATCTTC
GGGCGCATCTGGCTGTCGATCGTCTTCGTCTTCCGGGTGATGGTGTTCGTGGTGGCGGCA
GAGCGCGTGTGGAGCGACGACCAGGCCAACTTTGACTGCGACACCCGGCAGCCCGGCTGC
AAAAATGTCTGCTACAACCACTTCTTCCCGGTCTCCCACATCCGCCTTTGGTCCCTTCAG
CTCATCTTCGTCACGTGCCCGTCCTTCTTGGTGGTTCTGCACGTGGCGTACCGCGAGGAG
CGCGAGCGTAAGTACCGCATCAAGCACGGCGAGCAGGCGCGTCTCTATGACAACACGGGA
CAAAAGCACGGGGGGCTGTGGTGGACCTACCTGTTGAGCCTCTTCTTCAAGACGGCCATC
GAGCTGGGGTTCCTCTACCTCCTCCACCTCATGTACGACAGCTTCAAGCTCCCCAGGCGC
GTCAAGTGCGGCGTCAGCCCCTGTCCCAACGTGGTGGACTGCTACGTGGCCAGACCCACC
GAGAAGACAGTTTTCACCTACTTCATGGTGGCTGCGTCCATGGTGTGCGTGGTCCTGAAC
CTGTGCGAGATATTCTATTTGATCACCGTGCGCCTGTTGACGATGAAGCGCCAGGGTAGG
CCTACAGTCCGCACGGCTTCGAAGAGGATCATCTCTGAAAATAAGGATTTGATGTAA

>Gm-cx34.4-G19007 Our modification. Underlined sequences are added from the Ensembl-predicted introns, giving the indicated stop codon.
ATGAACTGGGCATTCCTTCAGGGCCTCCTCAGCGGGGTCAACAAATACTCCACGGCGTTT
GGCCGCGTGTGGCTCTCCATCGTCTTCCTCTTCCGCGTCATGGTGTTCGTGGTGGCCGCC
GAGAAGGTGTGGGGCGACGAGCAGAAGGACTTCAAGTGCAACACGGCGCAGCCGGGCTGC
CACAACGTGTGCTACGACCACTTCTTCCCCGTGTCCCACGTGCGCCTGTGGGCCCTGCAG
CTCATCTTCGTCACCTGCCCCTCGCTGCTGGTGGTCATGCACGTGACCTACCGCGAGGAG
CGGGAGAAGAAGAACAAGGCGAAGCACGGCGAGAACTGCCGCCGCCTGTACGCCAACCCG
GGCAAGAAGCGCGGCGGCCTGTGGTGGACCTACGTCCTGACGCTGGTGTTCAAGATCGGC
GTGGACACGGTGTTCGTGTACCTCATCTACTACATGTACGAGGGCTACGACTTCCCCTCG
CTCGTCAAGTGCGTGGAGGCGCCGTGCCCCAACACGGTGGACTGCTACATCGCGCGGCCC
ACCGAGAAGCGCATCTTCACCCTGTTCATGGTGGTCACCAGCATGGTGTGCATCCTGCTC
TCCATCTTTGAGATCGTGTACCTGGTGGGCAAGAAGTGCCGCGAGGGCGTGGTGAAGCTG
CACTACCAGCACCGCTCGCACCAGAACCAGCAGGCCAGGGACTTGGGCCCCTCGCTGGCG
GGGGGCAAGAGCGGGAACCTGGTGGAGGCCAACACTCTGAGGCTGGTGGAGAAGGTTCTC
CCCGGCACGCCTGCGCCGTCGTACAGCGTGGCCGTCGCTTCGGACGAGGTCACCCCCAGA
TGA

>Gm-NN-cx34.4-G04662 Our modification. Ensembl-predicted introns included (underlined). Splice site. Essentially identical to ENSGMOG00000004650, which ends at the splicing site.
ATGAATTGGGCCTTTCTCCAAGGCCTCCTGAACGGAGTCAACAAGTACTCCACCGTCTTC
GGCCGCATATGGCTCTCCGTGGTGTTCATCTTCAGGCTCATGGTTTTCGTTGTGGCTGCT
GAGAAGGTGTGGGGCGACGACCAGAAGGATTTCGACTGCAACACGAGGCAGCCCGGCTGC
CACAACGTCTGCTACGACAACTTCTTCCCCATCTCCCACACCCGCCTCTGGGCTCTGCAG
CTCATCTTCGTCACATGCCCATCATTGCTGGTCGTGCTGCATGTGGCCTACCGGGAGGAG
CGCGAGCGCAAACACCGGCTGAAGTACGGCGAGGACTGCAAGCCGCTCTACGACAACACG
GGAAAGAAGCGCGGAGGTCTGTGGTGGACCTACTTCCTCAGCTTGCTGTTCAAGATGCTG
GTGGAGGCCGTGTTTGTCTTCCTGCTCTTCTACATTTACGAAGCCCCCTTCTTCCCCGCCG
CTTGTCAAATGTGATGAATCCCCGTGTCCCAACGTGGTGGACTGCTACATCGCCAGACCT
ACAGAGAAGAAGGTCTTCACCGTGTTCATGGTGGTGACCAGCTTTGTGTGCATTCTGCTC
ACTATTTGCGAGGTATTTTATCTCTGTGGTAAGAGGTTCTGGGAGTGCTGTCGTGAACAA
CAGCACCCCGGTCGACACAACGGCAACTCCTTCGTCATGGCCAAAATCCCCCAGACCAGA
AGTGTTAACTCCGTCTACAAAGAGCCTCTCACATCAGAGAAGATGACGATGGTGGATGGT
AAAGGCCCAACGACTCCGGAGAGTTCTGCACCGGCGTACAGTCTGGCCATCTCTTGA

>Gm-cx35.4-G20298 Underlined: Extended in 3'-direction to reach stop codon.
ATGGACTGGAAGACCTTCCAATCCCTGCTGAGCGGTGTGAACAAGTACTCCACGGCGTTC
GGGCGGATATGGCTGTCCATCGTGTTCGTGTTCCGCGTCATGGTGTACGTCGTGGCGGCC
GAGCGGGTGTGGGGCGACGAGCAGAAGGACTTTGACTGCAACACCAAGCAGCCCGGCTGC
GCCAACGTGTGCTACGACTACTACTTCCCCATCTCCCACATCCGCCTGTGGGCCCTGCAG
CTCATCTTCGTCACCTGCCCCTCCTTCATGGTGGTCATGCACGTGGCGTACCGCGACGAG

CGCGAGCGCAAGTACCGCATCAAGTTCGGCGAGGAGAAAAAGCTGTACAACAACACGGGC
AAGAAGCACGGCGGCTTGTGGTGGACCTACCTGATCAGCCTCTTCGTCAAGACGGCCATC
GAGGTGGCCTTCCTCTACATCCTGCACTACATCTACGACAGCTTCTACCTGCCGCGCCTG
GTCAAGTGCGAGGTGTCCCCCTGCCCGAACAAGGTGGACTGCTACATCGGCCATCCCACG
GAGAAGAAGGTGTTCACCTACTTCATGGTGGGCGCCTCGGCCCTGTGCATCGTGCTCAAC
ATCTGCGAGATCATTTACCTCATCTCCAAGCGTGTGGCGCGCTGCGCCAACAAGCTCAAG
AAGCGGACCCGAGGCATGCCGCAGGAGACGCACGCCGGCTACGACGACAACCACGCCCCC
AACAGCTACCCCATGGAGATGATGTCCAAGCGAGACGTGACCAGGGACTTGCCTCCGTCC
TTCAGGACCAGCTGCAAGCCTCCGTACCAGCCCGCCATGCATTTGCTGAGGGCGGAGAAG
AGGGCGGAGATGA

>Gm-cx28.8-G20475 Our modification. Extended in 3’-direction until reasonable stop codon (underlined)
ATGAACTGGGGCTTCCTGGAGAACGTGCTGAGCGGCGTGAACAAGTACTCCACCGTCATC
GGTCGCATCTGGCTCTCCATCCTCTTCATCTTCCGCATCCTGGTGTACGTGGCGGCCGCC
GAGCAGGTGTGGAAGGACGAGCAGAAGGACTTCACCTGCAACACCCGGCAGCCGGGCTGC
GAGAACGTCTGCTACGACCACCTGTTCCCCATCTCCCAGACGCGCCTTTGGGCCCTGCAG
CTCATTATGGTGTCTACCCCGTCCCTCCTGGTGGCCCTGCACGTCGCATACCGGGAGCAC
AAGGAGTCCAAGTGCGGCCACACGCTCTACGAGGACCGGGGCAGGATCGACGGGGGCTG
CTTGGCACCTACATCGCGAGCATCATCATCAAGACCTTCTTCGAGGTGGCCTCGCTGCTC
GCCTTCTACTTCCTGTACAGCGGGTTTGAGGTGCCCCTGTTGTACCGCTGCGAGGAGAGC
CCCTGCCCCAACATAGTGGATTGTTACATCGCCAGGGCCACGGAGAAAAAGATCTTCCTG
TACATCATGGGATGTACGTCCGTCCTTTGCATTGTGCTGAATGTGGTCGAGCTTTTCTAT
ATTCTATGGAAGCAGTGCTCCAAGTACTTTAACAAGCGTTATGTCTCCGTGGAAGAGAGG
CAACGCAGACGCCACAGGTTTATGGTTTCTAATTACAATCTGCCTGTTTCTAACGTCAGC
AAGCCCGCATCCCCGCAGGCTCCACCCGGGCCGAACAAGGGCCTCAGGCGATCAGAGCCG
TCCTGTACACAAAGCCTCCCACATCTGATGAGACATTGA

>Gm-cx36.7-G16800 Our modification. Ensembl-predicted introns included (underlined). Splice site.
ATGACGGAGTGGACGCTGCTGAAGCGCCTGCTGGACGCCGTCCACCAGCACTCCACCATG
ATCGGCCGGCTCTGGCTCACCGTCATGGTGATCTTCCGGCTGCTGGTGGTGGCGGTGGCC
ACGGAGGACGTGTACGCAGACGAGCAGGAGATGTTCGTGTGCAACACCCTGCAGCCGGGC
TGCGCCACCGTGTGCTACGACGCCTTCGCCCCCATCTCGCAGCCCCGCTTCTGGGTCTTC
CACATCATCAGCGTGTCCACGCCCTCGCTCTGCTTCATCATCTACACCTGGCACAACCTC
TCCAAGCTGCCCCACCGCACGGGACACAGGCCCAGGGCCAGGCCCAGGCCGGGGCCCGAC
CACCCCCCGAGGGTCCCCGAAGGGGTCCTCTCCAAGTGTTACGTCTTCCACGTGTGCGTG
CGGGCCGTCCTGGAGGTGGGCTTCGTGACGGCCCAGTGGATGCTGTTCGGCTTCCGGGTG
CCCGTGCACTTCCTGTGCCCGTCGGCGCCCTGCACCCAGCCGGTGGACTGCTACGTGTCC
CGGCCCACGGAGAAGACCATCTTCCTGCTCTTCATGTTCTGCGTGGGGGTGTTCTGCATC
CTGCTCAACCTGCTGGAGCTCAACCACCTGGGCTGGAAGAAGATCCGCACGGTGGCCAAG
CTCCGGGAGACGGGGTCCTGGGAGGCTCGCCCCGCGAGGAGGGCGGGCTACGTGGCCTTA
CCCCCGGGATGCCCCTCGCTCGCGTCCACGCTGGGCTTCAGGGACGTGACCAGCACCACG
TCCCTCCCCACCCTGGACCTGGTGGTGGGGCACCAGCCCGACTGGACGTGCGCAGGGAAC
TGCACCCTGTTCGGGGCCACCGGGCCGGGCACGAAGGCCGAGCCGCGGCCGCCCGGGAGG
CCAGAGGGTGTGAGGAAGGAGGGACAGCCGCTGAGGATTAAGAGAGAGAACCGGGGACCC
AAGCAGCACAGTGCTGAAGTGTGGATATGA

>Gm-GJC1-G14340 Our modification. Ensembl-predicted introns included (underlined).
ATGAGCTGGAGCTTCCTCACGCGGCTGCTGGAGGAGATCCACAACCACTCCACCTTCGTG
GGGAAGCTGTGGCTCACCGTGCTCATCGTCTTCCGCATTGTGCTCACGGCCGTCGGAGGC
GAGTCCATCTACTACGACGAGCAGAGCAAGTTCATCTGCAACTCGGGCCAGCCGGGCTGC
GAGAACGTCTGCTACGACGCCTTTGCCCCGCTCTCTCATGTGCGCTTCTGGGTCTTCCAG
ATCATCCTGGTGGCCATGCCCTCCCTCATGTACATGGGCTACGCCGTCAACAAGATCGCC
CGCGCGGACGAGGCCAAGGGAGGCACCGGCGCCGCGGCTGTCAGGACCGCCGTCGGGGCG
TCCGGGGGCTACACCCACCGGAAGCCCCGCAAGATCTGTTTCGGGGCACGGCAGCACCGG
GGCATCGAAGAGGCAGAGGAGGACCACGAGGACGACCCCATGATCTACGAGGTTCCCGAA
ATTGAGCCGCCACATCGGCCACGGGACCCTTTGCAGCCCACTCCCAGGCCCAAGATCCGC
CACGACGGCCGCACACGCATCCGGGACGAGGGGCTGATGCGTATCTATGTGTTGCAGCTG
GTGACCCGTACCGTCCTGGAGGCGGGCTTCCTGGCGGGGCAGTACCTGCTGTACGGGTTT
CGCGTGGCGCCCGTGTTTGTGTGCTCGGGCGAGCCCTGCCCCCATAGCGTGGACTGCTTT
GTGTCGCGTCCCACCGAGAAAACCATCTTCCTGCGCATCATGTACGGCGTCACCGTGCTC
TGCCTCACGCTCAACATCTGGGAAATGCTGCACCTCGGCGTCGGATCCATCTGCGACATC
CTGCGACGCCGGCGCTGCCCGCCCCCGGAGGACGAGTACCAGCTGGGTCTGCTCGGCACC
ATTGGAGCCAGCGAGGGGTCTGTGGGCGTCTCGGGTCCCGAGGCCGGCGAGGGCGAAGGA
CCGGTCGGGGAGGGCGGTGCGGACTACATCGGATACCCATTCTCCTGGAACCCTACCCCG
TCGGCACCCCCGGGATACAACATCGTAGTGAAGCCGGAGCAGATCCCGTACACGGATCTC
AGCAACGCTAAGATGGCGTGCAAGCAGAACCGGGAGAACATCGCCCAGGAGGAGCAGCAG

CAGTTCGGCAGCAACGAGGACAACTTCCCCACCGGAGGGGAGGCTCGCGTGGCGCTGAAC
AAGGACATGATCCAGCAGGCTCACGATCAGCTGGAGGCCGCCATCCAGGCCTACAGTCAG
CAGCACTGTGTGGAGGATCTGGGAGAACACAGGGACGATAAGCCTCGCAGTAACATTATT
CAGACCCAGCCCCCGCCCCCGATGCAGCCGCAGAAGGAGCGCAAACAACGGTCCAAACAC
GGCAAGGGAGGCAGCACCGGCGGATGTAGCAGCAGCAACAGTAGCAGCAGTAAGTCAGCA
GAGGGGAAGCCCTCTGTGTGGATTTAA

>Gm-NN-gjc1-G06421 Our modifications. Underlined: Extended into intron predicted by Ensembl. The extended sequence runs into a row of Ns (in Ensembl) Lower case: Sequence extended by Blast into GenBank wgs.

ATGAGCTGGAGCTTCCTGACCCGCCTGCTGGAGGAGATCCACAACCACTCCACCTTCGTG
GGGAAGATCTGGCTGACGGTGCTCATCGTGTTCCGCATCGTGCTGACGGCGGTGGGCGGC
GAGTCCATCTACTACGACGAGCAGAGCAAGTTCATCTGCAACTCGGGCCAGCCGGGCTGC
GAGAACGTCTGCTACGACGCCTTCGCCCCGCTCTCCCACGTCCGCTTCTGGGTCTTCCAG
ATCATCCTGGTGGCCACGCCCTCCCTCATGTACCTGGGCTACGCCGTCAACAAGATCGCC
CGCGCCGACGACCGGGCGGGcgtcggcggcggcgtcggcggggacggtttgccccagcgc
cgcgcgcgccaggaagtcgtacccgggcgcccggaggcagcaccgcggcgtggaggaggc
ggaggacgaccacgaggaggaccccatgatctacgaggtggccgagccggagagcgacgg
cggaggggccgggggagcggcgggcgggggccgacgggggcgcggcgcggggccgacggg
ggaggggtcaaggccaaggcgcgccaCGACGGGCGCCAGCGCATCAAGGAGGACGGGCTG
ATGCGTATCTACGTGCTGCAGCTGCTGGCGCGCTCCCTGCTGGAGGTGGGCTTCCTGCTC
GGGCAGTACGCGCTGTACGGCATGGCGGTGCCCTCCACGTACGCCTGCTCGGGCCCGCCC
TGCCCGCACACGGTGGACTGCTTTGTGTCGCGGCCCACCGAGAAGACCATCTTCCTGCTC
ATCATGTACGCCGTGTCCCTGCTCTGCCTGGCCCTCAACTTCTGGGAGATGCTGCACCTG
GGCGTGGGCACCATATGTGACATCCTGGGCTCCCAGCGCTCCCCAGCGCCCTCCAACGAC
GAGGCCTGA

>Gm-NN-cx43.4-G08258 Our modification. Ensembl-predicted introns are included (underlined).

ATGAGCTGGAGTTTCCTGACGCGTCTGCTCGACGAGATCTCCAACCACTCGACGTTCGTC
GGCAAGATCTGGCTCACGCTGCTGATCGTGTTCCGCATCGTGCTGACGGCGGTGGGCGGC
GAGTCCATCTACTACGACGAGCAGAGCAAGTTTGTGTGCAACACGCAGCAGCCCGGCTGC
GAGAACGTCTGCTATGACGCCTTCGCGCCCCTCTCCCACATTCGCTTCTGGGTGTTCCAG
GTGATCATGATCACCACGCCCACCATCCTGTACCTGGGTTTCGCCATGCACAAGATCGCC
CGCATGGACGACTCGGAGTACCAGCCGCGGCCGCGCAAGCGCATGCCGGTGGTGAGCCGG
GGCGCCAACCGCGACTACGAGGAGGCGGAGGACAACGGCGAGGAGGACCCCATGATCCTG
GAGGAGATCGAGCTGGAGAAGGACGCCGGCGGCGACAAGGCACCGGAGAAGCCGTGCCGC
AAGCACGACGGGCGCCGGCGCATCAAGCGCGACGGGCTCATGAAGGTGTACGTCTTCCAG
CTGATGGCGCGCGCCACCTTCGAGGCGGCCTTCCTGTTCGGCCAGTACGTCCTGTACGGC
CTGGAGGTGGCGCCGTCGTACGTGTGCACGCGCTCGCCCTGCCCGCACACGGTGGACTGC
TTCGTGTCGCGGCCCACCGAGAAGACCATCTTCCTGCTGATCATGTACGGCGTCAGCGCC
CTGTGCCTGCTCTTCACAGCGCTGGAGATCCTGCACCTGGGCTTTAGCGGCATGCGCGAC
TGCCTGTGCGGCGCCCGCTCGCCGCCG

>Gm-cx44.2-G14499 Modified according to XM_030353496. Ensembl-predicted introns are included (underlined). Suggested splice site.

ATGAGCTGGAGCTTCCTCACGCGGCTGCTGGACGAGATCTCCCAGCACTCTACCTTTGTG
GGCAAGGTGTGGCTGTCGGTGCTCATCATCTTCCGCATCGTGCTGACGGCGGTGGGTGGA
GAGACCATCTACCACGATGAGCAGAGCAACTTTGTGTGCAACACGCAGCAGCCCGGCTGT
GAGAACGTCTGCTACGACGCCTTCGCGCCGCTCTCGCACGTCCGCTTCTGGGTCTTCCAG
GTGCTGATGATCACCACGCCCACCATCATGTACCTGGGCTTCGCCACGCACAAGGTGGCC
CGCATGGGTGACCCCCAGTACCAGCCCACCCGCCGCGCCCGCAAGCGCATGCCTATTGTG
ACCTCCGGGGCCGCGCGCAACTATGAGGAGGCAATGGAGGACGGGGAGGAGGACCCCATG
ATGGAGGAGGAGATCGAGCCCGAGAAGGCGAAGGCGGACAATGGCCCGGAGAAGAAGCAC
GACGGCCGGCGTCAGATCCAGGCGGACGGCCTGATGAAGGTCTATGCCTGCCAGCTGCTG
ACCCGCGCCGCCTTCGAGATGGCCTTCCTCTACGGCCAGTTCCTCCTGTACGGCTTCCGC
GTTGCGCCGGACTACGTGTGCACGCGTCTGCCCTGCCCCCACACGGTTGACTGCTATGTG
TCACGGCCCACCGAGAAGACCATCTTCCTGCTGATTATGTACGTGGTGTCCTTTCTCTGC
CTGCTCCTCACGCTCCTGGAGATGGTGCACCTCGGCGTTGGCGGCCTCCGCGACACCTTC
CGCCGCAGGGCCACCCTGGTCTCCCGAACCAGGCCAGCCGGAGGAGGAGGAGGAGGAGGA
GCCTCGGCGCCCCCCAGGCTACCACGCCACGGTGAAGCATGA

>Gm-cx43.4-G17444 Our modification. Ensembl-predicted introns are included (underlined).

ATGAGCTGGGACTTCCTGACGAGTCTGCTCGACGAAATCTCCAACCACTCGACGTTCGTG
GGCAAGACCTGGCTCACGCTGCTCATCGTGTTCCGCATCGTGCTGACGGCGTTGGGCGGC
GAGTCCATCTACGAAGACGAGCAGAGCAGCTTCGTCTGCAACACGCTGCAGCCCGGCTGC
GAGAACGTCTGCTACGACGCCTTCGCACCCCTCTCGCACATTCGCTTCTGGGTGTTCCAG

GTGATCGTGATCACCACGCCCACCGTCCTCTACCTGGGCTTCGCCATGCACAAGATCGCC
CGCATGGACGACTCGGAGTACCGGCCGCGTCCGCGGCAACGCCTGCCGTCAGTGAGCCGT
GGCGCCGGCCGCGACCACCAGGAGGCGGAGGCCAACTGCGAGGAGGACCACGTGATCCTG
GAGGAGAACGAGCCGGAGAAGGACACCGGCGACAAGGCGCCGGAAAAGCTGCGCCGCAAG
CACGACGGGCGCCGGCGCATCGAGCGCGACGGGCTCATGAAGGCGTACGTCTTCCAGCTG
ACGGCGCGCGCCACCTTCGAGGGGGCCTTCCTGTACGGCCAATACCTCCTGTACGGCCTG
GAGGTGGCGCCGTCGTACGTGTGCACGCGCCCGCCCTGCCCGCGCACGGTGGTCTGCTTC
GTGTCGCGGCCCACCGAGAAGACCATCTTCCTGCGGGTCATGTACGGCGTCAGCGCCCTG
TGCCTGCTCTTCACGGCGCTGGAGATCCTGCACCTGGGCGTCAGCGGCGTTCGGGACTGC
CTTTGCGGCCGCCGGCCCTCGGCCCGCCCCCCGGCTACCACTTGA

>Gm-cx47.1-G19771 Our suggested modification. Underlined: Predicted by Ensembl as intron, here included as part of exon.

ATGAGCTGGAGCTTCCTCACACGGCTGCTGGAGGAGATCCACAACCACTCCACCTTTGTG
GGCAAGGTGTGGCTGACGGTGCTCATCATCTTCCGCATCGTGCTGACGGCGGTGGGCGGC
GAGTCCATCTACTCGGACGAGCAGACCAAGTTCACCTGCAACACCAAGCAGCCCGGCTGT
GACAACGTGTGCTACGACGCCTTCGCGCCACTCTCCCACGTGCGCTTCTGGGTCTTCCAG
ATCATCATGATCTCCACGCCGTCCGTCATGTACCTGGGCTACGCCATCCACAAGATCGCC
CGCAGCTCCGAGGACGAGCGCAAGAGGAGCCGGCACCACGGCCGCCTCCGCAGGAAACCC
CCGCCGCACACCCGGTGGCGGGAGAGCCGGCGGCTGGACGAGGCGCTGGAGGAGGAGCTG
GACGTCGACGACGGCGAGCCAATGCTGTACGACGACGTCCTGGACGCCAGGCCAGAGCCG
GCGGTGGCCGGCGGCGGAGGTCCGCAGAAGCACGACGGGCGCCGGAGGATCGTGCAGGAG
GGCCTCATGAGGATCTACGTCCTGCAGCTCATGTCCCGGGCCATCTTCGAGATCAGCTTC
CTGGCGGGGCAGTACCTGCTGTACGGGTTCCGCGTCAGCCCGTCGTACGAGTGCGACCGC
CTGCCCTGCCCGCACCGCGTGGACTGCTTCATCTCCAGGCCCACGGAGAAGACCATCTTC
CTGCTCATCATGTACGTGGTGAGCTGCCTGTGTCTGCTGCTCAACGTGTGCGAGATGTTC
CACCTGGGCATCGGAACGTTCCGGGACACCCTCCGCCAGAAGAGGGACCGCGGCCGGCGG
ACGTCCTACGGCTACCCCTTCTCCCGGAACATCCCGTCGTCCCCGCCCGGGTACAACCTG
GTGGTGAAGTCGGACAAACCGCTCCACCGGATCCCCAACAGCCTGATCACACACGAGCAG
AACATGGCCAACGTGGCCCAGGAGCAGCAGTGCACCAGCCCGGATGAGAACATCCCCTCC
GATCTGGCCAGCCTCCACCGCCACCTCCGGGTGGCCCAGGAGCAGCTGGACATGGCCTTC
CAGACGTACAGCTCCAAGAACGACAACCAACCCCCCTCCAGGACGAGCAGCCCCATGTCA
GGGGGCACCATGGCCGAGCAGAACCGGGTGAACACGGTTCAGGAGAAGCAGGGAGCCCGG
CCGAAGTCGGCCACGGAGAGACCGGGGACCCTTTTAAAAAACGGGAAGACTTCTGTGTGG
ATTTAA

>Gm-NN-gjd2-G14288 Underlined: Sequence predicted as introns by Ensembl are here included as exon. Italics: Ensembl has a long row of Ns in this area, which we have partially replaced by sequences found by Blast in GenBank cod wgs.

ATGGGAGAATGGACCATCCTGGAGCGCCTCCTGGAGGCCGCTGTGCAGCAGCACTCTACC
ATGATTGGGAGGATCCTGCTGACAGTGGTGGTGATCTTCCGCATCCTGATCGTTGCCATC
GTGGGCGAGACGGTGTACGAGGACGAGCAGACCATGTTCATCTGCAACACCCTCCAGCCG
GGATGCAACCAGGCCTGCTACGACAAGGCCTTCCCCATCTCCCACATCCGCTACTGGGTG
TTCCAGATCATCCTAGTGTGCACGCCCAGCCTCTGCTTCATCACCTACTCTGTGCACCAG
TCGGCCAAGCAGCGGGACCGCCGCTACTCCTTCCTGTACCCCATCCTGGAGCGGGACTAC
GGCGGCCTGGGGGGCGGCCTGGGG*GCGGCCTGGGGGCGGCCTGGGGCGGCGGAG*CGCGGC
GGAGGCGGGGGCGGCGTGGGACGCAAGCTGCGCAACATCAACGGCATCCTGGTGCAGCAC
GGCGACAGCGTGGGCGGCAAGGAGGAGGCGGACTGCCTGGAGGTGAAGGAGATCCCCAAT
GCGCCGCGCGGCCTCACGCACAGCAAGAACTCCAAGGTGCGGCGGCAGGAGGGCATCTCA
CGCTTCTACATCATCCAGGTGGTGTTCCGCAACGCTCTGGAGATCGGCTTCCTGGCGGGC
CAGTACTTCCTGTACGGCTTCAGCGTGCCGGGCATCTTCGAGTGTGACCGCTACCCGTGT
CTGAAGGAGGTGGAGTGCTACGTGTCACGGCCCACCGAGAAGACGGTGTTCCTGGTGTTC
ATGTTTGCCGTGAGCGGCCTGTGCGTGGTGCTCAACCTGGCCGAGCTCAACCACCTGGGC
TGGAGGAAGATCAAGGCGGCCATCCGGGGCGTGCAGGCCCGCAGGAAGTCCATCTGCGAG
ATCCGGAAGAAGGACATGGCGCACCTGTCCCAGCCACCCAACCTGGGCCGCACGCAGTCC
AGCGAGTCCGCCTACGTCTGA

>Gm-NN-gjd2-G03494 Underlined: Sequences predicted as introns by Ensembl are here included as exon. Lower case: Sequence extended in 5′ direction by Blast in GenBank cod wgs. Exon 1 found from XM_030360300.

atgggcgagtggaccatcctggagcgacttctggaggctgcggtccagcagcactccact
atgatcggccggatcctactgactgtagtggttatcttccggatcctgatcgtaggaaTC
GTCGGTGAGAAGGTGTACGAGGATGAGCAGATCATGTTCATCTGCAACACCATGCAGCCG
GGCTGCAACCAGGCCTGCTACGACAAGGCCTTCCCCATCTCCCACATCCGCTACTGGGTG
TTCCAGATCATTCTGGTGTGCACGCCCAGCCTGTGCTTCATCACCTACTCGGTGCACCAG
TCGGCCAAGCAGCGCGAGCGCAGCTACGCCTTCCTGCACCCGTACATGGACGGGGCCACC
GTGGCCCACCAGGCGCACGGCCAGACCGGCCACGGCGGGGGCCACGGCCGCCACGACCAC
CACGCGGCCCGCAAGCTGCGCAACATCAACGGCATCCTGGTGCAGAACGACAGCAGCAAG

GAGGACCACGACATGGAGACCAAGGAGATCCCCAACATGGCGCGCAGCCTGCCGCACGGC
AAGAGCGCCAAGGTGCGGCGGCAGGAGGGCATCTCGCGCTTCTACGTCATCCAGGTGGTG
TTCCGCAACGTCCTCGAGATCGGCTTCCTCGCGGGCCAGTACTTCCTGTATGGCTTCAAT
GTGCCGGGGATGTTTGAGTGCGACCGCTACCCCTGTGTGAAGGAGGTTGAGTGCTACGTA
TCGCGGCCGACAGAGAAGACCGTCTTTCTGGTCTTCATGTTCGCCGTTAGCGGCATCTGT
GTGCTGCTCAACCTAGCAGAACTCAACCACCTCGGCTGGCGGAAGATTAAAACGGCCGTC
AGAGGGGTGCAGGCGCGTAGGAAGTCCATCTGTGAGGTGCGTAAGAAGGACGTGTCCCAC
CTCTCCCAGGCCCCCAACCTTGGCAGGACCCAGTCTAGCGAGTCGGCCTACGTCTGA

>Gm-GJD2-G09811
ATGGGGGAATGGACTATACTAGAGAGGCTCCTGGAGGCTGCTGTCCAGCAGCACTCGACT
ATGATAGGAAGGATCCTACTCACTGTGGTGGTCATCTTCCGGATCTTAATCGTAGCGATA
GTCGGAGAGACTGTCTATGATGACGAGCAAACCATGTTTGTGTGTAACACCTTACAACCG
GGCTGCAACCAGGCATGTTACGACAAGGCATTTCCCATTTCACACATCCGATACTGGGTG
TTTCAAATTATCATGGTGTGCACGCCGAGCCTGTGCTTCATCACCTACTCGGTGCACCAG
TCGGCCAAGCAGAAGGAGCGGCGCTTCTCAACGGTGTACCTGACGCTGGACAAGGACCAA
GACTCCATGAAGAGGGAAGAGAGCAAAAAGATCACCAAGAGCACCATCGTGAACGGAGTA
CTGCAGAACACGGAGAACACCACCAAAGAGGCCGAGCCGGACTGCTTGGAGGTCAAGGAG
ATCCAGAACTCGGCCATGAGAACTAAGTCGAAATTAAGGCGCCAGGAGGGAATCTCGAGG
TTCTACATCATCCAAGTGGTGTTCAGAAACGCGTTGGAGATCGGTTTTCTGGTGGGGCAA
TATTTCTTGTACGGATTCAACGTGCCGTCCGTGTACGAGTGCGATCGATACCCGTGCATC
AAAGACGTCGAGTGCTACGTCTCCAGGCCCACGGAGAAGACCGTGTTCCTGGTGTTCATG
TTCGCCGTCAGCGGGTTTTGTGTGATTCTGAATCTGGCGGAACTCAATCATCTGGGCTGG
CGAAAGATCAAGACGGCCGTGCGGGGCGTGCAGGCGCGACGAAAGTCCATCTATGAGATC
CGAAACAAAGACTTGCCGAGAATGAGTGTGCCCAATTTCGGGCGTACTCAATCCAGTGAC
TCCGCTTATGTGTAA

>Gm-NN-gjd2-G01582 Modified according to XM_030345236 Splice site
ATGGGGGAATGGACCATCCTCGAGCGTCTACTGGAGGCGGCCGTTCAACAGCACTCTACT
ATGATAGGGAGGATCCTGCTCACGGTGGTGGTGATCTTCCGGATCCTGATCGTGGCCATC
GTCGGGGAGACCGTCTACGATGACGAACAGGAGATGTTTGTGTGCAACACCCTGCAGCCG
GGCTGCAACCAGGCGTGCTACGACCAGGCCTTCCCCATCTCCCACATCCGGTACTGGGTT
TTCCAGATCATCATGGTGTGCTGCCCCAGCCTCTGCTTCATCACCTACTCCGTCCACCAG
TCGTCCAAGCAGAAGGAGCGCGGCTTCTCCGGCGTGTACCTGTCGGTGGACCGGACGGGC
CGGCCAGACGACAACCTGCTGAAGAACACTCTGGTGAACGGCCTGCTGCAGAACTCGGAG
AACTCGTACAAGGAGGCGGACCCGGACGCTCACATCTTCCCCCGGCAGTGTGTGAGGACG
CAGTCCAAGATGAGGAGGCAGGAGGGCATCTCCCGCTTCTACATCATCCAGGTGGTCTTC
CGGAACATGCTGGAGGTGGGCTTCCTGGTGGCCAGTACTTCCTGTACGGGTTCAACGTG
CCCCCGGTGTACGAGTGCGACCGGTACCCGTGCATCAAGGACGTCGAGTGCTACGTCTCA
CGGCCCACGGAGAAGACCGTGTTCCTGGTCTTCATGTTCGCCATCAGCGGCGTCTGCGTG
GTCTTCAACCTGGCGGAGCTCAACCACCTGGGCTGGAAGAAGATCAAGGAGGCCGTGAGG
GGCGTGCAGGCCCGGAGGAAGTCCGTCTACGAGATCCGCAAGAAGGACCCGGCCAAGATG
AGCGGATTTGGACACATCCAGTCCAGTGACTCCGCCTACGTTTGA

>Gm-NP-cx39.2
ATGGGGGACTGGTCAATACTGGGACGTTTTCTGTCCGAAGTGCAAAACCATTCCACAGTG
ATAGGGAAGATTTGGCTGACCATGCTGCTCATCTTCCGCATCCTGCTGGTGACCCTGGTG
GGAGACGCCGTCTACAGTGACGAGCAGTCCAAGTTCACCTGTAACACCCAGCAGCCCGGA
TGCAACAACGTCTGTTACGACACCTTTGCACCTGTGTCACATCTGCGTTTCTGGGTTTTT
CAGATTGTGCTGGTATCCACTCCATCTATCTTCTACATCGTTTTTGTTCTCCATAAAATT
GCCAAGGATGAGAAGCTGGATGTCCAGAAAGGAAAGTTCATAATCCAAGCCCCCTCCAAA
AATAACTATGTTGAGCTTGGTAGCAGTTGTATGGAGGGCACCAGGGTGGAGCCCATCTAC
AGTCCCAAGTACATGGAGGAATGGGGCACAAAAGACCAAGAAGGAATGGAGCAAAGTCTC
CTTGACGAGGATTATGCTGAACTTGGTGAAGATCCAACCCAGCTATCGAGCCAAGTCCTA
CTCATTTACATTCTTCACGTGTTGTTACGTTCTGTTATGGAGATAACCTTTTTGGTGGGC
CAGTATTACTTGTTTGGTTTTGAAGTGCCGCACCTGTATCGCTGCGAGACCTATCCCTGC
CCAACACGCACTGACTGCTTTGTTTCTCGTGCCACAGAGAAGACAATTTTTCTGAATTTC
ATGTTTAGTATCAGCCTGGGCTGCTTTGTTCTCAACATCGCCGAGCTTCACTATCTTGGC
TGGGTGTACATATTCCGCATCTTGTGCTCAGCTTGTTCTACGTGCTGTACTCATGAGAGG
GATGCTAAGGGGCGGTACTCCCACCAGAACCCCTTGCTGCTGCAGCTGAAGCACTCCCTC
AGGGGGAGGCTGGTCCTACAGACGCCTGCCCCCAGGAGCCAGGAGAAGGCTCGAGGTCTG
CTCAGTCACGCCCCGGCCATCTCCTTTGAGACGGATTCCACCGTGGAATGCACCTCCAAG
AGGACTTTAGAGGAGAGGGACAAAGTGAAGCTCAAATTAGCCAACATGGCAAAACTAGGA
AGAACTAAGAAGTCCTGGTTATAA

>Gm-GJD3-G20235 Ensembl prediction. The sequence has been extended in 3’-direction (underlined) until stop codon.
ATGGGGGAATGGAGCTTCCTGGGTGATCTGTTTGAACACCTCCAGGCACACTCGCCCATG

CTGGGTCGCTTCTGGCTCTTCATCATGCTCGTGTTCCGCATTCTGATCCTGGGCACCGTG
GCGTCTGACCTGTTTGACGACGAGCAGGAAGAGTTCTCCTGCAACACCCTGCAGCCGGGC
TGCAAGCAGGTGTGCTACGACCATGCCTTCCCCATCTCCCTGTACCGGTTCTGGGTCTTC
CACATCATCCTCATCTCCACCCCGGCAATGCTCTACCTGATGTACGCCATGCACCACGTC
TCCAAGAAAAAGCCCTCCTCGTCCGCCGACGGCACCGCCTCCACCTGCAGCCAGGATAAC
CAAGAGGAGAGGCGCCTGAGGCAGCTCTACCTGGTGAACGTGGCCTTCCGCCTGATGGCG
GAGGTGGGCGTGCTGGTGGGCCAGTGGTGGCTGTACGGCTTCAAGCTGGAGGCCCAGTTC
CCCTGCAGCCGCTACCCCTGCCCGTACACGGTGGACTGCTTCACCTCCAGGCCCGCCGAG
AAGACCGTCTTCTTGGTGTTCTACTTTGGGGTGGGCGTGGTGTCGGCCGCCTCCAGCCTG
GTGGAGCTCCTCTACGCCGCTGTCAAGTGGTTCTGCCCCAGGAAACAGGGGCGGCGCGGC
ATGCCCGATAGGTCCTACGAGTCTCATAGCCTCAGCAACCTGCGGAAACAGGAGGAGGAG
GCGAACCTGCGGTTTGTGGGAGGGGGGAAGGCGCTGTCCGACAGCGCGCTGAGCAGCGCG
AGGATGAAGACAGGCCCCGTGAGGAGCAGCGGTGCGAGGAAGACCTCCAGCGTCGGACAC
AAGACCTCCAGGCTCCCCAGCAGCCGGTCCTTCATGGCGTGA

>Gm-NN-gjd4-G11373
ATGGGCATGCTGGATGCAGTCCTTGTCGCCATAAGCCACAACATCTCCTTTGTGGGTAAA
ACCTGGTGGCTACTCATGGTAGGCCTACGTCTTATTGTGGTCCTGCTGGCCGGCTTCACC
CTCTTCAGTGATGAGCAGGAGCGCTTCGTCTGCAACACCATCCAGCCGGGTTGCTCCAAC
GTCTGCTTCAACCTGTTGGCGCCCGTTTCCCTGTTCCGCCTCTGGCTGCTCCACATCGTC
CTCCTGTCTCTGCCGTACCTCATGTTCGCTACACACATCGCACACAGGCTCCTGTGGGAT
CCCAACTCTGGAGCGGGCTACGTGGCGATAAACCGCCATGGGAGTCAGGGAAGCCCTTGC
TCCACGCCAGAGATTTCCCAGTTCCTCCTTCTTCATCATCATCATCTTCCTGGGCAGGAC
CCCTCCCAGTGCGTGCGGCCCGTGCCGAGCTTCCACTATGCCTACCTATTGGTCGTGACC
GTGCACATCCTGATGGAGGCGGCCTTCGTGGCGGGTCACGTCCTGTTCTTCGGGTTCTTC
ATCCCAAGAAGCTTCCTGTGCTACGAGGCCCCGTGCACGTCGGGCGTGCAGTGCTACGTC
TCCCGGCCCACAGAGAAGACGCTGATGCTCGACCTCATGCTGGGCCTGGCCTGTCTGTCA
GTGGTGCTGAGCCTGGTGGACCTTGTGGCCGGCGCACGCCGGGCTCTGAGGCGGCGGAGG
AGAGCGACGTCTGTGTCGGAGGAGATGGGCAAAGGAGAGCAGAGCAGCGTGTCCTCCAAC
GTGAGTGGTGCAGGAGACCTCAACCTCCTCTTGAACAAGAGGATGGCCAACGGGTTTGAG
AGCGACATCCAAGCCACGCCTAGCTCCTCTACAGACAGTGTGCCTAATGTCGCCGCCGCG
CTGAAGGGCGAGGCAGAGGGTAAGGCTGGCAAGCTGACCGACGAGAAGGGCTTGCCGTGG
CAACACAGTGCCAACGGAAAGATCGGGTTCTCGACGAATCCAAAAGCCATGCCGCTGCCC
TTCGTCGTCCACAACCAGCAGAAACCTCCAGAGTTGGCTTCCCTGGACGGCAGCCTAGCA
CCGAGGCTGGAGAACTTCACCCCGGCTGACACTAGGAAACAGGGCCAGCTAGCCTCAATG
GAGTCCACCTCTACCTCCAGGCAGAACTCTACCCCCAGTGAGGGTCCTGACAAGAGGGCT
TGGGTTTAG

>Gm-NN-gjd4-G17736 Splice site. Underlined: sequence extended in 3-direction, and was further extended (lower case) by Blast against GenBank cod wgs
ATGAGTGGAGCCAGTGCCTGTGAAGTCATCTTCATCTCGGTCAGCCATAGCACCACACTG
ATGGGGAAGGTGTGGCTGGTCATCATGGTGTTCCTCCGGGTCCTCGTCCTGCTGCTGGCC
GGCTACCCGCTCTACCAGGACGAGCAGGAGCGCTTCGTGTGCAACACCATCCAGCCGGGC
TGCGCCAACGTCTGCTACGACCTCTTCGCCCCCGTCTCCCTCTTCCGCTTCTGGCTGGTG
CAGCTGGTGTCCGTCTCCCTGCCCTACCTCGTCTTCGTAGTCTACGTGGTCCACCGGGTC
CTGTGCGGGCTGACTGCCGGCTCTGCCCTTCCTCCTCCTCCCTCTGCACCTCCTCTTCCT
CTGGCACAGGACGTCGGGAGGGCTGGGAAGGACCCGCCCGGGACGGCggcggcggcggcg
gcggccctgcgtgccgagctcggccccgggcggcggtgcttcgcgggggcctacctcctg
cagctggtcttccgcatcctgttagaggtgggcttcggcgccgcccactacctgttt
ggcttccacatccccagccgcttcctgtgccagcaggcgccgtgcaccacccaggtggac
tgctacatctcgcggcccaccgagaagagcgtgatgctgtgcctcatgctgggcgccggc
gcgctctccctggggctcaacgcgctggacgtggtgtgcgccgtcaagcgctcggtgagg
cagagcgcgaggaggaggcggcggcggcggaggacgcgtgtggagaagctgtacgaggaa
gagcgctattacctcatcaacggtggaagccacagcggcagtggtggcggtggtggcggg
ggcagtgggggtgacggtagggggggaggaggaggacggcggccacgaggtcggttcactg
gtgcaccacgaggcggcgtcgacgggaggagcgtcccgccccggaagcttccggaagcgg
gggccgagcaaggcctcgagcgtctgcggccccgtccccgaccactgctcggtacggggc
tccctggtggccctcagcccgggggccccccgcggcctgaacaccaacaacggcaacaac
ggctacgggcaggcccagcgggaggaggcggcgccccacggcagcgacgtggcccacgga
ccctccgagcccccgccacgccccggtccatccgcgtcaacaagcagggccgcctcaag
ccccctcccccgccgcggcgggaccccgccgcgccgccgctggggtcgttcggggtcgtc
tccaaggcgacagcggacggcggcagcagaagggggcggtcagtacactcaggtggaactt
ggcggttgccaggacgacggccaggcggagaggtcggaatgggtgtga

>Gm-GJE1-G16314 Ensembl prediction. No modifications. Splice sites.
ATGTCTTTAAACTACATTAAAAACTTTTATGAAGGATGCCTCAGGCCTCCTACGGTGATA
GGCCAGTTCCACACCCTGTTCTTCGGCTCCGTGCGGATGTTCTTCCTAGGGGTGCTGGGC
TTTGCTGTGTACGGTAACGAGGCGCTCCACTTCAGCTGTGACCCCGACCGCAGAGAGCTC

AACCTCTACTGCTACAACCAGTTCCGACCAATCACGCCGCAGGTTTTCTGGGCACTACAG
CTGGTGACGGTGCTGTTTCCCGGGGCCGTGTTCCACCTCTACGCCGCCTGCAAGACCATC
GACCAGGAGGAGATTCTCCAGAGACCCGTCTACACCGTGTTCTACATCATCTCAGTGCTG
CTGCGCATCATCCTGGAGGTCATCGCCTTCTGGCTGCAGAGCCACCTCTTCGGCTTCCAG
GTCCACCCTCTCTTCATGTGTGACGCCATTGCGCTGGAGCGCTCCTTCAACGTGACCAAG
TGCATGGTCCCGGAGCACTTTGAGAAGACCATCTTCCTCAGCGCCATGTACACCTTCACC
GTCATCACCATCCTGCTCTGTGTGGCCGAGATCTTCGAGATCCTCTGCCGGAGGCTGGGG
TACCTCAGCAACAAGTGA

Suppl. Fig. 11. Japanese eel (*Anguilla japonica*) connexins.

Yellow: Conserved domains as defined by Cruciani and Mikalsen (2007)
Green: Conserved cysteine codons (cysteine signature)
Grey: 15 nt added at the ends of the conserved domains
Other colors are explained where necessary.

We here use the Japanese eel linkage groups (essentially equals a chromosome level assembly) as identification in addition to the naming of each sequence.

>Aj-NN-cx43-BEWY01000019 C, added to keep reading frame; nucleotide chosen according to BDQN01000172, AVPY01141929 (both *A. japonica*), AZBK01844958 (*A. anguilla*) and LTYT01001410 (*A. rostrata*).
ATGGGTGACTGGAGCGCTTTAGGGAGACTTCTGGACAAGGTCCAGGCCTACTCCACCGCT
GGAGGAAAGGTCTGGCTCTCTGTCCTCTTCATCTTCCGTATCCTGGTCCTGGGGACGGCC
GTGGAGTCCGCCTGGGGCGACGAGCAGTCGGCCTTCAAGTGCAACACCCAGCAGCCCGGT
TGCGAGAACGTCTGCTACGACAAGTCCTTCCCTATCTCGCACGTCCGCTTCTGGGTCCTC
CAGATCATCTTCGTCTCCACGCCAACGCTGCTCTACCTCGCCCACGTCTTCTACCTGATG
CGCAAGGAGCAGAAGCTGAACAAGAAGGAGGAGGAGCTGAAGGCGGTGCAGAACGACGGC
GGCGACGTGGACATACCGCTGAGGAAGATCGAGCTGAAGAAGGTCAAGCACGGGCTGGAG
GAGCATGGGAAGGTCAAGATGAAGGGCGCCCTCTTGCGCACCTACATCGTCAGCATCTTG
TTCAAGTCCATCTTCGAGGTGGGCTTCCTGATGATCCAGTGGTACATTTACGGCTTCTCG
CTGGCCGCCGTCTACACCTGCGAGAGGGACCCCTGCCCCCACAGGGTAGACTGCTTCCTG
TCCCGCCCCACGGAGAAAACGGTCTTCATCATCTTCATGCTGGTGGTGTCCCTGGTGTCC
CTCATGCTGAACGTCATTGAGCTGTTCTACGTCTTATTTAAACGGATCAAGGATCGCGTG
AAAGGGAAAGATAACCACTACCCCACCAGCGGTACCCTGAGCCCCACCCCCAAAGACCTG
TCCCCAACTAAGTACGCCTACTACAATGGCTGCTCCTCCCCCACCGCCCCCCTGTCCCCA
ATGTCACCTCCCGGGTACAAGCTGGCCACTGGGGAGAGGACCAACTCCTGTCGCAATTAC
AACAAACAAGCCAACGAGCAGAACTGGGCCAACTACAGCACCGAGCAGAACCGGCTGGGC
CAGAACGGCAGCACCATCTCCAACTCGCATGCGCAGGCCTTCGATTACCCCGACGACGGC
CAGGAGCACAAGAAACTGACCGCTGGCCACGAGCTGCAGCCATTGGCCCTGATGGACCCC
CGGCCGTCCAGTCGGGCCAGCAGCCGCATCAGCAGCCGGCCGAGGCCGGACGACCTCGAC
GTCTAG

>Aj-CXA1-BEWY01000007
ATGGGAGACTGGAGTGCTTTGGGGAGGCTCCTTGACAAGGTCCAGGCCTACTCCACTCCT
GGAGGAAAGGTCTGGCTCTCTGTCCTCTTTATCTTCCGGATCCTGGTCCTGGGGACGGCT
GTGGAGTCTGCCTGGGGGGATGAGCAGTCGGCATTCAAGTGCAACACCCAGCAGCCTGGC
TGCGAGAATGTCTGCTATGACAGATCCTTCCCCATCTCCCACGTTCGCTTCTGGGTCCTG
CAGATCATCTTTGTCTCCACACCAACACTGCTCTATCTTGGCCACATCTTCTACCTGATG
CACAAGGAGGAGAAGCTGAACAAGAAGGAGGAGGACCTGAAGGTTGTCCAGGGGGAGGGC
ATTGATGTGGATGCAGCACTACAGAAAATTGAGTTCAAGAGGGTCAAGTATGGGATAGAG
GAACACGGGAAGGTCAAGATGAAGGGTGCCCTCCTGCGCACCTATGCTGCAAGCATTGTC
TTCAAATCAGTCTTTGAGGTGGGCTTCCTGGTGATACAGTGGTACATATATGGGTTCAGC

CTGGCAGCTGTGTACACCTGCGAGAGGCTACCCTGCCCACACAGGGTCGATTGCTTCCTG
TCCCGACCTACGGAGAAAACGGTCTTCATCATATTCATGCTGGTGGTGTCCCTGGTATCC
TTGCTCCTCAATGCTATTGAGCTCTTCTATGTATTCTTCAAGAATGTCAAGGACCGGGTG
AAAGGGAAGGAAGACCACTTTCACAACAGCGGCACCCTCGGTTCCATTGTCAAGGACATG
TCGCCCTCCAAGTATGCCTACTACAATAGTTGCTCCTCTGCTGGAGTCCCCTTGTCTCCA
GTATCACCCCCAGGGTACAAGCTGGCAACTGGGGACAGGACCATGGGCTCCAGCCGCAAT
TATAACGAACAGGCAAATAAGCAGAACTGGGCCAATTACAGCACTGAGCAGAACCAGCTG
GGTCAAAATGGGAGTACCATCTCAAATTCCCATGCCCAGCCAGTCCATTTCCCTGAGGAC
ACTCAGGATCACAAAAAATTGACTGCTGGGCATGAACTTCTGCCCCTTGGGTTGCTGGAT
CCTCGGCCAATCAGCAGGGCCAGCAGTCGGATGAGCAGTCGGGCAAGGCCAGGTGACCTT
GATGTCTAA

>Aj-gja3-BEWY01000014
ATGGGCGACTGGAGCTTTCTGGGGCGGCTGTTGGAGAACGCGCAGGAACACTCGACGGTG
ATCGGCAAGGTGTGGCTGACGGTCCTCTTCATCTTCAGGATCCTGGTGCTGGGGGCGGCG
GCGGAGGAGGTGTGGGGCGACGAGCAGTCCGACTTCACCTGCAACACGCAGCAGCCCGGC
TGCGAGAACGTCTGCTACGACGAGGCCTTCCCCATCTCGCACATCCGCTTCTGGGTGCTG
CAGATCATCTTTGTGTCCACGCCCACCCTCATCTACCTGGGCCACGTGCTGCACATCGTC
CGCATGGAGGAGAAGCGCAAGGAGAAGGAGGAGGAGCTGCGCAAGGCCAGCAGGCTCCAG
GAGGAGAAGGAGCTCCTCTTTAAAAACGGAGCGGGCGGAGGAGGGGACGCCGGCGGCGGG
GGAGGCGGCGGAAAGAAGGAGAAGCCGCCGATCAGGGACGAGCACGGGAAAATCCGCATC
AGGGGGGCGCTGCTGCGCACCTACGTGTTCAACATCATTTTTAAGACCCTGTTTGAAGTG
GGCTTCATCTTAGGCCAGTACTTCCTGTACGGCTTTCAGCTGCGGCCGCTGTACAAGTGC
GCGCGGTGGCCCTGCCCCAACACCGTAGACTGCTTCATCTCCCGGCCCACAGAAAAGACC
ATCTTCATCATATTTATGCTTGTGGTGGCTTGCGTGTCCCTTTTGCTGAATTTGTTAGAG
ATCTATCACCTCGGATGGAAGAAGGTCAAGCAGGGCATGACCAACGAGTTTTCCCCCGAG
CGCGAGTCGCCGCCCCGCACCGACGCTGAGCCAGAGTCCGCGACCCCCGCCCCGAGAACT
GCCCCTCCAAACCTCAGCTACCCGCCGAACTACACGGACGTGACCGCGGGGGGCGCGTAC
CCCCTGCCGGCCGCCACGGCGGCCGAATTCAAGATGGATCCTCTGCAGGAGGACCTGCAG
GAGGCGCCCTCCTCCTTCTACATCAGCAACAACAACAACCACCGGCTGGCCTCCGAGCAG
AACTGGGCCAACCAGGCTACCGAGCAGCAGACTCGGGAGAGGAATCCAGGCTCCCCTTCC
CCCTCCTCTTCCTCCTCCTCCTCGTCCTCAACCTCCAGCGTCCGAGATGAGCTGCTGCAG
CAGCCGAAGGACGCCGCCTCGCCCGCCGCCACCTCCACCTCGAGCGGCGGGGGCTGGGGC
GGAGGGAAGGGCCCGTTGGAAGAGGGTCACATGACCACCATGGTGGAGATGCACGAGGCG
CCCGCGGCGGTCACGGCGGTAATGGCGGTCACGGACGCCCGGCGGCTCAGCAGGGCCAGC
AAGAGCAGCAGCGCCAGAGCCCGGCCCAACGACCTGGCGGTTTAG

>Aj-gja3-BEWY01000008 Likely assembly error. The indicated sequence is repeated on either side of a row of Ns.
ATGGGTGACTGGAGCTTTCTGGGGCGGCTGTTGGAGAATGCTCAAGAACACTCGACGGTG
ATCGGCAAGGTGTGGCTGACGGTCCTCTTCATCTTCAGGATCCTGATCCTGGGGGCGGCG
GCTGAGGATGTGTGGGGCGACGAGCAGTCCGACTTCACCTGCAACACCCAGCAGCCCGGG
TGCGAGAACGTCTGCTACGACGAGGCCTTCCCCATCTCCCACATCCGCTTCTGGGTGCTG
CAGATCATCTTCGTCTCCACGCCCACCCTCATCTACCTGGGCCACGTGCTGCACATCGTC
CGCATGGAGGAGAAGCGCAAGGAGAAGGAGGAGGAGCTGCGCAAGATCCGGCTGCAGGAG
GAAAAGGAGCTCCTCTTTAAGAACGGGGGAGGGGGCGGGGCGAATGCTNGGTGGAGGCGG
GGAGGGGGCGGCGGCAAAAAGGAGAAGCCGCCGATCAGAGACGAGCACGGGAAGATCCGC
ATCAGGGGTGCCCTGCTGCGCACCTACGTGTTCAACATCATTTTAAGACACTGTTCGAG
GTGGGGTTCATCCTGGGCCAGTACTTCCTCTACGGCTTCCAGCTGCGGCCGCTGTACAAG
TGCGCCCGCTGGCCCTGCCCCAACACGGTGGACTGCTTCATCTCCAGGCCCACGGAGAAA
ACAATCTTCATCATATTTATGCTTGTGGTGGCTTGCGTGTCCCTTTTGCTGAATTTGTTA
GAGATCTATCACCTGGGATGGAAGAAGGTCAAGCAGGGCATGACCAATGAGGCTTCACCC
GAGCATGAGTCGCTGCCCTGCGCTGACCCGGAGTCTGAGCCCGGCCCTGCTACCCCAATC
CCTGCCCCGAGAACTGTTGCCCCCGTCCTCTGCTACCCACCGAACTACACAGAGGTGACT
GCGGCGGGGGGCGGGGCGTACCCATTACCAGCGGGGCCCGCGGCCGAGTTCAAAATGGAG
GACCCGCTGGAGCTGATCTCCTCCTTCTACACCAGCAACAACAACAACCACCAGCAGCAG
CAGCACCAGCGGCGGGCCTTGGAGCAGAACTGGGCCAACCAGGCCACCGAGCGGCTGCAG
ACTCTGGAGAGGAAGCCCGAGTCCCCCTGCCCCTCCAAACCCTCTTCCTCCCCCTCGTCC
CCCTCCCCGACTTCCTCT

>Aj-NN-cx39.9-BEWY01000008
ATGGCTGACTGGAACTTGCTGGCGAAGCTTCTGGAAAAGGCCCAGGAGCACTCGACAGTG
GTGGGGAAGGTCTGGCTGACAGTCCTCTTCATTTTTCGCATCATGATTCTGGGCGCGGCT
GCGGAGAAGGTGTGGGGCGACGAGATGTCCGGCTTCACCTGCGACACCAAGCAGCCCGGT
TGCCAGAACGTGTGCTACGACAAGACCTTCCCCATCTCGCACATCCGCTTCTGGGTGCTG
CAGATCATCTTCGTCTCCACGCCCACGCTGATCTACCTCGGCCACATCCTCCACCTCGTG
CGCATGGAGGAGAAGGTGAAGCAGAAGGAGAAAGAGCAGGCTCAGCACGGGAACGGCCAC
GCCCACCCGCTGCTGCCCAACGGCAAGCCCAAGAAGCCGTCGGTCCGGGACGACCAGGGT

CGCATCCGCCTGCAGGGGGTGCTGCTGCGCACCTACGTCTTCAACATCATCTTCAAGACC
CTGTTCGAGGTGGGCTTCATTGTGGCCAGTACTTCCTCTACGGCTTCCAGCTGAAGCCG
CTCTACACCTGCGACCGCTGGCCTTGCCCCAACATGGTCAACTGCTACATCTCGCGGCCC
ACGGAGAAGACGATCTTCATCATCTTCATGCTGGTGGTGGCCTGCGTCTCGCTGCTGCTC
AACCTCATCGAGATGTACCACCTGGGTTTCAAGAGGTGCCAGCAGGGCATCCAGTACAGG
CGCTCAAAGCTGGCCTACGAGGAGGGCTTCAAGCCGCCCAGCGAGACCGCGGTGCCCTAC
GCGCCCGGCTACAACTTCTTCTCCCAGCACCCCACCGGCCCGTTCCCGCAAGGCCCCGGG
TACGACATGCCTGCCCTGGGGGAGTCCGAAGTCCCGATCAACCCGTACAGCACCAAGTCC
GCGTACAAGCAGAACCGCGACAACTTTGCCGTGGAGCGGGGCGACAGGACCGAGGAGGTT
TGCAACTCCAGGCCCGCCAGGGACTCAGGTTCGACCGGGGGCTCCGGCGAGGACTCCGCC
GCCGGGTCCGCTCCTGGCTCCGTTCCCGGGTCTCCGGCGGAGAAGACGAGGAGGTACAGC
CGATCCAGCAGGCGCAGCAACAACAGGACTAGAGAGGACGACCTGCGGGTCTGA

>Aj-CX39.9-BEWY01000015
ATGGGGGACTGGAACCTGCTGGGGAAGCTGCTGGAGAGTGCCCAGGAGCATTCCACGGTG
GTGGGCAAAGTCTGGCTCACCGTCCTGTTCATCTTCCGAATCCTGGTACTGGGTGCCGCC
GCCGAGAAGGTGTGGGGTGACGAGCAGTCAGGCTTCACTTGCGACACCAAGCAGCCCGGT
TGCCAGAACGTCTGCTACGACAAGACCTTCCCCATCTCGCACATCCGCTTCTGGGTGCTG
CAGATCATCTTTGTCTCCACACCAACGTTGATTTACCTGGGCCACATCCTGCACCTGGTG
CGAATGGAGGAGAAACACAAGCAGCAGGAGAAGGAGCGGGCTCAGCTCGCCCTGCAGAAC
GACAAGCAGCCGCTGCTCGGGAGCAAGGCCAAGAAGGCCTCGGTTCGGGACGAGCAGGGC
CGCATCCGCCTGCACGGGGTCCTCCTGCGCACCTACGTCTTCAACGTCATCTTCAAGACC
CTCTTCGAGGTGGGCTTCATCGTGGCCCAGTACTTCCTTTATGGCTTCGAGCTCAAGCCC
CTCTACACATGCAACCGGCCGCCGTGTCCCAACGTGGTCAACTGCTACATCTCCAGACCC
ACCGAGAAGACCATCTTCATCCTCTTTATGCTGGTAGTGGCCTGCATCTCCCTGCTGCTC
AACCTGGTGGAGATGTACCACCTGGGTTTCACCAAGTGCCGCCAAGGGCTGAGGTACAGG
CGCTCTCACCTCGCGTCTGAATTGGGCTCCAAGGCTCCCAGCGAGGCGGCGGTTCCTTTC
GTACCCAATTACAACTGTTTTCCCAGGCACCATCCTGTCCCTGGACCCTTTCAGACTAGT
GCCGGGTTCAGCCTCTCACCGCTCACAGAGCCGGACTCCATTTACCAGCCCTACAACAGC
AAGGCTTACAAGCAGAACAGGGACAACCTGGCCGTGGAGCGCAACAGTAAACCCGAGGAA
TGCGACCTGAAGGTGAAGAAGGGTTCAGGCTCGGCCCCGGGGTCGCCGGTGGAGAACCAG
CGTCGGCCCAGCCGCTCCAGCAAGCACAGCAACAATAAGACCAGACTGGACGATCTGAAG
ATCTGA

>Aj-NN-cx39.4-BEWY01000004
ATGTCCAGAGCTGACTGGGGTTTTCTGGAGCGTTTCCTGGAGGAGGGACAGGAGTACTCG
ACGGGGATTGGACGGGTGTGGCTGACCGTGCTCTTCCTGTTCCGCATGCTGATCCTGGGC
ACGGCCGCTGAGTCCGCCTGGGACGACGAGCAGTCCGACTTCGTCTGCAACACCCAGCAG
CCCGGCTGCGAGCTGGCCTGCTACGACCGCGCCTTCCCCATCTCCCACTTCCGCTTCTTT
GTCCTGCAGGTCATCTTCGTCTCCACGCCCACCATCTTCTATTTCATCTACGTGGCCCTG
CGCATGGGATGGGAGAGGAAGCGCGAGGTGGAGGAGGCGGGGAGGAGGAGGGCGGAGGAG
GGACGGGCGAGCCCCGACGAGGGGGCGCGGGGGGCCGGGAAGGCGGGCGGCGAGGAAGGG
GAGGCGAAGGGCGTGAGAGGCGAGCAGGGCGACGAGCGGCGGGAGCGCCCCAAGCTGAAG
GGCAAACTGCTCTGTGCGTACACGCTCAGCATCGTCCTCAAAGTGCTGCTGGAGGCGGGC
TTCATCCTGGGGCTGTGGTTCCTCTACGGCTTCGTCGTCCACGCCAAGTACGTGTGCCAG
CGCCCGCCCTGCCCCCACACGGTGGACTGCTTCGTCTCCAGGCCCACTGAGAAGACCATC
TTCACCGTGTACATGCAGGCCATCGCCGGGGTCTCCATGCTCCTCAACGTCGTGGAATTT
CTCTACCTTGCGCAGCACACTGTCACCCACTACCTGGAGAAGAAGTACCTGGGCAAAACT
CCAGTCACTCTGCAAATAGACAGAGAGCCCTCACAGCTGGACCTGCCCAGGGAGTCCGCT
GTGCACTACCAGGAGAAGGGACACCTGTGCCTGCCTGGGGCTGGGTTTCCCCAGCCGTAC
CAGGAGTACGTGGAACCCGAAATTGAGCTCAGCTGGGGTGTCGGAGAACAGGGGACAACC
GAAGGCTCGCTCTCAAACCCGCTCCCCAGCTATTCGACTTGCATGAGGGCCATGAAATCC
ACTTCGAGCAGAGTGTCCTCAAAGGCATCCTCTCATAGAGAACAAAGCAAGAGGTCAAAG
AAAGGGAATTTGAAACAATATGTCTGA

>Aj-NN-cx39.4-BEWY01000007
ATGTCGAAGTCAGACTGGACCTTCCTGGAGCTCCTGCTGGAGCAGGGGCAGGTGCACTCC
ACAGGCGTGGGGAAGATGTGGCTGACGGTGCTCTTCCTGTTCCGCGTGCTGGTGCTGAGC
ACGGCGGCTGAGTCGGTGTGGGGCGACGAGCAGTCCGACTTCGTCTGCAACACGCAGCAG
CCGGGCTGCGAGGCAGTCTGCTACGACAAGGCCTTCCCCATCTCCCACTTCCGCTTCTTC
ATCCTGCAGGTCATCATCGTCGCCTCGCCCGCCATCTTCTACCTCAGCTACGCCGCCCTG
CACGCCAGGTGGCAGAGGAAGAGGGAGGAGGAGGAGGAGAAGGAGGAGGAGAGGAGGAAG
AGGGCGGAGGAGGCGAAGGGAAGGGACTCGGAGGTGGAGAAGAAGGAGAAGGAGGGAGGG
CGGGAGAGAGAGGGCGCAGGGCAGGGGGGGAGGGTACCCCCGAACGCGCCCAGGCTGAGA
GGCAAGCTGCTCCGGGTGTACCTGTGCGTCACCGTGCTCAAGCTGCTGCTGGAGGCGGCC
TTCATCCTGGTGCTGTGGCACCTGTACGGCTTCACCGTGCCTGCCCGCTACGTGTGCCAG
CGCTGGCCGTGCCCGCACACGGTCGACTGCTTCGTGTCGCGGCCCAAGGAGAAGACCGTC
TTCACCGTGTACATGCAGGCCATGGCGGGCGTGTCGCTGCTCTTCAACCTGCTGGAGGTG

TGCGTGCTCCTCCGCCGATACTGCTGCCCGCCCCGGTCAGCTGGGCCCCCCCGCGCCCCT
GCTGCGCCCCATCCCACAGAGAGGGCTCGCCTTCACCTGCCCACAGGCAAGGGCGGGGCC
CCTCCGGGATGGGAGGCTCGGATTAGCTGGGGTGCCCAGCATGCTTTCGGGGCGGAGCCA
GCCTTACTTGCCCCTCCCCCTTCTCTGACCCGTCTCCCTCAGGAGGCCCCCAGGGGCAGC
TGGTCCACACAGTGGCGTTATCCCCAGGTCTACCAGCAGGGCGGCGCCTCAATATGA

>Aj-CXA5-BEWY1000014
ATGGGTGATTGGAGTCTCCTGGGAAACTTCCTTGAAGAGGTGCAGGAGCACTCGACTTCG
GTGGGCAAGGTCTGGCTGACCGTCCTCTTCATCTTCCGCATCCTGGTCCTGGGCACGGCG
GCGGAGTCCTCCTGGGGCGACGAGCAGTCGGACTTCATGTGCGACACCAAGCAGCCTGGC
TGCGTCAACGTGTGCTACGACAAGGCCTTCCCCATCGCCCACATCCGCTACTGGGTCCTG
CAGATTGTGTTCGTCTCCACCCCCTCCCTCATCTACATGGGCCACGCCATGCACACGGTC
CGCATGGAGGACAAGCGGCGCCAGAGGGAGCAGGAGCAGGGTGGGGAAGGGGGCGGGGCT
GAGGAGAAGGGCTACCTGGAAGAGAGGGAGGCTGGGAAGCCCGAGCCCTGGGGAAAGATT
CGCCTGAAGGGGGCGCTGCTGAAGACGTACGTGCTCAGCATCCTGATCCGCACCGTCATG
GAGGTGACCTTCATCGTGGTGCAGTACATGATTTACGGGATCTTCCTCAACTCTCTCTAC
CTCTGCGAGGCCTGGCCCTGTCCAAACCGGGTCAACTGCTACATGTCCCGCCCCACTGAG
AAGAACGTCTTCATCGTCTTCATGCTGGCAGTGGCGGGCGTGTCGCTCTTCCTGAGCATA
GTGGAGCTCTACCACCTGGCCTGGAAACAGTCCAAAAAGTGCCTGAGGGCCTATGCTGCC
TCCCACGCCCTGGACAGCACGCCCTCTATGGTGGTGCAAGTTTCCCCAGAGACCAGTGCG
CCACCCCACACCTCCTGCACTCCGCCCCCTGACTTCAGTCAGTGCCTGGCACCGCCACCC
GCTCACACCCACCCCAACTGCCACCCCTTCAACAACAGGATGGCCCACCAGCAGAACTCT
GCCAACCTGGCGACCGAACGCCGCCACAGCCACGGCAACCTGGAGGGGGAAGACTTCCTG
GAGATGAGCTCCGTGGAGGGGGCAGAGACTCCAGCTCCGCTGCTCCACGCCACATTCCTC
AAGGACAAGCGCCGCCTCAGCAAGACCAGCGGCTCCAGCAGCCGCGCGCGGCCTGACGAC
CTGGCCGTGTAG

>Aj-NN-gja5-BEWY01000008
ATGGGTGATTGGAGTCTTATGGGAACCTTCCTTGAAGAGTTGCAGGAACACTCGACTTCG
GTAGGCAAGGTCTGGCTGACCGTCCTCTTCGTCTTCCGCGTCCTGGTCCTGGGCACGGCA
GCGGAGTCCTCCTGGGGCGACGAGCAGTCGGACTTCATGTGCGACACTGAGCAGCCCGGC
TGTGAGAATGTGTGCTACGACAAGGCCTTCCCCATCGCCCACATCCGCTACTGGGTCCTG
CAGATTGTGTTTGTCTCTACCCCCTCCCTCATATACATGGGTCACGCCATGCACATACTG
CGGGTAGAGGAGAAGCGCAGGCGGAGGGAGCTGGAGGACAAGGGTGGGGGTGAGGTCGGG
GGTGGGGGTGGGGAGAAGGAGTACCTGGAGGGGAAGGAGTCTGGGAGGGCGGAGGACACG
GGGAAGTTGCACCTGAGGGGGGCACTGCTGAAAACGTATGTGCTGAGCATCCTGATCCGC
ACTGCGATGGAGGTAACCTTCATTGTGGCGCAGTACATGATCTATGGAGTCTTCCTCAAT
CCGCTGTATGTCTGTGAGGCCTGGCCCTGTCCCAACCCGGTCAACTGCTACATGTCTCGG
CCAACAGAGAAGAACGTATTCATTGTCTTCATGCTGGTGGTGGCGGGCGTGTCCCTGTTC
CTGAGCGTGGTGGAGCTCTACCACCTGGCCTGGAAGCAATCAAAGCGATGCTTTCGAGAC
TACCTGGCCTCCCGCGCTCGGCAGCCCAAACCTGCCCCCGTGGCACCCATTGGCTGCGAG
CTCGAGACTCCCCTGCAGGTCTCCCGCACCCGCACCCCACCCCCTGATTTCGACCAGTGC
CTGGCGACGGCACTACCCCACTCTCACACTGGCCATGCCCACCCAAGCTGCCAACCGTTC
AACAACAGGATGGCCCACCAGCAGAACTCCGTCAACCGGGCAACCGAGCGCCACCACAGC
CACGACAACCTGGAGACGGTGGACTTCCTGCAGATGAGCTACACGCAGGAAACCGAGGCA
GCTGACACCTGTGGCCTGCCCTCGGCTCCGCCTCCTGCTCTGGCCCTGAACAACGGCTTC
TTAAAGGACAAGCGGCGCCTCAGCAAGACCAGTGGCTCCAGCAGCCGAGTGAGGCCGGAT
GACTTAGCCGTGTAG

>Aj-NP-gja8-BEWY01000014
ATGGGTGACTGGAGCTTCTTGGGGAACATTTTAGAGGAAGTAAATGAACACTCGACGGTG
ATAGGGAGGGTGTGGCTGACTGTGCTCTTCATTTTTAGGATTTTAATCCTGGGCACGGCC
GCTGAGTTTGTTTGGGGGGACGAGCAGTCGGATTATGTTTGCAACACCCAGCAGCCGGGT
TGCGAGAACGTCTGCTACGATGAGGCTTTCCCCATCTCCCACATCAGGCTGTGGGTGCTC
CAGATCATCTTCGTCTCCACGCCCTCGCTGGTGTACGTGGGCCACGCCGTGCACCATGTG
CACATGGAGGAGAAGCGCAAGGAAAGGGAGGAGGCGGAGATGAACCGCCAGCAGGAGATG
AACGAGGAGAGGCTGCCTCTGGCGCCCGACCAGGGCAGCGTCAGGACCACCAAAGAGACC
AGCACCAAGGGCAGCAAGAAGTTCCGCCTGGAGGGCACCCTGCTGAGGACCTACATCTGC
CACATCATCTTCAAAACCCTGTTCGAGGTGGGCTTCGTGGTGGGGCAGTACTTCCTCTAC
GGCTTCCGCATCCTGCCCCTGTACCAATGCAGCCGCTGGCCCTGCCCCAACACCGTCGAC
TGCTTCGTCTCGCGTCCCACTGAGAAGACCGTCTTCATCATCTTCATGCTGGCGGTGGCC
TGTGTCTCCCTCTTCCTCAACTTTGTGGAGATCAGCCACTTGGGCCTGAAGAAGATTCAC
TTTGTTTTCCGCAAGACCCCCCAGCAGCAAGCAGAGGGGGGGCTTGTCCCAGAGAAAAGC
CTGGCCTCCATGGCCGTCTCTTCCATCCAGAAGGCCAAGGGCTACAAACTGCTGGAAGAG
GACAAGCCCGCGTCCCACTTCTTCCCCCTGACAGAGGTGGGAATGGAGGCTGGCAGACTC
CCCACATCATTTGAGACATTGGAGGAGAAGCTGGAGGAGGCAGGACCCCCGGAAAATATA
TCTAAGGTATATGATGAGACCTTGCCCTCCTACGTTCAGACCACTGAGGCAGAGGAGGGG
GTGCTACAGGAGGAGGAGGAAGAGGAGGATGAAGAGGAACCTCCTGCTGAAGCTGAAGGG

GAGGCCACTGAGACAATAGAAGACACCAGACCGCTGAGCAGTTTGAGCAGAGCCAGCAGC
AGGGCCAGGTCAGATGATTTGACAGTATGA

>Aj-gja8-BEWY01000008
ATGGGTGACTGGAGCTTCTTGGGAAACATTTTAGAGGAAGTGAACGAGCACTCGACAGTG
ATTGGCAGGGTGTGGTTGACCGTGCTCTTCATCTTCAGGATCCTGATCTTGGGCACAGCT
GCTGAGTTTGTCTGGGGGGACGAGCAGTCGGATTATGTTTGCAACACCCAGCAGCCGGGT
TGCGAGAACGTCTGCTATGACGAGGCTTTCCCCATCTCCCACATCAGGCTGTGGGTGCTC
CAGATCATCTTCGTCTCCACGCCCTCGCTGGTGTACGTGGCCACGCCGTGCACCATGTC
CACATGGAGGAGAAGCGCAAGGAAAGGGAGGAGGCGGAGATGAACCGTCAGCAGGAGATG
AATGAGGAGAGGCTGCCTCTGGCGCCCGACCAGGGCAGCGTCAGGACCACCAAGGAGACC
AGTACCAAGGGCAGCAAGAAGTTCCGCCTGGAGGGCACGTTGCTGAGGACGTACATCTGC
CACATTATCTTCAAGACCCTGTTTGAGGTGGGCTTCGTGGTGGGACAGTATTTCCTCTAT
GGCTTCCGCATCCTGCCCCTCTACAAGTGCAGCCGCTGGCCCTGCCCCAACACTGTCGAC
TGCTTCGTCTCGCGCCCCACCGAGAAGACCGTCTTCATCATCTTCATGCTGGCGGTTGCC
TGCGTCTCCCTCTTCCTCAACTTTGTGGAGATCAGCCACCTCGGACTGAAAAAGATCCGC
TTTGTTTTCCAGAAGCCCCCCCAGCAGCAAGCAGAGGGGGGGCTGGTTCCAGAGAAGAGT
TTGACCTCCATGACTGTCTCTTCCATCCAGAAGGCCAAGGGCTACAAACTGCTGGAGGAG
GACAAGCCTGTGTCCCACTACTTCCCCCTGACAGAGGTGGGGATGGAGGCAGGCAGGCTG
CCGACACCCTTTCAGACTTTTGAGGAGAAGTCCTGCGTGGATGAGGTAGGGCCCCCTGAA
GACATGTCCAAGTTGTGTGATGAGACCCTGCTCTCCTATGTCCAGACCACTGAACAGGAG
GAGGAGCAGAAGCAGGGGCAGGAACAGGACAATGAGGAGGAGCAGCAAGATCAGGAAGAA
GAGGACGAAGAAGAGGAAGAGGGGGAGAAGCCACCCACCGAGACTGATGTGGAGGCTACT
GAGACGATAGAAGACACCAGACCGCTAAGCAGCTTGAGTAAAGCAAGCAGCAGGGCCAGG
TCAGATGATTTGACAGTATGA

>Aj-NN-gja10-BEWY01000019
ATGGGGGACTGGAACTTGCTTGGAAGTATCTTAGAAGAAGTCCATATTCACTCCACCATA
GTGGGAAAAATTTGGCTCACAATTCTTTTCATATTTCGAATGCTTGTTCTCGGCGTTGCG
GCTGAAGACGTCTGGGATGACGAACAAAGCGAGTTCATCTGCAACACGGAGCAACCCGGA
TGCCGGAACGTCTGCTACGACAAAGCGTTCCCAATTTCTCTTATACGGTTCTGGGTGCTG
CAAATAATCTTCGTGTCATCTCCATCGCTGGTATACATGGGTCACGCATTATACAAGCTC
AGGGCGCTTGAAAAAGAGAGGCATAAGAAAAAGGCTCAACTGAAGGCGGAGTTGGAAGAG
GTCGAGCCTAGTCTGGAGGAGCACAAAAGAATCGAGAGGGAGCTGAGAAAACTAGACGAG
CAGAAAAAGGTCAGTAAAGCTCCTCTGCGGGGTTCGTTATTGCGCACATATGTTTTCCAT
ATCCTGACGAGATCAGTGGTGGAGGTGGGCTTCATAGTGGGCCAATATGTCTTGTATGGA
ATTGGACTAGATCCCCTGTACAAGTGCGAGAAGGTACCATGTCCAAATAGCGTGGATTGC
TTCGTTTCGCGCCCAACGGAAAAAACTATTTTCATGGTCTTCATGATAGTAATCGCATGT
GCTTCTCTCTGTCTGAACCTTCTTGAAATTTCCCACTTGGGAGTAAGAAAATTAAAACAA
AATCTGTTTGGTGAGACGGGTGGAGACGACGACAGTGCTTGCAAATCAAAGAAAAACTCA
ATGGTTCAGCAAGTATGTGTCCTCTCGAACTCATCGCCGCACAAAATGGTGCAATTAACA
ATAATGCCAGATGGACAAATTGATCCTTTTCCGGTTTACATGGCTTCTGCTGCATCTCGG
CCGAGCCAGGAAATGCAGAGATACAACGGCATCACCGGGGACCGCGACCAAGTACATTTA
TCGGATCAGCACCCCAGACAGCTCCTCAGGCCCAGCCAAGACGAGATCCACGCTCTACGC
ATGCTTGCCTCCACGGAACGTCGCAAAACTTCGGACAACCGGGATCATTTATTCAACAGC
GATGACTCGAATGGCAGCAATGGGCCTAAGAGTTCAGGCCAAGCACCGCAAGCGAAACAA
TCATCACAGTCTAGCCACATGGAATTGCCAGCAGCCTTGCGCAATGCTTTGCGCAAACAG
AGCCGCGTTAGCTGCTTGAACGGGGACCGAAGTGATTCTCCCGACAGTGGTCACTATCCT
TCCAGTAGAAAGGCCAGTGTCATGTCTCGTGGCATGTCTGAAGGCAAGCTAACAAGTTCA
TCCAGTAACCAAGCTTTGGAAAGTGGCTCCGGCTCTGAATCTAAACGTCTGAGCCAAGGA
GAGAGTCCACCGATTACCCCGCCTCCCGCCAATGGACGGAGAATGTCAATGGCAAGTATA
GCCTAA

>Aj-NN-gja9-BEWY01000068
ATGGGGGACTGGAACTTCCTGGGCGGGATCTTGGAGGAAGTGCACATTCACTCCACCATG
GTGGGCAAGATCTGGCTGACCATCCTCTTCGTCTTCCGCATGCTGGTGCTGGGCGTGGCG
GCGGAGGACGTGTGGAACGACGAGCAGTCGCACTTCGTCTGCAACACGGAGCAGCCGGGC
TGCCGCAACGTGTGCTACGACCGCGCCTTCCCCGTGTCTCTCATCCGCTACTGGGTGCTG
CAGGTCATCTTCGTGTCCTCGCCCTCGCTGGTCTACATGGGCCACGCGCTCTACCAGCTG
CGCGCCCTGGAGAAGGAGCGGCAGCGCAAGAAGGCGCAGCTCCGGCGCGAGCTGGAGGCG
GCGGAGGCGGAGCCCGCGGAGGCGCGCCGGCGGCTGGAGCGGGAGCTGCGCCAGCTGGAG
CAGGGCCGGCTGAACAAGGCGCCGCTGCGCGGCTCGCTCCTGCGCACCTACGTGGCGCAC
ATCCTGACCCGCTCCGCCGTGGAGGTGGGCTTCATGCTGGGCCAGTATCTGCTCTACGGC
CTCCGCCTGGAGCCGCTCTACAAGTGCGAGCGCGAGCCCTGCCCCAACGCCGTCGACTGC
TTCGTGTCGCGGCCCACGGAGAAGAGCGTCTTCATGGTGTTCATGCAGGGCATCGCCGCC
GTCTCCCTCTTCCTCAACATCCTGGAGATCCTGCACCTGGGCTACAAGAGGCTGAAGAAG
GGCCTGCTGGACTACTACCCGCACCTGCGGGACGACCTGGACGACTACTGCGTCAGCCGG
TCCAAGAAGAACTCGGTGGTGCAGCAGGTGTGCGCGGGCCGGAAGGCCACCATCCCCACC

GCGCCCAGCGGCTACACCCTCCTGCTGGAGAGGCAGGGCAACGGGCCCACCTACCCCGTG
CTGGAGACCTCCTCCACCTTCGTCCCCATCCAGGGCGACCCCGCCGCCTGCAAGACGGGC
CTGGACGTCGTCCTCCTCAAGGAGGCGTCGCCCGGCCCCGCCGAGCCCAACGGCGCCTCC
AAAACCAACACCAGCAGCGAGACGCGGTCGCCGCCCGCCGACAAGCAGGGCGACTCGGA

>Aj-NN-gja9-BEWY01000007
ATGGGAGACTGGAACTTCCTGGGCGGATCCTGGAGGAGGTGCACATCCACTCCACCATG
GTGGGGAAGATCTGGCTCACCATCCTCTTCGTCTTCCGCATGCTGGTGCTGGGCGTGGCG
GCGGAGGACGTGTGGAACGACGAGCAGTCCGAGTTCGTGTGCAACACGGAGCAGCCGGGC
TGCCACAACGTGTGCTACGACCGCGCCTTCCCCGTGTCGCTGGTGCGCCTGTGGGTGCTG
CAGGTCATCTTCGTGTCCTCGCCCTCGCTGGCCTACATGGGCCATGCCCTGTACCGNNNN
NNNNNNNNNNNNnnGCGGCGGCGGCTGGAGCGGGAGCTGCGCGCGCTGGAGCGGCGGCGG
ATCGACAAGGCGCCGCTGCGCGGCTCGCTCCTGCACTCGTACGTGGCGCACATCCTGACC
CGCTCCGCCGTGGAGCTGGGCTTCATGCTGGGCCAGTACCTGCTCTACGGCTTCCGCCTG
GAGCCGCTCTACAAGTGCGAGCGCGAGCCCTGCCCCAACGCCGTCGACTGCTTCGTGTCG
CGGCCCACGGAGAAGAGCTTCTTCATGGTGTTCATGCAGTGCATCGCCGGCGTCTCGCTG
CTCCTCAACCTGCTGGAGATCCTGCACCTGGCCTACGGCCGCGTGAGGACGGGCCTCCTG
GACTACTGCCCGCAGCTGCAGCGCGACGAGCTGGACGACTGCTACGCGGGCCGGCCGCAG
GTGTGCGTCGCCCCTGTCCCCCCCACCGCCCCACCTGACNNNN

>Aj-NN-cx34.5-BEWY0100019
ATGGGAGAGTGGGACTTTCTGGGACGGCTTCTGGACAAAGTCCAGACCCACTCCACGGTC
ATCGGGAAGGTCTGGCTGACCGTCCTGTTCGTCTTCAGGATCCTGGTCCTGGGGGCCGCG
GCGGAGAGGGTGTGGGGGGACGAGCAGTCCGACTTCGTCTGCAACACGGAGCAGCCCGGG
TGCGAGAACATGTGCTACGACCACGCCTTCCCCATCTCCCACGTCCGCATCTGGGTGCTG
CAGATCGTCTTCGTCTCCACGCCGACCCTGGTCTACCTGGGGCACGTCCTGCACGTGGTC
CACATGGAGAAGAAGTACAGGGAGAGAACGCGTAAGCAGGCCGAGGAGGAGCTCAGCAGC
CTGATCCTGAGGAACGGGTACAAGGTCCCCAAATACTCAGACAGCGAGGGGAAGGTCAGC
CTGCACGGTCGACTCCTTCAGAGCTACCTGGTGAACCTGCTCTTCAAGATCTTGCTGGAA
GTGGGGTTCATCCTGGGGCAGTACTACTATTACGGCTTCACCTTGCAGGCCCGCTACGTC
TGCAGCCGGTTCCCCTGCCCGCACCAGGTGGACTGTTTCCTCTCCAGGCCCACGGAGAAG
ACCATCTTCATTTGGTTCATGCTGGTGGTCGCCTGTGTCTCCCTGGTCCTGAACCTGGTC
GAGATCCTCTATCTGTGCACCAGGGCCGTCACCAAGTGCGTGGACAAGAAGCAGGGTTAC
ATGGTCACTCGCGTAACTCCAGTCCTGCAGAGAAACGAGTTCAAAAACAAGGACCTGGCC
ATCCAGAACTGGGTCAACCTGGAGCTGGAGCTACAGGGAAGGAAGCTAGGCAGTGGGGTC
ACTAAAAGCCTGGAATCGGAGGACGTAAGCACCAACATGGAGGAGGTCCACATCTGA

>Aj-NN-cx32.3a-BEWY01000019
ATGGGCGACTGGTCATTTCTGTCAAAACTGCTGGACAAAGTGCAATCGCACTCCACGGTC
ATAGGGAAGATATGGATGAGCGTGCTGTTCATCTTCAGGATCCTGGTTCTGGGGGCGGGA
GCAGAGAGCGTGTGGGGGGACGAGCAGTCGGGGTTCATCTGCAACACCCAGCAGCCCGGT
TGCGAGAACGTGTGCTACGACCACACCTTCCCCATCTCCCACATCCGCTTCTGGGTCATG
CAGATCATCTTCGTCTCCACGCCGACCCTGATGTACCTGGGCCACGCCATGCACGTCATC
CACGAGGAGAACAAGCTGAGGCAGCACCTGAGCCAGAACGGCAAGTGCCCCAAGTACACG
AATGACAGGGGCAAGGTCAAGATCAAGGGGAACCTGCTGGGCAGCTACCTGACGCAGCTG
TTCTTCAAGATCGTCTTCGAGCTGGGCTTCATCGTGGGCCAGTACTACCTGTACGGCTTC
ATCATGGTTCCCATGTTCCCCTGCTCCAGGAAGCCGTGCCCGTTCACCGTGGAGTGCTAC
ATGTCCCGCCCCACCGAGAAGACCATCTTCATCATCTTCATGCTGGTGGTGTCCTGCGTC
TCCCTGCTCCTGAACGTGGTGGAGGTGTTCTACCTGATCTGCACCCGGGTGGGGTGCCGG
ACCAGGAGGCGCACGCTTCACCGCACCTCCCCCGAAAACCCCGCCGGCCTAGGGTGGCAG
GGCCGCGAAGAGGCGCAGCGGCAGAACGCGGTGAACATGCAGTATGAAAACGGGCAGAGT
CCGAGCCTCGGGGGCAGCCTGGAAGGAGCCAAGGAGGAGAAAAGCTTACTGGCCGAAAAA
TAA

>Aj-NN-cx32.3b-BEWY01000019
ATGGGAGACTGGTCATTTCTTGCAACGCTGCTGGACAAAGTCCAGACCCACTCCACGGTC
ATCGGGAAGGTCTGGCTCACCGTCCTCTTCATCTTCAGGATCCTGGTTCTCTCTGCCGGA
GTGGAGAAGGTGTGGGGGGACGAGCAGTCGGGATTCATCTGCAACACCAAGCAGCCCGGA
TGTAAGAACGTGTGCTACGACCACGCCTTCCCCATCTCCCACGTCCGCTTCTGGGTGATG
CAGATCATCTTCGTCTCCACGCCGACGCTCCTCTACCTGGGCCACGTCATGCTCATCGTG
CACAAGGAGAACAAGCTGAGGCGCCATTTGCAAAGCCAGGAGGGCCACGCGCTGAAGGCG
CCCAAATACAGCGACGAGAGGGGGAAGGTTCAGATCAAGGGCGATCTGATGGGTAGCTAT
TTGGTGAACGTGTTTTTTAAGATCTTGTTCGAGTCAGCTTTCATCGTGGGCCAGTATTAT
CTTTACGGTTTTATGCTTGTGCCCATGTTCGAGTGCTCCAGAACTCCCTGCCCGTTTACC
GTGGAGTGTTACATGTCACGACCCACGGAGAAGACCATCTTCATCATCTTCATGCTGGTG
ATGGCCTGCGTGTCTCTGCTGTTAAACGTGGTGGAGATCTTTCACTTGATTTGCACCAGG
TTCAAGGGTAGATCTCGCAGACACCGGAAAGATCAGGTTATTCCAGTCAGCATTCCTCTT
CACAGCGATGCAGTTCTGCAGAATAGGGAGAATGCGCTGCATGATAAAGTCGACCTCAGC

TTTGGAGCTGGACAGAATGATAATCAGAGCACAGCGTAG

>Aj-NN-cx27.5a-BEWY01000008
ATGAACTGGGCGTCCTTTTATGCCGTGATCAGCGGCGTAAACAGGCACTCCACGGGCATC
GGCCGCATCTGGCTGTCCGTCCTCTTCATCTTCCGCATCCTGGTGCTGGTGGTGGCGGCC
GAGAGCGTGTGGGGCGACGAGAAGACGGGCTTCACCTGCAACACCCAGCAGCCCGGCTGC
AACAGCGTCTGCTACGACCAGTTCTTCCCCATCTCGCACATCCGCCTGTGGGCCCTGCAG
CTCATCCTGGTCTCCACCCCGGCCCTGCTGGTGGCCATGCACGTGGCCCACCGTCGCCAC
GTCGACAAGAAGCTCCTCAAGCTGTCGGGCCGCAGCGCCAGCGCCAAGGACATGGAGGAG
ATCAAGAGCCAGAAGTTCAAGATCACCGGCGCCCTCTGGTGGACGTACACCGTCAGCATC
GTTTTCCGCATCGCCTTCGAGGCCGCCTTCATGTACATTTTCTACCTCATCTACCCCGGC
TACAAGATGCTGCGGCTGGTCAAGTGCGACTCGTACCCGTGCCCGAACACCGTGGACTGC
TTCGTCTCCCGGCCCACGGAGAAGACCATCTTCACCATCTTCATGCTGGCGGCGTCGGGC
GTGTGCGTCCTGCTGAACGTGGCCGAGCTGGCCTACCTGGTGGGCCGGGCCTGCGTCCGA
AACCTCCGGCGCACGGAGGCCCCGCCCAAGGGCGTGTGGCTGTCCCAGAAGCTCTCCTCC
TACAGGCAGAACGAGATAAACCAGCTGATCGCCGAGCACTCGCTCAGGGCCAAGCTCAGC
GGAACCCGCCGGAACCCCGTGGACAAGGAGGAGAAGTGCGCGGCGAGTTAG

>Aj-cxb1-BEWY01000015
ATGAACTGGGCGTCCTTTTATGCCGTTATAAGCGGCGTAAATAGGCACTCCACTGGCATC
GGCCGCATCTGGCTCTCCGTCCTCTTCATCTTCCGCATCCTGGTCCTGGTGGTGGCGGCC
GAGAGCGCCTGGGGCGACGAGAAGGCCGGCTTCACCTGCAACACCCAGCAGCCCGGCTGC
GACAGCGTCTGCTACGACCAGTTCTTCCCCATCTCGCACATCCGCCTGTGGGCCCTGCAG
CTCATCCTGGTCTCCACCCCTGCCCTCCTGGTGGCCATGCATGTGGCTCACCGCCGCCAC
ATCGACAAAAAGATCCTCAAGCTGTCCGGGAAGGGCAGCCCCAAGGACCTGGAGCAGATC
AAGAGCCACAAGTTCAAGATCACCGGCGCTCTCTGGTGGACGTACATGATCAGCCTCGTA
TTTCGCGTCCTCTTCGAGGTTGGATTCATGTACATATTCTACATGATCTACCCGGGCTAC
AAAATGTTTCGTCTCGTCAAGTGCGACTCCTACCCCTGCCCCAACACGGTGGACTGCTTC
GTGTCCCGTCCCACTGAAAAGACTATCTTCACCGTCTTCATGCTGGCGGTTTCCGGGGTG
TGTATCCTGCTCAACATCGCTGAGGCGCTGTATCTAGTGGGAAGGGCATGCAGCAGGCAC
TTCCAGAATGCTGAGGACTCACCCATGGGAGCTTGGATCACCAAAAAGCTTTGCTTTTAG

>Aj-NN-cx30.3a-BEWY01000008
ATGAGCTGGGGGGCGCTGTACGCCCAGTTGGGCGGAGTCAACAAGCACTCCACCAGCCTG
GGGAAGATCTGGCTGTCGGTCCTCTTCATCTTCCGCATCATGATCCTGGTGCTGGCGGCG
GAGAGCGTGTGGGGCGACGAGCAAGCCGGCTTCACCTGCAACACGCAGCAGCCTGGCTGC
AAGAACGTCTGCTACGACCACTTCTTCCCCGTCTCGCACATCCGGCTCTGGTGCCTGCAG
CTCATCTTCGTCTCCACGCCGGCGCTGCTGGTGGCCATGCACGTGGCCTACCGCAAGCGC
GAGACCAAGCGGAGCATCATCCGCGCCCACGGCGACAAGGTGCAGGACGACCTGGAGAGC
CTGCGCAAGCGCCGGCTGTCCATCACCGGGCCGCTGTGGTGGACCTACACCTCCAGTCTG
TTCTTCCGGCTGATCTTCGAGGGCAGCTTCATGTACATTCTGTACTTCATCTACAATGGC
TTCCAGATGCCGCGGCTCGTCAAGTGCGAACAGTGGCCCTGTCCAAACAAGGTGGACTGC
TTCATCTCCCGGCCCACGGAGAAGACCGTCTTCACCCTCTTCATGGTGTGCTCGTCGGCT
ATCTGCATGGTGCTGAACGTGGCGGAGCTGTTCTACCTCATCTGCAAGGCGCTGCTACGC
TGCTCCCACCAGAGGCAGAAGCGCCAGCGCCGCGGTATCATGCTTGCTGATGCGACAGAG
GAAAAGGCCCTGTCTCAGAATGAGAAGAATGAGATGATACAGTCAGCCAATGCGAAAGCT
CTGTGA

>Aj-NN-cx30.3b-BEWY01000014
NNNNNCTGGGGTGCCCTGTACGCCCAGCTGGGCGGAGTCAACAAGCACTCCACCAGCCTG
GGGAAGATCTGGCTCTCGGTCCTCTTCATCTTCCGCATAATGATCCTGGTGCTGGCGGCG
GAGAGCGTGTGGGGCGATGAGCAGTCCGACTTCACCTGCAACACGCAGCAGCCCGGCTGC
AAGAACGTCTGCTACGACCGCTTCTTCCCCGTCTCGCACATCCGGCTCTGGTGCCTGCAG
CTCATCTTCGTCTCCACGCCGGCGCTGCTGGTGGCCATGCACGTGGCCTACCGCAAGCGC
GAGACCAAGCGGAGCATCATCCGCGCCCACGGCGACAAGGTGCAGGACGACCTGGAGAGC
CTGCGCAACCGGCGGCTGCCCATCACCGGCCCGCTCTGGTGGACCTACACCTCCAGCCTC
TTCTTCCGCCTGGTTTTCGAGGGCGGCTTCATGTACGTCTTCTACTTCATCTACGACGGC
TTCCAGATGCCCCGCCTGGTGAAGTGCGAGGAGTGGCCCTGCCCCAACGTGGTGGACTGC
TTCATATCGCGCCCCACCGAGAAGACCGTCTTCACCATCTTCATGGTGGCCTCCTCGGGC
ATCTGCATGGTGCTGAACGTGGCCGAGCTGGCCTACCTGATCGTCAAGGCGCTGCTGCGG
TGCTCCAGCGGCTCCGGGGGCAAGCACACCTTCCCCGGACAACGCCTCCAAGGACAAGGCC
TTCCTGCAGAACAAAAGGAACGAGATGCTGCTGTCCTCCTCGGACTCCTCCAGCAGCAAG
GCCGTGTGA

>Aj-NN-cx28.6-BEWY01000007 (3’-term; underlined)+ BEWY01000024 (5’-term) The latter is unplaced, but that these two parts belong together is indicated by AVPY01364480 and BDQN01002772 from *A. japonica*, AZBK01632506 from *A. anguilla*, and LTYT01005556 from *A. rostrata*

ATGAACTGGTCGGGCCTGGAGAGCCTGCTGAGCGGGGTGAATAAGTACTCCACGGTTTTC
GGGCGGGTGTGGCTGTCCATGGTGTTCGTGTTCCGCGTGCTGGTGTTCGTGGTGGCGGCC
CAGCGCGTCTGGGGGGACGAGAGCAAGGACTTCGTCTGCAACACCCGGCAGCCCGGCTGC
ACCAACGTGTGCTACGACAGCATCTTCCCCATCTCGCACATCCGCCTGTGGGCGCTGCAG
CTCATCTTCGTCACCTGCCCCTCGCTCATGGTGGTGGCGCACGTCAAGCACCGCGAGGAG
CTCGACCGAAAGTACGTGGCCTCGCACCCCGGAGCGCACCTGTACGCCAACCCGGGCAAG
AAGCGCGGCGGGCTGTGGTGGACCTACCTGCTGAGCCTGGTGTTCAAGGCCGCCTTCGAC
GCCGCCTTCCTCTACATCCTCTACTTCATCTACCACGGCTACGACATGCCGCGCCTGTCC
AAGTGCTCGCTGGATCCCTGCCCCAACACCGTGGACTGCTTCATCTCCCGGCCCACCGAG
AAGAAGATCTTCACGCTCTTCATGGTGGCCTCGTCCGCCATCTGTGTGTTCATGTGCCTG
TGCGAGATGGTCTACCTCGTGGGCAAGCGCTGCCACAAGGTCCTGCGCATACGGCGGGAG
AACGAGCAGCTGCTGTTCGCCGAGCGGCACGACATCACCGTCCTGGCCCGGCCCCGGTCC
GACTACAGCCGGCTGGACCCCACCGCCTCGGCCCCGCCCACCTGCGGCATCGCCGACGGC
AACACGCTCAAGGCCAAGAGGACCAAGAAGGCCGAGGAGGCCGAGAAGATGGAGGAGGCC
GCCACAGCCGCCGCCGCCGCGATCGGGAAGATGGCTTAG

>Aj-NN-cx28.6-BEWY01000004 1:25112498-25113309 **C,** inserted to keep reading frame, nucleotide chosen according to AVPY01029196 and BDQN01000086 (both *A. japonica*), and supported by LTYT01001691 (*A. rostrata*) and AZBK01815961 (*A. anguilla*).
ATGAACTGGTCAGCATTGGAAGTTCTCATTAGTGGAGTCAACAAGTACTCCACTGTGTTT
GGCCGCGTATGGCTCTCCATGGTGTTCGTGTTCCGCGTGCTGGTGTTCGTGGTGGCGGCG
CAGCGGGTGTGGGGTGACGAGAACAAGGACTTTGTTTGCAACACGGCGCAGCCGGGCTGC
GCCAACGTCTGCTACGACAGCGTCTTCCCCATCTCCCACATCCGTCTGTGGGCCCTGCAG
CTCATCTTCGTCACCTGCCCCTCCCTCATGGTGGTGGCGCACGTCAAATACAGAGAGGAG
AAAGACCAGAAGTACACCGCCCTTCACAAAGGCTCCCACCTGTACGCCAACCCGGGGAAG
AAGCGCGGTGGCCTGTGGTGGACCTACCTGATCAGCTTGGTCTTCAAAGCGGGCTTCGAT
GCCGCCTTCCTCTACATCCTCTACCACGTCTATGAGGGTTATGACATGCCCCGCCTCTCT
AAGTGTGCCCTGGAGCCCTGCCCCAACGTAGTGGACTGCTACATCTCCCGCCCCACAGAG
AAGAAGATATTCACCCTCTTCATGGTGGTATCATCGGCCGTCTGCATCCTCATGTGCATC
TGTGAGATGATATACCTGGTGGGCAAGCGCTTCATGAAGCTCATTACGGCCCGGAAGGAA
AGCGAACGGGCTCTTTTTGCCGAGCGGCACAATGTCACAGTCATGGCTCCACTCAACTCA
GAGTATGCCAAGCAGGATCCCACAGCCACTCCCTCCAACCAGAACAACAGCAACATTAAG
GCCGAGGAGGCATCCACCAGCAAGATAATGTAA

>Aj-NN-cx34.4-BEWY01000007
ATGAACTGGGCCTTCCTCCAGGGCCTCCTCAGCGGGGTCAACAAGTACTCGACGGCGTTC
GGGCGGGTGTGGCTGTCCATCGTCTTCCTGTTCCGGGTGATGGTGTTCGTGGTGGCGGCG
GAGAAGGTGTGGGGGGACGAGCAGAAGGACTTCAAGTGCAACACGGCGCAGCCGGGCTGC
CACAACGTCTGCTACGACCACTTCTTCCCCGTGTCCCACGTGCGGCTGTGGGCGCTGCAG
CTCATCTTCGTCACCTGCCCGTCGCTGCTGGTGGTGATGCACGTGACGTACCGTGAGGAG
CGCGAGAAGAAGAACCAGCAGAAGAACGGCGAGGGCTGCCGCCGGCTGTACAAGGACACG
GGCAAGAAGCGCGGCGGCCTGTGGTGGACCTACGTCCTCACGCTGGTCTTCAAGATGGTG
GTGGACGCCGTCTTCGTCTACCTCCTCTACTACATCTACGAGGGCTACGACTTCCCGTCG
CTGGTCAAGTGCACGCAGGTGCCCTGCCCCAACACGGTGGACTGCTTCATCTCCCGGCCC
ACGGAGAAGCGCATCTTCACGATCTTCATGGTGGTCACCAGCCTGGTCTGCATCCTGCTC
ACCTTCCTCGAGATCGTCTACCTGGTGGGCAAGCGCTGTCGGGAGTGCATCTCCTCCCTC
GGGCACTCCCGCCACATGGTGCCCGTCCACACTTCGCTGGTCAACAGCAAAGACGGCCTC
TCCGTGGTGGAGACCCGGTCCGTCAAGCTGAGTAACCACGACAACATCCGGGGCAAGGCT
CCAGCCTACAGCGAGGCCATGTCCTGA

>Aj-NN-cx35.4b-BEWY01000004
ATGGATTGGAAGATGTTTCAAAGCCTCCTCAGTGGGGTGAACAAATACTCCACGGGATTT
GGGAGGATCTGGCTGTCCGTGGTGTTTGTGTTCCGGGTGTTGGTGTTCGTGGTCGCTGCC
GAGCGCGTGTGGAGCGACGACCTGAAGGACTTTGACTGCAACACAAAGCAGCCCGGCTGC
CCCAACGCTTGCTACAACTACTACTTCCCCATCTCACACACCCGACTGTGGGCGCTGCAG
CTTATCTTCGTCACCTGTCCCTCCCTGCTGGTGGTGATGCACGTAGCCTACCGAGAAGAC
CGCGAACGTAAGTACCATTTGAAGCATCCCGAAGGAGCAAAGCTTTACGACAACACGGGC
CAGAAGCACGGAGGCTTGTGGTGGACGTACCTCCTCAGCCTCTTCTTCAAAACGGGCATC
GAGGTCACCTTCCTCTACCTGCTTCACCTCATCTACCACAACTTCTTCTTGCCCCGCCTT
GTCAAGTGTGACATCAGCCCCTGCCCCAATCACGTGGACTGCTACGTTGGACGCCCCACC
GAGAAGACAGTCTTCACCTACTTCATGGTGGGGGCCTCAGCCTTCTGCATCGTGCTGAAC
GTCTGCGAGATCGTCTACCTGATCGGCATGCGCATCCACCACCTGATTCACAAGGGCAGC
AAGAAATTTCCCCGTGACAGGTGCAAGGACGAAGACTGCAGCGAATGCAATGAGCCAGTC
ACCCACCTGGATTCCAAGCCGGGCTCCCGGCCAGGAGAGGAGATGCCAGCGTTGGCCCCA
AACCTCTCCTTCAAATGTTAG

>Aj-NN-cx35.4a-BEWY01000007
ATGGACTGGAAGATGTTCCAAGCGCTCCTTAGCGGGGTGAACAAGTATTCAACGGCGTTC

GGGCGGATCTGGCTGTCGGTGGTGTTCGTGTTCCGGGTGCTGGTGTACGTGGTCGCGGCA
GAGCGCGTCTGGGGGGACGAGCAGAAGGACTTTGACTGCAACACCAAGCAGCCGGGCTGC
CCCAACGTCTGCTACGACTACTTCTTCCCCATCTCCCACATCCGCCTGTGGGCGCTGCAG
CTCATCTTCGTCACCTGCCCCTCGCTCATGGTGGTGATGCACGTGGCGTACCGCGACGAA
CGGGAGCGCAAGTACCGCGCCAAGTTCGGCGAGGACACCAAGCTCTACGACAACACGGGC
AAGAAGCACGGCGGCCTGTGGTGGACCTACCTGCTGAGCCTCTTCTTCAAGACGGGCATC
GAGATCGTCTTCCTCTACCTGCTGCACATGATCTACGACAGCTTCTACCTTCCCCGGGTG
GTCAAGTGCGAAGTCAAGCCCTGCCCCAACCAGGTGGACTGCTACATCGGCCACCCCACG
GAGAAGAAGGTCTTCACCTACTTCATGGTGGGCGCCTCTGCCCTCTGCATCGTTCTGAGC
GTGTGCGAGATCATCTACCTGATCGCCAAGCGCATCGCGCGCGTCATCCGCAAGATGAAG
AGCCGCGACCGCCTGGTGGCGCTGCAGCACCAGCGGTACAAGGACGAGGACCGGGGAAGC
TACCAGCAGCTGCCCATGAAGCGCCTCGACCTCAAGCCCACCTTCAGGGTCAACGACGAG
ATGCGGGCGTCCGCCCCCAACCTGTCCACTGCCGCCTGA

>Aj-cx34.4-BEWY01000004
ATGAACTGGCTTTTCCTCCAGGGCCTCCTCAGTGGGGTGAACAAGTACTCCACAGCGTTC
GGCCGCATCTGGCTCTCAGTGGTCTTTGTCTTCAGGGTGTTGATTTTGGTGGTGGCTGCG
GAGAAGGTGTGGGGCGATGAACAGAAGGAATTCTCCTGCAACACGGCCCAGCCGGGTTGC
CACAACGTCTGCTATGACCATTTCTTCCCCGTGTCCCTCGCGCGACTGTGGGCCCTGCAG
CTCATCTTCGTCACCTGCCCCTCGCTGCTGGTGGTGTTGCACGTGGCCTACCGCAAGGAG
CGAGAGCGCAAACACTGCCTGAAGTACGGCGACGGGTGCCCTCGCCTCTACGCCGACGTG
GGGAAGAAGCGCGGAGGTCTGTGGTGGACATACTTCCTGAGCCTGCTCTTCAAGATGGGA
GTGGACACGGTCTTTGTCTACCTGGTCTACTACATCTATGAGGCTAATTTTTTCCCCCTG
CTAGTCAAGTGCAAAGAGGAGCCCTGCCCCAACGAGGTGGACTGCTTCATCACCCGGCCA
ACGGAGAAGCGCATCTTCGCCACTTTCATGGTGGTTATCAGCCTGATATGCATCCTCCTC
ACTCTCAGCGAGCTCCTTTACCTGGCCGGCAAGCGCTGCAGGGAGAGCTGCAGGCCCAGG
CGGCGCTCTGTTCAGCCGTCTGCGGCCTCTTCAGCGCTGGATAAGAATAAGACTTCCTTC
TTGGAGGTCCACCCTCTGAAGCAGCTTGATAATGGTAATAAAGACACCAGCGTTCCCGCG
TACAGCGTGGCGGTCTCCTGA

>Aj-NN-gjb7-BEWY01000019
ATGAACTGGGGGTTTCTGGAGAACATTTTGAGCGGGGTGAACAAGTACTCCACGGTGATC
GGTCGCATATGGCTCTCCATAGTTTTCATCTTCCGCATCCTGGTGTACGTTGCAGCCGCG
GAGCAGGTGTGGAAGGACGAGAACAAGGACTTTGTGTGCAACACCCAGCAGCCAGGCTGC
GAGAACGTGTGCTTTGACCACTTCTTCCCCATCTCCCAAGTGCGTCTGTGGGCCCTGCAG
CTCATCGTCGTGTCCACACCCTCCCTGCTGGTGGCGCTGCACGTGGCCTACCGCGAGCAC
AGGGAGAAACGGCACGGGAAGAAGCTCTACGAGAACAAGGGCAGCATAGACGGCGGTCTG
CTCTGCACCTACCTGCTCAGCCTCGCCTTCAAGACCAGTTTCGAGGTGGGGTCCCTGCTG
GCCTTCTACTTCCTCTACAGCGGCTTCGATGTCCCCCGGCTCCTGCAGTGCAGCCTGAGT
CCCTGCCCCAACACGGTGGACTGCTACATCTCCAAAGCCACGGAGAAGAAGGTCTTCCTC
TACATCATGGGGTGCACCTCTGTCCTGTGCATTGTGCTGAACCTCTGCGAGACGGCCTAC
ATCGTGTCCACGCAGTGCTGGAAGTGCTTCAGCAAGCGTTATGTCCCCATCGAGGAGAGG
GTGCACTGCCGCTGTCACCCTCCACCTGCCAGTGTTGCCACAGCAGTGCCACCAAACTTT
AAACCACCGGGCAAAGACATGGAAAACTCATGCCTTCCGCAAGCCACAAAGGACAGTCCA
ACAGCTGTCTCTTAA

>Aj-CXG1-BEWY01000014
ATGAGCTGGAGCTTTCTCACACGCCTCCTGGAGGAGATCTCCAACCACTCCACCTTCGTG
GGGAAGATCTGGCTGACGCTGCTCATCGTCTTCCGCATCGTGCTGACGGTGGTGGGCGGG
GAGTCCATCTACTATGACGAGCAGAGCAAGTTCGTCTGCAACACGCAGCAGCCCGGGTGC
GAGAACGTGTGCTACGACGCCTTCGCGCCGCTCTCGCACGTCCGCTTCTGGGTCTTCCAG
ATCATCATGATCACCACGCCCACCATCATGTACCTGGGCTTCGCCATGCACAAGATCGCC
CGCATGGAGGACGAGGACTACCGGCCCAAGGGCCGGAAGGGCAGGATGCCCATCATCAAC
CGCGGCGCCAACCGCGACTACGAGGAGGCGGAGGACAACGGCGAGGAGGACCCCATGATC
GTGGAGGAGATCGAGCCCGAGAAGGAGAAGAAGGCAGAGAAGCCGTCGGCCAAGCACGAC
GGGCGCCGGCGCATCAAGAAGGACGGCCTGATGAAGATCTACGTGGTGCAGCTGTTCTCC
CGCGTGGCCTTCGAGGTGGCCTTCCTCTTCGGCCAGTACGTCATGTACGGCTTCGAGGTG
GCGCCCTCGTACGTGTGCACGCGCATGCCCTGCCCGCACACGGTGGACTGCTTCGTCTCC
CGGCCCACGGAGAAGACCATCTTCCTCATCATCATGTACGTGGTGAGCGTGCTGTGCCTG
GCGCTGACGGTGCTGGAGATCCTGCACCTGGGCGTGAGCGGCATCCGCGACTCCTTCCGC
AGCCGCTCGGCGCGCCACCGCGCCCTGGCCGCCGCCCGGCCCTCGCTGTGCCGCCAGGCG
CCCACCGCGCCGCCCGGCTACCACACGGCGCTGAAGAAGGGCCCGGGGGGCAAGCCGTCG
AAGCCCGAGTTCCGGGACAACCTGGCCGACTCGGGCCGGGAGTCCTTCATGGACGAGGCG
TCGAGCCGCGACCTGGACCGCCTGCGGCGGCACCTGAAGATGGCGCAGCAGCACCTGGAC
CTGGCCTACCAGGCCGAGGAGGGGAACCCGTCCCGCAGCAGCAGCCCCGAGTCCAACGGC
ACCGCCGCCGAGCAGAACCGCCTCAACTTCGCCCAGGAGAAGCAGGGAGGGACGTGCGAC
AAAGGTGCGTGTTGGGGCCCGAGCCGTTTGGGGCCCGAGCCGCTTGGGACCCCGTGCTAA

>Aj-CXG1-BEWY01000001
ATGAGCTGGAGCTTCCTCACTCGTCTGCTGGAGGAGATCCACAACCACTCCACCTTCGTG
GGGAAACTGTGGCTGACCGTGCTGATCGTCTTCCGCATCGTTCTCACCGCCGTCGGAGGG
GAGTCCATTTACTACGACGAACAGAGCAAGTTCGTCTGCAACTCAGGCCAGCCGGGCTGT
GAGAACGTCTGCTACGATGCCTTCGCCCCCCTCTCGCACGTGCGCTTCTGGGTCTTCCAG
ATCATCCTGGTGGCCACGCCCTCGCTCATGTACCTCGGCTACGCCGTCAACAAGATCGCC
CGGCTGGAGGAGGGAAAGTCCGGCGGGGCCGGGGGCCTGTCCCACCGGAAGCCGCGGAAG
ATGTTCTTTGGTGGGCGACGGCAGCACCGGGGGATCGAAGAGGCCGAGGACGACCAGGAG
GAGGACCCGATGATCTACGAGGTGCCCGAGATGGAGAGCCGCAGCGAGGCCACGCCCCAG
GGGAAGCCCAAGGCCAGGCACGACGGGCGGCAGCGAATCAGGGAGGACGGGCTGATGCGC
ATCTACGTGCTGCAGCTGCTGACTCGCACGCTGCTGGAGGTGGGCTTCCTGGCAGGCCAG
TACGCCTTGTACGGGTTCGCCGTTCCGCCAGTCTTTGTGTGCTCAGGAAAGCCCTGCCCA
CACAGTGTCGACTGCTTTGTGTCACGGCCCACCGAGAAGACCATCTTCCTGCGCATCATG
TACGGGGTGACGGTCCTCTGCCTGGCGCTCAACATATGGGAGATGCTGCACCTGGGCGTG
GGCACCATCTGCGACATCCTGCGCACCCGCCGCGGTCCGCCAGAGGAAGACGTGTACCAC
CTGGGCTCCATGGGTCCCGGGGTCGCTAGCAGGGCTCCTGTGGTACCGGTTGGCGAATCC
GGGGAGGTGGGAGGGTACGGCAGTTACCCCTTCTCCTGGAACACCCCCTCCGCCCCGCCT
GGCTACAACATCGTGGTGAAGCCCGACCAGATCCAGTTCACAGACCTGAGCAACGCCAAG
ATAGCCTGCAAGCAGAACAAAGCCAACATAGCCCAGGAGGAGCAGCAGCAGTTTGGCAGC
AACGAGGACAACTTCCCCCTGGAGGAGGTGCGCGGGAGCCTGCAGAAAGAGATCCGGCAG
GCCCAGGACAGGCTGGAGGCCGCCATCCAGGCCTACAGCCACCAACACCAGAACAACAAC
CACAGCAACATCAGCCAGCCTCAGCGGGACCGCAAGCACCGCTCTGGCTCCAAGCACGGC
GCCAACAAGGCAGGCGGAGGCAGCAGCAGCAACAGCAGCAGCAAGTCCGTGGAGGGCAAG
CCTTCCGTGTGGATCTAA

>Aj-NN-cx45-BEWY01000018 C, inserted to keep reading frame. Nucleotide chosen according to BDQN01000333 (*A. japonica*) and LTYT010006439 (*A. rostrata*).
ATGAGCTGGAGCTTCCTCACCCGGCTCCTGGAGGAGATCCACAACCACTCCACCTTCGTG
GGGAAGCTGTGGCTGACGGTGCTCATCGTCTTCCGCATCGTGCTGACGGCGGTGGGCGGG
GAGTCCATCTACTACGACGAGCAGAGCAAGTTCGTCTGCAACTCGGGCCAGCCGGGCTGC
GAGAACGTGTGCTACGACGCCTTCGCCCCGCTCTCGCACGTCCGCTTCTGGGTCTTCCAG
ATCATCCTGGTGGCCATGCCCTCGCTCATCTACCTGGGCTACGCCATCAACAAGATCGCC
CGGCTGGAGGAGGGGGGCGGAGCCTGCCAGCCGGGGGCGGGGCC**C**CCGGGATTCACCCAC
AGGAGACCCCGCAAAATATTCTTCGGTGGGCGGGGCCAGGGGAGGGGCGTGTCCGAGGAG
GCGGAGGAGGACCAGGAGGACGACCCCATGATCTATGAGGTGCCCGAGATGGAGGGGCGG
ATGGAGATGGCCCCGCCCCGGCGGAGGACGAAGGCGCGCCACGACGGGCGGCGGCGTATC
CGTGCCGACGGGCTGATGCGGGTGTACGTGGCGCAGCTGCTGACGCGCACGGCCCTGGAG
GCGGGGTTCCTGGCCGGGCAGTACGCCCTGTACGGCCTGGCCGTGCCGTCCGTCTTCGTC
TGCTCCGACCCGCCCTGCCCGCACCGCGTGGACTGCTTCGTCTCGCGGCCCACGGAGAAG
ACCATCTTCCTGCGCATCATGTACAGCGTCACCCTGCTCTGCCTGGCGCTCGACCTGTGG
GAGATGCTGCACCTGGGGGCGGGCACCCTCTGTGACATCATACGCCGCCGTCGGGGCCCG
CCCCCCGAGGACGAGTACCAGCTGGGCNNNNNNNNNNNNNNNNNNNNNNNNNNNNNNNNN
NNNNNNAGAGCGGGGGGAGGCGTGCCGGGCGGGGACTACGGCAGCTACCCCTTCTCGTGG
AACGCCCCCCTGGCCCCGCCCGGCTACAACATCGCGGTGAAGCCCGAGCAGCCGCCGTAC
GCAGACCTGAGCAACGGCAAGATGGCCTGCCGGCAGAACCGGGCCAACATCGCCCAGGAG
GAGCAGCAGCAGTTCGGCAGCAACGAGGAGAACTTCCCCACGGGGGAGACGCGCGTCTCC
CTGCAGAAGGAGGTCCAGGAGGCGCAGGACCAGCTGGAGGCGGCGCTCCAGGCCTACAGC
CGGCAGCACGAGGACGGGGAGAAGCCTCAGAGCAACGTGGCGCTCCCGCACCGCGAGCGG
AGGCAGCGCGAGCGGAAGCAGCGCTCCGGATCCAAACACGGCAGCGCCAAGAGCAGGGAG
GACTCCAGCACCAGCAGCGTCAGCAGCAACAGCAAGTCCGCAGAGACCAAGCCCTCTGTG
TGGATCTGA

>Aj-gjc2-BEWY01000004
ATGAGCTGGAGTTTTCTCACCCGTCTTCTGGAAGAAATCCACAACCACTCCACGTTTGTG
GGGAAAGTGTGGCTGACTGTCCTGATCATCTTCCGCATTGTCCTGACGGCGGTTGGTGGG
GAGTCCATCTACTCGGACGAGCAGACCAAGTTCACCTGCAACACCAAGCAGCCCGGCTGC
GACAACGTGTGCTATGACGCCTTCGCTCCCCTTTCACATGTCCGCTTCTGGGTCTTCCAG
ATCATCATGATCTCCACCCCCTCCATCATGTACCTGGGCTACGCCATCCACAAGATCGCC
CGATCCTCGGAAGAGGAGCGCCGCAAGTACCGCAGGTCCCGCAAGAAGCCCCACGCCATC
AAGTGGAGGGCCACCCGCACCCTTGAGGAGGTCCTGGAGGAAGAGGAAGAGGAGGAGCCC
ATGATCTACGAGGACACCCTGGAAGTGCAGGAGATCAAGCCGGAGCCGGCCAAGCCCCCT
GGGCGGGACCAGCAGAAACACGACGGCCGGCGGAGGATCATGGAGGAGGGCCTGATGCGC
ATCTATGTGCTGCAGCTCCTGGCCCGCGCTGTCTTTGAGGTGGGCTTCCTGGCAGGCCAG
TACCTCCTGTACGGGTTCCATGTCTACCCGTCCTACGTCTGCAACAAGAGTCCCTGCCCT
CACAGCGTGGACTGCTTCATCTCCCGGCCCACGGAGAAGACCATCTTCCTCCTCATCATG
TACGTGGTGAGCTGCCTCTGCCTACTGCTGAACGTCTGTGAGATGTTCCACCTGGGCATC
GGGGCCTTCCGGGACATGCTCCGCAGGCGCCGGGGCAAAGGGCAGCGGCCCTCGTACAGC
TACCCCTACCACCGGAACATCCCCGCCTCCCCTCCAGGGTACAACCTGGTGGTGAAGTCG

GACAAGCCTGGCCCGATCCCCAACAGCCTCATAACTCACGAGCAGAACCTGGCCAACGTG
GCCCAGGAGCAGCAGTGCACCAGCCCCGACGAGAACATCCCCTCAGACCTGGCCAGCCTG
CACCACCACCTGCGTGTGGCGCAGGAGCAGCTGGACATGGCCTTCCAAACGTACAACAAT
AAGAGCAACCCGCAGCCCTCCCGAACAAGCAGCCCCGCCTCGGGGGGCACCATGGCTGAA
CAGAACCGGGTCAACACAGCCCAGGAAAAGCAAGGGGCCAGGCCGAAATCCAGCGCCGAG
AAACCTGGGACCGTAATAAAAAATGGCAAGACGTCCGTTTGGATTTAG

>Aj-gjd2-BEWY01000019 Splice site
ATGGGGGAATGGACCATCTTGGAGCGGCTCCTGGAGGCAGCTGTACAGCAACACTCCACT
ATGATAGGAAGGATCCTATTAACAGTGGTGGTGATCTTCCGGATTCTGATCGTAGCAATA
GTCGGAGAAACAGTCTATGACGACGAGCAAACCATGTTTGTGTGCAATACCTTACAACCG
GGCTGCAACCAGGCATGCTACGACAAAGCATTCCCAATTTCGCACATCAGATATTGGGTG
TTCCAAATCATTATGGTCTGTACTCCGAGTCTGTGTTTCATTACTTACTCTGTGCACCAG
ACTGCCAAACCAAAGGATCGCAAATACTCCACTGTCTATCTGTCGTTAGACAAGGACTCG
GATTCGAACAAACGTGATAACAGTAAAAAGATTAAAAACACAATTGTTAATGGAGTACTT
CAGAATACAGAAAACCCAACGAAAGCGGCCGAGCCAGACTGCTTAGAAGTAAAAGACATT
CCCACCTCGGCTATGAGACCTACCAAGTCGAAAATGAGGAGGCAAGAAGGCATCTCAAGA
TTCTACATTATCCAAGTCGTTTTCAGAAACGCGCTTGAGATTGGATTTCTCGTAGGTCAA
TATTTCCTTTACGGATTCAACGTCCCAGCTGTGTACGAGTGCGATCGATACCCGTGCATT
AAAGATGTAGAATGCTACGTCTCGAGACCAACGGAAAAGACTGTCTTCCTAGTCTTTATG
TTTGCCGTGAGTGGGATTTGCGTAGTCCTGAATTTAGCAGAACTCAATCACCTAGGATGG
AGGAAAATCAAAACTGCCGTGAGGGGTGTGCAAGCCAGGAGGAAGTCCATCTACGAAATC
AGGAATAAAGATTTGCCCCGAATGAGTATGCCAAATTTCGGCCGCACTCAGTCAAGTGAC
TCCGCTTATGTTTAG

>Aj-NN-gjd2-BEWY01000015
ATGGGAGAGTGGACCATTCTGGAGCGGTTGCTAGAGGCTGCTGTACAGCAACACTCTACC
ATGATCGGCCGGATTCTGCTGACAGTGGTGGTGATCTTCCGTATCCTGATTGTGGGCATT
GTGGGTGAGAAAGTATATGAGGATGAGCAGATCATGTTCATCTGCAATACTATGCAGCCT
GGCTGCAACCAGGCCTGCTATGACAAGGCCTTCCCCATCTCCCACATCCGCTATTGGGTC
TTCCAGATCATTCTGGTGTGCACACCCAGTCTGTGCTTCATCACCTACTCCGTGCATCAG
GCCGCCAAGCAGCGTGACCGCAGCTACTCCTTCCTGCACCCCTATATGGAGCGGGACCAC
GGCCGGCATGAGGGTGCCCGAAAACTGCGCAACATCAACGGCATCCTGGTGCACAACCCA
GAGAGTGGGGGCAAGGAGGAACATGACTGTCTGGAGGTGAAGGAGATACCCAATGCACCC
CGCGGCCTCACACACGGCAAGAGCGCCAAGGTGCGCCGCCAGGAGGGCATCTCCCGCTTC
TATGTCATCCAGGTTGTGTTCCGCAATGCACTGGAAATTGGTTTCCTGGCCGGGCAGTAT
TTCCTGTATGGTTTCAACGTGCCAGGCATGTTTGAGTGTGATCGCTACCCCTGTGTAAAG
GAGGTGGAGTGCTATGTGTCGCGGCCCACTGAGAAGACAGTGTTTCTCGTCTTCATGTTT
GCGGTCAGCGGGATCTGCGTGCTGCTCAACTTGGCTGAGCTTAACCACCTGGGTTGGCGC
AAGATTAAAACCGCCATCCGTGGTGTACAGGCACGACGCAAGTCTATCTGTGAGGTGCGC
AAAAAGGATGTCTCTCACCTGTCCCAGGCTCCCAACCTGGGTCGGACCCAGTCCAGTGAG
TCAGCTTACGTTTGA

>Aj-gjd2-BEWY01000156 Exon 1 lacking; Splice site.
NNGATCCTGCTGACGGTGGTGGTGATCTTCCGCATCCTGATCGTGGCCATCGTGGGCGAG
ACGGTGTACGAGGACGAGCAGACCATGTTCATCTGTAACACCATGCAGCCCGGCTGCAAC
CAGGCCTGCTACGACAAGGCCTTCCCCATCTCCCACATCCGCTACTGGGTCTTCCAGATC
ATCCTGGTGTGCACGCCCAGCCTGTGCTTCATCACCTACTCCGTGCACCAGTCCGCCAAG
CAGCGCGATCGCCGCTACTCCTTCCTGTACCCGCTGCTGGAGAAGGACTACGGGCGCGGC
GACGCCACCCGCAAGCTGCGCAACATCAACGGCATCCTGGTGCAGAGCCCCGACAGCGGG
GGCAAGGAGGAGCCCGAATGCCTGGAGGTGAAGGAGATCCCCAACGCGCCGCGCGGGCTC
ACGCACTCCAAGAGCTCCAAGGTGCGGCGCCAGGAGGGCATCTCCCGCTTCTACATCATC
CAGGTGGTCTTCCGCAACGCGCTGGAGATCGGCTTCCTGGCCGGCCAGTACTTCCTGTAC
GGCTTCAACGTGCCCAGCATCTTCGAGTGCGACCGCTACCCCTGCGTCAAGGAGGTGGAG
TGCTACGTGTCGCGGCCCACCGAGAAGACCGTGTTCCTGGTCTTCATGTTCGCGGTCAGC
GGGATCTGCGTGGTGCTCAACCTGGCCGAGCTCAACCACCTCGGCTGGCGCAAGATCAAG
ACCGCCATCCGGGGGGTGCAGGCGCGGCGGAAATCCATCTGCGAGGTGCGCAAGAAGGAC
ATCTCGCACCTGTCGCACGCCCCCAACCTGGGCAGGACCCAGTCCAGCGAATCCGCCTAC
GTTTGA

>Aj-gjd2-BEWY01000007 8885469-8886309 Exon 1 is lacking; Splice site.
NNGATCCTATTAACAGTGGTGGTGATCTTCCGGATTCTAATCGTAGCGATAGTTGGAGAG
ACAGTCTATGATGACGAGCAAACCATGTTCGTGTGCAATACATTACAGCCTGGTTGCAAC
CAGGCATGCTACGACAAGGCATTCCCGATCTCCCACATCCGATACTGGGTGTTTCAGATC
ATTATGGTTTGCACCCCAAGCCTCTGTTTTATTACTTACTCCGTCCACCAGTCTGCAAAA
AAGGAGCGCAAATATTCCACCGTGTTTCTAACGTTGGATAAGGACCAGGATCCATTGAAG
CGCGATGACAGCAAAAAGATTAAAAACACAATTGTAAATGGAGTACTTCAAAACACAGAA
AACTCAACCAAAGAAGGCGAGCCTGACTGTTTGGAAGTCAAAGAGATACCAAATTCGGCC

ATGAGAACTACTAAGTCAAAAATGAGGCGCCAGGAAGGCATCTCCAGATTTTACATCATC
CAAGTGGTTTTCAGAAACGCCCTGGAGATTGGGTTTTTAGTGGGTCAATATTTCCTTTAC
GGATTCAACGTGCCCTCCGTGTACGAGTGTGATCGATACCCCTGCATAAAAGATGTCGAG
TGCTATGTTTCCAGACCAACGGAGAAGACCGTGTTTTTGGTCTTCATGTTTGCGGTCAGT
GGGTTTTGCGTGGTGCTGAATTTAGCGGAACTCAATCATTTGGGATGGAGGAAAATCAAA
ACGGCGGTAAGAGGCGTGCAGGCGAGGAGGAAGTCCATTTATGAAATTAGGAATAAAGAC
TTGCCCAGAATGAGTGTTCCGAATTTCGGACGCACTCAGTCAAGTGACTCCGCGTATGTG
TAG

>Aj-NN-cx39.2-BEWY01000015
ATGGGGGACTGGTCCATTCTTGGCCGTTTCCTAACGGAGGTGCAGAACCACTCCACGGTG
ATTGGCAAGATCTGGCTGACCATGCTGCTGATCTTCCGCATCCTCCTGGTGACGCTGGTG
GGCGACGCGGTCTACAGCGACGAGCAGTCCAAGTTCACCTGCAACACGCTGCAGCCCGGC
TGCAACAACGTCTGCTACGACACCTTCGCCCCCGTCTCGCACCTGCGCTTCTGGGTCTTC
CAGATCGTGCTGGTCTCCACGCCGTCCATCTTCTACATCGTCTACGTGCTGCACAAGATC
GCCAAGGACGAGAAGCTGGAGATGGAGAAGGTGCAGGTGCAGGTGCTGGCCAAGCGGGGA
CCCCCGCGGGTGCGGGCGGGGCCATCGAACCGGGGCGGGGAGAGGGAGGAAACTCTGGAG
GCAGCCACGCCCGCCTTCAGCCCCCGTTTTGAGGAGGAGTGGAGCCCCCAGGAGGGGGAA
TGCGTGGAGCAGAGCCTCCTGGAGGAGGAGCTCGGGGAGGTGGGAAAGGACCCCACCCAG
CTGTCCAGCCAGGTGCTGCTCATCTACATCGTCCACGTGGTGCTGCGCTCCATCATGGAG
ATCGCCTTCCTGGTGGGGCAGTACTACCTGTTCGGCTTCGAGGTCCCCCACCTCTTCCGC
TGTGAGACCTACCCCTGCCCCAACCGGACCGACTGCTTCGTGTCCCGGGCCACCGAGAAG
ACCATCTTCCTCAACTTCATGTTCAGCATCAGCCTGGGCTGCTTCATCCTCAACATCGTT
GAGCTGCACTACCTGGGCTGGGTCTACATCTTCCGCATACTCTGCTCCGCCTGCTCCACC
TGCTGCGAGCAGGACCGGGACCCGGCCGAGCGCGTGGGGCTCTACAACGTCCACAACCCC
CTCCTGCTGCAGCTCAAGCACTCGCTGCGGGGACGGGTGGTCCTGCAGACCCCCCCGCCC
CTGTCCCAGGAGAAGACCGGCGGCCTGCCCACGCACGCGCCCGCCATCTCCTTCGAGACG
GACTCCACCGTGGAGTGCACGTCCAAGAGGAGCCCCGACGACAAGGAGCGCGCCAAGGCC
AAGCTGGCAAACGTGGCCAAACTGGGGCGCGGCAAGAAATCCTGGCTGTGA

>Aj-NN-cx36.7-BEWY01000002
ATGACCGAGTGGACCCTGCTGAAGCGCCTCCTGGACGCCGTGCACCAGCACTCCACCATG
ATCGGTCGGATCTGGCTCACCGTCATGGTGATCTTCCGGCTCCTCATCGTCGCCGTGGCC
ACCGAGGACGTCTACACGGACGAGCAGGAGATGTTCGTCTGCAACACCATCCAGCCGGGT
TGCTCCAACATCTGCTACGACTCCTTCGCGCCCATCTCCCAGCCCAGGTTCTGGGTGTTC
CAGATCATCACCGTGTCCACGCCGTCCTTGTGCTTCATCATCTACACCTGGCACAACCTC
TCAAAACATCCGGAGGGGGAGCACGTTAAAGAGAGCCGAGAGACGTACGACAGGAGTTGC
GACTCCGACAGCTGCTCCATCAAATCCCACAGACACCTGGGGCACAGCCTGGCAGACGTG
CTGGAGGGCATCGCTGCTCAGAGCAATCACAAAGCGGCCAGCGGCAGCTCTGTCGCAAGA
TCGCGAGTCTTCCAGGAAAGCGGGAAGTCGGGAGTCCTGTCGAAATACTACGTCTTCCAC
GTGTGCTTCCGGGCGACCCTGGAGATCGGCTTCGTCTTCGCCCAGTGGCTCCTGTTCGGG
TTCCACGTCCCCTCTCACTTCGTGTGCACCGCATTCCCCTGCTCCCAGAGGGTGGACTGC
TACGTCTCCCGGCCTACGGAGAAGACCGTCTTCCTCGTCTTCATGTTCTGCGTTGGGATA
TTCTGCATCTTCCTGAACTTCCTGGAGCTGAACCACTTGGGCTGGAAAAAGATCAAGACG
TCTATCCGCATAAGAGAGAGCCCGTGGAGAGGCTACGAGGCCATAAACCAGGACAGCCAA
TCGGTGGCCTCCCTTACATTCAGGGACATTACTAGCACCACGTCTCTGCCCACGTTAGAC
CTGGTGGTGGAACACAAGCCAGACTGGACCTGCACGGGAAACTGCTCCCCGCTCAAAGAC
GAAACGGCCGCAGAAGCGCAAAGCCAGGACAACCCCAGAGGAACGCAGTCTGTAAAGAGC
AAGACCAGCAAAGGGAGGTCCTCCAAGCAGAGGAGCTCCGAGGTCTGGATATAA

>Aj-CXD3-BEWY01000001
ATGGGGGAATGGGGCTTCCTCAGCGGGCTCTTCGACGCCCTGCAGGCCCACTCGCCCATG
CTGGGCCGCTTCTGGCTGCTGCTCATGCTGGTCTTCCGCATGCTGATCCTGGGCACGGTG
GCCACGGACCTGTTCGAGGACGAGCAGGAGGAGTTCGCCTGCAACACCCTGCAGCCGGGC
TGCAAGCAGGTCTGCTACGACCAGGCCTTCCCCATCTCCCAGTACCGCTTCTGGGTCTTC
CACATCGTGCTCATCTCCACCCCTGCGCTGGTCTTCCTCATGTACGCCATGCACCACAAC
AACAAGAAGGCGGCGGGACGCTGCCTCGACTCCGCCTCCTGCGCCCGGGAGGGCCTCCGC
CTGCGCCGCCTGTACATGGTCAACGTGGGCTTCCGGCTGCTGGCGGAGGTGGGCTTCGTG
GTGGGCCAGTGGTGGCTCTATGGCTTCCGGGTGGAGGCCCAGTTCCCCTGCAGCCGCTTC
CCCTGCCCCTACACGGTGGACTGCTTCACCTCCCGGCCCATGGAGAAGACGGTCTTCCTC
TGCTTCTACTTCGCCGTGGGCGTGCTGTCGGCCCTGGCCAGCCTGGCCGAGCTGCTGCAC
GTCACCTACAAGTGGTTCTCCGCGTCCTCCAGGGAGGCGCGCTACGGCAGCCAGAACCTG
CGCAACCTGGCCCAGGAGGAGGCCTCGTCCCTGCGGGGGCCCGGGGCCCCCCCGGCCAGG
CGAGGCCGGGGGGGCAGCGGCAGGCACAAACGCGGCTCCCTGCTCAGCACCGGCAGCAGC
AGCAAGGTTTCCAGCATCGGCCGCAAGTCCTCCAAATCCAAGAGCCTCAAGACCTCCATA
GCTGTATGA

>Aj-gjd4-BEWY01000005 1:52818799-52819950 Exon 1 lacking; Splice site.
NGGAAGATCTGGCTGGTGCTGATGGTCCTGCTGAGGGTCCTGGTGCTCCTGCTGGCGGGG
TACCCCCTCTACCAGGACGAGCAGGAGCGCTTCGTCTGCAACACCATCCAGCCGGGCTGC
GCCAACGTGTGCTACGACGTCTTCTCCCCGCTCTCCCTGTTCAGGTTCTGGCTGGTGCAG
CTCACCACGCTCTGCCTGCCCTACCTGGTGTTCGTGGTCCACGTCGTCCACAAAGTGTCC
CGGGGCCTCGCCGCGGAGGGCCGCGCCCCCGGCAGGGCCAAAGCCGCGCCCCCGTACAAG
GCCCGGCAGGAGCCTGGCGGGAAGGCCGCCTCTCCGAGCGGGGCGGAGAGGGGCGGGGCC
CGCAGCTTCACGGCCGCCTACGTGGTCCACCTGCTGCTGCGCATGGTGCTGGAGGCCGGT
TTCGGCGTGGCGCACTACTACCTGTTCGGCTTCCACATCCCCAAGCAGTTCCTGTGCCAG
CAGGCGCCCTGCACCACCACCGTGGACTGCTACATCTCCCGGCCCACGGAGAAGACGGTC
ATGCTCAACTTCATGCTGGCGGTGAGCGCCCTGTCCTTCCTGCTCAACTTCGCCGACCTC
GTCTGCGCCATCGAGTGGTCGGTCAAGCAGAGGAGCGGGAGCAAGACGGTGGTGGAGAAG
GCGTACGAGGAGGAGCAGTACTACCTCTCACCCCCGAGCGGGCGGAGCGTGGGGGCGGAG
CTCCCGCTCCCGCTCGCCCGCGACCTCGTGACCTCCGCCGCCTTCCGCAAGAGGGCGGCC
AGCAGTCCAGCACCGACGAGGCGGNNNNNGCGGGACAAAGCCGCGGCCCCACCCACCCCG
GAGCTGTGCGGTCGGAGGGTGGGCCAGTACACTCTGGTGGAGCTGGCCTCAGAGCTGCAG
TCCAACAGCAGCGACATGCAGGAGAAGAGGTCTGAGTGGGTGTGA

>Aj-NN-gje1-BEWY01000019 Splice sites
ATGTCTTTAAATTATATCAAGAACTTTTATGAAGGATGTCTCCGGCCTCCAACTGTGATT
GGCCAGTTCCACACTCTGTTCTTCGGCTCCGTGCGCATGTTCTTTCTTGGGGTCCTGGGC
TTTGCTGTTTATGGCAATGAGGCTCTACATTTCAGCTGCGACCCCGATAGGCGGGAGCTA
AACCTCTACTGTTATAACCAATTCAGGCCAATTACACCTCAGGTATTCTGGGCATTGCAG
CTAGTGACTGTTTTGGTACCTGGAGCTGTCTTTCACCTGTATGCAGCCTGTAAAAATATT
GACCAGGAAGAAATCCTCCAACGGCCCAAATACACTGTCTTTTACATTATCTCTGTCCTG
TTAAGAATCATTCTTGAGATCATAGCATTTTGGCTGCAGAGTCATCTTTTCGGGTTCCAA
GTGCACCCGCTTTACATGTGTGACGCTAGCGCCCTGGAGAAAATGTTCAACGTTACCAAG
TGCATGGTGCCTGAACACTTTGAAAAGACCATCTTCCTCAGTGCAATGTACACCTTTACT
GTAATCACAGTGGTGCTGTGCGTAGCTGAGATTTTTGAGATACTCTGTAGAAGATTGGGC
TATTTGACCAGTCAATGA

Suppl. Fig. 12. *Connexin39.2* (“*gjd2like*”) from mammals.

Note that some entries present in GenBank have wrong subfamily designation. In humans and koala the sequence is said to belong to the alpha subfamily, in Egyptian rousette it is said to belong to the gamma subfamily, while in black flying fox it is said to belong to the delta subfamily (which is correct). The corresponding opossum sequence (Md-*GJD2like-39.2*-XM_001376506), which was the first *cx39.2* sequence found in mammals (Cruciani and Mikalsen, 2005), is depicted in Suppl. Fig. 3. Several of these sequences are (supposed) pseudogenes (indicated by *GJA4**P***, *cx39.2**P***). To show the exact alignments of these sequences used in the phylogenetic analyses, which showed that they belonged to a single orthologous group, we have indicated the gaps (-). The gaps have been adjusted to fit the codon borders as much as possible. Be aware that most alignment tools remove gaps before performing alignment.
The previously non-predicted sequences (indicated by “NP”) were found by blasting other *cx39.2* sequences into Ensembl genomes or GenBank wgs (using Placentalia, marsupials, bats, or Afrotheria as species groups).

```
>Hs-GJA4P-NG_026166
ATGAGCGACTGGTCATTCCTGGGCTGGCTCCTGACCCGAGTGCAGAACGATTCCACCGTG
GTTGGCAAGGTATGGCTCACTG---TCCTGGTCTTACACATCCTGCTTGTCGCCCTGCTG
GGAAGTGCTGTCTGT-GGGATGAGCACTGCAAGTTCATCTGCAATACCCTGCGGCCTGGC
TGCACCAA------------------TGACCACTTCTCCCACTTCCGCT--GGGGCTTTC
---CAGATT---GTGCTGGTGGCCGTACCCTCCATCTTCTTTGTTGTCTGTGTGCTGCAC
TAGATGGTGAATGGGAGACAGTGGATGTGGAGAGGGGGTACCTGCTGGAAACCGTGCAAG
AGCTGGCAGCTGGAGGGGCTCTCCCTGGACCCAGGCTGGGGCCCCTTGGGGCTTCTTTCT
TTCTAGAGGGGCAGCTCTTAGTAGGAGAGGAGGTTTTTCCCCAAATGCCTTGGGGCTGCC
ACCTGGTACCCCAGCCTGCAGTCATACAGGGTCCTGGCTGTCTGCACTGCCCACGTGGTG
CTGCGGGCCTGCATGGAGCTGGCCTTCCTGGTGGGGT----CTA-CTCTCTGGGTGTGAT
ATGCCATGGTTGCTTCACTGCCACTCCTCCCCTGTCCCTCC---AGTCCTGACTGCTTTG
TGTCCAGAGCCATGAGGAAGAAAATCTTCCTGAACTTCATGTGCAG-GTGGGGTTGGGCT
GCTTCCTCCTGAACCCGATGGAGTTGTGCTACCTGGGCTGGGTCTTCCCTTGCCAGGCAC
GCTCTGTGGCCTGCACCAGCTAGTGCTACTTCTGCTCCACTGTGATGAGGAAGGACCGTG
CTCCAGGTGCCCTCC

>Wallaby-NP-cx39.2 Notamacropus eugenii.
ATGGGCGACTGGTCATTCCTAGGCCGGCTTCTTACTGAAGTCCAGAACCACTCCACCGTC
ATTGGCAAGATCTGGCTCACCGCACTCCTCATCTTCCGAATACTCCTGGTCACGCTGGTG
GGGGATGCAGTCTACAGGGACGAACAGTCCAAGTTCACCTGTAACACCCTCCAGCCAGGC
TGCACCAACGTCTGCTACAACAGCTTCGCCCCCTTTTCCCACCTCCGCTTCTGGATTTTC
---CAAATC---GTTCTGGTGGCCACACCTTCCATCTTCTACATCGTATGCTTGATGCAC
CAGGTGGCCCTGGAGGAGCGGATGGATGTGGAGAGGGACCGCCTGCTGGAGCTGTGGCAA
AGACAGGCAGCCGCTTATCAAGTCTATCCAAGATCGGGCTCTGGGGTCCTCTTGCCCTCT
GGCTCCTTGGAGGGCCAGAGCCTGGAGGAGGAAAAGGTTCTCCCAAAGCATGTCGGGTCC
ACAGCTCAGGACCCCATCCAGCTGGCCAACCGGGTGTTGATCATTTACATTGCGCACGTA
GTGCTGAGGTCCTTCCTGGAGCTGGGGTTCCTAGTGGGGCAATATTACCTGTTTGGCTTT
GATGTGCCCCATTTATATCGCTGCGAAACCTACCCGTGTCCCACA---AAGACAGACTGC
TTTGTCTCCAGGGCTACAGAAAAAATGATCTTTCTGAATTTTATGTTTGGGGTGGGGCTT
GGCTGTTTTCTTCTGAGCTTGGCAGAGCTGCATTATCTGGGCTGGCTCTTCACCTTCCGG
ATGCTCTTCAAGGCTTGTGTCAATTGCTGCCAATATCTGAGGAAGGCTTCCCCACCTTAC
AAGCCCCGGCTCCTGCCTCTTCTGGACTCGAGTCAGGAAAGGATGCTTCTGGAGGTCTCC
TTGCTGCCTGTATGGGGGTCAGGGCATCCCCGGCCACAGCATACTGTGA

>Koala-gja4like-XM_020963328 Phascolarctos cinereus
ATGGGCGACTGGTCATTCCTGGGGCGGCTTCTCACTGAAGTCCAGAACCACTCCACTGTC
ATCGGCAAGATCTGGCTCACCGCCCTCCTCATCTTCCGCATCCTCCTGGTCACTCTGGTG
GGCAATGCAGTCTACGGGGATGAACAGTCCAAGTTCACCTGTAACACCCTCCAGCCCGGC
TGCACCAACGTCTGCTACAACAGCTTCGCCCCCATATCTCACCTCCGCTTCTGGATTTTC
---CAGATT---GTCCTGGTGGCCACGCCCTCCATCTTCTACATCGTCTGCGTGATGCAC
CAGGTGGCCCTGGAGGAGTGGATAGATGTGGAGAGGGACCGCCTGCTGGAGCTGTGGCAA
AAGCAGGCAACTTCTCACCAGGCCTTTCCGCAGTCAGACTCTGGGGTCCTGGTGCCCTCC
AGCTCCTTCGAGAGCCAGAGCCTGGAGGAGGCCGAGGAGATCCTTCCAAAGCATGCCGGC
GCCACAGCTCAGGACCCCATCCAGCTGGCCAACCGGGTGTTGGTCATTTATATTGCCCAC
GTGGTGCTGAGGTCCTTCCTGGAGCTGGGATTCCTAGTGGGGCAATATTAACTGTTTGGG
TTTAACGTGCCCCATTTATACCGCTGCGAAACCTACCCGTGTCCCACC---AAGACAGAC
TGCTTTGTCTCCAGGGCAACAGAGAAAATGATCTTCCTGAATTTTATGTTCGGGGTGGGG
```

CTTGGCTGTTTTCTTCTGAACTTGGCAGAGCTGCATTACCTGGGCTGGCTCTTCACCTTC
CGGACGCTCTTCAAGGCTTGTGTCAATTGCTGCCAATATCTGGGGAAGGCTTCCCCACCC
CACAGGCCCCAACTTCTGCCCCTTCTGGACTCTAGTCAGGATGGGATGCTCCTGGAGGCC
TCCTTGCTGCCTGCATGGGGGTCAGGGCATCCCCGGCCACAGCATACAGTGA

>Pv-NP-cx39.2 *Pteropus vampyrus* Latge flying fox
ATGAGTGACTGGTCGTTCCTGGGCAGGCTCCTGACACAAGTGCAAAACCATTCCACCGTG
GTTGGCAAGGTGTGGCTCACCGTCCTTCTGGTCTTCCGCATTCTGCTGGTCACCCTGGTG
GGAGATGCAGTCTATGGGGATGAGCAGTCCAAGTTCACCTGCAATACTCTGCAGCCTGGC
TGCACAAATGTCTGCTATGACCGCTTCTCACCTGTCTCCCACTTCCGCTTCTGGGTTTTT
---CAGATT---GTGCTGGTAGCCACTCCCTCTATCTTCTATGTCATCTATGTCCTGCAT
CAGATAGCAAGGGAGGAAAGAGTAGATATGGAGAGGGAGTACCTGCTGGATACATTGCGA
AAGCTAGCATCTGGGGGAGCCCTGCAGAGACCCAAGCTGGGGCTCTTGGCGTCTTCTCAC
TTCCTAGAGGGACAGCTGCTGGTGGGAGAGAGGGTTCTCCCCAAATGCCTTGGGGCTGCA
GCCAGGAATCCAGGCCTGAGGTCACAAAGAGTCTTGGCCATCTACATTGCCCATGTGGTG
CTGCGGGCCTTCATGGAGCTGGCTTTCCTGGTGGGGCAATACTATCTGTTTGGGTTTGAT
GTTCCATACTTGTTTCACTGCCACTCCTATCCCTGTCCTACT---AGTACTGACTGCTTT
GTATCCAGGGCCACAGAGAAGATGATTTTCCTGAACTTCATGTTTGGGGTCGGGGTGGGC
TGCTTCCTCCTGAACCTGGTGGAGTTGCACTACCTGGGCTGGGTCTTTACCTACCGGTTC
CTCTTTGCAGCCTGCACCAGTTGCTGCCACTTCTGTGGGCAGCCTGCCCATCCTCCACTG
CTCTACTCTGATGAGGACAGTACTGGGCTCCAGGTGTATTCCTGCCTTTAG

>Pa-GJD2like-XM_006925175 *Pteropus alecto* Black flying fox
ATGAGTGACTGGTCGTTCCTGGGCAGGCTCCTGACACAAGTGCAAAACCATTCCACCGTG
GTTGGCAAGGTGTGGCTCACCGTCCTTCTGGTCTTCCGCATTCTGCTGGTCACCCTGGTG
GGAGATGCAGTCTATGGGGATGAGCAGTCCAAGTTCACCTGCAATACTCTGCAGCCTGGC
TGCACAAATGTCTGCTATGACCGCTTCTCACCTGTCTCCCACTTCCGCTTCTGGGTTTTT
---CAGATT---GTGCTGGTAGCCACTCCCTCTATCTTCTATGTCATCTATGTCCTGCAT
CAGATAGCAAGGGAGGAAAGAGTAGATATGGAGAGGGAGTACCTGCTGGATACATTGCGA
AAGCTAGCATCTGGGGGAGCCCTGCAGAGACCCAAGCTGGGGCTCTTGGCGTCTTCTCAC
TTCCTAGAGGGACAGCTGCTGGTGGGAGAGAGGGTTCTCCCCAAATGCCTTGGTGCTGCA
GCCAGGAATCCAGGCCTGCGGTCACAAAGAGTCTTGGCCATCTACATTGCCCATGTGGTG
CTGCGGGCCTTCATGGAACTGGCTTTCCTGGTGGGGCAATACTATCTGTTTGGGTTTGAT
GTTCCATACTTGTTTCACTGCCACTCCTATCCCTGTCCTACT---AGTACTGACTGCTTT
GTATCCAGGGCCACAGAGAAGATGATTTTCCTGAACTTCATGTTTGGGGTCGGGGTGGGC
TGCTTCCTCCTGAACCTGGTGGAGTTGCACTACCTGGGCTGGGTCTTCACCTACCGGTTC
CTCTTTGCAGCCTGCACCAGTTGCTGCCACTTCTGTGGGCAGCCTGCCCATCCTCCACTG
CTCTACTCTGATGAGGACAGTACTGGGCTCCAGGTGTATTCCTGCCTTTACCTCACTAGT
TGCCACGCCTACACCGTCAAGGGCTTCCTCCCACCCTTTGCTTGTTCATCCTTCTGGGGA
GCTCTGCCCTGGCAGGCTTCAGAGCCCAACTAA

>Ra-GJC2like-XM_016138748 *Rousettus aegypticus* Egyptian rousette
ATGAGTGACTGGTCGTTCCTGGGCAGGCTCCTGACGCAAGTGCAAAACCATTCCACCGTG
GTTGGCAAGGTGTGGCTCACCGTCCTCCTGGTCTTCCGCATTCTGCTGGTCACCATGGTG
GGAGATGCAGTCTATGGGGATGAGCAGTCCAAGTTCACCTGCAATACGCTGCAGCCTGGC
TGCACAAATGTCTGCTATGACCGCTTCTCACCTGTCTCCCACTTCCGCTTCTGGGTTTTT
---CAGATT---GTGCTGGTAGCCACTCCCTCTATCTTCTATGTCATCTATGTCCTGCAT
CAGATAGCAAGGGAGGAAAGAGTAGATATGGAGAGGGAGTGCCTGCTGGATACATTGCAA
AAGCTAGCATCTGGGGGAGCCCTGCAGAGACCCAGGCTGGGGCTCTTGGGGTCTTCTCAC
TTCCAAGAGGGACAGCTGCTGGTGGGAGAGAGGGTTCTTCCCAGATGCCTTGGAGCTGCA
GCCAGGAATCCAGGCCTGCGGTCACAAAGAGTCTTGGCCATCTACATTGCCCATGTGGTG
CTGCGGGCCTTCATGGAGCTGGCCTTCCTGGTGGGGCAATACTATCTGTTTGGGTTTGAT
GTTCCATACTTGTTTCACTGCCACTCTTATCCCTGTCCTACT---AGTACTGACTGCTTT
GTATCCAGGGCCACAGAGAAGATGATTTTCCTGAACTTCATGTTTGGGGTCGGGGTGGGC
TGCTTCCTCCTGAACCTGGCAGAGTTGCACTACCTGGGCTGGGTCTTCACCTGCCGGTTC
CTCTTTGCAGCCTGCACCAGTTGCTACCACTTCTGTGGGCAGCCTGCCCATCCTCCACTG
CTCTACTCTGATGAGGACAGTACTGGGCTCCAGGTGTCTTCGTGCCTTTAA

>Tm-NP-cx39.2P *Trichechus manatus* Manatee
ATGAGCGACTGGTCATTCCTGGGCCGGCTCCTGACCCAAGTG-AGAACCATTCCGCCATG
GTCAGCAAGTTGTGGCTCACCATCCTCCTGGTCTTCCGCATCCTGCTGGTCACCCTGGTG
AGAGGCGCTGTCTATAGGGATGAGCAGTCCAAGTTCACCTGCAACACTCTGCAGCCTGGC
TGCACCAACGTCTGCTACGACCACTTCTCGCTTGTCTCGCACTTCCGCTTCTGGGTCTTG
---CAGAAGG---TGCTGGTGGCCACACCCTCCATCTTCTATGTCGTCTGTGTCCTGCAC
CAGATCGCGGCTGGGGGAGCCCTGCAGGGACCCAGTCTGGTGTTTCTGGGGTCCTCTCAT
TTCCTAGAGGGACAGCTCCTGGTACGAGAGGGCGATTCTCCCCAAATGCCTTGGGGCTGC
AGCCCAGGACCCCAGCCTTTGGTCTCACAGCGTCTTGGTCATCTACATTGCCCACATGCT

GCTGCTGGCCTTTATGGAGCTGGCCTTCCTGGTGGGGCCATACTATCTGTTTGGGTTTGA
TGTCCCATACTTATTTCACTGTCACTCCTACCCC-GTCCTACC---AGTATTAACTGCTT
TGTTTCCAACGCCACAGAG---ATGATCTTCCTGAACTTCATGTTTGG-AGTGGGGCAGG
CTGCTTCCCTTTGAACCTGGTGGAGCTGCACTAGCTGGGCTGAGTCTTCACCTGACAGAC
CCTCTTTGTGGCCTGTGCCAGCTGCTGCCA

>La-NP-cx39.2P African elephant *Loxodonta africana*
ATGAGCGACTGGTCATTTGTGGGCCAGCTCCTGACCCAAATG-AGAACCATCCCACCATG
GTCAGCAAGGTGTGGCTCACCGTCCTCCTGATCTTCCACATCCTGCTGGTCACTCTGGTG
AGAGACACTGTCCATAGGGACGAGCAGTCTAACTTCACCTGCAACACCCTGCAGCCTGGC
TGCACCAACATCTGCTGTGACCGCTTCTCGCCCGTCTCCCACTTCTGCTTCTGGGTCTTT
---CAGATC--CCTGCTGTTGGCCATGCCCTCCATCTTCTCTGTCATCTGTGTCCTGCAC
CAGATCACAAGGGAGGAGAGAGTCAGTGTGGAGAAAGGGTACCTGCTGGAGACCTTGCAG
AAGCTGGTGGCAGGGGAGCCCTGCAGGGACCAGGCCAGTGTTCCCTGGGTCCTCTCATTT
CCTAGAGGGACAGCTCCTAGTGGGAGAGGGAGATTCTCCCCAAATGCCTTGGGGCTGCAC
CTCAGGACCCCAGCCTTTGGTCTTGCAGGGTCTTGGTCATCTACATTGCCCATGTGGTGC
GGCAGGCCTCTATGGAGCTAGCCTCCCTGTTGGGGCAATACTATCTGTTTGGGTTTGATG
TCCCATAGTTATTTCACTGTCGCTCCTACCCCTGGCCCACC---AGTATTGACTGCTTTT
TGTCCAGGGCCACAGAG---ATGACCTTCCTGAACTTCACATTTGG-GGTGAGGTAGGCT
GATTCCTCCTGAACCTGGTAAAGCTGCGCTACCTGGGCCGAGCCTTCACCTGCCAGGCCC
TCTTTGCAGCCTGTACCAGCTGCTGCCA

>Afer-NP-cx39.2P Aardvark *Orycteropus afer.* Note that the first Cys codon in the first conserved dokmain is mutated.
-----------GTCATTCCTGGGTTGGCTCCTGACCCAACTGCAGAACCATTCCACCATG
GTCAGCAAGGTGTGGCTCACTATCCTCCTGGTCTTCTGCGTCCTTCTGGTCACTCTGGTG
GGAGACTCTGTCTATGGAGACAAGCCATCCGAGTTCACTGGCAACACCCTGCAGCCTGGC
TGCACCATTATCTGCTATGATTGCTTCCTACCCTTCTCCCACTTCCATTTCTATGTC---
---CAAATC---ATGCTGGTGGTCACACCCTCCATCTTCTATGTCATCTCAGTCCT-CAC
CGGATCACAAGGGAGGAGAGAGTCAAAGTGGAGAGAGGTTATCTATTGCAGACCTTGCAA
GAGCTGGCAGCTGGGGGATCCCTGCAGGGACCCAGGCAACAGTTAGTGGGATCCTCTCAC
TTCCTAGAGGGACAACTCCCAGTGGGAAAGGGGATTCTCTCCAAATGCCTTGTGGCTGCA
GCCAAGGACCCCAACCTTCTGTCTTGCAGGATCTTGGTCGTCTACATTGCCCATGAGGTG
CTACAGGCCTTTATGGAGCTGGCTTTCTTGGTGGGGCAATGCTAT--GGTAGGCTT-GAT
GTCTCATAG-TATTTCACCGTCACTCCTACCCCTGTCCTACA---AGTACTGACTGCTTT
GTGTCCAGGGCCACCGAGAATATGACCCTCCTGAACATCATGTTTGG-GGTGGGGTAGGC
TGCTACCTCCTGAACCT-GTGGAGCTATACT-CCTGGGCTGGGTCTTTACCTGCC--

>Dn-NP-cx39.2P *Dasypus novemcinctus* Armadillo. Note that the first Cys codon in the first conserved dokmain is mutated.
ATGAGCCACTGGTGGTTCCTGGGCCAGCTCGTGACCCAAGTGCCAAATCCGTCCACTGTG
ACGGGCAAGGTATGACTCACCATGCTCCTGGCCTCCTGCATCCTGCTGGTCACCTTGCGG
GGAGAAGCTGCCTATGGGGACGAGCAGTCCAAGTTCACCCGCAATCCCCTGCAGCCTGGC
TGCACCAATGTCTGCTGTGGCCACTTCTCACCTGTCTCC-ACTTCCACTCCTGGATCTTC
--CCAGATT---GTGTTGGTG-ACCCACTGCCCATCTTCTGTGTCATCTGTGTCCTGGAC
CAGATCAGGAGGAGAGAGTGGAAATGGAGGGGTCCTTGCTGGAGAATTTGCAAATGCTGG
TGGCTCGGGGAGCTCTGCAGGGACTGCGACTGGGACTCTGGGGTCCTCTTACTTCCTAGA
TGGACAACTCCTGGTGGGAGAGGGGATTCTCCCCAAGTGCCTTGGGGTCACACAGCCCAG
GCCTTGGGCCTGCAGTCACAGAGGGTCTTGGCCGTTTGTGCGGCCGCAC-GGTGCTGCGA
GCCTTTATGGAGCTGGCCTTGCTGGTGG-ACAGTGTTGCCTGTTTGGGTTTGATGTTCCA
CACTTATTTCACTGCCACGCCTATTCCCTGTCCCAC--GAGGACTGA-TGCTTAGTGTCC
TGGGTCAT-GAA--GATGACCT---TGAGCTTCAGGCTCGGGGAGGGGGTGGCTGCTTC
CCCTTGAGGCTAGCGGAGCTGCATCACCTGGGCTGGGTCTTCACCTGCCGGGCACGCTTT
GTGGCCTGTGCCAGCTGCTTCCACTTCTGGAGATGACCTGCTGGTC

Suppl. Fig. 13. Comparisons of human “*GJA4P*” against *connexin39.2* and *GJA4*.

Suppl. Fig. 13A. Alignment of conserved domains in human “*GJA4P*” (NG_026166) against *connexin39.2* (“*gjd2like*”) in various species at protein level.

The *cx39.2* sequences given in Suppl. Fig. 12 were translated to protein and aligned. Among the pseudogenes, only the human sequence is included, as aligning several pseudogenes strongly decreases the total number of identities (*) or similarities (: or .). Also the corresponding sequence from eel (Aj-NN-cx39.2) was included. ?, corresponds to a codon that contains one or more unknown nucleotides or a gap. <, corresponds to a stop codon. n, the first conserved domain is N-terminal to n, and the second conserved domain is C-terminal to n; thus this n corresponds largely to the intracellular loop. The Muscle (https://www.ebi.ac.uk/Tools/msa/muscle/) identity matrix is found in Suppl. Table 8.

```
Hs-GJA4P                  MSDWSFLGWLLTRVQNDSTVVGKVWLT??LVLHILLVALLGSAVC?DEHCKFICNTLRPG
Aj-NN-cx39.2              MGDWSILGRFLTEVQNHSTVIGKIWLTMLLIFRILLVTLVGDAVYSDEQSKFTCNTLQPG
Pv-NP-cx39.2              MSDWSFLGRLLTQVQNHSTVVGKVWLTVLLVFRILLVTLVGDAVYGDEQSKFTCNTLQPG
Pa-XM_006925175           MSDWSFLGRLLTQVQNHSTVVGKVWLTVLLVFRILLVTLVGDAVYGDEQSKFTCNTLQPG
Ra-XM_016138748           MSDWSFLGRLLTQVQNHSTVVGKVWLTVLLVFRILLVTMVGDAVYGDEQSKFTCNTLQPG
Wallaby-NP-cx39.2         MGDWSFLGRLLTEVQNHSTVIGKIWLTALLIFRILLVTLVGDAVYRDEQSKFTCNTLQPG
Koala-XM_020963328        MGDWSFLGRLLTEVQNHSTVIGKIWLTALLIFRILLVTLVGNAVYGDEQSKFTCNTLQPG
Md-XM_001376506           MGDWSFLGRLLNEVQNHSTVIGKIWLTALLIFRILLVTLVGDAIYGDEQSKFTCNTLQPG
                          *.***:**.:*. *** ***:**:***  *::.****:::*.*:  **:.** ****.**

Hs-GJA4P                  CT???????DHFSHFR?GAFQIVLVAVPSIFFVVCVLH<MVNGnRVLAVCTAHVVLRACM
Aj-NN-cx39.2              CNNVCYDTFAPVSHLRFWVFQIVLVSTPSIFYIVYVLHKIAKDnQVLLIYIVHVVLRSIM
Pv-NP-cx39.2              CTNVCYDRFSPVSHFRFWVFQIVLVATPSIFYVIYVLHQIAREnRVLAIYIAHVVLRAFM
Pa-XM_006925175           CTNVCYDRFSPVSHFRFWVFQIVLVATPSIFYVIYVLHQIAREnRVLAIYIAHVVLRAFM
Ra-XM_016138748           CTNVCYDRFSPVSHFRFWVFQIVLVATPSIFYVIYVLHQIAREnRVLAIYIAHVVLRAFM
Wallaby-NP-cx39.2         CTNVCYNSFAPFSHLRFWIFQIVLVATPSIFYIVCLMHQVALEnRVLIIYIAHVVLRSFL
Koala-XM_020963328        CTNVCYNSFAPISHLRFWIFQIVLVATPSIFYIVCVMHQVALEnRVLVIYIAHVVLRSFL
Md-XM_001376506           CTNVCYNSFAPISHLRFWIFQIVLVATPSIFYIVCVLHQVALEnRALIIYIAHVVLRAFL
                          *.         .**:*   ******:.****::: ::* :.   ..* :  .*****: :

Hs-GJA4P                  ELAFLVG???LSGCDMPWLLHCHS?PCPSSPDCFVSRAMRKKIFLNFMC?VGLGCFLLNP
Aj-NN-cx39.2              EIAFLVGQYYLFGFEVPHLFRCETYPCPNRTDCFVSRATEKTIFLNFMFSISLGCFILNI
Pv-NP-cx39.2              ELAFLVGQYYLFGFDVPYLFHCHSYPCPTSTDCFVSRATEKMIFLNFMFGVGVGCFLLNL
Pa-XM_006925175           ELAFLVGQYYLFGFDVPYLFHCHSYPCPTSTDCFVSRATEKMIFLNFMFGVGVGCFLLNL
Ra-XM_016138748           ELAFLVGQYYLFGFDVPYLFHCHSYPCPTSTDCFVSRATEKMIFLNFMFGVGVGCFLLNL
Wallaby-NP-cx39.2         ELGFLVGQYYLFGFDVPHLYRCETYPCPTKTDCFVSRATEKMIFLNFMFGVGLGCFLLSL
Koala-XM_020963328        ELGFLVGQY<LFGFNVPHLYRCETYPCPTKTDCFVSRATEKMIFLNFMFGVGLGCFLLNL
Md-XM_001376506           ELGFLVGQYYLFGFDVPHLYRCETYPCPTKTDCFVSRATEKMIFLNFMFGVGLGCFLLNL
                          *:.****    * * ::* * .* : ***. .*******  * ******  :.:***:*.

Hs-GJA4P                  MELCYLGWVFPCQ
Aj-NN-39.2                VELHYLGWVYIFR
Pv-NP-cx39.2              VELHYLGWVFTYR
Pa-XM_006925175           VELHYLGWVFTYR
Ra-XM_016138748           AELHYLGWVFTCR
Wallaby-NP-cx39.2         AELHYLGWLFTFR
Koala-XM_020963328        AELHYLGWLFTFR
Md-XM_001376506           AELHYLGWLFTFR
                           ** ****::  .
```

Suppl. Fig. 13B. Alignment of conserved domains in human “GJA4P” (NG_026166) against GJA4 (connexin37) from human and eel at protein level.

The human *GJA4P cx39.2* sequence given in Suppl. Fig. 12 were translated to protein and aligned with eel *cx39.2*, human *GJA4*, and the two eel-*gja4* (*cx39.4*) sequences. Identities (*) or similarities (: or .) are indicated below the alignment. . ?, corresponds to a codon that contains one or more unknown nucleotides or a gap. <, corresponds to a stop codon. n, the first conserved domain is N-terminal to n, and the second conserved domain is C-terminal to n; thus this n corresponds largely to the intracellular loop. The Muscle (https://www.ebi.ac.uk/Tools/msa/muscle/) identity matrix is shown in Suppl. Table. 9.

```
Hs-GJA4P            --MSDWSFLGWLLTRVQNDSTVVGKVWLT??LVLHILLVALLGSAVC?DEHCKFICNTLR
Aj-NN-cx39.2        --MGDWSILGRFLTEVQNHSTVIGKIWLTMLLIFRILLVTLVGDAVYSDEQSKFTCNTLQ
Hs-GJA4-Cx37        --MGDWGFLEKLLDQVQEHSTVVGKIWLTVLFIFRILILGLAGESVWGDEQSDFECNTAQ
Aj-NN-cx39.4-1      MSKSDWTFLELLLEQGQVHSTGVGKMWLTVLFLFRVLVLSTAAESVWGDEQSDFVCNTQQ
Aj-NN-cx39.4-2      MSRADWGFLERFLEEGQEYSTGIGRVWLTVLFLFRMLILGTAAESAWDDEQSDFVCNTQQ
                       .** :*  :*   *  ** :*.:***  :::.:*::   ..:.  **:..* *** .

Hs-GJA4P            PGCT???????DHFSHFR?GAFQIVLVAVPSIFFVVCVLH<MVNGnRVLAVCTAHVVLRA
Aj-NN-cx39.2        PGCNNVCYDTFAPVSHLRFWVFQIVLVSTPSIFYIVYVLHKIAKDnQVLLIYIVHVVLRS
Hs-GJA4-Cx37        PGCTNVCYDQAFPISHIRYWVLQFLFVSTPTLVYLGHVIYLSRREnALMGTYVASVLCKS
Aj-NN-cx39.4-1      PGCEAVCYDKAFPISHFRFFILQVIIVASPAIFYLSYAALHARWQnKLLRVYLCVTVLKL
Aj-NN-cx39.4-2      PGCELACYDRAFPISHFRFFVLQVIFVSTPTIFYFIYVALRMGWEnKLLCAYTLSIVLKV
                    ***          .**:*   :*.::*: *::.:.  .     .  ::       : .

Hs-GJA4P            CMELAFLVG???LSGCDMPWLLHCHS?PCPSSPDCFVSRAMRKKIFLNFMC?VGLGCFLL
Aj-NN-cx39.2        IMEIAFLVGQYYLFGFEVPHLFRCETYPCPNRTDCFVSRATEKTIFLNFMFSISLGCFIL
Hs-GJA4-Cx37        VLEAGFLYGQWRLYGWTMEPVFVCQRAPCPYLVDCFVSRPTEKTIFIIFMLVVGLISLVL
Aj-NN-cx39.4-1      LLEAAFILVLWHLYGFTVPARYVCQRWPCPHTVDCFVSRPKEKTVFTVYMQAMAGVSLLF
Aj-NN-cx39.4-2      LLEAGFILGLWFLYGFVVHAKYVCQRPPCPHTVDCFVSRPTEKTIFTVYMQAIAGVSMLL
                     :* .*:     * *  :     *   ***   ******.  *.:*  :*  :.  .:::

Hs-GJA4P            NPMELCYLGWVFPCQ
Aj-NN-cx39.2        NIVELHYLGWVYIFR
Hs-GJA4-Cx37        NLLELVHLLCRCLSR
Aj-NN-cx39.4-1      NLLEVCVLLRRYCCP
Aj-NN-cx39.4-2      NVVEFLYLAQHTVTH
                    * :*.  *
```

Suppl. Fig. 14. Expanded branches from the phylogenetic tree shown in Fig. 1.

For simplicity, we will in the title of the Figures often refer to both the mammalian and teleost sequences using the mammalian annotation.

Suppl. Fig. 14A. Expanded view of mammalian and teleost *GJA1* branch.

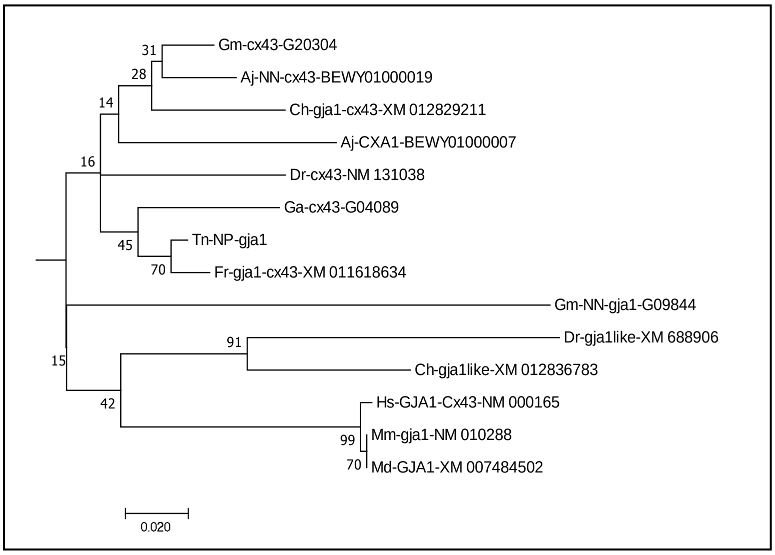

Suppl. Fig. 14B. Expanded view of the mammalian and teleost *GJA3* branch, and the associated teleost *cx39.9*.

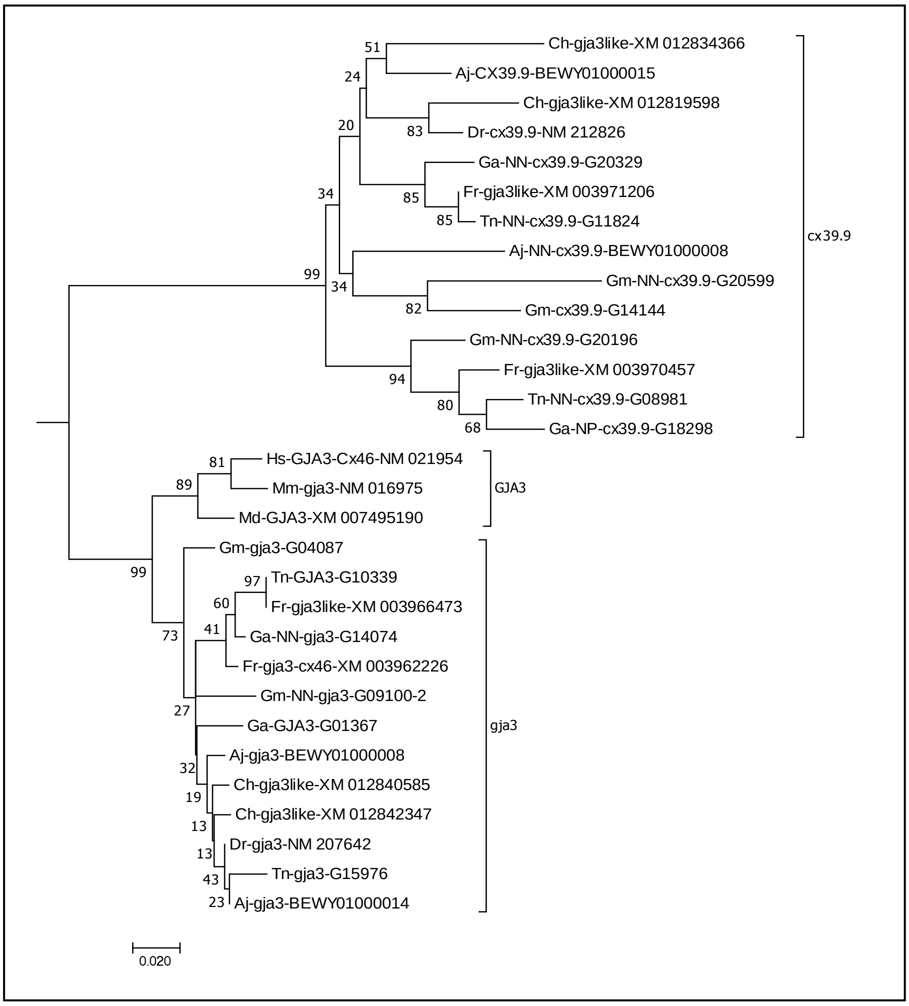

Suppl. Fig. 14C. Expanded view of the mammalian and teleost *GJA4* branch.

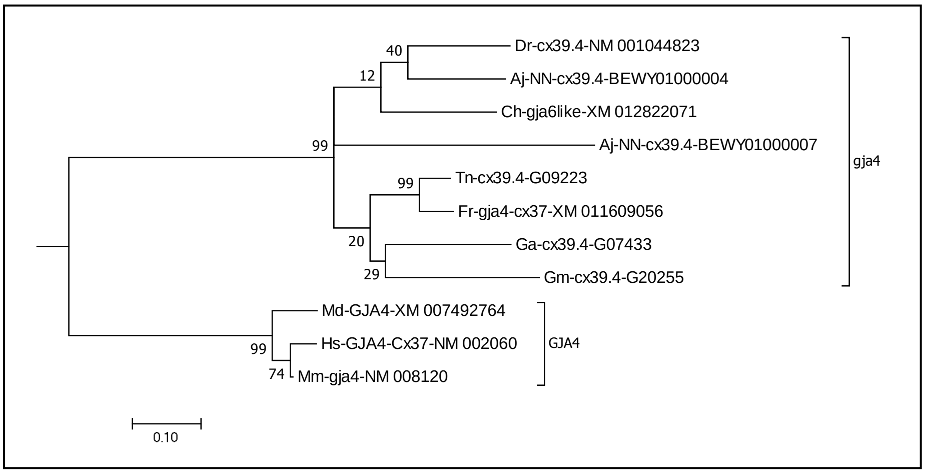

Suppl. Fig. 14D. Expanded view of the mammalian and teleost *GJA5* branch.

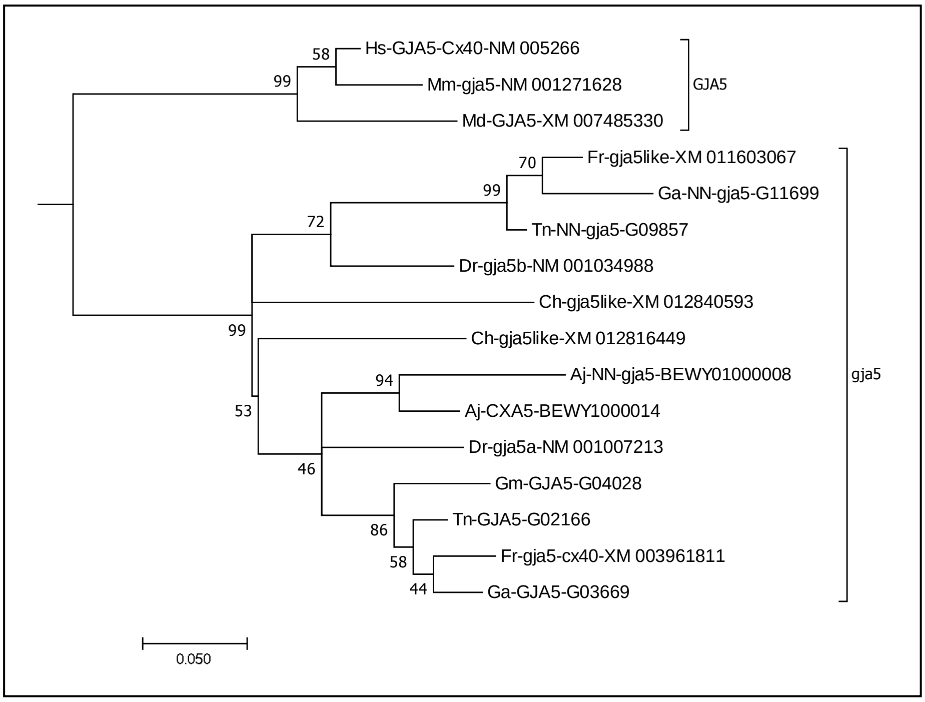

Suppl. Fig. 14E. Expanded view of the mammalian and teleost *GJA9* and *GJA10* branches. In most of the statistical analyses, *GJA10* and *gja10* switched location, i.e., *gja10* was locating outside ((*GJA9* – *gja9*) - *GJA10*).

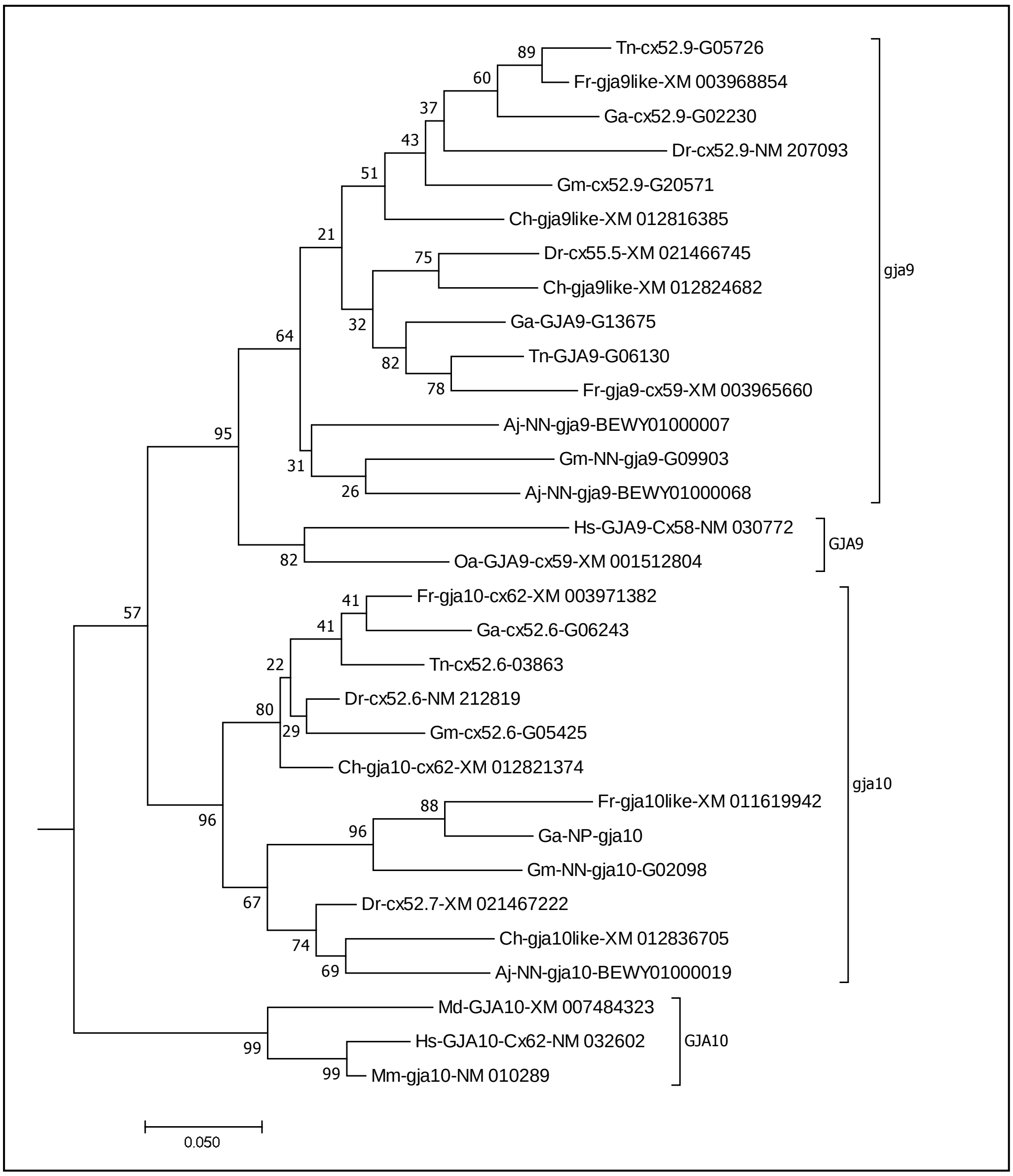

Suppl. Fig. 14F. Expanded view of teleost *cx34.5* and *cx32.2* branches. No mammalian sequence did ever locate together with these two groups.

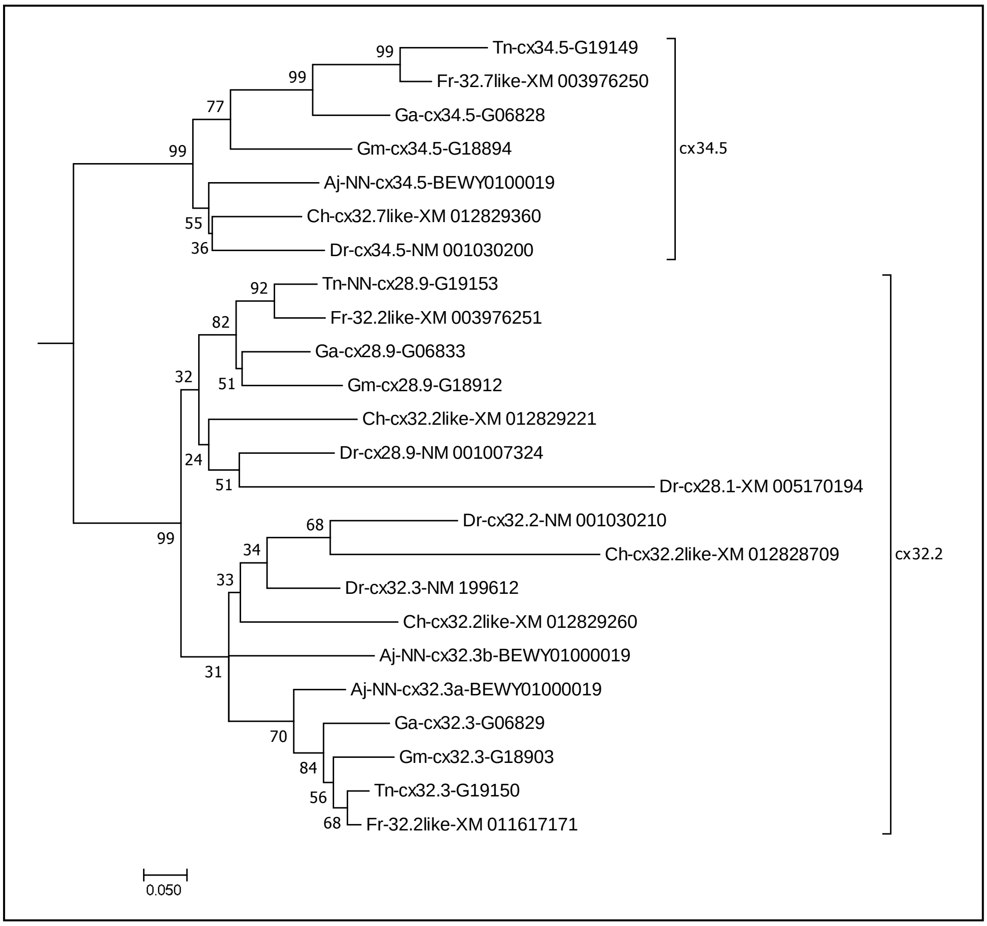

Suppl. Fig. 14G. Expanded view of mammalian and teleost *GJB1*.

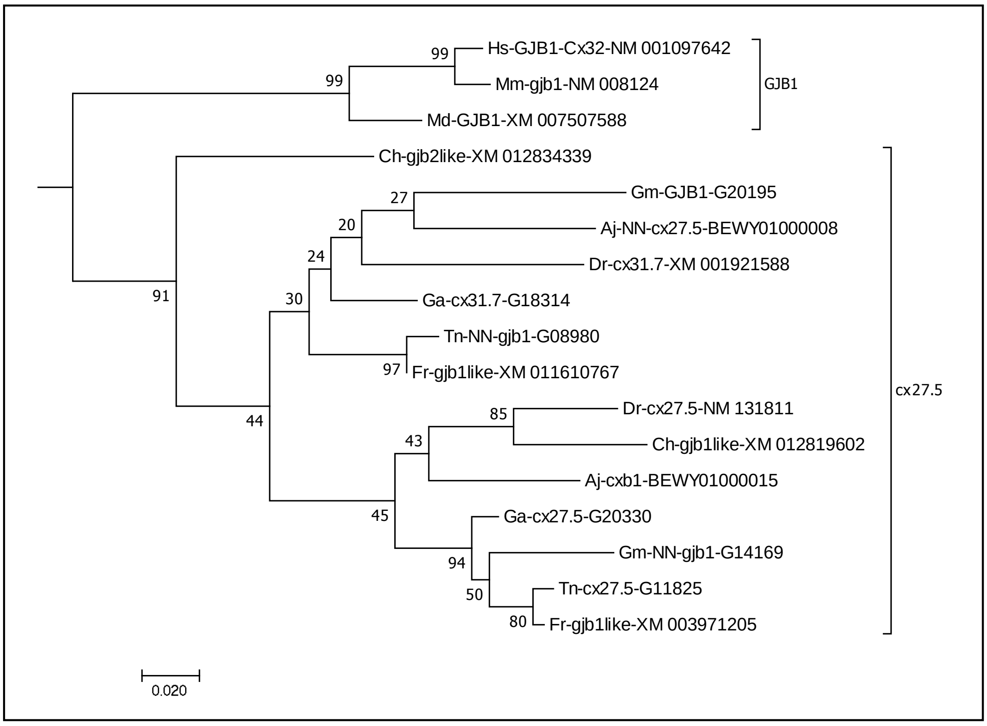

Suppl. Fig. 14H. Expanded view of mammalian *GJB2* and *GJB6*, and teleost *cx30.3*. *GJB2* and *GJB6* always located together in a dichotomous topology, and *cx30.3* did never locate in a dichotomous topology with either *GJB2* or *GJB6*. Thus, there is no reason to claim that *cx30.3* is more closely connected with *GJB2* than with *GJB6*, as the naming of some *cx30.3* sequences could suggest.

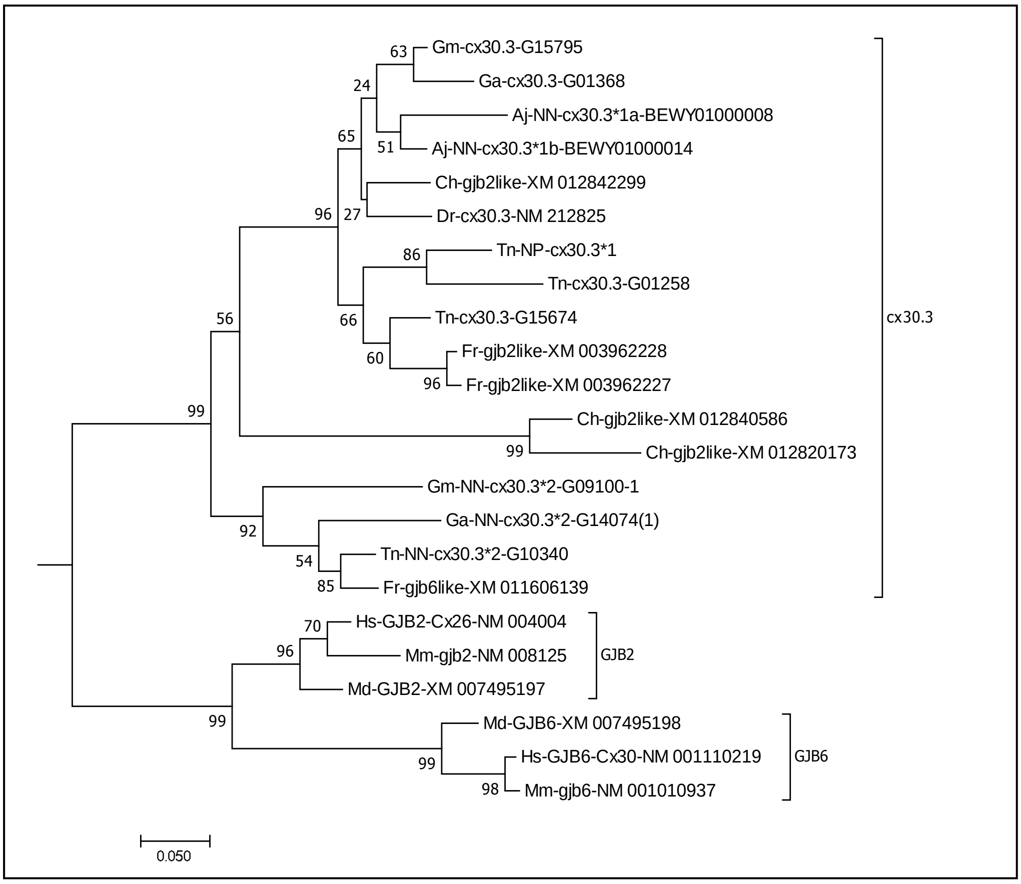

Suppl. Fig. 14I. Expanded view of mammalian *GJB3* and teleost *cx35.4*. These two groups located together in all analyses, and it is reason to suggest that these groups are orthologs, despite that the teleost sequences are lacking the hallmark of mammalian GJB3 protein sequences, the CX_5CX_5C motif in the second extracellular loop, but rather have the standard CX_4CX_5C motif.

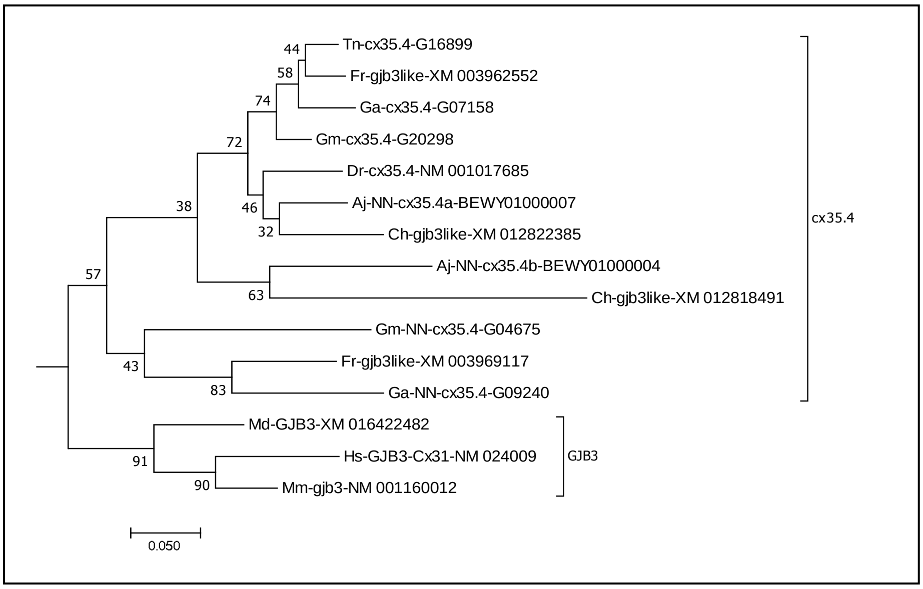

.

Suppl. Fig. 14J. Expanded view of mammalian *GJB4* and *GJB5*, and teleost *cx34.4*. *GJB4* and *GJB5* always located together in a dichotomous topology, and *cx34.4* did never locate dichotomously with either *GJB3* or *GJB4*. Thus, there is no reason to claim the *cx34.4* is more closely related to *GJB4*, as the naming of some *cx34.4* sequences could suggest.

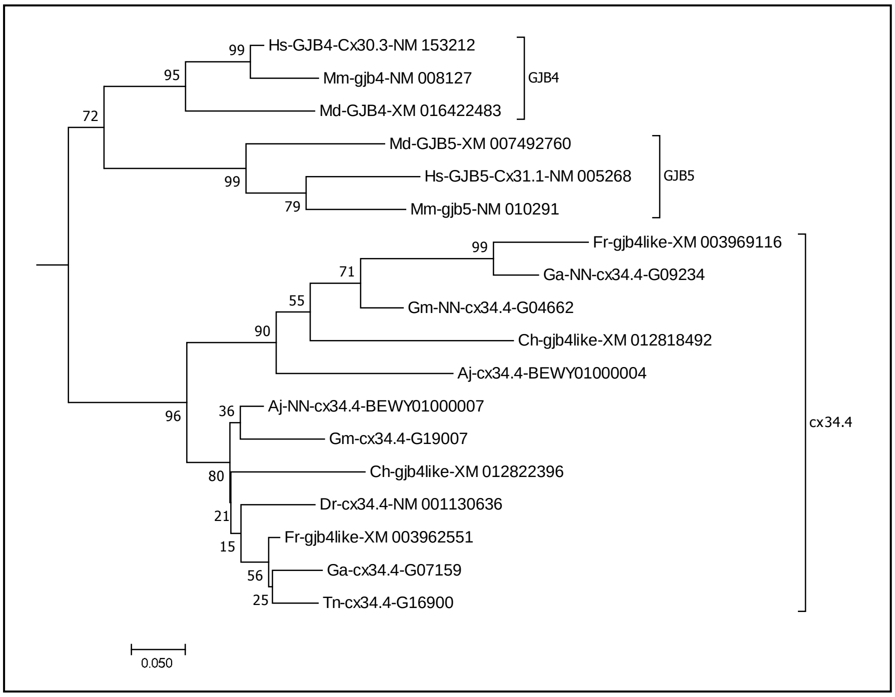

Suppl. Fig. 14K. Expanded view of mammalian and teleost *GJB7*.

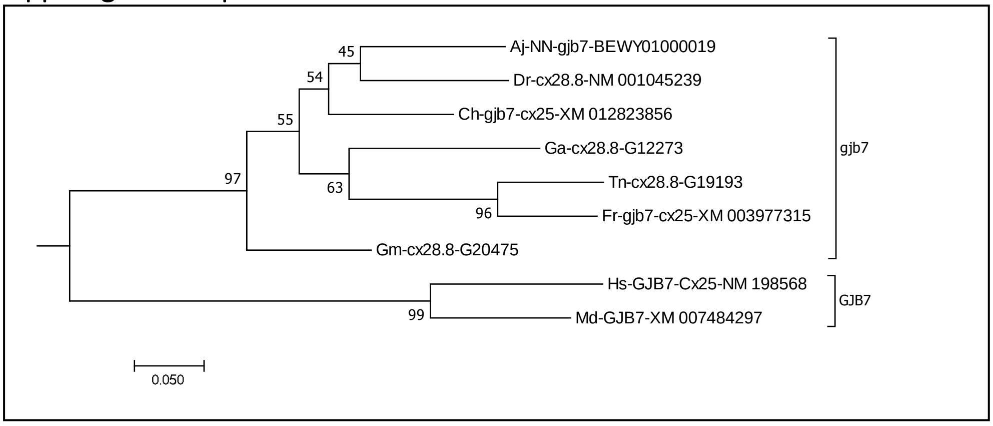

Suppl. Fig. 14L. Expanded view of the teleost *cx28.6* group, and its relationship with *GJB3*/*GJB4*/*GJB5*. *Cx28.6* located in most cases outside *GJB3*/*GJB4*/*GJB5* as this figure illustrates, but in some cases it was located outside the *GJB3-cx35.4* clade, but generally with poorer statistics. Thus, there is no reason to claim that *cx28.6* is more closely related to *GJB4*, as the naming of some sequences could suggest.

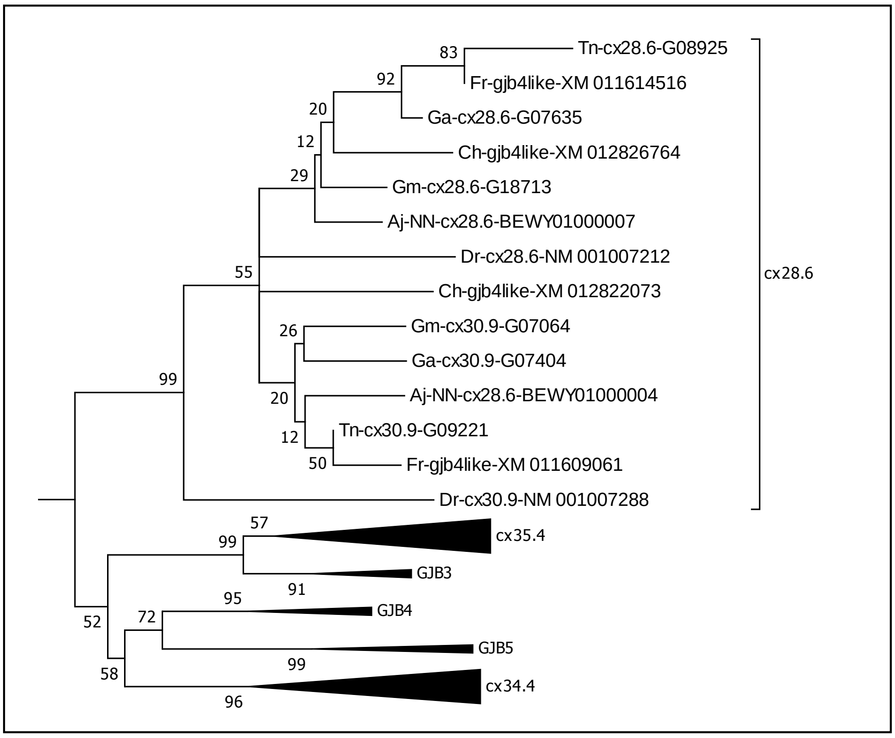

Suppl. Fig. 14M. Expanded view of eutherian *GJC3* and marsupial *GJC1like* and *GJC2like*. In spite of different names, there was only one single statistical analysis (of 21) (Suppl. Table 1) that did not group them together dichotomously. Thus, it is likely that *GJC1like*/*GJC2like* are orthologs of eutherian *GJC3*.

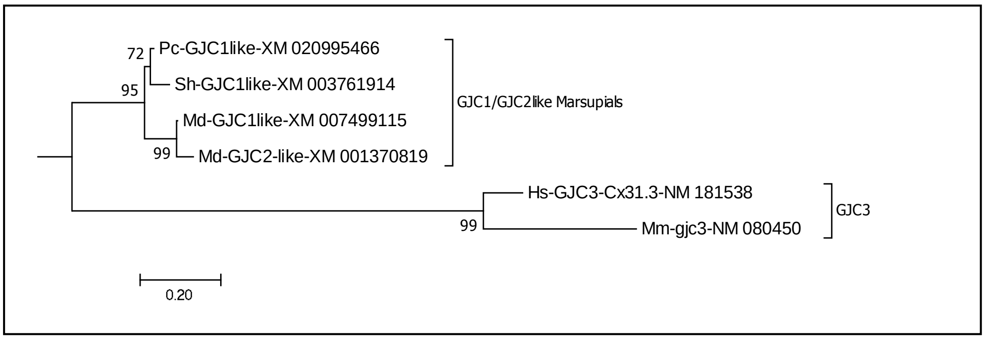

Suppl. Fig. 14N. Expanded view of mammalian and teleost *GJC1* and teleost *cx43.4*. *Cx43.4* had variable locations in the different analyses, and could locate close to *GJC1*/*gjc1*, or *GJC2*/*gjc2*, or outside ((*GJC1 – gjc1*) - (*GJC2 – gjc2*)). Whatever the location of *cx43.4*, the statistics was usually relatively poor (<50).

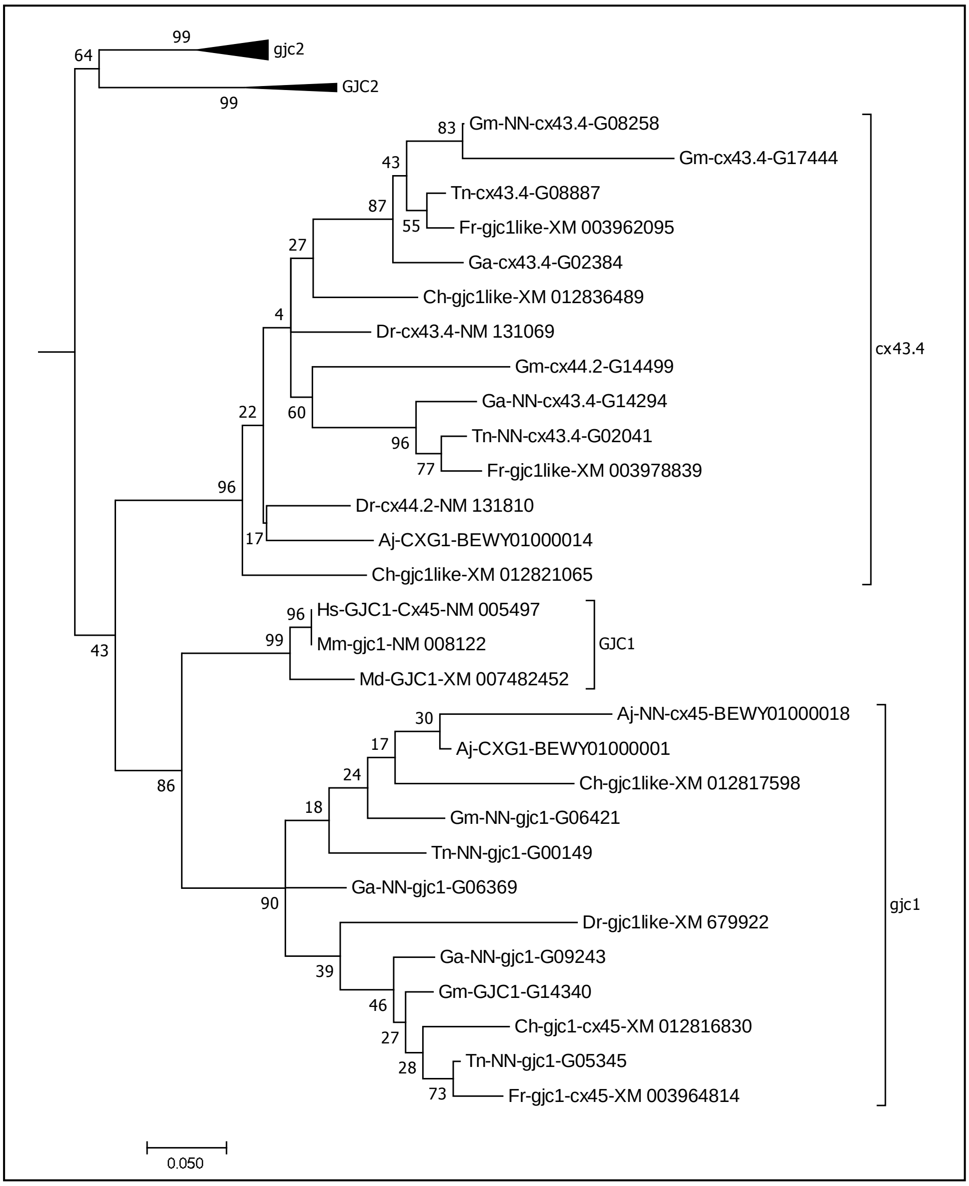

Suppl. Fig. 14O. Expanded view of mammalian and teleost *GJC2*, and its relationship with *GJC1* and *cx43.4*. In phylogenetic analyses using amino acid sequences, the relationship between *GJC2* and *gjc2* was as shown here, while when using nucleotides, the relationship broke, and *GJC2* and *gjc2* located themselves with other relationships to *GJC1*/*gjc1* and *cx43.3*.

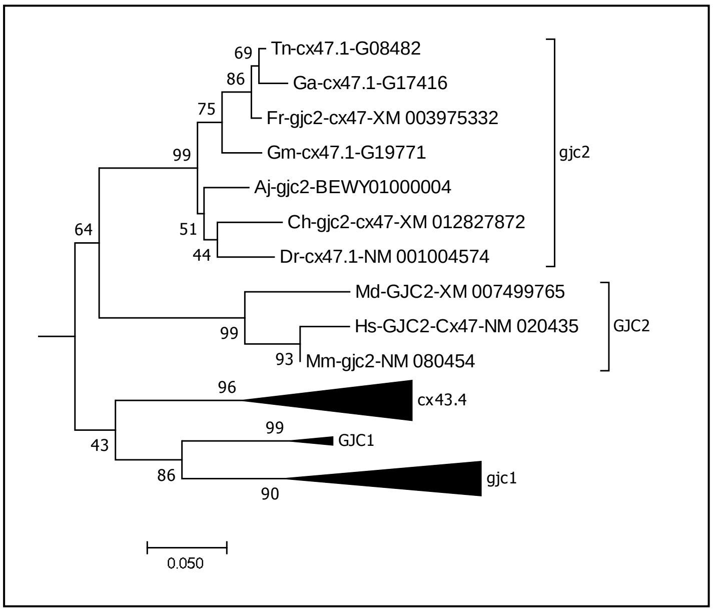

Suppl. Fig. 14P. Expanded view of mammalian and teleost *Cx39.2*. Note the position of human *GJA4P*-NG_026166 among the other mammalian sequences. Further note the confusion in naming of these orthologs (*gjd2like*, *gja4like*, *GJC2like*).

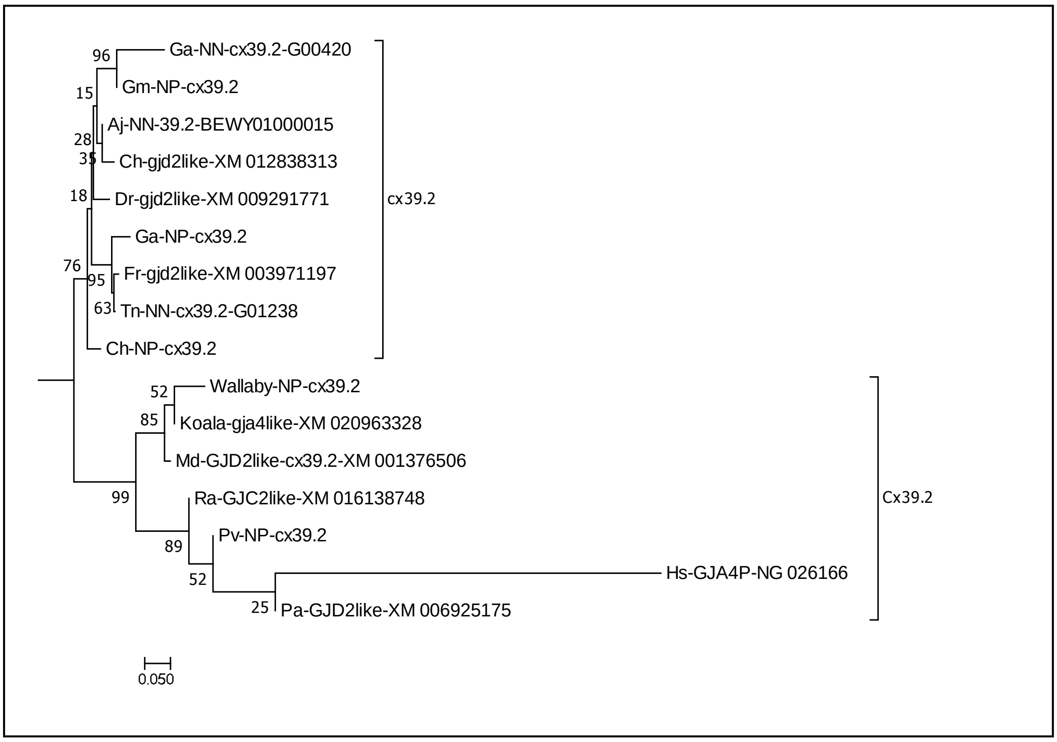

Suppl. Fig. 14Q. Expanded view over the central *GJD2* complex. Mammalian *GJD2* most often located dichotomously together with *gjd2*1*, and with the *gjd2*2* and *gjd2*3* dichotomously located outside, as indicated in this figure. However, in several instances the relative locations of the groups differed, including the non-dichotomous splitting of the *gjd2*2* and *gjd2*3*.

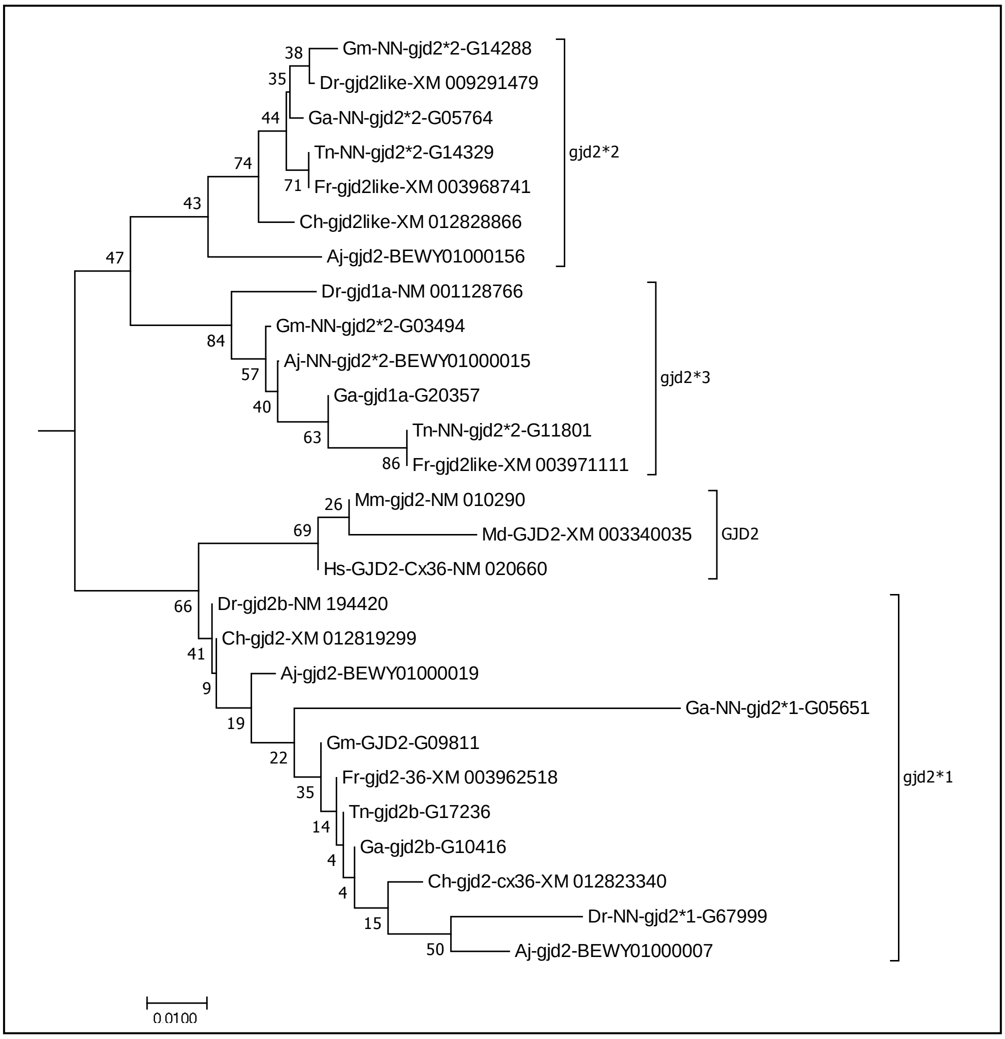

Suppl. Fig. 14R. Expanded view of mammalian and teleost *GJD3*.

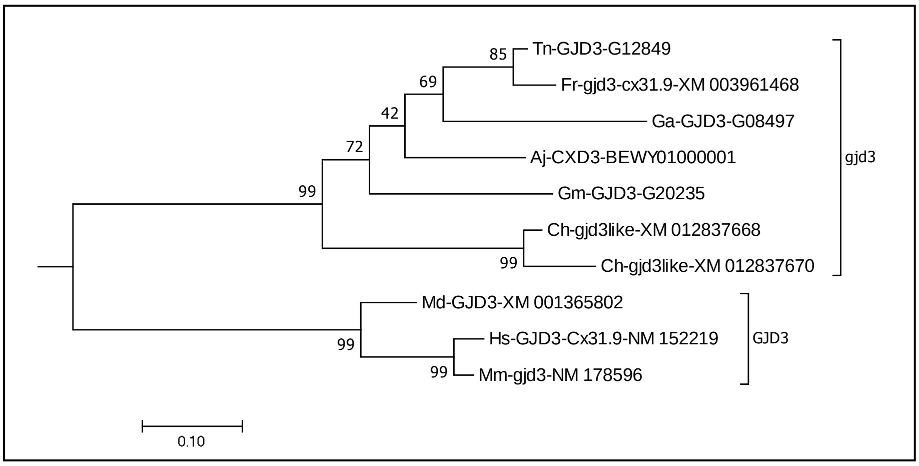

Suppl. Fig. 14S. Expanded view of mammalian and teleost *GJD4*.

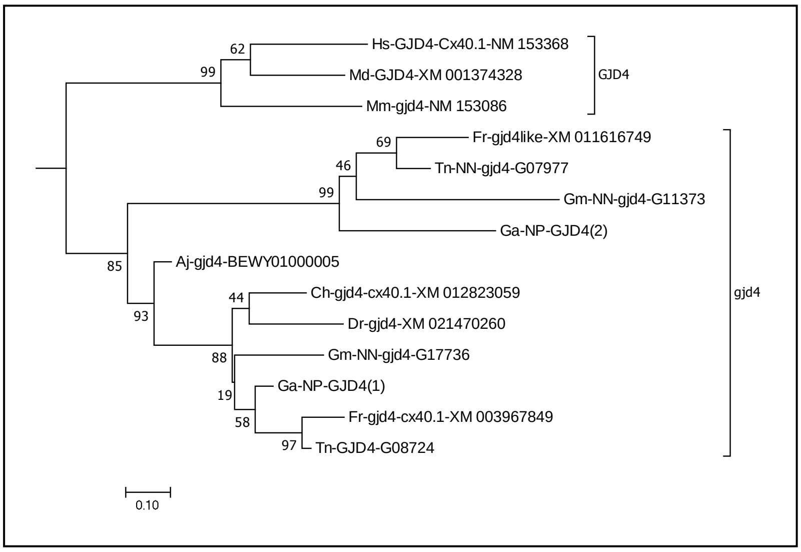

Suppl. Fig. 14T. Expanded view of teleost *cx36.7*. This is one of several groups containing sequences called *gjd2like*. *Cx36.7* most often split off from the root of the *GJD2* complex, but did on occasions associate with the *GJD3* or *GJD4* groups.

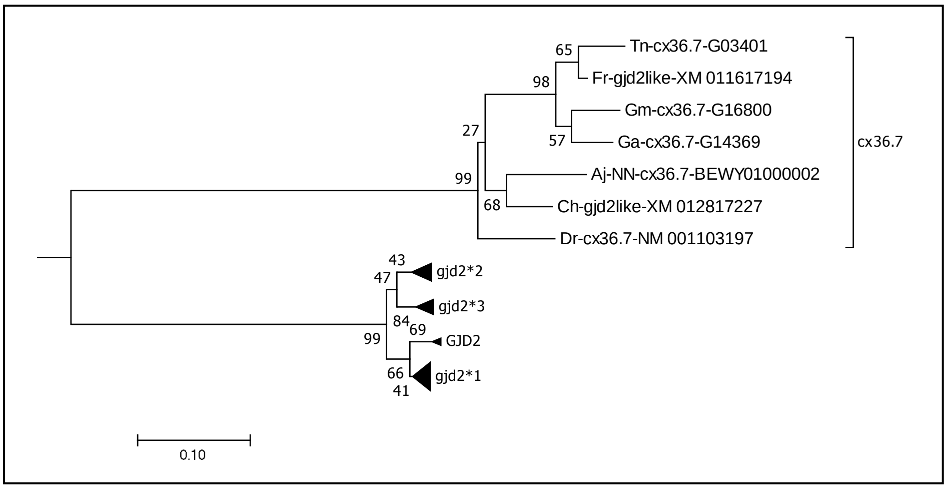

Suppl. Fig. 15. Compressed phylogenetic tree illustrating long-branch attraction between *gjc3*, *gjd4* and *gje1* groups. The tree is made under the same conditions as for Fig. 1 in the manuscript, except that *GJE1*/*gje1* has been added, and that necessary adjustment of alignment (introduction of aligned gaps in all other sequences) were performed. (Figure on next page.)

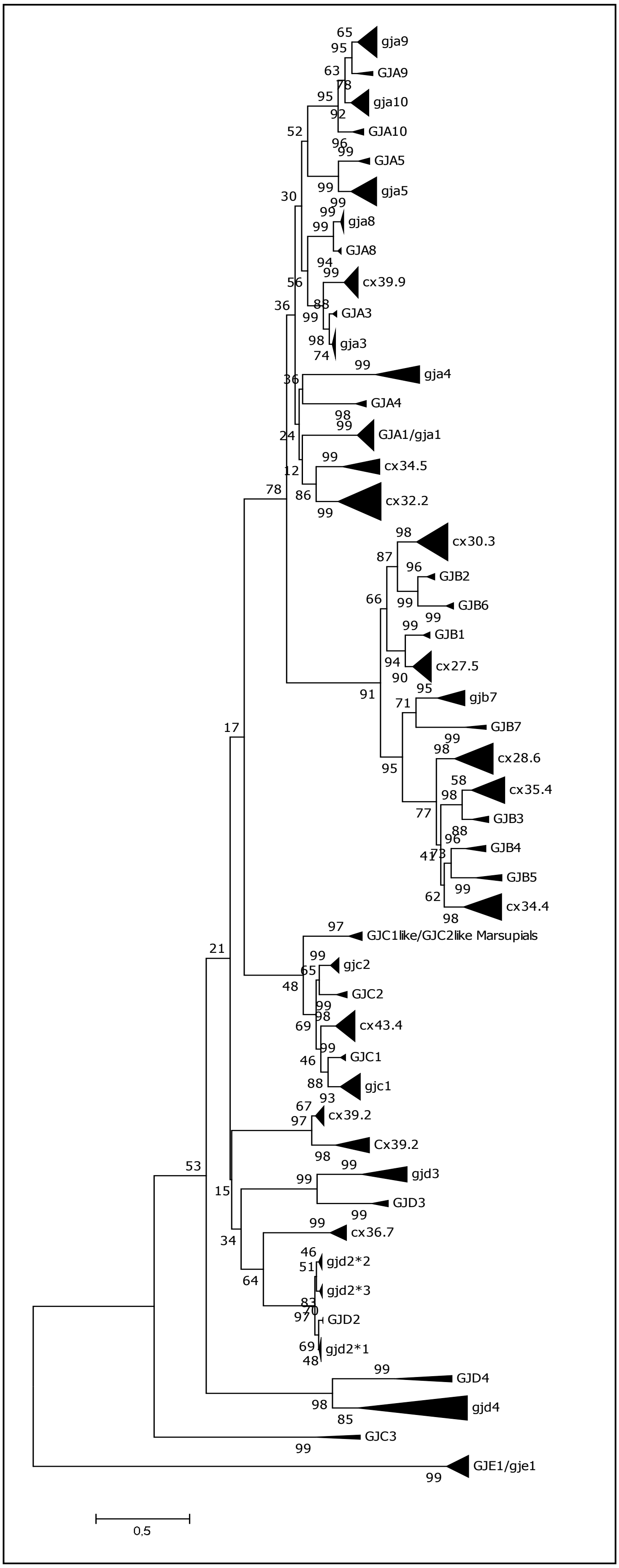
gja9
GJA9
gja10
GJA10
GJA5
gja5
gja8
GJA8
cx39.9
GJA3
gja3
gja4
GJA4
GJA1/gja1
cx34.5
cx32.2
cx30.3
GJB2
GJB6
GJB1
cx27.5
gjb7
GJB7
cx28.6
cx35.4
GJB3
GJB4
GJB5
cx34.4
GJC1like/GJC2like Marsupials
gjc2
GJC2
cx43.4
GJC1
gjc1
cx39.2
Cx39.2
gjd3
GJD3
cx36.7
gjd2*2
gjd2*3
GJD2
gjd2*1
GJD4
gjd4
GJC3
GJE1/gje1
0.5

Suppl. Fig. 16. Searching for positions of connexins lacking in chromosomal assemblies.

Suppl. Fig. 16A. Problem in cod assembly of chromosome 20 at assumed position of *gja5*. Cod scaffold HE571867 contains *gja5* in position 173000-174000. This scaffold was aligned with cod chromosome 20 assembly LR633962 position 0 to 2,000,000 using the alignment option in Blast and word size 32. Dot plot is one of the options on the results page. The position of *gja5* on HE571867 is indicated by the red dotted line. There is an obvious lack of alignment between the scaffold and the chromosomal assembly in the area where *gja5* was expected, and there is an inversion in the sequence corresponding to the scaffold.

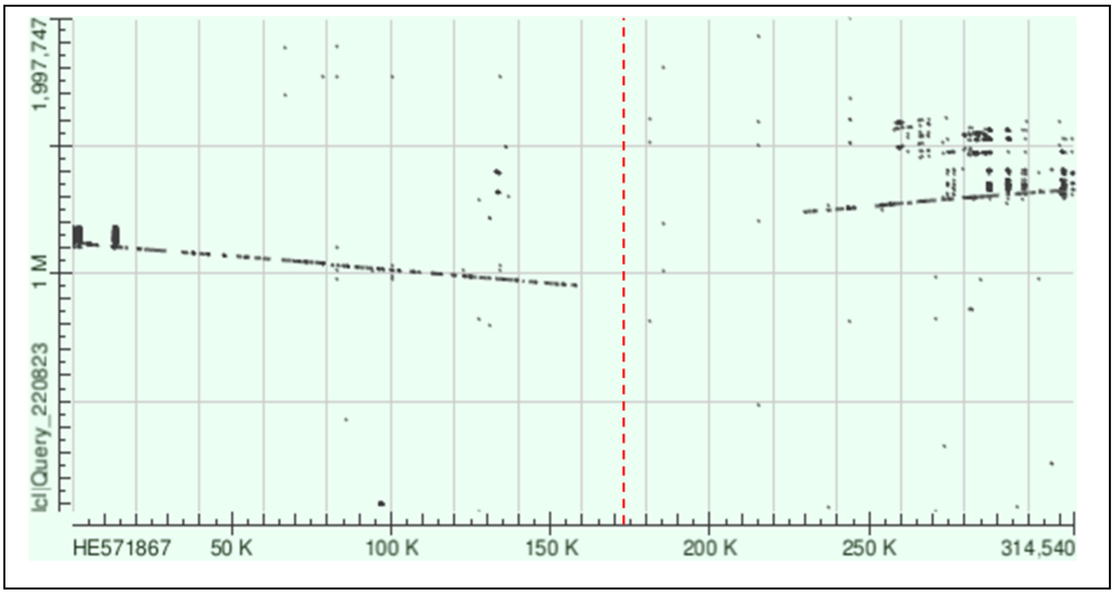

Suppl. Fig. 16B. Alignments with sequences from herring and stickleback point to the same area on cod chromosome 21, indicated expected position of *gja10-cx52.6*. Cod chromosome 21 (LR633963) position 1,000,000 to 4,000,000 was aligned with (upper panel) herring scaffold NW_012220189 (where *cx52.6* is in position 1,390,000) and (lower panel) stickleback scaffold VDFJ01000317 (where *cx52.6* is in position 476,000). Word size 16 was used in both cases. Dot plot is one of the options on the results page. The position of *cx52.6* on the two scaffolds are indicated by the red dotted line, and the blue dotted line indicate the expected position of *cx52.6* on cod chromosome 21. Both herring and stickleback alignments indicate a problem with the cod assembly at position around 2.7 – 2.8 million (as this alignment starts at 1 million). Note the similarities in alignment pattern for the herring and stickleback scaffolds (ovals; see also Suppl. Fig. 16C).

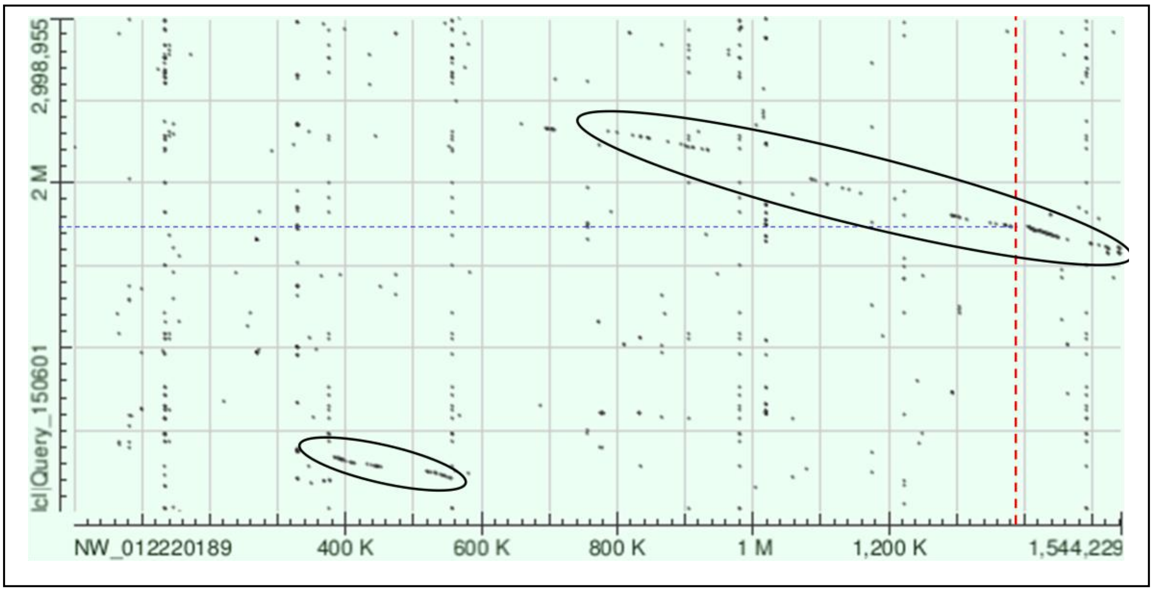

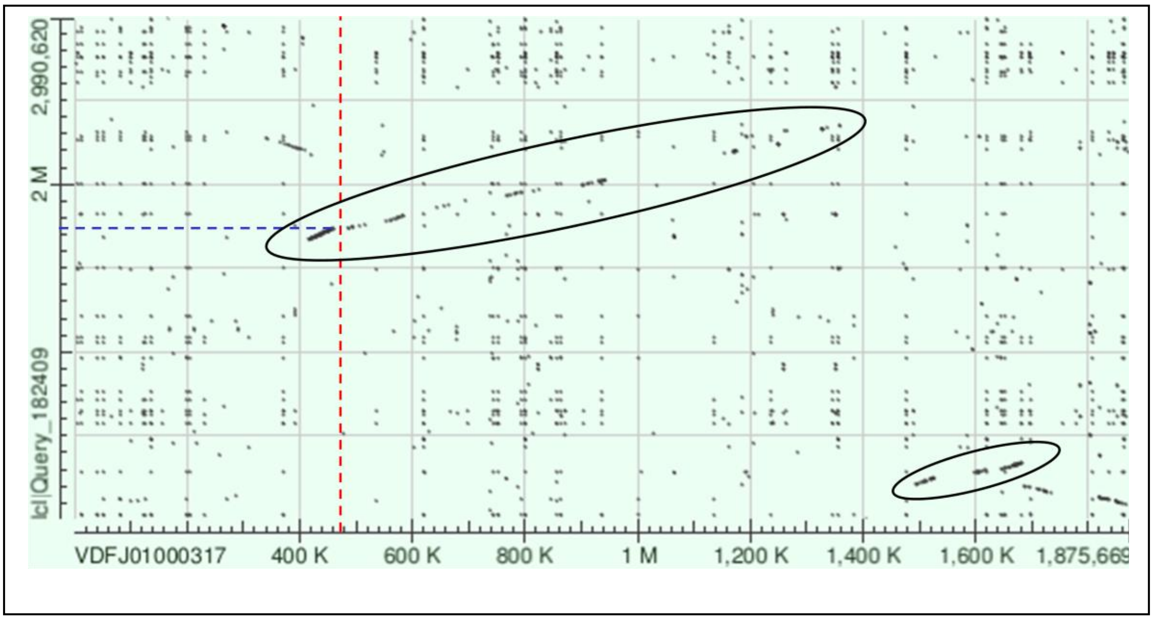

Suppl. Fig. 16C. Alignments of herring and stickleback scaffolds containing *cx52.6*. Herring scaffold NW_012220189 (where *cx52.6* is in position 1,390,000) and stickleback scaffold VDFJ01000317 (where *cx52.6* is in position 476,000), both used in Suppl. Fig. 16C were aligned. Word size 16 was used. Dot plot is one of the options on the results page. The position of *cx52.6* on the two scaffolds are indicated by the red dotted lines. The extensive alignment between these two species, which are evolutionary further apart than either of herring-cod or cod-stickleback that both gave poorer alignments (Suppl. Fig. 16B) supports the possibility of erroneous assembly in the relevant area of cod chromosome 21.

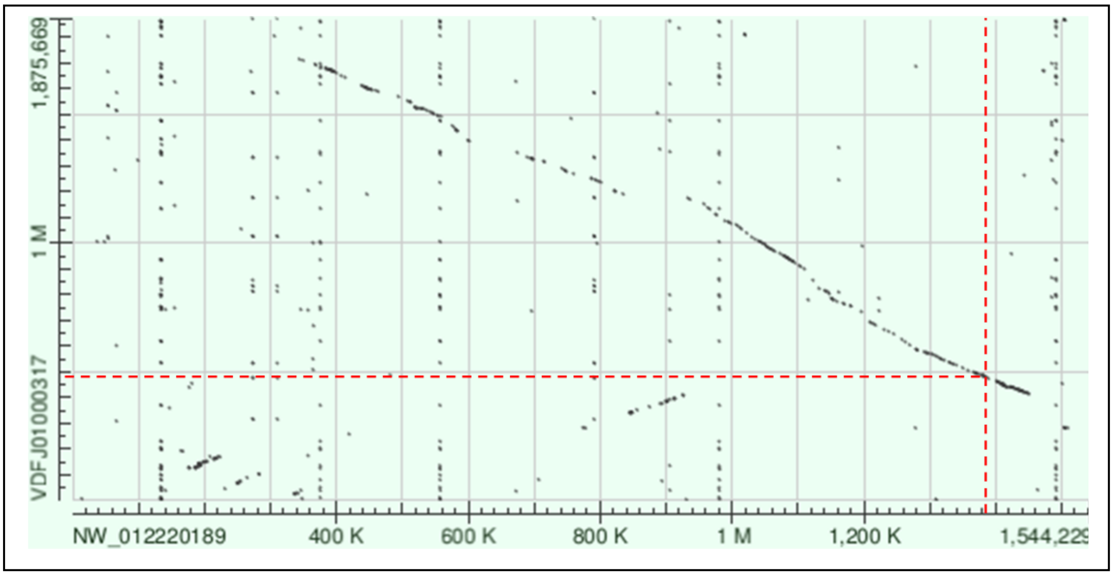

Suppl. Fig. 17. A homogeneous and consistent nomenclature for gap junction protein genes .

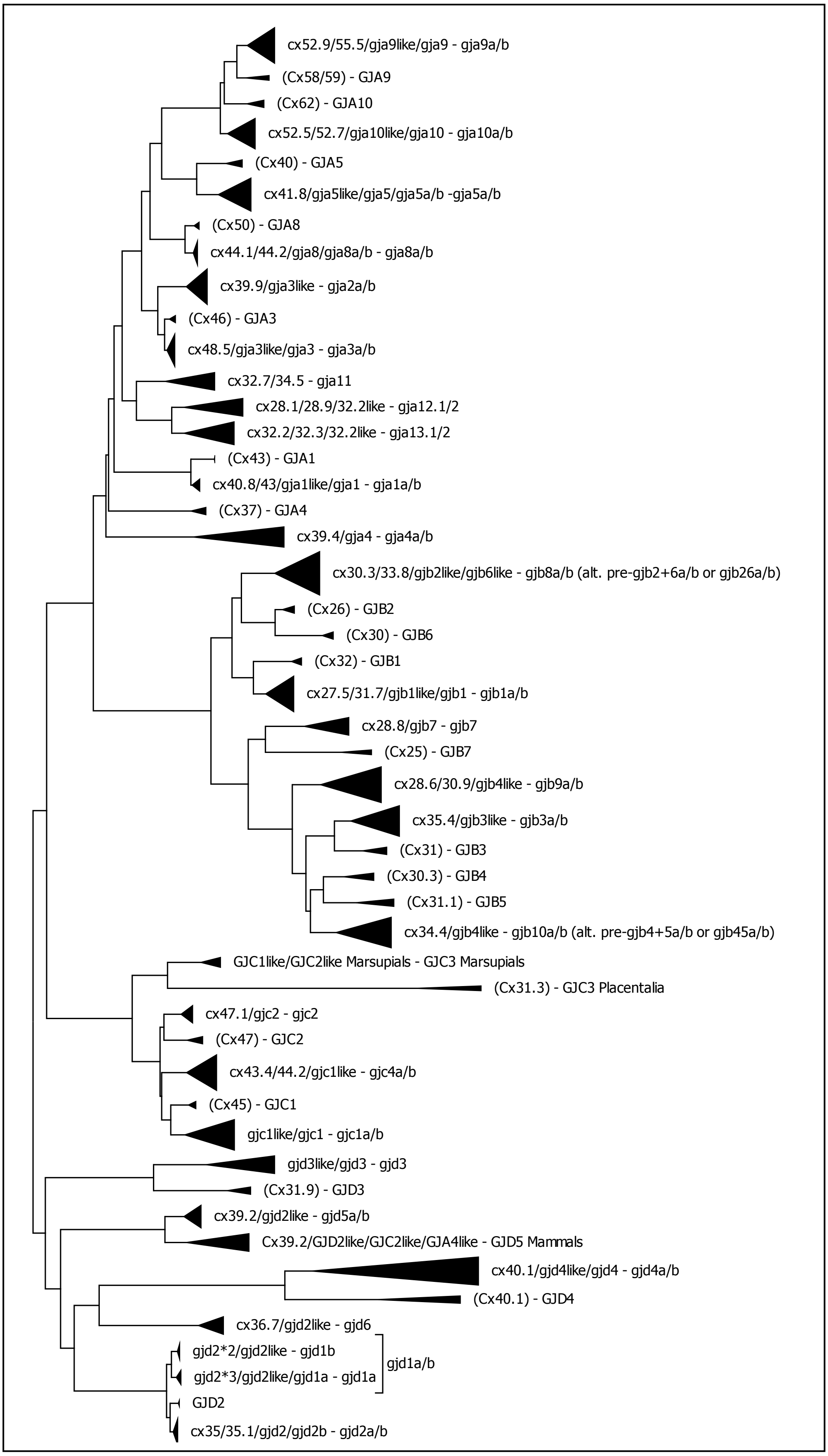

Fig. 17. Legend: The following annotation is used for the compressed branches: UPPER CASE, mammals. The classic size nomenclature for humans is also shown in parentheses. Lower case, teleosts. For the teleosts, some of the previous or commonly used names in the group are given first, and after the dash, our suggested Greek nomenclature name. If we find ohnologs in the group, this is indicated by a/b after the suggested name (e.g., *gja1a/b*). Note that ohnologies may only apply to some of the investigated species (i.e., ohnologs may not be found in all species). For example, for *gja4*, ohnology has only been established in eel. In one case only herring support ohnology (*gjd5*), which potentially means that the other member of the pair must have been lost three times (in eel [diverged before herring], zebrafish [diverged together with herring], and in the line leading to later diverging fishes (the remaining species in this investigation). In two cases, we indicate that duplicated genes within the group probably have been generated by tandem gene duplication (*gja12.1/2* and *gja13.1/2*).

The tree was made using the Neighbor-Joining method at amino acid level. The substitution model was JTT, and the rate variation among sites was modelled with a gamma distribution = 1.0. To simplify the tree, all sequences within the *GJE1* group were excluded, together with the pseudogenes within the *Cx39.2* (*GJD5*) group, except for the human pseudogene with accession number NG_026166. Additionally, a single sequence that often branched off from the stem of the corresponding group was excluded (Aj-NN-*32.3b*-BEWY01000019). Similarly, sequences that disturbed a clear dichotomy for GJA1/gja1 (Mm-*gja6*-NM_01001496, Dr-*gja1like*-XM_NM_688906, Ch-*gja1like*-XM_012836783, Gm-NN-*gja1*-G09844, Aj-*CXA1*-BEWY01000007) were excluded. This gave a total of 347 sequences in this tree. The root branches of the *gjd* subfamily have been fused using the root function in the MEGA Tree Explorer.

Suppl. Fig. 18. Schematic outline of the major procedures.

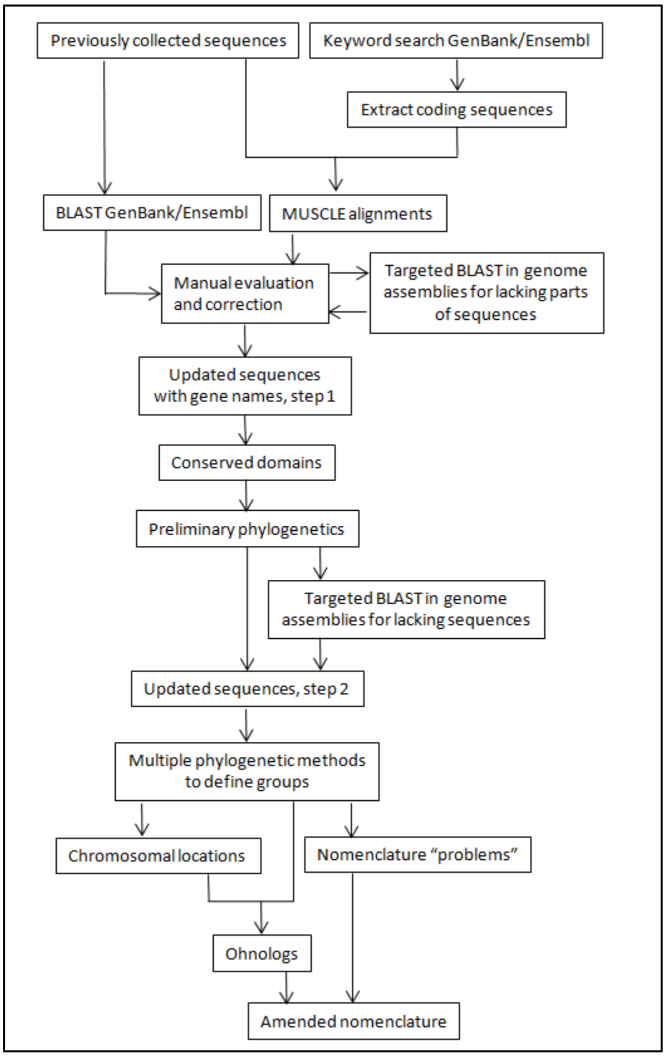

Supplement: Supplementary file 1 — Additional file 1. Suppl. Figure 1. Human (Homo sapiens) connexins. Suppl. Figure 2. Mouse (Mus musculus) connexins. Suppl. Figure 3. Opossum (Monodelphis domestica) connexins. Suppl. Figure 4. GJC1like and GJA9 connexin sequences from other marsupials and platypus. Suppl. Figure 5. Zebrafish (Danio rerio) connexins. Suppl. Figure 6. Japanese pufferfish (Fugu; Takifugu rubripes) connexins. Suppl. Figure 7. Green spotted pufferfish (Tetraodon nigroviridis) connexins. Suppl. Figure 8. Three-spined stickleback (Gasterosteus aculeatus) connexins. Suppl. Figure 9. Atlantic herring (Clupea harengus) connexins. Suppl. Figure 10. Atlantic cod (Gadus morhua) connexins. Suppl. Figure 11. Japanese eel (Anguilla japonica) connexins. Suppl. Figure 12. Connexin39.2 (“gjd2like”) from mammals. Suppl. Figure 13. Comparisons of human “GJA4P” against connexin39.2 and GJA4. A. Alignment of conserved domains in human “GJA4P” (NG_026166) against connexin39.2 (“gjd2like”) in various species at protein level. B. Alignment of conserved domains in human “GJA4P” (NG_026166) against GJA4 (connexin37) from human and eel at protein level. Suppl. Figure 14. Expanded branches from the phylogenetic tree shown in Fig. 1. A. Expanded view of the mammalian and teleost GJA1 branch. B. Expanded view of mammalian and teleost GJA3 branch, and the associated teleost cx39.9. C. Expanded view of the mammalian and teleost GJA4 branch. D. Expanded view of the mammalian and teleost GJA5 branch. E. Expanded view of the mammalian and teleost GJA9 and GJA10 branches. F. Expanded view of the teleost cx34.5 and cx32.2 branches. G. Expanded view of the mammalian and teleost GJB1 branch. H. Expanded view of mammalian and teleost GJB2 and GJB6 branch, and teleost cx30.3 branches. I. Expanded view of the mammalian GJB3 and teleost cx35.4 branches. J. Expanded view of mammalian GJB4 and GJB5, and teleost cx34.4. K. Expanded view of the mammalian and teleost GJB7 branch. L. Expanded view of the teleost cx28.6 group, and i [file 12864_2020_6620_MOESM1_ESM.pdf]
