# Supplementary material for: Phylogeny of teleost connexins reveals highly inconsistent intra- and interspecies use of nomenclature and misassemblies in recent teleost chromosome assemblies
Source: BMC Genomics. 2020 Mar 11;21:223. doi: 10.1186/s12864-020-6620-2 (PMC7066803; doi:10.1186/s12864-020-6620-2)
Supplement: Supplementary file 2 — Additional file 2. Suppl. Table 1. Statistical support for clade grouping. Suppl. Table 2. Parameter overview for statistical analyses of phylogenetic trees. Suppl. Table 3. Comparison between zebrafish connexin sequences from Ensembl and GenBank. Suppl. Table 4. Comparison between Fugu connexin sequences from Ensembl and GenBank. Suppl. Table 5. Comparison between cod connexin sequences from the Ensembl and GenBank assemblies. Suppl. Table 6. Comparison between herring connexin sequences from the GenBank genome assembly predictions and the Ensembl chromosomal level assembly predictions. Suppl. Table 7. Naming of connexin genes in Ensembl and GenBank. Suppl. Table 8. Percentages of amino acid identities between conserved domains in mammalian Cx39.2, including human “GJA4P”-NG_026166, and eel cx39.2 (one of the “gjd2like” sequences). Suppl. Table 9. Human GJA4P is more similar to GJD2like (connexin39.2) than GJA4 at nucleotide level. Suppl. Table 10. Ohnology among teleost connexins. [file 12864_2020_6620_MOESM2_ESM.pdf]

Mikalsen SO, Tausen M, í Kongsstovu S. Phylogeny of teleost connexins reveals highly inconsistent intra-and interspecies use of nomenclature and misassemblies in recent teleost chromosome assemblies.

Contents

Suppl. Table 1. Statistical support for clade grouping. 2

Suppl. Table 2. Parameter overview for statistical analyses of phylogenetic trees. 4

Suppl. Table 3. Comparison between zebrafish connexin sequences from Ensembl and GenBank. 6

Suppl. Table 4. Comparison between Fugu connexin sequences from Ensembl and GenBank. 8

Suppl. Table 5. Comparison between cod connexin sequences from the Ensembl and GenBank assemblies. 10

Suppl. Table 6. Comparison between herring connexin sequences from the GenBank genome assembly predictions and the Ensembl chromosomal level assembly predictions. 12

Suppl. Table 7. Naming of connexin genes in Ensembl and GenBank. 14

Suppl. Table 8. Percentages of amino acid identities between conserved domains in mammalian Cx39.2, including human "GJA4P"-NG_026166, and eel cx39.2 (one of the "gjd2like" sequences). 17

Suppl. Table 9. Human *GJA4P* is more similar to *GJD2like* (*connexin39.2*) than *GJA4* at nucleotide level. 17

Suppl. Table 10. Ohnology among teleost connexins. 18

All data in this Supplemental Information have been collected and curated manually. Human errors and inconsistencies cannot be excluded. We would be grateful if detected errors and major inconsistencies are reported to us (sveinom@setur.fo).

Suppl. Table 1. Statistical support for clade grouping. It is referred to Fig. 1 in the paper for the naming of the different groups. To avoid some of the long-branch attraction and affected statistics, the *GJE1*/*gje1* group and the pseudogenes in the *Cx39.2* group, except the human pseudogene, were omitted in these statistical runs. The parameters for each run are given in Suppl. Table 2. For simplicity, the number of the analyses was counted from 1 when using the amino acid sequences, and from 20 when using nucleotide sequences. The white columns indicate bootstrap statistics (500 iterations) and the grey columns indicate interior branch statistics (500 iterations). The phylogenetic methods are abbreviated as follows: NJ, Neighbor Joining; ML, Maximum Likelihood; ME, Minimum Evolution; MP, Maximum Parsimony.

Mammal or mammal-teleost	Teleost	Sum>50 (Total)[E]	Amino acids											Nucleotides									
Main model			NJ					ML		ME			MP	NJ				ML			ME		MP
Analysis # (Suppl Table 2)			1	2	3	4	5	6	7	8	9	10	11	20	21[D]	22	23	24	25	26	27	28	29
GJA1	gja1	21/21	A	A	A	A	A	A	A	A	A	A	A	A	A	A	A	A	A	A	A	A	A
-	cx34.5-32.2	21/21	78	99	81	99	79	83	85	80	99	86	52[B]	82	75	71	99	82	88	82	83	99	68
GJA3	gja3	18/21	82	75	93	98	87	-	25[F]	74	79	99	-	92	84	99	99	78	84	81	98	99	89
Outside (GJA3-gja3)	cx39.9	19/21	98	99	98	99	99	-	91[B]	94	99	99	-	97	96	99	99	85	85	83	97	99	86
GJA4	gja4	3/21 (17/21)	17[B]	-	31	73	23[B]	19	25	27	-	40	-	41	48	15[B]	64	28	33	28[B]	41	65	-
GJA4 Not dichotomous	gja4	-	-	Tri[C]	-	-	-	-	-	-	Tri	-	-	-	-	-	-	-	-	-	-	-	-
GJA5	gja5	21/21	99	99	99	99	99	99	99	99	99	99	97	99	99	100	99	99	99	99	99	99	99
GJA8	gja8	21/21	99	99	99	99	99	99	99	99	99	99	99	99	99	100	99	99	99	99	99	99	99
GJA9	gja9	18/21	80	96	86	99	90	73	85	90	96	95	50[B]	76	80	76	98	-	-	-	67	97	52
GJA10	gja10		25[B]	-	-	-	-	20[B]	-	58[B]	-	-	31[B]	-	-	-	-	-	-	-	-	-	-
Outside (GJA10-GJA9-gja9)	gja10	11/21	99	Tri	99	Tri	99	99	99	Tri	Tri	-	-	99	99	100	Tri	99	99	99	Tri	Tri	-
GJB1	cx27.5	21/21	96	99	90	99	92	91	83	99	99	98	90	97	96	99	99	89	91	72	99	99	90
GJB2-GJB6	-	21/21	98	99	99	99	98	86	93	99	99	99	90	99	99	100	99	94	93	99	99	99	97
Outside (GJB2-GJB6)	cx30.3	18/21	58	85	66	89	68	59	63[B]	78	88	87	44	56	46	54	82	62	59[B]	62	83	82	-
GJB3	cx35.4	21/21	94	98	97	99	98	95	97	99	99	99	95	92	91	97	99	98	97	98	99[B]	99	96
GJB4-GJB5	-	21/21	92	91	76	70	76	84	80	91	92	72	67	87	83	97	79	83	79	67[B]	75	80	62
Outside (GJB4-GJB5)	cx34.4	7/21 (19/21)	64	54	56	35	47	40[B]	36	64	57	58	41	-	-	56	31	44[B]	37[B]	46[B]	45	32	46
Outside ((GJB3-cx35.4)-(GJB4-GJB5))	cx34.4	4/21	-	-	75[B]	-	73[B]	-	-	-	-	-	-	65	53	-	-	-	-	-	-	-	-
Outside ((GJB3-cx35.4)-cx28.6)	cx34.4	(5/21)	-	-	-	-	-	21	24	-	-	-	-	-	-	-	-	26	28	31	-	-	-
Outside ((GJB3-cx35.4)-(GJB4-GJB5)-cx34.4)	cx28.6	16/21	98	99	94	99	93	96[B]	-	96	99	85	-	74	69	89	99	90[B]	-	-	78[B]	99	-
Outside (GJB3-cx35.4)	cx28.6	2/21 (7/21)	-	-	-	-	-	38	39	-	-	-	-	-	-	-	-	53	47	66	20	-	39
GJB7	gjb7	17/21 (19/21)	80	79	71	91	80	99	94	88	82	81	96	46	43	74	-	88	91	80	76	-	74
GJC1	gjc1	14/21 (20/21)	62	89	80	90	80	25[B]	46	77	76[F]	86	39	54	41	-	65	67	70	56	46	68	40
GJC2	gjc2	10/21 (16/21)	69	49	73	56	57	75	70	69	52	64	55	16[B]	-	-	-	31[B]	34[B]	33[B]	-	-	49

Outside (GJC1-gjc1)	cx43.4	1/21 (4/21)	-	-	25	76	37	-	-	-	-	43	-	-	-	-	-	-	-	-	-	-	-
Outside ((GJC1-gjc1)-(GJC2-gjc2))	cx43.4	4/21 (6/21)	-	-	74[B]	-	90[B]	-	93[B]	32	-	-	-	-	-	47	-	-	88[B]	-	-	-	-
Outside (GJC2-gjc2)	cx43.4	2/21 (12/21)	33	12	-	-	-	59	42	82[B]	12	-	43	13[B]	-	-	-	19[B]	18[B]	17[B]	-	-	34
-	cx43.3-gjc2	(2/21)	-	-	-	-	-	-	-	-	-	-	-	26		-	-	-	-	-	21	Tri	-
GJC3-(GJC1like/GJC2like)	-	14/21 (19/21)	54	31	78	78	89	-	58	63	28	83	-	85	78	87	63	49	54	27[B]	88	62	39
GJD2	gjd2*1	8/21 (14/21)	30[B]	-	-	-	54	53	36[B]	59	76	66	-	-	-	-	93	47	45	45	64	92	34[B]
Outside (GJD2-gjd2*2-gjd2*3)	gjd2*1	4/21 (5/21)	-	-	-	-	-	-	-	-	-	-	24	70[B]	-	100	-	50	-	99[B]	-	-	-
GJD2 Not dichotomous	-	-	-	Tri	-	Tri	-		-	-	-	-	-				-	-	-	-	-		-
GJD2	gjd2*2	(4/21)	8	-	29	-	-	-	-	-	-	-	-	10	-	-	-	-	43	-	-	-	-
Outside (GJD2-gjd2*1)	(gjd2*2-gjd2*3)	11/21 (12/21)	99[B]	-	-	-	99	99	98[B]	99	99	99	-	20[B]	-	99	-	-	99	-	99	-	99
-	gjd2*2-gjd2*3	7/21 (11/21)	44[B]	-	-	-	52	66	60[B]	55	77[F]	47	-	29[B]		59	-	-	-	61[B]	25[B]	Tri	-
Outside (GJD2-gjd2*2-gjd2*1)	gjd2*3	3/21	-	99	99	99	-	-	-	-	-	-	-	-		-	Tri	-	-	-	-	-	-
GJD3	gjd3	21/21	96	99	99	99	99	98	99	99	99	99	98	99	99	99	99	99	99	99	99	99	98
GJD4	gjd4	21/21	99	99	99	99	99	99	99	99	99	99	99	99	99	99	99	99	99	99	99	99	99
Outside GJD2 complex	cx36.7	14/21 (15/21)	59	95	61	97	-	-	-	58	99	67	49[B]	60	59	88	-	51	-	55	64	-	58
Outside (GJD3-gjd3)	cx36.7	(2/21)	-	-	-	-	-	22[B]	-	-	-	-	17	-	-	-	-	.-	-	-	-	-	-
Outside (GJD4-gjd4)	cx36.7	2/21 (8/21)	-	-	29[B]	-	38	-	-	-	-	-	-	39[B]	-	-	67	29[B]	32	-	31[B]	68	-
Cx39.2	cx39.2	21/21	98	99	99	99	99	97	98	96	99	99	99	99	99	100	99	99	98	99	99	99	99[B]
Hs-GJA4P within Cx39.2	-	21/21	Y	Y	Y	Y	Y	Y	Y	Y	Y	Y	Y	Y	Y	Y	Y	Y	Y	Y	Y	Y	Y

A. The mammalian sequences and the teleost sequences mix, so there is no clear dichotomy between mammalian and teleost sequences.

B. Bootstrap value from consensus tree. The consensus tree value was used if the original tree showed unexpected branching patterns.

C. Tri/Tetra: The branching pattern was trichotomous or tetratomous.

D. 53 of the bootstrap cycles failed. Thus, the bootstrap values are based on 447 replications.

E. Numbers without parentheses are summing up the number of analyses where the statistics is >50. The total number of analyses are 21. The numbers in parentheses are total number of analyses where there is some statistical support, no matter how weak.

F. One or two of the sequences split off from the remaining sequences in the group.

Suppl. Table 2. Parameter overview for statistical analyses of phylogenetic trees. The following parameters were permanent (if allowed within the phylogeny method): Rates among sites, gamma = 1.04* (implying exponential distribution of evolutionary rates among the sites); rates among lineages, different (if allowed); missing data treatment, pairwise deletion. All these statistical analyses were run in MEGA7. If the analyses were performed on nucleotide (NT) sequences, only position 1 and 2 in the codons were used. Otherwise, all substitutions are included, whether the analyses were performed on amino acid (AA) level or nucleotide (NT) level. The phylogenetic methods are abbreviated as follows: NJ, Neighbor Joining; ML, Maximum Likelihood; ME, Minimum Evolution; MP, Maximum Parsimony.

Analysis #	Phylogenetic method	Statistical test and number of iterations	Substitution model		Rates among lineages	# gamma categories	Gaps (deletion)	Tree interference	Initial tree	Branch swap filter	ME/MP search level
			AA/NT	Subst. matrix							
1	NJ	Bootstr 500	AA	Equal input	Different	-	Pairwise	-	-	-	-
2	NJ	Int branch 500	AA	Equal input	Different	-	Pairwise	-	-	-	-
3	NJ	Bootstr 500	AA	Dayhoff	Same	-	Pairwise	-	-	-	-
4	NJ	Int branch 500	AA	Dayhoff	Same	-	Pairwise	-	-	-	-
5	NJ	Bootstr 500	AA	JTT	Same	-	Pairwise	-	-	-	-
6	ML	Bootstr 500	AA	Equal input	-	2	Partial (90% coverage)	Nearest neighbor interchange	NJ	None	-
7	ML	Bootstr500	AA	JTT	-	2	Partial (90% coverage)	Nearest neighbor interchange	NJ	None	-
8	ME	Bootstr 500	AA	Equal input	Different	-	Pairwise	Close neighbor interchange	NJ	-	1
9	ME	Int branch 500	AA	Equal input	Different	-	Pairwise	Close neighbor interchange	NJ	-	1
10	ME	Bootstr500	AA	Dayhoff	Different	-	Pairwise	Close neighbor interchange	NJ	-	1
11	MP	Bootstr500	AA	-	-	-	Partial (90% coverage)	Subtree-Pruning-Regrafting	10 (# initial trees)	-	1
20	NJ	Bootst 500	NT	Tamura 3 param.	Different	-	Pairwise	-	-	-	-
21	NJ	Bootstr 500	NT	Tamura-Nei	Different	-	Pairwise	-	-	-	-
22	NJ	Bootstr 500	NT	Max Comp likelihood	Different	-	Pairwise	-	-	-	-

23	NJ	Intbranch500	NT	Max Comp likelihood	Different	-	Pairwise	-	-	-	-
24	ML	Bootstr500	NT	Tamura 3 param.	-	2	Partial (90% coverage)	Nearest Neighbor interchange	NJ	None	-
25	ML	Bootstr500	NT	Tamura-Nei	-	2	Partial (90% coverage)	Nearest Neighbor interchange	NJ	None	-
26	ML	Bootstr500	NT	General Time reversible model	-	2	Partial (90% coverage)	Nearest Neighbor interchange	NJ	None	-
27	ME	Bootstr500	NT	Max Comp likelihood	Different	-	Pairwise	Close neighbor interchange	NJ	-	1
28	ME	IntBr 500	NT	Max Comp likelihood	Different	-	Pairwise	Close neighbor interchange	NJ	-	1
29	MP	Bootstr500	NT	-	-	-	Partial (90% coverage)	Subtree-Pruning-Regrafting	10	-	1

*An analysis of estimated gamma for the whole set of amino acid sequences was performed, indicating a gamma of approximately 1. A number of analyses were performed with different gamma values surrounding 1. Using a gamma value slightly above 1 reduced the number of instances where single or a few sequences split out of its/their group, making the branching pattern (and corresponding statistics) cleaner. Thus, gamma = 1.04 was chosen in the cases where the gamma value could be specified in the parameters.

Suppl. Table 3. Comparison between zebrafish connexin sequences from Ensembl and GenBank. The sequences were initially extracted from Ensembl, but were later named according to the available GenBank sequences. The **bold font** indicates differences in naming (including not being predicted in one of the databases). The percentages of identities between the Ensembl data and the GenBank predictions are given in the right-most column.
The Ensembl gene numbers are abbreviated as follows: G41799 = ENSDARG00000041799.

Name used in our phylogenetic analyses (from GenBank)	Ensembl name and gene number	Identity (nucleotide)
Dr-*cx43*-NM_131038	*cx43*-G41799	99.91%
Dr-*gja1like*-XM_688906	***cx40.8*-G71192**	**100%**
Dr-*gja3*-NM_207642	*gja3*-G21889	99.25%
Dr-*cx39.9*-NM_212826	*cx39.9*-G04082	100%
Dr-*cx39.4*-NM_001044823	*cx39.4*-G70357	100%
Dr-*gja5a*-NM_001007213	*gja5a*-G40065	99.50%
Dr-*gja5b*-NM_001034988	*gjb5b*-G69450	99.37%
Dr-*gja8b*-NM_131809	*gja8b*-G15076	100%
Dr-*gja8a*-NM_001128350	*gja8a*-G69451	100%
Dr-*cx55.5*-XM_012466745	**Not predicted**	**100%**
Dr-*cx52.9*-NM_207093	*cx52.9*-G86453	100%
Dr-*cx52.6*-NM_212819	*cx52.6*-G34930	100%
Dr-*cx52.7*-XM_021467222	*cx52.7*-G57792	100%
Dr-*cx34.5*-NM_001030200	*cx34.5*-G69411	100%
Dr-*cx32.2*-NM_001030210	*cx32.2*-G76789	100%
Dr-*cx32.3*-NM_199612	*cx32.3*-G41787	100%
Dr-*cx28.9*-NM_001007324	*cx28.9*-G41797	99.74%
Dr-*cx28.1*-NM_005170194	*cx28.1*-G41792	100%
Dr-*cx27.5*-NM_131811	*cx27.5*-G35553	100%
Dr-*cx31.7*-XM_001921588	*cx31.7*-G58064	100%
Dr-*cx30.3*-NM_212825	*cx30.3*-G42707	99.75%
Dr-*cx35.4*-NM_001017685	*cx35.4*-G42866	99.78%
Dr-*cx34.4*-NM_001130636	*cx34.4*-G75854	100%
Dr-*cx28.6*-NM_001007212	*cx28.6*-G03925	99.47%
Dr-*cx30.9*-NM_001007288	*cx30.9*-G70362	100%

Dr-*cx28.8*-NM_001045239	*cx28.8*-G71042	100%
Dr-*gjc1like*-XM_679922	**Not predicted**	**100%**
Dr-*cx47.1*-NM_001004574	*cx47.1*-G73896	100%
Dr-*cx44.2*-NM_131810	*cx44.2*-G34207	99.74%
Dr-*cx43.4*-NM_131069	*cx43.4*-G07099	99.65%
**Dr-NN-*gjd*2-G67999 (no hit in GenBank)**	**unnamed-G67999**	
Dr-*gjd2b*-NM_194420	*gjd2b*-G70781	99.13%
Dr-*gjd1a*-NM_001128766	*gjd1a*-G111977	100%
Dr-*gjd2like*-XM_009291479	**Unnamed pseudogene-G35765**	**99.90%**
Dr-*cx36.7*-NM_001103197	*cx36.7*-G17927	98.97%
Dr-*gjd2like*-XM_009291771	**Unnamed-G54744**	**100%**
Dr-*gjd4*-XM_021470260	*GJD4*-G116895	100%
Dr-*cx23*-NM_001013546	*cx23*-G54150	99.3%
Dr-*gje1like*-XM_021473060	*gje1like*-G53062	100%

Suppl. Table 4. Comparison between Fugu connexin sequences from Ensembl and GenBank. These sequences were originally extracted from Ensembl (Cruciani and Mikalsen, 2007), and blasted against the predictions in GenBank. The names have been changed accoring to the naming in GenBank. We have not revised SNPs (or similar) when we changed their naming according to the names in GenBank. Therefore, a number of the sequences are 100% identical to the Ensembl genomic sequence, although when the sequence is Blasted against GenBank sequences, they are generally 99.5-100% identical. **Bold font** indicates differences in naming of the Ensembl entries relative to the GenBank entries, not considering upper and lower case letters. The Ensembl gene numbers are abbrevated as follows: G08716 = ENSTRUG00000008716. **Note.** GenBank has recently updated the annotations/predictions for Fugu (July 2019). A number of the accession numbers have been made obsolete, and replaced by other accession numbers, and partly also revised the identification/name is indicated in the second column of the table. In some cases, the accession we have used (from GenBank before July 2019) is now given to a specific transcript, which may not always be transcript variant 1. This is now indicated in the second column by including the present transcript number variant (e.g., *gja3*-X3-XM_003962226).

Name used in our phylogenetic analyses (extracted from GenBank before July 2019)	Names and accession number in updated GenBank entries (July 2019)	Ensembl names and gene numbers	Identity (nucleotide) 1st and 3rd column
Fr-*gja1-cx43*-XM_011618634	*gja1*-XM_011618634	***cx43*-G08716**	100%
Fr-*gja3-cx46*-XM_003962226	*gja3*-X4-XM_003962226	*gja3*-G19543	100%
Fr-*gja3like*-XM_003966473	No change	*gja3like*-G21588<br>(also gjb6like-G07919)	100%<br>(100% pos 1-633)
Fr-*gja3like*-XM_003971206	No change	***cx39.9*-G12999**	100%
Fr-*gja3like*-XM_003970457	No predicted<br>(*gjb1*-X3-XM_029847578 is 100% id over 23% of coverage)	***cx31.7*-G21650***	100%
Fr-*gja4-cx37*-XM_011609056	*gja4*-XR_003890210	***cx39.4*-G19570**	100%
Fr-*gja5-cx40*-XM_003961811	*gja5*-XM_003961811	*gja5a*-G12287	100%
Fr-*gja5like*-XM_011603067	*gja5like*-XM_029834852<br>(although same locus, it is only 97.97% id to XM_011603067)	*gja5like*-G21114	100%
Fr-*gja8-cx50*-XM_003961810	*gja8*-XM_029841996	*gja8a*-G12308	100%
Fr-*gja9-cx59*-XM_003965660	*gja9*-XM_003965660	**Not predicted**	
Fr-*gja9like*-XM_003968854	No change	***cx52.9*-G06302**	100%
Fr-*gja10-cx62*-XM_003971382	*gja10*-XM_003971382	***cx52.6*-G05845**	100%
Fr-*gja10like*-XM_011619942	*gja10like*-XM_029832702	***NN*-G01958**	100%
Fr-*32.7like*-XM_003976250	*Cx32.7like*-XM_029830045	***cx34.5*-G03388**	99.7% (mean over exons)

Fr-*32.2like*-XM_003976251	*Cx32.2like*-XM_029849996	***cx28.9*-G23632**	100%
Fr-*32.2like*-XM_011617171	No change	***cx32.3*-G21311**	99.88%
Fr-*gjb1like*-XM_011610767	*gjb1like*-X6-XM_011610767	***cx31.7*-G21650***	100%
Fr-*gjb1like*-XM_003971205	No change	***cx27.5*-G12976**	100%
Fr-*gjb2like*-XM_003962228	No change	***cx30.3*-G15746**	100% (98.86% id to seq G15753 below)
Fr-*gjb2like*-XM_003962227	No change	***cx30.3*-G15753**	100%
Fr-*gjb3like*-XM_003962552	No change	***cx35.4*-G06550**	100%
Fr-*gjb3like*-XM_003969117	No change	*gjb3like*-G12651	100%
Fr-*gjb4like*-XM_011614516	No change	*gjb4like*-G24884	100%
Fr-*gjb4like*-XM_011609061	*gjb4like*-X5-XM_011609061	***cx30.9*-G15038**	100%
Fr-*gjb4like*-XM_003962551	*gjb4*-XM_003962551	***cx34.4*-G21372**	100%
Fr-*gjb4like*-XM_003969116	No change	*gjb4like*-G25438	100%
Fr-*gjb6like*-XM_011606139	*gjb6like*-XM_029840970 (same locus as XM_011606139, but only 97.65% id)	*gjb6like*-G07919	100%
Fr-*gjb7*-*cx25*-XM_003977315	*gjb7*-XM_003977315	***cx28.8*-G04810**	100%
Fr-*gjc1*-*cx45*-XM_003964814	*gjc1*-XM_029836267	*GJC1*-G24389	100%
Fr-*gjc1like*-XM_003961198	No change	**Not predicted**	
Fr-*gjc1like*-XM_003978839	No change	*gjc1like*-G03267	100%
Fr-*gjc1like*-XM_003962095	No change	***cx43.4*-G05284**	100%
Fr-*gjc2*-*cx47*-XM_003975332	*gjc2*-X2-XM_003975332	***cx47.1*-G14012**	100%
Fr-*gjd2*-*cx36*-XM_003962518	*gjd2*-XM_003962518	*gjd2*-G14359	100%
Fr-*gjd2like*-XM_003971111	*gjd2like*-X2-XM_003971111	***gjd1a*-G14581**	100%
Fr-*gjd2like*-XM_003968741	*gjd2like*-XM_029844379	*gjd2like*-G05315	100%
Fr-*gjd2like*-XM_011617194	No change	***cx36.7*-G04144**	100%
Fr-*gjd2like*-XM_003971197	No change	*gjd2like*-G25896	100%
Fr-*gjd3*-*cx31.9*-XM_003961468	*gjd3*-XM_003961468	**Not predicted**	
Fr-*gjd4*-*cx40.1*-XM_003967849	*gjd4*-XM_003967849	***GJD4*-G20169**	100%
Fr-*gjd4like*-XM_011616749	No change	**Not predicted**	
Fr-*gje1*-XM_011611785	*gje1*-X4-XM_011611785	***cx23*-G20284**	100%

*Note that the Ensembl prediction is wrong. These are two closely located genes on chromosome 14. *Gja3like* is located at positions 5900872-5901948, and *gjb1like* (the "real" *cx31.7*) is located at positions 5898154-5898954, both in forward direction.

Suppl. Table 5. Comparison between cod connexin sequences from the Ensembl and GenBank assemblies. The sequences were initially extracted from the cod genome assembly in Ensembl. When the GenBank chromosome assembly and gene predictions became available in the summer 2019, the comparison was made by blasting the our modified Ensembl sequences (Suppl. Fig. 10) into GenBank. Grey font: Ensembl had not named the prediction (NN) or the sequence was not predicted (NP). **Bold font:** Identical names in Ensembl and GenBank, with the following simplification: *GJD2* = *gjd2*. The identities between our modifications of the Ensembl data (Suppl. Fig. 10) and the GenBank predictions are given in the right-most column. The Ensembl gene numbers are abbreviated as follows: G20304 = ENSGMOG00000020304.

Name used in our phylogenetic analyses (extracted from Ensembl)	GenBank name and accession number	Identity (nucleotide)
Gm-NN-*gja1*-G09844	*gja1like*-XM_030363165	100%
Gm-*cx43*-G20304	*gja1*-XM_030345664	100%
Gm-NN-*gja3*-G09100-2	*gja3like*-XM_030354576	99.8%
**Gm-*gja3*-G04087**	***gja3*-XM_030344249**	**99.5%**
Gm-NN-*cx39.9*-G20599	*gja3like*-XM_030361297	100%
Gm-*cx39.9*-G14144	*gja3like*-XM_030361290	99.21%
Gm-NN-*cx39.9*-G20196	*gja3like*-XM_030369091	99.82%
Gm-*cx39.4*-G20255	*gja4like*-XM_030346321	99.83%
Gm-*GJA5*-G04028	no hit	
Gm-*gja8a*-G19707	*gja8like*-XM_030342843	100%
Gm-NN-*gja9*-G09903	*gja10like*-XM_030358853	99.6%
Gm-*cx52.9*-G20571	*gja9like*-XM_030347153	99.73%
Gm-NN-*gja10*-G02098	*gja10like*-XM_030356227	99.92%
Gm-*cx52.6*-G05425	no hit	
Gm-*cx28.9*-G18912	*Cx32.2like*-XM_030345669	100%
Gm-*cx32.3*-G18903	*Cx32.2like*-XM_030345666	100%
Gm-NN-*gjb1*-G14169	*gjb1like*-XM_030361303	100%
Gm-*GJB1*-G20195	*gjb1like*-XM_030369092	100%
Gm-NN-*cx30.3*-G09100-1	*gjb6like*-XM_030354646	100%
Gm-*cx30.3*-G15795	*gjb2like*-XM_030343242	99.73%
Gm-*cx28.6*-G18713	*gjb4like*-XM_030355963	100%
Gm-*cx30.9*-G07064	*gjb4like*-XM_030346271	98.86%
Gm-*cx34.5*-G18894	*Cx32.7like*-XM_030345675	100%

Gm-NN-*cx35.4*-G04675	*gjb3like*-XM_030347058	100%
Gm-*cx34.4*-G19007	*gjb4like*-XM_030356267	99.53%
Gm-NN-*cx34.4*-G04662	*gjb4like*-XM_030347057	100%
Gm-*cx35.4*-G20298	*gjb3like*-XM_030356265	100%
Gm-*cx28.8*-G20475	*gjb7*-XM_030344823	100%
Gm-*cx36.7*-G16800	*gjd3like*-XM_030377055	100%
Gm-*GJC1*-G14340	*gjc1like*-XM_030347793	99.59%
Gm-NN-*gjc1*-G06421	*gjc1like*-XM_030340829	99.56%
Gm-NN-*cx43.4*-G08258	*gjc1like*-XM_030343838	100%
Gm-*cx44.2*-G14499	*gjc1like*-XM_030353496	99.89%
Gm-*cx43.4*-G17444	Only genomic hit, no prediction	99.89%
Gm-*cx47.1*-G19771	*gjc2*-XM_030363067	99.92%
Gm-NN-*gjd2*-G14288	*gjd2like*-XM_030337042	98.39%
Gm-NN-*gjd2*-G03494	*gjd2like*-XM_030360298	100%
**Gm-*GJD2*-G09811**	***gjd2-XM*_030357265**	**100%**
Gm-NN-*gjd2*-G01582	*gjd2like*-XM_030345236	100%
Gm-NP-*cx39.2*	*gjd2like*-XM_0303811705	100%
**Gm-*GJD3*-G20235**	***gjd3*-XM_030339390**	**99.77%**
Gm-NN-*gjd4*-G11373	*gjd4like*-XM_030338456	99.83%
Gm-NN-*gjd4*-G17736	*gjd4*-XM_030348429	99.71
**Gm-*GJE1*-G16314**	***gje1*-XM_030345017**	**100%**

Suppl. Table 6. Comparison between herring connexin sequences from the GenBank genome assembly predictions and the Ensembl chromosomal level assembly predictions. The sequences were initially extracted from the herring genome assembly and predictions in GenBank (GCA_000966335). In the summer 2019, a chromosomal level assembly became available (GCA_900700415), and Ensembl released the gene predictions and annotation in September 2019. Comparisons were made by blasting our modifications of the GenBank predictions (Suppl. Fig. 9) into Ensembl. The percentages for the sequence identities between the GenBank predictions and the Ensembl data are given in the right-most column. Note the the identity concerns the genomic sequence in Ensembl, and not the gene prediction itself, as this in many cases differs from the GenBank (and our) predictions. **Bold font**: **Identical names** in Ensembl and GenBank, with the following simplifications: (i) *gjc1* = *GJC1*, and (ii) *gja3like-cx39.9* = *gja3like*. The Ensembl gene number is abbreviated as follows: G15623 = ENSCHAG00000015623.

Name used in our phylogenetic analyses (extracted from GenBank)	Ensembl chromosomal level assembly	
Name and accession #	Name and gene #	Identity(%)
Ch-gja1-cx43 -XM_012829211	*cx43*-G15623	99.91
Ch-gja1like-XM_012836783	*cx40.8*-G23765	100
Ch-gja3like-XM_012842347	*gja3*-G06056*	100
Ch-gja3like-XM_012840585	*NN*-G03786*	100
Ch-gja3like-cx39.9-XM_012834366	***gja3like***-G05396	99.82
Ch-gja3like-cx39.9-XM_012819598	*cx39.9*-G03843	100
Ch-gja6like-XM_012822071	*cx39.4*-G02465	98.57
Ch-gja5like-XM_012816449	*NN*-G00991*	97.01
Ch-gja5like-XM_012840593	*gja5b*-G23474	100
Ch-gja8-cx50-XM_012840595	*gja8b*-G23477	99.91
Ch-NP-gja8-XM_012816450	*NN*-G00910	99.65
Ch-gja9like-XM_012824682	No hit	
Ch-gja9like-XM_012816385	*cx52.9*-G00710	99.51
Ch-gja10-cx62-XM_012821374	*cx52.6*-G22263	99.93
Ch-gja10like-XM_012836705	*cx52.7*-G21865	99.87
Ch-cx32.7-XM_012829360	*cx34.5*-G15504	99.66
Ch-cx32.2like-XM_012829221	*cx28.9*-G15524*	99.73
Ch-cx32.2like-XM_012829260	*cx32.3*-G15512*	99.76
Ch-cx32.2like-XM_012828709	*NN*-G15600*	97.99
Ch-gjb1like-XM_012819602	No hit	
Ch-gjb2like-XM_012834339	*cx31.7*-G05405	99.98

Ch-gjb2like-XM_012842299	*cx30.3*-G06047	99.98
Ch-gjb2like-XM_012820173	*NN*-G02862*	99.74
Ch-gjb2like-XM_012840586	*NN*-G03940*	99.88
Ch-gjb3like-XM_012822385	*cx35.4*-G22760*	100
Ch-gjb3like-XM_012818491	*GJB3*-G02489*	100
Ch-gjb4like-XM_012822073	*cx30.9*-G02449*	99.54
Ch-gjb4like-XM_012826764	*NN*-G09826*	99.88
Ch-gjb4like-XM_012822396	*cx34.4*-G22767*	99.51
Ch-gjb4like-XM_012818492	***gjb4like***-G02461*	100
Ch-gjb7-cx25-XM_012823856	No hit	
Ch-gjc1-cx45-XM_012816830	***GJC1***-G16677	99.80
Ch-gjc1like-XM_012817598	*NN*-G04340*	99.45
Ch-gjc1like-XM_012821065	*cx44.2*-G03801	99.93
Ch-gjc1like-XM_012836489	*cx43.4*-G12428	99.66
Ch-gjc2-cx47-XM_012827872	*cx47.1*-G15884	99.84
Ch-gjd2-cx36-XM_012823340	***gjd2***-G02358	100
Ch-gjd2-XM_012819299	*gjd2b*-G05207	100
Ch-gjd2like-XM_012828866	*NN*-G11324*	99.76
Ch-gjd2like-XM_012817227	*cx36.7*-G08179*	99.63
Ch-gjd2like-XM_012838313	*NN*-G16625*	99.65
Ch-NP-cx39.2	*NN*-G14441	99.82
Ch-gjd3like-XM_012837668	*NN*-G13965*, **	99.82
Ch-gjd3like-XM_012837670	*NN*-G13965*,**	99.1
Ch-gjd4-cx40.1-XM_012823059	*NN*-G03250*	99.50
Ch-gje1like-XM_012822376	*cx23*-G12894*	100

*The Ensembl gene prediction is likely wrong. We (and GenBank) have included sequences that are considered as introns by Ensembl, or we have extended the open reading frame until a reasonable stop codon.
**G13965 predicts two connexins into one transcript.

Suppl. Table 7. Naming of connexin genes in Ensembl and GenBank. Only sequences named in GenBank or Ensembl are included, i.e., predicted sequences without a name and sequences not predicted in GenBank or Ensembl, are not included. The classification has been simplified in the following manners: (i) Upper case names are considered the same as lower case names (*GJA1* = *gja1*), and (ii) names that include both Greek nomenclature and size nomenclature are considered the same as names with only the Greek nomenclature (*gja1-cx43* = *gja1*).

Gene name	Zebrafish (GenBank)	Fugu (GenBank pre-July 2019)	Fugu (GenBank post July 2019)	Fugu (Ensembl)	Tetraodon (Ensembl)	Stickleback (Ensembl)	Herring (GenBank)	Herring (Ensembl; chromosomal level)	Cod (Ensembl)	Cod (GenBank; chromosomal level)
cx43	1			1		1		1	1	
cx40.8								1		
gja1		1	1		1		1			1
gja1like	1						1			1
gja3	1	1	1	1	2	1		1	1	1
gja3like		3	2	1			4	1		4
cx39.4	1			1	1	1		1	1	
cx39.9	1			1				1	1	
gja4		1	1							
gja4like										1
gja5		1	1		1	1			1	
gja5a	1			1						
gja5b	1							1		
gja5like		1	1	1			2			
gja6like							1			
gja8		1	1				1			
gja8like										1
gja8a	1			1	1	1			1	
gja8b	1							1		
gja9		1	1		1	1	1			
gja9like		1	1				1			1
cx55.5	1									
cx52.9	1			1	1	1		1	1	

cx52.6	1			1	1	1		1	1	
cx52.7	1							1		
gja10		1	1				1			
gja10like		1	1				1			2
cx34.5	1			1	1	1		1	1	
cx32.7like		1	1				1			1
cx32.2	1									
cx32.2like		2	2				3			2
cx32.3	1			1	1	1		1	1	
cx28.9	1			1		1		1	1	
cx28.1	1									
gjb1									1	
gjb1like		2	2				1			2
cx27.5	1			1	1	1				
cx31.7	1			1		1		1		
gjb2like		2	2				4			1
cx30.3	1			2	3	1		1	1	
cx35.4	1			1	1	1		1	1	
gjb3								1		
gjb3like		2	2	1			2			2
cx34.4	1			1	1	1		1	1	
gjb4			1							
gjb4like		4	3	2			4	1		4
cx28.6	1				1	1			1	
cx30.9	1			1	1	1		1	1	
gjb6like		1	1	1						1
gjb7		1	1				1			1
cx28.8	1			1	1	1			1	
gjc1		1	1	1			1	1	1	
gjc1like	1	3	3	1			3			4
gjc2		1	1				1			1
cx47.1	1			1	1	1		1	1	
cx44.2	1							1	1	

cx43.4	1			1	1	1		1	1	
gjd1a	1			1		1				
gjd2		1	1	1			2	1	1	1
gjd2like	2	4	4	2			3			4
gjd2b	1				1	1		1		
cx36.7	1			1	1	1		1	1	
gjd3		1	1		1	1			1	1
gjd3like							3			1
gjd4	1	1	1	1	1		1			1
gjd4like		1	1							1
cx23	1			1				1		
gje1		1	1			1			1	1
gje1like	1						1			

Suppl. Table 8. Percentages of amino acid identities between conserved domains in mammalian Cx39.2, including human “GJA4P”-NG_026166, and eel cx39.2 (one of the “gjd2like” sequences). The alignment is found in Suppl. Fig. 13A. The sequences are from eel (Aj), bats (Pv, Pa, Ra) and marsupials (wallaby, koala, opossum [Md]). Despite their different names, they all belong to the same orthologous group.

	1. Hs	2. Aj	3. Pv	4. Pa	5. Ra	6. Wallaby	7. Koala	8. Md
1. Hs-GJA4P-NG_026166	100.00	-	-	-	-	-	-	-
2. Aj-NN-cx39.2	54.92	100.00	-	-	-	-	-	-
3. Pv-NP-cx39.2	66.32	78.24	100.00	-	-	-	-	-
4. Pa-GJD2like-XM_006925175	66.32	78.24	100.00	100.00	-	-	-	-
5. Ra-GJC2like-XM_016138748	66.32	77.20	98.45	98.45	100.00	-	-	-
6. Wallaby-NP-cx39.2	59.07	79.79	81.87	81.87	81.87	100.00	-	-
7. Koala-GJA4like-XM_020963328	59.07	79.79	81.87	81.87	81.87	95.85	100.00	-
8. Md-GJD2like-XM_001376506	59.07	79.27	82.90	82.90	82.90	95.34	95.34	100.00

Suppl. Table 9. Human *GJA4P* is more similar to *GJD2like* (*connexin39.2*) than *GJA4* at nucleotide level.

Percentages of identity from full-length alignments of the nucleotide sequences between the presumed human *GJA4* pseudogene (NG_026166) with the presumed closest relatives *GJA4,* and *GJD2like* (= *connexin39.2*). The data show that NG_026166 had higher identity with the *GJD2like-cx39.2* group than with the *GJA4-cx37* group. The alignment was performed with Muscle (https://www.ebi.ac.uk/Tools/msa/muscle/). Hs, human; Md, opossum; Pa, black flying fox.

	1. Md-*GJA4*	2. Hs-*GJA4*	3. Pa-*GJA4*	4. Md-*GJD2like*	5. Hs-*GJA4P*	6. Pa-*GJD2like*
1. Md-*GJA4*-XM_007492764	100.0	-	-	-	-	-
2. Hs-*GJA4*-NM_002060	79.9	100.0	-	-	-	-
3. Pa-*GJA4*-XM_006924262	76.3	87.3	100.0	-	-	-
4. Md-*GJD2like*-XM_001376506	53.5	55.6	54.2	100.0	-	-
5. Hs-*GJA4P*-NG_026166	53.9	56.6	56.3	65.3	100.0	-
6. Pa-*GJD2like*-XM_006925175	54.9	57.6	56.4	67.7	79.4	100.0

Suppl. Table 10. Ohnology among teleost connexins. Ohnology is here functionally defined as being on different chromosomes, linkage groups or long scaffolds. For each main cell, the upper half shows the number of genes in the group for the different species (generally 1 or 2), while the lower half, which might be divided in two, shows the chromosome(s) or scaffolds (prefix, “sc”)where these genes locate. If the location is given as “1/1” or “2/2/2”, the genes are not ohnologs, but were generated by tandem gene duplication. Rand, scaffold/contig numbered “random”. Genes that have found in other assemblies or by other groups, but are not found in the chromosomal assembly, are marked with “no hit”.

Connexin group	Japanese eel		Herring		Zebrafish		Cod		Stickleback		Fugu		Tetraodon	
gja1	2[A]		2		2		2		1		1		1	
(43)	7[B]	19[B]	14	15	17	20	7	21	18		sc1725[C]		Rand	
cx34.5	1		1		1		1		1		1		1	
(32.7)	19		15		20		21		18		sc1917		14	
cx28.9	1		1		2		1		1		1		1	
(32.2)	19		15		20	20	21		18		sc1917		14	
cx32.2	1		2		2		1		1		1		1	
(32.2/32.3)	19		15	15	20	20	21		18		sc1917		14	
gja3	2		2		1		2		2		2		2	
	8	14	2	21	9		4	20	1	sc115[C]	1	8	2	3
cx39.9	2		2		1		3		2		2		2	
	8	15	8	20	5		7/7	10	4	7	14	15	1	7
gja4	2		1		1		1		1		1		1	
(39.4)	4	7	19		19		22		10		12		21	
gja5	2		2		2		1		2		2		2	
	8	14	2	21	1	9	no hit		6	16	1	4	7	17
gja8	1		2		2		1		1		1		1	
	8	14	2	21	1	9	20		16		1		2	
gja9	2		2		2		2		2		2		2	
(52.9/55.5)	7	sc68[C]	19	no hit	16	17	6	22	10	20	7	12	21	Rand
gja10	1		2		2		2		2		2		1	
(52.6/52.7)	19		14	15	17	20	5	no hit	18	sc128[C]	16	sc1843[C]	Rand	
gjb1	2		2		2		2		2		2		2	
(27.5/31.7)	8	15	20	no hit	5	14	7	10	4	7	14	15	1	7

cx30.3	2	3	1	2	2	3	4
(33.8)	8 14	2 8 21	9	4 20	1 sc115[C]	1/1 8	2/2/2 3
cx28.6	2	2	2	2	2	2	2
(30.9)	4 7	14 19	17 19	5 22	10 15	2 12	10 21
cx35.4	2	2	1	2	2	2	1
	4 7	14 19	17	5 22	10 15	2 12	10
cx34.4	2	2	1	2	2	2	1
	4 7	14 19	17	5 22	10 15	2 12	10
gjb7	1	1	1	1	1	1	1
(28.8)	19	no hit	20	21	18	sc1688[C]	14
gjc1	2	2	1	2	2	1	2
	1 18	1 1	3	2 18	5 11	5	2 3
gjc2	1	1	1	1	1	1	1
(47.1)	4	25	2	8	3	22	15
cx43.4	1	2	2	3	2	2	2
(44.2)	14	2 21	6 9	4 20 23	1 16	1 sc3571[C]	2 Rand
*gjd2*1*	2	2	2	2	2	1	1
	7 19	14 15	17 20	5 21	15 18	2	10
*gjd2*2/3*	2	1	2	2	2	2	2
	15 sc156[C]	9	5 15	7 16	1 7	15 11	7 16
gjd3	1	2	-	1	1	1	1
	1	1 1		18	5	1	Rand
gjd4	1	1	1	2	2	2	2
	5	17	24	2 23	3 21	10 22	15 Rand
cx39.2	1	2	1	1	2	1	1
	15	8 9	15	16	7 sc119[C]	15	7
cx36.7	1	1	1	1	1	1	1
	2	6	7	14	2	sc1921[C]	Rand
gje1	1	1	2	1	1	1	-
	19	14	17 20	21	18	16	-

A: The number of sequences in this group in this species.
B: The chromosomal location of the genes mentioned in the subcell above.
C: The scaffold has not been placed into a chromosome.
